# The mitochondrial genome and Epigenome of the Golden lion Tamarin from fecal DNA using Nanopore adaptive sequencing

**DOI:** 10.1186/s12864-021-08046-7

**Published:** 2021-10-07

**Authors:** Nicole Wanner, Peter A. Larsen, Adam McLain, Christopher Faulk

**Affiliations:** 1 Department of Animal Sciences, University of Minnesota, College of Food, Agricultural, and Natural Resource Sciences, 1988 Fitch Ave., Saint Paul, MN 55108 USA; 2 Department of Veterinary and Biomedical Sciences, College of Veterinary Medicine, University of Minnesota, Saint Paul, MN USA; 3 Department of Biology and Chemistry, College of Arts and Sciences, SUNY Polytechnic Institute, Utica, NY USA

**Keywords:** Mitochondria, DNA methylation, DNA hydroxymethylation, Poop, Primates, Lion Tamarin

## Abstract

**Background:**

The golden lion tamarin (*Leontopithecus rosalia*) is an endangered Platyrrhine primate endemic to the Atlantic coastal forests of Brazil. Despite ongoing conservation efforts, genetic data on this species remains scarce. Complicating factors include limitations on sample collection and a lack of high-quality reference sequences. Here, we used nanopore adaptive sampling to resequence the *L. rosalia* mitogenome from feces, a sample which can be collected non-invasively.

**Results:**

Adaptive sampling doubled the fraction of both host-derived and mitochondrial sequences compared to sequencing without enrichment. 258x coverage of the *L. rosalia* mitogenome was achieved in a single flow cell by targeting the unfinished genome of the distantly related emperor tamarin (*Saguinus imperator*) and the mitogenome of the closely related black lion tamarin (*Leontopithecus chrysopygus*). The *L. rosalia* mitogenome has a length of 16,597 bp, sharing 99.68% sequence identity with the *L. chrysopygus* mitogenome. A total of 38 SNPs between them were identified, with the majority being found in the non-coding D-loop region. DNA methylation and hydroxymethylation were directly detected using a neural network model applied to the raw signal from the MinION sequencer. In contrast to prior reports, DNA methylation was negligible in mitochondria in both CpG and non-CpG contexts. Surprisingly, a quarter of the 642 CpG sites exhibited DNA hydroxymethylation greater than 1% and 44 sites were above 5%, with concentration in the 3′ side of several coding regions.

**Conclusions:**

Overall, we report a robust new mitogenome assembly for *L. rosalia* and direct detection of cytosine base modifications in all contexts*.*

**Supplementary Information:**

The online version contains supplementary material available at 10.1186/s12864-021-08046-7.

## Introduction

The golden lion tamarin (*Leontopithecus rosalia*) is an endangered Platyrrhine primate endemic to the Atlantic coastal forests of Brazil [1]. It is a member of the family Callitrichidae, a taxonomic grouping that includes marmosets, tamarins and lion tamarins [2]. Although once teetering on the brink of extinction, golden lion tamarins have benefitted from a successful captive breeding and reintroduction program that has seen their numbers climb from a few hundred individuals in the wild to several thousand. In addition to wild individuals, several hundred animals are maintained in captivity globally as part of the captive breeding and reintroduction program, and stud books are maintained to promote genetic diversity in these animals and avoid inbreeding [3]. Golden lion tamarins are gregarious, living in social groups typically centered around a monogamous breeding pair and their dependent infants, juveniles and subadults [4]. Golden lion tamarins also play an important ecological role as seed dispersers [5]. Genetic data from *L. rosalia* remains scant, however. Increased availability of genetic data will benefit breeding and conservation efforts for this species.

Mitochondria are the powerhouse of the cell and contain their own genome [6]. The mitogenome in animals is small, circular, and mutates more rapidly than the nuclear genome. Two characteristics make it especially useful for phylogenetic comparisons and non-invasive sampling. First, it is present in many more copies per cell than the nuclear genome, making it easier to recover from degraded samples such as feces or ancient DNA. Second, its higher mutation rate enables more accurate delineation of closely related species. The first mitochondrial sequences of *L. rosalia* were published in 2008 and 2011 and were limited to the sequences of cytochrome b and the hypervariable region of the displacement control region (D-loop) [7, 8]. A more complete version of the mitogenome became available in 2013, but it contained gaps and shared surprisingly limited homology with the closely related black lion tamarin (*L. chrysopygus*) with only 96% shared identity [9]. To create a more accurate comparison of the *L. rosalia* mitogenome to its sister species, we chose to resequence its mitochondrial genome to high coverage using a novel technique with easily collected fecal samples.

Nanopore sequencing is based on electrical signals generated by cylindrical protein pores as nucleic acids pass through [10]. The MinION sequencer from Oxford Nanopore Technologies (ONT) is field-portable with minimal reagent requirements. It can provide long reads with an average read length of 20 kb and occasional single reads over 1 Mb in length, limited only by the molecular weight of the input DNA. Since mitochondria have ~ 16 kb genomes, read lengths are sufficient to cover the entire mitogenome. However, in fecal samples, the mixture of DNA sources presents a challenge to sequencing.

Traditionally, targeted sequencing of specific loci is performed by enzymatic or PCR enrichment prior to sequencing [11]. Recently, a form of computational enrichment called adaptive sampling has been developed [12]. This method allows for sequencing of whole genomic DNA combined with locus-specific enrichment by rejecting off target reads. Crucially, adaptive sampling can enrich target regions up to 30-fold, dependent on sequence length and percentage of the genome, enough to bring target loci to high enough coverage for analysis. Here, we employed adaptive sampling in order to obtain sufficient coverage of the mitogenome for accurate assembly.

From an epigenetic perspective, mitochondrial DNA methylation has been a matter of debate [13]. Determining its methylation pattern has been challenging due to unique characteristics including resistance to bisulfite transformation, which is used in nearly all epigenetic methods [14]. Direct sequencing of native genomic DNA preserves base modifications which can then be detected with nanopore sequencing. Neural network models are capable of providing simultaneous calls of nucleic acid sequence and base modifications from unenriched genomic DNA input [15]. Here we focus on 5’methylcytosine at both CpG and non-CpG sites. Other DNA modifications such as 6 N-adenine (m6A) are present in the human genome and mitochondria [13, 16]. Theoretically, m6A could be detected from the raw signal by future neural network models, however, none are currently able to accurately detect it. Due to the recent availability of models for calling DNA 5’hydroxymethylcytosine, we are the first to report native detection at base-pair resolution in mitochondria by sequencing. The combination of adaptive sampling, long-reads, and epigenetic information, drove our selection of the Oxford Nanopore MinION to resequence the mitogenome of *L. rosalia*.

Fecal DNA serves as a rich source of host information that can be collected non-invasively from wild populations and processed for DNA extraction in the field [17]. Fecal microbiomes from nanopore sequencing have been the subject of multiple studies [18–20], however, host DNA enrichment by nanopore sequencing from fecal samples has not yet been documented. Due to degradation of DNA by digestive processes, choosing high abundance targets such as mitochondria naturally increases their sequencing frequency at the cost of sequence read length.

We describe an improved assembly of the mitogenome of the golden lion tamarin extracted from a fecal sample. We find that this species is most closely related to the black lion tamarin based on the high level of sequence identity. This finding is consistent with prior taxonomic studies using genetics and morphology, which found the two species to be closely related [7, 21, 22]. We confirm the pattern of diverging mutations falls mostly within the non-coding D-loop. Our method resulted in 285x coverage from a single flow cell. The use of a ‘high accuracy’ neural network model with 97.8% modal accuracy in base calling allows high confidence in the consensus. Sequence polishing, error-correcting, and assembly resulted in a circular contig of 16,597 bp in length. We directly measured DNA methylation and hydroxymethylation levels from the nanopore signal level read data using the Megalodon tool provided by ONT. We find that DNA methylation is negligible across the entire mitogenome, in line with human mtDNA [6]. Surprisingly, we find elevated levels of DNA hydroxymethylation only in the CpG context, suggesting biological function of this mark in mitochondria. We conclude that fecal samples provide a rich source of host DNA suitable for nanopore sequencing, providing a new capability for field use in conservation research.

## Results

### Fecal DNA yields a large quantity of highly fragmented DNA

Wild-collected feces represent an abundant supply of host genomic, metabolomic, and metagenomic information. We collected a fresh, wet fecal sample from a captive golden lion tamarin at the Utica Zoo. Two kit-based methods of DNA extraction were used with manufacturer protocols designed to enrich the host DNA fraction and yielded similar results. A total of 9 μg of DNA was derived from 400 mg of fecal material and used for library prep and nanopore sequencing. Library prep with 2 μg input yielded between 200 and 700 ng of product per reaction. Mean read length from untargeted nanopore sequencing was 1230 bp, indicating short fragment length likely due to digestive degradation.

### Nanopore adaptive sampling enriches host DNA from feces

A nanopore MinION flowcell was loaded with 500 ng of DNA and sequenced for 24 h. Since over 99% of fecal DNA is derived from microbiome and digesta, enriching the small fraction of host-derived fragments makes better use of limited sequencing capacity. We used adaptive sampling to enrich for sequences that matched to the unfinished genome of a distantly related species, *Saguinus imperator* (emperor tamarin). This target genome consists of 3.4 Gb total sequence divided into 1,666,189 scaffolds. *S. imperator* was chosen over the more closely related *Leontopithecus chrysopygus* (black lion tamarin) since the latter did not have an available nuclear genome.

Our initial sequencing run yielded 15 million reads containing 6.3 Gb of sequence with a mean read length of 411 bp, indicating that adaptive sampling was rejecting most reads in less than 1 s since the read rate averaged over 400 bases/s. All base-called sequence was kept, whether or not it is from a rejected strand, and used for analysis. We identified 0.8% of reads matching *S. imperator*, derived from the fecal sample of *L. rosalia* DNA, compared to 0.39% without enrichment, therefore adaptive sampling was successful in enriching host tamarin sequences (Table 1).

**Table 1:** Mapping statistics for Control and Adaptive Sampling Runs

	Control run (1 h) on total fecal DNA, no enrichment		Adaptive Sampling for 24 h, enriching to *S. imperator* scaffold genome		2nd 24 h run enriching to *S. imperator* scaffold + *L. chrysopygus* mitogenome	
	Total Reads	Alignments to *L. chrysopygus* mitogenome	Total Reads	Alignments to *L. chrysopygus* mitogenome	Total Reads	Alignments to *L. chrysopygus* mitogenome
Number of Sequences	133,852	18	15,346,306	2810	13,362,519	2448
Total Length (bp)	164,604,258	25,700	6,308,923,013	1,990,083	5,683,370,590	3,187,574
Average Length (bp)	1230	1428	411	708	425	1302
Mb aligned to *S. imperator*	649,354		50,352,101		46,619,227	
% Aligned to *S. imperator*	0.39%		0.80%		0.82%	
MtDNA hits per Mb		0.11		0.45		0.43
mtDNA Mb / Total Mb		0.016		0.032		0.056
mtDNA to host nuclear DNA	3.96%		3.95%			6.84%

### Mapping mitochondrial reads

To identify mitochondrially derived reads, we initially mapped the total read set to the previously reported *L. rosalia* reference mitogenome (NC_021952). We found 4585 matching reads with a mean length of 1104 bp. However, the alignment revealed a high number of SNPs, gaps, 3′ artifacts, and low fidelity to the existing reference. Our resulting contig had 96.83% identity across only 92% of the query length to *L. rosalia* NC_021952, quite unexpectedly divergent for members of the same species. An unusually large number of reads were mapped to a small, disconnected fragment on the 3′ end of this reference (Fig. S1). To determine whether these were sequencing artifacts or errors in the reference, we aligned our reads to the closely related *L. chrysopygus* mitogenome (accession NC_037878) [23] which yielded fewer reads (2810) of shorter average length (708 bp) but much more uniform coverage. Our resulting contig had 99.86% identity over > 99% of the length of this mitogenome without gaps, suggesting it would provide a better reference for further analyses.

### Mitochondrial DNA is enriched by adaptive sampling

To determine whether adaptive sampling enriched mitochondrial DNA along with nuclear DNA, we compared reads from a non-adaptively sampled control run vs. the first 24 h of an adaptive sampling run targeting the *S. imperator* scaffold genome. We found a 4-fold increase in number of mtDNA sequences per Mb when mapping to the *L. chrysopygus* mitogenome from the *S. imperator* enriched reads (Table 1). By read percentage, host mtDNA doubled from 0.016 to 0.032% of the total fecal DNA, precisely mirroring the doubling seen in total host genomic DNA enrichment. Nanopore adaptive sampling revealed no differences between mitochondrial and host nuclear DNA ratio, with mtDNA making up 3.95% of the host DNA in both. This result was expected since we enriched for an entire tamarin genome, not just mtDNA specifically.

### Higher quality targets increase enriched read length but not sampling efficiency

Next, we explored whether adaptive sampling efficiency could be improved by adding a more accurate target for enrichment. The *S. imperator* genome initially used for adaptive sampling contains fragments covering the entire *S. imperator* mitogenome but with very poor quality. When assembled and compared against the high-quality *L. chrysopygus* mitogenome, the two share only ~ 70% sequence identity, indicating many errors. In contrast, *L. chrysopygus* matches 95% to the existing *L. rosalia* mtDNA (NC_021952) and > 99% to the *L. rosalia* mitogenome that we ultimately assembled. We reasoned that inclusion of the *L. chrysopygus* mitogenome along with the *S. imperator* full genome might improve adaptive sampling efficiency since it would share much higher sequence identity with *L. rosalia* mtDNA. A second aliquot of the same library was run on the MinION for an additional 24 h, with both the *S. imperator* scaffold genome and *L. chrysopygus* mitogenome as enrichment targets, resulting in 5.6 Gb of additional reads.

**Fig. 1:**
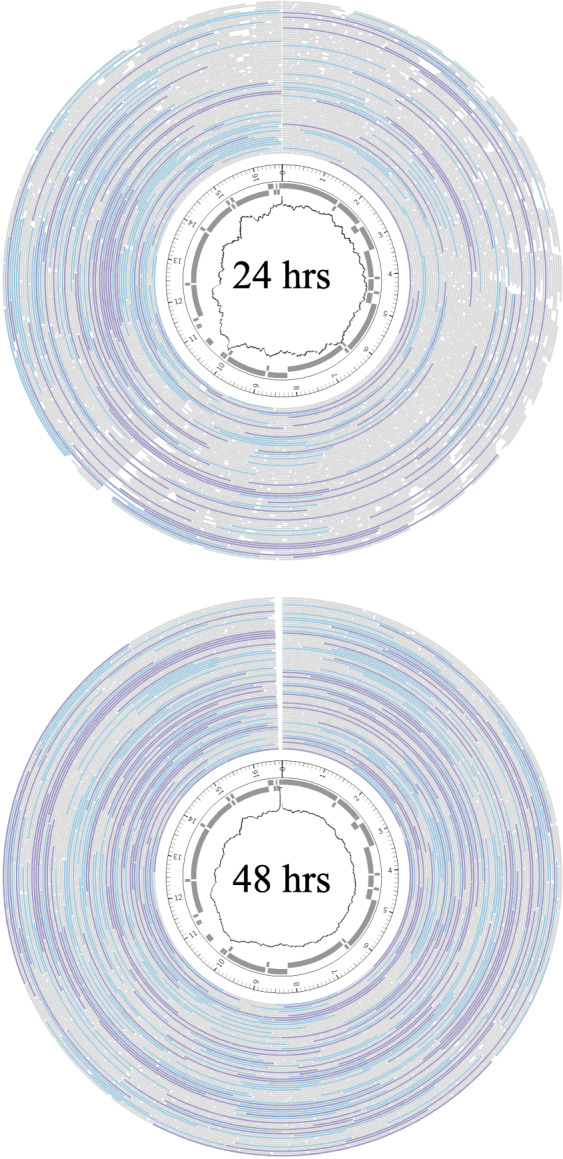
Coverage density of the mitogenome. Outer layer shows mapped reads where > 2 kb are purple, 1-2 kb are blue, < 1 kb are gray. Inner layers show gene position and coverage. For the first 24 h run (**A**), the minimum coverage is 52x, at the D-loop, maximum = 168x at the large subunit-rRNA. For the combined data over 48 h (**B**), minimum coverage is 169x at the D-loops, and maximum = 392x at the large subunit-rRNA

In the second run, reads matching the *L. chrysopygus* mitogenome were 1302 bp in length, double the 708 bp read length of initial run and is a result of the improved matching to the more closely related and better quality *L. chrysopygus* mitogenome target. This improved length did not result in greater overall enrichment, however, as enrichment remained at 4-fold increase over background. Increased read length did result in 60% greater coverage of the mitogenome, improving contig coverage from 106x in the first run to 179x in the second run. Read length improvement and coverage is illustrated in Fig. 1. The region of lowest coverage was the D-loop, while the nearby large subunit rRNA had the highest average coverage. Interestingly, in the second run the ratio of mtDNA to host nuclear DNA nearly doubled from 3.95 to 6.83% due to the increased read length from having a more accurate mitogenome target.

### Nanopore adaptive sampling tolerates large target enrichment sequence divergence

Both rounds of adaptive sampling resulted in 4-fold improvement in mitogenome coverage vs. non-targeted sequencing, in line with Oxford Nanopore’s guidance. Since there was only 70% homology between the available *S. imperator* mtDNA assembly fragments and our reconstructed *L. rosalia* mitogenome, that indicates adaptive sampling can tolerate at least 30% sequence identity divergence from the target while still enriching as highly as compared to a perfectly matching target. For subsequent analyses, we combined reads from both runs.

### Mapping reveals high coverage of host mitochondrial DNA

Overall, there were 5516 reads aligning to *L. chrysopygus* mtDNA with a mean length of 984 bp, resulting in 285x read coverage with improved continuity particularly at the 3′ end (Fig. S1). Assembly of reads with Flye yielded a circular draft contig of 16,592 bp with 99.86% identity to *L. chrysopygus* over 99% of its length. This draft assembly contained several indels proximal to homopolymeric regions as seen in other reports of unpolished nanopore-generated contigs, suggesting polishing could improve the sequence quality [24]. When our unpolished contig was compared to the existing *L. rosalia* (NC_021952) reference our assembly matched at only 96.83% identity for 92% of its length, representing a strong improvement in both measures. The next nearest BLAST match in the NCBI nucleotide database was Goeldi’s marmoset (*Callimico goeldii*) at 85.18% identity with 97% query length coverage.

### Polishing of the assembly

Identification of open reading frames was performed with MITOS2 and revealed that the indels in the draft assembly were causing multiple frameshift mutations. For polishing, both the Oxford Nanopore tool Medaka and the 3rd party tool Nanopolish were compared with the former outperforming the latter. Medaka eliminated all frameshifts and was used for the final assembly. Comparison of the draft and polished assemblies revealed 5 indels all proximal to homopolymeric regions and 1 SNP, with identity to *L. chrysopygus* dropping the match to 99.63% for > 99% of its length. This is a substantial improvement compared to the existing *L. rosalia* reference which matched to the polished contig at 95.95% identity for 95% of its length. MITOS2 annotation of the polished assembly found that all frameshift mutations were resolved, and no manual intervention was required. The polished assembly was rearranged to place *COX1* at the start position and submitted to NCBI under accession number MZ262294.

### Mitogenome organization is highly conserved with mutations preferentially in the D-loop

**Fig. 2:**
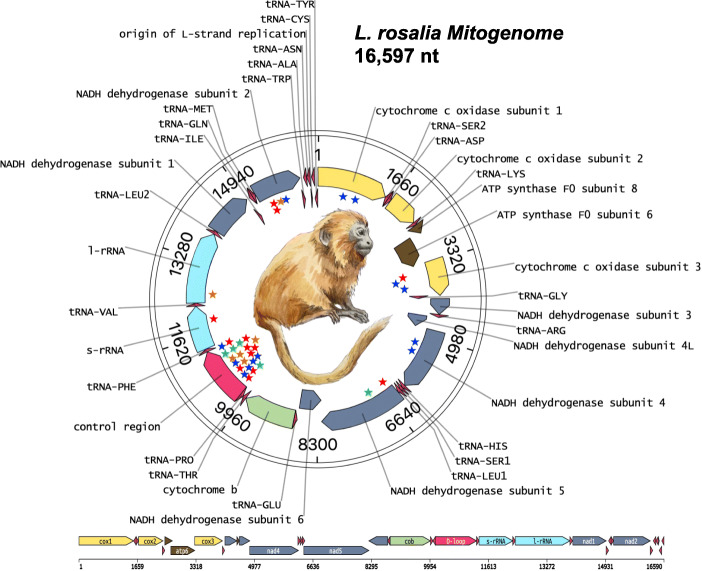
The mitogenome of the Golden Lion Tamarin. The *L. rosalia* mitogenome is 16,597 bp in length. Stars represent SNPs in comparison to the closely related Black Lion Tamarin, *L. chrysopygus.* Star colors indicate base change where red = “T”, blue = “C”, orange = “G”, green = “A”. The majority of SNPs are in the D-Loop control region

The mitogenome of *L. rosalia* has a total length of 16,597 bp (Fig. 2). Our assembly is shorter than the previous reference by 275 bp, and is very similar in size to the closely related *L. chrysopygus* (16,618 bp) [23]. Coding regions are nearly identical to *L. chrysopygus* with 2 amino acid changes in *NADH* and none in any other. Gene order is the same as in humans and other primates. We then investigated mutations that accrued since the divergence of *L. rosalia* and *L. chrysopygus*. A total of 38 SNPs are mapped on Fig. 2. As expected, the majority of SNPs are concentrated within the non-coding D-loop. Only 16 SNPs are located throughout the rest of the genome.

### DNA methylation is negligible in mitochondria at CpG sites

**Fig. 3:**
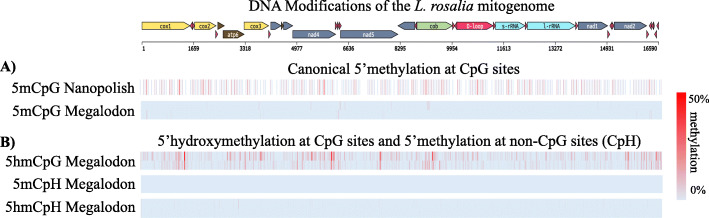
DNA modifications of the *L. rosalia* mitogenome. Cytosine modifications are shown mapped to the mitogenome of *L. rosalia*. **A**) 5’methylation is shown by Nanopolish and Megalodon. **B**) 5′ hydroxymethylation at CpG and CH sites is shown with Megalodon. Each lane is illustrated with replicates at top and bottom from the first and second runs respectively. Hydroxymethylation in the CpG context has the highest prevalence and replication. Percentage of total modified base reads are shown from blue to red

Native genomic DNA contains modifications of interest in epigenetic analyses. We chose a sequencing library preparation kit that excludes PCR steps in order to preserve these modifications for downstream analyses. As others have reported both the presence and absence of DNA methylation in mitochondria using a variety of methods, we sought to resolve this controversy by leveraging nanopore’s ability to directly detect DNA modifications. First we used the Nanopolish package to detect 5’methylation (5mC). Nanopolish was able to call methylation at 259 of the 642 CpG sites in the forward strand only (Fig. 3A). Methylation was called for sites with more than 40 reads (average 160 reads) by Nanopolish. Of these, 29 sites had > 10% methylation and only 3 sites had > 20% methylation. Nearly three quarters, 189 sites, had < 5% methylation. Both technical replicates were combined to generate sufficient coverage for basecalling (Table 2).

**Table 2:** DNA 5’methylation by Nanopolish

	Methylation					
5mC CpG	Total Sites	> 1%	> 5%	> 10%	> 20%	Coverage
	259	189	68	29	3	160x

To validate and expand these findings, we basecalled methylation again using the ONT package Megalodon as it can call both 5mC and 5hmC modifications simultaneously. It uses a neural network model trained on modified cytosines in all contexts, whereas Nanopolish uses a hidden Markov model approach that only supports 5mC and is less accurate on natural samples containing hydroxymethylation [25]. Megalodon called substantially lower 5mC at all 642 CpG sites in both strands. Due to increased calling efficiency, the first and second 24-h runs were analyzed separately as technical replicates. Across CpG sites within the mitogenome, DNA methylation > 1% was seen in just 13 of the 642 total sites, and none of these had greater than 0% in the technical replicate. Only 2 CpG sites were found to have > 5% methylation, and neither was above 0% in the technical replicate. Read coverage was 40x and 63x for each replicate respectively (Table 3). We found no significant correlation in between forward and reverse strand methylation levels at any threshold.

**Table 3:** DNA modification summary by Megalodon

		> 1% methylation			> 5% methylation			Coverage	
	Total Sites	1st run	2nd run	# replicated	1st run	2nd run	# replicated	1st run	2nd run
5mC CpG	642	11	13	0	2	2	0	43x	63x
5hmC CpG	642	328	309	196	124	67	44	43x	63x
5mC CH	5469	0	0	0	0	0	0	49x	73x
5hmC CH	5469	183	189	18	4	0	0	49x	73x

### DNA hydroxymethylation is moderate at CpG sites

In contrast to 5mC, mitochondrial hydroxymethylation greater than 1% was called at nearly half the 642 CpG sites, with 196 of these replicating between runs (Fig. 3B). When filtering for hydroxymethylation greater than 5%, 44 sites were still detected and replicated between runs. At 4 sites 5hmC exceeded 20% in both replicates. Interestingly, 5hmC is concentrated on the 3′ side of several genes including *COX1*, *COX3*, *NAD4*, and *NAD5* indicating a non-uniform distribution and likely biological function.

### Cytosines outside of CpG context contain no base modifications

In non-CpG context, identified by the dinucleotide ambiguity code CH where H stands for “any nucleotide except G”, no cytosines were methylated in any of the 5469 possible CH sites. For hydroxymethylation, we found 3.4% of sites with detectible levels above 1%, however, only 18 of these sites replicated. At the 5% threshold, no replication remained. Taken together these findings indicated strong support for the presence of hydroxymethylation in the CpG context, with neither methylation nor hydroxymethylation present outside of CpG sites. (Supplementary File 1).

## Discussion

The purpose of this study was to selectively enrich and sequence host DNA from a non-invasively collected sample using nanopore adaptive sequencing, assemble a mitogenome, and assess for epigenetic modifications. We chose the golden lion tamarin, *L. rosalia,* as it represents an endangered primate with an unsequenced genome and a mitochondrial genome in need of improvement. The fraction of host DNA sequenced from feces was less than 1% of the total reads in line with other studies [26], but this fraction was doubled with the use of adaptive sampling. Remarkably, our coverage of the mitogenome was high enough to accurately assemble a full length contig. These findings suggest that mitogenomes make the best target for nanopore sequencing from fecal DNA.

Recently, the mitogenome of the related Brazilian buffy-tufted-ear marmoset (*Callithrix aurita*) was assembled by genomic skimming with a nanopore sequencer [27]. Genomic skimming uses low coverage sequencing and leverages naturally high copy number sequences (e.g. repeats, and mitogenomes) to assemble high coverage of those regions. It had high concordance with a Sanger sequenced replicate, however it resulted in only 9x coverage of the mitogenome and required two full flow cells as opposed to our 285x coverage with a single flow cell. Closer to our target species, de Freitas et al. recently used short-read sequencing to generate a high quality mitogenome of *L. chrysopygus* with over 3000x coverage, providing us with an accurate reference for our nanopore adaptive sampling method [23]. That mitogenome required bootstrapping alignments with manual k-mer adjustments, whereas our long-read pipeline assembled an error-free mitogenome despite lower coverage. Both methods leverage the ability to use related species as alignment targets.

The enrichment of mitochondrial DNA was surprising despite its overabundance compared to nuclear chromosomes. Generally, the ratio of mtDNA to nuclear DNA is about 0.1%, despite containing hundreds to thousands more copies per cell in most somatic tissues [28]. Here we found a 40-fold increase in this ratio, at 3.96% mtDNA to nuclear DNA. By including an accurate copy of the *L. chrysopygus* mitogenome, we increased that ratio to 6.84%, doubled the length of aligned reads, and quadrupled the hits per megabase of reads. Our increased efficiency may be a product of the sample source. Bulk stool contains highly fragmented digested DNA, and the described method may not hold for less degraded mitochondrial DNA. Nanopores draw in only linearized DNA, therefore any mtDNA molecules must be degraded enough to lose their native circular conformation before sequencing. Even though we did not use any double strand break treatments prior to sequencing (e.g. enzymatic treatment, sonication, hydrodynamic shearing), we still sequenced 285-fold coverage of the mitogenome, as compared to less than 0.1x of the nuclear genome.

Oxford Nanopore guidance indicates that longer targets and read lengths are correlated with higher enrichment. Our lower level of enrichment (4-fold) over non-enriched sequencing is likely due to short fragment lengths. Indeed, when sequencing human mitochondrial DNA from high molecular weight libraries from liver cells, Goldsmith et al. found reads averaging over 80% of the entire mitogenome [6]. Meanwhile, adaptive sampling yields best results with targets greater than 15 kb and read lengths greater than 10 kb [12]. A second reason for our low level of enrichment was the poor match of the emperor tamarin mtDNA sequences. With the better *L. chrysopygus* match our aligned reads lengthened to 1300 bp, slightly longer than the average read length of the control run with no rejection taking place. This result indicates that DNA fragmentation in the feces was the limiting factor.

The presence of mitochondrial pseudogenes in the nuclear genome did not appear to bias our results, given the strong concordance of our assembly to the *L. chrysopygus* mtDNA which was created with careful emphasis to eliminating nuclear coded mt-pseudogenes [23]. The coding structure of our assembly mirrors the *L. chrysopygus*, with SNPs primarily in the D-loop. SNP accumulation in the D-loop was expected due to its non-coding status.

Our full sequence of the *L. rosalia* mitogenome shares 99.63% sequence identity with the published *L. chrysopygus* mitogenome, indicating very recent divergence of these sister species. Moreover, the assembly fits better with the known phylogenetic distances within Callitrichidae. The sister taxa relationship between these species had been under debate until 2001 when photoreceptor intron sequences unambiguously established their relationship [21]. In, 2008 the first mitochondrial sequences, of the D-loop, confirmed their close relationship [7]. We extend this evidence by showing near perfect synteny throughout the coding regions.

The presence of mitochondrial DNA modifications has long been debated. Here we report essentially zero DNA methylation at every CpG position in the mitogenome in stark contrast to other reports of low but consistent methylation in mtDNA. Mitochondrial methylation using a nanopore sequencer has been reported by at least four groups [6, 29–31]. All of these studies found low but measurable mtDNA methylation, averaging less than 7% across a variety of conditions and cell types, though a few specific CpG sites had over 20%. These studies all used Nanopolish which does not distinguish 5mC from 5hmC. Since bisulfite-based methods also cannot distinguish 5mC from 5hmC, they are similarly prone to overestimating the level of 5mC. It is also well-known that bisulfite does not transform circular DNA with high efficiency, again leading to an overestimate of 5mC [32]. Nanopore alleviates these concerns by avoiding bisulfite conversion, and all DNA molecules sequenced through a pore are linearized by necessity. Here we used Megalodon’s neural network model capable of detecting 5hmC to avoid Nanopolish’s 5mC bias. In support of our findings, a recent careful analysis of mitochondria methylation by Bicci et al. validated nanopore sequencing across multiple primary and cancer cell lines [30]. When accounting for all confounders, they found negligible DNA methylation as we have here. We suggest that previous findings of mitochondrial 5mC were artefacts of either bisulfite or miscalled nanopore basecalling.

We found very high levels of 5’hydroxymethylation within the mitogenome in the CpG context. Interestingly, the most well-cited work on mitochondrial base modifications by Shock et al. also reported 10x greater levels of 5hmC than 5mC, using antibody-based methods [33]. Biological function of 5’hydroxycytosine is strongly suggested for two reasons. First, we found no non-CpG modifications at all. This argues for reader proteins capable of recognizing cytosines in dinucleotide context rather than these modifications resulting from non-specific oxidation processes modifying cytosines by chance. Interestingly we saw no correlation in 5hmC between strands, indicating a non-palindromic oxidation process. This stranded-ness in mitochondrial base modification was seen by Dou et al., though they used bisulfite methods and called it as 5mC [34]. Second, we see enrichment on the 3′ side of several genes, showing non-uniform distribution which is a hallmark of function [35]. Taken together, our data suggests a specific biological function of 5hmCpG in mitochondria.

Epigenetic data is challenging to recover from wild species but has been done in hyenas and is expanding to other vertebrates [36, 37]. Therefore, our study serves two purposes. First, it confirms the ability to detect base modifications by nanopore sequencing of mitogenomes [14]. Second, it provides proof-of-concept that epigenetic analyses can be performed on fecal derived samples, which is applicable to both host nuclear genome methylation and the fecal microbiome, even potentially including food species in digesta.

It is important to consider some caveats to our method. Nanopore accuracy is still low relative to short-read and Sanger sequencing, though neural network models are improving at a rapid pace [15]. Polishing helps as indicated by Medaka’s ability to removing all frame shift inducing indels from our assembly. Modified base calling is also improving rapidly and is also dependent on models trained preferably for a specific modification and with taxa-specific data.

Interest in long-read sequencing of vertebrate mitogenomes continues to increase. A study by Formenti et al. recently sampled 100 species and corrected many long-standing errors in reference mitogenomes [38]. However, their approach is unsuitable for the most easily available tissue, fecal DNA, since the first step is to remove short fragments less than 10 kb. Here we show the strength of short fragments and long-read technology together to generate accurate assemblies and epigenomes useful for comparative genomics [39]. Accurate genomic resources are critical to the conservation of endangered species like the Golden Lion Tamarin.

## Materials and methods

### Sample collection

A single fecal sample weighing 10 g was collected 10 min post defecation from the environment of the two golden lion tamarins at the Utica Zoo The sample was witnessed as being deposited by the female of a pair, Arie, who was 6 years old at the time of collection. The sample was collected under the approval of the Utica zoo. Fecal samples are exempt from IACUC protocol approval at the University of Minnesota.

### DNA extraction

Qiagen QIAamp DNA stool mini kit (cat no. 51504) was used with the protocol, “Isolate of DNA from Stool for Human DNA Analysis” supplied by manufacturer. This kit yielded approximately 4 μg of purified DNA from 200 mg of fecal sample. Additionally, the Omega Bio-tek E.Z.N.A. Stool DNA kit (cat no. D4015–00) was used also with 200 mg of stool and manufacturer’s protocol, “DNA Extraction and Purification from Stool for Human DNA Detection” and yielded a similar quantity of DNA. We used two kits since the Qiagen kit is no longer manufactured and its replacement is twice the price of the Omega Bio-tek kit which yielded similar quantity and quality.

DNA from both kits were combined and library prep was performed using the Oxford Nanopore Technologies (ONT) SQK-LSK109 “Genomic DNA by Ligation” protocol with the following changes: 1) NEBNext products were used for end-repair as suggested by ONT (cat no. M6630, E7546, and E6056), 2) Axygen AxyPrep Mag PCR Clean-up beads were substituted for Agencourt AMPure beads, 3) magnetic beads were optionally diluted to 25% of original volume with in-house prepared carboxy bead dilution buffer (https://bomb.bio/protocols/) for cost savings with no loss in recovery efficiency [40].

### Nanopore adaptive sequencing

Sequencing was conducted on a single MinION sequencer on a FLO-min106 flow cell with pore chemistry R9.4 for two runs of 24 h each. Prior to the first run, the cell was run for 1 h without adaptive sampling, providing control data. Between 24 h runs the flow cell was washed with nuclease from the Flow Cell Wash Kit (WSH004) and loaded with storage buffer. A second aliquot from the same initial library prep was used for the second run. Live basecalling was performed using the fast basecalling model in ONT basecalling software, Guppy v4.5.4, GPU enabled with a GeForce RTX 2080Ti. Adaptive sampling was enabled in MinKNOW core v.4.2.5 and set to enrich sequences matching to the *Saguinus imperator* (emperor tamarin) genome, SagImp_v1_BIUU (accession PRJNA399417), scaffold assembly. After sequencing, reads were basecalled again with Guppy using the high accuracy model and these reads were used for all subsequent analyses. Basecalled reads were mapped using Minimap2 [41] to the *L. rosalia* (NC_021952) and *L. chrysopygus* (NC_037878) mitogenomes. Resulting bam files were sorted and indexed using samtools and converted to bed files for viewing in IGV with bamToBed from bedtools [42, 43]. Visualization of read statistics was performed with Bamstats (http://bamstats.sourceforge.net).

### Mitogenome assembly

Assembly proceeded in two steps. We used the total reads aligning to *L. chrysopygus* mtDNA in fasta format to generate the assembly. Flye v2.8.3 was used for draft assembly and first round polishing of the initial consensus [44]. Flye has an expected error rate of 0.5 to 1% for ONT reads and therefore was followed by an additional polishing step. Medaka v1.3.2 uses neural network models applied to a pileup of individual sequencing reads against a draft assembly to improve consensus sequences. In our testing, it outperformed Nanopolish in generating a contig without frameshift indels in the final contig [25]. Both packages were used with default settings.

### Circularization and annotation

Flye generated a circularized assembly, i.e. no repeats on the ends, ready for annotation. Identification of protein coding regions was performed through the MITOS2 website (http://mitos2.bioinf.uni-leipzig.de). Start position was manually set at the *COX1* gene following convention. The genome was visualized in Open Vector Editor (https://github.com/TeselaGen/openVectorEditor) and submitted to NCBI.

### DNA methylation analyses

Nanopolish calls methylation uses raw signal data with a hidden Markov model. The Nanopolish call-methylation function was applied on the same reads used for consensus building. Site-specific methylation percentages were determined using the calculate_methylation_frequency.py helper script.

Megalodon extracts high accuracy modified bases and variant calls from raw nanopore reads by using intermediate output from the neural network model provided by the basecaller Guppy. We used Megalodon v2.3.1 with the Guppy v4.6.4. The following research model from Rerio repository, res_dna_r941_min_modbases_5mC_5hmC_v001.cfg, was used as it is able to call both 5mC and 5hmC modifications at cytosine sites in any context. The “--mod-binary-threshold = 0.8” flag was set to slightly decrease the stringency of Megalodon’s modification calling in line with suggested practices [6]. Further details are in supplementary file 2.

## Supplementary Information

**Additional file 1: Fig. S1.** Alignment of reads to *L. rosalia* from the first 24 h run to the reference mitogenome (KC757399) has 96.83% contig identity over 92% of its length, with 5′ gaps (top). Alignment to *L. chrysopygus* reference (NC_037878) improves to 99% with complete coverage over the full length (bottom). The 5516 reads are combined from 2810 (1st run) and 2706 (2nd run).

**Additional file 2: Supplementary File 1.** Supplementary Methods.

**Additional file 3: Table S7.** 5′ methylcytosine CpG calls from Nanopolish.

**Additional file 4: Supplementary File 3.** Consensus sequence of *L. rosalia* mitogenome in fasta format.

**Additional file 5: Supplementary File 4.** Alignment reads used for mitogenome assembly.

## Data Availability

The mitogenome sequence has been deposited into NCBI GenBank with accession number MZ262294 and is available as supplementary file 3. Reads used to generate this assembly are available as supplementary file 4. Prior tamarin sequences were sourced from NCBI Genbank which is an open public access repository [https://www.ncbi.nlm.nih.gov/genbank/].

## Abbreviations

CpG: Cytosine-phosphate-guanine
ONT: Oxford Nanopore Technologies
5mC: 5’methylcytosine
5mCpG: 5’methylation at CpG site
5hmC: 5’hydroxymethylcytosine
5hmCpG: 5’hydroxymethylation at CpG site
CH: Cytosine plus any non-G base

## References

[1] Rylands AB, Kierulff MCM, de Souza Pinto LP, Kleiman DG, Rylands AB (2002). Distribution and status of lion tamarins. Lion Tamarins: biology and conservation.

[2] Groves CP (2001). Primate Taxonomy.

[3] Kierulff MCM, Ruiz-Miranda CR, de Oliveira PP, Beck BB, Martins A, Dietz JM, Rambaldi DM, Baker AJ (2012). The Golden lion tamarin Leontopithecus rosalia: a conservation success storyTitle. Int Zoo Yb.

[4] Coimbra-Filho AF, Mittermeier RA. Distribution and ecology of the genus Leontopithecus lesson, 1840 in Brazil. Primates. 1973;14(1):47–66.

[5] Lapenta MJ, Procópio-de-Oliveira P, Kierulff MCM, Motta-Junior J (2008). Frugivory and seed dispersal of golden lion tamarin (Leontopithecus rosalia (Linnaeus, 1766)) in a forest fragment in the Atlantic Forest, Brazil (Frugivoria e dispersão de sementes por Micos-Leões-Dourados) (Leontopithecus rosalia) em um fragmento florest. Braz J Biol.

[6] Goldsmith C, Rodríguez-Aguilera JR, El-Rifai I, Jarretier-Yuste A, Hervieu V, Raineteau O, Saintigny P, Chagoya de Sánchez V, Dante R, Ichim G, Hernandez-Vargas H. Low biological fluctuation of mitochondrial CpG and non-CpG methylation at the single-molecule level. Sci Rep. 2021;11:8032. 10.1038/s41598-021-87457-8 PMC8044111 33850190

[7] Perez-Sweeney BM, Valladares-Padua C, Martins CS, Morales JC, Melnick DJ. Examination of the taxonomy and diversification of Leontopithecus using the mitochondrial control region. Int J Primatol. 2008;29(1).

[8] Matauschek C, Roos C, Heymann EW. Mitochondrial phylogeny of Tamarins (Saguinus, Hoffmannsegg 1807) with taxonomic and biogeographic implications for the s. nigricollis species group. Am J Phys Anthropol. 2011;144(4). 10.1002/ajpa.21445 21404233

[9] Finstermeier K, Zinner D, Brameier M, Meyer M, Kreuz E, Hofreiter M, et al. A Mitogenomic Phylogeny of Living Primates. PLoS One. 2013;8(7). 10.1371/journal.pone.0069504 PMC3713065 23874967

[10] Lu H, Giordano F, Ning Z. Oxford Nanopore MinION sequencing and genome assembly. Vol. 1e4, Genomics, Proteomics and Bioinformatics. 2016. 10.1016/j.gpb.2016.05.004 PMC5093776 27646134

[11] Gilpatrick T, Lee I, Graham JE, Raimondeau E, Bowen R, Heron A, et al. Targeted nanopore sequencing with Cas9-guided adapter ligation. Nat Biotechnol. 2020;38(4). 10.1038/s41587-020-0407-5 PMC7145730 32042167

[12] Payne A, Holmes N, Clarke T, Munro R, Debebe BJ, Loose M. Readfish enables targeted nanopore sequencing of gigabase-sized genomes. Nat Biotechnol. 2020; 10.1038/s41587-020-00746-x PMC7610616 33257864

[13] Sharma N, Pasala MS, Prakash A. Mitochondrial DNA: Epigenetics and environment. Vol. 60, Environmental and Molecular Mutagenesis. 2019. 10.1002/em.22319 PMC6941438 31335990

[14] Chandler J, Camberis M, Bouchery T, Blaxter M, Le Gros G, Eccles DA. Annotated mitochondrial genome with Nanopore R9 signal for Nippostrongylus brasiliensis. F1000Research. 2017;6. 10.12688/f1000research.10545.1 PMC5399971 28491281

[15] Wick RR, Judd LM, Holt KE. Performance of neural network basecalling tools for Oxford Nanopore sequencing. Genome Biol. 2019;20(1). 10.1186/s13059-019-1727-y PMC6591954 31234903

[16] Xiao C Le, Zhu S, He M, Chen D, Zhang Q, Chen Y, et al. N 6 -Methyladenine DNA Modification in the Human Genome. Mol Cell. 2018;71(2). 10.1016/j.molcel.2018.06.015 30017583

[17] Wang Z, Zolnik CP, Qiu Y, Usyk M, Wang T, Strickler HD, et al. Comparison of fecal collection methods for microbiome and metabolomics studies. Front Cell Infect Microbiol. 2018;8(AUG). 10.3389/fcimb.2018.00301 PMC6127643 30234027

[18] Cuscó A, Salas A, Torre C, Francino O. Shallow metagenomics with Nanopore sequencing in canine fecal microbiota improved bacterial taxonomy and identified an uncultured CrAssphage. bioRxiv. 2019.

[19] Moss EL, Maghini DG, Bhatt AS. Complete, closed bacterial genomes from microbiomes using nanopore sequencing. Nat Biotechnol. 2020;38(6). 10.1038/s41587-020-0422-6 PMC7283042 32042169

[20] Shanmuganandam S, Hu Y, Strive T, Schwessinger B, Hall RN. Uncovering the microbiome of invasive sympatric European brown hares and European rabbits in Australia. bioRxiv. 2019. 10.7717/peerj.9564 PMC7441920 32874776

[21] Mundy NI, Kelly J. Phylogeny of lion tamarins (Leontopithecus spp) based on interphotoreceptor retinol binding protein intron sequences. Am J Primatol. 2001;54(1). 10.1002/ajp.1010 11329166

[22] Rosenberger AL, Coimbra-Filho AF. Morphology, Taxonomic Status and Affinities of the Lion Tamarins, *Leontopithecus* (Callitrichinae, Cebidae). Folia Primatol. 2008;42(3–4).

[23] de Freitas PD, Mendez FL, Chávez-Congrains K, Galetti PM, Coutinho LL, Pissinatti A, et al. Next-generation sequencing of the complete mitochondrial genome of the endangered species Black Lion Tamarin *Leontopithecus chrysopygus* (primates) and mitogenomic phylogeny focusing on the callitrichidae family. G3 Genes, Genomes, Genet. 2018;8(6). 10.1534/g3.118.200153 PMC5982826 29650540

[24] Baeza JA. Yes, we can use it: a formal test on the accuracy of low-pass nanopore long-read sequencing for mitophylogenomics and barcoding research using the Caribbean spiny lobster *Panulirus argus*. BMC Genomics. 2020;21(1). 10.1186/s12864-020-07292-5 PMC7726883 33297960

[25] Simpson JT, Workman RE, Zuzarte PC, David M, Dursi LJ, Timp W (2017). Detecting DNA cytosine methylation using nanopore sequencing. Nat Methods.

[26] Sharma AK, Pafčo B, Vlčková K, Červená B, Kreisinger J, Davison S, et al. Mapping gastrointestinal gene expression patterns in wild primates and humans via fecal RNA-seq. BMC Genomics. 2019;20(1). 10.1186/s12864-019-5813-z PMC6567582 31200636

[27] Malukiewicz J, Cartwright RA, Dergam JA, Igayara CS, Nicola PA, Pereira LMC, et al. Genomic Skimming and Nanopore Sequencing Uncover Cryptic Hybridization in One of World’s Most Threatened Primates. bioRxiv [Internet]. 2021 Jan 1;2021.04.16.440058. Available from: http://biorxiv.org/content/early/2021/04/17/2021.04.16.440058.abstract 10.1038/s41598-021-96404-6 PMC8390465 34446741

[28] Robin ED, Wong R. Mitochondrial DNA molecules and virtual number of mitochondria per cell in mammalian cells. J Cell Physiol. 1988;136(3). 10.1002/jcp.1041360316 3170646

[29] Aminuddin A, Ng PY, Leong CO, Chua EW. Mitochondrial DNA alterations may influence the cisplatin responsiveness of oral squamous cell carcinoma. Sci Rep. 2020;10(1). 10.1038/s41598-020-64664-3 PMC7217862 32398775

[30] Bicci I, Calabrese C, Golder ZJ, Gomez-Duran A, Chinnery PF. Oxford Nanopore sequencing-based protocol to detect CpG methylation in human mitochondrial DNA. bioRxiv. 2021; 10.1093/nar/gkab1179 PMC8682748 34850165

[31] Lüth T, Klein C, Schaake S, Tse R, Pereira S, Lass J, et al. Analysis of mitochondrial genome methylation using Nanopore single-molecule sequencing. bioRxiv [Internet]. 2021 Jan 1;2021.02.05.429923. Available from: http://biorxiv.org/content/early/2021/02/06/2021.02.05.429923.abstract

[32] Mechta M, Ingerslev LR, Fabre O, Picard M, Barrès R. Evidence suggesting absence of mitochondrial DNA methylation. Front Genet. 2017;8(NOV). 10.3389/fgene.2017.00166 PMC5671948 29163634

[33] Shock LS, Thakkar P V., Peterson EJ, Moran RG, Taylor SM. DNA methyltransferase 1, cytosine methylation, and cytosine hydroxymethylation in mammalian mitochondria. Proc Natl Acad Sci U S A. 2011;108(9). 10.1073/pnas.1012311108 PMC3048134 21321201

[34] Dou X, Boyd-Kirkup JD, McDermott J, Zhang X, Li F, Rong B, et al. The strand-biased mitochondrial DNA methylome and its regulation by DNMT3A. Genome Res. 2019;29(10). 10.1101/gr.234021.117 PMC6771398 31537639

[35] McLain AT, Faulk C. The evolution of CpG density and lifespan in conserved primate and mammalian promoters. Aging (Albany NY). 2018; 10.18632/aging.101413 PMC5940106 29661983

[36] Laubach ZM, Faulk CD, Dolinoy DC, Montrose L, Jones TR, Ray D, et al. Early life social and ecological determinants of global DNA methylation in wild spotted hyenas. Mol Ecol. 2019; 10.1111/mec.15174 31291495

[37] De Paoli-Iseppi R, Deagle BE, McMahon CR, Hindell MA, Dickinson JL, Jarman SN. Measuring animal age with DNA methylation: From humans to wild animals. Vol. 8, Frontiers in Genetics. 2017. 10.3389/fgene.2017.00106 PMC5572392 28878806

[38] Formenti G, Rhie A, Balacco J, Haase B, Mountcastle J, Fedrigo O, et al. Complete vertebrate mitogenomes reveal widespread gene duplications and repeats. bioRxiv. 2020. 10.1186/s13059-021-02336-9 PMC8082918 33910595

[39] Colwell M, Drown M, Showel K, Drown C, Palowski A, Faulk C (2018). Evolutionary conservation of DNA methylation in CpG sites within ultraconserved noncoding elements. Epigenetics [Internet].

[40] Oberacker P, Stepper P, Bond DM, Höhn S, Focken J, Meyer V, et al. Bio-On-Magnetic-Beads (BOMB): Open platform for high-throughput nucleic acid extraction and manipulation. PLoS Biol. 2019;17(1). 10.1371/journal.pbio.3000107 PMC6343928 30629605

[41] Li H. Minimap2: Pairwise alignment for nucleotide sequences. Bioinformatics. 2018;34(18). 10.1093/bioinformatics/bty191 PMC6137996 29750242

[42] Li H, Handsaker B, Wysoker A, Fennell T, Ruan J, Homer N, et al. The Sequence Alignment/Map format and SAMtools. Bioinformatics. 2009;25(16). 10.1093/bioinformatics/btp352 PMC2723002 19505943

[43] Quinlan AR, Hall IM. BEDTools: A flexible suite of utilities for comparing genomic features. Bioinformatics. 2010;26(6). 10.1093/bioinformatics/btq033 PMC2832824 20110278

[44] Kolmogorov M, Bickhart DM, Behsaz B, Gurevich A, Rayko M, Shin SB, et al. metaFlye: scalable long-read metagenome assembly using repeat graphs. Nat Methods. 2020;17(11). 10.1038/s41592-020-00971-x PMC10699202 33020656

