# Supplementary material for: The mitochondrial genome and Epigenome of the Golden lion Tamarin from fecal DNA using Nanopore adaptive sequencing
Source: BMC Genomics. 2021 Oct 7;22:726. doi: 10.1186/s12864-021-08046-7 (PMC8499546; doi:10.1186/s12864-021-08046-7)
Supplement: Supplementary file 1 — Additional file 1: Fig. S1. Alignment of reads to L. rosalia from the first 24 h run to the reference mitogenome (KC757399) has 96.83% contig identity over 92% of its length, with 5′ gaps (top). Alignment to L. chrysopygus reference (NC_037878) improves to 99% with complete coverage over the full length (bottom). The 5516 reads are combined from 2810 (1st run) and 2706 (2nd run). [file 12864_2021_8046_MOESM1_ESM.pdf]

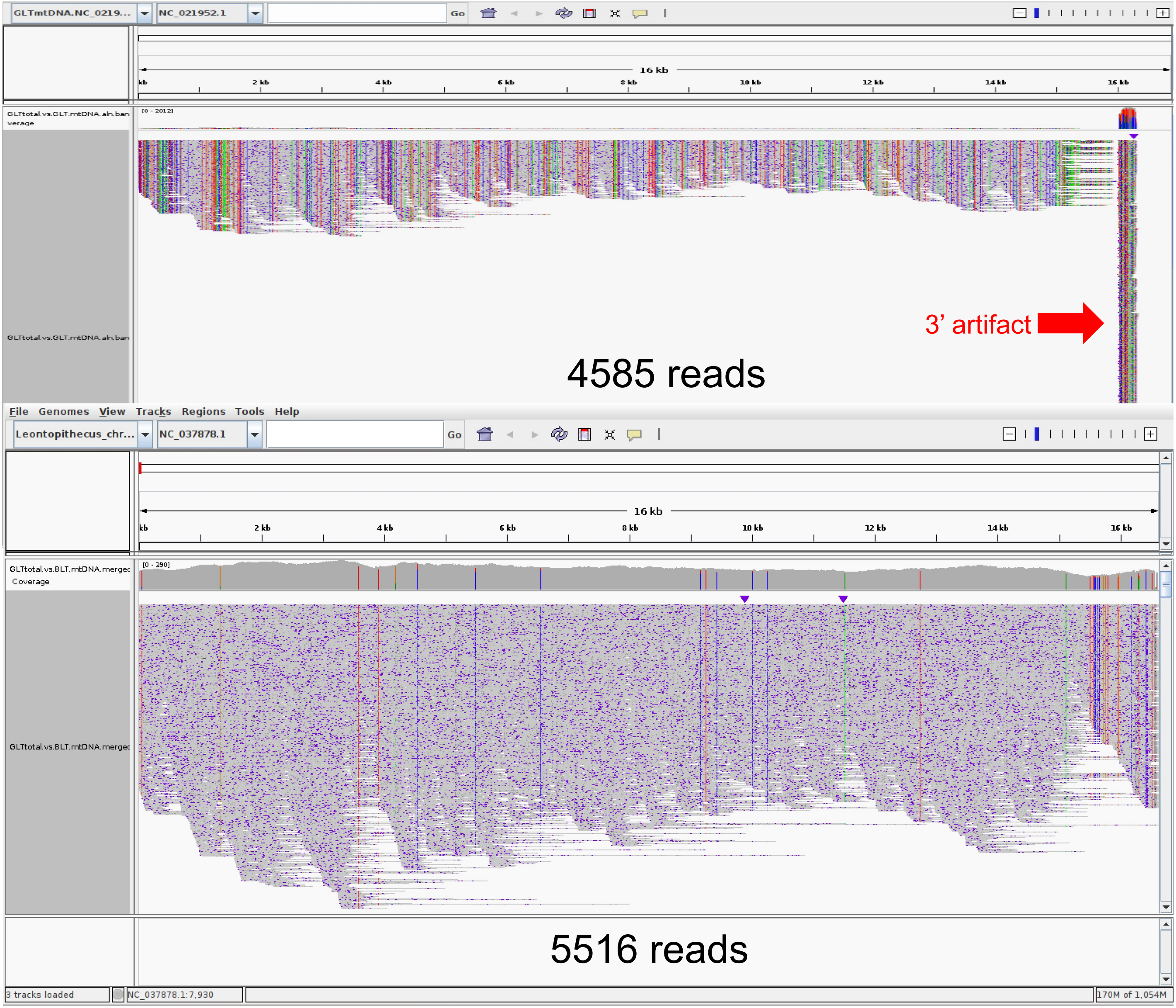

Fig S1. Alignment of reads to *L. rosalia* from the first 24 hr run to the reference mitogenome (KC757399) has 96.83% contig identity over 92% of its length, with 5' gaps (top). Alignment to *L. chrysopygus* reference (NC_037878) improves to 99% with complete coverage over the full length (bottom). The 5516 reads are combined from 2810 (1st run) and 2706 (2nd run).
