# Supplementary material for: The mitochondrial genome and Epigenome of the Golden lion Tamarin from fecal DNA using Nanopore adaptive sequencing
Source: BMC Genomics. 2021 Oct 7;22:726. doi: 10.1186/s12864-021-08046-7 (PMC8499546; doi:10.1186/s12864-021-08046-7)
Supplement: Supplementary file 2 — Additional file 2: Supplementary File 1. Supplementary Methods. [file 12864_2021_8046_MOESM2_ESM.docx]

**Supplementary File 1: Supplementary Methods**

**Sample prep and sequencing choices**

Since this method is intended for future field use, we adapted and simplified the Oxford Nanopore protocol (SQK-LSK109) for practicality and cost considerations. DNA concentration was measured concentration via nanospectroscopy (Implen N60) in lieu of a Qbit. End-repair was performed as instructed, however, we used standard 1.5 ml tubes rather than LoBind tubes, and no Hula mixer. Our tests indicated that DNA lost from bead-washing was remaining in the bead elute, i.e. it was never bound to beads in the first place. We increased bead binding efficiency by longer incubation times and pipetting to mix at least 30 times. We dried the pellet by removing ethanol and immediately proceeded to the next step instead of waiting 30 seconds. To reduce cost of Axygen beads, magnetic beads were diluted to 25% of original volume with in-house prepared carboxy bead dilution buffer (<https://bomb.bio/protocols/>) with no loss in recovery efficiency. We used the Long Fragment Buffer to enrich for DNA fragments >3kb, however our reads averaged 1230 bp upon sequencing, due to digestive degradation. We did not calculate the fmol *a priori*, since we did not have a size estimate, but based on sequencing mean length, we estimate that we loaded between 300 and 600 fmol per run.

**Computational environment and software**

Sequencing computer was built with an AMD Ryzen 3900x processor with 12 threads, 24 cores, 64 Gb RAM and a 1 Tb SSD, and a GeForce 2080Ti with 4352 CUDA cores running Ubuntu Linux 18.04 with Cinnamon desktop. The GPU was tested for defects using gpu-burn (https://github.com/wilicc/gpu-burn). The computer was remote monitored with NoMachine (https://www.nomachine.com). We monitored the CUDA cores with nvtop (https://github.com/Syllo/nvtop).

We built a minimap2 index (.mmi) to use with adaptive sampling using:

minimap2 -d TargetGenome.mmi TargetGenome.fasta

We found that the sequencing run with adaptive sampling used about 40% of the GPU during sequencing. During post hoc high accuracy base calling it used 100% and took approximately 3 hours to process 300 Gb of fast5 files resulting in 6 Gbase of sequence.

*Read generation*

Fastq files were generated from Oxford Nanopore Minion instrument and called with Guppy v4.5.4. We used the GPU enabled version of guppy in concert to enable live basecalling for adaptive sampling and for post-hoc basecalling with the high accuracy model. We used the following post-hoc parameters.

guppy_basecaller --config dna_r9.4.1_450bps_hac.cfg --device "cuda:0" -q 0 --qscore_filtering --nested_output_folder --recursive --input_path /path/to/fast5/ --save_path /path/to/output/location

All resulting fastq files were concatenated and used for further processing.

**Mitochondrial genome assembly**

*Alignment*

The combined fastq file containing all reads was mapped to the Black Lion Tamarin *Leontopithecus chrysopygus* mtDNA genome due to better quality and completeness than when attempting to align to the Golden Lion Tamarin, *L. rosalia*, reference mtDNA genome (NC_021952). We used the following parameters:

minimap2 -ax map-ont Leontopithecus_chrysopygus.NC_037878.mtDNA.fasta CombinedReads.fastq -a -o CombinedReads.vs.BLT.mtDNA.sam -t 24

*File conversion and indexing*

Sort and filter for mapped reads only and convert to bam:

samtools sort CombinedReads.vs.BLT.mtDNA.sam -o CombinedReads.vs.BLT.mtDNA.bam

samtools view -F 0x4 -b CombinedReads.vs.BLT.mtDNA.bam > CombinedReads.vs.BLT.mtDNA.aln.bam

samtools index CombinedReads.vs.BLT.mtDNA.aln.bam

*Create bed file and convert bam to fasta*

bamToBed -i CombinedReads.vs.BLT.mtDNA.aln.bam > CombinedReads.vs.BLT.mtDNA.aligned.bed

samtools fasta f CombinedReads.vs.BLT.mtDNA.aln.bam > CombinedReads.vs.BLT.mtDNA.aln.fasta

*Determine Aligned Read Stats*

seqkit stats CombinedReads.vs.BLT.mtDNA.aln.fasta

*Assemble and Polish*

Use Flye and Medaka with default settings

flye --nano-raw CombinedReads.vs.BLT.mtDNA.aln.fasta --out-dir flyeout --threads 4

medaka_consensus -i CombinedReads.vs.BLT.mtDNA.aln.fasta -d flyeout/assembly.fasta -o . -t 2

*Correct Start Position*

Submit consensus fasta file to MITOS2 (<http://mitos2.bioinf.uni-leipzig.de/index.py>) and determine start position of CoxI gene. Manually rearrange CoxI TSS to position 1 in the fasta file. Resubmit to MITOS2 for new gene positions. Manually adjust annotation table since MITOS2 misses the first TSS of CoxII, and aggregate the D-loop annotations.

*Create Gene Map Figure*

Submit the consensus fasta (single fasta format) to Galaxy at Texas A&M (<https://cpt.tamu.edu/galaxy-pub>) along with ‘NCBI five column tabular’ file from the output of MITOS2, e.g. Leontopithecus.tbl. Use the Galaxy tool “Genome Polishing and Submission -> Five column tabular to Genbanck to create a .gb file. View the .gb file with Open Vector Editor (https://github.com/TeselaGen/openVectorEditor) .

***Visualization***

Integrative Genome Viewer was used to view the .bed and .bam files. <https://software.broadinstitute.org/software/igv/>

***DNA methylation analysis***

*Call methylation and hydroxymethylation at all cytosine contexts (CpG + CH)*

Download basecalling neural network models for guppy from the ONT Rerio.

<https://github.com/nanoporetech/rerio>

Use megalodon with the 5mC + 5hmC all context model v001.

megalodon /path/to/fast5/ --outputs basecalls mappings mod_mappings mods per_read_mods --reference /path/to/assembly.fasta --devices 0 --processes 24 --guppy-server-path /usr/local/ont-guppy/bin/guppy_basecall_server --output-directory <output-5mC_5hmC> --guppy-params "-d /usr/local/ont-guppy/data/rerio/basecall_models/" --guppy-config res_dna_r941_min_modbases_5mC_5hmC_v001.cfg --mod-binary-threshold 0.8

*Post process megalodon output*

Filter output by motif and modification.

Ex. “--motif CG 0” filters for all modifications in the CpG context

megalodon_extras modified_bases create_motif_bed --motif CG 0 --out-filename CG-motif.bed /path/to/assembly.fasta

Repeat with “--motif CH 0”

*Intersect motif bed file with target modification bed file to obtain modification + levels.*

Ex. “CG-motif.bed” contains all modifications in CG context. Intersect with modified_bases.5hmC.bed results in output file with all existing sites in that context for the contig, along with depth of coverage and methylation percentage (in the last 2 columns).

bedtools intersect -a modified_bases.5mC.bed -b CG-motif.bed > CG-motif.methvalues.bed

Repeat with “modified_bases.5hmC.bed”

Circular figure with mapped reads was created with Circos.

**Rationale for alternatives not chosen**

Filtering for quality

Guppy automatically filters adapter sequences and can trim barcodes. Guppy filters for quality scores >7. Therefore, no additional filtering was performed.

Alternatives for assembly, polishing and circularization

We originally used a pipeline of Canu v2.1.1 + Nanopolish to assemble and polish the mitogenome. Due to unresolvable indels in coding regions, we replaced this pipeline with Flye + Medaka. The Canu assembly was linear with overlapping repeats on the end and was circularized with Circlator <https://sanger-pathogens.github.io/circlator/>. Circlator was abandoned in favor of Flye which was able to natively assembly and produce circularized contigs.

Alternatives for DNA methylation

*Calling methylation with Megalodon default model*

The default megalodon neural network model, dna_r9.4.1_450bps_modbases_5mc_hac.cfg, called anomalously high methylation at CpG sites that were inconsistent with Nanopolish and previous mtDNA findings. When we switched to using the most up to date Rerio research model that called both methylation and hydroxymethylation, we found all 5mC calls dropped to nearly zero. The default model is unable to distinguish 5hmC from 5mC, and is likely to miscall 5hmC as 5mC, much in the same way as bisulfite-based methods.

*Call methylation with Nanopolish*

Nanopolish calls methylation uses raw signal data with a hidden Markov model. The Nanopolish call-methylation function was applied on the same reads used for consensus building. Site-specific methylation percentages were determined using the calculate_methylation_frequency.py helper script.

Our Nanopolish pipeline is below:

*Create index with sorted and merged fasta files from both runs.*

Note that the -s parameter is optional but speeded up index creation from days to minutes.

nanopolish index -d /First24Run/fast5_pass/ -s First24Run/sequencing_summary.txt -d /Second24Run/fast5_pass/ -s /Second24Run/sequencing_summary_HAC.txt CombinedReads.sorted.aln.fasta

*Map reads to the consensus.*

minimap2 -a -x map-ont /path/to/medaka-output/consensus_mtDNA.fasta CombinedReads.vs.BLT.fastq > CombinedReads.vs.consensus.sam

Convert .sam to .bam, sort, then merge.

*Call methylation*

nanopolish call-methylation -t 23 -r CombinedReads.vs.consensus.merged.aln.fasta -b CombinedReads.vs.consensus.merged.aln.bam -g medaka-output/consensus_mtDNA.fasta > methylation_calls.vs.consensus_mtDNA.tsv

*Calculate frequencies using helper script*

~/Desktop/nanopolish/scripts/calculate_methylation_frequency.py methylation_calls.vs.consensus_mtDNA.tsv > methylation_frequency.vs.consensus_mtDNA.tsv

*Convert methylation calls to .bed format (for reading in IGV)*

PycoMeth creates nice format and methylation percentages (https://adrienleger.com/pycoMeth/)

pycoMeth CpG_Aggregate -i methylation_calls.vs.consensus_mtDNA.tsv -f consensus_mtDNA.fasta -b methylation_calls.vs.consensus_mtDNA.CpG_Aggregate.bed -t methylation_calls.vs.consensus_mtDNA.CpG_Aggregate.tsv -s consensus_mtDNA --progress

*Make methplotlib figure*

methplotlib -m methylation_calls.vs.consensus_mtDNA.tsv -n "Leontopithecus" -w "Leontopithecus” --dotsize 6
