# Supplementary material for: The mitochondrial genome and Epigenome of the Golden lion Tamarin from fecal DNA using Nanopore adaptive sequencing
Source: BMC Genomics. 2021 Oct 7;22:726. doi: 10.1186/s12864-021-08046-7 (PMC8499546; doi:10.1186/s12864-021-08046-7)
Supplement: Supplementary file 4 — Additional file 4: Supplementary File 3. Consensus sequence of L. rosalia mitogenome in fasta format. [file 12864_2021_8046_MOESM4_ESM.docx]

Supplementary File 3. Consensus sequence of *L. rosalia* mitogenome in fasta format.

>Leontopithecus_rosalia_mtDNA: Consensus sequence of L. rosalia mitogenome.

ATGTTCATAAACCGCTGACTATTTTCAACTAATCACAAAGACATTGGGACATTATATTTATTATTTGGCGCATGGGCGGGGGCAGTAGGTACAGCCCTAA

GCCTCCTAATTCGAACAGAACTAGGACAACCCGGAAGCCTAATAGAAGACGACCATGTATACAATGTTATTGTCACCGCCCACGCATTCATCATAATTTT

TTTCATAGTAATACCAATTATGATTGGGGGTTTTGGGAACTGACTTATTCCTTTAATAATTGGCGCTCCCGATATAGCATTTCCTCGAATAAATAACATA

AGCTTCTGACTTCTACCACCCTCCCTACTCCTACTGCTTGCATCATCAACTTTAGAAGCCGGTGCCGGCACTGGTTGGACAGTCTACCCACCCCTAGCAG

GCAATTTATCCCACCCAGGAGCCTCTGTAGATTTAACCATTTTTTCACTACATTTAGCAGGCATTTCTTCTATTCTTGGAGCTATTAACTTTATTACAAC

AATTGTAAATATGAAACCGCCAGCCATGACTCAATATCAAACTCCGTTGTTTGTGTGATCCGTCCTAATTACTGCAGTCCTTCTTTTACTTTCTCTTCCA

GTTCTAGCTGCAGGGATTACCATACTATTAACTGACCGTAATTTAAATACTACTTTCTTTGATCCTGCTGGTGGCGGCGACCCTATCCTATACCAACACT

TATTCTGATTCTTCGGTCACCCTGAAGTATATATTCTAATCTTACCGGGTTTTGGAATAATTTCACACATTGTAACATATTATTCTAATAAAAAAGAACC

CTTTGGTTATATGGGCATGGTATGAGCCATAATATCTATTGGTTTCCTAGGCTTTATTGTATGAGCCCATCACATATTCACAGTAGGAATAGATGTAGAT

ACTCGTGCATATTTTACATCAGCTACCATAATCATTGCTATTCCCACTGGAGTAAAAGTATTTAGCTGATTAGCCACACTGCACGGCGGTAATATCAAAT

GATCTCCCGCAATATTATGAGCCCTGGGCTTTATTTTTCTTTTTACCGTGGGTGGACTAACAGGAATTGTGTTAGCTAACTCATCATTAGATATTGTATT

ACATGATACATACTATGTAGTAGCCCACTTTCACTATGTGTTATCAATAGGAGCAGTATTTGCCATTATAGGGGGATTTATTCACTGATTCCCGCTCTTT

TCAGGCTATACTCTTGACCAAACATATGCCAAAATCCACTTCACCATTATATTTGTCGGCGTAAATTTAACTTTCTTCCCACAACACTTCCTTGGCTTAT

CCGGAATACCTCGACGATACTCAGACTATCCAGACGCATATACTACATGAAATATCGTCTCATCTATCGGTTCATTTATTTCACTTACAGCAGTAGTCCT

GATGGTTTTTATAATTTGAGAAGCTTTCTCTTCAAAACGAAAAGTCTTAGCCATTGAGCAACTATCCACCAATCTAGAATGATTATACGGCTGCCCTCCT

CCTTACCACACATTCGAAGAGGCAACTTACGTTAAATCCTAGACGAAAAAGGAAGGATTTGAACCCCCAAAAATTGGTTTCAAGCCAATCCCATATACCC

TATGACTTTTTCAATAAGATATTAGTAAAATAATTACATAACTTTGTCAAAGTTAAATTATAGACTAAATATCTATATATCTTAATAGCAACACCAGCTC

AACTAGGTTTACAAAATGCCACATCACCTATCATAGAAGAACTTATTGCTTTCCACGACCATGCACTCATAATTATTTTCCTGATTAGTTCCTTAGTCTT

ATATATTATTTCCCTTATACTTACCACAAAACTCACTCATACTAGCACCATAAATGCTCAAGAGATCGAAATAATCTGAACTATTCTCCCTGCACTGATT

CTAATTACAATTGCCCTCCCATCACTGCGTATTCTATATATGACAGACGAATTTAATAAACCTTATTTAACCCTTAAAGCAATTGGTCACCAATGATACT

GAACCTATGAATATTCCGACTATGAAGACTTAGTATTTGACTCTTACATTATGCCAACATATTTCCTTGAGCCAGGGGAATTTCGACTCCTCGAAGTTGA

TAACCGAACAACTTTACCTATAGAAGCGGATATTCGCATATTAATCTCATCACAAGACGTCTTACACTCATGAGCCGTACCATCACTAGGCGTAAAAACA

GATGCAATTCCTGGACGTTTAAACCAAGCCATACTAGCCTCTATACGACCAGGCCTATTTTATGGACAATGCTCGGAAATTTGCGGGTCCAATCACAGCT

TTATACCTATTGTTCTAGAATTCATTTATTTCCAAGATTTCGAAGTATGAGCTTCATACTTATATATTGTATCACTGTAAAGCTAACTTAGCATTAACCT

TTTAAGTTAAAGATTGAGAGAACAAACTCTCTATAGTGAATGCCTCAACTAAATATTTCACCGTGACCAATAGTAATTATATCTATAATTGTTACCTTAT

TTTATATTATACAATTGAAAATACTGAACTTTACTTTCCATTATTACCCACTACCAAAATTAGTAGAAACACAAAAACATAAAACAACTTGAGAACTAAA

ATGAACCAAAATCTATTTGCCTCATTCAATATTCCAACAATACTAGGAGTACCCTTAGTATTTTTAATTATTGCACTCCCCACTACATTAATTTTATCCT

CCAAAAACTTATTCAACAACCGACTCTCTTCAATTCAACAATGGCTAATTCAACTAACACTTAAACAAATAATATTAACCCACACCACTAAAGGGCGAAC

CTGATCCCTTATACTCCTAGCCCTAATTTCTTTTATTGCCCTAAATAACATTCTCGGACTTACACCATATGCATTTACACCAACCACCCAACTGTCAATA

AATCTAGGCATAGCTATTCCTCTATGAGCAGCAACTGTACTAATAGGCCTCCGATTTAAAACAAAATCATCCCTCGCTCATTTTTTACCACAAGGAACAC

CAATTCCACTAATCCCTATATTAATTATTATTGAAACAATTAGTCTATTCATTCAACCTGTAGCCTTAGCCGTACGATTAACAGCCAATATTACAGCAGG

TCACCTATTAATGCATTTACTTGGGGATACAACATTAACTCTTCTATCAATTTACCTCTCCACTTCCACAATCACTATCATTATTATTATTTTATTAATT

ACCCTAGAGTTGGGTGTAGCCTTAATTCAAGCCTATGTATTTACCCTCTTAGTAAGCCTGTACTTACATGATAATTCATAATGACTCACCAAACCCATGC

TTATCACATAGTCAACCCAAGCCCTTGACCACTAACAGGAGCATTATCAGCTTTTCTTCTCACATCCGGCCTAGTTATATGATTCCACTTTTACCACACA

CTGCTTCTCACTGCAGGTCTACTAGCTAGTTCTATAACAATATTTCAATGATGACGTGATGTAGTACGAGAAAGTACATATCAAGGCCACCATACTGCAC

CTGTCCAAAAAGGCCTACGATACGGAATAATTCTATTTATTATTTCAGAAATTTTCTTCTTTGCAGGTTTCTTCTGAGCATTTTATCATTCTAGTCTAGC

CCCAACTCCACAAACAGGGGGACTATGACCACCTACAGGCATTACTCCCCTCAACCCAATAGAAGTCCCTCTCTTAAATACAACCGTACTACTAGCATCA

GGAGTTACAATTACATGAGCACATCACAGCCTCATAGAAGCTAACCGAAAAGAATCAACCCAAGCACTACTCCTAACCATTATATTAGGGATCTACTTCA

CCTGCCTACAATTATCAGAATATTCTGAAGCCCCATTTACTATCTCCGACGGAGTATATGGATCCACATTTTTTATGGCTACAGGCTTTCATGGCCTTCA

CGTAATTATCGGAACCACTTTCCTCACCACCTGTTACTTTCGCCAACAATTATATCACTTCACATCTAGCCACCATTTCGGCTTCGAAGCCGCTGCATGA

TATTGACATTTCGTAGATGTAGTATGACTTTTCCTCTATATTTCCATCTATTGATGAGGCTCTTACTCTCTTAGTATAAAAAGTATTATTGACTTCCAAT

CAACGGGCCTCGAATGATTCGAGAGAGAGTATTATAAATTTAATTTTAGCCCTAATGACTAACATTACTTTGGCCTTACTTCTTATCACAATTACATTTT

GACTTCCACAATTAAATATTTATACAGAAAAGCACAACCCTTACGAGTGCGGATTTGATCCTACAACCTCCGCCCACTTACCATTCTCCATAAAATTTTT

CCTAATCGCCATCACATTTCTCCTATTTGATCTGGAAATTGCCCTACTTCTACCCCTACCATGGGCAACCCAAACAAATAATTTAACTCTAACAATAAAT

ATAATTTTTACCCTACTTATTATTCTGGCTTTAGGGTTAGCCTACGAGTGGTCCCAAAAAGGATTAGATTGGGCTGAATTGGTATATAGTTTAATTAAAA

CAAATGATTTCGACTCATTAGATTATGAAAGCTCATATTTACCAAATATGCCTTTTATCTATATTAATGTAATACTAGCATATTTCATATCATTATTAGG

GTTATTAATTTATCGATCTCACCTAATATCATCACTGCTATGTTTGGAAGGCATAATATTATCATTATTTATCATAATTACACTCACAACTTTCAATATG

CACTTCATATTAATGTATATGATACCCCTCATTCTCCTAGTATTTGCCGCATGCGAAGCTGCAGTAGGCCTAGCCTTGTTAATTTTAGTCTCCAACCTAT

ATGGCCTAGATTATGTACAAAACCTAAACTTACTCCAATGTTAAAATTTATTTTTCCAACCATCATAATACTTCCCACCATATGACTTTCAAAAAATTAT

ATAATATGAATCAACACAATAATCTGTAGTCTACTAATCAGTATATACGCCCTCATATTACTCCACACACCAAACAACTCATGCAACCTATCACTGATTT

TTTCCTCAGATTCACTAACATCACCACTTCTTATATTAACAGCCTGACTTCTGCCACTAATAATTCTAGCAACACAACAGCATTTATATAATAACCCCAC

CCCACGAAAAAAACTATATATCTCAATATTAATTCTATTACAAATTTCACTTATTATAACTTTTTCAGCTACCGAACTAATTTTATTTTATATTCTATTT

GAAACTACCCTAATCCCCACCCTAATTATTATTACCCGTTGAGGGTATCAACCAGAACGCCTTAATGCTGGTTCATATTTTCTATTCTACACACTAGCAG

GATCCTTACCTCTATTAATTACCCTCCTATACTATCTAAATACTTTGGGATCCCTAAGCATACTTACAATAATTATTAATTCTAACGAAATACTTACTTC

ATGAACCAATAGTATTATATGATTGGGATGTATAATAGCTTTTATAGTCAAAATACCCCTATATGGGTTACACCTATGACTCCCAAAAGCTCACGTAGAA

GCCCCCATCGCTGGCTCAATAGTACTTGCAGCAATCTTACTAAAACTAGGAGGGTATGGTATAGTGCGAATTACTCCTATCCTCAATCCACTAACAGAAA

AAATAAGCTACCCTTTTATCATTCTATCCCTATGAGGGATAGTGATAACAAGCTCCATCTGCTTACGACAAGCCGACCTAAAATCACTTATCGCTTACTC

CTCCGTCAGCCACATAGCACTTGTTATTTTAGCTATTATAATTCAAACCCCATGAAGCCTTACCGGTGCAATAATACTAATAATCTCCCATGGACTTACC

TCATCCCTGCTATTTTGTTTAGCAAATACTAACTACGAACGAATTCACAGCCGAACTATAATATTTACACGAGGCCTTCAAACATTATTTCCCCTTCTAG

CACTTTGATGACTCCTAGCTAACCTAGCAAATTTAGCTCTCCCCCCAACTATTAATCTAATAGGTGAACTATTAACAATCTTAGCTTCTTTCTCTTGATC

TAATTTTACCATCATATTTACAGGTTTCAACATGTTAATTACAGCCCTCTACTCACTTCACATATTTACCTCAACACAACGAGGGCCATTAACGTACAGC

ACTAGCAGCATCAAACCCCTCTTTACACGAGAAAATGTACTAATAATAATACATTTAATACCCATCCTACTATTAACTACAAACCCCAAGATAATTATAG

GTTTGACGCCCTGTAGTTATAGTTTAATAAAAACATTAGATTGTGAATTTAATGATAGAAGCCCATAACTTCTTAACTACCGAGAAAGTATGCAAGAACT

GCTAATTCATGCTACCAGGCCTAACAACTTGGCTTCCTCAACTTTTAAAGGATAGTAGTTATCCATTGGTCTTAGGAACCAAAAATATTGGTGCAACTCC

AAATAAAAGTAAAAATATACTCCTCAATAATTATATTCACTGTTATTCCACTACTAGTACCAATCTTAATAACTATAGTTAATTTACATAAAAGCCTCCT

ATATCCTTATTACGTAAAACTAGCTATTATTTATGCCCTCACTGCCAGCATCCTATGTATAGCAATATACATCTTTACAGGCCAAGAATTGATGATTTCA

AACTGACACTGAACTACTATTCAAACTATCAAGTTATCACTCAGCTTTAAAATAGATTTTTTCTCCACAATATTTGCCCCCGTAGCACTCTTTGTCACCT

GATCAATTGTAGAATTCTCAACATGGTATATAAGCTCAGACCCAAACATTAACCAATTCCTTAAATATCTTCTCATTTTCTTAATTACAATATTAATTCT

AATTACTGCAAACAATCTATTTCAACTTTTTATCGGATGGGAGGGGATAGGTATTATATCATTTCTATTAATTAGCTGATGGTACGGACGAACAGATGCC

AACACAGCAGCCCTGCAGGCAATTTTATATAACCGGATCGGAGATATCGGTTTTATTTTAGCAATAACGTGGTTCTTTTTATATCATAACTCATGAGACT

TTCAACAAATATTTATACTAGATTATACTCCTAACTCTTTTCCCCTAACAAGCTTACTTTTAGCAGCAACAGGAAAATCTGCTCAATTTGGTCTACATCC

GTGATTACCCTCCGCTATAGAAGGGCCCACCCCAGTGTCAGCACTACTGCACTCTAGCACAATAGTTGTTGCAGGAATCTTTTTAATTATCCGTTTTTAT

CCTTTAATAGAAAATAATCAATTTATCCAAACAATAGCGCTATCACTTGGCGCAATCACCACCCTATTTACAGCAATTTGTGCCCTAACACAAAATGACT

TAAAAAAGATTGTAGCCTTTTCTACCTCAAGCCAACTAGGCCTTATAATAGTGACAATTGGTATTAACCAACCACACCTAGCCTTCCTCCATATCTGCAC

TCACGCCTTCTTCAAAGCCATATTATTTCTATCCGCAGGGTCCATTATTCACAGCCTAAACAACGAGCAGGACATCCGCAAAATAGGTGGACTATTTAAA

ATACTCCCATTCACATCCTCCTCACTTGTCATTGGCAGTTTCGCACTTATAGGTATACCCTTCCTCACAGGCTTTTACTCAAAAGACCTAATCATCGAAA

TCGCCAACACGTCGTATACCAACGCCTGAGCACTCACAACCACCCTAGTAGCTACCTCTATTACAGCTATATATAGTATTCGTATTATTTACTTTACCAT

AACAGGGCACCCACGTTTCATAACTCTAACACCAATCAACGAAAATAACCCTATACTAATAAATCCCATTAATCGCCTAGCAATTGGTAGCATTTTCGCC

GGGTTTCTTATTTCTAATTGTGTTGCTATTACCTCACACCCCCAAGTCACCATACCTTACTACCTCAAACTAACAGCTTTGAGCGTAACCGTCCTAGGAC

TTATAATAGCAACAGAACTTAGTCTAATAACTAACAATATAAAATTAAGCACCCCATTAAAAACTTTCTACTTTTCTAACATACTAGGTTTTTACACTAT

TACTACACACCGACTCAACCCCCATTCAAGCCTAAACATAAGCCAAAACATTACTTCAACTCTACTAGACCTATTCTGACTAGAAAAATCTATACCAAAA

ATAACAATACAAACTCAAATCTCAATATCTACAACTTCCACAACTCAAAAAGGCCTGATTAAATTGTATTTCTTATCCTTCTTCATTCCACCAACTCTAG

CCCTACTTTTAACTATTTAACCACCACCTCGAGTGAGCTCAATAGCAATATGCATACCCATAAACAGAGCCCAACAAGTAACTACAACAACTCAAACACC

ATAATTATATAAAGCCGCAGCACCTGTAGGATCCTCACGAATCAACCCAGGTCCTTCTCCCTCATAAATTATTCAACTTGCCACAGTCTTATAATTGACT

GTAATTTCCACCGTTTTAACAGGATCCCCACCCAATAATAATACTATCATTATTTCCATCATTAAACCCAATACAAAAATTCCTAAAATATCAACACTTG

AAATTCAAGTTTCAGGATGTTCATCAATTGCTATAGCCGCAGTATAACCAAATACAACCATTATACCTCCTAGATAAATTAAAAAAACCATAAGCCCCAT

ATAAGACCCACCAAAATATAACGTAATCGCACAACCCACAGCACCACTAAAAATTAACACCAACCCGCCGTAAATAGGAGAAGGTTTAGAAGAAAACCCC

ACAAAACCTATTACCAAAATAATACTTAATGAAAATAAAGCATATGTCATTATTCCTACATGGACTATAACCATGACTAATGATATGAAAAACCATTGTT

GTATTTCAACTATAAGAATAATAATGACCTCCCCTCGCAAAACCCACCCACTAGCAAAAATCATCAACGAATCATTTATTGACCTCCCCACACCATCCAA

TATCTCATCCTGATGAAATTTCGGTTCACTTTTAGGCACCTGTCTAATTATTCAAATCACCACAGGCCTATTTCTAGCAATACACTACACACCAGACACT

TCCACCGCTTTCTCCTCAGTAGCCCACATTACCCGAGATGTTAATTACGGATGAATAATTCGTTATTTACACGCCAACGGTGCATCCATATTTTTCATCT

GCCTTTTCCTCCACATTGGACGAGGCCTATATTACGGATCTTTTCTTTCTCTGAAGACTTGAAATGTGGGTACAATCCTACTATTAGCAACTATGGCCAC

GGCATTTATAGGTTATGTACTTCCATGGGGCCAAATATCATTCTGAGGGGCTACAGTAATTACAAACCTCTTATCAGCCATCCCCTACATCGGCTCCGAC

TTAGTGCAATGAATCTGAGGCGGATTCTCAGTAGATAAAGCCACCCTAACACGATTTTTTACCTTTCACTTTATTTTACCTTTCATTATCGCAGCCTTAG

CAACAATTCACCTTCTCTTTCTGCATGAAACAGGTTCAAGTAACCCATCAGGAATAGCCTCAGAACCCGACAAAATCACATTTCACCCATATTATACAAC

CAAAGACATTCTTGGACTAATTTTTCTTCTCTTATTCCTAATAAGCCTAACACTATTTTTACCTGACCTCTTAACAGACCCAGACAACTACACACTAGCC

AACCCCCTAAACACCCCTCCCCATATTAAACCAGAATGATACTTCCTATTTGCATATGCCATCCTACGATCTATCCCCAATAAACTGGGAGGAGTTCTAG

CACTTATACTTTCTATCCTAATTCTAATAATTATTCCCATAACACATTTATCCAAACAACAAAGCATAAAATTCCGACCTATTTCCCAAATCCTATTCTG

AACCCTAGTAGCTGATTTACTTACACTAACATGAATTGGAGGGCAACCAGTAGAATATCCATTTATCACCATTGGCCAAACAGCCTCCATTATATATTTT

CTAATTATCATCACCTTAATCCCCCTCTCTTCCTTAATCGAAAATAAAATACTTAAATGATAAAGTCCTTGTAGTATAAATTAATACTCTGGTCTTGTAA

ACCAGAAATGGAGACAGATCTACTCCCCGGGGCAATTCAGGGGAAGAACAACTAATTCTACCATCAACACCCAAAGCTGATATTCTAGTATTAAACTACC

CCCTGAGCAACAAAGAATTTATTCTATTGGCCCAACCATGAGGTACTTTATAAGTATTAACATTCTCTCATGACCTCATGTAATTCGTGCATTAATGCAC

GCCCACATGAATAATGTACAGTACCAAAAATGCTTAACTATACATAGTACATTAAACACCAAACGTACATAAAATCCTCATCAAGCATGCTTACAAGCAA

GAACTATTATTGCACTCCGGACTATAGAACATGCGTTTACACAACCCATAAAATGCTCAACCCTACGAATATCATTTAGAACAAGAACTCATTTATAGTA

CATGAGTACATTATTTACATTGATCGGACATAGCACATTCTGTCAGATCATTGTCCGGTCTATGGATATCCATCTTATACTTTTGGTCTCTTAATCTACC

AACCTCCGTGAAACCAGCAACCCGCCCACTTCTACTAGTATTCTCGCTCCGGGCCCATATAGACAGGGCTTGGTTATACTGAAACTATATCTGGCATTTG

GTTCCTACCTCAGGGCCATGAACCTAGGTTCCCACTACTGGTTCCCCTTAAATAAGGCATCACGATGCACCACGGCGCTGCCACCCTCTTTATCTCGTCA

CTGGATGCATACAGCGCCCCTGGTAGGAAAGGAATGTACTCATCAGCATCGCCGAACGGGCTCCTGGAAAGAGGTTCCAATTAACATCCTGTAGTACCTG

ACTGTGTTTTGCCAGACTTTATGCTATCATCGCGCCTATTATTGAATGTCTTTGCCCCCATCCCGCCCCCAAGTGGATATCAAGTTAATGGTTACAGGAC

ATGATAAAAGGTAAAAACACCTTTTTTTTTATTTTATTTTTTTTTATTTTTTTTTTATTTTTTTTTATTTTTTTTTTTATTTTTTTTATTTTTTTTTTTA

ATTTTATTAACTTTACAATACGTTAACTCAAATCCTCAAACCCAACCTACCTATATTTTCTGAAGGATGAACCATAAAGAGACATGTAACTATTAACATT

TCAATCTCCGACCACAATATAAACTTATATTTATATATATATATATACAATTATAGATTACATTATCAATACTACTCACTTCTACTCCCCACACACCTCA

AATTTTACCCTTCAGAGAATATACTTATATTACGCTATATATATTTTTTTGCTAATTTATTAATTTAAATTTAACAAGTTAATGTAGCTTAAGTTTAAAG

CAAGACACTGAAAATGTCTAGACGGGTAATTATTACTCCATAAACACATAGGTTTGGTCCTAGCCTTTCTATTAGCCCTCAGTAAGATTACACATGCAAG

CATCCACGACCCTGTGAAAATGCCCTCCACCAAAACATGAGGAGCGAGTATCAAGCACGCAAACATGCAGCTCAAGACACTTTGCTTAGCCACACCCCCA

CGGGAGACAGCAGTGACAAACTTTTAGCAATAAACGAAAGTTTAACTAAGCTATACTGACTATTAGAGTTGGTCAATTTCGTGCCAGCCACCGCGGCCAT

ACGATTGACTCAAGTTAATAGAGCACGGCGTAAAGGGTGTTTAAGATTTTATTAACTCAAATAAAGCTAACCTATAACTAAGTCGTAGAAAACCCCAGTT

ACAGTAAAATAAACTACGAAAGTGGCTTTAATATTCTGAATACACTACAGCTAAGGTACAAACTGGGATTAGATACCCCACTATGCTTAGCCTTAAACCT

TAATAATTCAATTAACAAAATTATTCGCCAGAACACTACAAGCAACAGCTTGAAACTCAAAGGACCTGGCGGTGCTTTACATCCGTCTAGAGGAGCCTGT

TCTATAATCGATACACCCCGATACACCTCACCACCTCTTGCCCTCAGCCTGTATACCGCCATCTTCAGCAAACTCCTTAATGATCGTAAAGTAAGCAGAA

GTGTCATCATAAAAACGTTAGGTCAAGGTGCAGCCAATGAGGTGGGAAGAAATGGGCTACATTTTCTACCCCAGAAAATTACACGATACCCCTTATGAAA

CCTAAGGGCCCAAGGTGGATTTAGCAGTAAACCAAGAATAGAGAGCTTGATTGAAACAAGGCCATTAAGCACGCACACACCGCCCGTCACCCTCCTCAAA

CACCACACAAAAGTACATTAACAATAAAATACTAAAAACTGGTATAGAGGGGATAAGTCGTAACATGGTAAGCGTACTGGAAAGTGCGCTTGGACAAACC

AAAATGTAGCTTAAAATAAAGCATCCGGCTTACACCCGGAAGATGTCATAACAAATGATCATTTTGAGCTAATCCTAGCCCAACTCTCCATTAAATATAT

TATTCATTTATATTAATTAAATCATTTACCTATAGTAAGAGTATAGGCGATAGAAATTACATACTAGGCGCAATAGATATAGTACCGTAAGGGAAAGACA

TTACCTAATAAGCATATAAAAGCAAAGACGAGTCCTTATACCTTCTGCATAATGAATTAACTAGAAGTAGTTTTATAAAGAGAACTTCAACAAAGTACCC

CGAAACCAAGCGAGCTACCCAAGGACAGCTATAAGAGCACACCCGTCTATGTGGCAAAATAGTGGGAAGATCTATGGGTAGTGGCGACAAACCTAACGAG

CTTGGTGATAGCTGGTTATCCAAGACAGAATCTTAGTTCGACTTTAAATTTATCCCCAGAATTATTAAATCCTCATGTAAATTTAACTGTTAGTCTAAAG

AGGGACAGCTCTTTAGACCCTAGGAAAAAACCTTTAATAGAGAGTAAGTAGTATAAATCCCATAGTTGGCCTAAAAGCAGCCATCAATTAAGAAAGCGTT

CAAGCTCAATATTCTCATATATTTAATTCTACTAATTTTATCGAACTCCTGAAACAAATTGGATTAATCTATTATCTAATAGAAGCAATAATGTTAGTAT

AAGTAACATGAATCTATTCTCCCCGCATAAGCTTATTTCAGACCGAAACAACTACTGTTAGTTAACAGCCTAATAATCATAAACTACAAATTAAAATATC

AATTAACTAAACTGTTAACCCAACACAGGCATGCACTAAGGAAAGATTAAAAAAAGTAAAAGGAACTCGGCAAGCTCTAACCCCGCCTGTTTACCAAAAA

CATCACCTCTAGCATTTCTAGTATTAGAGGCACTGCCTGCCCAGTGACATATGTTCAACGGCCGCGGTACCCTGACCGTGCAAAGGTAGCATAATCACTT

GTTCTCTAAATAGGGACTTGTATGAATGGCCACACGAGGGTTTAACTGTCTCTTACTTTTAATCAGTGAAATTGACCTATCCGTGAAGAGGCGGATATAC

ATAAATAAGACGAGAAGACCCTGTGGAGCTTTAATTTAATGATACAAACTAGATTTGTAAAAACCAACAGGCATTAATTTACCGTCAATGTATTATAAAT

TTCGGTTGGGGCGACCTCGGAGTAAAATAGAACCTCCGAAAAACATATACCAAGACCTTACCAGTCTAAGTAAGCAAGCACCTATTGACCCAATAATAAT

TTGATCAACGGACCAAGTTACCCTAGGGATAACAGCGCAATCCTATTTTAGAGTCCATATCGATAATAGGGTTTACGACCTCGATGTTGGATCAAGACAT

CCTAATGGTGCAGAAGCTATTAAGGGTTCGTTTGTTCAACGATTAAAGTCTTACGTGATCTGAGTTCAGACCGGAGCAATCCAGGTCGGTTTCTATCTAT

TTAAATATTTCTCCCAGTACGAAAGGACAAGAGAAATGGGGCCCACTTCATAAAGCGCCCTCAACAATCAGATGACCTCCATCTCAACCTCACATATTAT

AATCTTGCCCAAGAACAGGGCTCGTTAAGGTGGCAGAGCCCGGTAATTGCATAAAACTTAAAACTTTATAATCAGAGGTTCAATTCCTCTTCTTAACAAC

ATGTATATAATTAATTTACTAATACTAGTCCTACCTGCCCTAATTGCCATAGCCTTTCTAACACTCACAGAACGAAAAATCCTGGGCTATATACAATTCC

GAAAAGGCCCTAATATTGTAGGCCCCTACGGAATACTTCAACCAATCGCTGACGCCATAAAACTCTTCACAAAAGAACCCTTATTACCTACCACATCCAC

CATAACTTTATATTTAACTGCCCCCACCCTAGCTCTTTCCATTGCTCTTCTACTATGAACGCCACTCCCCATACCATATCCTCTTATCAACTTCAATCTT

GGTCTCCTATTTATCCTCGCAACATCAAGCCTAGCTGTTTACTCAATTTTATGATCTGGCTGAGCATCCAACTCAAACTACGCACTAATTGGCGCACTAC

GAGCTGTAGCCCAAACAATCTCATATGAGGTCACCCTTGCCATTATCTTACTATCAACACTACTAATAAGCGGCTCATTCAATCTACAATCGCTTATTAC

CACTCAAGAACACTACTGACTTCTACTTCCATCATGGCCCCTAGCCATAATATGATTTATTTCCACATTAGCAGAAACTAATCGAGCTCCATTCGACCTG

ACAGAAGGCGAATCAGAACTAGTATCAGGTTTCAACATTGAATACGCTGCAGGCTCATTCGCCTTATTCTTCATAGCAGAGTATATAAATATTATTATAA

TAAATGCCCTAACTACCACTATTTTCTTATCCACACCCTACAATATAATTATACCAGAAACATTTACTATTAATTTTATAGCCAAAACCCTCCTACTAAC

CACTTTATTTTTATGAATTCGAACAGCCTACCCCCGCTTCCGCTACGATCAATTAATATTCTTACTATGAAAAAACTTTTTACCACTTACATTAGCACTA

TGTATATGATATGTTTCAATACCCATCCTAGCATCTGGCATCCCACCCCAAACATAAGAAATATGTCTGATAAAAGAGTTACTTTGATAGAGTAAATTAT

AGAGGTTCAAACCCTCTTATTTCTAGGATTACAGGAATTGAACCTACACCTGAGAACTCAAAACTCTCCGTGCTACCGATTACACCATATCCTAAACAGT

AAGGTCAGCTAAATAAGCTATCGGGCCCATACCCCGAAAATGTTGGTTTAATCCTTCCCGTGCTAACATTAATCCTCTAGCCCACCTTATTATCTCCTTC

ACCATTCTAACAGGGACCGTAATCACAATTTTAAGCTCACATTGATTCCTAGCCTGAATAGGCTTAGAATTAAATATACTAGCCATCGTACCAATCCTTG

CCAAAAGTACCAATCCCCGCTCCACAGAGGCATCCACCAAATATTTTTTAATTCAAGCAACAGCATCAATACTTCTATTAGTATCCATTTTCCTTAACAA

TCTACTAACTCAACAATGAACAATCAATCCTCCTTATAACCAAATATTATCCACAATAATATTTATTGCTCTAGCAATAAAAATAGGGATAGCCCCACTT

CACTTCTGACTCCCAGAAATTACCCAAGGAATCCCTCTAATCCCAGCTATAATTATTCTCACGTGACAAAAACTCGCCCCAATATCAATTCTCCTCCAAA

TTTTTCCGTCAACAAACCTAAACTTGATTCTAACAATCTCAGTTCTATCAATTATAATTGGCAGCTGAGGAGGACTCAACCAAACACAACTCCGCAAAAT

CCTAGCCTATTCTTCAATTACTCACATAGGATGAATAATAGCAGTATTATATTACGACCCTAATATTACTATATTAACTTTAATTATTTATATTTTCCTA

ACAATCTCTACATTAATAATCTTTTATTTAACCTCAAATGTAACAACCCTATCCCTATCACATACCTGAAACAAACTAGCATGAACAATACCCATTATTC

CACTAATAATAATATCCCTAGGAGGTCTACCCCCACTAACAGGTTTTTCCCCCAAATGAGCTATTATACAAGAACTTATTAAAAATGATAACTTAATTAT

TCCCCTTATAATAGCTTTACTAACATTAATAAATTTATATTTTTATATACGTTTAATATATTATATCTCAATGACAATATTCCCAACATCAAATAACACA

AAAATCAACTGGCAACTAAATTATATAAAGCCAATACCGTTTCTATCCCCACTTGTAGTGTCTTCTACCTGTCTCCTACCCCTAACTCCACTAATACTTA

TAACTTAGAAATTTAGGTTAATAAGACCAAGAGCCTTCAAAGCCCTTAGTAAGTAAATTTTACTTAATTTCTGCACAACAAATAAGGACTGCAAAACTTT

ATTCTGCATCAACTGAACGCAAATCAATTACTTTAATTAAGCTAAGCCCTTCCTAGATTGATGGGATTTTAACCCACAAAAATTTAGTTAACAGCTAAAT

AACCTAATCAACTGGCTTCAATCTACTTCTCCCGCCGTTAGGGAAAAAAGGCGGGAGAAGCCCCGGCAGAATTGAAGCTGCTTCTTTGAATTTGCAATTC

AATGTGATAGTTCACCTCAGGGCTGGTAAAAAGAGGGTTCACTCCTCTGTCTTTAGATTTACAGTCTAATGCTTGCTCAGCCATTTTACCCCTACCT
