# Supplementary material for: The mitochondrial genome and Epigenome of the Golden lion Tamarin from fecal DNA using Nanopore adaptive sequencing
Source: BMC Genomics. 2021 Oct 7;22:726. doi: 10.1186/s12864-021-08046-7 (PMC8499546; doi:10.1186/s12864-021-08046-7)
Supplement: Supplementary file 5 — Additional file 5: Supplementary File 4. Alignment reads used for mitogenome assembly. [file 12864_2021_8046_MOESM5_ESM.docx]

Supplementary File 4. Alignment reads used for mitogenome assembly.

>f327e926-ad2b-4c52-b873-fb27a5a0d441

ATTGTCACTTCGTTCAGTTACGTATTGCTGGGCTGGTAAAAAGAGGTTTCACTCCTCTGTCTTTTTAGATTCCACAGTCTAATGCTTGGCTCAGCCATTTTACCCTACCTATGTTCATAAACCGCTGACTATTTTCAACTAATCACAAAGACATTGGGACATTATATTTATTATTTGGCGCATGGGCGGGGGCAGTAGGTACAGCCTAAGCCTCCTAATTCGAACAGAACTAGGACAACCCGGAAGCCTAATAGAAGACGACCATGTATACAATGTTATTGTCACCGCCCACGCATTCATCATAATTTTTCATAGTAATACCAATTATGATTGGGGGTTTTGAGACGACTTATAGCAATACGTAATAACATCATTGTACTTCGTTCAGTTACGTATTGCTCAGTGGCTTTAAAACTTCAAGTGTTCTTTCTTTTACGTTGTAGTCAGCAAATTTGCAACATTCCAATAGCTTTACTGATTTGTTCTGCCTTTTGCTCATGAGGTCTTGACATGTAGTTTAAAAGAGCTGTTTGCCTAAGATTTAACTCTTTTACAATATGAATTAAAGCTCTCAATCGACCTAATCTGTCGTAGATTGATGGCTTCTCTTTAAAGCCGTATTTAAATGCACATTTTAAAGAGAATCGGTATTAAGACATTTGCCTTCAGTAAAGAGTTCTGAGAGTTTTAATACTTTGTTTACATCTGTAAGAGCATAAACATTATCTAAAAGATAGTAGGTTCCTGAATTGTCTAAGACACATGCTCTCTAAGATTTTTAAATTACGAATAGACCAAGTAAAGATAGTACATTGGGATTATGTAAGATCTTTTCTGACTCATCTAAAAACTTTCGACGCTAAGCCCATTTTAATTGTGTAAATCGATACCCTGAGTGAAAAGATAACTTGGACTTGGTAGTCTGTAGTCATAGCCTTTACAGGTAATAAGCTCATTTGTCGATAATATTAAAGTTCAAATCTGTTAATACATAGGATAATGAATACAGACTTGGGTATTTATCATCAGGTGAGGTTACTGGCTCTGGAAGTAAAATAGTAACTTTCTATCGTAGTTTATCATTGTAAAACTGACTACATCTATTCAATTGTAAAATCAAAAGTCTTTAAGCTCTTACCTAAACTGATTCTGTTCCAAATTGTATTCAAAGAAACCTGCTACTTTTAATTTTCCAGTAAGCTCATTCTTATCAATATCATTAGTAGCACACAGTTCATCTAATGATGAGTATTCATCACGTAACCATATTTACCGAAAAAACTGATAACAGAATGAATGGATCCATGTTTTTAAAATTAGCTAAAGACATCATAATAAAATCCTCGCAAAGATCTAATTTAGTTTAGATGGTATCGTAATGTATTTGCCCACAATAAAATTCCACCTTCAGCGCTGTAGTGATTGTCATCTACAAACAACGGCACTCCATTATCGTCATAGGTCTTGTAATAGCAATACGTAGCA

>768f7268-31ad-466c-9470-de7fa831f13a

AGTACCTTTACGTTCAGTTACGTATTGCTAATTTGCAATTCAATGTGATAGTTCACCTCAGGGCTGGTAAAAAGAGGTTCACTCCTCTGTCTTTAGATTACAGTCTAATGCTTGCTCCTTCATTTACCCCTACCTATGTTCATAAACCGCTGACTATTTTCAATAATCCACAAAGACATTGGGACATTATATTTATTATTTGGCGCATGGGCGGGGGCAGTAAGGTACAGCCCTAAGCCTCCCTAATTCGAACAGAACTAGAGACAGCGGAAGCCCAATAGAAGACGACCATGTATGCAATGTTATTGTCACCGCCCGCATTCATCATAATTTTTTTCATAGTAATACCAGCAATACGTAAC

>797c9b15-3176-474b-8d66-67bb36f34b30

AATTGTACTTCGTTCCCAGTTACGTATTGCTAGGGCTGGTAAAGAGGGTTCACTCCTCTGTCTTTAGATTTACAGTCTAATGCTTGCTCAGCCATTTTACCCCTACCTATGTTCATAAACCGCTGACTATTTTCAACTAATCACAAGAAGACATTGGGACATTATATTTATTATTTGGCGCATAGGCAGGGGCAGTAGGTACAGCCTAAGCCTCCTAATTCGAACAAGAATAGGACAACCCGGAAGCCTAATAGAAGACGACCATGTATACAATGTTATTGTCACCGCCCACGCATTCATCATAATTTTTTTCATAGTAATACCAATTATGATTGGGGTTTTGGGAACGACTTATTCCTTTAATAATTGGCGCTCCCGATATAGCATTTCCTCGAATAAATAACATAAGCTTCTGACTTCTACCACCCTCCTACTCCTACCTTGCATCATCAACTTTAGAAGCCGGTGCCGGCACTGGTTGGACAGTCTACCCACCCCTAGCAGGCAATTTATCACCCAGGAGCCTCTGTAGATTTAACCATTTTTTCACTACATTTAGCAGGCATTTCTTCTATTCGCGGAGCTATTAACTTTATTACAACAATTGTAAATATGAAACCGCCAGCCATGACTCAATATCAAACGCTCCGTTGTTTGTGTGATCCGTCCTAATTACTGCAGTCCTTCTTTTACTTTCTCTTCCAGTTCTAGCTGCAGGGATTACCATACTATTAACTGACCGTAATTTAAATACTACTTTCTTTGATCCTGCTGAGTGGCAGCGACCTATCTATACCAACACTTATTCTGATTCTTCGGTCACCCTGAGTATGCTATTCTAATCTTACCGGAGTTTGGAATAATTTCACACATTGTAACATATTATTCTAATAAAAAAGAACCCTTTGGTTATATGGGCATGGTATGAGCCATAATATCTATTGGTTTCCTAGGCTTTATTGGCATGAGCCCATCACATATTCACAGTAGGAATAGAAGCAATACGTAACA

>e8c88527-3f0d-410f-9829-96140171399b

ATTGTACTTCGTTCAGTTACGTATTGCTCTAGATTGATGGGATTTTAACCCACAAAAATTTAGTTAACAGCTAAATAACCTAATCAACTGGCTTCAATCTACTTCTCCCGCCGTTGGAAAAAGAAAATGGGAAGCCCCGGCAAGGAGTGAAGCTTAACAACTCTAATTTGCAATTCAATGTGATAGTTCACCTCAGGGGCTACGAGTAAGAGGGTTCACTCCTCTGTCTTTAAAGATTTACAATCTAATACTTGCTCAATAGCCATTTACCCTACCTATGTTCATAAACCGCTGACTATTTTCAACTAATCACAAAGACATTGGGACATTATATTTATTATTTGGCGCATGGGCAGGGGCAGTAGGTACAGCCTAAGCCTCCTAATTCGAACAGAACTAGGATGACAGAGCCTAATAGAAGACGACCATGTATACAATGTTATTGTCACCGCCAGCAATACGTAAC

>8eb9783f-aa92-4a2b-a745-d071bed09510

TTGTACTTCGTTCAGTTGCTCCCCCCAAATGAGCTATTATACAAGAACTTATTAAAAATGATAACTTAATTATTCCCCTTATAATAGCTTTATTAACATTAATAAATTTATATTTTTATATACGTTTAATATATTATATCTCAATGACAATATTCCCAACATCAAATAACACAAATCAACTGGCAACTAAATTATATAAAGCCAATACCGTTTCTATCCCCACTTGTAGTGTCTTCTACCTATCGTCCTACCCCTAACTCCACTAATGTATAACTTAAAAATTTAGGTTAATAAGACCAAGAAGCCTTCAAAGCCCTTAGTAAGTAAATTTTACTTAATTTCTGCACAACAAATAAGGACTGCAAAACTTATTCTGCATCAACTGAACGCAAATCAGTACTTTAATTAAGCTAAGCCCTTCCTAGATTGATGGGATTTTAACCCACAAAAATTTAGTTAACAGCTAAATAACCTAATCAACTGGCTTCAATCTACTTCTCCCCGCCGTTAGGGAGAAAAAAGGCGGAGAAGCTGACAGAATTAGGCTTCTTCTTCGAATTTACGTTCAATGTGATAGTTCACCTCAGGGCTGGTAAAAGAGGGTTCACTCCTCTGTCTTTAGATTTACAGTCTAATGCTTGCTCAGCCATTTTACCCCTACCTACCATGTTCATAAACCACTTGACTATTTTTTCAACTAATCACAAAGACATTGGGACATTATATTTATTATTTGGCGCATGGGCGGGGGCAATGGTACAGCCCTAAGCATAATTCGAACAGAACTAGGACAACCCGGAAGCCTAACTTAGAAGGCGACCATGTATACAATGTTATTTGTACCGCCCACGCATTCATCATAATTTTTTTCATAGTAATACCAATTATGATTGGGGGGTTTTTGGAACTGACTTATTCCTTTAATAATTGGCGCTCCCGATATAGCATTTCCTCGAATAAATAACATAAGCTTCTGACTTCTACCACCTCCCTACTCCTACTGCTTGCATCATCAACTTTAAAGCCGGTGCCGGCACTGGTTGGACAGTCTACCCACCCCTAGCAGGCAATTATCCCACCCAGGAGCACTCTGTAGATTTAACCATTTTTTCACTACATTTGCTTGAGCATTTCTTCTATTCTTGGAGCTATTAACTGTACAACAATTGTAAATATGAAACCGCCAGCCATGACTCAATATCAAACTCCGTTGTTTGTGTGATCCGTCTAATTACTGCAGTCCTTCTTTTACTTTCTCTTCCAGTTCTAGCTGCAGGGATTACCATACTATTAACTGACCGTAATTTAAATACTACTTTCTTTGATCCTGCTGAGTGGCGGCGACCTATCCTATACCAACACTTATTCTGATTCAGCGGTCACCCTGAAGTATATATTCTAATCTTACCGGGTTTTGGAATAATTTCACACATTGTAACATATTATTCTAATAAAAAAGAACCCCTTTAGCAATACGTAACA

>79dca44f-d3df-4a42-9d0f-be9483e28794

TTGTACTTCGTTCAGTTACGTATTGCTAGTCAGCAGCAACAAGCCGTTTACTACCTATGTTCATAAACCATGACTATTTTCAACTAATCACAAAGACATTGGGACATTATATTTATTATTTGGCGCATGGGCGGGGGCAGTAGGTACAGCCTAAGCCTCCTAATTCGAACAGAACTAGGACAGCCCGGAAGCCTAATAGAAGACGACCATGTATACAATGTTACATGCCGCCCACGCATTCATCATAATTTTTTTCATAGTAATACCAATTATGATTGGGGGTTTTGGGAACTGACTTATTCCCTTTAATAATTGGCGCTCCCGATATAGCATTTCCTCGAATAAATAACATAAGCTTCTGACTTCTACCACCCTCCTACTCCTACTGCTTGCATCGTATCAACTTTAGAAGCCGGTG

>8d90520a-4618-471e-b7a6-ee1e04ac463f

ATTGCATTACTTCGTTCAGTTGCGACATTACAGCCCTTCCTAGATTGATGAGTTTAACCCACAAAATTTAGTTAACAGCTAAATAACCTAATCAACTGGCTTCAATCTACTTCTCCATAGTTAAGGAAAAAAGGCGGGAGAAGCCCCGGCGAAATTAGCTTCCTTCTTTGAATTTGCAATTCAATGTGATGATTCACCTCAGGGCTGGTAAAAGAGGGGTTATCCTCTGTCTTTAGATTTACAGTCTAATGCTTGCTCAGCCATTTTACTACTATGTTCATAAACCGCTGACTATTTTCAACTAATCACCAAAGACATTGGAGCATTATATTTATTATTTGGCGCATAGGCTGGGGCAGTAGGTACAGCCCTAAGCCTCTAATTCGAACAGAACTAGGACGGCAGGCCTAATAGAAGACGACCATGTATACAATGTTATTGTCACCGCCCACGCATTAATCATAATTTTTTTCATAGTAATACCAATTATGATTGGGGGTTTAGGAATTTGAGCTTATTCCTTTAATAATTGGCGCTCCCCGATAATAGCAATACGTAACA

>1b0dacc6-56f9-4456-b457-c7176eb57e3e

ATGTGCTTCGTTCAGTTACGTATTGCTAAAGAAGGGTTCACTCCTCTGTCTTTAGATTTACAGTCTAATGCTTGCTCAGCCATTTTACCCCTACTATGTTCATAAACCGCTGACTATTTTCAACTAATCACAAAGACATTGGGACATTATATTTATTATTTGGCGCATGGGCGGGGGCAGTAGGTACAGCCCACGTAAGAAGCCTCCTAATTCCGAACAGAACTAGGACAACCCGGAAGCCTAATAGAAGACGACCATGTATACAATGTTATTGTCACCGCCCGCATTCATCATAATTTTTTTCATAGTAATACCAATTATGATTGGGGGATTCAGGAACTGACTTATTCCTTTAATAATTGGCGCTCCCGATATACCAGCATTTCCTCCA

>8c54f9ec-66fc-4fe5-adbb-13abd64c848b

GTTGTACTTCGTTCAGTTACGTATTGCTCGCAAATCAATTACTTTAATTAGCTAGCCCTTCCTAGATTGATGGGATTTTAACCCACAAAAATTTAGTTAACAGCTAAATAACCTAATCAACTGGCTTCAATCTACTTCTCCGCCGTTAGGGAAAAAGGCGGGAGAAGCTGTGAAGGTGAAGCTGCTTCTTTGAATTTGCAATTCAATGTGATAGTTCACCTCCAGGGCTGGTAAAAAGAGGGTTCACTCCTCTGTCTTTAGATTTACAGTCTAATACTTGCTCAATACGTTTACCCCTACCTATGTTCATAAACCGCTGACTATTTTCAACTAATCACAAAGACATTGGGACATTATATTTATTATTTGGCGCATGGGCGGGGGCAGTAGGTACAGCCCTAAGCCTCTAATTCGAACAGAACTGGACAACCCGGAAGCCTAATAGAAGACGACCATGTATACAATGTTATTGTCTGCGCCCCACGCATTCATCATAATTTTTCATAGTAATACCAATTATGATTGGGGGTTTTGAACTGACTTATTCCTTTAATAATTAGCGCTCCGATATAGCATTTCGAATAACCGTACGTAA

>f452ba44-d17b-4bc4-b5b9-e085a43384ad

GTTGTACTTCGTTCAGTTGCGTATTGCTCTTGAATTTACAGTCTAATGCTTGCTCAACCATTTACCCCTACCTATGTTCATAAACCGCTGACTATTTTTCAACTAATCACAAAGACATTGGGACATTATATTTATTATTTGGCGCATGGGCAGGGAGGCAGTAAGGTACAGCCCTAAACCTCTAATTCGAACAGAACTAGGACAGCCCGGAAGCCTAATAGAAGACGACCATGTATACAATGTTATTGTCACCGCCTACGCATTCATCACTTAATTTTTTCATAGTAATACCAATTATGATTGGGGTTCAAGGAACGACTTATTCTTCAATAATTGGCAACTCCCGATATAGCATTTCCTCGAATAAATAACATAAGCTTCTGACTTCCTACCACACCACCCTCCCTACTCCTACTGCTTGCATCATCAACTTTAGAAGCCGGTGCCGGCACTGGTTGGATGATCTACCCCACCTAGCAGGTAATTTATCTTCCACCAGGCCTCTGTAGATTTAACCAGCTTTCCACTACATTTAGCAGGCATTTCTTCTAATTCTTGGAGCTATTAACTTTATTACAACTAA

>ab184894-e492-4671-b18b-1097f247d4e1

TTGTACTTCGTTCAGTTACACGTATTGCTCTAAATAACCTAATCAACTGGCTTCAATCTACTTCTCCGCCGTTAGGGAAAAAAAGGCGGGAGAAGCCCCATTAAGTGAAGCTGCTAACGAATTTGCAATTCAATGTGATGGATTACTCAGGGCTGGTAAAAAGAGGTTCACTCCTCTGTCTTTAGATTTACAGTCTAATGCTTGCTCAGCCATTTTACCCCTACCTATGTTCATAAACCGCTGACTATTTTCAACTAATCACAAAGACATTGGGACATTATATTTATTATTTGGCGCATGGGCGGGGCAGTAGGTACAGCCTAAGCCTCCTAATTCGAACAGAACTAGGACAGCCCGGAGACCTAATAGAAGACGACCATGTATACAATGTTATTGTCACCGCCCGCATTCATCATAATTTTTTTCATAGTAATACCAATTATGATTGGGGGTTTTGGGAACTGACTTATTCCTTTAATAATTGGCGCTCCCGATACTAGCATTTTCCTCGAATAAATAACATAAGCTTCTGACTTCTACCACCCTCCCTACTCCTACTGCTGCATCATCAACTTTACATTATAAATTCATGAAACATAACTTTTAAGCCCATCGGATCCGATGGGCTTAGAAGGTATGATGTGTGTGCCTTTCAATGTTAAAGATAGACTCGTGAACTAA

>7fec7be7-8927-4f52-a5e6-db91965b7334

GTATTACTTCGTTCAGTTACGTATTGCTACCTAATCAACTGGCTTCCAATCTACTTCTCCCGCCGTTAGGGAAAAAGGCGGGAGAAGCCCCGGCTTAGGAGTGAAGCTGCTTCTTCTGAATTTGCAATTCAATGTGATAGTTCACCTCAGGGTGGTAAAAAGAGGGTTCACTCCTCTGTCTTTACAGATTACAGTCTAATGCTTGCTCTGGCCATTTACCCTACCTATGTTCATAAACCGCTGACTATTTTCAACTAATCACAAGAACATTGGGACATTATATTTATTATTTGGCGCATGGGCGGGGGCAGTAGGTACAGCCCTATAAGCCTCCTAATTCGAACAGAACTAGGACAACCCGGAAGCCTAATAGAAGACGACCATGTATACAATGTTATTGTCACCGCCCACGCATTCATCATAATTTTTTTCATAGTAATACCAATTATGATTGGAGCAATACGTAAAC

>45aeaa43-98c4-48f2-b15a-0434e36db368

ATGTTGTAGCTACACCGGTTGTATTGCTCTTCAATCTACTTCCCGCCGTTAGGGAAAAAGGCGGGGAGAAGCCCCGGCAGGGTGAAGCTGCTTCTTTGAATTGCAATTCAATGTGATAGTTCACCTCAGGGCTGGTAAGAGAGGTTCTCCTCTGTCTTTAGATTTACAGTCTAATGCTTCTTTCAACCATTTACTACCTATGTTCATAAACCGTGACTATTTTCAACTAATCACAAAGACATTGGGACATTATATTTATTATTTGGCGCATGGGCAGGGGCAGTAGGTACAGCCTAAGCCTCTAATTCGAACAGAACTAGGACAACCCGGAAGCCCTAATGAAGACGACCATGTATACAATGTTATTGTCACCGCCCACGCATTCATCATAATTTTTTTCATAGTAATACCAGTGCGTTGGGGGTTTT

>0515a1af-8ac0-4780-8afb-4f8da5b2dc11

TTATACTTCGTTCAGTTACGTATTGCTGTTAACAGCTAAGCCGCCTAATCCAACTGACGCAATCCTGCTTCCACGCCGTTAGGAAAAAGGCAGGAGAAGCCCCGGCAAGGTGAAGCTGCTTCTTTCGAATTTGCAATTCAATGTGATAGTTCACCTCAGGGCTGGTAAAAGAGAGGGGTTCCACTCCTCTGTCTTTCCGTTTTACAGTCTAATGCTTGCTCAACCATTTACCCTACCTATGTTCATAAACCGCTGACTATTTTCAACCTGATCACAAAGACATTGGGACATTATATTTATTATTTGGCGCATGGGCGGGGGCGGTAGGTACAGCCCTAAGCCTCCTAATTCGAACAGAACTAGGACAACCCGGAAGCCTAATAGAAGACGACCATGTATACAATGTTATTGTCCGCCCGCATTCATCATAATTTTTTCATAGTAATACCAATTATGATTGGGGGTTTGGAACTGACTTATTCCTTTAATAATTGGCGCTCCCGATATAGCATTTCTCGAATAAATAACATAAACTACA

>fc8ab4f2-af35-41de-ba6d-3a56ae25f2d3

TTGTACTTCGTTCAGTTGTGTGCTTATGTTCATAAACCGCTGACTATTTTCAACTAATCACAAAGACATTGGGACATTATATTTATTATTTGGCGCATGGCGGGGGCAGTAGGTACGGCCTAAACAATTCCGAACAGAACTGAGACAACCGGAAGCCTAATAGAAGACGACCATGTATACAATGTTGTCACCGCCCACGCATTCATCATAATTTTTTTCATAATGTGCAATTATGATTGGGGTTTGGGAACTGACTTATTCCTTTAATAATTGGCGCTCCCGATATAGCATTTCCTCGAATAAATAACATAAGCTTCTGGCTTCTGCCACCCTCCCTACTCCTACTGCTTGCATCATCAACTTTAGAAGCCGGTGCCGGCACTGGTTGGACAGTCTACCCACCCCTAGCAGGCAATTTATCACCCAGGGCCTCTGTAGATTTAACCA

>5d9c558e-a279-4f63-9e51-579cbb6b69e3

AGTATGCTTCGTTCAGTTACGTATTAGCTTTTATATTTTTATATACGTTTAATATATTATATCTCAATGACAATATTCCCAACATCCAAACTTACAAAAATCAACTGGCAACTAAATTATATAAAGCAATACCGTTTCTATCACTTGTGAGTGTCTTCTGCCTGTCTCTACCCCTAACTCCACTAATACTTACTGGCGAGAATTTAGGTTAATAAGACCAAGAGCCTTACAAGCCTTAGTAAGTAAATTTTTACTTAATTTCTGCACACAACAAATAAGGACTGCAAAACTTTATTCTGCATCAACTGAACGCAAATCAATTACTTTAATTAAGCTAAGCACTTCTAGATTGATGGGTTTAATAACCCACAAAAATTTAGTTAACAGCTAAATAACCTAATCAACTGGCTTCAATCTACTTCTCCCGCCGTTAGGGAAAAAAAGGCGAGAGAAGCGTAAGCTGAAGCTGCTTCTTTGAATTTGCAATTCAATGTGATAGTTCACCTCAGGGCTGGTAAAAAAGAGGGTTCACTCCTCTGTCTTTAGATTTACAGTCTAATGCTTGCTCAGCCATTTTACCCCTACCTATGTTCAATAAACCGCTGACTATTTTCAACTAATCACAAAGACATTGGGACATTATATTTATTATTTGGCGCATGGGCGGGGGCAGTAGGTACAGCCCTAAGCCTCCTAATTCGAGCAGAACTAGGACGACCCTAATAGAAGACGACCATGTATACAATGTTATTGTCACCGCCCACACATTCATCATAATTTTTTCATAGTAATACCAATTATGATTGGGGGTTTCTGGGAACTGACTTATTCCTTTAATAATTGGCGCTCCCGATATAGCATTTCCTCGAATAAATAACATAAGCTTCTGACTTCTACCACCCTCCTACTCCTACTGCTTGCATCATCAACTTTAGAAGCCGGTGCCGGCACTGGTTGAACGAATCTACCCACCCCTATGCTTGGTAATTATCCCACCCAGGAGCCTCTGTAGATTTAACCATTTTCACTACATTTAGCAGGCATTTCTTCTATTCTTGGAGCTATTAACTTTATTACAACAATTGTAAATATGAAACGCCAACCATGACTCAATATCAAACTCCGTTGTTTGTGATTCGTCTAATGCACAGTCCTTCTTTTACTTTCTCTTCCAGTTCAGCTGCAGGGATTACCATACTATTAACTGACCGTAATTTAAATACTACTTTCTTTGATCCTGCTGGTGGCGGCGACCCTATCCTATACCAACACTTATTCTGATTCTTCGGTCACCCTGGTATATATTCTAATCTTACCGGGTTTTGGAATAATTTCACATTGTAACATATTATTCTAATAAAAAGAACGCTGGTTATATGGGCATGGTATGAGCCATAATATCTATTGGTTTCCTAGGCTTTAAGCAATATTACGTAACTTCG

>857cdf17-973d-408d-9748-ca5e5c917901

TTGTACTTCGTTCAGTTACGTATTGCTACTTCACTTCTGACTCCCAGAAATTACCCAAGGAATCATAATCCCAGCTATAATTATTCTCACGTGACAAAAACTCGCCCCAATATCAATTTATATCCCAAATTTTTCCGTCAACAAACCTAAACTTGATTCTAACAATCTCAGTTCTATCAATTATAATTGGCAGCTGAGGAGGACTCAACCAAACACAACTCCGCAAAATCCTAGCCTATTCTTTCAATGCTCCACATAGGTTGAATAATAGCAGTATATTACGACCCTAATATTACTATATTAACTTTAATTATTTATATTTTCCTAACAATCTCTACATTAATAATCTTTTATTTAACCTCAAATGTAACAACCTATCCTATCACATACCTGAAACAAACTAGCATGAACAATACCCATTATTCCACTAATAATAATATCCCTAGGAGGTCTACCCCCACTAACAGGTTTTCCCCCAAATGAGCTATTATACAGAGAACTTATTAAAAATGATAACTTAATTATTCCCCTTATAATAGCTTTACTAACATTAATAAATTTATATTTTTATATACGTTTAATATATTATATCTCAATGACAATATTCCCAACATCAAATAACACAAAAATCAACTGGCAACTAAATTATATAAAGCCAATACCGTTTCTATCCCCACTTGTAGGTGTCTTCTACCTGTCTCCTACCCCTAACTCCACTAATACTTATAACTTAGAAATTTGGGTTAATAAGACCAAGAGCCTTCAAACCCTTAGTAAGTAAATTTTTACTTAATTTCTGCACAACAAATAAGGACTGCAAAACTTTATTCTGCATCAACTGAACGCAAATCAATTACTTTAATTAAGCTAAGCCCTTCTAGATTGATGGGATTTTAACCCACAAAATTTAGTTAACAGCTAAATAACCTAATCCAACTGGCTTCAATCTACTTCTCCCGCCGTTAGGGAAAAAGCGGGAAAGCCCAGCAAGGTAAGAAGCTACTTCTTTGAATTTGCAATTCAATGTGATAGTTCACCTCAGAACTGGTAAAAAGAGGGTTCCTCCTCTGTCTTTAGATTTACAGTCTAATGCTTGCTCAGCCATTTACCCCTACCTATGTTCATAAACCGCTGACTATTTTCAACTAATCACAAAGACATTGGGACATTATATTTATTATTTGGCGCATGGGCGGGGGCAGTAGGTACAGCCTAAGCCTCCTAATTCGAACAGAACTAGGACAACCCGGAAGCCTAATAGAAGACGACCATGTATACAATGTTATTGTCACCGCCCGCATTCATCATAATTTTTTTCATAGTAATACCAATTATGATTGGGGTTTTGGGAACTGACTTATTCCTTTAATAATTGGCGCTCCCATTATAGCATTTCCTCGAATAAATAACATAAGCTTCTGACTTCTACCCACACCCTCTACTCCCCTACTGCTTGCATCATCAACTTTAGAAGCCGGTACCGGCATGGTTGGACAGTCTACCCACCCAGCAGGCAATTTATCCCACCCAGGAGCCTCTGTAGATTTAACCATTTTTTCACTACATTTAGCAGGCATTTCTTCTATTCTTGGAGCTATTAACTTTATTACAACAATTGTAAATATGAAACCGCCAGCCATGACTCAATATCAAACTCCGTTGTTTGTGTGATCCGTCCTAATTACACAGTCCTTCTTTTACTTTCTCTTCCAGTTCTGGCACAGGGATTACCATACTATTAACTGACCGTAATTTAAATACTACTTTCTTTGATCCTGCTGGTGGCGGCGACCTATCTATACCAACACTTATTCTGATTCTTCGGTCACCCTGAAGTATATATTCTAATCTTACCGGGTTTTGGAATAATTTCACACATTGTAACATATTATTCTAATAAAAAAGAACCCTTTGGTTATATGGGCATGGTATGAGCCATAATATCTATTGGTTCCTAGGCTTTGTGTATGAGCCCATCACATATTCACAGTAGGAATAGATGTAGATGCTCATTATATTTTACATCAGCTACCATAATCATTGCTATTCCCACTGGAGTAAAAGTATTTAGCTGATTAGCCACACTGCACGGCGGTAATATCAATGATCTCCGCAATATTATGAGCCCTGGGCTTTATTTTTCTTTTACCGTGGGTGGACTAACAGGAATTGTGTTAGCTAACTTCATCATTAGATATTGTATTACATGATACATACTATGTAGTAGCCACTTTCACTATATTGTTATCAATAGGAGCAGTATTTGCCATTATAGAGTTATTCACTGATTCCGCTCTTTTCAGGCTATACTCTTGACCAAACATATGCCAAAATCCACTTCACCATTATATTTGTCGGCGTAAATTTAACTTTCTTCCCACAACACTTCCTTGGCTTATCCGGAATACCTCGACGATACTCAGACTATCCAGACGCATATACTACATGAAATATCGTCTCATCTATCGTTCATTTATTTCACTTACAGCAGTAGTCCTGATGGTTTTTATAATTTGAGCAATACGTAACTT

>0695ee61-f5f7-46ec-9bb1-6fbc376372a4

ATTGTGTAGTTTCAGTTACGTATTGCTAATAACCTAATCAACTGGCTAAATCTCTACTTCTCCCGCCGTTAGGGAAAAAAGGCGGGAGAAGCCCCGGCAGAATTGAAGCTGCTTCTTTATTTGCAATTCAATGTGATATTAGTTCACCTCAGGGCTGGTAAAGAGGGTTCACTCCTCTGTCTTTAGATTTACAGTCTAATGCTTGCTCAGCCATTTTACCCTACCTATGTTCATAAACCGCTATTCTTTCAACTAATCACAAGGGAGCATTCCGGGACATTATATTTATTATTTGGCGCATGAAAGGCAGTAGGTACAGCCCTAAGCCTCTAATTCGAACAGAACTAGGACAACCCGGAAGCCTAATAGAAGACGACCATGTATACAATGTTATTGTCACCGCCCACGCATTCATCATAATTTTTCATAGTAATACCAATTATGATTGGGGTTTTGGGAACTGACTTATTCCTTTAATAATTGGCGCTCCCGATATAGCATTTCCTCGAATCAAATAACATAGCTTCTGACTCTACCACCCTCCCTACTCTACTGCTTGCATCATCAACTTTAGAAGCCGGTGCCGGCACTGGTTGGACAGTCTACCCACCCCTAGCAGGCAATTTATCACCAGGAGCCTCTATGAATTTAACCATTTTTTCACTACATTTATGAGCATTTCTTCTATTCTTGGAGCTATTAACTTTATTACAACAATTGTAAATATGAAACCGCCAGCCATGACTCAATATCAAACTCCGTTGTTTGTGCTGATCCGTCTATTACTGCAGTCCTTCTTTTACTTTCTCTTCCAGTTCTAGCTGCGGGGATTACCATACTATTAACTGACCGTAATTTAAATACTACTTTCTTTGACAGTCTACCAGTGGCGACTATCCTATATACCAACACTTATTCTGATTCTTATTCACCCTGAAGTATATATTCTAATCTTACCGGGTTTTGGAATAATTTCACACATTGTAACATATTATTCTAATAAAAAGAGCCTAGCAATACGTAA

>3cc9689e-9588-4461-9dab-910a0cac52b6

GATGTACTTCGTTCGTTACGTATTGCTATATTCCCAACATCAAATAACACCAAAAATCAACTGGCAACTAAATTATATAAAGCCAATACCGTTTCTATCCCCACTTGTAGTGTCTTCTACCTGTCTCACCCTAACTCCACTAATACTTATAACTTAGAAATTTAGGTTAAGCCAATAAGACCAAGAGCCTTCAAGGCCCTTAGTAAGTAAATTTTACTTAATTTCTGCACAACAAATAAGGACTGCCAAACTTTATTCTGCATCAACTGAACACCAAATCAATTACTTTAATTAAGCTAAGCCTTTACCTAGATTGATGGGATTTTTAACCCCACAAAATTTAGTTAACAGCTAAATAACCTGTCAACTGGCTTCAATCTACTTCTCCCGCCATTAAGGAAAAAGGCGGGAGAAGCTAGCCAAAAATTGAAGCTGCTTCTTTAATTTGCAATTTCAATGTATTGCAGTTCATAACAGTAAAGAAGGGTTCACTCCTCTGTGCTGGAGTTACAGTCCATGCTTGCTCAGCCATTTTTACCCCTACCTATGTTCATAAAGCACTGACTATTTCAACTAATCCACAAAGACATTAGGACATTATATTTATTATTTGTACATGGGCGAAACGTAGTACAGCCCTAAGCACTATAATTCGAACAGAACTAGGACAACCCGGAAGCCTAATAGAAGACGACCATGTATACAATGTTATTACCACTTCACCATTCATCATAATTTTTCATAGTAATGTAATTATGATTAGGAAGTTTTTAGAACTAGCTTATTCCTTTAATAATTGGCGCTCCCAGTCATAGCATTTTCCTCGAATAAATAACATAAGCTTCAACTACCACCACCTCCCTACTCTACTGGCATCATCTAACTTTAGAAGCCGGTGCCGGCACTGGTTGGACAGTCTACCCACTAGCGACAATTTATCCCCACCCGGAGCCTCTGTAGATTTAACCATTTTTTCACTACATTTAACTTAGGCATTTCTTCTATTCTTGGAGCTATTAACTTTATTACAACAATTGTAAATATGAAACCGCCAGCCATGACTCAATATCAAGCCCATTGTTTTTGTGTGATCGTCCTAATTACTGCAGTCCTTCTTGCTTTCTCTTCCAGTTCTAGCTGCGGGGATTTCCTGCTGATAACTGACCGTGATTTAAATACCATTTTCTTTGGTCATAACGGGTGGCAAGCATTTATCCTATGCAACGCTTATTCTGATTCTTCGGTCACCCTGAAGTATATATTACAATCTTGGGTTTGGAATAATTTCACACATTGTAACATATTATTCTAATAAAAAAAGAACCGCTGGTTATATGGGCAGTACTAAAGAGCCTGTATCTGTGGTTTCCTGGGCTTTATTGTATGAGCCCATCACATATTCACAGTAGGAATAGATGTAGATACTCGTGCATATTTTTACATCAGCTACCATAATCATTGCTATTCCCACTGGAGTAAAAGTATTTGTAGCTGATTAGCCACTTTTTTGCGGTAATATCAAATGATCTCCCGCAATATTATGAGCCTGGGCTTTATTTTTCTTTTGCAAATAGGTGGACTAACAGGAATTGTGTTAGCTAACTCATCATTAGATATTGTATTACATGATACATACTATGTAGTAGCCCACTTTCACTATGTGTTATCAATAGGGCGTCATTTGCCATTATATAGAAGTTTATTCACTGGTTTAAGCTCTTTTCGAGCTTATGCTGCCAATGACCAAACGTATACCCAAATCCACTTCACCACATTATATTTGTCGGCGTAAATTTAACTTTCTTACAACACTTCCTTGGCTTACATCGGAATACCTCAGCGATACTCAGACTAATGGACATAATATACACATGAAATATCGTCTCATCTATCAAGTTCATTTATTTCCTTTAAGCAGTAGTCCTGATGGTTTATAATTTGAGAAGCTTTCTCTTCAAAGCGAAAGATTATGATACAGACGCTCATCATAATCTACAGAATGATTATACATACCCTCCTCCTTACCACACATTCAGAAAAGAGGCAACTTACGTTAAATCCTAGATGAAAAAGGAAGGATTTGAACCCCAATTAA

>a0f14745-2d81-48da-b5c7-306ec278fba1

TTATGCTTCGTTCAGTTACATGTACTATACCGTTTCTATCCCCACTTGTAGTGTCTTCTACCTGTCTCCTACCCTAACTCCACTAATACTTATAACTTAGAAATTTAGGTTAATAAGACCAAGAGCCTTCAAAGCCTTAGTAAGTATAATTTTACCAATTTCTGCACAACAAATAAGGACTGCAAAACTTTATTCTGCATCAACTGAACGCAAATCAATTACTTTAATTAAGCTAAGCCCTTCCTAGATTGATGGGATTTTAACCCACAAAAATTTAGTTAACAGCTAAATAACTAATCAACTGGCTTCAATCTACTTCTCCCGCCGTTAGGGAAAAAGGCGGGAGAAGCCCCAACAATTAGGCTGCTTCTTTTGAATTTGCAATTCAATGTGATAGTTCACCTCAGGGCTGGTAAAAAGAGGAATTTCACTCCTCTGTCTTCGTTCTGATCTAATGCTTGCTCAGCCATTTTACCCTACCTATGTTCATAAACCACTGATGACTATTTTCAATAATCACGAACATTGGGACATTATATTTATTATTTGGCGCATGGGCGGGGGCAGTAGGTACGGCCCTAAAGCTCCACAATCGAACGGAACGCAGGACAACCCGGAAGCCTAATAGAAGACGACCATGTATCTAATGTTATTGTCACCGCCCACGCATTCATCATAATTTTTTTCATAGTAATACCAATTATGATTGGGGGTTTTCAGGAACTGACTTATTCCTTTAATAATTGGCGCCCCGATATAGCATTTCCTCGAATAAATAACATAAGCTTCGACTTCTACCACACCCTCCCTACTCCTACTGCTTGCATCATCAACTTTAAGAAGCCGGTGCCGGCACTGGTTGGACAGTCTACCCACCCCTAGCAGGCAATTTATCACCAGAGCCTCTGTAGATTTAACCATTTTTTCACTACATTGCTTATGACATTTCTTCTATTCTTGGAGCTATTAACTTTATTACAACAATTGTAAATATGAAACCGCCAGCCATGACTCAATATCAAACTCCGTTGTTTGTGTGATCCGTCTAATTACTGCAGTCTTCTTTACTTTCTCTTCCAGTTCTAGCTGCAGGATTACCATACTATTAACTGACCGTAATTTAAATACTACTTTTTCTTTGATCCTGCTGTTGGCAGCGACCCTATCTATGCAACACCTATTCTGATTCTTCGGTCACCCTGAAGTATATATTCTAATCTTACCAGGTTTTGGAATAATTACACATTGTAACATATTATTCTAATAAAGAACATGGTTATATGGGCATGGTATAAGACCATAATATCTATTGGTTTCCTAGGCTTTATTGTATGAGCCCATCACATATTTCACAGTAGGAATAGATGTAGATACTCGTGCATATTTTACATCAGCTACCATAATCAGTGCTATTCCCACTGGAGTAAAGTATTTAGCTGATTAGCCACACTGCACAGCGGTAATATCAAATGATCTCCCGCAATATTATGAGCCCTGGGCTTTATTTTTCTTTTACCGTGGGTGGACTAACAGATTGTGTTAGCTAACTCATCATTGAATATTGTATTACATGATACATACTATACAGTAGCCCACTTTCACTATGTGTTATCAATGGGAGCAGTATTTGCCGTCAGGGTTTATTT

>112c71e6-4c13-4dd9-ad6f-56ac6e2f04cf

TTGTACTTCGTTTCAGTTACGTATTGTAGAGATTTTGCTAATTTCTGCACAACAAATAAAGGACCGCAAAACTTTATTCTGCATCAACTGAACAAATCAATTACTTTAATTAAGCTAAGCCCTTCCTAGATTGATGGGATTTTAACCACAAAATTTGGTTAACAGCTAAATAACCTAATCAACTGGCTTCAGTACAACTTCTCCCGCCGTTGAGAAAAAAGGCAGAGCCCCGTAGGGTGGAAGCTGCGCTGTACAATTTTGCAGTTCAATAACGATGGTTTCACCTCTTAAGGGCTGGTAAAGGGTTCACTCCTCCAATGTTTAGATTTACAGTCTAATGCTTGCTCAGCATTTTACCCCTACCTATGTTCATAAACCGCTGACTATTTTCAACTAATCCCACAAAGACATTGGGACATTATATTATTTGGCGCATAGGCGGGGGCAATATTAGCCTAACCTCCTAATTCGAACAGGCGGACAACCGGAAACCCTAATAAAGACGACCATATGCAATGTTATTGTCACCGCCACATTCATCGCAATTTTTTCATAAACCAATACCAATTATGATTGGGGTTTGGAACAATATTCCTTTAATAATTAGCGCTCCCGATATAGCATTTCCTCGAATAAATGCTGCCAGCTGACTTCTACCCTCCTACTCCTACTGCTTGCATCGTAACTTTAAAGCCGGTGCCGGCACTAGTTCTTACTGCCCAGGCAATTTATCCCACCCAGGAGCCTCTGTAGATTTCTTTCCTTTTCATTTTGCTTAGTATTTCTTCTAATCTTGGAGCTATTAACTTTATTACAACAATTGTAAGGCATGAAACCGCCAACCATGACTCCAATATCAAACTCCGTTGTTGTGTGATCCGTCTAATTACTGCAGTCCTTCTTTACTTTCTCTTCCAGTTCTAGCTGCAGGGATTACCATACTAACTGACCGTAATTTAAATACTACTTTCTTTGAATCCTGCTGGTGGCGGCAGCCTAACCTATACCAACACTTATTCTGATTCTTCAGTCACCCTGATATATGTTATCTTACCGGGTTTTGGAATAATTTCACACATTGTAACATATTATTCTAATAAAAAGAACCCTTTGGTTATAGGCATAGTATGAGCCATAATATCTATTATTGGTTTCCTAGGCTTTATTGTATGAGCCCATCATATTCACAGTAGGAATAGATGTAGATACTCGTGCTATTTACATCAGCTACCCATAATCATTGCTATTTTCCCACTGGAGTAAAAGTATTTAGCTGATTAGCCACACTACACAGCAGTAATATCAAATAGTCTCACAATATTATAGGCCCTGGGCTTTATTTTCCTTTTTACGTGGGTGGATAACAGGAATTGTGTTAGCTAACTCATCATTAGATATTGTATTACATGATGCTATGCTATATGTTGGCCACTTACATATGTGTAATCCATCTTA

>b3b1b9e8-e84e-4996-9c0f-fa012cad025f

AATGTACTTCGTTCAGTTACGTATTGCTAGTTCACCTCAGGGCTGGCAAAAAGGGTTCACTCCTCTGTCTTTAGATTTACAGTCTAATGCTTGCTCAGCCATTTTACCCTACCTATGTTCATAAACCGCTTTATTCTTGCAACTAATCACAAAGACATTGGGACATTATATTTATTATTTGGCGCATGGGCGGGGGCAGTAGGTACAGCCCTAAGCCTCCTAATTCGAACATTAGGACGGCGGAAGCCTAATAGAAGACTGACCATGTATACAATGTTATTGTCACCACTTAAGCAATACGTAA

>122f8a41-ba57-4a7b-80b3-bf70ca2dc34c

TTGTACTTCGTTCAGTTATTACGTATTGCTTGTGATAGTTCACCTCAGGGCTACTGGTAAGAGGGTTCCTCCTCTGTCTTTAGATTTACAGTCTAATGCTTGCTCAGCCATTTTACCCCTTCACCTATGTTCATAAACCGCTGACTATTTTCAACTAATCACAAAAACATTGGGACATTATATTTATTTGGCGCATGGGCGGGGGCAGTAGGTACAGCCTAAGCCTCCTAATTCAGACAGAACTAGGACAACCCGGAAGCCTAATAGAAGACGACCATGTATACAATGTTATTGTCACCGCCCACGCATTCATCATAATTTTTTTCATATTATTACCAATTATGATTGGGGGTTTTAGGGCAAACTTATTCCTTTAACTTAATTGGCGCTCCCGATAGCATTTCCTCGAATAAATAACATAAGCTTCTGACTACCACCCTCCCTACTCTACTATGCATCATCAACTTT

>abd8d23f-ebed-413b-8d5e-d3ddc957c6c4

TTGTACTTCGTTCAGTTACGTATTGCTAAATTTTTACTTAATTTCTGCACAACAAATAAGAGCTGCAAAACTTTATTCTGCATCAACTGAACGCCAAATCAATTACTTTAATTAAGCTAGCCTTCCTAGATTGATGGGATTTTAACCCACAAAAATTTGATTGGCAATAAATAACTTCTAATCAACTGGCTTCAATCTCTTCCGCCGTTGGGAAAAAGTGGGCTTCAGCAGAATTGAAGCTGCTTCTTTGAATTTGCAATTCAATGTGATAGTTCACCTCAGGGCTGGTAAAAGAAGTTTAACTCCTCTGTCTTTAGATTTACAGTCTAATGCTTTGCTCATTGTTTTACCCTACCTATGTTCATAAACCGCTGACTATTTTTCAACTAATCACAAAGACATTGGGACATTATATTATTTGGCGCATGGGCGGGGGCGAGTAGGTACAGCCTAAGCCTCTAGACCGGGCAGAACTAGGACAACCCGGAAGCCTAATAGAAGACGACCATGTATACAATGTTATTGTCACCGCCCACGCATTCATCATAATTTTTTTCATAGTAATACCAATTATGATTGGGGTTTTGGAACTGACTTATTCCTTTAATAATTGAAAAAACCCAATTACTTAGCATTTCCTCGAATAAATAACACTTCTGACTTCTACCACCCTCCCTACTCCTACTGCTTGCATCATCAACTTTAAAAGCCGGTGCCGGCACTGGTTGGACAGTCTACCCACCCTAGCAGGCAATTTATCCCACCCAGGAGCCTCTGTAGATTTAACCATTTTTCCACTACATTTAGCAGGCATTTCTTCTATTCTTGGAGCTATTAACTTTATTACAACAATTGTAAATATGAAACCGCCAGCCATGACTCAATATCAAACTCCGTTGTTTGTGTGATCCGTCCTAATTACTGCAGTCCTTCTTTTACTTTCTCTTCCAGTTCTAGCTGCAGGGATTACCATACTATTAACTGACCAATAATTTAAATACTACTTTTCTTTGATCCTGCTGGTGGCGGCGACCCTATCCTATACCAACACTTATTCTGATTCTTCAGGTACCTAGTATATATTCTAATACTTACCAGGTTTTGAATAATTTCACACATTGTAACATATTATTCTAATAAAAAAGAACCACTTGATTTATGGGCGGTATGAGCCATAATATCTGTTGGTTTCCTAGGCTTTATTGTATGGGCCATCATATTCACAGTAGGAATAGATGTAGATACTCGTGCATATTTTACATCAACTACCATAATCATTGCTATTCCCACTGGAGTAAAAGTATTTAGCTTAGCCACACTGCGGCGGTAATATCAAATGATCTCCCGCAATATTATGAGCTGAAGCTTTATTTTCTTTTACCGTGGGTGGACTAACAGGAACAGCTAGCTAACTCATCATTAGATATTGTATTACATGATACATACTATGTAGTAGCCCACTTTCACTATGTGTTATCAATAGGAGCAGTATTTGCCATTATAGGGGGATTTATTCACTGATTCCGCTCTTTTCAGGCTATACTCTTGACCAAACATATGCCAAAATCCACTTCACCATTATATTTGTCGGCGTAAATTTAACTTTCTTCCCACAACACTTCCTTAGCTTATCTCCGGAATACCTCGACGATACTCAGACTACCGAACGCCTTTTTCATGATATCACGTCTCATCTATCGGTTCATTTATTTCACTTGGCAATGCTGAAAGTGGTTTTTATAATTTGAGAAGCTTTCTCTTCAAAACGAAAAGTCTTAGCCATTGAGCAACTATCCACCAATCTAGAATGATTATGGCTGCCCTCCTCCTTACAGCAATACGTGGCA

>5544a601-55c9-4af9-b88a-2dfcd691283b

ATTGTACTTCGTTCCAGTTGCGTGTTGCTTATCCCCACTTGTAGTGTCTTCTACCTGTCTCCTACCTAACTCCACTAATACTTATAACTTAGAAATTTAGGTTAATAAGACCAAGAGCCTTCAAAGCCTTAGTGATAAATTTTTATGAATTTCTGCACAACAAATAAGGACTGCAAAACTTTATTCTGCATCAACTGAACGCAAATCAATTACTTTAATTACGCTAAGCCCTTCCTAGATTGATGGGATTTTTAACCCACAAAATTTAGTTAACAGCTGATAACTAAATCAACTGGCTTCAATCCTTCTCCCGCCGTTAGGGAAAAAAGGCGGGGGAGAAGCCCCGACAGGGTGAAGCTGCTTCTTTAATTTGCAATTCAATGTGATAGTTCACCTCAGGGCTGGTAAAAGAGGGTTCACTCCTCTGTGCTTTAGATTCTGATCTAATGCTTGCTCGGCCATTTACCCCTACCTATGTTCATAAACCATGACTATTTTCAACTAATCACAAAGACATTGGGACATTATATTTATTATTTGGCGCATGGGCGGGGCAGTAGGTACAGCCTAGCCTCCTAATTCGAACAGAACTAGGACAACCCGGAAGCCCTAATGAAGACGACCCATCTAGCATACAATGTTATTGTCACCGCCCACTGCATTCGTCGCCCAATTTTTTTCGCCAGTAATACCAATTATGATTGGGGGTTTGGGGCGATATTCCTTTAATGATGGCGGCTCCCGATATAGCATTTCCTCGAATAAATAACATAAGCTTCACCTGACTTCTACCACCTCTACTCACTGCAACGCATCTCATCAACTTTAGGACCAAATTGCCTTTGGTTGAACAGTCTGCCCACCCCTAGCAGGCAATTTATCACCCCAGGAGCCTCTGTAGATTTAACCATTTTTTCACTGCATTTAGCAGGCATTTCTTCTATTCTTGGAGCTATTAACTTTATTTACAGCAATTGTAAATATGAAACCGCCAGCCATGACTCAATATCAAACTCCGTTGTTTGTGTGATCCGTCCTAATTACTGCAGTCCTTCTTTTACTTTCTCTTCCAGTTCTAGCTGCAGGGGATTACCATACTATTAACTGACCGTAATTTAAATACTACTTTCTTTGATCCTGCTGGTGGCGGCGACCCTATCCTATACCAACAGCAATACGTAA

>45921338-bec0-4c13-8458-9ce7756cd84b

GGTATACTTTGTTCAGTTACGTATTGCTGATGGGATTTTAACCCACAAAATTTAGTTAACAGCTAAATAACCTAATCAACTGGCTTCCAATCTACTTCTCCCGCCGTTAGGAAAAAAAGGCGGAGAAGCCCCGGCTGAGTGTGACTGCTTCTTCAATTTGCAATTCAATGTGATAGTTCACCTCAGGGCTGGTAAAAAGAGGTTCACTCCTCTGTCTTTAGATTTACAGTCTAATGTAGCATAAAGCCATTTTACCCCTACCTATGTTCATAAACCGCTGACTATTTTCAACTAATCACAAAGACATTGGGACATTATATTTATTATTTGGCGCATGGGCGGGGGCAGTAAGGTACAGCCCTAAGCCTCCTAATTCGAACAGAACTAGGACAACCCGGAAGCCTAATAGAAGACGATATGTATGCAATGTTATTGTCACCGCCCACGCATTCATCATAATTTTTTCATAGTAATACCAATTATCAGTTGGGGGTTTGAACTGACTTATTCCTTTAATAATTGGCGCTTTCCCGATATAGCATTTCCTCGAATAAATAACATAAGCTTCTGACTTCTACCACCCTCCCTACTCCTACTGCTTGCATCATCAACTTTAGAAGCCGGTGCCGGCACTGGTTGGACAGTCTCCACCCTAGCAGGCAATTTATCCACCAGAACCTCTGTAGATTTAACCATTTTTTCACTACATTTAACCCAGGCATTTCTTCTATTCTTGGAGCTATTAACAGCAATACGTAACA

>5114f099-e968-4897-a0c9-e4aeaf3eedc4

ATTGTACTTCGTTCAGTTACGTATTGCTCAAATAACACAAAAATCCAACTGGCAACTAAATTATATAAAGCCAATACCGTTTCTATCCCCACTTGTAGTGTCTTCTACCTGTCTCCTACCCCTAACTCCACTAATACTTATAACTTAGAAATTTTAGGTTAATAAGACAGAGCCTTCAAAGCCCTTAGTAAGTAAATTTTACTTAATTTCTGCACAACATAGGACTGCAAAACTTTATTCTGCATCAACTGAACGCAAATCAAATTACTTTAGTCTGTCATCTTTCTAGATTGATGGGATTTTAACCCACAAAATTTAGTTAACGCTAAATAACCTAATCAACTGGCTTCAATCTACTTCTCCCGCCGTTGGGAAAAAAAGGCAGAGCCGCTAGAATTAAGCTGCTTCTTTAATTTGCAATTCAATGTGATAGTTCACCTCAGGGCTGGTAAAAAGAGGGTTCACTCCTCTGTCTTTAGATTTACAGTCTAATGCTTGCTCCAGCCATTTACCCTACCTATGTTCATAAACCGCTGACTATTTTCAACTAATCACAAAGACATTGGGACATTATATTTATTATTTGGCGCATGGGCGGGGGCAGTAGGCACGACCTAAGCCTCCTAATTCGAACAGAACGGACAGCGGGAAGCCTAATAGAAGACGACCATGTATACAATGTTATTGTCACCACTCCCACATTCATCATAATTTTTTTCATGACATAATTATGATTGGGGGTTTTGGGGAACTGACTTATTCCTTTAATAATTTTGGCGCTCCCGATATAGCATTTCTCGAATAAATAACATAAACTTCGACTTCTACCACCCTCCCTACTCCTACTGCTTGCATCATCAACTTTAGAAGCCGGTGCCGGCACTGGTTGGACAGTCTACCCCACCCCTAGCAGGCAATTTATCCCACCCAGAGCCTCTGTAGATTTAACCATTTTTTCACTACATTTAGCAGGCATTTCTTCTATTCTTGAGCTATTAACTTTATTACAACAATTGTAAATATGGAATATAGCCATGACTCAATATCAAACTCCGTTGTTCTTGTGATCCGTCCCTAATTACTGCAGTCCTTCTTTTACTTTCTCTTCCAGTTCTAGCTGCAGGGATTACCATACTATTAACTGACCGTAATTTAAATACTACTTTCTTTGATCCTGCTGGTGGCGGCGACCCTATCTATACCAACACTTATTCTGATTCTTCGGTCGCCCTGAAGTATATATTCTAATCTTACCGGGTTTTGGAATAATTTCACATTGTAACATATTATTCTAATAAAAAAGAACCCTTTGGTTATATGGGCATGGTATGAGCCATAATATCTATTGGTTTCCTGGTTTTATTGTATGAGCCCATCACATATTCACAGTAGGAATAGATGTAGATACTCGTGCATATTTTACATCAGCTACCATAATCATTGCTATTCCCACTGGAGTAAAAAGTATTTAGCTGATTAGCCACACTGCACGGCGGTAATATCAAGTGATCTCCCGCAATATTATGAGCCCTGGGCTTTATTTTTCTTTTACCGTGGGTGGACTAACAGGAATTGTGTTAGCTAACTCATCATTAGATATTGTATTACATGATACATACTATGTAGTAGCCACTTTCACTATGTGAGCAATACGTAACA

>4bf681de-8384-4684-90be-64e2eed4b835

TTATCACTTCGTTCAATTACATGCTAAACTAACAAAAATCAACTGGCAACTAAATTATATAAAGCCAATACCGTTTCTATCCCCACTTGTAGTGTCTTCTACCTGTATCTCTCCTACTAACTCCACTAATACTTATAACTTAGAAATTTAGGTTAATAAGACCAAGAGCAACTTCAAAGCCCTTAGTAAGTAAATTTTACTTAATTTCTGCACAACAATAAGAACTGTAAACTTTATTCTGCATCAACTGAACGCAAATCAATTACTTTAATTAAGCTAAGCCCTTCCTAGATTGATGGGATTTTAACACAAAAATTTAGTTAACAGCTAAATAACCCTAATCAACCAGCTTCAATCTGCTTCTCCCGCCGTTAGGGAAAAAAAGGCGGGAGAAGCCCCGGCGAAGATAGGCTTCTTCTTTGAATTTGCAATTCAATGTGATAGTTCACACCTCAGGGCTGGTAAAAGGAGTTCACTCCTCTGTCTTTAGTTACAGTCTAATGCTTGCTCAGCCATTTTACCCCTACCTATGTTCATAAACCGCTGACTATTTTCAACTAATCACAAAGACATTTAGGACATTATATTTATTATTTGGCGCATGGGCAGGGGCAGTAGGTACAACCCTAAGCCTCCTAATTCGAACAGAACTAGGACAACCCGGAAGCCTAATAAAAGACGACATGTATACAATGTTATTGTCACCGCCCACGCATTCATCATAATTTTTTTCATAGTAATACCAATTATGATTGGGGGTTTGGGAGCAGCTTATTCCTTTAATAATTAGCGCTCCCGATACTTGTTCCCTCGAATAAATAACATAAGCTTCCCAAGTGACTTCTACCACCCTCCTACTCCTCTGCTTGCATCATCAACTTTGAAGCCGATTGCCGGCACTGGTTGGACGATCCTACCCACCCCTGACAGGCAATTTATCCCGCAGGGCCTCTATGGTTGTACCATTTTTTCACTACATTTAGCAGGCATTTTCTTCTATTCTTGGAGCTATTAACTTTATTACAACAATTGTAAATATGAAACCATAAAGCCATGACTCAATATCAAACTCCGTTGTTTGTGTGATCCGTCCTAATTACTGCAGTCCTTCTTTTACTCTTCCAGTTCTGGCTGCGGGGATTGCCATACTGTTAACTGACCGTAATTTAAATACTACTTTCTTTGATCCTGCTGGTGGCGGCGACCAGCAATGCGTAA

>9669b951-3353-4956-bcfc-bd09b4ca753a

TGTGTACTTCGTTCAGTTACATGTGCTCCTACCTAACTCCACTATGCCACTTAGAAATTTGAGTTAATAAGACCAAGAGCCTTCAACCCTTAGTAGTAGAATTTTACTTAATTTCTGCACAACAAATAAGGACTGCAAAAACTTTATTCTGCATCAACTGAACGCAAATCAATTACTTTAATTAAGCTAAGCCTTCCTAGATTGGTAGGATTTTAACCCACAAAAATTTAGTTAACAGCTAAATAACCTAATCAACTGGCTTCAATCTGCTTCTCCCGCCGTTAGGGAAAAAAGGCGGGAGAAGCCCCGGCCTAGGAGTGAAGCTGCTTCTTTGAATTTGCAATTCAATGTGATAGTTCACCTCAGGGCTGGTAAAAGAGGGTTCACTCCTCTGTCTTTGATTTACAGTCTAATGCTTGCTCAGCCATTTACCCTACCTATGTTCATAAACCTGACTATTTTCAACTAATCACAAAGACATTGGGACATTATATTTATTATTTGGCGCATGGGCAGGGGCGGTAGGTACAGCCCTAAGCCTCCTAATTCGAACAGAACTAGGACAACCCGGAAGCCTAATAGAAGACGACCGTGTATACAATGTTATTGTCACGCCCACATTCATCATAATTTTTCTTAGTATACCAATTATGATTGGGGGTTACACTGGGAACTTATTATTCCTTTAATAATTGGCGCTCCCGATATAGCATTTCCTCGAATAAATAACATAAGCTTCAGCTTCTACCACCTCTACTCCTACTGCTTGCATCAGCATCAACTTTGAGAAGCCGGTGCCGGCACTGGGTTGGACAGTCACCACCCCTAGCAGGCAATTTATCACCAGGAGCCTCTGTGATTGCTCATTTTTTCGCTACATTTAGCAGGCATTTTCTTCTATTCTTGGAGCTATTAACTTTATTACAACAATTGTAAATATGAAACCACCAATATGACTCAATATCAAACTCCGTTGTTTGTGTGATCCGTCCTAATTACTGCAAGTCCTTCTTTTACTTTCTCTTCCAGTTCTAGCTGAGATTACCATACCTGAATAACTGACCGTAATTTAAATATACTTTCTTTGATCCATAAGGTGGCGGCGACCCTATCTATACCAACACCTTATTCTGATTCTTCGGTCACCTAGTATATATTCTAATCTTACCGGGTTTTGGAATAATTTCACACATTGTAACATATTATTTCTAATAAAAAGAACCCTTTAGTTATATGGGCATGGTATGAGCCATAATATCTATTGGTTTCCTAGGCTTTATTGTATGAGCCCATCACATATTCACAGTAGGAATAGATGTAGATACTCGTGCATATTTTACATCAGCTACCATAATCATTGCTATTCCCACTGGAGTAAAAGTATTTAGCTGATTAGCCACTGCACGGCGGTAATATCAAATGATCTCCCGCAATATTATGAGCCCTGAGCTTTATTTTTCTTTTTACCGTGGGTGGACTAACAGGAATGTGTTAGCTAACTCATCATTAGATGTTGTATTACATGATACATACTATGTAGTAGCCCACTTTCACTATGTGTTATCAATAGGAGCAGTATTTGCCATTATAGGGAGAGTTATTCACTGATTCCGCTCTTTTCAGGCTATACTCTTGACCAAACATATGCCAAAATCCACTTCCACCATTATATTTGTCGGCGTAAATTTAACTTTCTTCCCCAACCACTTCCTTGGCCATCCGGAATACCTCGACGATATAGACTATCCAGACACATATACATACAGCGATATCAATATATCTATCAGTTCATTATTTCACTTACAGCAGTAGTCCTGATGGTTTTATAATTTGAAGCTTTCTCTTCAAAACGAAAAGTCTTAGCCATTGAGCAATCCGCAATCTAGAATGATTATGCGGCTGCCTCCTCCTTGTCACATTCGAAGAGAGCAACTTACGTTAAATCACAAGGCGAAAAAGGAAGGATTTGAACCCCCAAAAATTGGTTTCAAGCCAATCCCATATACCCTATGACTTTTCAATAAGATATTAGTAAAATAATTACATAACTTTGTCAAAGTTAAATTATAGACTAAATATCTATATATCTTAATAACAACACCAGCTCAACTAGGTTTACAAATGCACATCACCTATCATAGAAGAACTTATTGCTTTCACGACTTATGCACTCATAATTATTTTTCCTGATTAGTTCCTTAGCAATACGTAA

>1f86d728-1335-4ba3-9a53-065541d55fc3

GTGTACTTCGTTCAGTTACGTATTATATAAAACTTTATTCTGCATCAACTGAACGCAAATCAATTACTTTAAATTAAGCTAAGCCCTTCCTAGATTGATAGATTTTAACCCACAAAATTTAGTTAACAGCTAAATAACCTAATCAACTGGCTTCAATCTGCTTCTCCCGCCGTTAGGAAAAAAGGCGGAAGCCCTGGCAAATTGAAGCTGCTTCTTCAATTTGCAATTCAATGTGATGATTCACCTCTCAGGGCTACAGGTAAAAGAGGGTTCCACTCCTCTGTCTTTAGATTACAGTCTAATGCTTGCTCAACCATTTACCCCTACCTATGTTCATAAACCATGACTATTTTCAACTAATCACAAAGACATTGGGACATTATGTTATTATTTGGCACATGGGCGGGGGCAGTAGAGTACAGCCTAAGCCTCTAATTCGAACAGAACTAGGACAACCCGGAAGCCTAATAGAAGACGACCATGTATACAATGTTATTGTCACCGCCCGCATTCATCATAATTTTTTTCATAGTATTACCAATTATGATTGGGGGTTTTTGGGAACTGACATTTCCTTTGCTAATTGGCGCTCCCGATATAGCATTTCCTCGAATAAATAACATAAGCTTCTGACTTCTACCACCCTCCCTACTCCTACTGCTTGCATCATCAACTTTAGAAGCCAGTGCCGGCACTGGTTGGACAGTCTACCCACCCTAGCAGGCAATTTATCCCACCCAAGCAATGG

>653ab765-7f2e-4e53-a707-3bf56bfd09ec

ATTGTGCTGAACAGTTACGTATTGCTACAGCTAAATAACCTAATCAACTGGCTTCAATCTACTTCTCCCGCCGTTAGGGAAAAAAGGCAGAGGCCCCGGCAGGTAAGCTACGCCCCAACGTTTGCAATTCAATGTGATAGTTCACACTCAGGGCTGGTAAAAAGGGTTCCACTCTACTGTCTTTAGATTCCTGATCTAATGGTTGCTCAACCATTTTACCCTACCTATGTTCATAAACCGCTGTGCACCAACTAATCACAAAGAACATTGGGACATTATATTTATTATTTGGCGCATGGGCGGGGCAATGGTACAGCCCTAAGCCTCTAATTCGAACAGAACTAGGATACAACCCGGAAGCCTAATAGAAGAACGACCATGTATACAATGTTATTGTCACCGCCCACACATTCATCATAATTTTTTTCATAGTAATACCAATTATGATTGGGGGTTTTGGGAACTGACTTATTCCTTTAATAATTGGCGCTCCCGATATAGCATTTCCTCGAATAAATAACATAAGCTTCTGACTTCTACCACCACTCCCTACTCCTACTGCTTGCATCATCAGCACAGAAGCCGGTGCCGGCACTGGTTGAACTTGATCTACCCACCCCTAGCAGGCAATTTATCCCACCAGGAGCCTCTGTAGATTCCCAACCATTTTTTCACTACATTTAGCAGGCATTTCTTCTATTCTTGGAGCTATTAACTTTATTACAACAATTGTAAATATGAAACCGCCAGCCATGACTCAATATCAAACTCCGTTGTTTGTGTGATCGTCCTAATTACTGATCCCTTCTTTTACTTTCTCTTCAGTTCTAGCTGCAGGGATTACCATATTAACTGACCGTAATTTAAATACTACTTTCTTTGATCCTGCTGGTGGCGGCGACCTATCCTATACCAACACTTATTCTGATTCTTCGGTCACCCTGAAGTATATATTCTAATCTTACCGGGTTTTGGAATAATTTCACACATTGTAACATAATATTCTAATAAAAAAGAACGCTTTGGTTATATGGGCATGGTATGAGCCATATCTATTGGTTTTCTAGGCTTTATTGTATGAGCCCATACGCGTACAGTAAAGCAATACGTAACTTC

>3270f21d-3422-4244-a9b8-a48ef77a4666

TTGTGCTTAGTTTCAGTTACGTATTGCTAACATCAAATAACACAAAAATCAACTGTACGCTAAATTATATAAAGCCAATACGTTTCTGTCCCCACTTGTAGTGTCTTCTGCCTGTCTCCTACCTAACTCCACTAATACTTACTAACTTAGAAATTTAGGTTAATAAGACCAAAAAGCCTTCAAAGCCCTTATTAAGTAAATTTTACTTAATTTCACGCACAACAAATAAGGACGCAAAACTTTTATTCTGCATCAACTGAACGCAAATCAATTACTTTAATTAAGCTAAGCCCTTCCTAGATTGATGGATTTTAACCCACAAAAAATTTAGTTAACAGCTAAATAACCTAATCAACTAGCTTCAATCTATTGTCACATAGTTAGGAAAAAGAGCGGGAGAAGCCTAAACTGAATTAAGGCTGCTTCTTTGAATTTGCAATTCATCCATGATAGTTCACCTCAGGGCTGGTAAAAAGAGGGTTCACTCCTCTGTCTTTAGATTTACAGTCTAATGCTTGCTCAGCCATTTTACCCCTACCTATGTTCATAAACCGCTGACTATTTCCAACTAATCACAAAGACATTGGGACATTATATTTATTATTTGGCGCATGGGCGGGAGCGTGGAGTACGGCCCTAAGCCTCCTAATTCAGACAGAACTAGGACAACCCCGGAAGCCTAATAGAAGACGACCATGTATACAATGTTATTGTCACCGCCCACGCATTCATCATAATTTTTTTCATAGTAATACCAATTATGATTGGGGTTTTTGAGGCAGCTTATTCCTTTAATAATTGGCGCTCCCGATATAGCATTTCCTCGAATAAATAACATAAGCTTTACAGCTTCTACCACCCTCCTACTCCTACTGCTTGCATCATCAACTTTAGAAGCCGGTGCCGGCACTGGTTTAGACAATCTACCCACCCCTAGCAGGCAATTTATCCCACCCAGGAGCCTCTGTAGATTTAACCATTTTTTCACTACATTTGGGCATTTCTTCTATTCTTGGAGCTATTAACTTTATTACAACAATTGTAAATATGAAACCGCCAGCCATGACTCAGTATCAAACTCCGTTGTTTGTGTGATCTAATCCTAATTACTACCAGTCACTTCTTTACTTTCTCTTCCAGTTCTAGCTGCAAGGGATTACCATACTATTAACTGACCGTAATTTAAATGCTACTTTCTTTGATCCTGCTG

>b95b898c-b84f-49d2-bd89-adf1358689a3

GGTATGCTTCGTTCAGTTACGTATTGCTCCCGCCTGTTTACCAAAAACATCACCTCTAGCATTTCTAGTATTAGAGGCACTGCCTGCCCAGTGACATATGTTCAACGGCCGCGGTACCCTGACCGTGCAAAAGGTAGCATAATCACTTGTTCTCTAAATAGGGACTTGTATGAATGGCCACACGAGGTTTAACTGTCTCTTACTTTTAATCAGTGAAATTGACCTATCCGTGAAGAGGCGGATATACATAAATAAGACGAGAAGACCCTGTGGAGCTTTAATTTAATGATACAAACTGATTTGTAAAACCAACAGGCATTAATTTACCGTCAATGTATTATAAATTTCGGTTGGGGCGACCTCGGAGTAAAATAGAACCTCCGAAAAACATATACCAAGACCTTACCAGTCAAAGTAAACAACACCTATTGACCCAATAATTTGATCAACGGACCAAGTTACCTAGGGATAACAGCGCAATCCTATTTTAGAGTCCATATCGATAATAGGGTTTACGACCTCGATGTTGGATCCAAGACATCCTAATGGTGCGAAAGCTATTAAGGGTTCGTTTGTTCAACGATTAAAGTCTTACGTGATCTGAGTTCAGACCGGGTAATCCAGGTCGGTTTCTATCTATTTAAATATTTCTCCCAGTACGAAAAGGACAAGAGAAATGGGGCCCACTTTCATAAAAGCGCCCTCAACAATCAGATGACCTCCATCTCAACCTCACATATTATAATCTTGCCCAAGAACAGGGCTCGTTAAGGTGGCAGAGCCCGGTAGTTGCATAAAACTTAAAACTTTATAATCAGAGGTTCAATTCCTCTTTAACAACATGTATATAATTAATTTACTAATACTAGTCCTACCTGCCTAATTGCCATAGCCTTTCTAACACTCACAGAACGAAAAAATCTGGGCTATATACAATTCCGAAAAGGCCCATATTGTGAGCCCCTACGAATACTTCAATAATCGCTGACGCCATAAAACTCTTCACAAAAAGAACCCTTATTACCTACCACATCCACCATAACTTTATATTTAACTGCCCCCACCTAGCTCTTTCCCATTGCTCTTCTACTATGAACGCCACTCCCCATACCATATCCTCTTATCAACTTCAATCTTGGTCTCTATTTATCCTCGCAACATCAAGCCTAGCTGTTTACTCAATTTTATGATCTGGCTGAGCATCCAACTCAAACTACGCACTAATTGGCGCACTACGAGCTGTAGCCCAAACAATCTCATATGAGGTCACCCTTGCCATTATCTTACTATCAACACTACTAATAAAGCAGCTCATTCAATCTACAATCGCTTATTACCACTCAAAGAACACTACTGACTTCTACTTCCATCATGGCCCCTAATAATATGATTTATTTCCACATTAGCAGAAACTAATCGAGCTCCATTCGACCTGACAGAAGGCGAATCAGAACTAGTATCCAGGTTTCAACATTGAATACGCTGCAGGCTCATTCGCCTTATTCTTCATAGCAGAGTATATAAACTATTATTATAATAAATGCCCTAACTACCACTATTTTCTTATCCACACCCTACAATATAATTATACCAGAAACATTTACTATTAATTTTATAGCCAAAACCCCTCTACTAACCACTTTATTTTTTTATGAATTCGAACAGCCTACCCCCGCTTCCGCTACGATCAATTAATATTCTTACTATGAAAAAACTTTTTACCACTTACATTAGCACTATGTATATGATATGTTTCAATACCCATCCTAGCATCTGTCCCACCCCAAACATAAGAAATATGTCTGATAAAAGAGTTACTTTGATAGAGTAAATTATAGAGGTTCAAACCCTCTTATTTCTAGGATTACAGGAATTGAACCTACACCTGAGAACTCAAAACTCTCCGTGCTACCGATTACACCATATCCTAAACAGTAAGGTCAGCTAAATAAGCTATCGGGCCCTTGAAAATGTTGGTTTTAATCCTTCCGTGCTAACATTAATCCTCTAGCCCACCTTATTATCTCCTTCACCATTCTAACAGGGACCGTAATCACAATTTTAAGCTCACATTGATTCTAGCCTGAATAGGCTTAGAATTAAATATACCAACCATCGTACCAATCCTTGCCAAAAGTACCAATCCCCGCTCCACAGAGGCATCCACCAAATATTTTTTAATTCAAGCAACAGCATCAATACTTCTATTAGTATCCATTTTTCTTAACAATCTACTAACTCAACAATGAACAATCCAATCCTCCTTATAACCAAATATTATCCACAATAATATTTATTGCTCTAGCAATAAAGATAGGGATAGCCCACTTCACCTTCTGACTCCCAGAAATTACCCAAGGAATCCCTCTAATCCCAGCTATAATTATTCTCACGTGACAAAACTCGCCCCAATATCAATTTCTCCTCAAATTTTTCCGTCAACAAACCTAAACTTGATTCTAACAATCTCAGTTCTATCAATTATAATTGGCAGCTGAGGAGGACTCAACCAAACACAACTCACCAAAATCCTAGCCTATTCTTCAATTACTCACATAGGATGAATAATAGCAGTATTATATTACGACCTAATATTACTATATTAACTTTAATTATTTATATTTTCCTAACAATCTCTACATTAATAATCTTTTATTTTAACCTCAAATGTGCTAACCCTATCCCTATCAGAGCTACCTGAAACAAACTAGCATGAACAATACCCATTATTCCACTAATAATATCCCTAGGAGGTCTACCCCCACTAACAGGTTTTCCCCCAAATGAGCTATTATACAAGAACTTATTAAAAATGATAACTTAATTATTCCCTTATAATAGCTTTACTAACATTAATAAATTTATATTTTTATATACGTTTAATATATTATATCTCAATGACAATATTCCCAACATCAAATAACACAAAAATCAACTGGCAACTAAATTATATAAAGCCAATACCGTTTCTATCCCCCCACTTGTAGTGTCTTCTACCTGTCTCCTACCCTAACTCCACTAATACTTATAACTTAGAAATTTAGGTTAATAAGACCAAGAGCCTTCAAAGCCCTTAGTAAGTAAATTTTACTTAATTTCTGCACAACAAATAAGGACTGCAAAACTTTTATTCTGCATCAACTGAACGCAAATCAATTACTTTAATTAAGCTAAGCCCTTCCTAGATTGATGGGATTTTAACCCACAAAATTTAGTTAACAGCTAAATAACCTAATCAACTGACTTCAATCTACTTCTCCCGCCGTTAGGGAAAAAAGGCGGGAGAAGCCCCGGCAGGGTAGAGCTGCTTCTTGAATTGCAATTCAATGTGATAGTTCACCTCAGGGCTGGTAAAAAGAGGTTCTCCTCTGTCTTTAGATTTACAGTCTAATGCTTGCTCAGCCATTTTACCTACCTATGTTCATAAACCGCTGACTATTTTCAACTAATCACAAAGACATTGGGACATTATATTTATTATTTGGCGCATGGGCGGGGGCAGTAGGTACAGCCTAAGCCTCCTAATTCGAACAGAACTAGGACAACCCGGAAGCCCTAATAGAAGACGACCATGTACGCAATGTTATTGTCACCGCCCACGCATTCATCATAATTTTTTCATAGTAATACCAATTATGATTGGGGTTTTTTGGGAACTGACTTATTCCTTTAATAGCCCACGGCGCTCCCGATATAGCATTTTCCTCGAATAAATAACATAAGCTTCCTGACTACCGCCACCCTCCTACTCCTACTGCTTGCATCATCAACTTTAGAAGCCGGTGCCGGCACTGGTTGGACAGTCTACCCACCCCTAGCAGGCAATTTATCCCACCCAGGAGCCTCTGTAGATTTAACCATTTTTTCACTACATTTAGCAGGCATTTCTTCTATTCTTGGAGCTATTAACTTTATTACAACAATTGTAAATATGAAACCGCCAGCCATGACTCAATATCAAACTCCGTTGTTTGTGTGATCCGTCCTAATTACTGCAGTCCTTCTTTTTACTTTCTCTTCCAGTTCTAGCTGCAGGGATTACCATACTATTAACTGACCGTAATTTAAATACTACTTTCTTTTGATCCTGCTGGTGGCGGCGACCCTATCCTATACCAACACTTATTCTGGAGTGCTGGTCACCTGAAGTATATATTCTAATCTTACCGGGTTTTGGAATAATTTCACACATTGTAACATATATTATTCTAATAAAGAACTTAACTGGTTATATGGGCATGGTATGAGCCATAATATCTATTGGTTTCCTAGGCTTTATTGTATGAGCCCATCACATATTCACAGTAGGAATAGATGTAGCAGATACTCGTGCATATTTTACATCAGCTACCATAATCATTGCTATTCCCACTGGAGTAAAAGTATTTAGCTGATTAGCCACACTGCACGGCGGTAATATCAAATGATCTCCGCAATATTATGAGCCCTGGGCTTTATTTTCTTTTTACCGTGGGTGGACAACAGGAATTGTGTTAGCTAACTCATCATTAGATATTGTATTACATGATACATACTATGTAGTAGCCCACTTTCACTATGTGTTATCAATAGGAGCAGTATTTGCCATTATAGGGGGATTTATTCACTGATTCCCGCTCTTTCCCAGGCTATACTCTTGACCAAACATATGCCAAAATCCACTTCACCATTATATATTTGTCGTAATTTGCTTTTCTTCCCACAACACTTCCTTGACTTATGGAATACCTCAACGATACTCAGACTATCCAGACGCATATACTACATGAAATATCGTCTCATCTATCAGGTTCATTTATTTCACTTACAGCAGTAGTCCTGATGGTTTATAATTTGAGAAGCTTTCTCTTCAAAACGAAAAGTCTTAGCCATTGAGCAACTATCCACCAATCTAGAATGATTATACGGCTGCCTCCTCCTTACCACACATTCGAAGAGGCAACACGTTAAATCCTAGACGAAAAAGGAAGGATTTGAACCCCCAAAAATTGGTTTACTGACAATCCCATATACCCTATGACTTTTTCAATAAGATATTAGTAAAATAATTACATAACTGTCAAAGTTAAATTATAGACTAAATATCTATATATCTTAATAGCAACACCAGCTCAACTAGGTTTACAAAATGCCACATCACCTATCATAGAAAGAACTTGTTACTTTCCACGACCATGCACTCATAATTATTTTCCTGATTAGTTCCTTAGTCTTATATATTATTTCCCTTATACTTACCACAAAACTCACTCATACTAGCACCATAAATGCTCAAGAGATCGAAATAATCTGAACTATTCTCCTGCACTGATTCTAATTACAATTGCCCTCCCATCACTGCGTATTCTATATATGACAGACGAATTTAATAAACCTTATTTAACCCTTAAAGCAATTGGTCACCAATGATACTGAACCTATGAATATTCCGACTATGAAGACTTAGTATTTGACTCTTACATTAATATATTTCCTTGAGCCAGGGGAATTTCGACTCCTCGAAGTTGATCACCGAACAACTGCCCACGAAGCGGATATTCGCATATTAATCTCATCACAAGACGTCTTACACTCATGAGCCGTACCATCACTAGGCGTAAAAACAGATACAATTCCTGGACGTTTAAACCAATGCAACTTCTAATTTATTTGCGGACAATGCTCGGAAATTTGCGGGTCCAATCACAGCTTTATACCTATTGTTCTAGAATTCATTTATTTCCAAGATTTCAATGAGCTTCATACTTATATATTGTATCGCTATATAAGCTAACTTAGCATTAACCTTTTAAGTTAAAGATTGAGGAGAACAAACTCTCTATAGTGAATGCCTCAACTAAATATTTCACCGTAATAGTAATTATATCTATAATTATTTACCTTATTTTATATTATACAATTGAAAATACTGAACTTTACTTTCCATTATTACCCACTGCAAAATTAGTAGAAACACAAAAACATAAAACAACTTTGAGAACTAAAATGAACCAAAATCTATTTGCCTCATTAAAAATATTCCAACAATACTAGAATTGCCTGTATTTTTAATTATTGCACTCCCCACTACATTAATTTTATCCTCCAAAAAACTTATTCAACAACCGACTCTCTTCAATTCAACAATGCTGAATTCAGCTAACACTTAAACAAATAATATTAACCCACACCACTAAAGGGCGAACCTGATCCCTTATACAAGCCTAATTTCTTTTATTGCCCTAAATAACATTCTCGGACTTACACCATATGCATTTACACCAACCACCCAACTGTCAATAAAATCTAGGCATAGCCTTTATTCCTCTATGAGCAGCAACTGTACTAATAGGCCTCCGATTTAAAACAAAATCATCCCTCGCTCATTTTTTACCACCAAGGAACACCAATTCCACTAATCCCTATATTAATTATTATTGAAACAATTAGTCTATTCATTCAACCTGTAGCCTTAGCCGTACGATTAACAGCCAATGTGCAGCAGGTCTTATGTGTGCATTTACTTGGGGATACAACGCATTAACTCTTCTATCAATTTACCTCTCCACTTCCACAATCACTATCATTATTATTTATTTTATTAATTACCCTAGAGTTGGGTGTAGCCTTAATTCAAGCCTATGTATTTACCCTCTTAGTAAGCCTGTACCCATGATAATTCATAATGACTCACCAAACCCATACTTATCACATAGTCAACCCAAGCCCTTGACCACTAACAAGGAGCATTATCAGCTTTTCTTCTCACATCCGGCCTAGTTATATGATTCCACTTTTACCACACACTGCTACTCACTGCAGGTCTACTAGCTAGTTCTATAACAATATTTCAATGATGACGTGATGTAGTACGAGAAAGTACATATCCAAGGCCACCATACTGCACCTGTCCAAAAGGCCTACGATACGGAATAATTCTATTTTATTATTTCAGAAATTTTCTTCTTTGCAGGTTTCTTCTGAGCATTTTATCATTCTAGTCTAGCCCCAACTCCACAAACAGGGGACTATGACCACCTACAGGCATTACTCTAGCAATACGTAACC

>02728d49-0de3-421e-a32f-503d9f7d408b

AATGTACTTCGTTCAGTTACGTATTGCTCGAAGAGCCTTCAAGCCTAGTAAGTAAAGTACCCAGCAATTTTCTGCACAACAAATAAGACTGCAAAACTTTATTCTGCATCGGCTGAACTTAAATCAATATTTGTTATTTAAGCCCTTCTAGATTGATGGGATTTTAACCACAAAAATTTAGTTAACAGCTAAATAACCTAATCCAACTGGCTTCAATCTACTTCTCCGCCGTTAGGGAAAAAAGGCGGGAGAAGCCCCGGCAAGGTGAAGCTGCTTCTTTAATTTGCAATTCAATGTGATAGTTCACCTCAGGGCTGGTAAAAAGAGGTTCACTCCTCTGTCTTTAGATTTACAGTCTAATGCTTTTAACCAGCCATTTTACTATACCCTATGTTCATAAACCATGACTATTTTTCAACTAATCACAAAGACATTGGGACATTATATTTATTATTTGGCGCATGGGCGGGGGCGTGGTACGGTACAAGCCTCTAATTCGAACAACGGAACTAGGACAACCCGGAGAGCCTATTAGAAGACATTAAAACCATACAATGTTATTGTCACCGCCCACGCATTCATCATAATTTTTTTCATAGTAATACCAATTATGATTGGGGTTTTTGGGAACTGACTATTCCTTTAATAATTAGCGCTCTGATATAGCATTTCCTCGAATAAATAACATAAGCTTCTGACTACCACCCCACCTCCTACTCCTGCACTGGCATCATCAACTTTAGAAGCCGGTGCCGGCGGCACTGGTTGGACAGTCTACCCACTAATGGCGCGTTATCCCACCAGAGCCTCTGTAGATTTAACCATTTTCACTACATTTAGCAGGCGTTTTCTTCTATTCTTGGGCTATTAACTTGTCCACAGCAATTGTAAATATGGAAACCGCCAGCCATGACTCAATATCCGAGCTCCGTTGTTTTTGTGTGTGATCCGTCCTAATTACTGCAGTCCTTCTTTTCTCTTCCAGTTCTAGCTAATTACCATACTGTTAACTGACCATCGAAATTTGCATCACTACTTTCTTTGATCCTGCTGGTGGCGATAATCTATGCCAACACTTATTCTGATTCTTCGGTCACTTCGGAAGTATATATTCCTAGCTCTTCTAGGTTTTTGGAATAATTTCACACATTGTAACATATTATTCTAATAAAGAACCCGCACGGTTATATGGGCATGGTATGAGCCATAATATCTATTGGTTTCTATGAGCTTTATTGTATGAGCCCGTCACGCTATATTCACAGTAGGAATAGATGTAGATACTCGTGCATCAGCTACCGCAATCATTGCTATAGCAATACGTAACTT

>40826e3d-eba8-4a23-bc6b-d0caf5aab19d

TTGTGCTTTACGATTCGGTTACGTATTGCTATTTTTAACCCTGTAAAAAATTTAGTTAACAGCTAAATAACCTAATCAACTGGCTTCAATCTACTTCTCCCGCCGTTAGGGAAAAAAGGCGGGGCCCCGGCAAATTGAAGCTGCTTCTTCAATTTGCAATTCAATGTGATAGGTCCTACCTCAGGGCTGGTAAAAAAAGAGGGTTCACTCCTCTGTCTTTAGATTACCTTACAGTCTAATGCTTGCTCAGCCATTTTACCCCTACTATGTTCATAAACCGCTGACTATTTTCAACTAATCACAAAGACATTGCGGGACATTATATTTATTATTTGGCGCATGGGCGGGGGCAATGAGTACAGCCTAAGCCTCTAATTCGAACAGAACTAGGACAACCAGGAAGCCTAATAGAAGACGACCATGTATGCAATGTTATTGTCACCGCCCACGCATTCATCATAATTTTTTCATAGTATGCAATTTATGATTGGGGGTTTGGGAACTGACTTATTCCTTTAATAATTAGCGCTCCCGATATAGCATTCCTCCGAATAAATAACATAAGCTTCTGACTTCTACCACCCTCCTACTCCTACTGCTTTTGCATCATCAACTTTAGAAGCCGGTGCCGGCACTGGTTGGACAGTCTACCCACCCCTAGCAGGCAATTTATCCCACCCAGGAGCCTCTGTAGATTTAACCATTTTTTCACTACATTTTAGCAGGCATTTCTTCTATTCTTGGGCTATTAACTTTATTACAACAATTGTAAATATGAAACCGCCAGCCATGACTCAATATCAAACTCAAGTTGTTTGTGTGATCCGTCTAATTACTGCAGTCCTTCTTTTACTTTCTCTTCCAGTTCTAGCTGCGGGGATTACCATACTATTAACTGACCGTAATTTAAATACTACTTTCTTTGATCCTGCTGGTGGCGGCGACCCTATCCTATACCAACACTTATTCTGATTCTTCGGTCACCCTGAAGTATATATTCTAATCTTACCGGGTTTTGGAATAATTTCACACATTGTAACATATTATTCTAATAAAAAAGAACCCTTTGGTTATATGAATAGTGTGAGCCATAATATCTATTGGTTTCCTAGGCTTTTTATTGTATGAGCCCATCACATATTCTGATAGGAATAGATGTAGATACTCGTGCATATTTTACATCAGCTACCATAATCATTGCTATTCCCACTGGAAGTAAAAGTATTTAGCTGATTAGCCACACTGCACGGCGGTAATATCAAATGATTCTCCCGCAATATTATAGACCTGGGCTTTATTTTCTTTTTACCGTGGGTGGACTAACAGAATTTGTTCTTCTTAACTCATCATTAGATATTGTATTACATGATACATACTATGTAGTAGCCCACTTTCACTATGTGTTATCAATAGGAGCAGTATTTGCCATTATAGGGGGATTTATTCACTGATTCCCGCTCTTTTCAGGCTATACTCTTGACCAAACATATGCTGCAAAATCCGCTTCACCATTATATTTGTCAGCGTAAATTTAGCTTTCTTCCCACAACACTTCCTTGGCTTATCCGGAATACCTCGACGATACTCAGACTATCCAGACGCATATACTACATGAAATATCGTCTCATCTATCAGTTCAATTTGAAATTCACTTACAGCAGTAGTCTGATGGTTTTTATAATTTGAGGAAACTTTCTCTTCAAAACGAAAAGTCTTAGCCATTGAGCAACTATCCACCAATCTAGAATGATTATACGGCTGCCCTCCTCCTTACCACACATTCGAAGAGGCAACTTACGTTAAATCCTAGACGAAAAAGGAAGGATTTGAACCCCCAAAAATTGGTTTCAAGCCAATCCACTATACCCTATGACTTTTTCAATAAGATATTAGTAAAATAATTACATAACTTTGTCAAGGGATTAAATTATAGACTCATCTATATATCTTAAAGCAATACGTAA

>5df3a1a3-f557-4e0a-809f-86bebe2c75c6

TTGTACTTCGTTCAGTTGTGTGCTGAAGCCCCGGCAGGAATTGAAGCTGCTTCTTCAATTTGCAATTCAATGTGATAGTTCACCTCAGGGCTGGTAAAAGAGGGTTCACTCCTCTGTCTTTAGATTTACAGTCTAATGCTTGCTCAGCCATTTTACCCCTACCTATGTTCATAAACCATGACTATTTTCAACTAATCACAAAGACATTGGGATATTTGGCGCACATGGGCGGGGGCGAATAGGGTACAGCCCAGCCTCCTAATTCGAACAGAACTAGGACAACCCGGGAAGCCTAATAAAGACTGACCATGTACAAATGTTATTGTCACCGCCCACGCATTCATCATAATTTTTCATAGTAATACCAATTATGATTGGGATTTTTGGGGAACTGACTTATTCCTTTAATAATTGGCGCTCCCGATATAGCATTTCCTCGAATAAATAACATAGCTTCTGTTCCACCCTCCCCTACTCCTACTGCTTGCATCATCAACTTTAGAAGCCGGTGCCGGCACTGGTTGGACAGTCTACCCACCCCTAGCAGGCAATTTATCTCACCCATCTGTGATTTAACCATTTTTTCACTACATTTGAGCATTTCTTCTATTCTTGGAGCTATTAACTTTACAACAATTGCCTTAAATATGAAACCGCCAGCCATAGGCAATACGTAACT

>e586f257-cae3-4700-9707-0ea64791c477

AGTATTACTTCGTTCAGTTACGTGTGCTATACCCCGAAAATGTTGGTTTAATCCTTCCCGTGCTAACATTAATCCTCTAGCCCACCTTATTATCTCCTTCACCATTCAACAGGGACCGTAATCACAATTTTAAGCTCACATTGATTCTAGCCTGAATAGGCTTAGAATTAAATATACTAGCCATCGTACCAATCCTTGCCAAAAGTACCAATCCCCGCTCCACAGGACTCGTCACCAAATATTTTTTAATTCAAGCAACAGCATCAATACTTCTATTAGTATCCATTTTTCCTTAACAATCTACTAACTCAACAATGAACAATCAATCCTCCTTATAACCAAATATTATCCACAATAATATTTATTGCTCTAACAATAAATAGGGATAACCCACTTCACTTCTGACTCCCAGAAACACCCAAGGAATCCCTCTAATCAGCTATAATTATTCTCACGTGACAAAAACTCGCCCCAATATCAATTCTCCTCCAAATTTTTCCGTCAACAAACCTAAACTTGATTCTAACAATCTCAGTTCTATCAATTATAATTGGCAATGAGGAGGACTCAACCAAACACAACTCCGCAAAATCCTAGCCTATTCTTCAATTACTCACATAGAATTGAATAATAGCAGTATTATATTACAGCCCTAATATTACTATATTAACTTTAATTATTTATATTTTCTAAATCTCTACATTAATAATCTTTTATTAACCTCAAATGTAACAACCCTATCCCCTATCACATACCTGAAACAAACTAGCATGAACAATACCCATTATTCCACTAATAATAATATCCCTAGGAGGTCTACCCCCACTAACAGGTTTTTCCCCCAAATGAGCTATTATACAAGAACTTATTAAAAATGATAACTTAATTATTCCCCTTATAATAGCTTTTACTAATAAATTTATATTTTTTATATACGTTTAATATATTATATCTCAATGACAATATTCCCAACATCAAATAACACAAAAATCAACTGGCAACTAAATTATATAAAGCCAATACCGTTTCTATCCCCACTTGTAGTGTCTTCTACCTGTCTCCTACCCCTAACTCCACTAATACTTATAACTTGAAAATTTAGGTTAATAAGACCAAGAGCCTTCAAAGCCCTTGATAAATAAATTTTACTTAATTTCCTGCACAACAAATAAGGACTGCAAAACTTTATTCTGCATCAACTGAACATAAATCAATTACTTTAATTAAAGCTAAGCCCTTCCCTAGATTGATGGGATTTTAACCCACAAAAATTTAGTTAACAACTGAAAATAACCTAATCAACTGGCTTCAATCACTTCTCCCGCCGTTAGGGAAAAAGGCGGGAGAAGCCCCGACACAATTGAAGCTGCTTCTTTAATTTGCAATTCAATGTGATGTTAGTTCACCTCAGGGCTGGTAAAAGAGGGTTCACTCTCTGTCTTTAGATTTACAAGTCTAATGCTTGCTCAGCCATTTTACCCCTATGTTCATAAACCGCTGACTATTTTCAACTAATCACAAAGACATTGGGACATTATATTTATTTGGCGCATGGGCGGGGCAGTAGGTACAGCCCTAAGCCTCCTAATTCAGGCACAGAACTAGGACAACCGGAAGCCTAATAGAAGACGACCATGTATACAATGTTATTGTCACCACCCACGCATTCATCGTAATTTTTTTCATAGTAATACCAATTATGATTGGGGGTTTTAAGGAACTGACTTATTCCTTTAATAATTGGCGCTCCCGATATAGCATTTCCTCGAATAAATAACATAAGCTTCTGACTTCTACCACCCTCCCTACTCCTACTGCTTGCATCATCAACTTTAGAAGCCGGTGCGGCACTGGTTGGACAGATCTACCCACCTAGCAGGCAATTTATCCCCACCCAGGAGCCTCTGTAGATTTAACCATTTTTTCACTACATTTAGCAGGCATTTCTTCTATTCTTGGAGCTATTAACTTTATTACAACAATTGTAAATGAATACCGCCAGCCATGACTCAATATCAAACTCCGTTGTTTGTGTGATCCGTCCTAATTGCAGTCCTTCTTTTACTTTCTCTTCCAGTTCTAGCTGCAGGGATTACCATACTATTAACTGACATGTTTAAATACTACTTTCTTTGATCCTGCTGGTGGCGGCGACCTATCCTATACCAACACTTATTCTGATTCTTCGGTCACCCTGAAGTATATATTCTAATCTTACCGGGTTTTGGAATAATTTCACACATTGTAACATATTATTCTAGCTAAAAGAACCCTTTGGTTATGGGCATGAGTATGAGCCATAATATCTATTGGTTTCCTAGGCTTTATTGTGAGCCCATCACACTCACAGTAGGAATGGTGTAGATACTCGTGCATATTTACATCAGCTACCATAATCATTGCTATTCCCACTGGAGTAAAAGTATTTAGCTGATTAGCCACACTGCACGGCGGTAATATCAAATGATCTCCACAATATTATGAGCCCTGGGCTTTATTTTCTTTTTACCGTGGGTGGACTAACAGGAATTGTGTTAGCTAACTCATCATTAGATATTGTATTATGATACATACTATGTAGCCCACTTTCACTATGTGTTATCAATAGAGGCAGTATTTGCCATTATATGAGGATTTATTCACTGATTCCCACTCTTTTCAGGCTATACTCTTGACCAAACATATGCCAAAATCCACTTCACCATTATATTTGTCAGCGTAAATTTAACTTTCTTCCCACAACACTTCCTTAGCTTATCCGGAATACCTCGACGATACTCAGACTATCCAGATACGCATATACTACATGAAATATCGTCTCATCTATCCGGTTCATTTATTCACTTACAGCAGTAGTCCTGATGGTTTTTATAATTTGGCTTTCTCTAACCAAAACGAAAAGTCTTAGCCATTGAGCAACTATCCACCAATCTAGAATGATTATACGGCTGCCCTCTCTTACCACACATTCGAAGAGGCAACTTACGTTAAATCCTAGACGAAAAAGGAAGGATTTGAACCCCCAAAATTGGTTTCAAGCCAATCCCATATACTATGACTTTTCAATAAGATATTAGTAAAATAATTACATAACTTTGTCAAGAGTTAAATTATAGACTAAATATCTATATATCTTAATAGCAACACCAGCTCAACTAGGTTTACAAAATGCCACATCACCTATCATAGAAGAACTTATTTGCTTTCCACGACCATGCACTCATAATTATTTTCTGATTGATTTCCTTAGTCTTATATATTATTTCCCTTATACTTACCACAAAACTCACTCATACTAGCACCATAAATGCTCAAGAGATCGAAATAATCTGAACTATATTCGCCACCACTGATTCTAATTACAATTGCCCTCCCATCACTGCGTATTCTATATATGACAGACGAATTTAATAAACCTTATTTAACCCTTAAAAGCAATTGGTCTGTGATACTGAACCTATGAATATTCCGACTATGAAGACTTAGTATTTGACTCTTACATTATCGCAACATATTTCCTTGAGCCAGGGAAATTTCGACTCCTCGAAGTTGATAACCGGACAAGCTTTACCTATAGAAGCGGATATTCGCATATTAATCTCATCACAAGACGTCTTACACTCTGAGCCGTACATCACTAGGCGTAAAAACAGATGCAATTCCTGGACATTTAAACCAAAGCCATACTAGCCTCTATACGACCAATTTCATTTTGTGGACAATGCTCGGAAATTTGCGGGTCCAATCACAGCTTTATACCTATTGTTCTAGAATTCATTTATTTCCAAGATTTCGAGAAGTATGAGCTTCATACTTATATATTGTATCACTGTAAAGCTAACTTCAGCATTAACCTTTTAAGTTAAAGATTGAAGAACAAACTCTCTATAGTGAATGCCTCAACTAAATATTTCACCATTAATAGTAATTATATCTATAATTGTTACCTTATTTTATATTATACAATTGAAAATACTGAACTTTACTTTCCATTATTACCCACTACCAAAATTGGTAGAAACACAAAAACTTATAAAACAACTTGAGAACTAAAATGAACCAAAATCTGTTACCTCATTCAATATTCCAACATAATACTAGGAGTACCACTTAGTATTTTTAATTATTACACTCCCCACTACATTAATTTTATCCTCCAAAAACTTATTCAACAATGACTACTTCAATTCAACAATGGCTAATTCAACTAACACTTAAACAAATAATATTAACCCACACCACTAAAAGGGCGAACCTGATCCTTATACTCCTAGCCCTAATTTCTTTTGTTACCTAAATAACATTCTCGGACCACCATATGCATTTACACCAACCGCAACTGTCAATAAATCTAGGCATAGCTATTCCTCTATGAGCAGCAACTGTACTAATAGGCCTCCGATTTAAAACAAAATCATCCCTCGCTCATTTTTTTACCACAAGGAACACCAATTCCACTAATCCCTATATTAATTATTATTGAAACAATTAGTCTATTCATTCAACCTGTAGCCTTAGCCGTACAGTTAACAGCCAATATTACAGCGAGTCACCTATTAATGCATTTACTTGGGGATACAACATTAACTCTTCTATCAATTTACCTCTCCACTTCCACAATCACTATCATTATTATTATTTTATTAATTACCCCTACGGTTAGATTAGCCTTAGTCAAGCCTATGTATTTACCCTCTTAGTAAGCCTGTACTTACATGATAATTCATAATGACTCACCAAACCCATGCTTATCACATAGTCAACCCAAGCCCTTGACCACTAACTAGGAGCATTATCAGCTTTTCTTCTCACATCCGGCCTAGTTATATGATTCCATTTTTACCACACACTGCTTCTACTGCAGGGTCTATAGCTAGTTCTATAACAATATTTGTGATGTATTGATGTAGTACGAGAAAGTACATATCAAGGCCACCATACTGCACCGCATCCAAAAAGGCCTACGATACGGAATAATTCTATTTATTATTTCAAATTTTCTTCTTTGCAGGTTTGCTTTGAGCATTTTATCATTCTGATCTAGCCCCAACTCCACAAACAGGGGGGACTATGACCACCTGAGCATTCCCCTCAACCCAATAGAGAAGTCCCTCTCTTAAATACAACCGTACTACTAGCATCAAGGAGTTACAATTACATGAGCATCACAGCCTCGCAGAAACTAACCGAAAAGAATCAGCAAGCACTACTCCTAACTTATTAGGGATCTACTTCACCTGCCTACAATTGTCAGAATATTCTGGGAAGCCCCATTTACTATCTCCGACGGGTATATGGATCCACATTTTTTATGGCTACAGGCTTTCATGGCCTTCACGTAATTATCGGAACCACTTTCCTCACCACCTGTTACTTTCGCCAACAATTATATCACTTCACATCTAGCCACCATTTCGGCTTCGAAACCTGCATGATATTGACATTTCGTAGATGTAGTATGACTTTTCCTCTATATTTCCAAATATTGATGAGGCTCTTACTCTCTTAGTATAAAAAGTATTATTGACTTCCAATCAACGGGCCTCAGAATGATTCGAGAGAGAGTATTATAAATTTAATTTTATTTGTGACTAACATTACTTTGGCCTTACTTCTTATCACAATTACATTTGACTTCCACAATTAAATATTTATACAGAAAAGCACAACCCTTACGAGTGCAGTTTGGATCCCACCTCCGCCCACTTACCATTCTCCATAAAATTTTCCTAATCGCCATCATTTCTCCTATTTGATCTGGAAATTGCCCTGCTTCTACCCCTACCATGGGCAACCCAAACCAAAATAATTTAACTCTAATACCAATACAAATATAATTTTTACCCTACTTGTTATTCGGCTTTAGGGTTAGCCTACGAGTGGTCCCAAAAGGATTAGATTGGGCTGAATTGGTATATAGTTTAATTAAAACAAATGATTTCGACTCATTAGATTATGAAAGCTCATATTTACCAAATATGCCTTTTATCTATATTAATGTAATACTAGCATATTTCATATCATTATTAGGGTTATTAATTTATCGATCTCACCTAATATCATCACTGCTATGTTTGAAGAAGCATAATAATGTCATTATTTATCATAATTACACTCACAACTTTCAATATGCTTCATATTAATGTATATAGTACACTGTTCTCCTAGTATTTGCCGCATGCGAAGCTGCAGTAGGCCTAGCCTTGTTAATTTTAGTCTCCAACCTATATGGCCTAGATTGTACAAAACCTAAACTTGCTCAATGTTAAATTTATTTTCCAACCATCATAATACTTCCCACCATATGACTTTCAAAAAATTATATAATATGAATCAACACAATAATCTGTAGTCTACTAATCAGTATATACGCCCTCATATTACTCCACACACATAAATCATGCAACCTATCACTGATTTTCCTCCAGATTCACTAACATCACCCGCTTCTTATATTAACAGCCTGACTTCTGCCACTAATAATTCTAGCAACACAACAGCATTTATATAATAACCCCACCCCACGAAAAAAACTATATATCTCAATATTAATTCTATTACAAATTTCACTTATTATAACTTTTTCAGCTACCGAACTAATTTTATTTATATTCTATTTGAAACTACCTAATCCCCACCCTAATTATTACCCGTTGAGGGTATCAACCAGAACATATGTCTGGTTCATCTTACCTCTATTAATTACCCTCCTATACTGTCT

>603f860e-e8e4-4ab7-afda-2b4e070eca3b

ACATACTTCGTTCAGTTACGTATTTATATCTACTAACTCAACAATGAACAATCAATCCTCCTTATAACCAAATATTATCACAATAATATTTATTACTCTGCAATAAAAATAGTAGCCCCACTTCACTTCTGACTCAGAAATTGCCCAAGGAATCCCTCTAATCCCAATATAATTATTCTCACGTGACAAAAACTCGCCCCAATATCAATTCTCCTCAAATTTTTCGTCAACAAACCTAAACTTGATTCTAACAATCTCCAGTTCTATCAATTATAATTGGCAGCTGTGAGGAGGACTCAAACACAACTCCGCAAAATCCTAGCCTATTCTTCAATTACTCCGCACCGAGGATGAATAATAGCAGTATTATATTACGACCTAATATTACTATATTAACTTTAATTATTTATATTTTCCTAACAATCTCTACATTAATAATCTTTTTTATTTAACCTCAAATGTAACAACCCTATCCCTATCATACCTGAAACAAACTAGCATGAACAATACCCATTATTCCACTAATAATAATATCCCTAGGAGGTCTACCCCACTAACAGGTTTTTCCCCCAAATGAGCTATTATACAAGAACTTATTAAAAATGATAACTTAATTATTCCCCTTATAATAATAGCTTTACTAACATTAATAAATTTATATTTTTTTATATGCGTTTAATATATTATATCTCAATGACAATATTCCCAACATCAAATAACACAAAATCAACTGGCAACCTAAATTATATAAAGCCAATACCGTTTCTATCCCCACTTGTAGTGTCTTCTACCTGTCTCCTACCCCCTAACTCCACTATAATACTTATAACTTAGAAATTTAGGTTAATAAGCCCAAGAGCCTTCAAAGCCCACAGTAAGTAAATTTTTACTTAATTTCGTGCACAACAAATAAGGACTGCAAAACTTTATTCTGCATCAACTGAACATAAAATCAATTACTTTTAATTAGCTAAGCCCTTCCTAGATTGATGGGATTTTAACCCACAAAAATTTAGTTAACAAGACTAAATAACCTAATCAACTGGCTTCAATCTTCTCCCGCCGTTAGGGAAAAGAAAGCAGGCCCGGCTAAGTGAAGCTGCTTCTTTGAATTTGCAATTCAATGTGATAGTTCCTCAGGGCTGGTAAAAGGTTTCCTCCTCTGTCTTTAGATTTACAGTCTAATGCTTGCTCAGCCATTTTTACCCCTACCTATGTTCATAAACCGCTGACTATTTCAGCTAATCCCACCATAAGGACATTATATTTATTTGGCGCATGGGCAGGGCAGTAGAGTACAGCCCTAAAGCCTCTAATTCGAACAGAGACATAGGACAACCCGGAAGCCTAATGAAGACGACCATGTATACAATGTTATTGTCACCGCCCACGCATTCATCATAATTTTTTTCATAGTAATACCAGTATGATTGGGGTTTGGGAACTGACTTATTCCTTTAATAATTGGCGCTCCCGATATAGCATTTCCTCGAATAAATAACATAAGCTTCTGACTTCTACCACCCTCCCTACTCTACTGCTTGCATCATCAACTTTCAGAAGCCGGTGCCGGCACTGGTTTGACAGTCTACCACCCTACAGCAGGCAATTTATCCCACCCAGGAGCCTCTGTAGATTTAACCATTTTTTCACTACATTTAGCAGGCATTTCTTCTATTCTTGGAGCTATTAGCTTTATTACAACAATTGTAAATATGAAACCGCCAGCCATGACTCAATATCAAACTCCGTTGTTTGTGTGATCCGTCCTAATTACTGCAGTCCTTCTTTTACTTTCTTCCAGTTCTAGCTGCAGGGATTACCATACTATTAACTGACCGTAATTTAAATACTACTGCAGTCCTGCTGGTGGCGGCGACCCTATCCTATACCAACACTTATTCTGATTCTTCGGTCACCCTGAAGTATATATTCTAATCTTACCGGGTTTTGGAATAATTTCACACACATTGTAACATATTATTCTAATAAAAAAGAACCCTTTGGTTATATGGGCATGGTATGAGCCATAATATCTATTGGTTCCTGGAAGCTGTTGTATGAGCCCATCACATATTCTGATAGGAATAGATGTAGATACTCGTGCATATTTACATCAGCTACCATAATCATTGCTATTCCACTGGGTAAAAAAGTATTTAGCTGATTAGCCACACTGCGGCAGTAATATCAAGTGATCTCCCGCAATATTGAGCCCTATGGGCTTTATTTTCTTTTACCGTGGGTGGACTAACAGGAATTGTGTTAGTAACTCATCATTAGATATTGTATTACACATGATACATACTATGTAGTAGCCCACTTTCACTATGTGTTTATCAATAGGAGCAGTATTTGCCATTATAGGGGGATTTATTCACTGATTCCCGCTCTTTTCAGGCTATACTCTTGACCAAACATATGCCAAAATCCACTTCACCATTATATTTATCGGCGTAAATTTAACTGCTTCCCACAACACTTCAGCAACTCGGAATACCTCGACGATACTCAGACTATCCAGACGCATATACCCACTTGAAATATCGTCTCATCTATCGGTTCATTTATTTCACTTACGTAGTGGTCCTGATGGTTTTTATAATTTGGCTTCTCTTCAAAACGAAAGAATCTTGTATACATTGAGCAACTATCCACGTCTAAGATAAATTATACGGCTGCCCTCCTCCTTACCACACATTCGAAGAAACTTACGTTAAATCCCTGAACCCAAAAAGGAAGGATTTGAACCTTGAAATTGGTTTCAAGCCAATCCCATATACCCTATGACTTTTTCAATAAGATATTATTAAAATAATTACATAACTGTCCAAAGTTAAATTATAGACTAAATATCTATATATATCTTAATAGCAACACCAGCTCAACTAGGTTTACAAAATGCCACATCACCTATCATAGAAGAACTTATTGCTTTCCCGACCATGCACTCATAATTATTTTCCTGATTAGTTCTTAGTCTTATATATTATTTCCCTTACCTTTACCACAAAACTCGCTCTTTGCATATAAATGCTCAAGAGATCGAAATAATCTGAACTATTCTCCCTGCACTGATTCGATGATTGCCCTCCCATCCTGCGTATTCTATATGACAGACGAATTTAATAAACCTTATTTAACCCTTAAAGCAATTGGTCACCAATGATACTAGACCTATGAATATTCCAGGCTATGAAGGCTTAGTATTTGACTCTTACATTGTATGTAACATATTTCCTTGGCAGAATTTCGACTCCTCGAAGTTGATAACCGAACAACTTTACCTATATAGAAGCGGATATTCGCATATTAATCTCACAAGACGTCTTACACTCATGAGCCACGTACCATCACTAGGCGTAAAAACGAATTAAATTCCTGGACGTTTAAACCAAGCCATACTAGCCTCTATATACGACCAGGCCTATTTTATGGACAATGCTCGGAAATTTGCGGGTCCAATCACAGCTTTATACCTATTGTTCTAGAATTCATTTATTTCAAGATTTCGATGAGCTTCATACTTATATATTGTATCACTGTAAGCTAACTTAGCATTAACCTTTTAAGTTAAAGATTGAGAGAACAAACTCTCTATGATGAATGCCTCAGCTAAATATTTCACCGTGACCAATAATAATACTGTAATTATATCTATAATTGTTATTTTTTGTATTTTATATTATACTCACAATTGAAAATACTGAACTTTACTTTCTATTACCACTACCAATTAGTAGAAACACAAAAACATAAAACAACTTGAGAACTAAAATGAACCAAAATCTGTTTGCCTCATTCAATATTCCCAGCAATAGGAGTACCCTTAGTATTTTTAATTGTTGCCTCCCACTACATTAATTTTATCCTCCAAAAACTTGTTCAACAACCGACTCTCTTCAATTCAACAATGGCTAATTCAACCCCTAACACTTAAGCAAATAATATTAACCCACCACTAAAGGGCGAACCTGTCGCATATACTCCCTACCAGCCCTAATTTCTTTTATTACCTAAATAACATTCTCGGACTTACCATATGCATTTACACCAACCACCCAACTGTCAATAAATCTAGGCTGGCTATTCCTCTATGAGCAACAACTATTACTAATGACCTCCGATTTAAAACAAAATCATCCCTCGCTCATTTTTTACCACAAGGAACACCAGATTTTTTAATCCCTATATTAATTATTGAAACAATTAGTCTATTCATTCAACCTGTAGCCTTAGCCGTACGATTAACAGCCAATATTCTGACAGGTCTATTAATGCATTTTTCCAGGGATACAACATTAACTCTTCTATCATAAATTACCTCACTTCCACAATCATATCATTATTGATATTTTATTAATTACCCTAGAGTTGGGTGTATGAACCAAGCCTATGTATTTACCCTCTTAGTAAGCCTGTGTTACATGATAATTCATATTGTTACCAAACCCATGCTTATCATAGTCCAGCCCAAGCCTTGACTAACAGGAGCATTATCAGCTTTCTTCTCACATCCGGCTAGTTGTATGATTCCACTTTTACCCTGCTTCTCACTGCAGGTCTACTGGCTAGTTCTATAACAATATTCCAATGATGACGTGATGTAGTACGAGAAGAGAAGTACTATCCAAGGCCACCATACTGCACCTGTCAAAGGCCTGCAATCACGGAATAATTCTATTATTATTTCAGAAATTTTCTTCTTTGCAGGTTTCTTCTGAGCATTTTATCATTCTAGTCTAGCCCCAACTCCACAAACAGGGGGGACTATGACCACCTACAGGCATTACTCCCTCAACCCAATAAGTCCCTCTCTTAAATACAACCGTACTACTAGCATCAGGAGTTTTACAGCAGGCACATCACAGCCTCATAAGCTAACCGAAAAAATCAGCAAGCACTACTCTAACCATTATATTAGGGATCTACTTCACCTGCCTGCCTACAATTATCAGAATATTCTGAAGCCCCATTTACCTTATCTCCGACAGGTATATGGATCCACATTTTTTATGGCTACAGGCTTTCATGGCCTTCACGTAATCGGAACCACTTTTCCTCACCACCTGTTACTTTCGCCAACAATTATATCACTTCACATCTAGCCGCCATTTCGGCTTCGAAGCCGCTGCATGATATTGACATTTCGTAGATGTAGTATGACTTTTCCTCTATATTTCATCTATTGATGAGGCTCTTACTCTCTTAGTATAAAAAGTATTATTGACTTCCAATCAACGGGCCTCGAATGATTCGAGAGAGAAGTATTATAAATTTTTTGCCCCTAATGACTAACATTACTTTGGCCTTACTTCTTATCACAATTGTTTTTGGCTTTACAATTAAATATTTATACAGAAAAGCACAACCCACCGAGTGCGGATTTGATCCTGCTCCGCCCACTTACCATTCTCCATAAAATTTTTCCTAATCATAATCACATTTTCTCCTATTTGATCTGGAAATTGCCCACTATACCCCTACCAGCAATACGTAAC

>6fa89c90-1003-41ba-ab56-3fb8c133ae66

AATGTACTTCGTTCAATTGTGTGCTTAATCAACTGGCTTCAATCTGCTTCTCCCGCCGTTAGGGGAAAAAGGCGGGAGAAGCCTAAGCAAGGAGTGAAGCTGCTTCTTTCTGAATTTGCAATTCAATGTGATAGTTCACCTCAGGGCTGGTAAAAAGAGGTTCACTCCTCTGTCTTTAGATTTACAGTCTAATGCTTGCTCAGCCATTTACCCCTACCTATGTTCATAAACCGCTGACTATTTTCAACTAATCACAAAGACATTGGGACATTATCTTTTGTGCTTGTGGGCGGGGGCAGTAGGTACGGCTAAAGCCTCCTAATTCGAACAGAACTGGACAACCCCGGAAGCCTAATAGAAGACGACCATGTATACAATGTTATTATCACCGCCCACACATTCATCATAATTTTCATAGTATTACCAATTATGATTAGGGGGTTTTGGGAACTGACTTATTCCTTTAATAATTGAAAACTTGATATAGCATTTCCTCGAATAAATAACATAAGCTTCTGACTTCTAAGCTTAATACGTAATAA

>3ea33658-cbf0-4b8a-b937-66a3b0c727c3

AATGTACTTCGTTCAGTTACGTATTACTTATGTTCATAAACCATGACTAATACCAACTAATCACAAAGACATTGGGACATTATATTTATTATTTGGCGCATAGGCGAGGCAATAAGTACAGCCTAAACCTCTAATTCGAACAGAACTGAGACGACCGGAAGCCTAATAGAAGACGACCATGTATACAATGTTATTGTCACCGCCCACGTTCATCATAATTTTTTTCATAGTAATACAATTATGATTGGGGGTTTGGGAACTGACTTATTCCTTTAATAATTGGCGCTCCCGATATAGCATTTCCTCGAATAAATAAGCAATAGTAACA

>0bfefc8b-28bd-4f00-b63e-d72da0b66fe7

TTGTACTTCGTTCAGTTACGTATTACTTCAACTGTAGCTAAATTATATAAAGCCAATACCGTTTCTATCCCCACTTGTAGTGTCTTCTCTCTTCATCTATACTAACTCCACTAATACTTATAACTTGAAATTTTGGATTTAATAAGACAAGAGCCTTTAAAGCCCTTAGTAATCAAATTTACTTAATTTCTACACAACAAATAAGGACTGCAAAACTTTATTCTCACTGCATCAACTGAACGCAAATCAATTACTTTAATTAAATGTCCTTCTAGATTGATGATTTGCTTCACAAAAATTTGGTTAACAGCTAAATAACCTAATCATGGCTACAGTAGCACTTCTCCCGCCGTTAGGGAAAAAAGGCGAAGGACCCCGGCAAAATACGAGCTTCTTCTTTGAATTTGCAATTCAATGTGATAGTTCACCTCAGGGCTGGTAAAGGAGTTCCTCCTCTGTCTTTAGATTTACAGTCTAATGCTTGCTCAATAATTTTACCCCTACCTATGTTCATAAACCGCTGACTATTTTCAACTAATCACAAAGACATTGGAATATTATATTTATTATTTGGCGCATGGGCGGGGGCAGTAGGTACAGCCTAAGCCTCCTAATTCGAACAGAACTAGGACAACCCGGAAGCCTAATGAAGACGACCATGTATACAATGTTATTGTCACCGCCCACGCATTCATCATAATTTTTTTCTTTGTAGTAATACAGTATGATTGGGGGTTTTCGAGGAACTGACTTATTCCTTTAATAATTGGCGCTCCCGATATAGCATTTCCTCGAATAAATAACATAAACTTCTGACTTCCACCACCCTCCCTACTCCCTGCTGCTTCATCATCAACCTGGAAAGCCCGGTGCCGGCACTGGTTGGACAGTCCTACCCACTAGCGAGCAATTCGCCCACCAGGAGCCTCTGTAGATTTGCTCCATTTTTCACTACATTTAGCAGGCATTTCTTCTATTCTTGGAGCTATTAACTTCAGCAATACGTAA

>37f6b5c1-76a4-4ab7-8bb0-7d113872215c

AGTATGCTTCGTTCCAGTTACGTATTGCTCTAGCTTCAATCTACTTCTCCCGCCGTTAGGGAAAAAGGCGGGAGAAGCCGGCAGAATTGAAGCTGCTTCTTGCTGGTGCAATTCAATGCATGATAGTTCACCTCAGGGCTGGTAAAAAGAGGTTCACTCCTCTGTCTTTAGATTTACAGTCTCAATGCTTGCTCAGCCACATTTACCCTACTATGTTCATAAACCGCTGACTATTTCAACTAATCACAAAGACATTGGGACATTATATTTATTATTTGGCGCATGGGCGGGGCAATGGGCAGCCTAAGCCTCCTAATTCAATTAGAACAGGACAACCCGGAAGCCTAATAGAAGACGACCATGTATACAATGTTATTGTCACCGCCCACGCATTCATCATAATTTTTTCATAGTAATACCAATTATGATTGGGGGGTTTAGGAACAACTTATTCCTTTAATAATTGGCGCTCCCGATATAGCATTTCCTCGAATAAATAACATAGCTTCTGACTTCTACACCTCCTACTCCTACTGCTTGCATCATCAACTTTAAACCGGTGCCGGCACTGGTTGGACAGTCTACCCACCTAGCAGGCAATTTATCCACCAAAGGAGCCTCTGTAGATTTAACCATTTCCACTACATTTGTAGACATTTCTTCCCTATTCTTGGAGCTATTAACTTTATTACAACAATTGTAAATATGAAACCGCCAGCCATGACTCAATATCAAACTCCGTTGTTTGTGTGATCCGTCCTAATTACTGCAGTCCTTCTTTTACTTTCTCTTCCAGTTCTAGCTGCAGGGATTACCACTTTTTATTAACTGACCGTAATTTAAATCTCTTTCTTTGATCCTGCTGGTGGCGGCGACCCCCTATCCTATACCAACACTTATTCTGATTCTTCAGTCACCCTGAAGTATATATTCCTATCTTACCAGGTTTTGGAATAATTTCACACATTTGCAGCTTTCTTCTATTCTAATAAAAAGAACCCTTTGGTTATATGGGCATAGTATGAGCCATAATATCTATTGGTTTCCTAGGCTTTGTTGTGAGCCCATCACATTCACAGTAGGAATAGATGTAGATACTCGTGCATATTTTACATCAGCTATAATCATTGCTATTCCACTGGGTAAAGTATTTAGCTGATTGTTCCCCTGCACGGCAGTAATATCAAATGATCTCCACCTTATGAGCCTAGGCTTTATTTTCTTTTTTACCGTGGGTGGACTAACAGGAATTGTATTTAGCTAACTCATCATTAGATATTGTATTACATGATACATACTATGTAGTAGCCACACTTTCACTATGTGTTATCCAATAAAGACAGTATTTGCCATTATGGGGGATTTATTCGCTGATTCCGCTCTTTTCAGGCTATACTCTTGACCAAACATATGTAAAATCCACTTCACCATTATATTTGTCGGCGTAAATTTAACTTTCTTCCCCAACACTTCGTAGCTTGTCCGGAATACCTCGACGATACTCCAGACTATCCAGACGCATATACCCTTAATATCGTTATCATATCAGTTAAGCACGTAACA

>123ef4ef-272f-4f9f-aad9-7180a6e942cb

ATTGTACTTCGTTCAGTTACGTATTGCATTCTTCGGTACTCACATAGGATGAATAATAGCAGTATTATGTCCCGACCCTAATATTACTATAGTAACTTTAATTATTTATATTTTCCTAACAATCTCTACATTAATAATCTTTTATTTAATATAAATGTAACAACCCTATCCCTATCACATACACCTGAAGCAAACTAGCATAGACAATACCCATTATTCCACTAATAATAATATCCTAGGAGGTCTACCCCCACTAACAGGTTTTTCTGATGACTATTATACAAGAACTTATTAAAAATGATAACTTAATTATTCCCACCCTTAATAGCTTACTAACATTAATAAATTTATATTTTTATATGCGTTTAATATATTATATCTCAATGACAATATTCCCAACATCAAATAACACAAAAATCAATAACCTTAAATTATATAAAGCCAATACCGTTTCTATCCCCGTTGTAGTGTCTTCTACCTGTCTCCACCCTTTAACTCCACTAATACCTTATAACTTAGAAATTTAGGTTAATAAGACAAGAGCCTTCAAAGCCTTGGTAAGTAAATTTTACTTAATTTCTGCACAACAAATAAGGACTGCAAACTTTATTCTGCATCAACTGAACGCAAATCAATTACTTTAATTAGCTAAGCCCTTCCTAGTTGTTGGGATTTTAACCCACAAAAATTTAGTTAACAGCTAAATAACCTAATCAGCAAGCGCAATCTACTTCTCCCGCCGTTAGGGAAAAAGGCGGGAGAAATACAGCTGATGAAGCTGCTTCTTTGATGCAATTCAATGTGATAGTTCACCTGGAGGCACAGTAAAAGAGGGTTCCTCTACTCTGTCTTTAGATTGGTCTAATGCTTGCTCAGCCATTTTTACCCCTACCTATGTTCATAAGCCTTTGACTATTTTCAACTAATCACAAAGACATTGGGACATTATATTTATTATTTGGCGCATGGGCGGGGAGCGGTAGTACAGCCTAAGCCTCTAATTCGAACAGAACTAGGACAACCCGGAAGCCTAATAGAAGACGACCATGTATACAATGTTATTGTCACCGCCCGCACATTCATCATAATTTTTTTCATAGTAATACCAATTATGATTGGGGGTTTGGGAACTGACTTATTCCTTTGCAATAATTGGCGCCGATATAGCATTTCCTCGAATAAATAACGCATAAGCTTCTGACTTCTACCACCCTCCCTACTCTACCTCTTGCATCATCAACTTTAAGAAGCCGGTGCCGGCACTGGTTGAACAGTCTACCCACCCCTAGCAGGCGAATTTATCCACCAAGACCTCTGTAGATTTAACCAATTTTTCACTACATTTAGCAGGCATTTCTTCTATTCTTGGAGCTATTAACTTTATTACAACAATTGTAAATATGAAACCGCCAGCCATGACTCAATATCAAACTCGTTGTTTGTGTGATCCGTCGTACTACAGTCCGCCTTTTACTTTCTCTTCCAGTTCTAGCTGCTGGGGATTACCATACCTATTAACTGACCGTAATTTTAAATACTACTTTCTTTGATCCTGCTGGTGGCGGCGACCCTATCCTATACCAACACTTATTCTGATTCTTCGGTCACCCTGAAGTATATATTCTAATCTTACCGGGTTTGAATAATTTCTTATTGTAACATATTAATAAAAAGAATACTTGATTATATGAGCATAGTATAGCCTTAATATCTATTAACCTAGGGCTTTATTATGAATATACCATAACTACGATGAGATAAAGATATAGATATCGTGCATATTTTATGTATAATCGTTGCTTTATTCCCTGAGTAATATTTAGTAGTTGCCTTTACCTGCCACGGTGATAAATAATAAAATGATCTCCCGCAATATTATGAGCCCTGGGCTTTATTTTCTTTTTACCGTGGGTGGACTAACAGGAATTGTGTTAGCTAACTCATCGTGGAATATTGTATTACATGATACATACTATGTAGTAACCACTTTCACTATGTGTTATCAATAGGAGCAGTATTTGCCATTATAGGGGGATTTATTCACTGATTCCCGCTCTTTTCAGGCTATACTCTTGACCAAACATATGCCAAAATCACTTCACCATTATGTTGTCGGCGTAAATTTAACTTTCTTCCCACAACACTTCCTTGGCTTATCCGGAATACCTCGACGATACTCCAGACTATCCAGACGCATATACTACATGAAATATCGTCTCATCTATCAGTTCATTTATTTCACTTACAGCAGTAGTCCTGATGGTTTTATAATTTGAGAAGCTTTCTCTTCAAAACGAAAAGTCTTGCTTCATTGAGCAACTATCCACCAATCTAGAATGATTATACGGCCTGCCCTCCTCCTTACCACACATTCGAAGAGGCAACTTACGTTAAATCCTAGACGAAAAGGAAGGATTTGAACCCCCAAAAATTGGTTTCAAGCCAATCCCATACACCCTATGACTTTTCAATAAGATATTAGTAAAATAATTACATAACTTTGTCAAAGTTAAATTATAGACTAAATATCTATATATCTTAATAGCAACACCAGCTCAACTAGGTTTACAAAATGCCACATCACCTATCATAGAAGAACTTATTGCTTTCCACGACCATGCACTCATAATTATTTTCCTGATTAGTAGTTCCTTAGTCTTATATATTATTTCCCTTATACTTACCACAAAACTCACTCATACTAGCACCATAAATGCTCAAGAGATCAAAATAATCTGAACTATTCTCCCTGCACTGATTCTAATTACAATTGCCCTCCCATCACTGCGTATTCTATGCTGACAGACGAATTTAATAAACCTTATTTAACCCTTAAAGCAAGCAATACGTAGCA

>d0774d20-56b5-4a55-b34f-5c0a07a146a5

TTGTACTTCGTTCAGTTACGTATTGCTGGAATCCCTCTAATCCCAGCTATAATTATTCTCGTAATAAAAAACTCGCCCCAATATCAATTCTCCTCCAAATTTTCCGTCAACAAACCTAAACTTGATTCTAACAATCTCAGTTCTATCAATTATAATTGGCAGCTGAGGAGGACTCAACCAAACACAACTCACATAAAATCCTAGCCTATTCTTCAATTACTCATAGGATATGAATAATAGCAGTATTATATTACGACCCCTAATATTACTATATTAACTTTAATTATTATATTTTCCTAACAAATCTCTACATTAATAATCTTTTATTTAACCTCCAAATGTAACAACTTCCTATCCCTATCACATACCTGAAACAAACTAGCATGAACAATACCCATTATTCCATAATAATATCCCTAGGAGGTCTACCCCCACTAACAGGTTTTTCCCCCAAGTAAGCACAATATACAAGAACTTATTAAAAATGATAACTTAATTGTTCCCTTATAATAGCTTTACTAACATTAATAAATTTATATTTTTTTATATACGTTGTATTATATCTCAATGACAATATTCCCAACATCAAATAACACAAAAATCAACTGGCAACTAGAATTATATATAAAGCCAATACCGTTTCTATCACTTGTAATGTCTTCTACCTGTCTCCTACCCCTAACTCACTAATACTTATAACTTAGAAATTTAGGTTAATAAGACCAAGAGCCTTCAAAGCCCTTGGTAAGTAAATTTTACGCCAATTTCTGCACAGCAACAAATAAGGACTGCAAAACTTTATTCTGCATCAACTGAACGCAAATCAGTACTTTAATTAAGCTAAGCCCTTCCTAGATTGATGGGATTTCCCAACCACAAAAATTTAGTTAACAGCTAAATAACCTAATCAACTGACTTCAATCTACTTCTCCCGCCGTTAGGGAAAAAGGCAGGAGAAGCCCCGTAGGGTGAAGCTGCTTCTTTAATTTGCAATTCAATGTGTTAGTTCACCTCAGGGCTGGTAAAAGAGGGTTCACTCCTCTGTCTTTAAATTTACAGTCTAATGCTTGCTCAACCATTTTTACCCTACCTATGTTCATAAACCATGACTATTTTCAACTAATCCACAAAGACATTGGGACATTATATTTATTATTTGGCGCATGGGCAGAGGCAGTAAATTGCAGTTTCAGCCTCTAATTCAGGTAAGAACTTCGGGAACAACCCGAGCCTAATAGAAGACGACCATGTATCAATGTTATTGTCACCGCCCACGCATTCATCATAATTTTTTTCATAGTAATACCAATTATGATTGGGGGTTTTTAAGACTGACTTATTCCTTTAATAATTGGCGCTCCCGATATAGCATTTCCTCGAATAAATAACATAAGCTTCTGACTACCACCCTCCCTACTCCTACTGCTTGCATCATCAACTTTAGAAGCCGGTGCCGGCACTGGTTGGACAGTCTACCCACCCCTAGCAGGCAATTTGTCCATAGGAGCCTCTGTGGAATTTAACCATTTTTTCATACCATTTTAGCAGGCATTTCTTCTATTCTTGGAGCTATTAACTTTATTACAACAATTGTAAGCCATGAAACCGCCAACCATGACTCAACGTCAAACTCCGTTGTTTGTGTGATCCGTCTAATTACTGCAGTCTTCTTTTACTTTCTCTTCCAGTTCTAGCTGCAGGGATTACCATACTATTAACTGACCGTAATTTAAATACTACTTTCTTTGATCCTGCCTGGTGGCGGCGACCCTATCCTATACCAACAATATTCTAAATTCTTCGGTCACCCTGAAGTATATATTCCTAATCTTACCGGGTTTTGGAATAATTTCACACATTTGTAACATATTATTCTAATAAAAAAGAACACTTGGTTATATGGGCATGGTATGAGCCATAATATCTATTGGTTCTAGGCTTTATTATGAGCCATCCACATATTCACAGTAGGAATAGATGTAGATACTCGTGCATATTTTACATCAGCCATAATCATTGCTATTCCCACTGGAGTAAAAATATTTAGCTGATTAGCCGCACTGGCACGGTAATATCAAATGATCTCCGCAATATTATGAGCCCTAAGGCTTTATTTTCTTTACCGTGGGTGGACTAACAGGAATTGTGTTAGCTAACTCATCATTAGATGTTGTATTGCATGATACATACTATGTAGTAGCCCACTTTCACTATGTGTTATCAATAGGAGCAATGTTGCCATTATGAGGATTTATTCACTGATTCCCGCTCTTTTCGAGCTATATATCTTGACCAAACATATGCCAAAATCCACTTCACCATTATATTTGTCGGCGTAAATTGCTCTTCACAACACTTCCTTGGCTTATCCGGAATACCTCGACGATACTCCAGACTATCCAGACATATACTACATGAAATATCGTCTACATCTGTCAGTTCATTTATTTCACTTACAGCAGTAGTCCTGATGGTTTTTATAATTTGAGAAGCTTTCTCTTCAAAACGAAAAGTCTTAGCCATTGGTGCTATCCACCAATCTAGAATGATTATACGGCTGCCCTCCTCCTTACCACACATTCGAAGAGGCAACTTACGTTAAATCCTAGACGAAAAGGGAAAGGATTTGAACCCCCAAAAATTGGTTTCAAGCCAAATCCCATATACCCTATGACTTTTTCAATAAGATATTAGTAAAATAATTACATAACTTTGTCAAAGTTAAATTATAGATAAATATCTATATATCTTAATAACAACACCAAGCTCAACTAGGTTTAAATGCCACATGCCTATCTTAGAAGAACTTATTGCTTTCCACCATGCCTCGCAATTATTTTCCTGATTAGTTCCTTAGTCTTATATATTATTTCCCTTATACTTACCACAAACTCACTCATACTAGCACCATAAATGCTCAAAAGAGATCAAAAATAATCTGAACTATTCTCCCCTGCACTGATTCTAATTACAATTGCCCTCCATCACTGCGTATTCTATATATGACAGACAGATTTAATAAACCTTATTTAACCCTTAAAACAATTGGTCTCGAAAGTACTGAACCTATGAATATTCCGACTATGAAGACTTAGTATTTCTGACTCTTACATTATGCCAACATATTTCCTTGAGCCAGGGGAATTTCGACTCCTCGAAGTTGATAACCGAACAACTTTACCTATAGAAGCGGATATTCACATATTAATCTCATCACAAGACGTCTTACACTCATGAGCCGTACCATCACTAGGCGTAAAAACAGATACAATTCTGGACGTTTTAAACCAAGCCGTATAGCCTCTATGCGACCAGGCCTATTTTATGGACAATGCTCGGAAATTTGCGGGTCCAATCACAGCTTTATACCTATTGTTCTAGAATTCATTTATTTCCAAGATTTCGAAGTATGAGCTTCATACTTATATATTATCACTGTAAAGCTAACTTAGCATTAACCTTTTAAGTTAAGATTGAGAGAACAAACTCTCTATAGTGAATGCCTCAACTAAATATTTCACCGTGACCAATAGTAATTATATCTATAATTATTTACCTTATTTTATATTATACAATTGAAAATACTGAACTTTACTTTCCATTATTACCCACTATAAAATTAGTAGAAACACAAAAACATAAAACAACTTGAGAACTAAAATGAACCAAAATCTATTTGCCTCATTCAATATTCCAACAATACTAGGAGTACCCTTAGTATTTTTAATTATTGCACTCCCACTACATTAATTTTATCCTCCAAAAACATTCAACAACCGACTCTCTTCAATTCAACAATGGCTAATTCAACTAACACTTAAACAAATAATATTAACCCACACCACTAAGGGCGAACCTGATCCCTTATACTCCTAGCCCTAATTTCTTTTGTTACCTAAATAACATTCTCGGACTTACACCATATGCATTTACACCAACCACCCAACTGTCCAATAATCTAAACCTTAGCTATTCCTCTATGAGCAACAGCTGTACTAATAGGCCTCCGATTTAAAACAAAATCATCCCTCATCATTTTTACCACCAAGGAACACCAATTCCACTAATCCCTATATTAGTATTATTGAAACAATTAGTCTATTCATTCAACCTGTAGCCTTAGCCGTACGATTAACAGCCAATATTACAGCGGGTCACCTATTAATGCATTTACTTGGGGATACAACTTTTATTAACTCTTCTATCAATTTACCTCCTCCACTTCCACAAATCACTATCATTATTATTATTTTATTAATTACCCTAGAGTTGGGTGTAGCCTTAATTCAAACTGTATTTACCCTCTTGTGCAGCCTGTACTTACATGATATTCATAATGACTCTCAAACCCATGCTTATCACATAGTCAACCCCAAGCCCTTGACCACTAACAGGAGCATTATCAGCTTTTCTTCTCACATCCGGCCTAGTTATATGATTCCACTTTTTTTACCACACACCTTTCTCACCAGGTCTACTAGCTAGTTCTATAACAATATTTCAATGATGACGTGATGTAGTACGAGAAAGTACATCAAGGCCACCATACTGCACCTGTCCAAAAAGGCCTACGATACGGAATAATTCTATTTATTATTTCAGAAATTTTCTTCTTTGCAGGTTTCTTCTGAGCATTTTATCATTCTAGTCTAGCCCCAACTCCACAAACAGGGGGACTATGACCACCTACAGGCATTACTCCCCTCAACCCCAATAGAAGTCCCTACCAATACAACCGTACTACTAGCATCAGGAGTTACAATTACATGAGCACATCACAGCCTCATAAGCTAACCGAAAATCAACCCAAGCACTACTCCTAACCATTATATTAGGGATCTACTTCACCTGCCTACAATTATCAGAATATTCTGAAGCCCCATTTTACTATACTCCGACGGAGTATAACGGATCATTTTTTGTGGCTACAGGCTTTCATGGCCTTCACGTAATTATCGGAACCCTTTCCTCACCACCTGTTACTTTCGCCAACAATTATATCACCTTCACATCTAGCCACCATTTCGGCTTCTGACCGCTACCATGATATTGACATTTCGTAGATGTAGTATGACTTTTCCTCTATATTTCCATCTATTGATGAGCTCTTACTCTCTTAGTATAAAAAGTATTATTGACTTCCAATCAACGGGCCTCGAATGATTCGAGAAACGTATAAATTTAATTTTAGCCCTAATGACTAACATTACTTTGGCCTTACTTCTTATCACAATTACATTTTGACTTCCACAATTAAATATTTATGAAAAGCACAACCCTTACGAGTGCGGATTTGATCCTGCAACTCCGCCCACTTACCATTCTCCATAAAATTTTTCCTAATCGCCATCACATTTCTCCTATTTGATCTGGAAATTGCCCTACTTCTACCCCTACCATGGGCAACCCAAACAAATAATTTAACTCCCTAACAATAAATATAATTTTACCCTACTTATTATTCTGGCTTTAGGGTTAGCCTACGAGTGGTCCCAAAGTTAGATTGGGCTGAATTGGTATATAGTTTAATTAAAAACAAATGATTTCGACTCATTCAGATTATGAAAGCTCATATTTACCAAATATACCTTTTTATCTATATTAATGTAATACTAACATATTTCATATCATTATTAGGGTTATTAATTTATCGATCTCACCTAATATCATCACTGCTATGTTTGGAAGGCATAATATTATCATTATTTATCATAATTACACTCACAACTTTCAATATGCACTTCATATTAATGTATATGATACCCCTCATTCTCTAGTATTTGCCGCATGCGAAGCTGCGGTAGGCCTAGCCTTGTTAATTTTAGTCTCAACCTATATGGCCTAGATTATGTACAAAACCTAAACTTACTCCAATGTTAAATTTATTTTTAATATCCATAATACTTCCCACCATATGACTTTCAAAAAATTATATAATATGAATCAACACAATGATCTGTAGTCTACTAATCAGTACTTATACGCCCTCATATTACTCCACACACCAAACAACTCATATTACAACCTATCACTGATTTTTTCCTGGAATTCATAACATCACCACTTCACTTATATTAACAGCCTGACTTCTGCCACTAATAATTCTAGCAACAACAACATTTATATAATAACCCCACCCACGAAAAAACTATATATCTCAATATTAATTCTGTTACAAATTTCACTTATTATAACTTTTTCAGCTACCGAACTAATTTTATTTTATATTCTGTTTGAAACTACCTAATCCCCACCTAATTATTATTA

>be7091d3-8a8b-45a3-a8e0-3f9d974a78ed

ATTGTACTTCGTTCAGTTACGTATTGCTTTAATTTCTGCACAACAAATAAGGACTGCAAACTTTATTCTGCATCAACTGAACGCAAATCAATTACTTTAATTAAGCTAAGCCCTTCCTAGATTGATGGGATTTTAACCCACAAAAATTTAGTTAACAGCTAAATAACCTAATCAACTGGCTTCAATCTACTTCTCCCGCCGTTAGGGAAAAAGGCGGGAGAAGCTACGACAGGTGAAGCTGCTTCTTTTTGAATTTGCAATTCAATGTGATAGTTCACCTCAGGGCTGGTAAAAGAGGGTTCACTCCTCTGTCTTTAGATTTACAGTCTAATGCTTGCTCAGCCATTTTACCCCTACCTATGTTCATAAACCGCTGACTATTTTCAATAATCACAAAGACATTGGGACATTATATTTATTATTTGGCGCATGGGCGGGGGCAGTAAGGTACAGCCCTAAGCCTCCTAATTCGAACAGAACTAGGACAACCCGGAAGCCTAATGAAGACGACCATGTATACAATGTTATTGTCACCGCCCACGCATTCATCATAATTTTTTTCATAGTAATACCAATTATGATTGGGGGTTTTGAGAACTGACTTATTCCTTTAATAATTGGCGCTCCCGATATAGCATTTCCTCGAATAAATAACATAAGCTTCTGACTTCTACCACCACTCCCTACTCCTACTGCTTGCATCATCAACTTTAGAAGCCGGTGCCGGCACTGGTTGAACAGTCTTAACCCACCCCTAGCAGGCAATTTATCCCACCCAGGAGCCTCTGTAGATTTAACCATTTTTTCACTACATTTAGCAGGCATTTCTTCTATTCTTGGAGCTATTAACTTTATTACAACAATTGTAAATATGAAACCGCCAGCCATGACTCAATATCAAACTCCGTTGTTTGTGTGATCCGTCCTAATTACTGCAGTCTTCTTTCTCTTCCAGTTCTAGCTGCAGGGATTACCATATAATAACTGACCGTAATTTAAATACTACTTTCTTTGATCCTGCTGGTGGCGATTATCCTATACCAACACTTATTCTGATTCTTCGGTCACCCTGAAGTATATATTCTAATCTTACCGGGTTTTGGAATAATTTCACACATTTATCAATATATTATTCTAATAAAAAAAGAACCCTTTGGTTATATGGGCATGGTATGAGCCATAATATCTATTGGTTTTCCTAGGCTTTATTGTATGAGCCCATCACATATTCACAGTAGGAATAGATGTAGATACTCGTGCATATTTTACATCAGCTACCATAATCATTGCTATTCCCACTGGAGTAAAAGTATTTAGCTGATTAGCCACACTGCAGCGGTAATATCAAATGATCTCCCGCAATATTATGAGCCCTGGGCTTTATTTTTCTTTTTTACCGTGGGTGGACTAACAGGAATGGCAGCTAACTCATCATTAGATATTGTATTACATGATACATACTATGTAGTAGCCCACTTTCACTATGTGTTATCAATAGGAGCAGTATTTGCAGCAATACGTAACTA

>05ec6645-241c-49fe-8046-d20c10be5426

TTGTCTTTAAAGTTTCAGTTTACGTATTGCTATATCTCAATGACAATATTCCCAACATCCAAATAACACAAAAATCAACTGGCAATAAATTATATAAAGCTAATACCGTTTCTATCCTTGTAGTGTCTTCTGCATACTCTACCCCTAACTCCACTAATACCCATAACTTAGAATTTGATTTAATAAGACCAAGAGCCTTCAAAGCCCTTAGTAGTAAATTTTACTTAATTTCTGCACAACAAATAAGGACTGCAAACTTTTATTCTGCATCAACTGAACGCAAATCAATTACTGTTAAGCTAAGCCCTTCCCTAGATTGATGGGATTTTAACCCACAAAAATTTAGTTAACAGCTAAATAACCTAATCAACTAGCTTCAATCTACTTCTCCCGCCGTTAGGGAAAAAAGGCGGGGCCCGGCAGGAATTGAAGCTGCTTCTTTGAATTTGCAATTCAATGTGATAGTTCACCTCAGGGCTGGTAAAAAAGAGGTTCACTCCCTCTGTCTTTAGATTTACAGTCTAATGCTTGCTCAGCCATTTACCCCTACCTATGTTCATAAACCGCTGACTATTTTCAACTAATCACCAAAGACATTGGGACATTATATTTATTATTGGCGCATGGGCGGGGGCGGTAAGGTACAGCCCTAAGCCTCTAATTCAGCGAACAGAACTAGGACAACCCGGAAGCCTAATAGAAGACGACCATGTATACAATACTGACGTCACCGCCCACGCATTCATCATAATTTTTCATAGTAATACCAATTATGATTGGGGTTTGGGAACTGACTTATTCCTTTAATAATTGGCGCTCCCGATATAGCATTTCCTCGAATAAATAACATAAGCTTCTGACTTCTACCACCCTCCCTACTCCTACCAACACATCGCATCAACTTTAGAAGCCGGTATGGCACTGGTTGGTTTTGATCTACCCACCCTAGCAGGCAATTCATCACAGGAGCCTCTGTAGATTTAACCATTTTTTCACTACATTTAGCAGGCATTTCTTCTATTCTTGGAGCTATTAACTTTATTACAACAATTGTAAATATGAAACCGCCAGCCATGACTCAATATCAAACTCCGTTGTTTGTGTGATCCGTCCTAATTACTGCAGTCCTTCTTTTACTTTCTCTTCCAGTTCTAGCTGCAGGGATTACCATACTATTAACTGACCGTAATTTAAATACTACTGGCACAGTCTGCTGGTGGCGGCGACCCCATCACTGTCACAACACTTATTCTGATTCTTCGGTCACCCTGAGTATATGTTCTAATCTTACCGGGTTTTGGAGCAATTTCACACATTGTAACACATATTATTCTAATAAAAAGGAACCCTTTGGTTATATGGGCATGGTATGAGCCATAATATCTATTGGTTTCCTAGGCTTTATTGTATGAGCCCTATTACATATTCACAGTAGGAATAGATGTAGATACTCGTGCATATTTTACATCAGCTACCATAATCATTGCTATTCCCACTGGAGTAAAGTATTTAGCTGATTAGCCACACTGCACGGCGGTAATATCAAATGATCTCCCGCAATATTATGAGCCCTGGGCTTTATTTTTCTTTTTACCGTGGGTGGACTAACAGGAATTGTGTTAGCTAACTCATCATTAGATATTGTATTACATGATACATACTATGTAGTAGCCCACTTTCACTATGTGTTATCAGCCAGGACTTAGTATTTGCCATTATAGGGGGATTTATTCACTGATTCCCGCTCTTTTCAGGCTATACTCTTGACCAAACATATGCCAAAATCCACTTCACCATTATATTTGTCGGCGTAAATTTAACTGCTTCCCACAACACTTCCTTGGCTAATATCCGGAATACCTCGACGATACTCAGACTATCCAGACGCATATACTACATGAAATATCGTCTCATCTATCAGTTCATTTATTTCACTTACAGCAGTAGTCCTGATGGTTTTTATAATTTGAGAAGCTTTCTCTTCGAAGCAGAGTGTAGCCATTGAGCAACTATCCACCAATCTAGAATGATTATACGGCTGCCCTCCTCCTTACCACACATTCGAAGAGGCAACTTACGTTTAATCCTAGACAGGAAGGTTTGGACCCAAAAATTGGTTTCAAGCCAATCCATATACCCTATGACTTTTTCAATAAGATATTAAAATAATTACATAACTTTGTCAAAGTTAAATTATAGACTAAATATCTATATATCTTAATAGCAACACCAGCTCAACTGAGTTCATAAAATGCCACATCACCTATCATAGAAGAACTTATTACTTTCCTTTGACCATGCACTCATAATTATTTTCCTGATTAGTTCCTTAGTCTTATATATTATTTCCCTTATACTTACCACAAAACTCACTCATACTAGCACCATAAATGCTCAAGAGATCGAAATAATCTGAACTATTCTCCTGCACTGATTCTAATTACAATTGCCCTCCCATCTGCGTAATTCTATATATGACAGACGAATTTAATAAACCTTATTTAACCCTTAAAGCAATTGGTCGCCAATGATGAACCTATGAATATTCCGACTATGAAGACTTAGTATTTGACTCTTGTATTTATGCCAACATATTTCCTTGAGCCAGGGGAATTTCGACTCCTCGAAAGTTGATAACCGAACAACTTTACCTGGCAGAAGCGGATATTCGCATATTAATCTCATCACAAGACGTCTTACACTCATGAGCCGTACCATCACTAGGCGTAAAAACAGATGCAATTCCTGGACGTTTAAACCAAGCCAGCAATACGTAA

>1c33bb8b-c2ce-4667-be76-4ce3bb6e40ff

ATTGTACTTCGTTCAGTTACGTATTTGTAATAAACACAACTCCGCAAAATCTAGCCTATTCTTCAATTACTCACATAGGATGAATAATAGCAGTGATATATTACGACCCTAATATTACTATATTAACTTTAATTATTTATATTTTCCTAACAATCTCTACATTAATAATCTTTTATTTAACCTCAAAATGTAACAACCCTATCCCTATCACATACCTGAAACAAACTAGCATGAACAATACCCATTATTCCACTAATAATAATATCCCTAGGAGGTCTACCCCCACTAACAGGTTTTCCCCAAATGAGCTATTATACAAGAACTTATTAAAAATGATAACTTAATTATTCCCCTTATAATAGCTTTACTAACGATAATAAATTTATATTTTTTATATACGTTTAATATATTATATCTCAATGACAATATTCCCAACATCAAATAACACAAAAAAATCAACTGGCAACTAAATTATATAAAGCCAATACCGTTTCTATCCCCACTTGTAGTGTCTTCTACCTGTCTCCTACCCCTAACTCCACTAATACCTCAACTTAGAAATTTAGGTTAATAAGACCAAGAACCTTCAAAGCCCTTAGTAAGTAAATTTTACTTAATTTCTACAACAAATAAGGACTGCAAAACTTTATTCTGCATCAACTGAACGCAAATCAATTACTTTAATTAAGCTATTCCCTTCCTAGATTGATGGGATTTTAACCCACCAAAAATTTAGTTAACAGCTAAATAACCTAATCCAACTGGCTTCAATCTGCTTCTCCGCCGTTAGGGAGAAAAAAAGGCAGGAGAAGCCCCGGCAGGCAACAACAATTTGCAATTCAATGTGATAGTTCACCTCAGGGCTGGTAAAAAGAGGGTTCACTCCTCTGTCTTTAGATTTGATCTAATGCTTGCTAAGCCATTTTACTATATATGTTCATAAACCGCTGACTATTTTCAACTAATCACAAAGACATTGGGACATTATATTTATTATTTGGCGCATGGGCGGGGGCAGTAAGGTACAGCCCTAAGCCTCCTAATTCGAACAGAACTAGGACAACCCGGAAGCCTAATAGAAGACGACCATGTATGCAATGTTATTGTCACCGCCCACGCATTCATCATAATTTTTTCATAGTAATACCAATTATGATTGGGGGTTTGGGAACTGACTTATTCCTTTAATAATTGACGCTCCCGATATGGCATTCCTCGAATAAACAACATAAGCTTCTGACTTCTACCACCCACTCCCTACTCCTACTGCTTGCATCATCAACTTTAGAAGCCGGTGCCGGCACTGGTTGGTTGATCTACCCACCTAGCAGGCAATTTATCCCACCCAGGAGCCTCTGTAGATTTAACCATTTTTCACTACATTTAGCAGGCATTTCTTCTATTCTTGGAGCTATTAACTTTATTACAACAATTGTAAATATGGAAACCGCCAGCCATGACTCAATATCAAGAAACTCCCGTTGTTTGTGTGATCCGTCCTAATTGCAGTCCTTCTTTTACTTTCTTCCAGTTCTAGCTGCAGGGATTACCATACTATTAACTGACCGTAATTTAAATACTACTTTCTTTGATCCTGCTGGTGGCGGCGACCCTATCCTATACCAACACTTATTCTGATTCTTCGGTCACCCTGAAGTATATATTCTAATCTTACCAGGTTTTGGAATAATTTCACACATTGTAACATATTATTCTAATAAAAGACCCTTTGGTTATATGGGCATGGTATGAGCCATAATAATGTCTATTGGTTTCCTAGGCTTTATTGTATGAGCCCATCACATATTCACAGTAAAGATAGATGTAGATACTCGTGCATATTTTACATCAGCTACCATAATCATTACTATTCCCACTGGGTAAAGTATTTAATGATTAGCCACACTGCACGGCGGTAATATCAAATGATCTCCCGCAAGACCCTGGGCTTTATTTTTTCTTTGTGGTGGACTAACAGGAATTGTGTTAGCTAACTCATCATTAGATATTGTATTATGATACATACTATGTAGTAGCCCACTTTCACTATGTGTTATCAATAGGAGTAGTGTTGCCATTATAGGGTTATTCACTGATTCCCGCTCTTTTCAGGCTATACTCTTGACCAAACATATGTAAAATCCACTTCACCATTATATTTGTCGTAAATTTAACTTTCTTCCCACAACACTTCCTTGGCTTATCAGGAATACCTCGACGATACTCAGACTATCCAGACGCATATACTACATGAAATATCGTCTCATCTATCGGTTCATTTATTTCACTTACAGCAGTAGTCCTGATGGTTTTATAATTTGGCTTTCTCTTCAAAACGAAAAGTCATTGGTAGCTATCCACCAATCTAGAATGATTATGGCTGCCCTCCCTCCTTACCACACATTCGAAGAGGCAACTTACGTTAAATCCTAGACGAAAAAGGAAGGATTTGAACCCCCAAAAATTGGTTTTCAAGCCAGTCCCATAGCACCTATGACTTTTTCAATAAGATGTAGTAAAATAATTACATAACTTGTAAAGAGTTAAATTATAGACTAAATATCTATATCTTAATAGCAACACCAGCTCAACTAGGTTTGCAAAATGCCACATCACCTATCATGAAGAACTTATTGCTTTCCACGACCATGCACTCATAATTATTTTCCTGATTAGTTCCTTAGTCTTATATATTATTTCCCTTATACTTACCACAAAACTCACTCATACTAGCACCATAAATGCTCAAGAGATCCGAAATAATCTGAACTATTCTCCCTGCACTGATTCTAATTACAATTGCCCTCCCATCACTGCGTATTCTATATATGACAGACAGGTAATAAACCTTGTTTAACCCTTAAAGCAATTAGTCACCAATAATGATACTGAACCTATGAATATTCCGACTATGAACTTAGCAATACGTAACA

>fd968698-9096-4f19-bfe6-d7b0a0909ee4

AATGTACTTCGTTCAGTTACGTATTGCTCTCCACTAATACTTATAACTTAGAAATTTAGGTTAATAAGACCAGAGCCTTCAAAGCCACCAGTAAGTAAATTTTACTTAATTTCTGCACAACAAATAAGGACTGCAAAACTTTATTCTGCATCAACTGAACGCAAATCAATTACTTTAATTAAGCTAAGCCCTTCCTAGATTGATGGGATTTTAACCACAAAAATTTAGTTAACAGCTAAATAACCTAATCAACTGGCTTCAATCTACTTCTCCGCCGTTAGGAAAAAGGCGGAGAAGCCCCGGCTAAGGAGTGAAGCTGCTTCTTTGAATTGCAATTCAATGTGATAGTTCACCTCAGGGCTGGTAAAAAGAGGTTCTCCTCTGTCTTTAGATTTACAGTCTAATGCTTGCTCAGCCATTTTACCCCTACCTATGTTCATAAACCGCTGACTATTTTCAACTAATCACAAAGACATTGGATGTAATCGTGGTAGCTGGCATGGGCGGGGGCAGTAAGGTACAGCCCTAAGCCTCCTAATTCGAACAGAACTAGGACAACCCGGAAGCCTAATAGAAGACGACCATGTATACAATGTTATTGTCACCGCCCGCATTCATCATAATTTTTTTCATAGTAATACCAATTATGATTGGGGGTTTTGGGAACTGACTTATTCCCTTTAATAATTTAGCTCCCCGATATAACGCATTTCCTCGAATAAATAACATAAGCTTCTGACTTCTACCACCCTCCTACTCCTACTGCTTGCATCATCAACTTTAGAAGCCGGTGCCGGCACTGGTTGGACAGTCTACCCACCCCTAGCAGGCAATTTATCCCACCCAGGAGCCTCTGTAGATTTAACCATTTTTTCACTACATTTAGCAGGCATTTCTTCTATTCTTGGAGCTATTAACTTTATTACAACAATTGTAAATATGAAACCGCCAGCCATGACTCAATATCAAACTCCGTTGTTTGTGTGATCCGTCCTAATTACTGCAGTCCTTCTTTTACTTTCTCTTCCAGTTCTAGCTGCAGGGATTACATACTGTTAACTGACCGTAATTTAAATACTACTTTCTTTGATCCTGCTGGTGGCGGCGACCCTATCTATACCAACACTTATTCTGATTCTTCGGTCACCTGAAGTATATATTCTAATCTTACCGGGTTTTGGAATAATTTCACACATTGTAACATATTATTCTAATAAAAAAGAACCAGCTTGGTTATATGGGCATGGTATGAGCCATAATATCTATTGGTTTCCTAGGCTTTATTGTATGAGCCCATCACATACAGTAGGAATAGATGTAGATACTCGTGCATATTTTACATCAGCTACCATAATCATTGCTATTCCCACTGGAGTAAAAGTATTTAGCTGATTAGCCACACTGCACGGCGGTAATATCAAATGATCTCCCGCAATATTATGAGCCTAGGCTTTATTTTTTCTTTTTACCGTGGGTGGACTAACAGGAATTGTGTTAGCTAACTCATCATTAGATATTGTATTACATGATACATACTATGTAGTAGCCCACTTTCACTATGTGTTATCAATAGGAGCAGTATTTGCCATTATAGGGGGGATTTATTCACTGATTCCCGCTCTTTTCAGGCTATACTCTTGACAAACATATGCCAAAATCCACTTCACCATTATATTTGTCGGCGTAAATTTAACTTTCTTCCCACAACACTTCCTTGGCTTATCCGGAATACCTCGACGATACTCAGACTATCCAGACGCATATACTACATGAAGCGTCTCATCTATCGGTTCATTTATTTCACTTACAGCAATACGTAACTTCCG

>dd1ac151-3662-4d2e-a4c1-f79205c9d85e

TGTACTTCGTTCAGTTACGTATTGCTAACCTAATCAGCTGGCTTCAATCTACTTCTCCCGCCGTTAGGGAAAAAAGGCGGGGAGAAGCTAACAGGAGTGAGGAAAGCTGCTTCTTTGAATTTGCAATTCAATGTGATAGTTCACCTCAGGGCTGGTAAAAGAGGTTCACTCCTCTGTCTTTAGATTACAGTCTAATGCTTGCTCAGCCATTTTACCCCTACCTATGTTCATAAACCGCTGACTATTTTCAACTAATCACAAAGACATTGGGACATTATATTTATTATTTGGCGCATGGGCGGGGGCGAGTAGGTACAGCCTAAGCCTCCTAATTCGAACAGAATAGGACAACTTCCGGAAGCCTAATAGAAGACGACCATGTATACTAATGTTATTGTCACCGCCCACGCATTCATCATAATTTTCATAATGTGCCAATTATGATTGGGGGTTTGGGAACTGACTTATTCCTTTAATAATTGGCGCTCCCGATATAGCATTTCCTCGAATAAATAACATAAGCTTCTGACTTCTACCACCCTCCTACTCCTACTGCTTGCATCATCAACTTTAAGAAGCCCGGTGCCAGCACTGGTTGGACAGTCTACCCCACCTAGCGAGCAATTTATCCCACCCAGGAGCCTCTGTAGATTTAACCATTTTTTCACTACATTTAGCAGGCATTTCTTCTATTCTTGGAGCTATTAACTTTATTACAACAATTGTAAATATGAAACCACCAGCCATGACTCAATATCAAACTCCGTTGTTTGTGTGATCCGTCCTAATTACTGCAGTCCTTCTTTTACTTTCTCTTCCAGTTCTAGCTGCAGGGATTACCATACTATTAACTGACCGTAATTTAAATACTACTTTCTTTGATCCTGCTGGTGGCGGCGACCCTATCCTATACCAACACTTATTCTGATTCTTCGGTCACCCTGAAGTATATATTCTAATCTTACCGGGTTTTGGAATAATTTCACACATTGTAACATATTCTAAGCAATACGTAATGGCA

>635a4664-2c73-4ed5-a919-7baf60d9682a

ATACTTCGTTCAGTTACGTATTGCTCTAACTCCACTAATACTTATAACTTAGAAATTTAGGTTAATACAAGACCCGAAAAGGCCTTTAAAGACCCTTAGTAGAGTAAATTTTGCCAATTTCTGCTAACAAATAAGGACTGCAAAACTTTTATTCTGCATCAGATGAACGCAAATCAATTACTTTAATTAAGCTAAGCCCTTCCTACAAATTTGATGGGATTTTAACCTAAAAAATTTTAGTTAACAGCTAAATAGCCTAATCAACTGGCTTCAATCTACTTCTCCCATGCGTTAGGGAAAAAGGCGGAGAAGCCCCCACGCTGAAGCTGCTTCTTTGAATTTGCAATTCAATGTGATAGTTCACCTCAGGCTGGTAAAAGAGGGGATTCACTCATCTGTCTTTAGATTTACAGTCCTAATGCTTGCTCAGCCATTTTTACCCCTACCTATGTTCATAAACCGCTGACTATTTTCAACTAATCACAAAGACATTGGGACATTATATTTATTATTTGGCGCATGGGCGGGGGCATTAGGTACAGCCTAAGCCTCTAATTCGAACAGAACTAGGACGACCGGAAGCCTAATAGAAGACGACCATGTATACAATGTTGTGTCTGTACACATTCATCATGTGAGTCTTTTTCGCATAGTAAGCGCGAACATGATTGGGGTTTAGGAGACTTGACTTATTCCTTTAATAATTGGCGCTCCCGATACTTAATTTCCTCGAATAAATAACATAAGCTTCTGACTTCTACCACCCTCCCTACTCCTACTGCTTGCATCATCAACTTTAGAAGCCGGTGCCGGCACTGGTTGGACAGTCTACCCACCCCTAGCAGGCAATTTATCACTTGAGGAGCCTCGTGGGTTTAACCATTTTTTCGCTTTACATTTAGCAGGCATTTCTTCTATTCTTGAGCTATTAACTTTATTACAACAATTGTAAATATGAAACCGCCAGCCATGACTCAATATCAAACTCCGTTGTTTTGTGTGATCCGTCCTAATTGCAGTCCTTCTTTTTACTTTCTCTTCAGTTCTGCTGCAGGGATTACCATACTATTAACTGACCGTAATTTAAATGCTACTTTCTTTGATCCTGCTGGTGGCAGCGACCCTATCCTATACCAACACTTATTCTAGATTCTTCGGTCACCCTGAAGTATAGATATTCTAATCTTACCGGGTTTTGAATAATTTTTCACACATTGTCTTATATTATTCTAATAAAAAAGAATACCTTTGGTTATATAGGCATGGTATGAGCCATAATGTATTGGTTTCCTGAGGCTTTATTGTATACAGGCCATCACATATTCACGAGTAGGAATAGATGTAAGATACTCGTGCATATTTTATCAGCTACCATAGTATTTACTATTCCACTAGATAAAAAGTATTTAGCTTTGATTAGCCACACTGCACGGCGGTAGTATCAAATGGGAATCTCCGCTATTATGAGCCCTGGGCTTTATTTTCTTTTTACCGTGGGTGGACTAACGTGAATTGTATTCGCTAACTCATCATTAGATAGTAGTGACACATGATGTATGAATCGTAATA

>f7e18aa3-127a-4975-8d82-beb2745aec81

ATTGTACTTCGTTCAGTTACGTATTGCTGGTAAAAAGAGGGTTCATCTCTATCTTTAGATTTACAGTCTAATATGCTTGCTCAGCCATTTTACCTATGTTCATAAACCGCTGACTATTTTCAGCTAATCACAAAGACATTGGGACATTATATTTATTATTTGGCGCATGGGCGGGGGCAGTAGGTACAGCCCTAAGCCTCCTAATTCGAACAGAATTTGAGACAACCGGAAGCCTAATAGAAGACGACCATGTATACAGCTGTTGTCACCGCCCGCATTCATCATAATTTTTCATAATACCAATTATGATTGGGGGTTTTTTTTTGGGAACTGACTTATTCCTTTAATAATTGGCGCTCCCGATATAGCATTTCCTCGAATAAATAACATAAGCTACGGCTTCTACCCTCCCTACTCTCTGCTTGCATCATCAACTTTAGAAACCAGTGCCGGCACTGGTTGGACAGTCTACCCACCCCTAGCAGGCAATTTATCCTTACCCAGGGCCTCTGTAGATTTAACCATTTTTTCACTACATTTACTTGAGCATTTCTTCTATTCTTGGAGCTATTAACTTTATTACAACAATTGTAAATATGAAACCGCCAGCCATGACTCAATATCAAGCTCGTTGTTTGTGTGATCCGTCCTAATTACTGCAGTCCTTCTTTTACTTTCTCTTCCAGTTCTAGCTGCAGGGATTACCATACTGATAACTGACGTAATTTAAATACTACTTTCTTTGATCCTGCTGGTGGCGGCGACCCTATCCTATACCAACACTTATTCTGATTCTTCGGTCACCCTGAGTATATATTCTAATCTTACCGGGTTTTGGAATAATTTCACACATTGTAACATATTATTCTAATACAAAAAGAACCTTCAGTTATATGGGCATGGTATGAGCCATAATATCTGTGGTTCCTAGGCTTTATTGTATGAGCCCATCACATATTCACAGTAGGAACAGTGATGCTCGTGCATATTTTACATCGCTACATAATCATTGCTAATCACTGGAGTAAAAGTATTAGCTGGTG

>646bbb7d-24b6-4b44-826f-76cce2ecb4e7

AGTGTCACTTTAGTTCGGTTACGTATTGCTAAGGAAAAAAGGCGGGAGAAACTCCCGGCAGAATTAAACTGCTTCTTTGAATTTGCAATTCAATACGATGATTCACCTCCCAGGGCTGGTAAAAAGAGGGTTCACTCCTCTGTCTTTAGATTTACAGTCTAATGCTTGCTCAGCCATTTTACCCCTACCTATGTTCATAAACCACTTCGACTATTTTCAACTAATCACAAAGACGATGGGACATTATATTTATTATTTGGCGCATGGGCGGGGGCAGTAGGTACAGCCCTAAGCCTCTAATTCAAGTAGAACTAGGACAACCCGGAAGCCTAATAGAAGACGACCATGTATACAATGTTGACTATACCGCCATTAATCATAATTTTACTTCTAGTAATACCAATTATGATTGGGGGTTTTGGGAATTTGTTCTTATTCCTTTAATAATTGGCGCTCCGATATAGCATTTCCTCGAATAAATATATATAGCTTCGACTTCTACCTCTACTCCTACTGCTTGCATCATCAACTTTAGAAGCCGGTGCAACTGGTTGGACAGTCTACCCACCTAACTTGGCAATTTATCCCACCCAGGAGCCTCTGTAGATTTAACCATTTTTCACTACATTTATGAGCATTTCTTCTATTCTTGGAGCTATTAACTTTATTACAACAATTGTAAATATGAAACCGCCAGCCATGACTCAATATCAAACTCCGTTGTTTGTGTGATCAAATCTAATACTACCAGTCCTTCTTTTACTTTCTCTTCCAGTTCTAGCTACGAGGATTACCATACTATTAATGACCGTAATTAAATACTACTTTCTTTGATCCTGCTGGTGGCGGAGCTATCCTATACCAACACTTATTCTGATTCTTCGTTACCCTGAAGTATATATTCTAATCTTACCAGGTTTTGGAATAATTTCACACATTATGTGCTCTAATATTCTAATAAAAAAAGAAACCCTTTGAATTTATGAGCATGGTATGAGCCTTAATATCTATTAGTTTCCCTAGGCTTTATTGGCTTATGGTATCACATATTCACAGTAGGAATAGATGTAGATACTCTGTGTATTTGCATCATTTTACCATGTCATTGCTATTCCCACTGGAGTAAGTATTTAGCTGATTAGCCACTGCACGGTAATATCAAATGATCTCCCTGATAATATGAGCCTGGGCTTTATTTTCTTTTACCGTGGGTGGACTAACAGGAATTACAATTTAGCTAACTCATCATTAGATATTGTATTACATGATACATACTATGTAGTAGCCACTTTCACTATGTGTTATCAATAGGAGCAGTATTTGCCATTATAGGGGATTATTGCACGATTCCACTCTTTAAGGCTATCACTCTTGACCAAACATATGCCAAAATCCACTTCACCATTATATTTGTCGGCGTAAATTTAACTTTCTTCCCCAACACTTCCTTGGCTTATCCGGAATACCTCGACGATACTCAGACTATCAGACGCATATGCTACATGAAATATCGTCATCTATCAGTTTCGCATTTATTTCACTTACAGCAGTAGTCTGATGGTTTTTATAATTTGAGAAGCTTTCTCTTTTAAAGCAAAAGTCTTAGCCATTGAGCAACTATCCACCAATCTAGAATGATTATACGGCTGCCCTCTCCTCCACCCACCACATTCGAAGAACGTAGCTTACGTTAAATCCTAGACGAAAAAGGAAGGATTTGAACCCCCAAGTGGTTTCAAGCCGTCCATATACCCTAACAGAGCTTTTTCAATAAGATATTAGTAAAATAATTACATAACTTTGTCAAAGTTAAGTATAGACTAAATATCTATATATCTAATATAACACCAGCTCAACTAGGTTTTACAAAATGCCACATCACCTACTATCATAGAAGAACTTATTGCTTTCCACGACCATGCACTCATAATTATTTTCCTGATTAGTTCCTTAGTCTTATATATTATTTCCCTTATACTTACCACAAAAACTCACTCATACTAGCACCATAAATGCTCAAAGATCGAAATAATCTGAACTATTCTCCCTGCACTGATTCTAATTACAATTGCCCTCCCATCACTGCGTATTCTATATATGACAGACGAATTTAATAAACCTTATTTAACCCTTAAAGCAATTGTGGTCACCAATGATATAGCCTATGAATATTCCGACTATAACTGTTATTACTTCGGTTTCAGTTACCTATTATGTGCCCGGAATTGCGTCCGCAACGCTGTTCTGGCACTGGGCGAGGTTCGCTGGAAGTAAGATGAAGAAAATCTTGTTTGTTTGCCACGGCATTATATGATACATGCTGTGGGACCGATATGAATATCCGGGGCAGTTTGATTTGATGGAGATGGGTGCGATTTGATTCTCATACAGAATCGGTGTATGAAGCAAAATTTTACTCTATTATAATATACAATTTAGTTTATTTGAATCAAAGCAAAAAGCTCAAAAAGGAGAATTATTCATGGTTTTATTGGTTGTAGATGCACAGAATGGTATCGTGGATGAACGCTTATATGAGTTCAGAAAGTTTGTTGGTAATATCAAAAACTGGTGGAGCAGCTCGATAAAGGCATAGAAGTTATTTATGTACAGCATGATGATGGACCTGATACCGGATTCTCTATCGGGGATGATGAGTTTGAGGTTTATTCAAGGTTTCAGTAACCGATCTTACCTGATGAAAAACGGTTTATCAAGTCCGTATGCGAAGGTGCGATTCAAGAGTGGTCTGCTTGAGTATCTGACTGCAAAGGAAGAAAAAGACGTGACGATTTGTGGCATTGCCTTATTTGATTTCTGCTAATAAATGCCACGGTAGAGCTTGGTTTTGAGCATGGCTTGCACATGATTGTTCCAGCAATACGTAA

>f9cdc59b-4c4c-4fcc-b27e-88afdf26dc32

TTATGCTTTCGTTCAGTTACGTATTGCTAAGCCAATACCGTTTCATCCCCACTTGTAGTGTCAATACCTACCACCACCTAACTCCACTAATACTTATAACTTAGAATTTAGGTTAATAAGACCAAGAGCCTTCAAAGCCCTTAGTAAGTAAATTTTACTTAATTTCTGCAACAAATAAGGACTACTAGCTTTATTCTGCATCAACTGAACGCAAATCAATTACTTTAATTAAGCTAAGCCCTTCCTAGATTGATGGGATTTTAACCCACAAAAATTTAGTTAACAGCTAAATAACCTAATCAACTGGTAACTTCAATCTACTTCTCCGCCGTTAGGGAAAAAGGCGGGAGAAGCCCGGCAGAATTGAGAAGCTGCTTCTTTGAATTTGCAATTCAATGTGATGGATTCACCTCAGGGCTGGTAAAAGAGGGTTCACTCCTCTGTCTTTAGATTACGTCTAATGCTTGCTCAGCATTTTACCCCTACCTATGTTCATAAACCGCTGACTATTTTCAACTAATCACAAAGACATTGGAACATTATATTTATTATTTGGCGCATGGGCGGGGCGGTAGGTACAGCCCTAAGCCTCCTAATTCGAACAGAACTAGGACAACCCGGAAGCCTAATAGAAGACGACCATGTATACAATGTTATTGTCACCGCCCACGCATTCATCATAATTTTTTTCATAAATGTCTGAGTATGATTGGGGGTTTTGAGACAGCTTATTCGACTTTAATAATCAGCTCCCGATATAGCATTTCCTCGAATAAATAACATAAGCTTCCTGACTTCTACCACCCTCCTACTCCTACTGCTTGCATCATCAACTTTGACGGTGCCGGCACTGGTTGAACCAGTCTACCCACCCCTAGCAGGCAATTTATCCCACCCAGACCTCTAATAGATTTAACATTTTTTCCCACATGTAGGCATTTCTTCTATTCTTGAGGCCCCTATGCTTTATTACAACAATTGTAAATATGACCGCCAGCCATGACTCAATATCAAACTCCGTTGTTTGGTAGTGATCCGTCCTAATTACTGCAGTCCTTCTTTTACTTTCTTCCAGTTCCTTTGCAGGGATTACCATACTATTAACTGACCGTAATTTAAATACTACTTTCTTTAGGTCTGCTGGTGGCGGCGACCCCTATCCTATACCAACACTTATTCTGATTCTTCCAGTCACCCTGAAGTATATATTTACCGGGTTTTGGAATAATTTCACACATTGTAACATATTATTCTAATAAAAAAGAACCTTTGGTTATATGGGCATGGTATGAGCCATAATATCTATTGGTTTCCTAGGCTTTGTGTATGAGCCCATCACATATTCACAGTAGGAATAGATGTAGATACTCGTGCATATTTTATCAACACCATAATCATTGCTATTCCCACTGGAGTAAAAGTATTTAGCTGATTAGCCACACTGCACGGCGGTAATATCAAATGATCTCCCGCAATATTATGAGCCCTGGGCTTTATTTTTCTTTTACCGTGGGTGGACTAACAGGAATTGTGTTAGCTAACTCATCATTAGATATTGTATTACATGATACATACTATGTAGTAGCCCACTTTCACTATATTTATCAATAGGAGCAGTATTTGCCATTATAGGTTATTCACTGATTCCGCTCTTTTTCAGGCTATACTCTTGACCAAACATATGCCAAAATCCACTTCACCATTATATTTGTCAGCGTAAATTTAACTTTCTTCTAACCACTTCCTTGGCTTATCCGGAATACCTCGACGATACTCAGACTATCCAGACGCATATACTACATGAAATAATAATCTCATCTATCAGTTCATTTATTTCACTTACAGCAGTAGTCCTGATGGTTTTTATAATTTGAGAAGCTTTCTCTTCAAAACGAAAAGTCTTAGCCATTGAGCAACTATCCACCAATCTAGAATGATTGCTGACTGCCCTCCTCCTTACCATTCGAAGAGGCAACTTACGTTAAATCCTAGACGAAAAGGAAAGGATTTGAACCCCCAAAAATCGTTTCAAACCAATCCATATACCCTATGACTTTTTCAATAAGATATTAGTAAAAATAATTACATAACTTTGTCAAAGTTAAATTATAGATATAAATATCTATATATCTTAATAGCAACACCAGCTCAACTAGGTTTACAAAATGCCATACCTATCATAGAAGAACTTATTGCTAGCAATACGTAACCCG

>5fac2bf0-9a3d-4062-9430-556c0cfda263

ATGTGCTTCGTTCAGTTACGTATTGCTGATGGGATTTTTAACCCACAAAATTTAGTTAACAGCTAAATAACCTAATCAACTGGCTTCAATCTACTTCTCCCGCCGTTAGGGAAAAAAGGCGGAGAAGCCCCCGGCAAAGTGATAGCTATTTCTAATTTGCAGATCCAATGTGATGATCTCCCGGAGCTGGCTAAAAGGGTTCTCTGTCTTTGGAGTTACAGTCTGAAAATGGCATCAACCATTTTACCCCTACCTATGTTCATAAACCTTTGACATCTTTTCTTAACCAATCACAGGCATTGGGACATTATATTTATTATTTGGCACGTAGGCAAATTTGTAGTGGAATTTGCAACGAGCTAAACTTCTCTATTGATTCTCAATGGGGCTGATTCTTGGAAGCCTATGAAGACGACCGTATCTTCAATGTTTATTATCTGCGCCTACTGCGATCTCATCATGTTTTCTTAGTGTACCAAAATTCTATGATTGGGGTTCAGAACTATTATTCTTTAATAATTTGAAAACCCCATTAGCATTTCGAGGTAAATAACATAAACTGAAGCTCTACCGCCTCTCTTTCCCTACCTTTGCATCAACTTTAAAGCCATTTTGCATGATTGAACAGTCACTTCCACTGGCGAACGATTTATCCGGAGCCTCGTTAACGCATTTTCCTTTATTTGCTGAAACATTTCTTTATTCTTGGAGCTATTAACTTTATTACAATAATTGTAAATATAAACGCCAGCCATGACTCAATATCAAACTCCGTTGTTTGTGTGATCGTCTAATTACTGCAGTCTACTTCTTTACTTTCTCTTCGTTCTGCTACGAGATTATTCTAATAACGACATAATTTAAATACTACTTTCTTTGATCCTATATGGTGGCGAAAACGACCTATCTATACCAGCACTTGTCGATTCTTCGGTGCCTGATATATATTCTAATCTTACCGGGTTTTGGGAATAATTTCACACATTGTAACATATTATTCTAATAAAAAGAGAACCCTTTGGTTATGTAGACATGGTATAGTATAATATCTATTGATTTCCTAAGCTTTATTGTATGAGCCCATCACATATTCACCGTATAGACGATAAAATGCTCGTTACTGTGTCCCATCAGCTACCATAATCGTGTATTCTGGAGTAAAAGTATTTAGCTGATTAGCCCTTACGCACGGCGGTAATATCAAATGATCTCCGCAGCAATGTGAGCCTGAGCTTCATTTTCTTTTACCGTGGGTGGACTAGCAGGAATTATTATTACGGCTAACTCATCATTAGATATTGTATTACGCTGATACATACTATCATAGTATTGCTACTTTATATGTTATCAATAGGAAGGTAGTATTTGCGTATGGGGGGATTTATTCACTGATTCCCGCTCTTTTCAGGCGCTATACTCTTGATATCGTGCTGTACTAAAATCCACTTCACCATTATATTTGTCGGCGTAAATTTAACTTTTTGCTTCACAACACTTCCTTGGCTTATCCAGATACCTCGACGATACTCAGACTATCCAGACGCATATATACATATAAAATATCGTCTCTATCTATCAGTCTTAGCAATACGTAACGCA

>9ec8a821-f83c-4446-94e4-6141de2f7b33

GTTGTACTTCAGATAGTTACATTATTATCCCACTAACGGGTTTTCTACCCAAATGAGCTATTATACAAAACTTATTAAAAATGATAACTTAATTATTCCCCTTATAATAGCTTTACTAACATTAATAAATTTTCTTTATGTATTTAATATATTATATACTCAATGACAATATTCCTAATCAATAACACAAAAATCAACTTTTAATAAATTATATAAAGCCAATACCATTTCATCTCCCCACTTAGTATTATCTTCCTGTCTCCTACACCTAACTCCACTATTACTTATAACTTAGAAATTTAGGTTAATAAGACCAAAACCTTCAAAGCCCCTTAGTAAATAAATTTTACTTAATTTCTGCAACAAATAAGACCACCAAACTGTTTAACATCAATGAACGCAAATCAATTACTTTATTAATAAGCTGCTTGGTTGATGAGATTTTTTAAACACTGAAAATTTGATTTTAACAGCTAAATAACCTAATCAACTGGCACAATCTGCTTCTCCATAATTGGGAAAAAATGGCCCCAGGGAGAAGCCCCATAGGGTGGAGAAGCTGCTTCTTTGGATACACAATTCAATGTGATAGTTCACCTCAGGGCTGGTAAAAAGAGGGTTCACTCTCCTCATATATTTTTGAATTTACAGTCTATTCTTGCTCAGCCATTTACCCTACCTATGTTCATAAACCGCTGACTATTTTCAACTAATCACAAAGACATTTTCAGGACGACATTATATTTATTATTTGGCGCATGGGCGGGGGCAGTAGGTAGCCTAAGCCTCCTAATTCGAACAGAACTGGGGACAACCCGGAAGCCTAATAGAAGACGACCATGTATACAATGTTATTGTCTGCTTTATGAATATTCACAATTTTTTCATAAGTAATACCAATTATGATTTAACGGTTTTCGAGAACGACTTATTCCTTTAATAATTGGCGCTCCCGATACGGCCTCAATTAATATAAGCTTCTGACTTCACACTATCTGTCCCTACTTAGCATATATTAAGCTTTAAACCCGGTGCGGCACTGGTTGAACAGTCTACCCACCCTAGCAGGCAATTTATCCACCAGAACCTCTGTGATTTAACCATTTTTTTATTTTCCATTTAGCAGGCATTTCTTCTGTTCTTGTTCATCATATTATCAACAATTGGCTCTCGTGTGACTCAATATCAAACTCCGTTGTTTGTGTGATCCGTCTATAAATTACCAAGTCCTTCTTTTACTTTCTCTTCCAGTTCTAGCTGCAGGGATTACCATATATTAGCAGCCGTAATTCATCTGCTGCAGTCTACGGTAGCGGCATTATCCGCTACCAACATTATGATTCTTTGAGTGCTCGGAATGCATATTTAAATCACCAAGTAAAGATAATTTCACACATATACATATTATTCAACAAAGAACTATTTGAATTATAGACAGTATGAGCCATAATATGTGGTTTCCTAGGCTTTTATTGTGAGCCCATCACATATTCACAGTAGGAATAGATGTAGATGCTCGTGCATATTTTACATCATTACATAATCATTGCTATTCCCACTGGAGTAAAATATTGTTGATTAGCCACACTGCACGGCATTGTATCAGAATGACAATACGTAATAA

>2c3a5247-4534-4028-8a69-e4ede705874d

ATTACTTCGTTCAGTTACGTATTGCTAATAGCTTTTACTAACATTTAATAAATTTATATTTTTATATCGTTTAATATATTATATCAATGACAATATTTAACATCAAATAACACAAAAATCAACTGGCAACTAAATTATATAAAGCCAATACCGTTCTATCCCACTTGTAGTGTCTTCTACTGTCTCCTACCCCTAACTCCACTAATACTTATAACTTAGAAATTTAGGTTAATAAGACCAAAGAGCCTTCAAAGAGCCGCTAGTAAGTAAATTTTACTTAATTTCTGCACAACAAATAAGGACTGCAAACTTTATTCTGCATCAACTGAACGCAAATGTTCCGCTAATTAGCTAAGCCCTTCCTGATTGATGAAGTTTAACCCATTAGTTAACAGCTAAATAACTAAATCAATATTACCAATCTGCTTCTCCCGCCGTTAGAAAACAAAAGGCGGGAGCCCCAACGAAGCTGCTTCTTTGAATTTACAATTCAATGAATAAGTTCCCTCCATGAGGCTACTGGTAAAAAGAGGGTTCATCCTCTGTCTTTGAATTTACGAAGTCTAATGCTTGCTCAGCCATTTGCCCCTACCTATGTTCATAAACCGCCATTTATTTTCAGCTAATCACAAAGGCATTAGGACATTATCTTTATTATTTGGCGCATGGGCGGTGGTAGGTACAGCCCTAAGCCTCCTAATTTGAACAGAACGGAACAACCGGAAACCTAATAGAAGACGACCATGTATATTATTGTCACCCTTACATTCATCATAATTTTTTTCATAATAACCACCAATTGATTGGGGGTTTTGGGAACTGACTTATTCCTTTAATAATTGGCGCTCCCGATATAGCATTTCTCGAATATTAACACTAACAGCTTCTGACTTCTACCACCTCCTACTCCTACTAGCAACTGCATCATCAACTTTAGAAAGCTGATTGTACCTTTGATTTGGACGATCTACACCTAGCAGGCAGTTTAAACCACCAGAACCTCTGTAGATTTAACCATTTTTCTTTGTTTATAGGCATTCTTCTATTCTTGGAGCTATTAACTTTATTATATGTGTTGCCGTATGAAACCATAGCCGAAATATCAAACTCCGTTGTTGTCTTGATCGTCCTAATTGCACGGTCCTTCTTTTACTTTCTCTTCAGTTCTAGCTGCAGGGATTATATAATAACGACGTAATTTAAATACTACTTTCTTTGATCCTACAGGTGGCGTGACTATCTATACAACACTTATTTCGTTCTTCGGTCACCCTAGTATATATTCTAATCACCGGGTTTTGAATAATTTCTACTTTGTTAACAACATATTATTCTACAATAAAAAGAACCCTTTGGTTATATGGGCATAGTATGACCATAATATCTAATGGTTTCACCTATAGGCTTTATTGTGTTATCACATATTCACCATTAGGAATAGATGTAAATACTCGATATATGACCACACATCAGCTAAATCATTGCTATTCCCCTTTAGATAAAAATATTTAGCTGATTAATACGCAGCGGTAGCATATCAAATGATCCTCCTGTATGAGCCCTGGGCTTTATTTTTCTTTGCAGTAGGTGGACTAACAGGAACGTCCCATTTAGCTAACTCATCATTAGATATTCTTGCTGGCAGCGATCACATGCTATGGCCAACCCTTTCCTTATGTGTTATCAGCTAGAACATTATTTACTGCCATTATAGAGTTATTCACTGATTCCCGCTCTTTTCCAGGCTACTCTTGACCAAACATATGCTTTTACAAAATCCTTCACCATTATATTTGACCAGCGTAAATTCTTTCTTCCCTATGCTTCCTTGGCTTAAGCTCAGAATACTCGACGATACTCAAAACCATCCAGACGCATATACTACATGAAATATAGTCTCGTATCAGTTCATTTATTTCTCACCACACGATAAAACAGTCCTGATGGTTTTATAATTTAAAGCTTTCTTAAAACGAAAGTCTTATTGATGAGCAACTATCCACCAGTCTAGAATGATTACGGCTGCCCTCCTCAACACATTTGTCGAAGAGGCAACACGTTCGTCTAGACGAAGGAAGGATTTGAACCCCAAAGAATTGGTTTCAAGCCAATCCACCACTTTACGACAACAAATGGTATAAATGCACGTAAAAATTCATCACATTAATAATATTTACTATGCTAATAGCAACACCAATCCTTCAGAGGTTTACCATCACTATCATATTAACTTACGACCATATATAGCAATTATTTTCCACGATTGGTTCCTTAATCTTACCAATATTATTTTCCATAACACCGCAAGCTATCTTATTCTTTATAAATGTAAGAGATCAGCGAAATAATCTGAACTATTCTCCTGCTGATTCTAATTACAATTGCCCTCCCATCACTGCACAGGCAGCCGCGTAGCA

>17af00a9-2dbe-4304-9731-5643eab05b2e

TCGTTATTAAGCGTTCAGTTACATGCTCCTAACTCATAATACTTATAACTTAAAATTTGAGTTAATAGACCAAGAGCCTTAAAAACCACCAATGCGATAGAATTTCATAATTTCTGCACAACAAATAAGGACTGCAAAACTTTATTTACTGCCATCAACTGAACGCAAAATCAATTACTTTGAATTAAGCTGTTCCTGAATTGATGCAGTTTTAACCCACAAAATTTAGTTATGTGGCTAAATACTTCTAATCCAACTGGCCAGTCTACTTCTCCCGCCGTTAGGAAAAAGCGGGAGAAATACGACAATTAGGCTTTACTTCTTTGGGTGCAATTCAATGTGATAGTTCACCTCAGGGCTGGTAAAAAGAGGGTTCCTCCCTCATCTTTAGATTACAGTCTAATGCTTGCTCAACCATTTTGCCCTACCTATGTTCATAAACCGCTGACTATTTTCAACTAATCACAAAGACATTGGGACATTATGTTTATTGTTGGCGCATGGGCGGGGGCAATGAAGTACAGCCTAAGCCTCCCTAATTCGAACAGAACTAGGACAACCCGGAAGCCTATTAGAGGCGACCGTATATACAATGTTATTGTCACCGCCCACACCCTTCATCATAATTTTTTCATAGTAATACCAATTATGATTGGGGTTCTAAAGACTGACTTATTCGCTTCTAATAATTGGCGCTCCCGATATAGCATTTCTCGAATAAATAACGCAAGCTTCTGACTTCCACCGCCTCCCTACTCCTGCTTGCATCATCAACACAGAAGCCGGTGCCGGCACTGGTTGGACAGTCTACCACCTAGCGAGCAATTTATCGCAGGAGCTGGCAATACGTAGCA

>5830fa2e-2250-4842-9ea2-dfc3d12734f4

ATTCACTTCGTTCAGTTACGTATTGCTAATCCCAGCTATAATTATTCTCCGTGACAAAAACTCACTTCAATATCAATTCTCCTCCAAATTTCCGTCGGCAAATACCTAAACTTGATTCTAGCACGTCCTCAGTTCTCTTCTCGAAATTATAATTGGCAGCTGAGAGGACTCAACCAAACACAACTCCATAAAATCCTAGCCTATTATTCATTATACATAGTTGAATAATGGCAGTATTTATATTACGACCTATTACTATATTAACACTTTAATTATTTATATTTTCCTAACAATCTCTACATTAATAATCTTTTATTTAACCTCAAATGTAACAACCCTATCCCTATCATACCTGAAACAAACTAGCAGACAATACCCATTATTCCACTAATAATAATATCCCTAGGAGGTCTACCCCCACTAACAGGTTTTTCACACGAATGGGCTAACATACAAGAACTTATTTAAAAATGATAGCAATTATTCCACCATAATGGCACACTAGCATTAATAAAGTTATATTTTTATATACGTTTAATATATTGCCATCAATGACAATATTCCCAACATCAAATAACACAAAAATCAATGGCAACTAAATTATATAAGCCAATACCGTTTCTATCCCCACTTGTAATTAATCTTCTACCTGTCTCCTACCCTAACTCACTAATACTTATAACGAAAATTTAGGTTAATAAGACCAAGAGCCTTCAAAGCCCTTAGTAAGTAAATTTTACTTAATTTCTGCACAACAAATAAGGACTGCAAAACATTCTGCATCAACTGAACGCAAATCAATTACTTTATTAAGCTAAGCCCTTCCTAGATTGATGGGATTTTAACCCACAAGGGGTACGTTAACAGCTAAATAACCTAATCAACTGGCTTCAATCTACTTCTCCCGCCGTTAGGAAAAGAGCGAGAGAAGCCCCGCTGGGAGGTGAAGCTGCTTCTTTCCTAATTTGCAATTCAATGCGATGATTCACCCTCAGGGCTGGTAAAAAGAGGGTTCACTCCTCTGTCTTTTAGATTTACAGTCTAATGCTTGCTCTAATATTTTACCCCTACCTATGTTCATAAACCGCTGACTATTTTCAACTAATCACAAAGACATTGGGACATTATATTTATTATTTGTGCATGGGCGGGGCAGTAAATTTACCATACAAGCCTCCTAATTCGAACAGAACTGGGACAACCCGGAAGCCTAATAGAAGACGACCATGTATACAATGGTTATTTATGTCACCGCCCACGCATTCATCATAATTTTTTTCATAGTAATGCAATTATGGTTGGGGGTTTTGGGAACTGACTTATTCCTTTAATGAGTGGCGCTCCCAGAATACCGCCACATTTCCTCAGATAAATAACATAAGCTTCCTGACTTCTACCACCACTCCCTACTCCTACTGCTTGCATCATCTTTAGAAGCCGGTGCAGCACTGGTTTAAGGACAGTCTGCCCACCCTAGCGAATAGTTGTCCCACCCAGGAGCCTCCTTAGATTTAACCATTTTTCACTCACATTTCTGTGAGCATTTCTTCTATTCTTGGAGCTAATAACTTTATTACAACAATTGTAAATGCGGAAACCGCCAGCCATGACTCAATATCAAACTCCGTTGTTTGTGCTGAATCAAATCCCCTGATGCTACAGTCCTTCTTTTACTTTCTCTTCCAGTTCTACCAGCTGCAGGATTACCATATATTAACTGACCGTAATTTAAATACTACTTTCTTTGATCACTGCTGAGCATGGCGACCCTATCCTATACCAACACTTATTCTGATTCTTCAGTCACCCTAGTATATATTCTAATCTTACCGGGTTTGGAATAATTTCACACATTGTAACATATTATTCTAATAAAAGAACCCTTTGATTGTATGGGCATGGTATGAGCCATAATATCTATTGGTTTCCTAGGCTGTGTATGAGCCCATCACATATTCATGATGAAGATAGTCACTTAGATACTCGTGCATATTTTACATCAGCTACCATAATCATTGCCTTCCCACTGGAGTAAAAGTATTTAGCTGATTAGCCACACTGCACGGCGACAATATCAAATGATCTCCGCAATATTGTGAGCCCTGGGCTTTATTTTTTCTTTTTACCGTGGTGGACTAACAGGAATTGTGTTGGCTAACTCATCATTAGATATTGTATTACATGATACATACTATGTAGTAGCCACTTTCACTATGTGTTATCAATAGGAGCAGTATTTGCCATTATAGAAGTTTATTCAGCAATACGTAA

>a89446db-1714-4359-8f64-5857f023ce3b

ATTGTACTTCGTTCAGTTACGTATTGCTAACTGGCAACTAAATTATATAAAGCCAATACCGTTTCTATCCCCACTTATGTCTTCTACCTGTCTCCTACCCCTAACTCACTAATACTTATAACCCAGAAATTTAGGTTAATAAGACCAAAGAGCCTTCAAAGCCCTTGGATAGTAAATTTTACTTAATTTCTGCACAACAAATAAGGACTGCAAAACTTTATTCTGCATCAACTGAACGCAAATCAATTACTTTAATTAAGCTAAGCCCTTCCTAGATTGATGGGATTTTAACCCACAAAAATTTAGTTAACAGCTAAATAACCTAATCAACTGGCTTCAATCTACTTCTCCGCCGTTAGGAAAAAAGGCGGGAGAAGCCCGGCAGAATTGAAGCTGCTTCTTTAATTTGCAATTCAATGTGATAGTTCACCTCAGGGCTGAGGTAAAAGAGGGTTCCACTCCTCTGTCTTTAGATTTACAGTCTAATGCTTGCTCAGCCATTTTACCCCTACCTATGTTCATAAACATGACTATTTTCAACTAATCACAAAGACATTGGGACATTATATTTATTATTTGGCGCATGGGCGGGGGCAGTAGGTACAGCCCTAAGCCTCCTAATTCGAACAGAACTAGGACAACCCGGAAGCCTGACAAGAACTGACCATGTATACAATGTTATTGTCACCACCACGCATTCATCATAATTTTTTTCATAGTAATACCAATTATGATTGGGGTTTTACTTTGGGAACTGACTTATTCCTTTAATAATTGGCGCTCCGATATGCTTGCTTTCTCGAATAAATAACATAGCTTCTGACTTCTACCCTCCCTACTCTACTTTCTTGCATCATCAACTGCGAAGCCGGTGCCGGCACTGGTTGGACAGTCTACCCACCCCTAGCAGGCAATTTATCCCGCAGGAGCCTCTGTAGATTTAACCATTTTTGCACTACATTTGTAGGCATTTCTTCTATTCTTGGAGCTATTAACTTTACATTACAACAGGTATGAATGCTGGAAACCGCCAGCCATGACTCAATATCAAACTCCGTTGTTTGTGTGATCCGTCCTAATTACCAGTCCTTCTTTTACTTTCTCTTCCAGTTCTAGCTGCAGGGATTACCATACTGTTAACTGACCAAAAAATAATTTAAATGCTACTTTCTTTGATCCTGCTGGTGGCGTGACCCTATCCTATACCAACACTTATTCTGATTCTTCGGTCACCACAAAGTATATATTCTAATCTTACCGGAGTTTTGGAATAATTTCACACATTGTAACATATTATTTAATAAAAGACCTTTGATTATATGGGCATAGGTATGAGAGCCATAATATCTATTGGTTTCTAGGCTTTATTGTATGAGCTGCTCACATATTCACAGTAGGAATAGATGTAGATACTCGTGCATATTTTACATCAGCTACCATAATCTTGTTGCTATTCCCTGGAAGTAAAAGTATTTAGCTGATTAGCACACTGCACGGCGGTAATATCAAATGATCACCCGCAATATTATGAGCCCTGGGCTTTATTTTCTTTTTACCGTGGTGGACTAACGGGAATTATGTTAGCTAACTCATCATTAGATATTGTATTACATAATTATACTATGTAAACCTTTCACTATGTGTTATCAATAGACGATATTATTACCATTATAAGGGGGATTTATTCATGGTTCCGCTCTTTTCAGGCTATACTTTCTTGACCAAACGTATGCCAAAATGCTTCACCATTACCCTATTTGTCGGCGTAAATTTAACTTTCTTCCCACAACACTTCCTTGGCTTATCCGGAATACCTCGACGACCACTCAGACTATCAAAGACATACTACCATGAAATATATATCGTCATCTATCGGTTCATTTATTTCTGCTTACAGCAGTAGTCCTGATGGTTTTTATAGTACAGAGAAGCTTTCTCTTCAAAAACGAAAAGTCTTAGCCATTGAGCAACTATCACCAATCGACCAGTCTGTGCCCTCCTCCATACCACACATTCGAAGAGGCAACTTTTTTGTTAAATCCTAGACGAAAAAGGAAAGGATTTGAACCCCAAAAATTGGTTTCAAGCCAATCCCATATACCCTATGACTTTTTCAATAAGATATTAGTAAAATAATTACATAACTTTGTCAAAGTTAAATTATAGACTAAATATCTATATATCTTATCTTTTCAATACTCAACTAGGTTTACAAAATGCCACGTCACTATGTAGAAGAACTTGCTTCTTTCCAGCCATGCACTCATAATTATTTTCCTGATTAGTTCCCTTAGTCTTCTATATTATTTCCCTTATCCTTACCACAAAACTCACTCATACTAGCACCATAAATGCTCAAGAGATCGAAATAATCAAGACTATTCTCCCCTGCGCGATTCTAATTACAATTGCCACTCCCATCACTGCGTATTCTATATAGCAACAGACGAATTTAATAAACACTATTTAACCATAAACAATTGGTCACCCGTATTGATACAGCAATACGTAA

>6b8b6a68-3457-45cc-95ee-ada4e2e052e0

AGTAGCCACTTCGTTCCAGTTACGTATTGCTTAATTTCTGCACACAACAAATAAGGACTGCAAAACTTTGTGCATCAACTGAACGCAATCGTTACTTTAATTAGCTAAGCCCTTCCTAGATTGATGGGATTTTAACCCACCAAAATTTGATTAACAGCTAAATAACCTAATCAACTGGCTTCAATCTACTTCTCCCGCCGTTAGGGAAAAAGGCGGGGGCCCGGCTAGGGTGAAGCTGCTTCTTTGGGTAATTCAATGTGATAGTTCACCTCAGGGCTGGTAAAAAGGGTTCACTCCTCTGTCTTTAGATTTACAGTCTAATGCTTGCTCAGCCATTTTACCCCTACCTATGTTCATAAACCGCTGACTATTTTCAATAATCACAAAGACATTGGGACATTATATTTGATAACTGGCGCATGGGCTTGGTGACAGGTACAGCCCTAAGCCTCCTAATTCGAATAAAACTAGGACAACCCGGAAGCCTAATAGAAGACGACCATGTATACAAATGTTATTGTCACCGCCCACGCATTCATCTTAGTTTTTCATAGTAATACCAATTATGATTGGGGGTTTTTGGGAACTGACTTATTCCTTTAATAATTGGCGCTCCCGATATAGCATTTCCTCGAACCGAAACTAACATAAGCTTCTGACTTCACCACCCTCCCTACTCCTACTGCTTGCATCATCAGCTTTAGAAGCCGGTGCCGGCACTGGTTGAACAGTCTACCCACCTAGCAGGCAATTTATCCCACCCAGGAGCCTCTGTGATTTAACCATTTTTTTACCCCCATTTGTAGGCATTTCTTCTATTCTTGGGCTATTAACTTTTATTACAACAATTGTAAATACGACCGCCAACCATGACTCAATATCAAACTCCGTTGTTTGTGTGATCCGTCTAATTACTGCGGTGTGTACTTACTTTCTCTTCCAGTTCTAGCTGCAGGGATTACCATACTATTAACTGACCGTAATTTAAATACTACTTTCTTTGATCCTGCTGGTGGCGGCAGACCTATCCTATACCAACACTTATTCCTGATTCTTCGGTCACCTAAAATATATATTCGCTCTTACGGGTTTTGGAATAATTTCACACATTCGTAGCATATTATTCTAATAAAAAAGAACCGCTTGGTTATATGGGTATGGTATGAGCCATAATATCGTAGTTTCTGGGCTTTATTGTATGAGCAGCAATACATAGCA

>4d025672-88ac-4d1b-a951-58324c0f054b

GTGTACTTCGTTCAGTTACGTATTGCTCTAATAATAATATCCCTAGGAGGTCTACCCCCACTAACAGGTTTTCCCCCAAATGAGCTATTATACAAGAACTTATTAAAAATGATAACCCCTTATAATAAACTTTACTAACATTAATAAATTTATATTTTTTTATATACGTTTAATATATTATATCTCAATGACAATATTCCCAACATCAAATAACACAAAAATCAACTGGCAACTAAATTATATAAGCAGCCTACCGTTTCTATCCCCACTTGTAGTGTCTTCTACCTGTCTCTACCCCTAACTCCACTAATACTTAACTTAGAAATTTAGGTTAATAAGACCAAGAGCCTTCAAAGCCACTTAGTAAGTAAATTTTACTTAATTTCTGCACAACAAATAAGGACTGCAAAACTTTTATTCTGCATCAACTGAACGCAAATCAATTACTTTAATTAAGCTAAGCCTTCTAGATTGATGGGATTTTTAACCCACAAAAATTTAGTTAACAGCTAAATAACCTAATCAACTGGCTTCAATCTGCTTCTCCGCCGTTGGGAAAAAGGCGGGAGAAACTTGGCAGAATTGAAGCTGCTTCTTTGAATTTGCAATTCAATGTATTAGTTCACCTCAGGGCTGGTAAAAAGAGGGTTCTCCTCTGTCTTTAGATTTACAGTCTAATGCTTGCTCAGCCATTTTACCCCTACCTATGTTCATAAACCGCTGACTATTTTCAACTAATCAAGACATTGGACATTATATTTATTATTTGGCGCATGGGCGGAGACATTAGGTACAGCCCTAAGCCTCCTAATTCGAGCAGAACTAGGACAACCCGGAAGCCTAATAATAGAAATGACCATGTATACAATGTTATTGTCACCGCCCACGCATTCATCATAATTTTTTCATAGTAATACCAATTATGATTGGGGGTTTTGGGAACTGACTTATTCCTTTAATAATTGGCGCTCCCGATATAGCATTTCCTCCGAATAAATAACATAAGCTTCTGACTTCTACCACCCTCCTACTCCTACTGCTTGCATCATCAACTTTAGAAGCCGGTGCCGGCACTGGTTGGACAGTCTGCCCACCCCTAGCAGGCAATTTATCCCACCCAGGAGCCTCTGTAGATTTAACCATTTTTTCACTACATTTAGCAGGCATTTCTTCTAATCTTGGAGCTATTAACTTTATTACAACAATTGTAAATATGAAACCGCCAGCCATGACTCAATATCAAACTCCGTTGTTTGTGTGATCGTCTAATTACTGCATGATCCTTCTTTTACTTTCTCTTCCAGTTCTAGCTGCAGGGATTACCATACTATTAACTGACCGTAATTTAAATACTACTTTCTTTGATCCTGCTGGTGGCGGCGACCCTATCCTATACCAACACTTATTCTGATTCTTCGGTCGCCACAGGCGTACGTAA

>1c82bada-c25f-4a1e-ba89-72d67763ec87

ATTGTACTTCGTTCAGTTACGTATTGCTGATTTTAACCCACAAAAATTTAGTTAACAGCTAAATAACCCTAATCAACTGGCTTCAATCTACTTCTCCCGCCGTTGAGGAAAAAAGGCAGGAGAAGCCTTTGGAGTGAAGCTGCTTCTTTAATTTGCAATTCAATGTGATAGTTCACCTCAGGGCTGGTAAAAGAGGTTCACTCCTCTGTCTTTAGATTCTGATCTAATGCTTGCTCAGCCATTTACCTACCTATGTTCATAAACCGCTGACTATTTTCAACTAATCACAAAGACATTGGGACATTATATTTATTATTTGGCGCATGGGCGGAGCAGTAGGTACAGCCCTAAGCCTCCTAATTCAGACAGAACTAGGACAACCCGGAAGCCTAATAGAAGACGACCATGTATACAATGTTATTGTCACCGCCCACGCATTCATCATAATTTTTTTCATAGTAATACCAATTATGATTGGGGTTTACTGGGAACTGACTTATTCCTTTAATAATTGGCGCTCCCGATATAGCATTTCCTCGAATAAATAACATAAGCTTCTGACTTCTACCACCCTCCTACTCCTACTGCTTGCATCATCAACTTTAGAAGCCGGTGCCGGCACTGGTTGAACAGTCTACCCACCCCTAGCAGGCAATTTATCCCACCCAGGAGCCTCTGTAGATTTAACCATTTTTTCACTACATTTAGCAGGCATTTCTTCTATTCTTGGAGCTATTAACTTTATTACAACAATTGTAAATATGAAACCGCCAGCCATGACTCAATATCAAATTCGTTGTTTGTGTGATCCGTCCTAATTACTGCAGTCCTTCTTTTACTTTCTCTTCCAGTTCTAGCTGCAGGAGATTACCATACTATTAACTGACCGTAATTTAAATACTACTTTCTTTGATCCTGCTGGTGGCGGCGACCCTATCCTATACCAACACTTATTCTGATTCTTCGGTCACCCTGAAGTATATATTCTAATCTTACCGGGTTTTGGAATAATTTCACACATTGTAACATATTATTCTAATAAAAAAGAACAGCACTTTGGTTATATAGGCATGGTATGAGCCATAATATCTATTGGTTTCTAGGCTTTGTATAGCAATGCGTAACCT

>5e180c16-60e0-444e-9ce6-9d498fd34cee

ATTGTACTTCGTTCAGTTACGTATTGCTGCCAATACCGTTTCTATCCCCACTTGTAGTGTCTTCTACCTGTCTCCTACCCTAACTCCACTAATACTTATAACTTAGAAATTTAGGTTAATAAGACCAAAGAGGAAGCCTTCAAAGCCCTTAGTAAAGTAAATTTTACTTAATTTCTGCACAACAAATAAGGACTGCAAAACTTTATTCTGCATCAACTGAACGCAAATCAATTACTTTAATTAAGCTAAGCCCTTCCTAGATTGATGGGATTTTAACCCATAAAAATTTAGTTAACAGCTAAATAACCTAATCAACTGGCTTCAATCTACTTCTCCCGCCGTTAGGGAAAAAGGCGGGGAGAAGCCCCGGCAATTGAAGCTGCTTCTTTGAATTTGCAATTCAATGTGATAGTTCACCTCAGGGCTGGTAAAAAAGAGGGTTCACTCCTCTGTCTTTAGATTTACAGTCTAATGCTTGCTCAGCCATTTTACCCCTACCTATGTTCATAAACCGCCTGACTATTTTCAACTAATCACAAAGACATTGGGACATTATATTTATTATTTGGCGCATGGGCAGGGGGCAGTAGGTACAGCCCTAAGCCTCCTAATTCGAACAGAACTAGGATAACCGGAAGCCTAATAGAAGACGACCATGTATACAATGTTATTGTCACCGCCCACGCATTCATCATAATTTTTCATAGTAATACCAATTATGATTGGGGGGTTTTGGAACTGACTTATTCCTTTAATAATTGGCGCTCCCGATATAGCATTTTCCTCGAATAAATAACATAAGCTTCTGACTTCTACCACCCTCCCCTACTCCTACTGCTTGCATCATCAACTTTAGAAGCCGGTGCCGGCACTGGTTGGACAGTCTACCCACCCCTAGCAGGCAATTTATCCCACCCAGGAGCCTCTGTAGATTTAACCATTTTTTCACTACATTTAGCAGGCATTTCTTCTATTCTAGCAATACGTAA

>0d8374c6-621e-4ef4-a35c-b234f25813f9

CGTGCTTCGTTCAGTTACGTATTGCCTACGTTTCTATCCCACTTGTAATTCTTCTACCTGTATACTACTAACTCCCATAATACTTATAACACGAGAAATTTAGGTTAATAAGACAAAGAGCCTTCAAAGCCCTTAGTAAATAAATTTACTTAATTTCTGCACAGCAAATAAAGGACTGCAAAACTTTATTCTGCATCAACTGAACACTTAAATCAATTGCTTTAATTAGCTGTAATACTTCTCTAAATTCGTCAGTTTTAACCCTAAAAAGAATTTAAATTGTAGCTAAATAACTCAATCATTGTGCAATCTCACTTCTGCGATTGGAAAAAGGCGAGAAAGCTAACAGGAGAAGCTGCTTCTTTGTTTACATTCAATGTGATAGTTCACCTCGGGCTGGTAAAAAGAGGTTCACTCCTCTTGTCTTTTGAATTTACAGTCTAATGCTTGCTCAGCCATTTTACCCTACCTATGTTCATAAACCGCTGACTGTTTTCAACTAATCACAAAGACATTGGGACATTATATTTATTATTTTGGCGCATGGGCAGGGTGATGAGTACAGCCCTAAACCTCCTAATTCATTGAAACCTAGAACCAACCCGGAAACCCTAATAGAATGATAATAACACTGCAATGTTATTGTCTACTTACGCATTCATCATAATTTTTCATAGTAATACCAATTATGATTGGGGTTTTTGGGAACCAACTTATTCCTTTAATAATTGTAACACTCCAATATAGCATTTCCTCGATAAATGCTGCAAAACTTCATTGCTGCCTTCTCTCTACACCGCTTGCATCATCAGCTTTAGAAGCCGGTGCCGGCACTGGTTGAACAGTCTACCCACTAGCAGGCAATTTGGCATCCCACCCAGGAACTCTGTGAGATTCGCTCATTTTTTTCACTACATTTAATGACACATTTCTTCTATTCTTGGAGCTATACTTTATTACAACAATTGGCAAATATG

>b36b58a4-535a-4b22-8df3-dafe90fd475f

TTGTACTTCGTTCAGTTACGTATTGCTCACATAGGATGAATAACGTATTATATTAAACCTAATATTACTATATTAACTTTAATTATTTATATTTTCTAACAATCTCTACATTAATAATCTTTTATTTAACACTCAAATGTAACAACCTATCCCTATCACATACCTGAAACAAACTAGCATGAATAATACCCATTATTCCACTAATAATAATATCCCTAGGAGGTCTACCCCCACTAACAGGTTTTTCCCCCAAATGAGCTATTATACAAGAACTTATTAAAAATGATAACTTAATTATTCCCCTTATAATAGCTTTACTAGCATTAATAAATTTATATTTTTATATACGTTTAATATATTATATCTCAATGACAATATTCCCAACATCAAGTAACACAAAAATCAACTGGCAACTAAATTATATAAAGCCAATACCGTTTCTATCCCCACTTGTAGTGTCTTCCTACCTGTCTCTACCCACCTAACTCCACTAATACTTATAACTTAGAAATTTAGGTTAATAAGACCAAGAGCCTTCAAAGCTACTTAGTAGAAGTAAATTTTTAGCAATTTCTGCACAACAAATAAAGGACTGCAAAACTTTAATACCATCAACTGAACGCAAATCAATTACTTTAATTAAGCTAAGCCCTTCCTAGATTGATGGGATTTTTAACCCACAAAAATTTGGATTAGCGTAATAACCTAATCAACTGGCTTCAATCTACTTCTCCGCCGTTAGGGAAAAAGGCGGGAAGCCCCGGCAGGTGAAGCTGCTTCTTTGAATTGCAATTCAATGTGATGGATTCACCTCAGGGCTGGTAAAAAAGAGGGTTCACTCACTCTGTCTTTAGATTTACAGTCTAATGCTTGCTCCAGCCATTTTACCCCTACTATGTTCATAAACCACTGACTATTTTCAACTAATCACAAAGACATTGGGACATTATATTTATTATTTGGCGCATGGGCGAAGACAGTAGGTACAGCCCTAAGCCTCTAATTCGAACAGAACTAGGACAACCCGGAGAGCCTAATAGAAGACGACCATGTATACAATGTTATTGTCACCGCCCACGCATTCATCATAATTTTTTTCATAGTAATACCAATTATGATTGGGGGTTTTGGGAACTGACTTATTCCTTTAATAATTAGCGCTCCCGATATAGCATTTCCTCGAATAAATAACATAAGCTTCTGACTTCTACCACCCTCCCTACTCCTACTGCTTGCATCATCAACTTTAGAAGCCGGTGCCGGCACTGGTTGGACAGTCCTACCCACCCTAGCAGGCAATTTATCACCAGGAGCCTCTGTGATTTAACCATTTTTTCACTACGTTAGCAGGCATTTCTTCTATTCTTGGAGCTATTAACTTTATTACAACAATTGTAAATATGAAACCGCCAGCCATGACTCAATATCAAACTCCGTTGTTTGTGTGATCCGTCTAATTACTGCAGTCCTTCTTTTGCTTTCTCTTCCAGTTCTAGCTGCAGGGATTACCATACTGATAACTGACCGTAATTTAAATACTACTTTCTTTGATCCTGCTGGTGGCGAGCGACCCTATCCTATACCAACACTTATTCTGATTCTTCGGTCACCCTGAAGTATATATTCTAATCTTGCCAAGGTTTTGAATAATTTCACACATTGTAATATTGATACAATGAAACCCTTTGGTTATATGGGCATGGTATGAGCCATAATATCTATTGGTTTCCTAGGCTTTATTGTATGAGCCCATCCATATTCACAGTAGGAATAGATGTAGATACTCGTGCATATTTTACATCAGCTACCATAATCATTGCTATTCCCACTGGAGTAAAGTATTTAGCTGATTAGCCACTGCACGGCGGTAATATCAAATGATCTCCCACCACAATATTACCTTGGCAGGCCCTGGGCTTTATTTTTCTTTTTACCGTGGGTGGACTAACAGGAATTTAATGTTAGCTAACTCATCATTAGATATTGTATTACATGATACATACTATGTAGTAGCCCACTTTCACTATGTGTTATCAATAGGAGCAGTATTTGCCATTATGGGATTTATTCACTGATTCCCGCTCTTTTCAGGCTATACTCTTGACCAAACATATGCCAAAATCCACTTCACCATTATATTTGTCGGCGTAAAGATTTAACTTTCTTCCCACAACACTTCCTTGGCTTATCCAGGAACCTCGACGATACTCAGACTATCCAGACGCATATACTACATGAAATATCGTCTCATCTATCGGTTCATTTATTTCACTTACAGCAGTAGTCCTGATGGTTTATAATTTGAGAAGCTTTCTCTTCAAAACGAAAAGAATCTTAGCCATTGAGCAATATCACCAATCTAGAATGATTATACGGCTGCCCTCCTCCTTACCACACATTAAGAGGCAACCTGCGTTAAATCCTAGACGAAAAGGAAAGGATTTGAACCCCCAAGAAATTGGTTTCAAGCCAATCCCATATACCCTATGACTTTTCAATGAATATTAATTGAAATAAGTACATAACTTTGTCAAAGTTAAATTATAGACTAAATATCTATATCTTAATAGCAACACCAGCTCAACTAGGTTTACAAAATGCCACATCACCTATCATAGAAAGAACTTATTGCTTTCCACGACCATGCACTCATAATTATTTTTCCTGATTAGTTCCTTAGTCTTATATATTATTTCCTTATACTTGCCACAAACTCACTCATACTAGCACCATAAATGCTCAAGAGATCGAAATAATCTGAACTATTCTCCCTGCACTGATTCTAAGTACAATTGCCTCCATGCACGTATTCTACTTAATATGACAGACGAATTTAATAAACCTTATTTAACCCTTAAAGCAATTGGTCACCAATGATACTGAACTATGAATATTCCGACTATGAAGACTTAGTATTTGACTCTTACATTATGCCAACATATTTCCTTGAGCCAGGGAATTTCGACTCCTCGAAGTTGATAACCGAACAACTTTACCTATAGAAGCGGATATTCGCATATTAATGCCTGTCACAAGACGTCTTACACTCATGAGCCATGCCATCACTAGGCATTAAAAAACAGATGCAATTCCTGGACGTTTAAACCAAGCCATACTAGCCTCTATACGACCAGGCCTATTTTATGGACAATGCTCGGAAATTTGCAGTTAATCAGCTTTATACCTATTGTTCTAGAATTCATTTATTTCGAATTTCGAAGTATAGGCTTCATACTTATATATTGTATCACTGTAAAACTATTAACTTAGCATTAACCTTTTAAGTTAAAGATTGAAGAGAACAAACTCTCTATAATTGATACCTCAATAAATGTTTCACCGTGACCAATAGTAATTATATCTATAATTGTTACCTTATTTTTATATTATACAATTGAAAATACTGAACTTTACTTTCATTATTACCCACTACCAAAATTAAGTAAAAGTATACAAAAACATAAAACAACTTGAGAACTAAAATGAACCAAAATCTATTTGCCTCATTCAATATTCCAACAA

>ef55eebd-30c1-47a7-98c5-585121a05443

GTACTTTACGTTCAGTTACGTATTGCTTTCTAACAGGGACCGTAATCACAATTTTAAGCTCATTGATTCCTAGCCTGAATGAAGCTTAGAATTAAATATACCAGCCATCGTACCAATCCTTGCCAAAAGTACCAATCCCCCGCTCCACAGGCATCCACCAAATGTTTTTTAATTCAAGCAACAGCATCAATACTTCTATTAGTATCCATTTTCCTTAACAATCTACTAACAACAATGAACAATCAATGTCCTTATAACCAAATATTATCCACAATAATATTTTATTGCTCTAACAATAAAAATAGGGATAGCCCACTTCGCTGACTCCCAGAAATTGCAAGATCCCTCTAATCCCAGCTATAATTATTCTCACGTGACAAAAACTCACTTTGTATCAATTCTCCTCCCAAATTTTAAAATCAGCAAACTAAACAGTTCTAACAATCTCAGTTCTATAAATTATAATTGGCAGCTGAGGGGACTCAACCAAACACAACTCCGCAAAATCTAGCCTATTCTTCAATTACTCACATAGGATGAATAATAGCAGTATTGTAATACGACCCTAATATTACTATAACAACTTTTAATTATTTATATTGTACATTTTCCTAACAATCTCTACATTAATAATCTTTTATTTAACCTCAAATGTAACAACCCTATCCCTATCATACCTGAAACAAACTGAACAATACCCATTATTCCACTAATAATAATATCCCTAGGAGGTCTACCCCCACTAACAGGTTTTCCCCCAAATGAGCTATTATACAAGAACTTATTAAAAATGATAGCTTAATTATTCCCCTTATAATAGCTTTACTAACATTAATAAATTTATATTTTTATATACGTTTAATATATTATATCTCAATGACAATATTCCCAACATCAAATAACACAAAAATCAACCAGCAACTAGAATATATAAAGCCAATACCGTTTCTATCCCCACTTGTAGTGTCTTCTACCTGTCTCCTACCCCTAACTCCACTAATACTTATAACTTATTTAGGTTAATAAGACCAAAGAGCCTTCAAAGCCCTTAGTAAGTAAATTTTACTTAATTTCTGCACAACAAATAAGGACTGCAAAACTTTATTCTGCATCAACTGAACGCAAATACTTTAATTAGCTAAGCCCTTTCCTAGATTGATGGGATTTTAACCCACAAAAATTTAGTTAACAGCTAAATAACCTAATCAACTGGCTTCAATCTACTTCTCCCGCCGTTAGGAAAAAGGCGGGAGAAGCTAACGAATTGAGCTTCATCGAATTTGCAATTCAATGTGACCACCTCAGGGCTGGTAAAAGAGGGTTCACTCCTCTGTCTTTAGACCTGATCTAATGCTTGCTCAGCCATTTTACCCCTACCTATGTTCATAAACCATGACTATTTTTCAACTAATCACAAAGACATTGGAACATTATATTTATTATTTGGCGCATGGGCAGGGGCAGTAGGTACAGCCTAAGCCTCTAATTCGAACAGACGGGACAGCCCGGAAGCTAATAGAAGACGACCATGTATACAATGTTATTGTCACCGCCCACGCATTCATCATAATTTTTTCATAGTAATACCAATTATGATTGGGGGTTTTAAGAGACTGACTTATTCCTTTAATAATTGGCGCTCCCGATAGCATTTCCTCGAATAAATAGCAGCTTCCACGGCCTTACCACCCTCCCCTACTCCTACTGCTTGCATCATCAACTTTAGAAGCCGGTGCCGGCACTGGTTGGACAGTCTACCCACCCTAGCAGGCAATTTATCCCACCCAGGAGCCTCTGTAAGATTTAACCATTTTTTCACTACATTTAGCAGGCATTTCTTCTATTCTTGGAGCTATTAACTTTATTACAACAATTGTAAATATGAAACCGCCAGCCATGACTCAATATCAAACTCCGTTGTTTGTGTGATCCGTCCTAATTACTGCAGTCCTTCTTTTACTTTCTCTTCCAGTTCTAGCTGCAGGGATTACCATACTATTAACTGACGTAATTTAAATACTACTTTCTTTGATCCTGCTGGTGGCGGCGACCCTATCCTATACCAACTTACTTATTCTGATTCTTCGGTCACCCTGATATATATTCTAATCTTACCGGGTTTTGGAATAATTTCACACATTGTAACATATTATTCTAATAAAAAAGTTTCCTTTGGTTATATGGGCATGGTATGAGCCATAATATCTATTGGTTTCTAGGCTTTGTGGCATGAGCCCATCACATATTCTGTTAGGAATAGATGTAGATACTCGTGCATATTTTACATCAGCTACCATAATCATTGCTATTCCCACTGGAGGTAAAAGTATTTAGCTGATTAGCCACACTGCACGGCGGTAATATCAAATGATCTCCCGCAATATTATGAGCCCTGGGCTTTATTTCTTTTACCGTGGGTGGACTAACAGGAATTGTGTTGGCTAACTCATCATTAGATATTGTATTACATGATACATACTATGTAGTAGCCCACTTTCTATGTGTTATCAATAGGAACAGTATTTGCCATTATAGGGGGATTATTCACTGATTCGCTCTTTTCAGGCTATACTCTTGACCAAACATATGCCAAAATCCCTTCACCATGCATATTTGTCGCGTAAATTTAACTTTCTTTAACACTTCCTTGGCTTATCGGAATACCTCGACGATACTCAGACTATCCAGACGCATATACTACATGAAATATCGTCTCATCTATCGGTTCATTTATTTTACTTACAGCAGTAGTCCTGATGGTTTTTTATAATTTGAGAAGCTTTCTCTTCAAAACGAAAGTCTTAGCCATTGAACAACTATCCACCAATCACAGAATGATTATACGGCTGCCCTCCTCCCTTGCCACACATTCGAAGAGGCAACTTACGTTAAATCCTAGACGGAAGGATTTGAACCCCAAGTGGTTTCAAGCCAATCATATACCCTATGACTTTTCAATAAGATATTAGTAAAATAATTATAACTTTGTCAAGTTAAATTATAGACTAAATATCTATATATCTTAATAACAACACCAGCTCAACTAGGTTTACAAAATGCCACATCACCTATCATAGAAGAACTTATTGCTTTCCACGACCATGCACTCATAATTATTTTCCTGATTAGTTCCTTAGTCTTATATATTATTTCCTTATACTTACCACAAAACTCTTTCATTACTAGCACCATAAATGCTCAAGAGATCGAAATAATCTGAACATTACTCCTGCTGATTCTAATTACAATTGCCCTCCCATCACTGCGTATTCTATATATGACAGACGAATTTAATAAATGTATTTAACCCTTAAAGCAATTGGTCACAATGATACTGAACCTATGAATATTCCGACTATGAAGACTTGATACGACTCTTACATTATGCCAACATAATTTCAGCGAGCCAGGGAATTTCGACTCCTCGAAGTTGATAACCGAACAACTTTACCTATGAAGCAGGATATTCTGTATTAATCTCATCACAAGACGTCTTACACTCATGAGCCGTACCATACTGACGTAAAAACAGGTCACAATTCCTGGACGTTTAAACCAAACCATACTAGCCTCTATACGACCAGGCCTATTTTGTGAACCCAATGTCGGAAATTTGCGGGTCCAATCACAGCTTTATACCTATTGTTCTAGAATTCATTCCAAGATTTCGAAGTATGAGCTTCATACTTATATATTGTATCACTGTGAAGCTAACTTAGCATTAACCTTTAAAGTTAAGATTGAGAACAAACTCTCTATAGTGAATGCCTCAACTAAATATTTCACCGTGACCAATAGTAATTATATCTACCGTAATTGTTACCTTATTTTATATTATACAATTGAAAATACTGAACTTTACTTTCCATTATTACCCACTACCAAAATTAGTAGAAACACAAAAACATAAAAAAACAACTTGAGAACTACAAAATGAACCAAAATCTATTTGCCTCATTCAATATTCCAACATATAGGAGTACCCTTAGTATTTTAATTATTACCTCCCCACTACATTAATTTTATCCTCCAAAAAACTTATTCAACAACCGACTCTTCTTCAATTCAACAATGGCTAATTCAACTAACACTTAAACAAATAATATTAACCCACACCACTAAAGGGCGAACCTGATCCCTTATACTCCTAGCCTAATTTCTTTATTGCCCTAAATAACATTCTCGGACTTACACCATATGCATTTACACCAACCACCCAACTGTCAATAAATCTAGGCATAGCTATTCCTCTATGAAAGCAACTGTACAGCAATACGTAACTTT

>c035ceb8-ae61-4b7d-9b96-9840a6c1eda7

ATGTACTTCGTTCAGTTACGTATTGCTTAGATATTTAGTCTATAATTTAACTTTGACAAAGTTATGTAATTGTTACTAATATCTTATTGAAAAAGTCATAGGAGTATATGGGATTGGCAGGCTCAATTTTTGGGGGTTCAAATCCTTCCTTTTTCGTCTAGGATTTAACGTAGTTGCCTCTTCGAATGTGTGGTAAGGAGGAGGGCAGCCGTATAATCATTCTTAGATTGGTGGATAGTTGCTCAATGGCTAAGACTTTTCGTTTTTGAAGAGAAAGCTTCACCAAATTATAAAAACCATCAGGACTACTGCTGTAAGTGAAATAAATGAACCGATAAGTCGAGACGATATTTCATGTAGTATATGCGTCTGGATAGTCACAATTATCGTCGAGGTATTCCGGATAAGCCAAGGAAGTGTTGTGGGAAGAAAAGTTAAATTTACGCCGACAAATATAATAGTGAAGTGGATTTTGGCATATGTTTGGTCAAAATTATAGCCTGAAAAGAGCGGGAATCAGTGAATAAATCCCCCTATAATGGCAAATTCCTGCTCCTATGATAACACATAGTGAAAGTGGGCTACTACATAGTATGTATCATGTAATACAATATCTAATGATGAGTGTAACACAATTCCTGTTAGTCCACCCACGGTAAAGAAAAATAAAGCCCAGGGCTCATAATATTGCGGGAAGTCATTTGATGTACACTTAAGTACGGTAATATTAATCAGCTAAATACTTTTGCTCCCACGATGGGAATAGCAATGATTATAAATTAATGATGTAAAATATGCACGAGTATCTACATCTATTCCTACTGTGAATATGTGATGGGCTCATACAATAAAGCCTAAACCAATGATATTATGGCTATACCATTACACTTATAACCAAAGGAGTTTCTTTTTTATTAGAATAATATGTTACAATGTGTGAAATTATTCCAAAAAACCCAGTAAGGTAGAATATATACTTCAGGGTGACCGAAGAATCAGAATAAGTGTTGGTATGGGGATAGGGTCGCCGCCACCAGCAGGATCAAAGAAAGTAGTATTTAAATTACGGTCAGTTAATAGTATGGTAATCCCTGCAGCTAGAACTGGAAGAAAGTAAAGAAGGACTGCAGTAATTAGGACGGATCACACAAGCAACGGAGTTTGATATTGAGTCATGGCTGGCGGTTTCGTATTTACAATTGTTGTAATAAAGTTAATAGCTCCAAGAATAGAAGAAATACCTACTAAATGTAGTGAAAAAAATGGTTAAATCTACAGAGGCTCCTGGGTGGGATAAATTGCCTGCTGGGGGTGGTGAACTGTCCAGCCAGTGCCGGCACCGGCTTCTAAAGTTGATGATGCAAGCAGTAGGAGTAGGAGGGTGGTAGAAGTCAGAAGCTTATGTTATTTATTCGAAATGCTATATCGGGAGTAATTATTGAGAATAAGTCAGTTCCCAAAACCCCAATCATAATTGGTATTACTAGAAAAAAATTATGATGAATGCGTGGGCAGTGACAATAACATTGTATACATGGTCGTCTTCTATTAGGCTTCCGGTTGTCCCTAGTTCTGTTCGAATTAGGAGGCTTAGGGCTGTTCTGCCCCCGCCCATGCGCCAAAATAATAAATATAATGTCCCAATGTCTTTGTGATTAGTTGAAAATAGTCAGCGGTTTATGAACATAGGTAGGAGCAAATGGCTGAGCAAGCATTGAGCCTTGGAAATCTAAAGACAGAGAAATTGGACCACTCTTTTTACCAGCCCTGGCGGTGAACTATCACGTGGTGCAAATTCAAAGCAGCTTCAATTCTGCCGGGGCTTCTCCCGCCTTTTTCCCTAACGGCGGGAGAAGTAGATTGAAAGCCAGTTGGTCGATTATTTAGCTGTTAACTAAATTTTTGTGGAGTTCATAAAATCCCATCCAATCTAGGAAGGGCTTAGCTTAATTAAATTGATTTACGTTCAGTTGATGCAGAATAAAGTTTTGCAGTCCTTATTTGTTGTGCAGAAATTAAGTAAAATTTACTTACTAAGGGGCTTTGAAGGCTCTTGGTCTTATTAACCTAAATTTCTAAGTTATAAGTATTAGTGGAGTTAGGGGTAGGAGACAGGTAGAAGACACTACAAGTGGGGATAGAAACAGTATTGGCTTTATATAATTTAGTTGCCAGTTGATTTTGTGTTATTTGATGTTGGGAATATTGTCATTGAGATATGTATATTAAACGTATATAAAAATATAAATTTATTAATGTTAATAAGAAAACTAATATAAGAGAATAATTAGGTTATCATTTTTAATAAGTTCTTGGCATAATAATAGCTCATTTGGGGGAAGAAACCTGTTAGTGGGGTAGACCTCCTAGGGATATTATTATTAAGTGGAATAATGGGTATTGTTCATGTAGTTTGTTTCAGGTATGTGATAGGGATAGGGTTATTTACATTTGAGGTTAAATAAAGATTATTAATGTAGAGATTGTTAGGAAAATATAATAATTAAAGTTAATATAGTAATATTAGGGTCGTAACTCTAATACTGCTACTTATTCATCTAATATGTGAGTAATTGAAGAATAGGCTAGGATTTTGCGGAGTTGTGTTTGGTTGAGTCCTCCTCAGCTGCCAATTATAATTGATAGAACTGAGATTGTTAGAATCAAGTTTAGGGTTTGTTGACGGAAAAATTTGGAGGAGAATTGATATTGGGGCGAGTTTTTGTCACGTGAGAATAATTATAGCTGGATTAGAGGGATTCCTTGGGTAATTTCTGGGAGTCAGAAGTGAAGTAGGGCTAAAGCAATACGTAACTT

>f8896af6-4131-41b1-a29c-6de719db7153

GTGTACTTCGTTCGATTTACGTATTGTGAAAAAAATTATGATGAGTACGTGGGCGGTGACAATAACATTGTATACATGGTCGTCTTCTATTAGGCTTCCCGGGTTGTCCTAGTTCTGTTCGAATTAGGAAGCTTAGAGCTACCTACACTTTGCCATGCCAAATAATAAATATAATGTCCCAATGTCTTTGTGATTAATTGAAAATAGTCAGTTTTATGAACATGGGTAGGGGTAAAATGGCTTGAGCAAACATGAACTAACCAAATCTAAAGAGCAATACGTAA

>24c2cb69-6d13-4a6e-995b-e0db03b8501f

TTGTACTTCGTTCAGTTACGTATTGCTAGAAGTGGGAGGGTGGTAGAAGTCAGAAGCTTATATTGTTATTCGAGGAAATGCTATATCGGGAGCGCCAATTATTAAAGGAATAAGTCAGTTCCCAAAACCCCCAATCATAATTAGTATTACTATGAAAAATTATGATGAATGCGTGGGCAGTGACAATAACGGTAACGTATCATGGTCTCGTCTTTCTATTAGGCTTCGGGTTGTCTAGTTCTGTTCGAATTAGGAGGCTTAGGGCTGTACCTACTGCCCCCCGCCCGTACGCCAAATAATAAATATAATGTCCCAATGTCTTTGTGATTAGTTGAAAAATAGTCAGCGGTTTATGAACATAGGTAGGGGTAAAATGGCTGAACCG

>6985e5d3-bc0d-4484-8f05-f8b751a0649d

ATTACTTCGTTCAGTTACGTATTGCTGGTCAAGAGTATAGTACAGAAGAAGAGCGGGAATCAGTGAATAAATCCCCCTATAATGAGGCAAATACTGCTCCTGGTGATAACACATAGTGAAAGTGGGCTACTACATAGTATGTATCATGTAATACAATATCTAATGATGAGTTAGCTAACACAATTCCTGTTAGTCCACCCCACGGTAAAAGAAAAATAAAGCCCAGGGCTCATAATATTGCGGGAGATCATTTGATATTACCGCCGTGCAGTCATGGCTAATCAGCTAAATACTTTTACTCCAGTGGGAATAGCAATGATTATGGTAGCTGATGTAAAATATGCACGAGTATCTACATCTATTCCTACTGTGATTATGTGATGGGCTCATACAATAAAGCCTGAGAGCAATAGATATTATGGCTCATACCATGCCCATATAACCAAAGGGTTCTTTTTTATTAGAATAATATGTTACAATGTGGCGAAATTATTCCAAAACCCGGTAGATTAGAATATATACTTCAGGGTGACCGAAGAATCAGAATAAGTTATTGGTATAGGATAGGGTCGCCGCCACCAGCAGGATCAAAAAGTAATTATTTAGAATTGCGGTCAGTTAGTATGGTAATCCCTGCAGCTAGAACTGGGAAGAGAAAGTAAAAGAAGGACTGCAGTAATTGGGACGGATCACACAACAACGGAGTTTGATATTGAGTCATGGCTGGCGGTTTCATATTTACAATTGTTGTAATAAAGTTAATAGCTCCAAGAATAGAAGAAATGCCTGCTAAATGTAGTGAAAAAATGGTTAAATCTACAGAGGCTCCTGGGTGGGATAAATTGCCTGCTGGGGTGGGTAGACTGTCCAACCAGTGCCGGCACCGGCTTCTAAAGTTGATGATGCAAGCAGTAGGAGTAGGATCAGAAGTCAAACTTATGTTATTTATTCGAGGAAATGCTATATCGGGAGCGCCAATTATTAAAGGAATAAGTCAGTTCCCAAAACCCCCAATCATAATTGGTATTACTATAGAAAAAATTATGATGAATGCGTGGGCAGTGACAATAACATTGTATACATGGTCGTCTTCTATTAGGCTTCCGGATTGTCCTGGATTCTGTTCGAATTAGGAGGCTTAGGGCTGTACCTACTGCCCCGCCCATGCGCCAAATAATAAATATAATGTCCCAATGTCTTTGTGATTAGTTGAAAATAGTCAGCGGTTTATGAACATAGGTAGGGGTAAATGGCTGAGCAAGCATTAGACTGTAAAATCTAAAGACAGAAATTGAACCCTCTTTTACCAGCCCTGAGGCCGAACTATCACATTGAATTGCCAAATTCAAAAGAAACGCTTCAATTCTGCCGGGGCTTCTCCCGCCTTTTTTCCCTAACGGCGGGAGAAGTAGATGAAGCCAGTTGATTAGGTTATTTAGCTGTTAACTAAATTTTTGTGGGTTAAATCCCATCAATCTAGGAAGGAGCTTAGCTTAATTAAAGTAATTGATTTGCGTTCAGTTGATGCAGAATAAAGTTTTGCAGTCCTTATTTGTTGTGCAGAAATTAAGTAAAAGATTTTACTTACTAAGGGCTTTGAAGGCTCTTGGTCTTATTAACCTAAATTTCTAAGTTATAAGTATTAGTGGAGTTAGGGGTAGGAGATTAGTGGGGACACTTACAAAGTGGGGATAGAAACGGTATTGGCTTTATATAATTTAGTTGCCAGTTGATTTTTGTGTTATTTGATGTTGGGAATATTGTCATTGAGATATAATATATTAAACGTATATAAAAATATAAATTTATTAATGTTAGTAAAGCTATTATAAGGGGAATAATTAAGTTATCATTTTAATAAGTTCTTGTATAATATAGCTCATTTGGGGAAAAACCTGTTAGTGGGGGTAGACCTCCTAGGGATATTATTATTAGTGGAATAATGGGTATTGTTCATAGCAATACGTAACATTGTACTTCGTTCAGTTACGTATTGCTCCTGACAAGGATTACTACCAAGGACGGCAAGCGAGTGGCTACAGGTCCTGTTCAGGTCAAGGTGATCTACAGTAAGTTCAGGAATAAATTCATGGCAGATAATTTCCTTGAGCGCCACCGCAGATTACGAGCAACGCAAGGCTGCCTGGCTGAGACGCAGAAGCATCTTCCCCAAAGCCTGCCATTCAGCATCCTGATGATGAAGCTTTATAAAAAGATTCTATATGAAAAAATCCCAGCTGGAAGTTCTTTTCCAGCTGGGATTTTGATTATTTTTTCTATGAGAGAATGGTGAACTTGGCAAACTTCAGAAGCAACTGCTTGGTTCCTGCATTCTGGAACTCTACGGTAGCCTTCGTGTTCTCGCCGGTACCTTCAAGCAATACGTAACCCCA

>db8fe4dc-42b3-4a33-90dc-c28ab6cc7704

GATGTGCTTCGTTCAGTTACGTATTGCTCAGAGGCTCCTGGGTGGGATAAATTGCCTGCTAGGGGTGGGTAGACTGTCCAGCCAGTGCCGGCTTCAACTTCTAAAGTTGATGATGCAAGCAGTAGGAGTAGGAGAATTAGTGAAGTCAAACTTATGTTATTTATTCGAGGAAATGCTATATCAGGGCGCCAATTATTAAAAGGAATAAGTCAGTTCCCAAAACCCCCAATATAATTGGTATTACTATGAAAAAAATTATGATGAATGCGTGGGCGGTGACAATAACATTGTATACATGGTCGTCTTCTATTAGGCTTCCGGGTTGTCCTAGTTCTGTTCGAATTAGGGCTTAGGAGCTGTACCCGCCCGTACGCAAATAATAAATATAATGTCCCAATGTCTTTGTGATTAGTTAAAAAATAGTCGGTTTATGAACATAGGTGAGGGTAAAATGGCTGAGCAAGCATTAGACTGTAAATCTAAAGACAGAGGAGTGAACCCTCTTTTTACCAGCCTGAGGTGAACTATCACATTGAATTGCAAAATTCAAAGAAGCAGCTTCAATTCTGCGGGGCTTCTCCCGCCTTTTTTCTAACGGCAGGAAAGTAGATGAAAGCCAGTTGATTAGGTTATTTAA

>34713d0d-ef2f-4122-8567-bbf6c03cd5ba

AGTGTACTTCGTTCAGTTACGTATTTGCTATTACTATGAAAAAAATTATGATGATAGATACGTGGGCGGTGACAATAACATTGTATACATGGTCGTCTTCTATTAGGCTTCCGGGTTGTCCTAGTTCTGTTCGAATTAGGAGGCTTAGGGGCTGTACACCACTGCCCCCGCCCATGCATAAATAATAAATATAATGTCCCAATGTCTTTGTGATTAGTTGAAAATAGTCAGCGGTTTATGAACATAGGTAGGGGTAAAATGGCTGAGCAAGCATTAGACTGTAAATCTAAAGACAGAGGAGTGAACCCTCTTTTTACCAGCCCTGAGGTGAACTATCACATTGAATTGCAAATTCAAAGAAGCAGCTTCAATTCTGCCGGGGCTTCTCCCGCCTT

>c631b084-0226-41e4-97c0-043d533c151e

AATAACGTTCAGTTACGCTGTACTGGCACCGGCTTCTAAAGTTGATGATGCAAGCAGTAGGAGTAGGGAGGGTGGTAGAAGTCAGAAGCTTATGTTATTTATTCGAGGAAATGCTGCTATCGGGGCGCCAATTATTAAAGGAATAAGTCAGTTCCCAAACCACAATCTGGTGGTATTACTATGAAAAAATTATGGTGAATGCGTGGGCGGTGACAATAACATTGTATACATGGTCGTCTTCTATTAGTTCCCGGGTTGTCTAGTTCTGTTCCCGAATTAGGAGGCAGGGCTGTACCCTGCTGCCGCCCATGCGCCAAATAATAAATATAATGTCCCAATGTCTTTGTGATTAGTTGAAAATAGTCAGCAAGTTTTATGAACATAGGTAGGGAGTCAAATGGCTGAGCAAGCATTAGACTGTAAATCTAAAG

>7dc9546d-0a0b-4713-b4e8-1f73da2ef71f

GTACTTCGTTCGGTGCGTATTGCTGTAAGTCAGAAGCTTATGTTATTTATTCAGAGGAAATGCTATATCGGAGCGCCAATTGGACAAAGGAATAAGTCAGTTCCCAAAACCCCCAATCATAATTGATATTACTATGAAAAAATTATGATGAATGCGTGGGCGGTGACAATAACATTGTATACATGGTCGTCTTCTAATGTAGGCTTCCGGGTTATTCCACAGACTATTAATTTAGATGGGAAGCAGGAGCTGTACCTACTGCACGCCGCTACTTGAAATATTAAATATAATGTCCCAATGTCTTTGTGATTAGTTGAAAATAGTCAGCGGTTTATGAACATGGGTAGGGGTAAAATGGCTGAGCAAACGTGGAGCTGAAATCTAAAGACAGAGAAA

>1dde43b6-ee38-4c02-810c-7dc40b75eb5c

ACTTCGTTCAGTTACGTATTGTACCGGCTTCTAAAGTTGATGATGCAAACATTAGAGGTAGGGAAGGTGGTAAGTCGAAGCTTATGTTATTTATTCGAGAAGTATATATCAGGAGCGCCAATTATTAAAGGAATAAGTCAGTTCCCAAAACCCCCAATCATAATTGGTATTACTATGAAAAAATTATGATGAATGCGTGGGCAATTGACAATAACATTGTATACCATGGTCGTCTTCTATTAGGCTTCCGGGTTGTCCTAGTTCTGTTCGAATTAGGAGGCTTAGGGCTGTACCTACTGCCCCCCGCCCATGCGCCAAATAATAAATATAATGTCCCAATGTCTTTGTGATTAGTTGAAAATAGTCAGCGGTTTATGAACATAAAGGCTGAAAATTA

>1a27febd-865c-4897-8863-9421246b5ab7

TTGTACTTCGTTCCGGTTACGTATTGCTATTAGGCTTCCGGGTTGTCTAGTTCTGTTCGAATTGGGAGGCTTGGGAGCTGTGCCTGCTGCCCGCCCATGCGCCAAATAATAAATACTAATGTCCCAATGTCTTTGACGATTAGTTGAAAATAGTCAGCAGTTTATGAACATAGGTAGGGGTAAAATGGCTGAGCAAGCATTAGACTGTAAATCTAAAGACAGAGGAGTGAACCCTCTTTTTACCAGCCTGAGGTGAACTATCACATTGAATTGCAGAACC

>0ec7a030-3a3b-4cb4-8b63-8f29d133dea8

TTGTACTTCGTTCAGTTACATGTGCTTATTTATTCGAGGAAATGCTATCGGAGCGCCAATTATTAAAGGAATAAGTCCGGTTCCAAAACCCCAATGTAATTGGTATTATGAAAAAATTGATGGTCGTGAACCGGTGACAATAACATTGCTCTACATGGTCGTCTTCTATTAGGCTTCCGGGTTGTCCTGATTCTGTTCGAATTAGGAAGCAGGAGCTGTACCTACTGCCCCGCCAATGCGCCAAATAATAAATATAATGTCCCAATGTCTTTGTGATTAGTTGAAAATAGTCAGCGGTTTATGAACATAGGTAGGGGTAAAATGGCTGAGCAAGCATTAGACTGCAAATCTAAAGACAGAGAATTAGCCCTCTTTAAGCCCTGAGGTGAACTATCACATTGAATTGCAAATTCAAGAAGCAGCTTCAATTCTGCCGGGAGAAAGTTCTCCACGCCTTTTTTCCCCTAGAATGGTCCAAAGCCAGTTGATTAGGTTATTTAGCTGTTAACTAAATTTTTTGTGGGTTAAAATCCATCAATCTAGATGTACGTAACTTA

>9607be18-6d1e-4086-836f-cb5590198bb3

ATTGTACTTCGTTCAGTTACGTATTGCTCCTGGTCGTATAGAGGCTAGTATGGCTTGGTTTAAACGTCCAGGAATTGCATCTGTTTTTACGCCTAGTATTGATGGTACGGCTCATGAGTGTAAGACGTCTTGTGATGAACCTAATATGCGAATATCCACTTCTATAGGTAAGTTGTTCGGTTATCAACCGAGTCGAAATTCCTGGCTCAAAATATGTTGGCATAATGTGAGAGTCAAATACTAAGTCTTCATGGTCAGATATTCATAGGTTCAGTATCATTGGTGACCAATTGCTTTAAGGGTTAAATAAGGTTTGTTAAATTCGTCTGTCATATATAGAATACGCAGTGATGGGAGGGCAATTGTAATTAGAATCAGTGCAGGGGAGAATAGTTCAGATTATTTCGATCTCTTGAGCATTTATGGTGCTAGTATGAGTGAGTTTTGTGGTAGCAGTATAAGAAATAATATATAAGACTAAGGAACTAATCAGGAAAATAATTATGAGTGCATGGTCGTGGAAAGCAATAAGTTCTTCTATGATAGGTGATGTGGCATTTTGTAAACCTAGTTGAGCTGGTGTTGCTATTAAGATATATAGATATTTAGTCTATAGCGTTGCTTTGACAAAGTTATGTAATTATTTTACTAATATCTTATTAAGAAAAAGTCATAGGGTATATGGGATTGGCTGGAAACCAATTTTTGGGGTTCAAATCCTTCCTTTTTCGTCTAGGATTTAACGTAAGTTGCCTCTTCGAATGTGGTAAGGAGGGCAGCCGTATAATCATTCTAGATTGGTGGATAGTTGCTCAATGGCTAAGACTTTTCGTTTTGAAAGCTTCTCAAATTATAAAAACCATCCAGGACTACTGCTGTAAGTGAAATAAATGAACCGATAGATGGGACGATATTTCATGTAGTATATGCGTCTGGATAGTCTGAGTATCGTCGAGGTATTCCGGATAAGCCAAGGAAGTGTTGTGGGAAGAAAGTTAAATTTACGCCGACAAATATAATGGTGAAGTGGATTTTGGCATATGTTTGGTCAAGAGTATAGCCTGAAAAGAGCAGGAATCAGTGAATAAATCCCCCTATATTGGCAAATACTGCTCCTATTGATAACACATAGTGAAAGTGGGCTACTACATAGTATGTATCGCTATTATTACAATATCTAATGATGAGTTAGCTAACACAATTCCTGTTAGTCCACCCACGGTAAAGAAAAATAAAGCCCAGGGCTCATAATATTGCGGGAGATCATTTGTTATTACCGCCGTGCAGTGGCTAATCGGCTAAATACTTTTACTCCAGTGGGAATAGCAATGATTATGGTAGCTGATGTAAAATATGCACGAGTATCTACATCTATTCCTACTGTGAATATGTGATGGGCTCATACAATAAAGCCTAGAAACCAATAGATATTATGGCTCATACCATGCCCATATAACCAAAGGGTTCTTTTTTTATTAGAGCAATATGTTACAATGTGTGAAATTATTCCAAAACCCGGTAAGATTAGAATATATACTTCAGGGTGACCGAGAATCAGAATAAATGTTGGTATAGGATAGGGTCGCCGCCACCAGCAGGATCAAAGAAAGTGAGTAATTTAAATTACGATTAGAAGTTAATGGTAGGTAATCCCTGCAGCTAGAACTGGAAGAGAAAGTAAAGAAGGACTGCCGGTAATTAGGACGGATCACCTAAACAACGGAGTTTGATATTGAGTCATAGCTGGCGGTTCCTTATTTACGTTGTTGTAGGATTAATGGCTAGAATGAAGAGAAATGCCTGCTAAATGTAGTGAAAAAATGGTTAAATCTACAGAAATACCTGGGTGGGATAAATTGCCTGCTAGAGAATTGGGTAGGCGAACTGTTAACCAGTGCCGGCACCGGCTTCTAAAGTTGATGATGAAGCAGTAGGAGTAGGAGGGTGGTGAAGTCCCGAAGCTTATGTTATTTATTCGAAATGCTATATCAGGAGCACGCCAATTATTAAAGGAATAAGTCTCAGTTCCCCAAAACCCCCAATCATAATTGGTATTACTGAAAAATTATGATGGTATTAGATGACAATAACATTGTATACATGGTCGTCTTCTATTAGGCTTCAGGTTGTCTAAGTTCTGTTCGAATTAGGAGGCTTAGGGCTGTACCTACTGCCCCGCCCATGCGCCAAATAATAAATATAATGTCCCAATGTCTTTGGCGATTGGTTGAAAATAGTCAGCGGTTTATGAACATAGGTAGGGGTAAAATGGCTGAGCAAGCATTAGACTGTAAATCTAAAGACAGAGGAGTGAACCCTCTTTTACCAGCCCTGAAATTGAACTATCACATTGAATTGCAATTCAAAGAAGCAGCTATTCTGCCGGGCTTCTCCCGCCTTTTTTCCTAACGGCGGGAGAAGTAGATTTGAAACCAGTTGATTAGGTTGTTACAGCTGTTAACTAAATTTTTGTGGGTTAAATCCCATCAATCTAGGAAGGGCTTAGCTTAATTAAAGTAATTGGTTGCGTTTAAGTTGATGCAGAATAAAGTTTTGCAGTCACTTATTTGTTGTGCAGAAATTAAGTAAAATTTACTTACTATAAGGGCTTTGAAGGCTCTTGGTCTTATTAACTAAATTTCTAGAGTTATAAGTATTAGTGGAGTAGAAATTGTTGGAGACATTAGAAGACACTACAATTAGGGGATAAACAGTATTGGCTTTATATAATTTAGTTGCCAGTTGATTTTTGTGTTATTTGATGTTGGGAATATTGTCATTGAGATATAATATATTAAACGTATATAAAAATATAAATTTATTAATGTTAGTAAGCTATTATAAAGGGGAATAATTAAGTTATCATTTTTAATAAGTTCGCATATAATAGCTCATTTGGGAAAAAAACCTGTTAGTGGGGGGTGAACCCTCTAGGGATATTATTAGTGGAATAATGGGTAGTAATTAAATACTAGTTTGTTTCAGGTATGTGATAGGGATAGGGTTGTTACATTTGAGGTTAAATAAAAGATTATTAATGTAGGAATTATTATTAGAAAATATAAATAATTAAAGTTAATATAGTAATATTAGGGTCGTAATAATACTGCTATTATTCATCTATGTGAGTAATTGAAGAATAGGCTAGAGATTTTGATTATTGTTTGGTTGAATCCTCCTCAGCTGCCAATTATAATTGATAGAACTGAGATTGTTAGAATCAAGTTTAGGTTTGTTGACGGAAAAATTTGGAGGAGAAATTGATATTGGGGCGAGTTTTGTCACGTGAAAATAATTATAGCTGGGATTAAGGGATTCCTTGGGTAATTTCTGGGAGTCGAAGTGAAATTGGGGCTATCCCTATTTTTTGTGCTAGAGGCAATAATAGTATTATTGGATAATATTTGGTTATAAGGAGGATTGATTGTTCATTGTTGAGTTAAGTAGTTGTTAAGGAAAATGGATACTAATAGAAGTATTAGATGCTGTTGCTTGAATTAAAAAATATTTGGTGGATATACCTCTGTGGAGCGGGGATTGGTACTTTTGGCAAGAGTTGGTACGATGTAGCTAGTATATTTAATTCTAAGCCTATTCAGGCTAGGAATCAATGTGAGCTTAAAATTGTGATTACGGTCCCTGTTAGAATGGTGAAGGAGATAATAAAGGTGGGCTGAAGTTAATGTTAGCACAGGAAGGATTAAACCAACATTTTCGGGGTATGGGAGCAATACGTAA

>e955b410-1965-436e-9d07-c38d832194fc

GATGTACTTCGTTCAGTTACGTATTGCTCCCAATCATAATTGGTATTACTATGAAAAATTGATGAAGATGCGTGGGCGGTGACAATAACATTGTATACATGGTCGTCTTCTATTAGGCTTCCGGGTTGTCCCTAGTTCTGTTCCGAATTAGGAAGCAGGGGCTGTACCTACTGCCCCCGCCCATGCGCCAAATAATAAATATAATGTCCCAATGTCTTTGTGATTAGTTGAAAATAGTCAGCGGTTTATGAACATAGGTAGGGGTAAAATGGCTGAGCAAGCATTAGACTGTAAATCTAAAGACGAGGTGAACCCTCTTTTTACCAGCCCTGAGGTGAACTATCACATTGAATTGCAAATTCAAAGAAGCAGCTTCA

>bb3f2178-3825-4a48-9edf-72ee6a1e7215

AGTGTACTTCGTTCAGTTACGTATTATAATCATAATTGGTATTACTATGAAAAAAATTATGATGAATGCGTGGAGGCGGTGACAATAACATTATATACATGGTCGTCTTCTATTAGGCTTCCGGGTTGTCCTAGTTCTGTTCGAATTAGGAGGCTTAGGGCTGTACCTACCTGCCCCCGCCCGTACGCCAAATAATAAATATAATGTCCCAATATCTTTGTGATTAGTTGAAAATAGTCGGTTTATGAACATAGGTAGGGGTAAAATGGCTGAGCAAGCATTAGACTGTAAATCTAAAGACAGAGAGTGGGCCTCTTTTTACCGATACAAAGGTGAACTATCACATTGAATTGCAAATTCAAAGAAAC

>511fbe57-0ed6-4c5c-8d62-bf340b06b5d6

AGTACATGCTTCGTTTCAGTTACGTATTGCTCTTCTAAAGTTGATGAGTGCAAGCAGTAGGAGTAGGGAGGGTGGTAAGTAGGAAACATGTTATTTATTCCGAGGAAATGCTATATCAGGAGCGCCAATTATTAAAGAGATAAGTCAGTTCCCAAAACCCCCAATCATAATTGGTATTACTATGAAAAAATTATGATGAATGCGTGAGCAGTGGATATTAACATTGTATACATGGTCGTCTTCTATTAGGCTTCCGGGTTGTCCTAGTTCTGTTCGAATTTAGGAGGCTTAGGGCTGGCACTACTGCCCCCGCCCATGCGCCAAATAATAAATATAATGTCCCAATGTCTTTGTGATTGGTTGAAAATAGTCAGCGGTTTTATGAACATAGGTAGAGGGGTAAAATGGCTGAGCAAGCATTAGACTGTAAATCTAAAGACAGAGTGAACCCTCT

>5b72c942-6fb5-457e-a3b5-0ece423f2e53

ATTACTTCGTTCAGTTACGTATTGCTAATAGAATAAATTCTTTGTTGCTCAGGGGTAGTTTAATACTAGAATATATCAGCTTTGGGTGTTGATGGTAGAATTGATTTGTTCTTCCCCTGAATTGCCCAGGGAGTAGATCTGTCTCCATTTCTGGTTTCAAGACCAGAGTATTAATTTATACTACAAGGACTTTATCATTTAAGTATTTATTTTCGATTAAGGAAGAGGGGGATTAAGGTGATGATAATTAGAAAATATATAATGGAGGCTGTTTGGCCAATGGTGATAAATGGATATTCTACTGGTTGCCACTCAATTCATGTTGGTGTAGTAAATCAGCTATAGGGTTCAGAATAGGATTTGGGAAATAGGTCGGAATTTTATATGCTTTGTTGTTTGGATAAATGTGTTATGGGAATAATTATTAGAATTAGGATAAGAAAGTATAAGTGCTAGAACTCCTCCCAGTTTAGTAGGGATAGATCGTAGGATGGCATATGCAAATAGGAAGTATCATTCTGGTTTAATGTGGGAGGTGTTTAGGGGGTTGGCTAGTGTGTGGTGTCTGGGTCTGTTAGAGGTCAGGTAAAAATAGTGTTAGGCTTATTAGGAATAAGAGAGAAGAAAAAATTAGTCCAAGAATGTCTTTGGTTGTATAATATGGGTGAAGTTTTGTCGGGTTCTGAGGCTATTCCTGATGGGTTACTTGAACCTGTTTCATACAGAAAGTTGAATTGTTGCTAAGGCTGCGATAATGAAAGGTAAAATAAAGTGAAAGGTAAATCGTGTTAGGGTGCTTTATCTACTGAGAATCCGCCTCGAATTCGTGCACTAAGTCGGAGCCGATGTAGAGAGATGGCTGATAAGAGGTTTGTAATTACTGTAGCCCCTCAGAATGATATTTGGCCCCATGGAAGTACATAACCTATAAATGCCGTGGCCATAGTTGCTAATAGTAGGATTGTACCCATTTCAGAATCTTCAGAAAAGAAAGATCCGTAATATAGGCCTCGTCCAATGTGGAGGAAAAGGCAGATGAAAAATATGGATGCACCGTTGGCGTGTAAATAACGAATTATTCATCCGTAATTAACATCTCGAGGTAATGTGGGCTACTGAGGAGAAAGCGGTGGAAGTGTCTGGTGTGTGAGTGTGTTGCTAGAAATAGGCCTGTGGTGATTTGAATAATTAGACAGGTGCCTAAAAGTGAACCGAAATTTCATCAGGATGAGATATTGGATGGTGTGGGAAGGTCAATAAATGATTCGTTGTGATACGTTTTGCTAGTGGGTGGGTTTTGCGAGGGAGGTCATTATTATTCTTATAGTTGAAACACAACAATAGTTTTTCATATCATTAGTCATGGATTTTACATAGTCCATGTAGGAATAATGACATATGCTTTATTTTCATTAAGTATTATTTTGGTAATAGGTTTTGTGGGGTTTATATTTCTAAACCTTCTCCTATTTACGGCGGTTGGTGTTAATTTTTAGTGGTGCTGTGGGTTTGTGCGATTACGTTATATTTTGGTGGGTCTTATATGGGGCTTATGGTTTTTTAATTTATCTAGGAGGTATAATGGTTGTATTTGGTTATACTGCGGCTATGCACAATTGATAGACATCCTGAAACCAATTTCAAGTGTTGATATTTTAGAATTTTGTATTGGGGTTTTGTATGATGGAAATAATGATAGTATTATTATTGGGTGGGGATCCTGTTAAAACGGTGGAAATTACAGTCAATTATAAGACTGTGGCAAGTTGAATAATTTGTAAGGAGAAGGACCTGGGTTGAATTCGTGAGGATCCTACAGGTGCTGCGGCTTTATATAATTATGGTGTTTGAGTTGTTGTAGTTACTTGTTGGGCTCTGTTTATAGGTATGCATATTGCTATTGAGCTCACTCGAGGTGGTGATTTAAATAGTTAAAAGTAGGGCTAGAGTTGGTGGAATGAAGAAGGATAAGAAATACAATTTAATCAGGCCTTTTGAGTTGTGGAAGTTGTAGATATTGAGATTTGAGTTTGTATTGTTATTTTTGGTATAGATTTTCTAGTCAGAATAGGTCTAGTAGAGTTGAAGTAATGTTTTGGCCCATGTTTAGGCTTGAATGGGGTTGAGTCGGTGTGTAGTAATAGTGTAAAAACCTAGTATGTTAGAAAAGTAGAAAGTTTTTAATGGGGTGCTTAATTTTATATTGTTAGTTATTAGACTAAGTTCTGTTGCTATTATAGGTCTAGGACTGATTTGCAACAGCTGTTAGTTTGAGGTAGTATGTGATGATATAGTGACTTAGGGAGTGAGTATAGCAACACAATTGAAATAAGAAACCCGGCGAAAATGCTATGTTGCTGCGGCATTAATGGAATTTGTGGTATAGGGTTATTTTCGTTGATTGGTGTTAGAGTTATGAAACGTGGGTGCCCTGTTATGGTAAAGTAAATAGCCACGAATACTATATATAGCTGTAATAAGTACTTCCCGGGTGGTTGTGAGTGCTCAGGCGTTGGTATACGACACGTGTTGGCGATTTCGATGATTAGGTCTTTTGAGTAAAAGCCTGTGAGGAAGGGTATACCTATAAGTGCGAAACTGCCAATGACAAGTGAGGAGGATGTGAATGGGAGTATTTTAAATAGTCCACCTATTTTGCGGATGTCCTGCTCGTTGTTTAGGCTGAATAATGGACCCTGCGGATAGAAATAATATGGCTTTGAAGAAGGCGTGAGTGCAGATATGGAGGAAGGGCTAGGGCAATGGTTGGTTAATACCAATTGTCACTATTATAAGGCCTAGTTGGCTTGAGGTAGAAAAAGGCTACAATCTTTTTTAAGTCATTTGTGTTAGGGCACAAATTGCTGTAAATAAATTGGTGATTGCGCCAAGTGATAGCGCTATTGTTTTTGGATAAATTGATTATTTTCTATTAAAGGATAAAAACGGATAATTAAAAGATTCCTGCAGCAACTATTATGTAGAGTGCAGTAGTGCTGACACTGGGGTGGGCCCTTCTATAGCGGAGGGTAATCACGGATGTAGACCAAATTCGACAGATTTTCTGTTGCTGCTAAAAGTAAGCTTGTTAAGGGAAAAGAGTTAGGAGTATAATCTAGTATAAATATTTGTTGAAAGTCTCATGAGTTATGATATAAAAAGAACCACGTTATTGCTAAAATAAAACGATATCTCAGTCCGGTTATATAAAGTACCTGCAGGGCTGCTGTGTTGGCATCTGTTCGTCCGTACCATCAGCTAATTAATAGAAATGATATAATACCTATCCCATCCGATAAAAAGTTGAAATAGATTGTTTGCAGTAATTAGAATTAATATTCAGTGGAAAATGAGAAGATATTTAAGGAATTGGTTAATGTTTGGGTCTGAGAACATACCATGTTGAGAATTCTACAATTGATCAGGTGACAAAGTACGGGGCAAATATTGTGGAGAAAAATCTATTTAAAGCTGAGTGATAACTTGATAGTTTGAATAGTAGTTCAGTGTCAGTTTGAAATCATCAATTACTGACCTGTAAAGATGTATATTGCTATACATAGGATGCTGGCAGTGAGGGCATAAATAATAGCTAGTTTTACGTAATAAGGATATAGGAGGCTTTTATGTAAATTAACTATAGTTATTAAGATTGGTACTAGTAGTGGAATAACGGTGAATATAATTATTGAGGAGTATATTTTTACTTTTATTTGGAGTTGCACCAATATTTTTGGTTCCTAAGACCAATGGATAACTTTACTATCCTTTAAAGTTGGGGGAAAGTAAAGTTGTTAGGCCTGGTAGCATGAATTAGCAGTTCTTGCATACTTTCTCGGTAGTTAAGTTATGGAGCTTCTATCATTAAATTCACAATCTAATGTTTTATTAAACTATAACTACAGGGCGTCAAACCTATAATCCAGGGTTTGTAGTTAATAGTAGGATGGGTATTAAATGTATTATTATTAGTACATTTCTCGTGTAAGAGGGGTTTGATGCTGCTAGTGCTGTACGTTAATGGCCCTCGTTGTGTTGAGGTAAATATGTGAAGTGAGTAGAGGGCTGTGTTAACATGTTGAAACCTGTAAATATGATGGTAAAATTAGATCAGAGAAAGAAGCAAGATTGTTAATGGTTCACCTGGCCCTGGGTTAATAGTTGGGGGGGGAGAGCTAAATTTGCTAGGTTAGCTAGGAGTCATCAAAGTGCTAGAAGGGGAAATAATGTTTGAAGGCCTCATTAATAGCGTGTATAGTTCGGCTGTGAATTCGTTCGTAGTTAGTATTTGCTAAACAAAATAGCAGGGATGAGTAAGTCCATGGGAGATTATTGAGTATTATTGCACCGGTAAAGGCTTCATGGGGTTTAGATGTAATGGCTAAAATAACAAGTGCTATGTGGCTGACGGAGGGGTAAGCGATAAGTGATTTTAGGTCGGCTTGTCGTAAGCAGATGGAGCTTGTTATCACTATCCCTCATAGGGATAGAATGATAAAAAGGTAGCTTATTTTTCTGTTAGTGGATTGAGGATAGGAGTAATTCGCACTATACCATACCACTCCTAGTTTTTAGTAAGATTGCTGCAAGTACTATTGAGCCAGCGATGGGGGCTTCGTGAGCTTTTGGGAGTCATAGGTGTAACTTATATGAAATTATTTTGACTATAAAAAAGCTATTATACATCCCAATCATATAATACTATTTGGTTCATGAAGTAAGTATTTCGTTAGAATTAATAATTATTGTAAGTATGCTTAGGGATCCCAAAAGTATTTAGATAGTATAGGAGGGTAATTAATAGAGGTAAGGATCCTGCTAGTGTGTAGAATTAGAAAATATGAACCAGCATTAAGGCATTTAATTGATACCCTCAACAGGTAATAATAATTAGGGTGGGGATTAGGGTAGTTTCAAATAGAATATAAAATAAAGATCGTTCAGTAGCTGAAAAGTTATAATAAGTGAAATTTGTAATGGGAATATTGAGATATAGTTTTTTCGTGGGGTGGGGTTATTATATAAATGCTGTTGTGTTGCTAGAATTATTAGTGGCAGAAGTCAGGCTGTTAATATAAAGTGGTGATGTTAGTGAATCTGAGGAAAAAATCAGTGATAGGTTGCATGAGTTGTTTGGTGTGGAGTAATATGAGGGTGTGTATACTGAGTTAGCTGACTTCACAGATTGTGTGTTGATTCATATTATATAATTTTTGAAAGGTATGGTGAAATGTGTGCATGATGGTTGGAAAAATAAATTTTAACATTGGAGTAAGTTTAGGTTTTGTACATAATCTAGGCCATATAGGTTGGAGACTAAAATTAGCAGGGCTAGGCCTACTGCAGCTTCGCATGCGGCAAATACTAGGAGAATGAGGGTATCATATACATTAACTTATGGTGCATATTGAAAGTTGTGAGTATGAAATTATGATAAATAATGATAATATTATGCCTTCCAAACATAGCAGTGATGATATTAGGTGAGATCGATAAATTAATAACCCTAATAATGATATAGGAAATATGCTAGTATTACATTAATATAGATAAAAGCATATTTGGTAAATATGAGCTTTCATAATCTAATGAGTCGAAATCATTTGTTTTAATTAAACTATATACCAATTGGCCCAATCTAATCCTTTTTGGGACCACTCGTAGGTAACCCTAAAGCCAGAATAATAAGTAGGGTAAAAATTTAATATTTATTGTTAGGATTAAATTATTTGTTTGGGTTACCCATGGTAAGAAGAGTAGGGCAATTTCCAGATCAAATAGGAGAAATGTGATTGGCGATTAGGAAAAATTTTTATGGAAGAATGTTGATGCTGGAGGATTGTAGGATCAAATCCGCACTCATGGGGTGTGCTTTTCTGTAAATATTTAGTGTGGAAGTCAAAATGTAATTGTGATAAGAAGTAAGGCCAAAGTAATGTTAGTCATTAGGGCTAAAATTAAATTTATAATACTCTCTCTCGAATCATTCGAGGCCCGTTGATTGGAAGTCAATAATACTTTTTATACTAAGAGAGTAGAGCCTCATCAATAGATGGAAATATAGAGGAAAAGTCATGCACATCTACGAAATGTCAATATCATGCAGCGGCTTCGAAGCCGAAATGGGTAGCTAGATGTGAAGTGATATAATTGTTGGCGAAAGCAACGAATTGAAGGAAAGTGGTTCCGATAATTACGTGAAGCCATGAAAGCCTGTAGCCATAAAAAATGTGGATCCATATACTCCGTCGGGAGATAGTAAATGGGGCTTCAGAATATTCTGATAATTGTAGGCAGGTGAAATGATCCTAATATAATGGTTAGGGTAGTGCTTGGTTGATTCTTTCGGTTAGCTTCTATGAGGCTGTGATGTGCTCGCTCGTAATTGTAACTCCTGATGCTAGTAGTACGGTTGTATTTAAGAGGAGGACTTCTATTGGGTTGAGGGAGTAATGCCTGTAGGTGGTCGCTAGTCCCCCTGTTTGTGGAGTTGGGCTAGACTAGAATGATAAAATGCTCAGAAACCTGCAAAGAGAAAGAAAATTTCTGAAATAATAAATAGAATTATTCCGTATCGTAGGCCTTTTTGGACGGAATTTTTTATTATAGTGGCACTTGATATGCTTACTTTCTCGTACTACATCACGTCATCACATTGAAATATTGTTATAGAACTAGCTAGTAGACCTGCAGTGAGAAGCAGTGTGGTAAAAGTGGAATCATATAACTAGGCCGGATGTGAGAAGAAAATAGCTGATAATGCTCCTGTTAGTGGTCAAGGGCTTGGTTGACTATGTGATAATGGGTTTGGTGAGTGGTATGAATTATCATGTAATTACAGGCTTACTAAGAGGGTAAATACATAGGCTTGAATTAAGGCTACACCCAACTCTAGGGTAATTAATAAAATAATAATAATGATGATGATTGTGGAAGTGGAGAGGTAAATTGATAGAGTTAATGTTGTATCCCCAAGTAAATGCATTAATAGGTGACCTGCTGCGATATTGGCTGTTAATCGTACGGCTAGGCTACAGGTTGAATGAATAGACTAATTGTTTCAATAATAATTAATATGGGATTAGTGGAATTGGTGTTCCTTGTGGTAAAAAATAGACGAGGGATGATTTTGTTTTAAATCGGAGGCCTATTAGTACAGTTACTGCTCATAGAGGAATAGCTATGCCTAGATTTATTGACAGTTGGGTAGTTGGTGTAAATGCATATGGTGTAAGTCCGAGAATGTTATTTAGGGCAATGAGAAATTAGGGCTAGGAGTATAAGGGATCAGGTTCGCCCTTTAGTGGTGTGGGTTAATATTATTTGTTTAATTGTTAGTTGAATTAGCCATTGTTGAATTGAAGAGAGTCGGTTGTTGAATAAGTTTTTGGAGGATAAAATTAATGTGAAGTGGGAGTGCAATAGTTAAAAATACTAGGGTACTCTAGTATTGTGCTAGAATATTGAATGAGGCAAATGATTAGTTCATTTTAGTTCTCAAGTTGTTTTATGTTTGTGTTTCTACTAATTTTTTGGTAGTGGGTAATAATGGAAAGTAAAGTTCAGTATTTTCCATTGTATAATATAAAATAAGGTAACAATTATAGATATAATTACTATTGGTCACAGTGAAATATTTAGTTGAGGCATTCACTATAGAAATTTGTTCTCTCAATCTTTAACTTAAAAGTTAAATACTAGTTAGCTACAGTGATACAATATATAAGTATGAAGCTCATACTTCGAAATCTTGGAAATAAATGAATTTAAAGACAATAGGTATAAAGCTGTGATTGGACCCGCAAATTTCCGAGCATTGTCCATAAAATAGGCCTAAAATCGTATAGAGGCTAGTATGGCTTGGTTTAAACGTCCAGGAATTGCATCTGTTTTTACGCCTAGTGATGGTACGGCTCATGGTGTAAGACGTCTTGTGATGAAGGGATTATTATGCGAATATGCTTCTATAGGTAAAGTTGTTCGGTTATCAACTACGAGTCGAAATTCCCCAATCAAGGAAATATGTTGGCATAAATGTAAGAGTCAAGTACTAAGTCTTCATAGTCAGAATATTCATAGGTTCAGTATCATTGTTGACCAATTACTTTGAAGGACAAAGTTCTGTACTTAAATTCGTCTGTCATATATAGAATACGCAGTGATGGGAGGTCAATTGTAATTAGAATCAGTGCAGGGAGAATAGTTCAGATTATTTCGATCTCTTGAGCATTTATGGTGCTAGTATGAGTGAGTTTTGTGGTAAGTATAAGGGAAATAATATATAAGACTAAGGAACTAATCAGGAAAATAATTATGAGTGCATGGTCGTGGAAAGCAATAAGTTCTTCTATGATAGGTGATGTGGCATTTTTGTAAACCTGGTGCAATTTCAAGGTGTTGCTATTAAGATATATAGATATTTAGTCTATAATTTAACTTTGACAAAGTTATGTAATTATTTACTAATATCTTATTGAAAAGTCATAGGGTATATGGGATTGGCTTGGAAACCAATTTTGGGGGGTTCAAATCCTTCCTTTTTCGTCACAAGTTAACGTAAGTTGCCTCTTCGGGCAATATTGGTAAGGAGGAGGGCAGCCGTATAATCATTCTAGATTGGTGGATAGTTTAACAATGGCTAAGACTTTTCGTTTGAAAGCTTCTCAAATTATAAAAACCATCAGGACTACTGCTGTAGTGAAATAAATGAACAAGATAGATGAGACGATATTTCATGTGGCCTTATGCGTCTGGATAGTCTGAGTATCGTCGAGTATTCCGGATAAGCCAAGGAAGTGTTGGGAAGAAAGTTAAATTTACGCCGACAAATATAATGGTGAAATTAGTTTTGGCATATGTTTGGTCAAGTATAGCCTGAAAAGAGCAGGGAATCAGTGAATAAATCCCTATATTGGCAAATACTGCTCCTATTGATAACACATAGTGAAAGTGGACTACTACATAGTATGTATCATACCAATACAATATCTAATGATGAGTTGGCCAACTACGATTCACTGTTAGATCACCCACGGTAAAGAAAAATAAAACCAGGGCTCTAATATTACAGAGGAGATCATTTGATATTACCGCCGTGCAGTGTAATAATCAGCTAAATACTTTTACTCCAGTGGAATAACCAATGATTATGAGCAGCTGATGTAAATATGCACGAGTATCTACATCTATTCCTGCTGTGAATATGTGATGAGCTCATACAATAAAGCCTAGGAAACCAATAGATATTATGGCTCATACCATGCCCATATAACCAAAGGGTTCTTTTTTATTAGAGCGTAATATGTTACAATGTGTGAAATTATTCCAAAACCCGGTAAAGATTAGAATATATACTTCAGGGTGACCGAAGAATCAGAATAAGTGTTATAGGATAGGGTCGCCGCCACCAGCAGGATCAAAGTAGTATTTAAATTACAGTCAGTTAATGAGTATGGTAATCTGCAGCTAGAACTGGAAGAAAGTAAAGAAGGACTCACCGTGAAACGGACGGATCCTGAAACAACGGAGTTTGATATTGAGTCATGGCTGGCGGTTTCATATTTACAATTGTTGGCAATAAAGTTAATAGCTCCAGAATAGAAGAAATGCCTGCTAAATGTAGTGAAAAAATGGTTAAATCTACAGAGGCTCCTGGGTGGGATAAATTGCCTGCTAGGGGTGGGTAGACTGTCCAACCAGTGCCGGCACCGGCTTCAAGTTGATGATGCAAGCAGTAGGAGTAGGGAGGGTGGTAGAAGTCAGAAGCTTGTATTTATTTATTCGAGGGAAATTACTTTATATCAAGACTTAATTATTAAGGAATAAGTCAGTTCCCAAAACCCCCAATCATAATTGGTATTACTATGAAAAAATTATGATGAATGCGTGGGCGGTGACAATAACGTATACATGGTCGTCTTCTATTAGGCTTCGGGTTGTCCTAGTTCTGTTCGAATTAGGAGGCTTAGGGCTGTACCTACTGCCCCGCCCATGCGCCAAATAATAAATATAATGTCCCAATGTCTTTGTGATTAGTTGAAAATAGTCAGCGGTTTTTATGAACATGGTAGGGGGTAAAATGGCTGAGCAAGCATTAGACTGTAAATCTAAAGACAGAGGTGAACCCTCTTTTTTACCAGCCCTGAAGTGAACTGTCGCATTGAATTGCAAATTCAAAAGAAGCAGCTTCGAATTCTGCCGGGGCTTCTCCGCCTTTTTTCCCTAACGGCTTGGGAGAAGTAGATTGAAAGCAGTTGATTAGGTTATTTAGCTGTTAACTAAATTTTGGCCGGGTTAAAATCCCATCAATCTAGGAAGAGCTTAGCTTAATTAAAAATTGGTTGCGTTCAGTTGATGCAGAGTGGTGCAGTCCTTATTTGTTATTAGAAATTAAGTAAAATTTACTTACTATAAGGGCTTTGAAGGCTCTTGGTCTTATTAACCTAAATTTCTAAGTTATAAGTATTAGTGGGTGGGGGTAGGAGACGGTGAAGACACTACAAGTGGGGATAGAAACGGTATTGGCTTTATATAATTTAGTTGCCAGTTAGTTTTGTGTTATTTGATGTTGGGAATATTGTCATTGAGATATAATATATTAAACGTATATAAAAATATAAATTTATTAATGTTAGTAAAGCTATTATAAGAGGGAATAATTAAGTTATCATTTTTAATAAGTTCTTGTATAATAGCTCATTTGGGGGAAAAACCTGTTAGTGGGGGTAGACCTCCTAGGATATTATTATTAGTGGAATAATGGGTATTGTTCAGCGCTAGTTTGTTTCTTTTAGGTATGTGATAGGGATAGGGTTGTTACATTTGAGGTTAAATAAAAGATTATTAATGTAGAGATTGTTAGGAAAATATAAATAATTAAAGTTAATAGTAATATTAGGGTCGTAATATAATACTGCTATTCATCCTATGTGAGTAATTGAAGAATAGGCTAGGATTTTGCGGAGTTGTGTTTGGTTGAGTCACCTCGCTTATGATTATAATTGATAGAACTGAGATTGTTAGAATCAAGTTTAGGTTTGTTGACGGAAAATTTTGGAGGAGAATTGATATTGGGGCGAGTTTTTGTCACGTGAGAATAATTATAGCTGGGATTAGAGGGATTCCTTGGGTAATTTCTGGGGAGTCAAGTGAAGTGGGGCTATCCCTATTTTTATTGCTAGAGCAATAAATATTATTGTGGATAATATTTGGTTATAAGGAGGATTTGATTGTTCATTGTTGAGTTAGTAGATTGTTAAGGAAAATGGATACTTATGCAGTATTGATGCTGTTGCTTGAATTAAAAAATATTTGGTGGATGCCTCTGTGGAGCGGGAGTTGGTACTTTTGGCAAGTTGGTGATGGCTAGTGATATTTAATTCTAAGCCTATTCAGGCTAGGAATCAATGTGAGCTTAAAATTGTGATTACGGTCCCTGTTAGAATGGTGAAGGAGATAGAAAAGGTGGGCTAGAGGATTAATGTTAGCACGGGAAGAGATTAAACCAACATGCCAAGGTGTATGGGCCCGATATTTATTTAGCTGACCTTACTGTTTAAGGATATGGTATGATGTTGCTTTTGGAGAGTTTTGAGTTCTCAGGTGTAGGTTCAATTCCTGTAATCCTAGAAATAAGAGGGTTTTGAACCTCTATAATTTACTCTATCAAAGTAACTCTTTTATCAGACATATTTCTATGTTTGGGGTGGGATACCAGATGCTAGGATGGGTATTGAAACATATCATATACATAGTGCTAATGTAAGTGGTAAAAAGTTTTCATAGTAAGAATATTAATTGATCGTAGCAGGCGGGGGTAGGCTGTTCAGATTCATAAAAATAAAGTGGTTAGTAGGAGGGTTTTGGCTATAAAATTAATAGTAAATGTTTCTGGTATAATTATTGTAGGGTGTGGATAAGAAAATAGTGGTAGTTAGGGCATTTATTATAATAATATTTATATACTCTGCTATGAAGAATAAGGCGAATGAGCCTGCAGCGTATTCAATGTTGAAACCTGATACTAGTTCTGATTCGCCTTCTGTCAGGTCGAATGGAGCTCGATTAGTTTACCGCTAATGTGGAAATAAATCATATTATGGCTAGGGGCCATGATGGAAGTAGAAGTCAGTAGTGTTCTTGAGTGGTAATAAGCGATTGCTTGTAGATTGAATGAGCCGCTTATTAGTAGTGTTGATAGTAAGATAATGGCAAGGGTGACCTCATGTAAGTTGTTTGGGCTACAGCTCGTAGTGCGCCAATTAGTGCGTAGTTTGAGTTGGATGCTCAGCCAGATCATAAAATTGAGTAAACAGCTAGGCCTTGATGTTGCGAGGATAAATAGGAGACCAAGATTGAAATTTGATAAGAGGATATGGTATGGGAGTAGCGTTCTGGTAGAGCAATGGAAAGAGCTAGGGTGGGGGCAGTTAAATATAAAGTTATGGTGGATGTGGTAGGTAATAAGGGTTCTTTTTTTAGCGAAGAGTTTTATGGCGTCAGCGATTGGATTGAAGTATTCCGTAGGGCCTACAATATGGGGCCTTTCGGGTGTATATAGCCAAGTTTTTTCGTTCTGTGAGTGTTAGAAAGGCTATGGCAATTGGGGCAGGTAGGACTAGTATTGGTAAGTTAATTTATATACATGTTGTTAGAAATTGAACACCTCTGATTGTCAGTTTAAGTTTTATGCAATTACCGGGCTCTGCCACCTTAACGAGCCCTGTTCTTGGGCAAAGATTATAATATGTGAGGTTGAGATGGAGGTCATCTGATTGTTGAGGGCGGCGCTTTATAGTGGGCCCCATTTCTCATTGTCCTTTCGTACTGGGAGAAATATTTAGAATAGATAGAAACCGACCTGGATTGCTCCGGTCTGAACTCAGATCACGTAAGACTTTAATCGTTGAACAAACGAACCATAATAGCTTCTGCACCATTAGGATGTCTTGATCCAACATCCCCAGGTCGTAAGCCCTATTATCGATATGGACTCTAAATGGGATTGCGCTGTTATCCCTAAGTGCAACTTGGTCCGTTGATCAAATTATTATTGGGTCGCCGTTGCTTGCTTACTTAGACTGGTAAGGTCTTGGTATATGTTTTTCGGAGGTTTCTATTTACTCCGAGTCGCCCCAACCGAAATTTATAATACATTGACGGTAAATTAATGCCTGTTGGTTTTTACAAATCTAGTTTGTATCATTAAATTAAAAGCTCCACAGGGTCTTCTCGTCTTATTTATGTATATCCGCCTCTCTTCACGGATAGGTCAATTTCACTGATTAAAGAAGTAAGAGACAGTTAAACCACGTCGTGTGGCCATTCATACAAGTCCCTATTTGAGAACGAAGTGATTATGCTACCTTTGCACGGTCAGGGTACGCGGCCCGTTGAACATATGTCACTGGGCAGGCGGTGCCTCTAATGCCCTAGAAATGCTAGAGGTGATGTTTTTGGTAAACAGGCGGGGTTAGAGCTTGCCGAGTTCCTTTTACTTTTTTTAATCTTTCCTTAGGTGCATGCCTGTGTTGGGTTAACAGTTTAGTTAATTGATATTTTAATTTGTAGTTTATGATTATTAGGCTGTTAACTAACAGTAGTTGTTTCGGTCTGAAATAAGCTTGGCCAGGAGAATAGATTCATGTTACTTATGTAACATTATTGCTTCTATTAGATAATAGATTAATCCAATTTGTTTCAGGAGTTGATAAAATTAGTAGAATTAAATATATGAGAATATTGACTTGAACGCTTTCTTAATTGATGGCTGGCATTTTAGGCCAACTATGGGATTTATACTACTTACTCTCTATTAAAGGTTTTTTCCTAGGGTCTAAAGAGCTGTCCCTCTTTAGACTAACAGTTAAATTTACATGAGGATTTAATAATTCTGGGGATAAATTTAAAGTCGAACTAAGATTCTGTCTTGGATAACCAGCTATCACCAAGCTCGTTAGGTTTGTCATACTACCCATAGATCTTCCCACTATTTTGCCACATAGACGGGTGTGCTCAAAGCTGTCCTTATGGGTAGCTCGCTTGGTTTCGGGGTACTTTGTTGAAGTTCTCTTTATAAAACTACTTCTAGTTAATTCATTATGCAGAAGGTATAAGGACTCGTCTTTGCTTTTATATGCTTATTAGGTAATATGCTTTCCTTACGGTACTATATCTATTGCTACTTACGATATGCTCAATTTCTATCGCCTATACTCTTACTATAGGTAAATGATTTAATTAATATAAATGAATAATATATTTAATGGAGAGTTGGGCTAAGTTGCTCAAAATGATCATTTATTTACTTGTCTTTCGGGTGTAAGAAGCCGGATGCTTTATTTTAAGCTACATTTTGGTTTGTCCAAGCGCACTCCAGTACGCTTACCATGTTACAACTTATCCCCTCTATACCAGTTTTTTAGTATTTTATTGTTAATGTACTTTTGTGTGGTGTTTGAGGAGGGTGACGGGCGGTGTGTGCGTGCTTAATGGCCTTGTTTCAATCAAGCTCTCTATTCTTGGTTTTACACTAGAATTTTATGGGCCCTTAGGTTTCATAAGGGGTATCGTGTAATTTTCTGGGGTGGGGATATTGTAGCCCATTTCTTCCCACCTCATTGGCTGCACCTTGACCTAACGTTTTATGAAGTGACACTTCTGCTTGCCACGAACTCATTAGAGTTTGCTGAAGATGGTATACAGGCTGAGGGCAGAGGGTGGTGGTGTATCAGGGTGTATCGATTATAGAACCAGGCTCCTCTAGACGGATGTAAAGCACCGCCAGGTCAGCACGAGTTTTTGACTGTTGCTTGTAATTGTTCTGGCGAATAATTTTGTTAATTGAATTATTAGGTTTAAGGCTAAGCATAGTGGGGTATCTAATCCCAGTTTGTCATAGCTGTAGTGTATTCAGAATATTAAAGCCACTTTCGTAATTTATTTTACTGTAACTGGGGTTTTCTACGACTTGTATAGGTTAGCTTTATTTGAGTTAATAAAATCTTAAACACCTTTACGCCGTGCTCTATTAACTTGAGTCAATCGTATGGCCGCGGTGGCTGGCAGAAATTGACCAACTCTGTAGTCAGTATATAGCTTAGTTAAACTTTCGTTTATTGCTAAAAGTTTATCACTGCTGTCTCCGTGGGGGTGTGGCTAAGCAAAGTGTCTTGAGCTGCATGTTTGCGTGCTTGATACTCGCTCCTCATGTTTTGGTGGAGGGCATTTCACAGGGTCGTGGATGCTTGCATGTGTAATCTTACTGAGGGCTAATAGAAAGGCTAGGACCAAACCTATGTGTTTATGGAGTAATTACCCGTCTAGACATTTTCGGATGTCTTACTTTAAACTTAAGCTACATTAACTTATTAGAATTTAAATTAATAATAAATTAGCAAAAAAAATATATATAGCGTAATATAAGTATATTCTCTGAAGGGTAAAATTTGAGGTGTGTGGGGAGTAGAAGTGAGTAGTATTGATAATATATAATCTATAATTGTATATATATATATATATAAATATAAGTTTATATTGTGGTCAAAGGAATTGAAATGTTAATAGTTACATGTCTCTTTATGGTTCATCCTTCAGAAAATATAGGTAGGTTGGGTTTGAGGATTTGAGTTAACGTATTGTAAAGTTAATAAAATTAAAAAAAAAAAATAAAAAAAATAAAAAAAAAAATAAAAAAAAATAAAAAAAAAATAAAAAAAAAATAAAATAAAAAAAAAGGTGTTTTTTACCTTTTATCATGTCCTGTAACCATTAACTTGATATCCACTTGGGGGCGGGATGGGGGGCAAAGACATTCAATAATAGGCGCGATGATAG

>37a42abc-147a-4ab7-abb6-adaa07d23bec

TTATACTTCGTTCAGTTACATGCATTTACTGCTTCTAAAGTTGGTGATGCAAGCAGTGAGGTAAGGAGAATTGGTAGAAGTCAGAAGCTTATGTTATTTATTCGAAATGCTATATCGGGCGCCAATTATTAAAGGAATAAGATCAGTTCCCAAAACCCCTGTATAATTGGTATTACTATGAAAAAAATTATGATGAATGCGTGGGCAGTGACAATAACATTGTATACATGGTCGTCTTCTATTAGGCTTCCGGGTTGTCCTAGTTCTGTTCGAATTAGGAGGCTTAGGGCTGTACCTACCCTTTTATATACGCCAAATAATAAATATAATGTCCAATGTCTTTGTGATTAGTTAGAAAATAGTCAGCAGGTTTGAACATGATGGGGTAAAATGGCTGAGCAAGCATTAGACTGTAAATCAAGACAGAGAGTGAACCCTCTTTTACCAGCCCTGAGGTGAACTCCGCATTGAATTGCAAATTCGAGAAAAAGCTTCAATTCTGCC

>654407eb-c29f-46da-a1f0-db35bd7b794a

GGTAATAACCGTTCAGTTACGTATTGCTAGTCTTCATAGTCGGAATATTCATAGGTTCAGTATCATTGGTGACCAATTGCTTTAGGGTTAAATAAGGTTTATTAAATTCGTCTGTCATATATAGAATACGCAGTGATGGAGGGGCAATTGTAATTAGAATCAGTGCAGGAGAATAGTTCAGATTATTTCGATCTCTTGAGCATTTATGGTGCTAGTATGAGTGAGTTTTGTGAAAGAGTAAAGTATAAGGGAAATAATATATAAGACTAAGAGACTATCAGGAAAATAATTATGAGTGCATGGTCGTGGAAAGCAATAAGTTCTTCTATGATGATGTGGCATTTTGTAAACCTAGTTGAGCTGGTGTTGCTATTAAGATATATAGATATTTAGTCTATAATTTAACTTTGACAAAGTTATGTAATTATTTTACTATCTTATTGAAAAAGTCATAGGGTATATGGGATTGGCTTGAAACCAATTTTGGGGTTCAAATCCTTCCTTTTCGTCTAGGATTTAACGTAAGTTGCTCTTCGAATGTGTGGTAAGGAGGGCAGCCGTATAATCTCATTCTAGATTGGTGGATAGTTGCTCAATGGCTAAGACTTTTCGTTTTTTGAAGAGAAAGCTTCTCAAATTATAAAAACCATCAGGACTACTGCTGTAAGTGAAATAAATGAACCGATAGATGAGACGATATTTCATGTAGTATATGCGTCTGGATAGTCTAGGTATCGTCGAGGTATTCCGGATAAGCCAAGGAAGTGTTGTGGGAAGAAAGTTAAATTTACGCCGACAAATATAATGGTGAAGTGGATTTTGGCATATGTTTGGTCAAGAGTATAGCCTGAAAAAGGCAGGAATCAGTGAATAAATCCCCCTATAATGAAATACTGCTCCTAATGATACAACACATAGTGAAAGTGGGCTACTACATAGTATGTATCATGTAATACAATATCTAATGATGAGTTGTTCTTCCTGATTCTGTTAGTCCACCACGGTAAAAAGAAAAATAAAGCCCAGGGCTCATAATATTACGGGAGATCATTTGATATTACCGCCGTGCAGTGTGGCTAATCAGCTAAATACTTTTACTCAGTGGGAATGGCAATGATTATGGTAGCTGATGTAAAATATGCACGAGTATCTACATCTATTCACTGTGAATATGTGATGGGCTCACTTCAATAAAGCCCCTAGAAACCAATAGATATTATGGCTCATACCATGCCCATATAACCAAGGGTTCTTTTTATTAGAATAATATGTTACAATGTGTGAAATTATTCCAAAACCCGGTAAGATTAGAATATATACTCAGGGTGACAGAAAAATCAGAATAAGTGTTGGTATAGGATAGGGTCGCCGCCACCAGCAGGATCAAAGAAAGTAGTATTTAAATTACGGTCAGTTAATAGTATGGTAATCCCTGCAGCTAGAACTGGAAGAAAATATAAAGAAGGACTGCAGTAATTGGGATTGGATCCACACAAACAACAGGTTTATTATTGAGTCATGGCTGGCGGTTTCATATTTACAATTGTTGTAATAAAGTTAATAGCTCCAAGAATAGAAGAAATGCCTGCTAAATGTAGTGAAAAAAATGGTTAAATCTACAGAGGCTCCTGGGTGGGATAAATTGCCTGCTAGGGGTGGGTAGACTGTCCAACCAGTGCCGGCACCAAGCTTCTAAAGTTGATGATGCAACCCGGTAGGGTAGGGAGAGACAGTGAAGTCAGAAGCTTACCATTATTTGATGGGAAATGCTTATATCGGGAGCGCCAATTATTAAAGGGAATAAGTCAGTTCCCAAAACCCCACAATCATAGTGTATTTATGAAAAATTATGATGAATGCGTGGGCGGTGACAATAACATTGTATACATGGTCGTCTTCTGATAGGCTTCAAGGTTGTCTAGTTCCTGTTCGAATTAGGAGGCTTAGGGCTGTACCTACTGCCCCCGCCCATCACGCCAAATAATAAATATAATGTCCCAATGTCTTTGTGATTAGTTGAAAATAGTCAGCGGTTTATGAACATAGGTAGGGGTAAAATGGCTGAGCAAGCATTAGACTGTAAATCTAAAGACAGAGGAGTGAACCCTCTTTTTTACCAGCCCTAAGGTGAACTATCACATTGAATTTTAAATTCAAAGAAGCAGCTTCAATTCTGCCCAGGGCTTCTCCCGCCTTTTTTTCCCTAACGGCGGGAGAAGTAGATTGAAAGCCAGTTGATTAGGTTATTTAGCTGTTAACTAAATTTTTGTGGGTTAAAATCCCATCAATCTAGGAAGGAGCTTAGCTTAATTAAAGTAATTGATTTGCGTTCAGTTGATGCAGAATAAAAAGTTTGCAGTCATGTTGTTGTGCAGAAATTAAGTAAAATTTACTTACTAAGGGCTTTGAAGGCTCTTGGTCTTATTAACTAGAATTTCTAAGTTATAAGTATTAGTGGAGTTAGGGGTAGGAGACAGGTAGAAGACACTACAAGTGGGGATAGAAACGACATTGGCTTTATATAATTTAGTTGCCAGTTGATTTTTGTTATTTGATGTTGGGAATATTGTCATTGAGATATAATATATTAAACGTATATAAAAATATAAATTTATTAATGTTAGTAAAGCTATTATAAGGGGAATAATTAAGTTATCATTTTTAATAAGTTCTTGTATAATAGCTCATTTGGGGGAAAAACCTGTTAATTGAGGTAGACCTCCTAGGGATATTATTATTAGTGGAATAATGGGTATTGTTCATGCTAGTTTGTTTCAGGTATGTGATAGGGATAGGGTTGTTACATTTGAGGTTAAAATAAAAGATTATTAATGTAGAGATTGTTAGGAAAATATAAATAATTAAAGTTAATATAGTAATATTAGGGTCGTAATATAATACTGCTATTATTCATCCTATGTGAGTAATTGAAGAATGGGCTAGGATTTTGCGAGTTGTGTTTGGTTGGAGTCCTCCTCAGCTGCCAATTATAGTGATAGAACTGAGATTGTTAGAATCAAGTTTAGGTTTGTTGACAGAAAAATTTGGAGGAGAATTGATATTGGGCCCAGTTTTGTCACGTGAGAATAATTATAGCTGGGATTAGAGGGGATTCCTTGGGTAATTTCTGTAGTCAGAAGTGAAGTGGGGCTATCCTATTTTTATTGCTAGAGCAATAAATATTTATTTAATGGATAATATTTGGTTATAAGGAGGATTGATTGTTCATTTGTTGAGTTAGTAGATTGTTAAGAAAATGGATACTAATAAGAAGTATTGATGCTGTTGCTTGAATTAAAAAATATTTGAGGTGGATGCCTCACTGTGGAGCGGGGATTAGCAATACGTAACTA

>2dbb719c-d69c-48c2-9095-6f23850034cd

ATTATTACTTCGTTGATTACGTATTGCTTGCTATATCAGGAGCGCAATTATTATTAAAGGAATAAGTCAGTTCCCAAAACCCCAATCATAATTGGTATTACTATGAAAAAAATTATGATGAATGCGTGGGCGGTGACAATAACATTGTATGCATGGTCGTCTTCTATTAGGCTTCCGGGTTGTCCTAGTTCTGTTCGAATTAGGAAGCAGAAGCTACCTACTGCCCCCGCCCATGCGCCAAATAATAAATATAATGTCCCAATGTCTTTGTGATTAGTTGAAAATAGTCAGCGGTTTATGAACATAGGTAGGGGTAAAATGGCTGAGCAAGCATTAGACTGTAAATCTAAAGACAGAGGGATGAACCCTCTTTTTTACCAGCCCTGAGGTGAACTATCACATTGAATTGCAAATTCAAAGAAGCAGCTTCAATTCTGCCGGGGCACCTTTTTTCCCTA

>072cd11d-c788-4f1c-89c6-d7f848ce04fd

AATCATACTTCGTTCAGTTACGTATTGCTAAAAAATGGATTTAAATCTACAGAAGGCTCCTGGGTGGGATAAATTGCCTGCTAGGGGTGGGTAGACTGTCCAACCAGTGCCGGCACCGGCTTCTAAAGTTGATGATGCAAGCAGTAGGAGTAGGGAGGGTGGTAAGTCAGAAGCTTATGTTATTTATTCGAGGAAATGCTATATCAAGGAGCGCCAATTATTAAAGGAATAAGTCAGTTCCCAAAACCCCCAATCATAATTGGTATTACTATGAAAAAAATTATGATGAATGCGTGGGCGGTGACAATAACATTGTATACATGGTCGTGTTCTATTAGGCTTCGGGTTGTCCTAGTTCTGTTCGAATTAGGAGGCTTAGGGCTGTACCTACTGCCCCCGCCCATGCGCCAAATAAAAAAATAAATATAATGTCCCAATGTCTTTGTGATTAGTTAGTAGTCAGCGGTTTTATGAACATAGGTAGGGGTAAAATGGCTGAGCAAGCATTAGACTGTAAATCTAAAAAGACAGGCTT

>e6f322bb-c265-43f3-aff2-f5faf9199201

GTGTACTTCGTTCAGTTACGTATTGCTGGTGGTAGAAGTCAGAAGCATGGTATTATTCCGAGGAAATGCTATATCGGGAGTACGCCAATTATTAAAGATAAGTCAGTTCCCGAAAACCCCAGTCATAATTGGTATTACTATGAAAAAAATTATGATGAATGCGTGGGCAGTGACAACATTGTATACATGGTCGTCTTCTATTAGGCTTCCGGGTTTGTCCTAGTTCTGTTCGAATTAGGAGGCTTAGGGCTGTACCTACTGCCCCCGCCCATGCGCCAAATAATAAATATAATGTCCCAATGTCTTTGTGATTAGTTGAAAATAGTCAGCAGTTTATGAACATAGGTAGGGGTAAAATGGCTGAGCAAGCATTAGCAATACGTAA

>88f00b58-7776-42e3-aa9e-631a2a8b1c0a

TTAATACTTCCGTTCAGTTACGTATTGCTATTGTTCGAGGAAATGCTATATCGGGGCGCACCAATTAATGAGAATAAGTCAGTTTAAAACCCCCAATCATAATTGGTATTTACTATGAAAAAAGTCATGATGAATGCGTGGGCGGTGACAATAACATTGTATACATGGTCGTCTTCTAATAGGCTTCAGGTTGTCCTAGTTCTGTTCGAATTAGGAGGCTTAGGGCTGTACCCTTGCCCCCCGCCCATGCGCCAAATAATAAATATAATGTCCCAATGTCTTTGTGATTAGTTGAAAATAGTCAGCGGTTTATGAACATAGGTAGGGGTAAAATGGCTGAGCAAAGCATTAGACTGTAAATCTAAAGACAGAGGGTGAACCTCTTTTTTACCA

>c5a5bd34-40ec-4c39-8ac3-f99e05b16262

AGTAATATTCGTTCCGGTGCGTATTGCTTGATGATGCAAGCGATACGCAGGGTAAGGTGGTAAGTCAGAAACATGTTATTTATTCGAGGAAATGCTATATCAGGGCGCCAATTATTAAGGGAATAAGTCAGTTCCCAAAACCCCCAATCATAATTGGTATTACTATGAAAAAAAATTATGATGAATGCAGTAGGCAGTGACAATAACATTGTATGCATGGTCGTCTTCTATTAGGCTTCAGGTTGTCCCTAATTCTGTTCGAATTAGGAAGCTTAGGGCTGTACCTGCGCCCCGCCCATCACGCAAATAATAATATAATGTCCCAATCTTTGTGATTAGTTGAAAATAGTCAGCAGTTTTATGAACATAGGTAGGGAGGTAAATGGCTGAGCAGCATTAGACTGTAAAATAAAGACAGAGGAGTGAACCCTCTTTTTACCA

>54ef2279-4224-41c1-ad87-bfe6661d4e2a

TTGTACTTCGTTCAGTTACATATTACTTGCTACTATGAAAAAAATTATGATGAATGCGTGGGCGGTGACAATAACGATTGTATACATGGTCGTCTTCTATTAGGCTTCCGGGTTGTCCTAGTTCTGTTCCGAATTAAGGAGGCTTGGGAGCTGTACCTACTGCCCCCCGCCCATGCGCCAAATAATAAATATGTCCCAATGTCTTTGTGATTAGTTGAAAATAGTCAGCAGTATGAACATAGGTAAGGGTAAAATGGCTGAGCAAGCATTAGATATTTAAATCTAAAGACAGAGGTAGGCCTACATTTTTTACCAGCCCTGGTGAACTATCACATTGAATTGCAAATTCAAAGCAGCTTCAATTCTGCCGGGGCTGCCTTTTTCGGGGCTGGGTGAAGCCAGTTGATTAGGTTATTTAGCTGTTAACTAAATTTTGTGGGTTAAAATCCCG

>853eaa2d-2097-483f-81b8-3d5057b63dff

TTCATACTTCGTTCAGTTACGTATTGCTGCTATATCAGGAGCGCAATTATTAAAGGAATAAGTCAGGACGACCCCCAATCACTAATTGGTATTACTATGAAAAGAAATTATGATGAATGGAAGTAGGCGGTGACAATAACATTGTATACATGGTCGTCTTCTATTAGGCTTCCGGGTTGTCCTAGTTCTGTTCGAATTAGGAGGCTTAGGGCTGTACCTACTGCTTTTGCCATACGCCAAATAATAAAATATAATGTCCCAATGTCTTTTGTGATTAGTTGAAAATAGTCAGCGGTTTATGAACATAGGTAGAGGTAAAATGGCTGAGCAAGCATTAGACTGTAAATCTAAAGACAGAGGAGTGAACCCTCTTTTTACCAGCCCTGAGGTGAACTATCACATTGAATTGCAATTAAAAGCAACTCTGCCGGGGCTTCTCCACCTTTTTTCCCTAACGG

>16907cc8-8715-4464-9446-71cc1e4d3830

GATGTACTTCGTTCAGTTACGTATTGCTAGGGAGGGTGGTAGAAGTCAGAAACATGTTATTTATTCGAGGAAATGCTATATCAGAGCGCCAATTATTAAAGGAATAAGTCAGTTCCCAAAACCCCCAATCATAATTGGTATTACTGAAAAAAATTATGATGAATGCGTGGGCAGTGACAATAACTTGCATTGGCCATGGTCGTCTTCTGGCTAGGCTTCCGGGTTGTCCTGGATTCTGTTCGAATTAGGAGGCTTAGGGCTGTACCTACTGCCCCCGCCCATGCCAAATAATAAATATAATGTCCCAATGTCTTTGTGATTGGTTGAAATAGTCAGCGGTTTATGAACATAGGTAGGGGTAAAATGGCTAGGCAAGCACATTAGACTGTAAATCTAAAGACAGAGGAGTGAACCAGCAATACTGTAA

>0523686a-4236-4786-8821-b0b119186a8a

AGTATGCTTCGTTCAGTTACGTATTACTCCTATATTGGCAAATACTGCTCATAAGTAACACATAGTGAAATTGGGCTACTACATAGTATGTATCATGTAATACAATATCTAATGATGAGTTAGCTAACACAATTCCTGTTAGTCCACCCACGGTAAAAGAAAAATAAAGCCCAGAGCTCATAATATTGCAGGGAGATCATTTGATATTACCGCTTAAGTACGGTGTGGCTAATCAGCTAAATACTTTTACTCAGTGCGGGAATAGCAATGATTATGGTAGCTGATGTAAAATATGCTGATATCTACATATTCTATTCCTGTGAATATGTGATGGGCTCATACAATAAAGCCTAGGAAACCAATAGATATTATGTCATACCATGCCCATATAAGCAAAGTTCCTTTTTTATTAGAATAATATGTTACAATGTGAAATTATTTAGAAACCAGTAAGATTAGAATATATACTTCAGGGTGACCGAAGAATCAGAATAAGTGTTGGTATAGGATAGGGTCGCCGCCACCAGCAGGATCAAAGTAGTATTTAAATTACGGTCAGTTAATGAGTATGGCATTAATCCCTGCAGCTAGAACTGGAAGAGAAAGTAAAAGAAGGACTGCCAGTAATTAGGACGGATCACACAAACAACGGAGTTTGATATTGAGTCATAGCTGGCGGTTTCCATGTTTACAATTGTTGTAATAAAACAGCTCCAAGGAGCGAAAGTATACTAAATGTAGTGAAAAAAATGGTTAAATCTACAGAGGCTCCTGGGTGGATAAATTGCCTGCTAGGGGTGGGTAGACTATCCAACCAGTGCCGGCACCAGCTACAAAGTTGATGGTGCAAGCAGTAGGAGTAGGAGGGTGGTAGAAGTCAGAAGCTTATGTTATTTATTCAGAAATGCTATATCGGGAGCGCCAATTATTAAGGGAATAAGTCAATTCCCAAGAGCCACAATCATAATTGGTATTACTATGAAAAAAATTATGATGAATGCGTGGGCGGTGACAATAACATTGCCTTGTTGATGCGTCTTCTATTGAGCTTCCGGGTTGTCCTAGTTCTGTTCGAATTAGGAGGCTTAGGGCTGTACCTACTGCCCCCGCCCATGCGCCAAATAATAAATATAATGTCCCAATGTCTTTGTGATTAGTTGAAAATAGTCAGCGGTTTATGAACATGAGTGAAATTAGGCGTGGCTGAAAACTGACGACCAGAACTGTAAATCTAAAGACAGAGGAGTGAACCCTCTTTTTACCAGCCCTGAGGTGAACTATCACATTGAATTGCAAATTCAAAGAAGCAGCTTCAATTCTGCCGGAGGCTTCTCCCGCCTTTTTTTCCCTAACGGCGGGAGAAGTAGATTGAAGCCAGTTGATTGAGGTTATTTAGCTGTTAACTAAATTTTTGTGGGTTAAAATCCATCAATCAAGGAAGAGCTTAGCTTAATTAAAGTAATTGATTTGCGTTCAGTTGATGCAAATTAAAAGTTTTGCAGTCCTTATTTGTTGTGCAGAAATTAAGTAAAATTTACTTACTAAGGGCTTTAGGCTCTTGGTCTTATTAACCTAAATTTCTAAGTTATAAGTATTAGTGGAGTTAGGGGTAGGGGAGACAGGTAGAAGACACTGAGTGGGGATAGAAACAGTATTGGCTTTATCTCAATTCCCAAAGTTTGCCGGTTAGTTTTTTGTGTTATTTGATGTTGGGAATATTGTCATTGAGATATAATATATTAACGTATAAAAATATAAATTTATTAATGTTAGTAAAGCTATTATAAGGGGAATAATTAAGTTATCATTTTTAATAAAGTTCTTGTATAATAGCTCATTTGGAGGAAAAAACCATTAGGTAAGGGGTAGACCTCCTGGGGATATTATTATTAGTGGAATAATGAAATTATTTGTTCATGCTAGTTTGTTTCAGGTATGTGATAGGAATGAGGTTGTTACATTTGAGGTTAAACTAAAGATTATTAATGTAAAGATTGTTGGAAAAATATAAATAATTAAAGTTAATATAGTAATATTAGGGTCGTAATATAATACTGCTATTATTCATCCTATGTGAGTAATTGAAGAATGACTAGGATTTTTGCGGAGTTGTGTTTGGTTGAGAGCAATACGTAACA

>e37ab1a8-da73-49f2-9c26-28fa8933fbda

AGTGTACTTCGTTCAGTTACACGTATTGCTCTGGCGGTTTCATATTTACAATTGTTGTAATAAAGTTAATAGCTCCAAGAATAGAAGAAATGCCTGCTAAATGTAGTGAAAAAATGGTTAAATCTACAGAGGCTCCTGGGTGGGATAAATTGCCTGCTAGGGGTGGGTAGACTGTCCAACCAGTGCCGGCACCGGCTTCTAAAGTTGATGATGCAAGCAGTAGGAGTAGGGAGGGGTGGTAGAAGTCAGAAGCTTATGTTATTTATTCGAGGAAATGCTATATCGGGAGCGCCAATTATTAAAGGAATAAGTCAGTTCCCAAAACCCCCAATCATAATTGGTATTACTATATGAAAAAAATTATGATGAATGCGTGGGCGGTGACAATAACATTGTATACATGGTCGTCTTCTATTAGGCTTCCGGGTTGTCCTAGTTCTGTTCGAATTAGGAGGCTTAGGGCTGTACCTACTGCCCCGCCCATGCGCCAAATAATAAATATAATGTCCCAATGTCTTTGTGATTAGTTGAAAATAGTCAGCGGTTTATGAACATAGGTAGGGGTAAAATGGCTGAGCAAGCATTAGACTGGCAAGTCTAAAGACAGAGGGAGTGAACCCTCTTTTTACCAGCCCTGAGGTGAACTATCACATTGAATTGCAAATTCAAAGAAGCAGCTTCAATTCTGCCGGGGCTTCTCCCACCTTTTTCTAACAGCGGGAAAGTAGCGTTGAAAGCCAGTTAATTATTTAGCTGTTAACTAAGTTTTTGTGGGTTAAATCCCATCAATCTAGGAAGGGCTTAGCTTAATTAAAGTAATTGATTTGCGTTCAGTTGATGCAGAATAAAGTTTTGCAGTCCTTATTTGTTGTGCAGAAATTAAGTAAAATTTACTTACTAAGGGCTTTGAAGGCTCTTGGTCTTATTAACCTAAATTTCTAAGTTATAAGTATTAGTGGAGTTAGAGCAATACGTAACTA

>7efd5361-6d69-4581-ae40-6b119cd814a0

ATCGGTTACGTATTGCTAAAATAAAGCCCAGGGCTCATAATATTGCGGAGATCATTTGATATTACCGCCGTGCAGTGTGGCTAATCGGCTAAATACTTTACTCCAGTGAGATCATAATGATGTAGTAGCTGATGTAAAATATCACACGAGTATCTACGTATTACTGTGAATATGTGATGGGCTCATACGTAAACCTAGAAACCAATAGATATTATGGCTCATACCATGCCCATATAACCGGGGTTCTTTTTATGCGAATAATATGTTACAAATATAATAAAATTATTCCAAACCACGGTAAGATTAGAATATATACTTCAGGGTGACCGAAGAATCAATAAGTGTTGGTATAGGGATAGGGTCGCCGCCACCAGCAGGATCAAAAAGTGAAGTATTTAAATTACGATTAATAGTATGGTAATCCGCTAGAACTGGAAGAGAAAGTAAAGAAGGACTGCAGTAGTGGGGACGGATCACACAAACAACGGAGTTTGATGTTGAGTCATGGCTGGCGGTTTCATATTTACAATTGTTGTAATAAAGTTAATAGCTCCAAGAATAGAAGAAATGCCTATTAAATGTAGTGAAAAATGGTTAAATCTGCAGAGGCTCCTGGGTGGGATAAATTGCCTGCTAGGGGTGGGTAGACTGTGTAGTGCCGGCACGGCTAAAGTTGGTAATTAAAAGCAGTAGGAATTAGGGAGGGTGTTAAAAGTCAGGAAAACATGTTTATTTATTCGAAATGCTATATCAGGGCGCCAATTATTAAAAAGGAATAAAGTCAGTTCCCAAAACCCCCAATCACTTAATTGGTATTACTATGAAAAAATTATGATGAATGCGTGGGCAGTGGCAGCAACGTATACATGGTCGTCTTCTATTAGGCTTCGGGTTGTCCTAGTTCTGTTCGAATTAGGAGGCTTAGGGCTGTACCTGCCCCCGCCCATGCGCCAAATAATAAATATAATGTCCCAATGTCTTTGTGATTAGTTGAAAATAGTCGGCGGTTTTATGAACATAGGTAGAGTCCAAAAATGGCTGAGCAAGCATTAGACTGTAAAATCTAAAGACAGTTGAGCCTCTTTTTACCAGCCCTGAGG

>cb80fb34-3b77-4637-acf4-a4eb301e1a6a

ATTTGTACTTCGTTCGGTGCAGCATTACTTGTAATACAATATCTATTGATAGGTTATTAACACAATTCCTGTTAGTCCACCCACGGTAAAAGAAAAATAAAGCCCAGGGCTCATAATATTGCAGGAGATCATTTGATATTACCGCCGTGCAGGGTGGCTAATCAGCTAAATACTTTTACTCCAGTGGGAATAGCAATGATTATGGTAGCTGATTAATAAAATATGCACAGTATCTACATCTATTCCTACTGTGAATATGTGATGGGCTCATACAATAAAGCCTAGGAAACCAATAGATATTATGGCTCATACCATCCCATATAACCAAGGGTTCTTTTTTATTAGAATAATATATTACAATGTGTGAAATTATTCCAAAACCCGGTAAGATTAGAATATATACTTCAGGGTGACCGAAGAATCAGAATAAGTGTTGGTATAGGATAGGGTCGCCGCCACCAGCAGGATCAAAAGTAGTATTTAAATTACGGTCAGTTAATAGTATGGTAATCCCTGCAGCTAGAACTGGAAGAAAGTAAAAGAAGGACTGCAGTAATTAGGACGGATCACACAAACAACGGAGTTTGATGTTAGTGCATGGCTGGCGGTTTCGTAACCCACAATTGTTGTAATAAAGTTAATAGCTCAAAGAATAGAAGAAATGCCTGCTAAATGTAGTGAAAAAAATGGATTTAAATCTACAGAGGCTCCTGGGTGGGATAAATTGCCTGCTAGGGGTGGGTAGACTGTCCAACCAGTGCGGCACCGGCTTCTAAAGTTGATGATGCAAGCAGTAGGAGTAGGGAGGGTGGTAGAGTCAAAGCTTATGTTATTTATTCGAGGAAATGCTATATCGGGAGCGCCAATTATTAAAGAATAAGTCAGTTCCCAAAACCCCCAATCATAATTGGTATTACTATGAAAAAATTATGATGAATGCGTGGGCGGTGACAATATCATTGTATACATGGTCGTCTTCTATTAGGCTTCCGGGTTGTCCTAGTTCTGTTCGAATTAGGAGGCTTAGGGCTGTACCTACTGCCCCCGCCCATGCGCCAAATAATAAATATAATGTCCCAATGTCTTTGTGATTAGTTGAAAATAGTCAGCGGTTTATGAACATAGGTAGGGGGTAAAATGGCTGAGCAAGCATTAGACTGTAAATCTAAAGACAGAGGTAGACCCTCTTTTTACCAGCCCTGAGGTGAAGACTATCCACGCATTGAATTGCAAATTCAAAGAAGCAGCTTCAATTCTGCCGGGGCTTCTCCCTTTTTTCCTAACGGCGGGAGAAGTAGATTGTCGGTTGATTAGGTATTAGCTGTTAACTAAATTTTGTGGAGTTCAAAATCCATCAATCTAGGAAGGGCTTAGCTTAATTAAAGTAATTGATTTGCGTTCAGTTGATGCAGAATAAAGTTTTGCAGTCAGCAATACGTAA

>d48da370-34fc-46d1-8f7f-c5e4504a0b7f

TTGTACTTCGTTCGGTTACCGTATTGCTAGGGTGGTAGAAGTCAGAAGCTTATGGTATTTATTCGAAATGCTATATCGGGGAGCGCCAATTATTAAAGGAATAAGTCAGTTCCCAAAACCCCAATCTGAGTGGTATTACTATGAAAAAAATTATGATGAATGCGTGGGCAGTGACTTAATAGCATTAATATACATAGTCGTCTTCTATTAGGCTTCCGGGTTGTCTAGTTCTGTTCGAATTAGGAGGCTTAGGGCTGTACCTACTGCCCCCGCCCATGCGCAAATAATAAATATAATGTCCCAATGTCTTTGTGATTAGTTGAAAATAGTCAGCGGTTTATGAACATAGCAATACGAAAA

>763e17ae-6c63-4138-bc27-3d389377fec4

ATTGTACTTCGTTCAGTTACATGTATTGCTAAAAGAGCGGGAATCAGTGAATAAATCCCCTATAATGGCAAATACGCTCCTATTGATAACACATAGTGAAAGTGGGCTACTACAGCCATGTATCATGTAATACAATATCTAATGATGAGTTAGCTAACACAATTCCTGTTAGTCCACCCACGGTAAAAGAAAAATAAAGCCCAGGGCTCATAATATTGCGGGAGATCATTTGATATTACCGCCGTGCAGTGTGGCTAATCAGCTAAATACTTTTACTCCAGTGAGAATAGCAATGATTATGGTAGCTGATGTAAAATATGCACAGTATCTACATCTATTCCTACTGTGAATATGTGATGGGCTCATACAATAAGCCTAGAGCCAATAGATATTATGTGGCTCACATACCGTACCATATATTAGGGGTTCTTTTTTATTAAAGATAATATGTTACAATGTGTGAAATTATTTCAAAACCCAGTAAGATTAGAATATACTTCAGGGTGACCGAAGAATCAGAATAGTGTTTGGTATAGGATGAGATTAGCCGCCACCAGCAGGATCAAAGTAGTATTTAAATTACGGTCAGTTAATAGTATGGTAATCCCACTGCAGCTAGAACTGGAAGAGAAAGTAAAAGAAGGACTACTATTAATTAGGACGGATCACACAAACAACGGAGTTTGATGTTAGTCATGGCTGGTTTCATATTTGTTGTAATAAAGTTAATAGCTCAAGAATAGAAGAAATGCCTGCTAAATGTAGTGAAAAATGGTTAAATCTGCAGAGGCTCCTGGGTGGGATAAATTGCCTGCTAGGGTGGGTAGACTGTCCAACCAGTGCCGGCACCGGCTTCTAAAAAGTTGATGATGCAAGCAGTAGGAGTAGGGAGGGTGGTAGGAAGTCAGAAGCTTATGTTTATTCGAAGGAAATGCTATATCAGGAGCGCCAATTATTAAAGGAAGATTTAATCTATGAATTGGTATTACTATGAAAAAATTATGATGAATGCGTGGGCGGTGACAATAACATTGTATACATGGTCGTCTTCAATAGGCTTCCGGGTTGTCCTAGTTCGTTCGAATTAGGAAGCAGGGCTGTACCTACTGCCCCCGCCCATGCGCCAAATAATAAATATAATGTCCCAATGTCTTTGTGATTAGTTGAAAATAGTCAGCGGTTTATGAACATAGGTAGGAGGTAAAATGGCTGAGCAAGCATTAGACTGTAAATCTAAAGACAGAGGAGTGAACCCTCTTTTTACCAGCCCTGAGGTGAACTATCACATTGAATTGCAAATTCAAAGAAGCAGCTGAATTCTGCCGGGGCTTCTCCCGCCTTTTTTTCCCTAACGGCCCGGGAGAAGTAGATTTAAGCCAGTTGATTAGGTTATTTAGCTGTTAACTAAATTTTTGTGGGTTAAAATCCCATCAATCTAGGAAGGGCTTAAGCAATTAAAGTAATTGATTTGCGTTCAGTTGATGCAGAATAAAGTTTTGCAGTCCTTATTTGTTGTGCAGAAATTAGTAAGTTACTTACTAAAA

>2abe4026-e80f-47fe-a107-b8c53c4486f3

AGTATGTGCTTTAGTTCAGTTACGTATTGCTCCGGCTTCTAAAGTTGATGATGCAAGCAGTAGGAGTAGGGAGGGTGGTAGAAGTCAGAAGCTTATGTTATTCGAGGAAATGCTATATCGGGAGCGCCAATTATTAAAGAGGAATAAGTCAGTTCCCAAAACCCCCAATCATAATTGGTATTACTATGAAAAAAATTATGATGAATGCGTGGGCGGTGACAATAACATTGTATACATGGTCGTCTTCTATTAGGCTTCCGGGTTGTCTAGTTCTGTTCGAATTAGGAGGCTTAGGAGCTGTACCTGCTGCCCCCGCCCATGCGCCAAATAATAAATATAATGTCCCAATGTCTTTGTGATTAGTTGAAAATAGTCAGCGGTTTATGAACATAGGTAGGGGTAAATGGCTGAAAGACAAGCATTAGACTGTAAATCTAAAGACAGAGGAGTAGACCCTCTTTTTTTACCAGCCCTGAGGTGAACTATCACATTGAATTGCAAAATTCAAGAAGCAACTTCAATTCTGTAAGGCTTCTCCGCCTTTTTTCCCTAACAGCGGGAGAAGTAGATTGAAGCCAGTTGATTGGGTTATTTAGCTGTTAACTAAATTTTTGTGGGTTAAAATCCCATCAATCTAGGAAGGGCAATACGTAA

>6e645ab4-e086-476f-bf9b-f11cecdc8c82

AGTGCTGCTTCGTTCGGTGCAGCTGTTGGGTCGCCGCCACCAGCAGGATCAAAGAAAGTAGTATTTAAGTACGGTCAGTTAATAGTATGAAGTAATCCCGCAGCTAGAACTGGAAGAAAGTAAAGAAAGGACTGCAGTAATTAGGACGGATCACACAAACAACGGAGTTTGATATTGAGTCATGGCTGGCGGTTTCATATTTACAATTGTTGTAATAAAGTTAATAGCTCCAAGAATAGAAGAAATGCCTGCTAAGTAAATGAAAAAATGGTTAAATCTACAGAGGCTCCTGGGTGGGATAAATTGCTAGGGGTGGGTAGACTGTCCAACCAGTGCCGGCACAAGCTACAAAGTTGATGATGCAAGCAGTAGGAGTAGGGGAGGGTGGTAGAAGTCAAGCTTATGTTATTTATTCGAGGAAATGCTATATCGGGAGCGCCAATTATTAAAGGAATAAGTCAGTTCCCCAAAACCCCCAATCATAATTGGTATTACTATGAAAAAAATTATGATGAATGCGTGGGCAGTGACAATAACATTGTATACATGGTCGTCTTCATTACAGGCTTCCGGGTTGTCCTAGTTCTGTTCGAATTAGGAGGCTTAGGGCCTGGCACCTGCTGCCCCCGCCCATGCGCCAAATAATAAATATAATGTCTTTGTGATTAGTTGAAAATAGTCGCCCGGTTTATGAACATATAGGTAGGGGTAAAATGGCTGAGCAAGCATTAGACTGTAAGAAATCTGCAAAGACAGAGGAGTAGACCCTCTTTTTACCAGCCCTGAGGTAAGACTATCACATTGAATTGCAAATTCAAGAAAGCTTCAATTCTGCAAAGACTTCTCCGCCTTTTTTCCCTAACGGCGGGAGAAGTAGATTGAAGCCAGTTGATTAGGTTATTTAGCTGTTAACTAAATTTTTGTGGGTTAAAATCCCATCAGAAAATCTAGGAAAGACTTAGCTTAATTAAAAGTAATTGATTTGCGTTCAGTTGATGCAGAATAAAGTTTTGCAGTCCTTATTTACATTTGAAATTAAGTAAAATTTATACTAAGGGCTTTGAAGGCTCTTGGTCTTATTAACCTAAATTTCTAAGTTATAAGTATTAGTGGAGTTAGGGTAGGAGACAATTGAAGCTTTACAGTGGGATAAACAGTATTGGCTTTATATAAATTTAGTTGCCAGTTGATTTTTTTGTGTTATTTGATGTTATGGGGAATATTTGTCATTTGAGATATAATATATTAAACGTATATAAAAATATAAATTTATTAATGTTAGTAAAGAAGCTATTATAAGGGGAATAATTAAGTTATCATTTTTAATAAGTTCTTGTATAATAGCTCAGCAGCCGTAA

>a935667a-a4c0-47b3-b065-4baf07f44f30

ACTTCGTTCAGTTACGTATTGCTCACGGTAAAATATTTAGTTGAGGCATTCACTATAGAGAGTTTGTTCTCTCAATCTTTAACTTAAAAAGGTTAATGCTAAGTTAGCTTTACAGTGATACAATATATAAGTATGAAGCTCATACTTCGAAATCTTGAAATAAATGAATTCTAGAACAATAGGTATAAAGCTGTGATTGGACCCGCAAATTTCCGAGCATTGTCCATAAAATAGGCCTGGTCGTATAGAGGCTATTATGGCTTGGTTTAAACGTCCAGGAATTGCATCTGTTTTTACGCCTAGTGATGGTACGGCTCATGAGTGTAAGACGTCTTGTGATGAGATTAATATGCGAATATCCGCTTCTATAGGTAAAGTTGTTCGGTTATCAACTTCGAGGAGTCGAAATTCCCCTGGCTCAAGGAAATATGTTGGCATAATGTAAGAGTCAAATACTAAGTCTTCATAGTCGGAATATTCATAGGTTCAGTATCATTGGTGACCAATTGCTTTAAGGGTTAAATGAGTTTATTAAATTCGTCTGTCATATATAGAATACGCAGTGATGGGAGGGCAATTGTAATTAGAATCAGTGCAGGGAGAATAGTTCGGGTCTTTCGATCCTCTTGAGCATTTATGGTGCTAGTATGAGTGAGTTTTGTGGTAAGTATAGGGAAATAATATATAAGACTAAGGAACTAATCAGGAAAATAATTATGAGTGCATAGTCGTATTGGAAAGCAATAAGTTCTTCTATGATAGGTGATGTGGCATTTTGTAAACTAGTTGAGCTGGTGTTGCTATTAAGATATATAGATATTTAGTCTATAATTTAACTTTGACAAAGTTATGTAATTATTTTACTAATATCTTATTGAAAAAGTCATAGGGTATATGGGATTGTGAAACCAATTTTTAGAGGTTCAAATCCTTCCTTTTCGTCTAAGTTTAACGTAAGTTGCCTCGAATGTGTGGTAAGGAGGAGAGCAGAGGAGGGCGTATAATCATTCTAGATTGGTGGATAGTTGCTCAATGGCTAAGACTTTTCGTTTTGAAGAGAAAGCTTCTCAAATTATAAAAACCATCAGGACTACTGCTGTAAGTGAAATAAATGAACCGATAGATGAGACGATATTTCATGTAGTATATGCGTCTGGATAGTCTGAGTATCGTCGAGTATTCCGGATAAGCCAAGGAAGTGTTGTGGGAAGAGAAAAGTTAAATTTACGCCGACAAATATAATGGTGAAGTGGATTTTGGCATATGTTTGGTCAAAGGTATAACCTGAAAAGAGCGGGAATCAGTGAATAAATCCCCCTATAATGGCAAATACTGCTCCTATTGATAACACATAGTGAAAGTGGGCTACTACATAGTATGTATCATGTAATACAATATCTAATGATGAGTTAGCTAACACAATTCCTGTTAGTCCACACGGTAAAAAGAAAAATAAGGCAGGGCTCATAATATTGCGGGAGATCATTTGATATTACCGCCGTGCAGTGTGGCTAATCAGCTAAATACTTTTACTCCAGTGGGAATAACAATGATTATGAAGTACAGCTGATGTAAAATATGCACAGTATCTACATCTATTCCTACTGTGAATATGTGATGGGCTCATACAATAAAGCCTAGGAAACCAATAGATATTATGGCTCATACCATGCCCATATAACCAAGGGTTCTTTTTTATTAGAATAATATGTTACAATGTGTGAAATTATTCAAAACCCGGTAAGATTAGAATATATACTTCAGGGTGACCGAAGAATCAGAATAAGTGTTGGTATAGGATGGGGTCGCCGCCACCAGCAGGATCAAAAGAAAGTAGTATTTAAATTACGGTCAGTTAATAGTATGGTAATCCCTGCAGCTAGAACTGGAAGAGAAAGTAAAAGAAAGGACTGCAGTAATTAGGACGGATCACACAAACAACGGAGTTTGATATTTAGTCATGGCTGGCGGTTTCATATTTACAATTGTTGTAATAAAGTTAATAGCTCAAAGAATAGAAGAAATGCCTGCTAAATGTAGTGAAAAAAATGGTTAAATCTACAGAGGCTCCTGGGTGGGATAAATTGCCTGCTAGGGGTGGGTAGACTGTCAACCAGTGCCGGCACCACAACTTCTAAAGTTGATGATGCAAGCAGTAGGAGTAGGGAGGAGGGTGGTAAAAGTCAGAAACATGTTATTTATTCGAGGAAATGCTATATCAGGGCGCCAATTATTAAAGGAATAAGTCAGTTCCCAAAACCCCCAATCATAATTGGTATTACTATGAAAAAAATTATGATGAATGCGTGGGCGGTGACAATAACATTGTATACATGGTCGTCTTCTATTAGGCTTCCGGGTTGTCCCTAGTTCTGTTCGAATTAGGAGGCTTAGGGCTATGCCTGCTGCCCCCGCCCATGCGCCAAATAATAAATATAATGTCCCAATGTCTTTGTGATTAGTTGAAAATGGTCAGCGGTTTATGAACATAGGTAGGGGTAAAATGGCTGAGCAAACATTAGACTGTAAATCTAAAGACAGAGGAGTGAACCCTCTTTTTACCAGCCCTGAGGTGAACTATCACATTGAATTGCAAATTCAAAGAAGCAGCTTCAATTCTGCCGGGGCTTCTCCGCCTTTTTTCCCCTAACGGCGGGAGAAGTAGATTGAAAGCCAGTTGATTAGGTTATTTAGCTGTTAACTAAATTTTTGTAGGTTAGAAATCCCATCAATCTAGGAAGGGCTTAGCTTAATTAAAGTAATTGATTTGCGTTCAGTTGATGCAAGAATAAAGTTTTGCAGTCCTTATTTGTTGTGCAGAAATTAAGTAAAATTTACTTACTAGTAATACGTAACA

>0bf596c9-9d8b-4345-b895-88f94240c2fa

TTATTACTTCGTTCAGTTACGTATTGCTAAGTAGTATTTAAATTACGGTCAGTTAATAGTATGGTAATCCACTGCAGCTAGAACTGGAAGAAAGTAAAGAGAAGGACTGCAGTAATTAGGACGGATCACACAAGCAACGGAGTTTGATATTGAGTCATGGCTGGCGGTTTCATATTTACAATTGTTTGTAATAAAGTTAATAGCTCCAAGAATAGAAGAAATGCCTGCTAAATGTAGTGAAAAATGGTTAAATCTACAGAGGCTCCTGGGTGGGATAAATTGCCTGCTAGGGGTGGGTAGACTGTCCAACCAGTGCAGCACCGGCTTCTAAAGTTGATGATGCAAGCAGTAGGAGTAGGAGGGAGTGTTGGAAGAAGTCAGAAACTTATGGTATTTATTCGAAATGCTATATCGGGAGCGCCAATTATTAAAGGAATAAGTCAGTTCCCAAAACCCCAATCATAATTGGTATTACTATGAAAAAAGAATTAATGATGAATGCATTAGGCGAGTGACAATAACATTGTATACATGGTCGTCTTCTATTAGGCTTCCGGGTTGTCCTAGTTCTGTTCGAATTAGGAGGCTTAGGGCTGTACCTACTGCCCCCATATGCATAAAATAATAAATATAATGTCCCAATGTCTTTGTGATTAGTTGAAAATAGTCAGCGGTTTATGAACATAGGTAAGGGAGTAAAATGGCTGAGCAAGCAGTAGACTGTAAATCTAAAGACAGATCGAACCCTCTTTTACCAGCCCTGAGGTGAACTATCACATTATGTTAAGCGTTATTGTTGAAGTTAGAAAATTTATTTCTTATTATTTTTTAAAAATTCAAAAAAGAAGCAGCTTCAATTCTGCCAGGCTTCTCCCGCCTTTTTTCCCTAACGGCGGGAGAAAGTAGATTAAAACCAGTTGATTAGGTTATTTAGCTGTTAACTAAATTTTGTGGGTTAAATCCATCAATCTAGGAAAGGGCTTAGCTTAATTAGTAATTGATTTGCGTTCCAGTTGATGCAGAATAAAGTTTTGCAGTCCTTATTTGTTATTTTAGAAATTAAGTAAAATTTACTTATACTAAAGGGCTTTTGAAGGCTCATGGTCTTATTAACCTAAATTTCTAAGTTATAAGTATTAGTGGAGTTAGGGGTAGGAGACAGGTGAAGACACTACAAGTGGGGATAAAAACGGTATTGGCTTTATATAATTTAGTTGTAATTGATTTTTGTGTTATTTGATGTTGGGAATATTGTCATTGAGATATAATATATTAAACGTATATAAAAATATAAATTTATTAATGTTAGTAAAGCTATTATAAGGGAATAATTAAGTTATCATTTTTAATAAGTTCTTGTATAATAGCTCATTTGAGAAAAACCTGTTGGTGGGGGGTAGACCTCTAGGGATTCTTATAATAGTCAGCATATATCTTCAAGTCTCTGATAGCTTCTTTCGGCAATGAGTAAGATTTCAATATTTCAACCTCGTTTTCAATACCATTTGAATTGCTCATCAAACCCAATGTACTACTGCTGTTGAGCAACATGTTGCCATACCGGCTACTTTTCTGACTATCGTTGTCTTTGATGAGCAACTTAGTACTTGCCTTATATACAGGAGTCGCATAACGAAGGTAAACTCTGCTACAAACTATACATAGGAGCATAGAAAGTAGATACCATTTCCAGTTAAGGATAAGGGTAGTGTAAATGGTAGTGAAATCAATGGATGATCTTTCTTCAGTTTCTGCACTGTTGTAAGAAGCGTCAATTTCAAGTTTCTTGTTTTCTTCCATTTTCTGTTATATCATTATAGTATTATTCTGTTAATCTAAGAAGATATTGACCATACTCGTTTTAATCATTGGTTTGTGCTTCTTTAAGCTTCTCGACTGTTATCAAAAGCCCTTCTTGAAGCTATCTCTTCAAGACAAAAACGCTTTCAGTCCCTGTCGCTTTTCAATAACTTCTATAAAAGTACTTGCTTCGCTCAGACTGTCGTGAGTGCCTGTATCAAATGACCATGCAAAACCGCGTTGTAGAGGTTCTTACTGCAGATTCACCTTTTTAGATATTCTTGGTTTACCGTTGTTATCTCAAGTTCACCACGTGCACTTGGCTTTATGTTTTTAGCAATTTTACAACGCTGTTAGGATAGAAATATAGTCCTACAACTGCATAATTGCTCTTAGGATGTTCTACTACGAAGTAGCGGTTGACTACCGGGCTGCAAGAAGGAAGACGTTCAGATGGATCTGAACGACGGCTACTGACCATTCAGGCAGTGCGCAGCAAAGCAACGATGAAAAGGATAAAGAGGGCCGCTATCTGCGGCAGATCCTCTCCGGCACCTGCGCACGCAGTTTACGTGGGCGATGTGAAGAGAAGACATCCACGCAAAGTTCGAGGATGGCGTCCTGCACGTCGAGCTGCCTGCTCCTCGGCGAACCGAAGGCTTTGCAAACGCGAAACCCGAACCTGATCGAGATCGAGTAAAACGCCTTGCCGATCGTGTACGCCAAAGCACAACGCACATAAGATGCCTGGAGAGCCGCAACGAGCCCTCCGGGAGCATTTTTGTTTCGCTATCTTTTCCGCAAAGGGAAAACCATGAGCTGTAAAGAACAACCCAACTCCGTTTGCTTGAGTACCTCACTATATCCTGTGAGCCGGGACAGCGGCTGACTTTTGCGGTATACTCCGTTCGGAAAGATCAAGCGCATGGAGGTAAAAAGCATGTTGACCTATACCTATGTTTCAGAGGGAACGTTTGCCCTGATGGAAAAAGCCAAAGCCGGTGCTGCACGAGCAGGACGCCATTATGTAAGGTGACCCTTGCCAGCATCACTCCAGCGACCTGCACATTAAGCATGGCAGCGTGCCCCGGGCGGTGCCCGGCATCACGGTAAGGGCGCGAGATGGTGGGCATCGTAGAAGAAGTGGGCAGTGCGGTGACCAATGTGAAACCCGGCGACCGGGTGACGGTGAACAGTGGAGACGCCTTCTGCGGAGGTACTTCTTCTGCAAAAAAGGCTTTGTGAACAACTGCACCGATCAAAACGGCGGCTGGGCGCTGGGCTGCCATCGACGGCAGGCAGGCAGAGTATGTCCGGGTGCCCTTTGCGGATCAGGGACTGAACAAGATCCCGGACGGCGTTACCGTCGGCAGAGCGCGCTGTTGAGCCGGGCGTCGCTGGCCACCGGGAAGGCGTCGAAGTGCCGCCTGCGGCGGAAGTTCGTGCCGTTGCGATCCTGGTGAGGCTCGTGTGGCATCTGCACGCTGCTGTGCGTCATGCTGCACAGCCCCAAACTGCATCATCGTCTGTGAAAAGGACGCAAGCCGCCTGCAGTTCATCCGTCGGCACTACCCGCAGGTGCTCACCGTGCGTAGGAGGACGCTGCGCCTGCTGTGCGTGCCCACGCGCCACGGCGGGGCCGATGGCCGAACTGGGAGGTGGCAGAGCAGACTCCACCTTCCGGCTGGCATGGGGTGTGCGGCCCAGCGCCATTGTGACGGTGGTGGCACTGTACAGTAAAGGCACAGACCCTGCCGCTGCCGGAAATGTACGGCAAAAACCTCACCTTCAAGACCGGCGGCGTGGACGGCTGCGATTGTGAAGAAACGCGCGTCATCGCCAGAAGGCAAGATCGATACCGAACCGCTAATCACCCACACCTACCCCCTGCGCCGGATCGCAGAGGGCTACGAGCTATTTGAAAAGCAGGGCGGCGTGATCAAGGTGGCAGTGGAGTGCTGACAGGTGCTGCGCCAGGCACAACGCACATAAGGTGACCCCGGAGAGCGCGTAACAAGCCCTCCGGGGCCATTTTTTGTTCTGGCATCTTTTCCGCAGCGGCAAGGCGTGGTAGAATAAGGGCAGAACCAATCCACAGGTGAAAAATCGAACTTCAGCCTGAAATGCGGAGCCGTTGCATCGAGCATCGCACGCCCTTCCCATAACCTGATCGTGGCGGGGGACAACGATGCCGACATAATGTTGGCCGGCAACACTGTGCGGAAAAACAAAGGCGGACTTTGCGTTTGTGGCAGATGGACAGGTCGTGGCAGAACTTGCGCTTCCGGTGGCCGGATTGGTGAGCACCAGCGCAGAGAGCGTTGCGGCGAAAATGCGACTGAACCGATGCCCTCAAGGCACATGGTGTAGCGGAAGATATCGGCATCTTTATGACCCTGCCCTTCGTCAGCCTTCCGGTCATTCCAAAACTCAGACTGGAACTATGCGGCATCATCGATGTAGCGCAGCAGAAAGTCGTTCCGGCTGTTTTTAATACGCGATAACAAAAATACTGCCGGAGACCGACCCGAATGGTCCGTCTCCGGCAACAGAACAGTTCGTTCCTACTCTTAGAAGCGAGCCGCATCAAGAAAAAATGACGGAAATTCTTATTTGATAAATCGAGTTGTATCAGCATAGAAGGAGAATATCGTATGATTCAGAACAATTCTTAAAAAGAAAAATGAAATCAGCATCGACACGGCAAGGCTCTTTTGCAGTCCAGCCGACGTGGCGTTCTTGCCGTAAAATGGAGATGACGGATATCCCTATGCAATTCCTATCAACTATGTCTACGATGACGATGCGCAGAAATTTATTTTCATGGAGCACGCGTAGGTCATAAAGTAGATGCACTGCGTGCCTGTGACAAAGTAAAACAACTACGATATATGGAAATGAAACCATCAAAGAAGAAGATTGGGCACCGTTTGTGCAGAGCGTTGTGGTGTTCGGCAGATGCCACCTCGTAGAATCCGGTGCAAGAGCAACAACTCTGTTAAAACGATTTGCAATGAAATACTATCCCAGCGAACAGTTAGTTGATGAGGAAATTGCCCACGCAGGAAAGGCTGTGCAAATTTTTTGAAATAGACATTAGGAGTACCTCAGTGGCAAGGAAATACAGGAACGATAAATTCGCGTTTGTGGAGCGAAGCAGAACAACTGGCAGTGTGGGGCTTTTAAGCGGCAGAACGGTCTTGCGTAGCAAAGATGGAGGAGCTTTGCCCCGACAAGTTCACGTTGTGGAAATTACAAGGGGTGCATCATGGAAATCGAGTATAAACAAAAGATTTCACGGCCGATGAATTGCCAGAAACTGTTTTTATCCGTTCACTGAAATCAACAATTATCCCGAAAAACTTGTCCGTGCGATGCACATTCCACACGAGTTATTTCTAATGGTGGGATGGTGATAAGCTGGTCGGGTTCGCGCGCTTGATAATGGAGAAATAGTCGCATTTTTGCATTATCTTTGGTGGACCTGCTTATCGCATGGCGACGAGTTGATGAAACGGATCCTTGATTTCTATCAAAATCTTCTGTGTAGAGATTATGCCGTCTGATCCTAAAACGATCCCATTTTATGAGCGATATGGCTTTCAACAATATGATAATTACTCTGCAATGGTGCGGAAGCACTTTTTGTGAGTTGGTATGATGAATCGATTTGCAGCTAGCAAGGAAACCGTAAAAGTACCATGGATGCAAACTGTACCAGTAAAGAAGGAAAGTCAAGAGTATGGAAACGGTATACAGCAATACGTAGCA

>cab508e2-7111-43c5-a3fc-b5a558d0f00b

ATACTTCGTTCAGTTACGTATTGCTTCCTATTGTTTGGAATATGAATAGAGCAAATAGATTTTGGTTCATTTTAGTTCCTACTGTTTGCTGTTTTTGTGTTTCTACTAATTTTGGTAGTGGAGTAATAATGGAAAGTAAAGTTCAGTATTTTCAATTGTATAATATAAAATAAGGTAACAATTATAGATATAATTACTATTGGTCACGGTGAGAAATATTTAGTTGAGGCATTCACTATGAAAGTTTGTTCTCTCAATCTTTAACTTAAAAAGGTTAATGCTAAGTTAGCTTTACAGTGATACAATATAAGTATAGTATACTTGAAATCTTGAAATAAATGAATTCAGACAATAGGTACTCGCTGTGATTGGACCCGCAAATTTCCGAGCATTGTCCATAAAATAGGCCCTGGTCGTATAGAGGCTAGTATGGCTTGGTTTAAACGTCCAGGGGCCATCTGTTTTTACGCCTAGTGATGGTACGGCTCTATAGGTGTAAGACGTCTTGTGATGAGATTAATACATATCCTTCTATATGATCAAGTTGTTCGGTTATCAGCAAGGAGTCAAGTACACTGGCTCAAAAGGAAATATGTTGTATAATGTAAGAGTCAAATACTAAGTCTTCATAGTCGGAATATTCATAGGTTCAGTATCATTGGTGACCAATTGCTTTAAGGGTTAAATAAGGTTTATTAAATTCGTCTGTCATATATAGAATACGCAGTGATGGGAGGGCAATTGTGATAGAATCAATTGGGGAGAATAGTTCAGATTATTTTCGATGTGAGCATTTATGGTGCTAGTGTAGTGAGTTTTGTGGTAAAGTATAAGGAAACATATATAAGACTAAGGAACTAATCAGGGACGAAATTGAGTGCATGGTCGTAGGAAAGCAATAAGTTCTTCTATGATAGGTGATGTGGCATTTAACCTAGTTGAGCTGGTGTTGCTATTAAGATATATAGATATTTAGTCTATAATTAACTTTGACAAAGTTATGTAATTATTTTACTAATATCTTATTGAAAAAGTCATAGGGTATATGGGATTGGCTTGAAACCAATTTTTGGGGGTTTAAAATCCTTCCTTTTCGTCTAGGATTTAACGTAAGTTGCCTCTTCGAATGTGTGTGGTAAGGAGGAGGGCAGCCGTATAATCATTCTAGATTGGTGGATAGTTGCTCAATGGCTAGACTTTTTCGTTTTGGAAGAGAAAGCTTCTCAAATTATAAAAACCATCAGGACTACTGCTGTAAGTGAAATAAATGAACCGATAGATGAGACGATATTTCATGTAGTATATGCGTCTGAATAGTCTGAGTATCGTCGAGGTATTCGGATAAACCAAGGAAGTGTTGTGGGAAGAAAGTTAGAATTTCGCCGACAAATATAATGAAGTGGATTTTGGCATATGTTTGGTCAAGAGTATAGCCTGAAGAGCGGGAATCAGTGAATAAATCCCCTATAATGGCAAGCTGCTCCTAATGATAACACATAGTGAAGTGGGCTACTATAGTATATCATGTAATATACAATATCTAATGATGAGTTAGCTAACACAATTCCTGTTAGTCCACCCACGGTAAAAAAGAAAAATAAAGCCCAGGGCTCATAATATTGCAGGAGATCATTTGATATTGCCGCCGTTGATGTGGCTAATCAGCAATAACGCACGCCAGTGGGAATAGCAATGATTATGGTAGCTGATGTAAAACATGCACAGTATCTACATCTATTCTACTGTGAATGGTGGGCTCATACAATAAAGCCTAGGAGGCAATAGATATTATGGCTCATACCATGCCATATAACCAAAGGGGTTCTTTTTTATTAGAATAATATAATGTTACAATGTGTGAAATTATTCCAAAACCCAGAGGTAAAGATTAGAATATATACTTCAGGGTGACCGAAGAATCAGAATAAGTGTTGGTGGGGATAGAGGTCGCCACCTTACCCAGCAGGATCAAAAGTAGTATTTAAATTACGGTCAGTTAATAGTATGGTAATCCCTGCAGCTAGAACTGGAAGAGAAAGTAAAAGAAGGACTGCAGTAATTAGGACAGATCACACAAACAACGGAGTTGATATTGAACAGCTGTCGGTTTCATATTTACAATTGTTGTAATAAAGTTAATAGCTCAAGAATAGAAGAAATGCCTGCTAAATGTAGTGAAAAAATGGTTAAATCTACAGAACTCTGGGTGGGATAAATTACCTGCTAGGGGTGGGTAGACTGTCCAAGCAGTGCCGGCACCGACTTCTAAAGTTGATGATGCAAGCGGTAGGAGTAGGGAGGGTGGTAGAAGTCAGAAGCTTATGTTATTTATTCGAGGAAATGCTATATCAGGAGCATGAATTATTAAAGGAATAAGTCAGTTCCACAAAAACCCCAATCATGAGTGGTATTACTATGAAAAAAAATTATGATGAATGCGTGGGCGAGCAGCAATAACATTGTATACATGGTCGTCTTCTATTAGGCTTCCGGGTTGTCCCTAGTTCTGTTCGAATTAAGGAGGCTTGGGGCTGTACCTACTGCCCCGTACGCCAAATATGTCTCAATGTCCAAATGTCTTTGTGATTAGTTAATAATTAAGCGGTTTATGAACATAGGTAGGGTAAATGACTGAGCAAACGCATTAGACTGTAAATCTAAAGACAGAGGAGTGAACCTCTTTTACCAGCCTGAACTATGTTGAATTGCAAATTCAAAGAAGCAGCTTCAATTCTGCCGGGGCTTCTCCCGCCTTTTTCCCTAACGGCAGGAGAAGTAGATTCAAAGTTGATTAGGTTATTTAGCTGTTAACTAAATTTTGTGGGTTAAAATCCCATCAATCTAGGAAGGGCTTAGCTTAATTAAAGTAATTGATTTGCGTTCAGTTGATGCAGAATAAAGTTTTGATCCTTATTTGTTGTGCAGAAATTAAGTAAAATTTTACTAAGGCTGAAGGCTCTTAGCAATACGTAA

>6552e5b3-afe6-4270-8d2d-5bcc7b7c9128

AGTATGCTTTAGTTTCAGTTACGTATTGCTAATATTGGCTGTTAATCGTACGGCTAAAGGCTGAGTTGAATGAATCGAACCAATTGTTTCAATAATAATTAATATAGGGATTAGTGGAATTGGTGTTCCTTGTGGTAAATGAGCGAGGGATGATTTTGTTTTAAATCGGAAGCCTATTAGTACAGTTGCTGCTCATGAAGATAGCTAGCACTAGATTTATTGACAGTTGGGTGGTTGGTGTAAATGCATATGGTGTAAAGTCAAGAATGTTTATTTAGGGCAATAAAAGAAATTAGGGCTAGGAGTATAAGGGATCAGGTTCGCCCTTTAGTGGTGCTAGTTAATATTATTTGTTTAAGTGTTAGTTGAATTAGCCATTGTTGAATTGAAGAGAGTCAGTTTGTTGAATTAGTTTTGGGATAAAATTAATGTAGTGGGGAGTGCAATAATTAAAAATACTAAGGGTACTCTAGTATTAATTTTGGAATATTGAATGAGGCAAATAGATTTTGGTTCATTTTAGTTCTCAAGTTGTTTTATGTTTTTGTGTTTCTACTAATTTTGGTGAGTAGGTAATAATGGAAAGTAAAGTTCCGGTATTTTCAATTGTATAATATAAAATAGGTAACAATTATAGATATAATTACTATTGGTCACGGTGAAATATTTAGTTGAGGCATTCACTATAGAGAGTTTGTTCTCTCAATCCTTTAACAAAGGTTAATACTAAGATTAGCTTTATGATACAATATATAAGTATGAAGCTCATACTTCGAAATCTTAGAAATAAATGAATTCTAGAACAATAGGTATAAAGCTGTGATTGGACCCGCAAATTTCCGAAGCATTGTCATAAAAATAGGCCTGGTCGTATAGAGAAGCTGAAATATGGCTTGGTTTAAACGTCCAGGAATTGCATCTGTTTTTACGCCTAGTGATGGTACGGCTCATGAGTGTAAGACGTCTTGTGATGGAATTAATATGTACACGAATATCGCCTATAGGTAAAGTTGTTCAGTTGCTCAACTACCGAGAGTCGAAATTACTGGCTCAAGGAAATATGTTGGCATAATGTAAGAATTAAATACTAAGTCTTCATAGTCGGAATATTCATAGGTTCAGTATTTGGTGACGAATTGCTTTAAAGGGTTAAATAAGGTTTATTAAATTCGTCTATCATATATAGAATACGCAGTGATGGGAGAACTGGTGTAATTAGAATCAGTGCGGAGAATAGTTCAGATTATTTCGATCTCGCTTGAGCATTTATGGTGCTAGTATGAGTGAGTTTTGTGGTAAGTATAAGGGAAATAATATATAAGACTAAGGAACTAATCAGGAAAATAATTATGAGTGCATGGTCATTGGCAATAAGTTCTTCTATGATAGGTGATGTGGCATTTTTGTAAACCTAGTTGAGCTGGTGTTGCTATTAAGATATATAGATATTTAGTCTATAATTTAACTTTGACAAAGTTATCATAGGGTATTTTACTAATATCTTATTGAAAAAAGTCATAGGGTATATGGGATTGGCTTGGAAACCAATTTTTGGGGGTTCAAATCCTTCCTTTTTCGTCTAGGATTTAACGTAAGTTGCCTCTTCGAATGTGTGGTAAGGAGGAGGGCAGCCGTATAATCCATTCTAGATTGGTGGATAGTTGCTCAATGGCTAAGACTTTTCGTTTTTGAAGAGAAAGCTTCTCAAATTATAAAACCATCAGGACTACTGCTGTAAGTGAAATAAATGAACGATAGATGAGACGATATTTCATGTAGTATATGCGTCTGGATAGTCTGAGTATCGTCGAGGAATTATTCGGATAAGCCAAGGAAGTGTTGTGGGAAGAAAGTTAAATTTACGCCGACAAATATAATGGTGAAATTAGTTTTGGCATATGTTTGGTGCGTGCCTGAAGAAAGAGCGGGAATCAGTGAATAAATCCCTATAATGTAAATACTGCTCCTATTGATAACACACATCTGGTAAAGTGGGCTACTACATAGTATATCATGTAATATCTAATGATGAGTTAGCTAACACAATTCTGTTAGTCCACCCACGGTAAAAGAAAATAAAGCCAGGGCTCATAATATTGCGAGAATCATTTGATATTACCCGCCGTGCAGTGTAGCCTAATCAGCTAAATGCTTTCCAGTTTGAATGCTTATTGATTATGGTAGCTGATGTAAAATATCTGATATCTACATCTATTCCTACTGTGAATATGTGATGAACAACCACAATAAAGCCTAACCAATAGATATTATGGCTCATACCATGCCCATATAACCAAAGGGTTCTTTTTTATTAGAATAATATGTTACAATGTGTGAAATTATTCCAAAACCCGGTAAGATTAGAATATATACTTCAGGTGACCGAAGAATCCAGAACAAATTGGTATAGGATAGGGTCGCCGCCACCAGCAGGATCAAAGAAAGTAGGTATTTAAATTACGGTCAGTTAATAGTATGGTAATCCCTGCAGCTAGAACTGGAAAGAGAAAGTAAGAAGGACTGCAGTAATTAGGACGGATCACACAAACAGCGGAGTTTGATGTTAGTGCATGGCTGGCGGTTTCATATTTACAATTGTTGTAATAAAGTTAATAGCTCCAAGAATAGAAGAAATGCCTGCTAAATGTAGTGAAAAATGGTTAAATCTGCAGGCTCCTGGGTGGGATAAATTGCCTGCTAGGGGTGGGTAGACTGTCCAACCAGTGCCGAAAACTGCAACCTACAAGTTGATGATGCGGCATTGATGATGGGAGGGTGGTAGAAAGTCAGAAGCTTATGTTATTTATTCGAGGAAATGCTATATCGGGAGCGCCAATTATTAAAGGAATAAGTCAGTTCCCAAAACCCCAATCATAATTGGTATTACTATATGAAAAAAAAAAAATTATGATGAATGCGTGGGCAGTGACAATAACATTGTATACATGGTCGTCTTCTATTAGGCTTCCAAAGAGATTGTCCTAGTTCTGTTGGAGTGGGGGCTTAGGGCCTTCTACTGCCCCCGCCCATGCGCAAATAATAAATATAATGTCCCAATGTCTTTTGTGATTAGTTGAAAATAGTCAGCGGTTTATGAACATAGGTAGGGTAAAATGGCTGAGCAAGCATTGAACTGTAAATCTAAAGACAGAGAGTGAACCCTCTTTTACCAGCCCTGAGGTGAACTATCACATTGAATTGCAAATTCAAAAGCAGCTTCAATTCTGCCGGGGCTTCTCATAGCACCTAACGGCGGGGCGTGGAGTCGAAAGCCAGTTGATTAGGTTATTTAGCTGTTAACTAAATTTTTGTGGGTTAAAATCCCATCAATCTAGGAAGGGCTTAAGCAATTAAAGTAATTGGTTGTTCGAGTTGATGCAGAATAAAGTTTTGGTCTTATTTGTTGTGCAGAAATTAAGTAAATTTAACACTAAGAGGGCTTTGAAGGCTCTTGGTCTTATTAACCTAAATTTCTAAGTTATAAGTATTAGTGGAGTTAGGGAGTAGGAGGGCAAGGGTAGAAGACACTACAGAGTGGGGATAGAAATGATGATGGCTTTATATAATTTTAGTTTGCCAGTTGATTTTTGTGTTATTTGATGTTGGGAATATTGTCATTATCGAGATATAATATATTAAACATATATAAAAATATAAATTTATTAATATTATTAGCTTTATTATAAGGGAATTAATTAAGTTATCATTTTTAATAAGTTCTTGTATAATAGCTCATTTGGGGGAAAAACCTGTTAATTAGGTAGACCTCTAGGTTATTATTATTAGTGGAATAATGGGTATTGTTCATGCTAGTTTGTTTCAGGTATGTGATAGGGATAGGGTTGTTACATTTGAGGTTAAATAAAAAGTTATTAATGTAGAGATTGTTAGGAAAATATAAATAATTAAAGTTAATATAGTAATATTAGAGATGTAATATAATACTGCTATTATTCATCCTATGTGAGGTAATTGAAGAATAGGCTAGGATTTTGCGGAGTTGTGTTTAGTTGGAGTCCTCCTCAGCTGCCAATTATAATTGATAGAACTGAATTGTTAGAATCAAGTTTAAGTTTGTTGACGGAAAAATTTGAGGGGGGAGAATTGATATTGGGGCGAGTTTTTGTCACGTGAGAATAATTATAGCTGGGATTAAGGGATTCCTTGGGTAATTTCTGGGAGTCAGAAGTGGTGGGGCTATCTATTTTTATTGCTAGAGCAATAAATATTATTGTGGATAATATTTGGTTATAAGGAGGATTGATTGTTCATTGTTGAGTTAGTAGATTGTTAAGGAAAATGGATACTAATAGAAGTATTGATGCTGTGGCAAGATTAAAAATGTTGGTGGATGCCTCTGTGGAGCGGGGATTGGTACTTTTGGCAAGGATTGGTACGATGGCTAGTATATTGAAGATTTGACTATTCAGGCTGGAATCAATGTGAGCTTAAAATTGTGATTACGGTCCCTGTTAGAATGGTGAAGGAGATAATAAGGTGGGCTAGAGGATTAATGTTAGCACGGGAAGGATTAAACCAACATTTTCGGGGGTATGGGCCCGATAGCTTATTTAGCTGACCTTACTGTTTAGGATATGGTGTAATCGGTAGCACAGGAGAGTTTGAGTTCTCAGGTGTAGAGTTCAATCTGTAATCCTAGAAATAAGAGGGTTTGAACCTCTATAATTTACTCTATCAAAGTAACTCCTTTTATCAGACATATTTCTTATGTTTGGGGTGGGATGCCAGATGCTAGGATGGGTATTGAAACATATCATATACATAGTGCTAATGTAAGTGGTAAAAAGAGTTTTTCATAGTAGAGCTATTAATTGATCATGTAGCGGAAGCGGGGGTAGGCTGTTCGAATTCATAAAAATAAAATTGGTTAGTACCCAGAGGAGGGTTTTGGCTATAAAATTAATAATGCAAATGTTTCTGGAGCAATACGTAGCA

>17e438e6-a125-45b2-8196-fd44b432112d

TTGTACTTCGTTCAGTTACGTATTGCTTATTAAGATATATAGATATTTAGTCTATAATTTAACTTTGACAAAGTTATGTAATTATTTACACTAATATCTTATTAGCAGAAAAAGTCATAGGGTATATGGGATTGGCTTGAAACCAATTTTGGGGTTCAAATCCTTCCTTTTTCGTCTAAGTTGTAAGTTGCCTCTTCGAATGTGTGGTAGGAGGAGGGCAGCCGTATAATCATTCTAGATTGGTGGATAGTTGCTCAATGGCTAAGACTTTTCGTTTTGAAAGCTTCTCAAATTATAAAAACCATCAGGACTACTGCTGTAAATTAGTAAATGAACCATTAGATGAGACGATATTTCATGTAGTATATGCGTCTGGATAGTCTGAGTATCGTCGAGGTATTCCGGATAAGCCAAATTGTGGGAAGAAAGTTAAATTTACGCCGACAAATATAATGGTGGATTTTGGCATATGTTTGGTCAAGTATAACCTGAAAGGCGGGAATCGAAGAGGAATAAATCCCCCTATAATGGCAAATACTGCTCCTATTGATAACACATAGTGAAGTGAGGCTACTATGCATTATATATCATGTAATGATATCTAATGATGAGTTAGCTAACACAATTCCTGTTGAATCCACCCACAGTAAAAAAAGAAAAAATAAAGCCCAGGAGCTCAACTTGTACAGGAGATCATTTGATATTACCGCCGTGCAGTGTGGCTAATCAGCTAAATACTCCGGTGGGAGCCAGCAATGATTATGGTAGCTGATGTAAAAAATGCATGCGGTATCTATTCCACGTAGATATGTGATGGGCTCATACAATAAAGCCTAGGGAAACCAATAGATATTATGGCTCACCATGCCCATATAACCAAGGGTTCTTTTTTATTAGGGTAGCCATGTTACAATGTGTGAAATTATTCCAAAACCCGGTAGATTAGAATATATACTTCAAGGAGTGACCGAAGAATCAAAATAAATTTGGTATAGGATGGTCATATACCAACGGATCAAAAAAATGGCTGTTTAAATTACGGTCAATTCAACCAGTATGGTAATCCACCAGCTACGTCTAGGAAGAAAGTAAAGAAGGACTGCAGTATTAAGGACGGATCCACACAAACCAGCAGTTTGATGTTAGGTCATGGCTGGCGGTTTTCATATTTACAATTGTTGTAATAAAGTTAATAGCTCCAAGAATAGAAAAATGCCTGCTAAATGTAGTGAAAAAATGGTTAAAAAAATCTACAGAGGCTCCTGGGTGAGATCAATTGCCTGCAGGAGTTACAGGTGACTACAATCCAAACAGGTGCCGGCACCGAGCTTCTAGTTGATGAATTATAGCATTTAGGAGATGGGAAGGATGGTGAAGTCAAGCCATATTTATTTATTCGAAATCTAACATATCAGGGGCGCCATTAGTAAGAGATGAGTCAGTTCTAAACCTAATCATAGTGGTATTCCTATGAAAAAAATTATAGTGATGGTATGAGCGGTGACAATAGCATTCGTATCCACATGGTCGTCAGCTATAATGAGGCTTCCGGTTATTCCTAGTTCTGTTCAATTAGGAGGCTTAGGGCTGTACCTACTGCCCCCGCCCATGCCGCTCAAATAATAAAATATAATCCCAATGTCTTTGCGATTGAAGTTGAAAATAGTCAGCGGTTTATGAACATAGGTAAGGGTAAAATGGCTAGTGACGATGAACTTAAAATCTGAAAGGAACGAAGAGTGAACCCTCTTTGTAAACCACGGGGTGAACATCTTATTATGAATTGCAAATTCAAAAAGAACCTTCAATTCTGCCGGGCTTCTCCCGCCTTTTTTCCCTAACGGCGGGAGAAGTAGATTGAAGCGCCAGTTGATTAGGTTATTTAGCTGTTAACCTAGATGCACCGTGATTAATCCCATCAATCTGGGAAGGGCTTAGCTTAATTAAAGTAATTGATTTGCGTTCAGTTGATGCAGAATAAGTTTTGCAGTCCTTATTTGTTGTGCAGAAATTAA

>d83bcb4c-033e-4bc2-a7de-96ee9476347e

GTTGTATTACTTCGTTCGATTCTACATTACTTCAAATCCTTCCTTTTCGTCTAGGATTTAACGTAAGTTACCTCTTGAATGTGTGGTAAGGAGGAGGGCAGCCGTATAATCATTTCTAGATTGGTGGATAGTTGCTCAATGGCTAAGACTTTTCGTTTTGTGAAAGCTTTATGAATTATAAAACAAAATAAGGACTACTGCTGTAGGTGTTGACAAATGAACCGATAGATGAGACGATATTTCAAAGCCATAGTATGTACGTCTGGATAGTCTGGTATCGTCGAGGTATTCCGGATAAGCCAAGGAAGTGTTGTGGGAAGAAAGTTAAATTACGCCGACAAATATAATAATTGAGAATAGTTTTGGCATATGTTTGGTCAAGAGAGTATAGCCTGAAAAAGAGCGGGAATCAGTGAATAAATCCCCCTATAATGGCAAATACTGCTCCTATTGATAACACATAGTGAAAGTGGGCTACTACATAGTATGTATCATGTAATACAATATCTAATGATGAGTTAGCTAACACAATTCTGTTAGTCCACCCACGGTAAAAGAAAAATAAGGCGGGGCTCATAATATTGCGGGAGATCATTTGATATTACCGCCGTGCAGTGTGGCTAATCAGCTAAATACTTTTACTCCGGTGGGAATAGCAATGATTATGGTAGCTGATGTAAAATATGCACAGTATCTACGTCCCCAGCTCACATGAATATGTGATGGGCTCATACAATAAAGCTAGGAAACCAATAGATATTATGGCTCGCTTTTACTTATATAGCTTAAGGGTTCTTTTTTAGTAGACCAAACCTGTTACAATGTGTGGAAATTATTCAAACACGGTAAGATTAGAACCATACTTCGGGGTGACCGAAGAATCAGAATAAGTGTTGGTATAGGATAGGGTCGCCGCCACCAGCAGGATCAAGAAAGTAGTATTTAAGTACGGTCAGTTAATAGTATGGTAATCCCTGCAGCTAGGGCTGGAAGAAAGTAAAAGAAGGACTGCAGTAATTAGGACGGATCACACAAACAGCAGGAGTTTGATATTAGTCATAGCTGGCGGAAGTTCATATTTACAATTGTTGTAATAAAGTTAATAGCTCAAGAATAGAAGAAATGCCTGCTAAATGTAGTGAAAAATAGTTTAAATCTTCCAGGCTCCTGGGTGGAGTAATTATGCAAGGAATTGGGTAGAGCTGTCCAAACAGTGCAACTTCCTTCTAAATTTGATGATGCAAACAGTAGGAGTAGGAGATTGGTAGAAGTCAGAAGCTTATGTGTTGTTAGGAAATGCTATATCTAAGGAGCACTTAGTATTAAAGGAATAAGTCAGTTCCCCAAAACCCCCAATCATAATTGGTATTACTATGAAAAAAATTATGATGAATGCGTGGGCGGTGACAATAACATTGTATACATGAATTACGTCTTCTATTGGGCTTCCACAGGTTGTCCTAGTTCTGTTCGAATTAGGAGGCTTAGGGCTGTACCTACTGCCCCCGCCCATGCGCCAAATAATAAATATAATGTCCCAATGTCTTTGTGATTAGTTGAAAATAGTCAGCGGTTTGAACATATAGGTAGGGGTAAAATGGCTGAGCAAGCATTAGACTGTAAATCTTAAAGAAGACGGTGAACCTCTTTTACCAGCCCTGAAGGTGAACTGGTCACATTGAATTGCAAATTCAAAGAAGCAGCCAATTCTGCCGGGGCTTCTCCCTTTTTCTTTTCCCTAACGGCGAAGTAGATTGAAGCCAGTTGATTAGGTTATTTAGCTATTAA

>cfdfdbb9-1603-46a8-be27-8beccae5d422

GGTAGCCCACTTCGTTCAGTTACGTATTGCTGCAATAATTAAAAATACTAAGGGTACTCCTAGTATTGTTGGAATATTGAATGAGGCAAATAGATTTTGGTTCATTTTAGTTCTCAAGTTGTTTTATGTTTTGTGTTTCTACTAATTTTTGGTAGTGGGTAATAATGGAAAGTAAAGTTCAGTATTTTCAATTGTATAATATAAAATAAGGTAACAATTATAGATATAATTACTGGTCACAGGTAGAAATATTTAGTTGAGGCATTCACTATAGAGAGTTTGTTCTCTCAATCTTTAACTTAAAGGTTAATGCTAAGTTAACTACAGTGATACAATATATAAGTATGAAGCTCATACTTCGAAATCTTGGAAATAAATGAATTCTAGAACAATAGGTATAAAGCTGTGATTGGACCCGCAAATTTCCGAGCATTGTCCATAAAATAGGCCTGGTCGTATAGAGGCTAGTATGGCTTGGTTTAAACGTCCAGGAATTGCATCTGTTTTTACGCCTAGTGATGGTACGGCTCATAGGTGTAAGACGTCTTGTGATGAGATTAATATGCGAATATCCGCTTCTATAGGTAAAGTTGTTTCGGTTATCAACTTCCGAGTCGAAATTCCCTGGCTCAGGAAATATGTTGGCATAATGTAGAGTCAAATACTAAGTCTTCATAGTCGGAATATTCATAGGTTCAGTATCATTGGTGACCAATTGCTTTAAGGGTTAAATAAGGTTTATTAAATTCGTCTGTCATATATAGAATACGCAGTGATGGGAGGGCAATTGTAATTAGAATCAGTGCAGGAGAATAGTTCAGATTATTTCGATCTCTTGAGCATTTATGGTGCTAGTATGAGTGAGTTTTGTGGTAAGTATAAGGGAAATAATATATAAGACTAAGGAACTAATCAGGAAAATAATTATGAGTGCATGGTCGTGGAAAGCAATAAGTTCTTCTATGATAGGTGATGTGGCATTTGTAAACCTAGTTGAGCTGGTGTTGCTATTAAGATATATAGATATTTAGTCTATAATTTAACTTTGACAAAGTTATGTGAATTATTTACTAATATCTTATTGAAAAAGTCATAGGGTATATGGGATTGGCTTGAAACCAATTTTTGGGGTTCAAATCCTTCCTTTTTCGTCTAGGATTTAACGTAAGTTGCCTCTTCAGATGTGTGGTAAGGAGGAGGGCAGCCGTATAATCATTCTAGATTGGTGGATAGTTGCTCAATGGCTAAGACTTTTCGTTTTGAAGAGAAAGCTTCTCAAATTATAAAAACCATCAGGACTACTGCTGTAAGTGAAATAAATGAACCGATAGATGAGACGATATTTCATGTAGTATATGCGTCTGGATAGTCTGAGTATCGTCGAGGTATTCCGGATAAGCCAAGGAAGTGTTGTGGGAAGAAAGTTAAATTTACGCCGACAAATATAATGGTGAAGTGGATTTTGGCATATGTTTGGTCAAAGAGTATAGCCTGAAAAGAGCGGGAATCAGTGAATAAATCCCCCTATAATGGCAAATACTGCTCCTATTGATAACACATAGTGAAAGTGGGCTACTACATAGTATGTATCATGTAATACAATATCTAATGATGAGTTAGCTAACACAATTCCTGTTAGTCCGCACCCACGGTAAAAAGAAAAATAAAGCCCAGGGCTCATAATATTGCAGGGGAGATGCATTTGATATTACCGCCGTGCGGTGTGGCTAATCAGCTAAATACTTTTACTCCAGTGGGAATAGCAATGATTATGGTAGCTGATGTAAAATATGCACGAGTATCTACATCTATTCCTACTGTGAATATGTGATAGGCTCATACAATAAAGCCTAGGAAACCAATAGATATTATGGCTCATACCATGCCCATATAACCAAAGAGGTTCTTTTTTATTAGAATAATATGTTACAATGTGTGAAATTATTCCAAAACCCGGTAAGATTAGAATATATACTTCAGGGTGACCGAAGAATCAGAATAAGTGTTGGTATAGGATAGGGTCGCCGCCACCAGCAGGATCAAAAGTAGTATTTAAATTACGGTCAGTTAATAGTATGGTAATCCCTGCAGCTAGAACTGGAAGAGAAAGTAAAAGAAGGACTGCAGTAATTAGGACGGATCACACAAACAACGGAGTTTGATATTGAGTCATGGCTGGCGGTTTCATATTTACAATTGTTGTAATAAAGTTAATAGCTCCAAGAATAGAAGAAATGCCTGCTAAATGTAGTGAAAAAATGGTTAAATCTACAGAGGCTCCTGGGTGGGATAAATTGCCTGCTAGGGGGGTGGGTAGACTGTCCAACCAGTGCCGGCACCGGCTTCTAAAGTTGATGATGCAAGCAGTAGGAGTAGGGAGGGTGGTAGAAGTCAGAAGCTTATGTTATTTATTCGAGGAAATGCTATATCGGGAGCGCCAATTATTAAAGATAAGTCCAGTTCCCAAAACCCCCAATCATAATTGGTATTACTATGAAAAAAATTATGATGAATGCGTGGCGAGCAATAACATTGTATACATGGTCGTCTTCTATTAGGCTTCCGGGTTGTCCTAGTTCTGTTCGAATTAGGAGGCTAGGGCTGTACCTACTGCCCCGCCCATGCGCCAAATAATAAATATAATGTCCCAATGTCTTTGTGATTAGTTGAAAATAGTCAGCGGTTTATGAACATAGGTAGGGGTAAAATGGCTGAGCAAGCATTAGACTGTAAATCTAAAGACAGAGGAGTGAACCCTCTTTTTACCAGCCCTGAGGTGAACTATCACATTGAATTGCAAATTCAAAGAAGCAGCTTCAATTCTGCCGGGGCTTCTCCCGCCTTTTTTTCCCTAACGGCGGAGAAGTAGGTGGAAAGCCAGTTGATTAGGTTATTTAGCTGTTAACTAAATTTTTGTGGGTTAAAATCCCATCAATCTAGGAAGGGCTTAGCTTAATTAAAGTAGCTGATTTGCGTTCAGTTGATGCAGAATAAAGTTTTGCAGTCCTTATTTGTTGTGCAGAAATTAAGTAAAATTTACTAAGGGCTTTGAAGGCTCTTGGTCTTATTAACCTAAATTTCTAAGTTATAAGTATTAGTGGAGTTAGGGGTAGGAGACAGGTAGAAGACACTACAAGTGGGGATAGAAACGGTATTGGCTTTATATAATTTAGTTGCCAGTTGATTTTTGTGTTGTTGATGTTGGGAATATTGTCATTGAGATATAATATATTAAACGTATATAAAAATATAAATTTATTAATGTTAGTAAAGCTATTATAAGGGGAATAATTAAGTTATCATTTTTAATAGTTCTTGTATAATAGCTCATTTGGGGGGAAAAACCTGTTAGTGGGGGTAGACCTCCTAGGGATATTATTATTAATTAGGTCAATGGGTATTGTTCATGCTAGTTTGTTTCAGGTATGTGATAGGGATAGGGTTGTTACATTTGAGGTTAAATAAAAAGATTATTAATGTAGAATTGTTAGGAAAATATAAATAATTAAAGTTAATATAGTAATATTAGGGTCGTAATATAATACTGCTATTATTCATCCTATGTGAGTAATTGAAGAATAGGCTAGGATTTTTGCGGAGTTGTGTTTGGTTGAGTCCTCCTCAGCTGCCAATTATAATTGATAGAACTGAGATTGTTAGAATCAAGTTTAGGTTTGTTGACGGAAAAATTTGGAGGAGAATTGATATTGGGGCGAGTTTTTGTCGTGAGAATAATTATAGCTGGGATTAGAGGGATTCTTGGGTAGTCTGGGAGTCAAGTGAAGTGGGGCTATCCCTATTTTTATTGCTAGAGCAATAAATATTATTGTGGATAATATTTGGTTATATAAGGAGGATTGATTGTTCATTGTTGAGTTAGTAGATTGTTAAGGAAAATGGATACTAATAGAAGTATTGATGCTGTTGCTTGAATTAAAAAATATTTGGTGGATGCCTCTGTGGAGCGGGGATTGGTACTTTTGGCAAGGATTGGTACGATGGCTAGTATATTTAATTCTAAGCCTATTCAGGCTAGGAATCAATGTGAGCTTAAAATTGTGATTACGGTCACTGTTGAAATGGTGAAGGAGATAATAAGGTGGGCTAGAGGATTAATGTTAGCACGGGGAAGGATTAAACCAACATTTTCGGGGGTATGGGCCCGATAGCTTGTGACCTTACTGTTTAGGATATGGTGTAATCGGTAGCACGGAGAGTTTTGAGTTCTCAGGTGTAGGTTCAATTCCTGTAATCCTAGAAATAAGAGGGTTTGAACCTCTATAATTTACTCTATCAAAGTAACTCTTTTATCAGACATATTTCTTATGTTTGGGGTGGGATGCCAGATGCTAGGATGGGTATTGGAAACATATCATATACATAGTGCTAATGTAAGTGGTAAAGTTTTTTCATAGTAAGAATATTAATTGATCGTAGCGGAAGCGAGGTACTTAGGCTGTTCGAATTCATAAAAATAAAATTGGTTATTAGGAGGTTTTGGCTATAAAATTAATATTAAATGTTTCTGGTATAATTATATTGTAGGGTGTGGATAAGAAAAGATAGTGGTAGTTAGGGCATTTATTATAATAATATTTATATACTCTGCTATGAAGAATAAGGCGAATGAGCCTGCAGCGTATTCAATGTTGAAACCTGATACTAGTTCTGATTCGCCTTCTGTCAGGTCGAATGGAGCTCGATTAGTTTCTGCTAATGTC

>d01a2149-91b5-4ce6-9d64-4f75211715a0

ATTGTACTTCGTTCAATTTTACGTATTGCTAGTACTATTGAGCCAGCGATGGGGCTTCTACGTGAGCTTTTGGGAGTCATAGGTGTAACCCATATAGAAGGAGATTATTTTGACTATAAAAGCTATTATACATATCCCAATCATATAATACTATTGGTTCATGAAGTAAGTATTTCGTTAGAATAATTATTGTAAGTAGCTATCTTAGGGATCCCAAGTATTTAGATAGTATAGGAGGTAATTAATAGAAGGTAAGGATCCTGCTAGTGTTAACTTAGATAGAAAATATGAACCAGCATTAAGGCGTTCTGGTTGATACCCTCAGCAGATGATAATAATTAGGGTGGGGATTAGGGTAGTTTCAAATAGAATATAAAATAAAATTAGTTCGGTAGCTGAAAAAGTTATAATAAGTGAAATTTGTAATAGAATTAATATTGAGATATATAACGTTTTTCGTGGGGTGGGTTATTATATAAATGCTGTTGTGTTGCTAGAATTATTAGTGGCAGAAGTCAGGCTGTTAATATAGAAGTGGTGATGTTAGGTGAATCTGGGAAAAATTAGAGTGATAGGTTATGGGTTGTTTGGTGTGTGGAGCCAATATGAGGCGTATATACTGATTAGTAGACTACAGATTATTGTGTTGATTCATATTATACTGTTTACGAAGAAGTCGATATTATGATGGTTGGAAAATAAATTTTAACATTTATGAATTATTTTAGGTTTTGTACATAATCTAGGCCATACAGGTTGGAGACTAAAATTAACAAGGCTAGGCCTACTGCAGCTTCGCATGCGGCAAATACTAGGAAGGAATGAGGTATCATATACATTAATATGAAGTGCATATTGAAAGTTGTGAGTGTAATTATGATAAATAATGATAACTTCAAACATAGCAGTGATGATATTAGGTGAGATCGATAAATTAATAACCTAATAATGATATGAAATATGCTAGTATTACACGTATATAGATAAAAGGCATATTTGGTAAATGGGCTTTTTCATAATCTAATGAGTCGAAATCATTTGTTTTAATTAAACTATATGCAAATTCAATCTAATCCTTTTAGGTTTCACTCGTAGGCCTAACCCTATTAGAATAATAAGTAAGTCAAAAATTATATTTATTGTTAGAGTTAAATTATTTGTTTGGGTTGCCCATGAAATTTACTTGAGGGGTAGAAGTAGGGCAATTTCCCGAATCAAATAGGAGAAATGTGATGACGGAGTTAGGAAAAATTTTATGGAGAATGGTAAGTGGGCGGAGGTTGTAGGATCAAATCCACTCGTAGTTGTGCTTTTCTGTATAAATATTTAACTGTGGAAGTCAAAATGCTAATTGTGATAAAGTAAGGCCAAAGTAATGTTGGTAGTCATTACGGGCTAAAATTAAATTTATAATACTCTCTCTCCAGATGTCTATTCTTCATTCGAGGCCCATTGATTGAAGTCAATAATACTTTTTATACTAAGAGAGTAAGAGCCTCATCAATAGATGGAAATATAGAGGAAAGATATACACATATCAAAATGTCAATATCATGCAGCGGCTTCGGCGAAATGGTGGCTTAGATGTGAAGTGATATAATTGTTGGCGAGAAGTAACAGGTGAGGCGAGGAAAGTGGTTCCGATAATTAGTAGAAGGCCATGAAAGCCTGTAGCCATAAAAAATGTGGATCTATACTCGTCGGGAATAGTAAATGGGGCTTCAGAATATTCTGATATGGTAGGCAGGTGAAGTAGATCCCTAATATAATGGTTAGAAGTAGTGCTTGGAGTTGATTCTTTTCGGTTAGCTTCTATGAGGCTGTGATGTGCTCATGTAATTGTAACTCCTGATGCTAGTAGTACGGTTGTATTTAAGAGAGGGACTTCTATTGGGTTGAGGGATAATACCTGTAGGTGGTCATAGTCCCCTGTTTGTGGAAGTTGGGGCTAGACTAGAATGATAAAATGCTCAGAAGAAACCTGCAAGAAAGAAAAATTTCTGAAATAATAAATAGAATTATTCTTCGTAGGCCTTTTTGGACGGGTGCAGTAGTGACATGATATGTACTTTCTCAAATCTTACGTCGTCATCATTGAAATATTGTTATAGAACTAGCTAAGTAGACCTGCAGTGAGAAGCAGTGTGTGGTAAAAGTGGAATCATATAACTAGGCCGGATGTGAGAAGAAAAGCTGATAATGCTCCTGTTAGTGGTCAGAGGGCTTGGGTTGACTATGTGATAAGCATGGGTTTGTTTGAGTCATTATGAATTATCATGTAAGTACAGGCCCCACTAGGGTAAATACATGAGGCTTGAATTAAGGCTACATTTCTAGGGTAGTAATAAAATAATAATATGATGATGATTGTGGAAGTGGAGAGGTAAATTGATAGAAGAGTTAATGTTGTATCCCCAAGTAAATGCATTAATAGGTGACCTGCTGTAATATTGGCTGTTAATCGACGGCTAAGGCTACAGGTTGAATGAATAGACTAATTGTTTCAATAATAATTAATATAGGGATTAGTGGAATTGGTGTTCCTTGTGGTAAAATGAGCGAGGGATATTTTGTTTTTTTAAATGGAGGCCTATTAGTACAGTTGCTGCTCATAGAGGAATAGCTATGCCTAGATTTATTGACAGTTGGGTGGTTGGTGTAAATGCATATGGTGTAGATCCAAATCTTATTTAGGGCAATAAAAGTAGGGCTAGGAGTATAAGGTCAGGTTCGCCCTTTAGTGGTGTGGGTTAATATTATTTGTTTAAGTGTTAGTTGAATTAGCCATTGTTAGATTGAAAGTCAGTTACTTGAACTCTTAAGTTTTGGAGGATGAAATTAATGTGATAGAGTACAATAATTAAAAATACTAAGGGTACTCCTAGTATTGTTGGAATGAATGAGCAAAATACGATTTGGTTCATTTAGTTCTCAAGTTGTTTATGTTTTGTGTTTCTACTAATTTTGGTATTAGGTATAATGGAAAGTAAAGTTCAATTATTTTCAATTGTATAATATAAAATAAGGTAACAATTATAGATATAATTACTATTGATCACAGTGGAAAGTTTAGTTGAGGCATTCACTATAGGGGACATTCTCTCAATCTTTAACTTAAAAGGTTAATGCTAAGTTAGCTTTACAGTGATACAATATATAAGTATGAAGCTCATACTTCGAAATCTTGAAATAAATGAATTCTAGAACAATAGGTATAAAGCTGTGATTGGACCACCAAATTTCCGAGCATTGTCCATAAAATAGGCCTGGTCGTATAGAGCTAGTATGGCTTGGTTTAAACGTCCAGGAATTGCATCTCAAACACGCCTAGTGATGGTACGGCTCATGAGTGTAAGACGTCTTGTGATGAGATTAATATGCGAATATCCGCTTCTATGGTGAAGTTGTTCGATTTTATCAACTTCGAGGAGTCGAAATTCCCCTGGCTCAAGGAAATATGTTGGCATAATGTAAGAGTCAAATACTAGATCTTCATAGTGGAATATTCATAGGTTCAGTATCATTGGTGACCAATTGCTTTAAGGGTTAAATAAGGTTTATTAGTCGTCTGTCATATAGAATACGCAGTGATGGGAGGGTAATTGTAAATTAGAATCAGTGCAGGAAGAATAGTTCAGATTATTTTCAGATCTCTTGAGCATTTATGGTGCTAGTATGGTGGTGGTAAGTATAAGGGAAATAATATATAAGACTAAGGAACTAATCAGGTAACTATTATGATTATGGTATTGGAAAGCAATGAGTTCTTCTATGATAGGTGATGGCATTTTGTGTAAGCCTAGTTGAGCTGGTGTTGCTATTAAGATATATAGATATTTAGTCTATAATTTAACTTTGACAAAGTTTTATGTAATTATTTTACTAATATCTTATTGAAAAGTCATAGAGATTTATATGGGATTGAGCACGGAAACCAATTTTTGGGGTTCAAATCCTTCCTTTTCGTCTAAGTTGTAATGAGGTGCCTCTTCGAATGTGTGGTAAGGAGGAGGGCAGCCGTATAATCATTCTAGATTGGTGGATAGTTGCTCAATGGCTAAGACTTTTCACGTTTTGAAGAGAAAGCTTCTCAAATTATAAAAGCCATCAGGACTACTGCTGTAAAGTGAAATAAATGAACCGATAGATGGTATTTCATGTAGTATATGCGTCTGGATAGTCTGAGTATCGTCGAGGGTATTCCGGATAAGCCAAGGAAGTGTTGTGGAAGAAAGTTAAATTTACGCCGACAAAATATAATATAAGTGGTAGTTTTGTATATGTTTGGTCAAGAGTATAGCCTGAAAAGAGCGGGAATCAGTGAATAAATCCCCTATAACTGGCAAATACTGCTCCTATTGTTCCTTTTTACATAATGAGTGAGTGTTTACTACATAGTATGTATCATGTAATACAATATCTAATGATGAGTTAGCTAACACAATTCCTGTTAGTCCACCCACGTTTAAAAGAAAAATAAAGCCCAGGGCTCACTTAATATTGCGGGAGATCATTTGATATTACCGCCGTGCAGTGTGGCTAATCAGCTAAATACTTTTACTCCAGTGGGAATAGCAATGATTATGGTAGCTGATGTAAAATATACAGTATCTACATCTATTCTACATGTGAATATGTGATAAGGCTCATACAATAAAGAAGCCTAGGAAACCAATAGATATTATAAAACATCATACCATGCCCTTATAACCAAGGTTCTTTATTAGAATAATATGTTACAATGTGTGAGAGTATTCCATTAGGCTGTGGTCGAATATACTTCAGGGTGACCGAAGAATCAGAATAAGTGTTGGTATAGGATAGGGTCGCCGCCACCAGCAGGATCAAGAGAAAGTAGTATTTAAATTACGGTCAGTTAATAGTATGGTAATCCCTGCAGCTAGAACTGGAAAGGAAAGTAAGAAGGACTGCAGTAATTAGGACGGATCACACAAGCAATATGGAGTTGATATTGAGTCATGGCTGGCGGTTTCATATTTACAATTGTTGTATCGGAACCAATAGCTCAAGAATGAGACGATATATATAAATGTTAATTGAAAAAATGGTTAATTATCTACAGAGGCTCCTGGGTGGGATAAATTGCCTGAGTTGGGTAGACTGTCCAACCAGTGCCGGCTTTTCCTTCTAAAGTTGATGATGCAAGCAGTAGGAGTAGGGAGGGTGGTAGGAGTCAAACTTATGTTGAGCTTTATTCAGTATATCGAGGGCACTTAATTATTAAAGGAATAAGTCAGTTCCCAAAACTAATCATAATTGATACTATGAAAAAAATTATGATGAATGCGTGAGCAGTGACAATAACATTGTATACATGGTCGTCTTCTATTAGACTGGTTTATTCTAGTTCTGTTCAAGATTAGGAGGCTTAGGGCTGTACCTACTGCTGCCCATGCGCCAAATAATGTACTACAATATTTCCCATTCTCTTTGTGATTAGTTGAAAATAGTCAGCGGTTTGCTGAACATAGGTAAGAGGTAAAATGGCTGAGCAAGCATTAGACTGGCAAATCTAAAGGCGCAGAGGAGTGAACCCTCTTTTTACCAGCCCTGGTGAACTATCACATTGAATTGCAAATTCGAAGCAGCTTCAATTCTGCAGGGCTTCTGCGCCTTTTTCCCTGTTTCGGCGGGAGAAGTAGATTGAAAAAGCAGTTAGGTTATTTAGCTGTTAACTAAATTTTTGTGGGTTAAAATCCCATCAATCTAGGACTTAGCTTAATTAAAAGTAATTGATTTGCGTTCAGTTGATGCAGAATAAAGTTTGCAGTCCTTATTTGTTATTACTGAGAGCAAAATTAAAATTTACTTACTAAGGGCTTTGAAGGCTCTTGGTCTTGGCCTTTAAATTTCTAAGTTATAAGTATTGGTGGAGTTAGGGGTAGGAAGACAGGTAGAAGACACTACAAATTGGGGATAGAAACATTATTGGCTTTATATATTTAGTTGCCGGATTGATTTTTGTGTTATTTGATGTTGGGAATATTGTCATTGAGATATAATATATTAAACGTATATAAAAATATAAATTTATTAATGTTAAAGTAAGACTATTATAAGGGGAATAATTAAGTTATCATTTTTAATAAGTTCTTATTATAACTTAAACTCATTTGAACTGTTAGTGGGGTAGACTACTCCTAGGGATAATATTATTAAATTGGAATAATAGTGTGTTCATGCTAGTTTGTTTCAGGTATGTGATACGGGGATAGGTTATTTACATTTGGTTAAATAAAAGATTATTAATGTAGAGATTGTTAGGAAAATATAAATAATTAAGAGTAATATAGTAATATTAGGGTCGTAATATAATACTGCTATTATTCATCTATGTGAGTAATTGAAGGAATAGGCTAGGACGGAGTTGTGTTTGGTTGAGTCCTCTCGGCTGCGCAAGTATAATTGATGAGCTGAGATTGTTTAATCAAGTTTAGGTTTGTTGACAGGAAGAATTTGGAGGAGAATTGATATTGGGGCGAGTTTTTGTCACGTGAGAATAATTATAGCTTCCAGGATTAGAGGAGTTCCTTGGGTAATTTCTGGGAGTCAGAAGTGAAGTGGGGCTATCCTATTTTTATTGCTAGAGCAATAAATATTATTGTGGATAATATTTATAAGGAGGATTGATTGTTCATTGTTGGAGTTAGAAAGTAGATTGTTTAAGGAAAATGGATACTAATAGAAGTATTGATGCTGTTGCTTGAATTAAAAAATATTTGGTGGATGCCTCTGTGGAGCGAGGTTATATGGCAAGGATTGGTGATGGCTAGTATATTTAATTCTAAGCCTATTCGGAGCTAGGAATCAATGTGAGCTTAAAATTGTGATTACGGTCCCTGTTAGAATGGTGAAGGAGATAATAAAGGAGTGGAACATTAGAGGATTAATGTTAGCACGGGAAGGATTAAACCAACATTTTCGGGTATGGGTGATAAACATTAGCTGACCTTACTGTTTAGGATATGGTTATTAATCAGTAGCACGGAGAGTTTTGAGTTCTCAGGTGTGGGGTTCAATTCCTGTAATCTAAATAGGGTTGGAGCCTCATAATTTACTCTATCAAGTAACTCTTTTATCAGACATATTTCATATGTTTGGGGTGGGATTATCCAGATGCTAGGATGGGTATTGAAACATATCATATACATGATGTTAATGTAAGTGGTAAAAGTTTTTTCATAGTAAGAATATTAATTGATCGTAGCGGAAGCTTGAGGAATTGCATGTTCGAATTCATAAAATAAAGTAGTTGTGGAGGTTTGGCTACAAGAGTAATAGCTAAATGTTTCGTGGTATAAACATATTGTAGATTAGTAGATAAGAAAATAGTGGTAATTTAGGGCATTTATTATAATAATATTTATATACTCTGCTATGAAGAATAAGGCGAATGAGCCTGCAGCGTATTCAATGTTATTGTTCCTGGATTCCTGATTCATATCTGTCAGGTCGATGGAGCTCGATTAGTTTCTGCTAATGTGGAAATAAATCATATTATGGCTAGGGGCCATGATGGAAGTAGAAGTCAGTAGTGTTCTTGAGTGGTAATAAGCGATTTGTAGGAATTGAATGAGTAACTTATTAGTAGTGTTGATATTGAGATAATGGCAAGAGGGTGACCTCATATGAGATTGTTTGGGCTACAGCTGTGGTGCTCTAATTAGTGCGTAGTTTGAGTTGGATGCTCAGCCAGATCATAAAATTGAGTAAACAGCTAGGCTTGATGTTGCGAGGATAAATAGGAGACCAAGATTGAGAGTTGATAGAGGATGGTATGGGGAGTGGCGTTCATAGTAGAAGAGCAATGGAAAGAGCTAGGGTGGGGGCAGTTAAATATAAAGTTATGATTGGATGTGGTAGGTAATGAGTTTTCTTTTGTGAAGAGTTTTATGGCGTCAACTGATTGGTGAAGTATTCCGTAGGGGCCTACAATATTAAGGGCCTTTTCAGGTGTATATAACCCAGGATTTTTCGTTCTGCAAGTGTTTAGAAGACTGGCAATTAGGGCAGGTAAGTTAGTATTAGTAAATTAATTATATACGATGTTGTTAGAAGAATTGAACCTCTGATTATAAAGTTTAAGTTTTATGCAATTACCGGGCTCTGCCACCTTAACGGCCCTGTTCTTGGGCAAGATTATAATATGTGGTTGAGATGGAGGTCATCTGATTGTTGAGGCACTTTATAGTGGGCCCCATTTCTCTTATCCGCTCGTACTGAAGATATTTAAATAGATAAACCGACACCAAGTTGCTCCGGTCTGAACTCAGATCACATGGAGCTTTAATCGTTGAACAAACGAACCCTTAACAAGCTTCTGCACCATTAGGATGTCTTGATCCAACATCGAGGTCGTAAGCCCTGTATCGATATGGACTCTAAAATAGGACGCGCTGTTATCCTAGGGTAACGGTCCGTTGATCAAATTATTGTAGGTCAATAGGTGCTTGCTTACTTAGACTGGTAAGGTCTTGGTATATGGCACTTTGGAAGTCTCGAGGTCGCCCCAACCGAAATTTATAATACATTGACGGTAAATTAATGCCTGTTGGTTTACAAATCCTAGTTTGTATCATTAAATTAAAAGCTCCACAGAAGTCTTCTCGTCTTATTTATGTATATCCGCCTCTTCACGGATAGGTCAATTTCACTGATTAAAAGTAAGAACCCAGTTAAACCCTCGTGTGGCCATTCATACAAAGTCCCTATTTAGAGAACAAGTGATTATGCTACCTTTGCACGGTCAGGGTACCGCGGCCGTTGAACATATGTACACTGGGCAGGCAGTGCCTCTAATGCTAAATGTAGAGGTGATGTTTTTGGTAAGCAGGCGGGGTTAGAGCTTGCCGAGTTCTTTTACTTTTTTAATCTTTCATGATGCATACCTGTGTTGGGTTAACAGTTTAGTTAATTGATATTTTAATTTGTAGTTTATGATTATTAGGCTGTTAACTAACGGTAGTTGTTTTCGGTCTGAAATAAGCATGCACGGGAGAATAGATTCTATGTTACTTATACTAACATTATTGCTTCTATTAGATAATAGATTAATCCAATTTGTTTCAGGAGTTCGATAAAATTAGTAAGGTAATATGAGAATATTGAGCTTGAACAGCTTTCTTAATTGATGGCTGCTTTTAGGCCAATATGGGATTTATACTACTTACTCTCTGTAAAGGTTTTCCTAGGGTCTAAGAGCTGTCCCTCTTTAGACTAACAATAATTTACATGAGGATTTAATAATTCTGGGGATAAATAATTTAAAGTCGAACTACGTTCTGTCTTGGATAACCAGCTATCACCAGCTCGTTAGGTTTGTCGCCACTACCCATAGAATATTCCCACTATTTTGCCACATAGACAGATGTGCTCTTATAGCTGTCCTTGGGTAGCTCGCTTGGTTTCAGGTACTTTGTTGAAGTTCTCTTTATAAAACTACTTCTAGTTAATTCATTATGCAGAAGGTATAAGGACTCGTCTTTGCTTTTATATGCTTATTAGGTAATGTCTCCTTACATTTTATATCTATTGCGCCTAGTATGTAATTTCTATCACTATACTCTTACTATAGGTAAATGATTTAATTAATATAAATGAATAATATATTTAATGGAGAGTTGGGCTAGGATTAACCAAAATGATCATTTGTTATGACATCTTCCGGGTGTAAGCCGGATGCTTTGTGCAAGCTGTTGCATTTTGGTTTGTCCAAGCGCACTAGCAATACGTAACTA

>e2bb48d4-aaf8-44cb-a0a7-af1bb8e2c069

GGTATGCTTCGTTCAGTTACATATTACTCATATAGCACCCATATAACCAAAGGTTCTTTTTTATTAAAGATAATATGTTACAATGTGTGAAATTATTCCAAAGCCAGTAAGATTAGAATATATACTTCAGGGTGACCGAAGAATCAGAATAAGTGTTGGTGGACAGGTCCTTATACCAACAGGATCAAAGAAAGTAGTATTTAATTACGTTAAATTTAATAGTATGGTAATCCCTGCAGCTAGAACTGGAAGAGAAAGTAAAAGAAGGACTGCAGTAATTAGGACGGATCCACACAAACAACTTGGAGTTTGATATTGAAAGTCATGGCTGGCGGTTTCATATTTACAATTGTTGTAATAAAGTTAATAGCTCCAAGAATAGAAGAAATGCCTGCTAAATGTAGTGAAAAAATGGTTAAATCTGCAAGAGGCTCCTGGGTGGGATAAATTGCCTGCTGAATTGGGTGAGCTGTCAACCAGTGCCGGCACCGGCTTCTAAAGTTGATGATGCAAGCAGTAGGAGTAGGGAGGTGGTAGAAGTCGAAGCTTATGTTTATTCGAGAGAATGCTATATCGGGAGCGCCAATTATTAAAGGGAATAATCAGTTCCCAAAACCCCAATCATAATTGATATTACTATGAAAAATATGATGAATGCGTGGAAAGCGGTGACAATAACATTGTATACATGGTCGTCTTCTATTAGGCTTCCGGGTTGTCTAGTTCTGTTCGAATTAGAAGGGATAAGGGCTGTACCTGCTGCCCCCGCCCATGCGCCAAATAATAAATATAATGTCCCAATGTCTTTGTGATTAGTTGAAAATAGTCAGCGGTTTATGAACATAGCAATACGTAGCA

>00c47ca1-4902-4ed3-9e53-a0601d4aaadb

GCAACCCACTTCGTTCGAGATTACATGTGTGTGCTCTGGCGGTTTCATATTTACAATTGTTGTAATAAAGTTAATAAGCTTTCAAGAATAGAAGAAATGCCTGCTAAATGTAGTGAAAAAATGGTTAAATCTACAGAGGCTCCTGGGTGGGATAAATTGCCTGCTAGGGGTGGGTAGACTGTCCAGCAAGTGCCGGCACCGGCTTCTAAAGTTGATGATGCAAGCAGTAGGAGTAGGGAGGGCATATTTATTCGAGGAAATGCTATATCAGGAGCGCCAATTATTAAAGGAATAAGTCAGTTAAACCCCAATCATAATTGGTATTACTGTGAAAAAATTAACATGATGAATGCGTGGGCGGTGACAATAACATTGTATACACAAACAGATCGTCTTCTATTAGGCTTCAGGTTGTCTAGTTCTGTTCGAATTAGGAGGCTTAGGGCTGTACCTACTGCCCCGCCCATGCATAAATAATAAATATAATGTCTAGCTATGCTTTGTGATTAGTTGAAAATAGTCAACGGTTTATAGAACATAGGTAGGGGTAAAATGGCTGAGCAAGCATTAGACTGTAAATCTAAAGACAGAGGAGTGAACCCTCTTTTTACCAGCCCTGAGGTGAACTATCACATTGAATTGCAAATTCAAAGCAGCACAATTCTGCCGGGGCTTCTCCCGCCTTTTTTCCCTAACGGCGGGAAGTAGGTGAAAACAGTGATTGGGATTATTGTATTAACTAAATTTTGTGGGTTAAAATCCCATCAATCTAGGAAGGGCTTAGCTTAATTAAAGTAATTGATTTGCGTTCAGTTGATGCAGAATAAAGTTTTTGCAGTCCTTATTGACAATGTACGAAATTAGTAAATTTACTTACTAAGGGCTGAAAAGCAAGAATTCAGGTACTAGACGAAGAAGTTCAGTCTGATAAAGATGTTGCAGATACTTTATCTGTATTAAAGTCATGTTTGCAGTATGCAATTGACTCATCTGATAAGACCTCAAGAGCTTTAATTGAAGATTCAGTTCCAAAACACTTAATATCTTCGATTTTAGAAGAGGATTCTTTATCAAAACTAACTGATACCTTAACACAGATCTTAAAGCTTGATACACAAACTAAGTACATGCTCTTATCTCATTGAATTCTGTAGAGCGTGGACGCATTTTAATCAGTGCTTTAAATGGATATTCATATAAATCTGAACTTGAGAACAGAATCATTGATTTAGCAAAGAAATCAATGGAAAAGAATCAGAAAGAGTATTTCTTAGCAGAACAGTTAAAAAGCAATTGAAAGAACTTGGTGTATCTGTTTCAGAGGATGATGATGTTCAAGCTTTTATGACACGAAATGAGGGAGCTAAAGGCTCTGAATACGTCCATAAACGCATTGAAAGAAATCAGAAAACTAAACTCAATGAGCATTAACTCATCTGAGTACTCTGTTGTAAGAAACTATATTGATTGCTTGTTATCTTTACCTTGGTTTGACTGCTCGGTAAATCATGATTTAAAAGAAGGCTAAAGAGGTCTTAGACAGCAATACATGGCCCAGGTGTACTTCGTTCAGTTACGTATTGCTGGTGGAGTACATCCGCTGCTCGACACGCGCGACCCGCTGGGCGACCTCCAGCCGGCTGCGCTCGGGACGGGCGAGGCGAAGTTTGCAGTGATCGTGCGCTTCCCGACGGCAGGTGCCAGTATTACCCGTGCCTTACTGGACGGCGATGCTCCGCGGCGGCTCCTATGACGAGAAAGCGGCTCATCTCGACGGCCAAGCGCTCGAAGCTGCGCAACCACACCTCGCTGAAGTATCTGGTGGAAGTGGAGCGGGAGCTGCACAGGAGAAGATGCGCCGCGAGGTATATCACCGACCGGAGCAGATGCGCGAGCGCGCGTCCGGCGGAGGAGAACATCCGCGACTTCGTCAGCGGGGCGGAGAACGCCGGCAAGGTCTGGATCGGCCGCCTACTATACCAACCCCGACGGCCACGAGGTGCGCGACGTGAAGATCGCGGATGGAAGGCACGGCCAAGGAGGGCGGCGACTGGGGCGAGGACGTGCAGGCGGCGGCCAACACGGTCTGCTACGCCGATAACATACACCCCAACCTCGTCAGCGCCGTGCGGGCAAAGAGCCAGAGCAACAACTCGAGCTCTGACAAAGAACTCTTCGTCATGAAGCAGGCGCTCGAGATGGCCTGGCACGACCCTCTTTCTCGAACCGCTGCGCCTGAGGTGATGCGCTTCAACGGCTGGTATCTCGTGCGCCCCGCGGTGCCGATACAGCTCACGACCCTCGCGAGCACGCCGACGCGCGTGCACCGTCACGACCTGAAGCAATACGTAACTTCG

>46afd0f4-b9bd-4bca-8929-687888660237

CGGTGTGCTTCAATTGATTTACATGTTATCTCGAATCGAACGAGGGCCCGTTGATGAAGTCAATAATACTTTTTTATACTAAAGTAAAACTCATCGAATCAAAATATAGAGGAAAAGTCTTCACGTCTGAAATGTCACAATGTCATGAAGCCTGACCGAAATGGTGGCTAGATGTGAAGTGATATAATTGTTGGCGAAAGTAACAGGTGGTGAGGAAAGTGGTTCCGATAATTACGTGAAGGCCATGAAAGCCTGTAGCCATAAAAAATGTGGATCCATATGCTCCGTCGGGAATAGTAAGTAGAGCTTCAGAATATTCTGATAATTGTAGGCAGGTAGTAGATCCCTAATATAATGGTTAGGAGTAGTGCTTGGGGTTGATTCTTTTCGGTTAGCTTCTATGAGGCTGTGATACCATCATGTAGTGTAATACGATGCTAGTAGTACGGTTGTATTTAAGAGGGACTTCTTGGTTGAAGGTAATGTAACAGCAAGGTGTAGTCCACCTGGTACACGGCGTGAGTTTGAGGCTAGACTAGAATGATAAAATGCTCAGAAGAAACCTGCAAGAAAATTTCTGAAATAATAAATGAATTATTCCGTATCGTAGGCCTTTTTGGACGGGTGCAGTATGGTGGCCTTGATATGTACTTTCTCGTACTCATCACGTCATCATTGAAATATTGTTATAAGACAGCTAGTAGTTCAGTGAGAAGCAGTGTGTGGTAAGTGAGATGTAAACTAGGCCGGATGTGAGAAGAAAAGCTGATGTCTCATATTGGTGGTCAAAGACTTGGGTTGACTATGTGATGGTAACTTATGGATTTGTAGTGAGTCATTATGAATTACCATGTAGTGAAGGAAACACTAAGAGGGTGTATAGACTTGAATTAAAGGCTACAACTCTAGAGGTAATTAATGAATAATAATAATAGTAAACGATTGTGGAAGTGGAGGGTGGTGATAGAAGAGTTAATGTTGTATCCCCAAGTAAATACATTAATAGGTGACCTGCTGTAATATTGGCTGTTAATCGTACGGCTAAGGCAGTTGAATGAATAGTCCATTGTTTTCAATAATAAGTTAATAAGGATTAGTGGAGTGAATTGTTCCTTGTGGTAAAAAATGAGCGGGGATGATTTTGTTTTAAATCGGAGGCCTATTAGTACGGTGCTGCTCATAGAGGAATAGCTATGCCTAGATTTGTCCAAACCAGTTGGTTGGTTGGTGTAAATGCATATGGTGTAAGTCGAGAATGTTATTTAGAACCGATAAAAGAGAAATTAGGGCTAGGTATAAGGGATCAGGTTCGCCTTTAGTGGTGTGGGTTAATATTATTTGTTTAAGTGTTAGTTGAATTAGCCATTGTTGAATTGAAGAGAGTCGGTTGTTTGAATAAGTTTTTGGAGGATAAAATTAATGTAGTGGGAGTGCAATAATTAAAAATACTAAGTACTCCTAGTATTGTTGGAATATTGAATGAGCAAATAGAATTTTTGGTTCATTTTGATTTCCTCAAAGATTGTTTTATGTTTTGTGTTTCTACTAATTTTGGTAGTGGGTAATAATGGAAAGTAAAGTTCAGTATTTTTCAATTGTATAATATAAAATAAGGTAACAATTATAGATATAATTGCACTATTGGTCACATTGAAATATTTAGTTGAGGCATTCACTATAGAGTTTGTTCTCTCAATCTTTAACTTAAAAGGTTAATGCTAAGTTAGCTTTACAGTGATACAATATATAGAGTATGAAGCTCATACTTCGAAATCTTGAAATAAATGAATTCTAGAACAATAGGTATAAACTGTGATTGGACGCAAATTTCCGACATGTCCATAAATAAATCAGTCAGTGTAGGCACAAGTATGGCTAGTTTAAACGTCGAATTGCATCTGTTTTTACGCCTAGTGATGGTACGGCTCATAGGTGTAGACGTCTTGTGATGGAATTAATATGCGAATATCGCTTCTATAGGTAAAGTTGTTCGGTTATCAACTTCAGGAGGTCGAAATTTTACAAATAGGAAATATGTTGGCATAATGTAGAGTCGTACTAAGTCTTCATAGTCGGAATATTCATAGGTTCAGTATCATTGGTGACCAATTGCTTTAAAAGGTTAATAAGGTTTATTAATTTAATGTATACACTATCGATACGCAGTGATGGGAGGGCAATTGTAATTAGAATCGATTTTGGGAAGAACAAGTTCAAGTTATTTCAGTCTCTTGAGCATTTATGGTGCTAGTATGCGGTAGTTTAATGATGCTATTATGAAATGTGTATGAGCCAAGACTAATCGGAAAATAATTATGAGTGCATGGTCGTGGAAAGCAATAAGTTCTTCTATGATAGGTGATGTGGCATTTTGTAGAAACCTAGTTTGACTGGTGTTGCTATTAAGATATATAGATATTTAGTCTATAATTTAACTTTGATAAAGTTATATAATTATTGCAATATCTTATTGAAAAAGTCATAAGGTACATATGGGATTGGCTTGAAACCAATTTTGGGGGTTCAAATCCTTCTTGCTACGTCTAGGATTTAGCATCGTTGCCTCTTTAGAATATTATTACATTTATTGTTGGGAGGAGGCAGCCGTATAATCATTCTAGATTGGTGGATAGTTTGCTCAATGGCTAAGACTTTTCGTTTTTGAAGAAAGCTTCTCAAATTATAAAAACCATCAGGACTACTGCTGTAAAGTAAATGAACCGATAGATGAGACGATATTTCATGTAGTATATGCGTCTGGATAGTCCTGCGAGTATCGTCGAGGTATTCGGATTATTAAAGAAGTGTTGTGGGAAGAAAGTTAAATTTACATGACAAATATAATGGTGAAGTGGATTTTGGCATATGTTTGGTCAAGAGTATAGCCTGAAAAAGCCAGGAATCAGTGAATAAATCCCCCTATAATGGCAAATACTGCTCCTATTGATAACATAGTGGTGGAACTACATAGTATGTATCATATAATACAATATCTAATGATGATTATTAAACACATTATATTTAATTCACCCACGGTAAAAGAGAAAAATAAGGCCCAGAGCATAATATGCGGGAGATCATTTGATCTTCATAGTTATGAATCAATGGCTAATCAGCTAAATACTTTTACTCAGTAGAATAGCAATGATTATGGTAGCTGATGTAAAGGCGTATTACACAGTATCTACATCTATTCTCTGTGAATATGTGATGGGCTCATACAATAAAGCCTAGGAAACCAATAGAATGTTCATGGCTTTCATACCATGCCCATATAACCAAAGGGTTCTTTTTTATTAGAATAATATGTGCAATGTGTGAAGTGTATTACAAACCGAGTAAGATTAGAATATATACTTCAAGGTGACAAAGTCAAAGATAAGTGTTGGTATAGAGGATAGGGTCGCCTGCAACAGGATCAAAGAAAGTAGTATTTAAATTACGGTCAGTTAATAGTATGGTAATCCCTGCAGCTAGATTTGGAAGAAAGTAAAAGAGGACCCATTAGTTGGGTGGATCACACAAACAACAGGGAGTTTGATGTTAGTCATGGCTGGCGGTTTCATATTTACAATTGTTGTAATAGGTTGATAGCTCCAGAATAAGAAGAAATGCCTGCTAAATGTAGTGAAAAAATGGTTAAATCTACAGAGGCTTCCTGGGTGGGATAAGTACGCTGCTAGGGGTGGGTAGACTGTCAACCAGTGCCGGCACCGGCTTCTAAAGTTGATGATGCAAGCAGTAGGAGTAGGAGGGTGGTAGAAGTCAAACTTATGTTATTTATTCGAGGAAATGCTATATCAGGAGCGCCAATTATTAAAGGAATAAGTCAGTTCCCAAAACCCCCAATCATAATTGGTATTACTGTAAGAAAAAATTATGATGAATGCGTGGGCGGTGACAATAACATTGTATATGATTATGTCTTCTATTAGGCTTCCAGGTTGTCTGGTTCTGTTCCGAGGTGGGAGGCTTAGGGCTGTACCTACTGCCCCGCCCATGCTGTAATATTAAATATAATGTCCCAATGTCTTTGTGATTGATTTGAAAATAGTCATGGTTTATGAACATAGGTACCAGGGTAAAATGGCTGACAGCATTGAACTGCATAAATCTAAAGACAGAGGAGTGAACCCTCTTTTGCAGCCCTGAGTGGACTATGCATTCAAATTGCAGTCAAGAAACGCTCAATTCTGCCGGGGCTTCTCCGCCTTTTTTTACCTAATGGGAGAGATGAATTGGCTCAAGTTGGGTCAGTTATTTAGCTGTTGAAAAAACTGCTAATTTTGTAAGGTTAAAATCCCATCAATCTAGGAAGGAGCTTAACCAATTAAAGTAATTGATTTGCATTCAGTTGAAGTGAGAATAAAGTTTTGCAGTCCTTATTTGTTGTGCAGAAATTAGTAAAATTGCCGCTAAGGGCTTTGAAAGGCTCTTAGTCTTATTAACCTAAATTTCTAAGTTATAAGTATTAATGTGGAGTTAGGGGTGGAGAACGATGAAGACACCTACAAGTGGGGATAGAAACGGTATTGGCTTTATATAATTTAGTTGCCAGTTGATTTTTGTGTTATTTGATGTTGGGAATATTGTCATTGAGATATAATATATTAAACGTATATAAAAATATAAATTATTAATGTTAAATAGAAAATGACCCATAGAGAGAATAATTAGATTATATTTTAATAGTTCTTTGTATAATAGCTCATTTGGGGGGAAAAACCTGTTAGTGGGGGCAGAGCCTCTAAGGGTCAATAAATATTAATGGAATAATGGGTATTGTTCATGCTAGTTTGTTTCAGGTATGTGATAAGGATAGGGTTGTTACATTTGAGGTTAAATAAAAGATTATTAATGTAGAGATTGTTGAAAATATAAATAATTAAGTTATATATTAATATTAGGGTCGTAATATAATACTGCTATTATTCATCCTATGAGTAATTGAAGAATAGGCTAGGATTTTGCAGGAGTTGTGTTTGGTTGAGTCCTCTACTCAGCTGCCAATTATAATTGATAGAACTGAGATTGTTAGAATCAGATTTAGGTTTGTTGACGGAAAAATTTGGAGGAGAATTGATATTGGTGAGATTTTTTGTCACGTGAGAATAATTATAGCTGGGATTAGAGGGATTCGCTTGGGTAATTTCTGGGAGTCGAAGTGAAGTGGGGCTATCCTATTTTTGTGCGAGCAGTAAATATTATTGTGGATAATATTTGGTTATAAGGAGGATTGATTGTTCATTGTTGAGTTAGTAGATTATTGGAAAATGGATACTAATAGAAGTATTGATGCTGTTGCTTGAATTAAAATATTTGGTGGATGCCTCTGTGGAGCGAGATTGCGTTACTTTTGTAAGAAGTTAGCGTTAAGCTAGTACTCATTGAATTCTAAGCCTATTCAAGGCTAGGAATCAATGTGAGCTTAAAATTGTGATTACGGTCCCTGTTAGAATGGTGAAGGAGATAATAGGCGGGCTAGAGGATTAATGTTAGCACGGGAAGGTCAAATAGCATTTTCGGGGTATGGGCCCGATAGCTTATTATGACCTTACTGTTTAGGATATGGTGTAATCGGTAGCACGGAGAGTTTTGGGTTCTCAGGTGTAGGTTCAATTCCTGTAATCCTAGAAATAAGAGGGTTTGAACCTCTATAATTCTCTGTAAATGTCCTTTTATCGGGCATATTTCTTATGTTTGGGGTGGGATGCCAGATGCTAGGATGGGTATTGAAACATATCATATACATAGTGCTAATGTAAGTGGTAAAAAGTTTTTTCATAGTAAATGTTAATTGATCGTAGCGGAAGCGGGGGTAGGCTGTTCGAATTCATAAAAATAAAGTGGTTGAAGTAGAAGGTACTGTTATAATTAGTAATTAGAAAATGTTTTGCATAAATTATGGTGTGGATGAAAATAGTGGTAGTTAGGGCATTTATTTATAAATATGTTACATCTGCTTTATTGAATAAGGTGAAGCAGCCTGCAGCGTGTTAGTGTTGAACTGATATGAATTTATACTAATTTACCACCCGGCAAGTTCTAGATGCGGAGCTGATATTTCTGCTAATGTGAAATAAATCATATTATGTGCAGGGCCATGATGGAAGTAGAAGTCAGTAGTGTTCTTGAGTGGTAATAAGCGATTGCTTGTAGATTGAATGAGCCGCTTATTAGTAGTGTTGATGGTAAGATAATGGCAAAGGGAGTGACCTCATATGAGATTGTTTGACTACAGCTCGTAGTGCGCCAATTAGTGCGTAGTTTGAGTTGGATGCCTCCAATAAAGATCATAAAATTGATAAACAATAGGCTTGATGTTGCGAGGATAAATAGGAGACCAAGATTGAAGTTGATGAAGGATATGGTATGGGGAGTGGCGTTCATAGTAGAAGAGCAATGGAAAGAGCTGGATAGGGGCAGTTAAATATAAGTTATGGTGGATGTGGTAGGTAATAAGGGTTCTTTTGTGAAGAGTTTTATGGCGTCAGCGATTGGTTGAAGTATTCCGTAGGGGCCTACAATATTAGGGCCTTTTCGGGGTAACTTATATAACTTAGGATTTTTCGTTCTGTGAGTGTTAGAAAGGCTATGGCAATTGAGGCAGGTGAATCTGGTATTAGTAAATTAATTGTATCCATATTTGTTAAGAGAAATTGAACCTCTGATTATAAAGTTTTAAGTTTTATGCAATAGCAATACGTAA

>a7322061-69f7-4e46-a7d2-ad29c0ad7093

GTGTGCTTCGTTCAGTTACGTATTGCTTAGTTTGGTTAATACCAATTGGCGTCACTATTATAGGAGCCTAGTTGGCTTGAGGTAATAAAGGCTACAATCTTTAAGTGTTTTATTGTTAGGGCACAAATTGCTGTAATAGGGTGATTACTTTGAAAGTGATGTGCTATTGTTTGGATAAATTGATTATTTTCTGGTAAGGATAAAAGCGGATAATTAAAGGTTGCAGCAACTATTGTGCCCAGAGTGCAGTAGTGCTGACACTGGGGTAGGCCCTTCTATATGGAGGTGTCCCACGGATGGGCCCGTTGAGCAGATTTTCCTGTTGCTGCTAAAAGTAAGGCTTGTTAGGGGAAAGAGTTAGGAGTATAATCTAGTATAAATGTTGTTGAAAGTCTCATGAGTTATGATATAAAAAGAACCGTTATCCCGCTTTAAATAAAGCCATTATCTCAAATCCGGTTATATAAAATTGCAGGAGCTGCTGTGTTGGCATCTGTTCGTCCGTACCATCAGCTAATTAATAGAAATGATATAATACCTATCCTCCCATCGATAAAGTTGAAATAGATGTTGCGGTAATTAGAATTAATATTTGTAATTAAGAAAATGAAAAGTATTTAAGGAATTGAATTAATGTTTAGATCTGAGCTTATATACCAGCTAAATTTGAGAATTCTACAATTGATCAGGTGACAAAGTGCTTTACGGAGACCGTATTGTGGAGAAAAATCTATTTTAAAACAGTGATAGCGGTAGTTTGAATAGTAGTTCAATGTCAGTTTAAATCATCAATTCTTGGCCTGTAAAGATGTATATTGCTTTATACATAGGATGCTGGCAGTGAGGGCATAAATAATAGCTAGTTTTACGTAATAAGGATAGCAGGAGGCTTTATGTAAATTAACTATAGTTATTAAGATTGGTACTGGTAATTGGAATAACAGTGAATATAATTATTGAGGTATATTTTTACTTTTATTTGGGTTACGCAATATTTTGGTTCCCTGAAAGCAATGGATAACTACTATCCTTTAAAAGTTAAGGGAAGCCAAGTTGTTGAAGCTGAGCATGAATTAGCAGTTCTTGCATACTTTCTCGGTAGTTAAGTTATGGGCTTCTATCATTAGAATTCACAATCTAATGTTTTATTAAACTATAACTACAGAACGTCAAACCTATAATTATCTTGGGGTTTGTAGTTAATAGTAGGATGGTGATTAAATGTATTATTATTAGTACATTTTCTCTCACGTGTAGAGGGTTTGATGCTCTTCAGTGCTTACGTGCGTTATGGCCCTCGTTGTGTTGAGGTAAATATGTGAAGTGGTAGAGGCTGTAATTAATGTTTGAAACCTGTAAATATGGTGGCAAAAATTAGATCAAAGAAAGAAGCTAAAGATTGTTAATAGTTCACCTAATTAGATTAATAGTTGGGGGAAATAAATTTGCTGGGTTAGCTAGAAGTCATCAAAGTGCTAGAAGGGAAATAATGTTTGAAGGCCTCGTGTAATGTTATAGTTCATTTGTAGTCGTTCGTAGTTAGTATTTGCTAAACAAAATAGGGATAGACAAGTCGGGAGATTAGTAGTATTATTGCACCGGTAAGGCTTCATGAGGTTTTGAATTATAATAGCTAAAATAACAAGTGCTATGTGGCCTGACGGAGGAGTAAATGATAAGTGATTTTAGGTCGGCTTGTCGTAAGCAGATGGAGCTTGTTATCACTATCCCTCGAGGATATTGATAAAAGAAAGGTAGCTTATTTCTGTTTAGTGGATTGAGGATAGGGGAGTAATTCGCACTATACCATACCTCCTAGTTTTAGTAAGATTGCTGCAAGTACTATTGAGCCAGCGATGGGAGAAGCCTACGTGAGCTTTGGGAGTCATAGGTGTAACCCATAAGGGTATATGCATTTCATAAAAGCTATTATACATCCAATCATATAATACTATTGGTTCATGAAATGTAGTATTTCGTTAAATTAATAATTATTGTAAGTATGCTTAGGGATCCCAAAGTATTTAGATAGTATAGGATAATTAATAGAAGGTAAAAAAGGATCCTTACTAGTGTGTAGGAATAGAAAATATGAACCAGCATTAAGGCGTTCTGGTTGATACCCTCCCACAACAGATAATGAATTAGGGTGGGATTAGGAGCAGTTTCAAATAGAATATAAAATAAAATTAGTTCGATATGAAAAAGGGTTATAATAAGTAATTTGTAATAGAATTAATATTGAGATATATAGTTTTTTCGTGGGGTGGGGTTATTATATAAATGCTGTTGTTATTGTTGCTAGAATTATTAGTGGCAGAAGTCAGGCTGTTAATATAAGAAGTGGTGATGTTAGTAGATCTGAGGGAAAAAATCAGTGATAAAATTGCATGAGTTGTTTGGTGTGTGGAGTAATATGAGGGCGTATATACTGATTAATTACAGATTATTGTGTTGATTCATATTATATAATTTTTGAAAGTCATATGGTAGAAGTATTGATGGTTGGAAAAAATAAATTTTAACATTAGATAAGTTTAGGTTTTGTACATAATCTAGGCCATATAGGTTGGAGACTAAAATTAACAAGGCTAGGCCTACTACAGCTTCACGCGCAGCAAATACTAGGAGAATGAGGTATCATACATTAATATGAAGTGCATATTGAAAGTTGTGAGTATGATATGATAAATAATGATAATATTATGCCTTCAAACATAGCAGTGATGATATTAGGTGAGATCGATAAATTAATAACCCTAATAATGATATGAAATATACTAGTATTACATTAATATAGATAAAAGGCATATTTAGTAAATGGAGCTTTTCATAATCTAATGAGTCGAAATCATTTGTTTTAATTAAACTATATACCAATTCAACCCAATCTAATCCTTTTTTGGGACCACTCGTAGGCTAACCCTAAAGTAGAATAATAAGTAGGGTAAAAGTGTATATTATTTGTTAGAGTTTAGAATTATTTGTTTGGGTTTGTACATGGTAGAATTATTAGAAGTAAGGAGCAATTTCAGATCAAATAGGAGAAATGTGATAGCGATTAGGAAAAATTTTATGGAGAATGGTAAGTGGGCGAGGTTGTAGGATCAAATCCGCACTCATGGAAGGTTTGTGCTTTTCTGTATAAATATTTAATTGTGGAAGTCAAAATGTAATTTGTGATAAGTAAGGCCAAAGTAATGTTAGTCATTGGAGCTAAAATTAAATTTATAATACTCTCTCTCAGATCATTCGAGCCCGTTGATTGAAGTCAATAATACTTTTTATACTAAGAGAGTAAAGAGCCTCATCAATGAGTCGATATAGAGGAAAAGTCATACTACATCTACGAAATGTCAATATCATGCAGCGGCTGACGAAATGGTGGCTAGATGTCAGTATAATTATTTGGCGAAAGTAACAGGTGGTGAGGAAAGAAATGGTTCCGCGATGAATACGTGATATGAAAGCCTGTAGCCATAAAAATGTGGATCCACTATCACTCCGTCAGGGGTAGTAAATGGGGCTTCAAGTGTCTGATAATTGTAGGCAGGTAAGTAGATCCCTAATATAATGGTTAGGAGTAATGCCAGGTTGAGTTCTTTTCAGTTGGCTTCTGTAAGGCTGTGATGTGCTCATGTAATTGTAACTCCTGATGATGCTAATGATGCAGTTGTATTTAAGAGGGACTTCTATTGAGTTGAGGAAATGTCTGTAGGTGGTCATAGTCCCCCTGTTTGTGGAGTTAGGCTAGACTAAATTTGATAAAATGCTCAGAAGAAACCTGCAAAGAAGAAAATTTCTGAAATAATAAATGAATTATTCGTATCGTAGGCCTTTTTGGACAGGTGCAGTATGGTGGCCTTGATATGTACTTATATTACTGCATCACGTCATCATTGAAATATTGTTATAGAACTAGCTAGTAGACCTGCAGTGAGAAAGCAGTGTGTGGTAAAAGTGGAATCATATAACTAGGCCGGATGTGAAAGAAAAGCTGATAATGCTCCTGTTGATGGTCAAAGGCTTGGGTTGACTATGTGATAAGCATGGGTTTGGTGAGTCATTATGAATTATCATGCTACAGGCTTACTAAGGGTAAATACATGGAAACTTAGTTAAGGCTACACCAACTCTAGGGTAGTGTAAAAAAATAATAATAATGATAGTGATTGTGGAAGTGGAGAGGTAAATTGATAGAAAGAGTTAATGTTGTATCCCCAAAGCCGTGCATTTAATAGGTGACCTGCTGTAATATTGGCTGTTAATCCGTACAGCTAAAGGCTACAGGTTGAATAGACGAACTAATTGTTTCAATAATAATTAATATATGAGTTAGTAAGGGTGGTGTTCCTTGTGGTAAAAAATAGGCGAGGATGATTTGTTTAAATCGGACCTATTAGTACAGTTGCTGCTCATGAGAGGAGCCATTGTACCTAGATTTATTGACAGTTGGGTGGTTGGTGTAAATGCATATGGTGTAGGGTCAAAATGTTATTTAGAACGAAGGCATCAAAAGAAATTAGGGCTAGGAGTATAAGGGATCAGGTTCATGTTGAGTGGTGTGGGTTGGTATTATTTGTTTGAAATTGTTGGTTGAATTAACCATTGTTGAATTGAAGAAGTCGGTTGTTGAATAAGTTTTGGAGGATAAAATTAATGTAGTGGGGAGTGCAATAATTAAAAATACTAAGGGTACTCTAGTATTGTTGGAATGTTGAATAGAGGAACTTGAAATAGATTTTGGTTCATTTAGTTCTCAAGTTGTTTTATGTTTTTGTGTTTCTTTACTAATTTTGGTAGTGGGTAATAATGGAAAGTAAAGTTCAGTGTTTTCAATTGTATAGTATAAAATAAGGTAACAATTATAGATATAATTTACTATTTGGTCACGGTGGAAATATTTAGTTGAGGCATTCACTATAGAGTTTGTTCTCTCAATCTTTAACTTAAAAGGTTAATGCTAAGATTAACTTTACAATTACAATATATAAGTATGAAGCTTCCATACTTCGAAATCTTGGAAATAAATGAATTCTAGAACAATAGGTATAAAGCTGTGATTGGACCCGCCGTTCGAGCATTGTCCATAAAATAGGCCTGGTCGTATAGAGGCTAGTATGGCTTGGTTTAAACGTCAGAATTGCATCTGTTTTACGCCTAGTGATGGTACAACTCATAGTGTAAGACGTCTTGTGATGAAAAGATGTTTAATATGCGAATATCCGCTTCTATAGGTAAAGTTGTTCAGTTATCAGCGAGTCGAAATTCTAATCAAGGAAATATATTTGGCATAATGTAAGTCAAATACTAAGTCTTCATAGTCGGAATATTCATAGGTTCAATATCGATGGTGACCAATTTGCTTTAAGGGTTAAATAGGTTTGTGGTTAAATCTGTCGCTTACTTAGAATACGCAGTGATGGGAGGGCAATTGTAATTAGAATCAATTGCAGGAGAATAGTTCAGATTATTTCGATCTCTTGAGCATTTATGGTGCTATGAGTGAGTTTTGTGGTAAGTATAAGGAAATAATATATAAGACTAAGGAACTAATCAGGAAAATAATTATGAGTGCATGGTCGTGAAGCAATAGGTTAATATGATAGGTGATGTGGCGTGTTGTAAACCTGGTTGAGCTGAAATTTGTTGCTATTAGATATATGGATATTTAGTCTATAATTTAACTTTGACAAAGTTATGTAATTATTTACTAATATCTTATTGAAAAAGATCATAGGGTATATGGGATTGGCAAGAGCCAATTTTAAGGGGGTTTCAAAATCCTTCACTTTAAATTACAGGATTTAACGTAAGTTGCCTCTTCGAATGTGTGGTAAGGAGGAGGGCAGCCGTAATCATTATTCTAGATTGTCGGATAGTTGCTCAATGGCTGAACTTTTCGTTTTGAAGAGAAAGCTTCTCAAATTATAAAAACCATCAGGACTAACATGGTGAAATAAATGAACCGATAGATAGAACTGATATTTCACATGTAGTATATGCGTCTGAGATGGTCTGGTATCGAGGTAATTCCGGATAAGCCAAGGAAGTGTTGTCAGGAAGAAAGTTAAGTTTATCGACAAATATAGTAGTGAAGTGGATTTTGGCATATGTTTGGTCAAGAGTATAGCCTGAAAGAGCGGGAATCAGTGAATAAATCCCTATAATGGCGGAAATACTGCTCCTATTGATAACACATAGTGAAAGTGGGCTACTACATAGTATGTATCATGTAATACAATATCTAATGATGAGTTAGCTAACACAATTCCTGTTGAGTCCACCCACGGTAAAGAAAAATGAAGCAGACTCTATAATATTGCGGGAGTCATTTTGATATTACCGCCGTGCAGTGTGGCTAATCAGCTAAATACTTTACTCAGTGGGAATAGCAATGATTATGGTAGCTGATGTAAAATATGCACAGTATCTACATCTATTCCCTGCAACTTAGATATGTGATGGAGGCTCATACAATAAAACTAGGAAACCAATAGATATTATGGCTCATACCATTGCCCATATAACCAAAGGGTTCTTTTTTTATTAGAATAATATGTTACAATGTGTGAAATTATTCCAAAACCCGATATTGGGTCAATATACTTCAGGGTGACCGAAGAATCAGAATAAGTGTTGGTATAGGACCGGGTCGCCGCCCACCAGCAGGATCGAAAGTAGTATTTAAATTACGGTCAGTTAATAGTATGGTAATCCCTGCAGCTAGAACTGGAAAGAAAGTAAAAGAAGGACTTGCAGTAATTAAGGACGGATCACACAAACAACGGAGTTTTGATATTAGTGCAATGGCAGTTTCATATTTGAAATTGTTTAATAAAGTTAATAGCTCAGAATAGAAGAAATGCCTGCTAAATGTAGTGAAAAAATGGTTAAATCTCAAACTCCTGGGTGGGATAAATTGCCTGCTAGGGTGGGTAGACTACATCCTTCGGTGCCGGCACCAACACAAAGTTGATGATGCAAGCAGTAGGGTAGGGAGAATTGGTAGAAGTCAAGCTGTGTTGTTTATTCCGATGCTATATCGGGGCGCAATTATTAAAGATATAAGTCAGTTCCAAGAAAAGAAAAGCCCCCAATCATAATTGGTATTACTATGAAAAAAATTATGATATGAATGCGTGGGCAGTGACAATAACATTAGCATACGTGATGGCTACTTCTATTAGGCCGGGTTGTCCTAGTTCTGTTCGAATTAGGAAGCAAGGCTGTACCTACTGCTTTACCCGTACGCCAAATAATACGTATAATGTCCCCAATGTCTTTGTGATTAGTTGAAAATAGTCAGCGGTTTATGAACATAGGTAGGGGTAAAATGGCTGAGCAAGCATTGGAGCGTAAATCTAAAGACGGAGAACCCTCTTTCTTGAAGGTGAACTATCGCATTGAATTGCAAGTTAAGAAAGCAAGCAATACGTAA

>e83f5635-639b-4d3f-a444-322b0c9a3d4c

AAGCGTATTTGGATTCAGTTACGTATTGCTAAGCAGTGTGTGGTTCAAAAGTGGAATCATATAACTAGGCCGGATGTGAGAAGAAAAGCTGATAATGCTCCTGTTAGTGGTCAAGGGCTTGGGTTGACTATGTGATAAGCATGGGTTTGGTGAGTCATTATGAATTATCATGTAAGTACAGGCTTACTAAGAGGTAAATACATAGGCTTGAATTAAGGCTACACCCAACTCTAGAGGTGTTAATAAAATAATAATAATGATAGTGATTGTGGAAGTGGAGAGGTAAATTGATAGAAGAGTTAATGTTGTATCCCCAAGTAAATGCATTAATAGGTGACCTGCTGTAATATTGGCTACTGTTAATCGTACGGCTAAGGCTGTAGGTTGAATGAATAGATGGTGTTTCAATAATAATTAATATAGGGATTAGTGGAATTGGTGTTCCTTGTGGTAAAAAATGAGCGAGGGATGATTTTGTTTCGTCAGGCCTATTAGTACAGTTGCTGCTCATAGAGGAATAGCTATGCCTAGATTTATTGACAGTTGGGTGGTTGGTGTAAATGCATATGGTGTAAGTCCGAGAATGTTATTTAGAACCAATAAAAGTGGAGCTAGGGTAAGGGATCAGGTTCGCCCTTTAGTGGTGTGGGTTAATATTATTTGTTTAAGTGTTAGTTGAATTAGCCATTGTTGAATTGAAGAGAGTCGGTTGTTGAATAAGTTTTGGAGGATAAAATTAATGTAGTGGGGAGTTGTAGTAATTAAAAATACTAAGGGTACTCCTAGTATTGTTGGAATATTGAATGAGGCAAATAGATTTTAGTTCATTTAGTTCTCAAGTTGTTTTATGTTTTTGTGTTTCTACTAATTTTGGTAGTGGGTAATAATGGAAAGTAAGTTCAGTATTTTCCGTTGTATAATATAAAATAAGGTAACAATTATAGATATACTGGTCACGGTGAAATGTTTAGTTGAGGCATTCACTATAGAGAGTTTGTTCTCAATCTTTAACTTAAAAGGTTAATGCTAAGTTAGCTTTTTACAGTGATACAATATATAGAGTATGACTCATACTTCGAAATCTTGGAAATAAATGAATTCTAGAACAATAGGTACCAGCTGTGATTGGACCCGCAAATTTCCACGAGCATTGTCCATAAAATAGGCCTGGTCGTATAGAGGCTAATATGGCTTGGTTTAAACGTCCAGGAATTGCATCTGTTTTTACGCCTAGTGATGGTACGCTCATGAGTGTAAGACGTCTTGTGATGAGATTAATATGCGAATATCCGCTTCTATAGGTAAAGTTGTTCGGTTATCCAACTTCGAGGAGTCGAAATTCCCCTGGCTCAAGGAAATATGTTGGCATAATGTAGAGTCAAATACTAAGTCTTCATAGTCGGAATATTCACCTTAGGTTCAGTATCATTGGTGACCAATTGCTTTAAAAAAAAAGGGTTGACAGGTTTATTAAATTCGTCTGTCAATACATCTTAGAATACGCAGTGATGGGAGGGCAATTGTAATTAGAATCAGTGCAGGAGAATAGTTCAGATTATTTCGATCTCTTGAGCATTTACATAGTGCGCTGAAATATGAGTGAGTTTTGTGGTAAGTATAAGGAAATAATATATAAGACTAAGGAACTAATCAGGAAAATAATTATGAGTGCATGGTCGTGGAAAGCAATAAGTTCTTCTATGATAGGTGATGTGGCATTGTAAAACTGGTTGAGCTGGTGTTGCTATTAAGATATAGATATTTAGTCTATAATTTGACTTTGACAAAGTTATGTAATTATTTTACTAATATCTTATTGAAAAAGTCATAGGGTATATGGGATTGGCTTGAAACCAATTTTTGGGGGTTCAAATCCTTCCTTTTTCGTCTAGGATTTAACGTAAGTTGCCTCTTCGAATGTGTGGTAAGGAGGAGGGCAGCCGTATAATCATTCTAGATTGGTGGATAGTTGCTCAATGGCTAAGACTTTCGTTTTGAAGAGAAAGCTTCTCAAATTATAAAAACCATCAGGACTACTGCTGTAAGTGAAATAAATGAACCGATAGATGAGACGATATTTCATGTAGTATATGCGTCTGGATAGTCTGAGTATCGTCGAGGTATTCCGGATAAGCCAAGGAAGTGTTGTGGGAAGAAAGTTAAATTTACGCCGACAAATATAATGGTGAAGTGGATTTTGGCATATGTTTGGTCAAGAGTATAGCCTGAAAAGAGCGGGAATCAGTGAATAAATCCCCTATAATGGCAAATACTGCTCCTATTGATGCCACATAGTGAAAGTGGGCTACTACATAGTATGTATCATGTAATACAATATCTAATGATGAGTTAGCTAACACAATTCCTGTTAGTCCACCCACGGTAAAGAGAAAAATAAAGCCCAGGGCTCATAATATTGCGGAGATCATTTGATATTACCGCCGTGCAGTGGCTAATCAGCTAAATACTTACTCAGTGGGAATAGCAATGATTATGGTAGCTGATGTAAAATATGCGAGTATCTACATCTATTCCTGAATATGTGATGGGCTCATACAATAAAGCCTGAAACCAATAGATATTATGACCATACCATGCCCATATAACCAAAGGGTTCTTTTTTTATTAGAATAATATGTTACAATGTGTGAAGTATTCCAAAACCAGTATAGATTAGAATATATACTTCAGGGTGACCGAAGAATCAGAATAAGTGTTGGTATAGGATAGGGTCGCCGCCACCAGCAGGATCCAAAGAAAGTAGTATTTAAATTACGGTCAGTTAATAGTATGGTAATCCCTGCAGCTAGAACTGGAAGAAAGTAAAAGAAGGACTAGCAGTAATTAGGGCGGATCACACAAACAACGGAGTTTGATATTGAGTCATGGCTGGCGGTTTCATATTTACAATTGTTGTAATAAAGTTAATAGCTCAGAATAGAAGAAATGCCTGCTAAATGTAGTGAAAAAATGGTTAAATCTACAGAGGCTCCTGGGTGGGATAAATTGCCTGCTAGGGGGTGGGTAGACTGTCCAGCCAGTGCCGGCACCAGCTTCTAAAGTTGATGCAAGCAGTAGGGTAGGGAGGGTGGTAGAAGTCAGAAGCTTATGTTATTTATTCGAGGAAATGCTATATCAGGAGCGCCAATTATTAAAGGAATAAGTCAGTTTCCCAAAACCCCCAATCATAATTGGTATTACTATGAAAAAAATTATGATGAATGCGTGGGCGGTGACAATAACATTGTATACATGGTCGTCTTCTATTAGGCTTCCGGGTTGTCCTAGTTCTGTTCGAATTAGGAGGCTTAGGGCTGTACCTACTGCCCCCGCCCATATATAAAATAATAAATATAATGTCCCAATGTCTTTGTGATTAGTTGAAAATGAGAAATCAGCGGTTTATGAACATAGGTAGGGGTAAAATGGCTGAGCAAGCATTAGACTGTAAATCTAAAGACAGAGGGAGTGAACCCTCTTTTTACCAGCCCTGAGGTGAACTATCACATTGAATTGCAAATTCAAAGAAGCAGCTTCAATTCTGCCGGGCTTCTCCCGCCTTTTTTCCCTAACGGCGGGAGAAGTAGATTGAAAACCAGTTGATTAGGTTATTTAGCTGTTAACTAAATTTTTGTGGGTTAAAATCCCATCAATCTAGGAAGGGCTTAGCTTAATTAAAGTAATTGATTTGCGTTCAGTTGATGCAGAATAAAGTTTTGCAGTCCTTATTTGTTGTGCAGAAATTAAGTAAAATTTACTTACTAAGGGCTTTGAAGGCTCTTGGTCTTATTAACCTAAATTTCTAAGTTATAAGTATTAGTGGAGTTAGGGGTAGGAGACGAGTGAAGACACTACAAGTGGGGATAGAAACGGTATTGGCTTTATATAATTTAGTTGCGGTTGATTTTTGTGTTATTTTGATGTTGGGAATATTGTCATTGAGATATAATATATTAAACGTATATAAAAATATAAATTTATTAATGTTAGTAAAGCTAGTATAAGGGGAATAATTAAGTTATCATTTTTAATAAGTTCTTGTATAATAGCTCATTTGGGGGAAAAAACCTGTTAGTGGGGGTAGACCTCCTAGGGATATTATTATTAGTGGAATAATGGGTATTGTTCATGCTAGTTTGTTTTCAGGTATGTGATAGGGATAGGGTTGTTACATTTGAGGTTAAATAAAAGATTATTAATGTAGAGATTGTTAGGAAAATATAAATAATTAAAGTTAATATAGTAATATTAGGGTCGTAATATAATACTGCTATTATTCATCCTATGTGAGTAATTGAAGAATAGGCTAGGATTTTGCGGAGTTGTGTTTGGTTGAGTCCTCCTCAGCTGCCAATTATAATTGATAGAACTGAGATTGTTAGAATCAAGTTTAGGTTTGTTGACGGAAAAAATTTGGAGGAGAATTGATATTGGGGCGAGTTTTTGTCACGTGAGAATAATTATAGCTGGGATTAGAGGGATTCCTTGGGTAATTTCTGGGAGTCAGAAATTGAAGTGGGGCTATCCTATTTTTATTGCTAGAGCAATAAATATTATTGTGGATAATATTTGGTTATAAGGAGGATGATTGTTCATTGTTGAGTTAGTAGATTGTTAAGAGGAAAATGGATGCTAATAGAAGTATTGATGCTGTTGCTTGAATTAAAAAATATTTGGTGGATGCCTCTGTGGAGCGGGGATTGGTACTTTTGGCAAGGATTGGTACGATGAAGCTGTGTCTGTTTAATTCTAAACCTATTCAGGCTAGGAATCAATGTGAGCTTAAAATTGTGATTACGGTCCCTGTTAGAATGGTGAAGGAGATAATAAGGTGGGCTAGAGGATTAATGTTAGCACAGGGAAGGATTAAACCAACATTTTCGGGGTATGGGCCGATAGCTTATTTAGCTGACCTTACTGTTTAGGATATGGTGTAATCGGTAGCACGGAGAGTTTTGAGTTCTCAGGTGTAGGTTTTCAATTCTGTAATCCTAGAAATAAGAGGGTTTGAACCTCTATAATTTACTCTATCAAAGTAACTCTTTTTATCAGACATATTTCTTATGTTTGGGGTGGGATGCCAGATGCTAGGATGGGTATTGAAACATATCATATACATAGTGCTAATGTAAGTGGTAAAAAGTTTTTTCATAGTAAGAATATTAATTGATCGTAGCGGAAGCGGGGGTAATACGTAA

>27e95b71-1fe3-4f08-a80f-8e2993362fc9

ATGTACTTCGTTCAGTTACGTATTGCTTGTGGTAAAAAATGAGCGAGGGATGATTTTGTTTTAAATCGGAGGCCTATTAGGCTACAGTTGCTGCTCATAGAGGAATAGCTATGCCTAGATTTATTGACAGTTGGGTGGTTGGTGTAAATGCATATGGTGTAAGTCCGAGAATGTTATTTAGGGCAATAAAAGAAATTAGGGCTAAATTATAAGGGATCAGGTTCGCCCTTTAGTGGTGTGGATTAATATTATTTGTTTAAGTGTTAGTTGAATTAGCCATTGTTGAATTGAAGAGTCGGTTGTTGAATAAGTTTTTTGGAGGATAAAATTAATGTAGTGGGAGTGCAATAATTAAAAATACTAAGGGTACTCTAGTATTGTTGGAATATTGACCCGAGCAAATAGATTTTGGTTCATTTTTAGTTCTCAAGTTGTTTTATGTTTTTGTGTTTCTACTAATTTTGGTAGTGGGTAATAATGGAAAGTAAAGTTCAGTATTTTCAATTGTATAATATAAAATAGGATAACAATTATAGATATAATTACTATTGGTCACGGTGAAATATTTAGTTGGAAGCATTCACTATAGAGTTTGTTCTCTCAATCTTTAACTTAAAAGGTTAATGCTAAGTTAGCTTTACAGTGATACAATATATAAGTATGAGCTCATACTTCGAAATCTTGAAATAAATGAATTCTAGAACAATAGGTATAAAGCTGTGATTGGACCCTGCCGAATTTCCGAGCATTGTCCATAAAATAGGCCTGGTCGTATAGAAGCTAGTATGGCTTGGTTTAAACGTCCAGGAATTGCATCTGTTTTACGCCTAGTGATGGTACGGCTCATGAGTGTAAGACGTCTTGTGATGAGATTAATATGCGAATATCCGCTTCTATAGAGTAAAGTTGTTCGGTTATCAACTTCGAGAGTCGAAATTCCCTGGCTCAAAGAAATATGTTGGCATAATGTAAGAGTCAAATACTAAGTCTTCATAGTCGGAATATTCATAGGTTCAGTATCATTGGTGACCAATTGCTTTAGGGTTAAATAAGGTTTATTAAATTCGTCTGTCATATATAGATACGCAGTGATGGGAGGGCAATTGTAATTAGAATCAGTGCAGGGAGAATAGTTCGAATTATTTTAAATCTCTTGACATTTATGGTGCTAGTATGAGTGAGTTTGTGGTAAGTATAGAGGGAAATAATATATAAGACTAAAGACTAATCAGGAAAATAATTATGAGTGCATGGTCGTGAAATAAATAAGTTCTTCTATGATAGGTGATGTGGCATTTTGTAAACTAGTTGAGCTGGTGTTGCTATTAAGATATATAGATATTTAGTCTATAATTTAACTTTGACAAAGTTATTGTAATTATTTTACTAATATCTTATTGAAAAGAATCATAGGGTATATGGGATTGGCTTGAAACCAATTTTTGGGAGTTCAAATCCTTCCTTTTTAAATCTAGGATTTAACGTAAGTTGCCTCTTCGAATGTGTGTGGTAAGGAGGAGGGCAGCCATATAATCATTCTAGATTGGTGGATAGTTGCTCAATGAAAAGCTAAGACTTTTCGTTTTGAAGAGAAAGCTACCAAATTATAAAAACCATCAGGACTACTGCTGTAAGTGAAATAAATGAACCGATAGATGAGACGATATTTCATGTAGTATATGCGTCTGGATAGTCTGAGTACTCGTCGAGGTATTCCGGATAAACCAAGGAAGTGTTGTGGGAAGAAAGTTAAATTTACGCCGACAAATATAATGGTGAAGTGGATTTTGGCATATGTTTGGTCAGAGTATAGCCTGAAAAGAGCGGGAATCAGTGAATAAATCCCCCTATAATGGCAAATACTGCTCCTATTGATAACACATAGTGAAAGTAGGCTACCCTTAGTATGTATCATGTAGCACAATATCTAATGATGAGTTAGCTAACACAATTCCCTGTTAGTCCACCCACGGTAAAAAAGAAAAATAAAGCCCAGGGCTCATAATATTGCAGGAGATCATTTGATATTACCGCCGTGCGGTGTGGCTAATCAGCTAAATACTTTTACTCCAGTGGGAATAGCAATGATTATGGTAGCTGATGTAAAATATGCACGAGTATCTACATCTATTCCTACTGTGAATATGTGATGGGCTCATACAATAAAGCCTAGAAACCAATAGATATTATGGCTCATACCATGCCCATATAACCAAAGGGTTCTTTTTATTAGAATAATATGTTACAATGTGTGAGGGGGTATTCCAAAGCCAGTAAGATTAGAATATATACTTCAGGGTGACCGAAGAATCAGAATAAGTGTTGGTATAGGATAGGGTCGCCGCCACCAGCAGGATCAAGAAAGTAGTATTTAAATTACGGTCAGTTAATAGTATGGTAATCACTGCAGCTAATGCCTGGAAGAGAAAGTAAAAGAAGGACTGCAGTAATTAGGACGGATCACACAAACAACGGAGTTTGATATTGAGTCATGGCTGGCGGTTTCATATTTGGTGACTGTAATAAAGTTAATAGCTCAAAGAATAGAAGAAATGCCTGCTAAATGTAGTGAAAAAATGGTTAAATCTACAGAGGCTCCTGGGTGGGATAAATTGCCTGCTAGGGTGGGTAGACTGTCCAACCGGAGGTGCCGGCACAACTCTAAAGTTTGATGATGCAAGCAGTAGGAGTAGGGAGGGTGGTAGAAGTCAGAAGCTTTACATGTTATTTATTCAGTACTATATCAGGGGGCATAATTATTAAAGGAATAAGTCAGTTCCCAAAACCCCAATCATAATTGGTATTACTATGAAAAAAATTATGATGAATGCGTGGGCGGTGACAATGGCATTTGTATACATGGTCGTCTTCTATTAGGCTTCCGGGTTGTCCCTGGATTCTGTTCCGAATTAGGAGGCTTAGGGCTGGCCACCACTGCCCCGCCCATGCGCCAAATAATAAATATAATGTCCCAATGTCTTTGTGATTAGTTGAAAATAATTAGCGGTTTATGAACATAGGTAGGGTAAATGGCTGAGCAGATGACTAGACTGTAAATCTAAAGACAGAGGTGAACCCTCTTTTTACCAGCCCTGAGGTGAACTATCGCATTGAATTGCAAATTCAAAAGAAGCAGCTTCAATTCTGCCGGGGCTTCTCCCGCCTTTTTTTTCCCTAACGGCGGGAGAAGTAGATTGAAGCGTTGATTAGGTTATTTAGCTGTTAACTAAATTTTTGTGGGTTAAAAATCCCATCAATCTAGGAAGGGCTTAGCTTAATTAAAGTAATTGATTTACGTTCAGTTGATGCAAGAATAAAGTTTTGCAGTCCTTATTTGTTGTGCAGAAATTAAGTAAAGTCCCACTTACTAAGGGCTTTGAAGGCTCTTGGTCTTATTAACCTAAATTTCTAAGTTATAAGTATTAGTGGAGTTAGGGGTGAAGACAGGTAGAAGACACTACAAGTGGGGATAGAAACGGTATTGGCTTTATATAATTTAGTTGCCAGTTGATTTTGTGTTATTTGATGTTGGGAATATTGTCATTGAGATATAATATATTAAACGTATATAAAAATATAAATTTATTAATGTTAGTAAAGCTATTATGGGGAATAATTAAGTTATCATTTTTAATAAGTTCTTGTATAATATGGCACAAATTTGGAGGGAAAAACCTGTTAGTGGGAATTTAGACCTCCTAGGGATATTATTAGTGGAATAATGGGTATTGTTCATGCTAGTTTGTTTCAGGTATGTGATAGGGATAGGGTTGTTACATTTGAGGTTAAATAAAAGATTATTAATGTAGAGATTGTTAGGAAAATATAAATAATTAAAGTTAATATAGTAATATTAGGGTCGTAATATAATACTGCTATTATTCATCCTATGTGGTAATTGAAGAATAGGCTAAGTTTTGCGGAGTTGTGTTTGGTTGAGTCCTCCTCAGCTGCCAATTATAATTGATAGAACTGAGATTGTTAGAATCAAGTTTAGGTTTGTTGACAGGAAAAATTTGGAGAATTGATATTGAGGCGAGTTTTTTGTCACGTGAGAGTAATTATAGCTGGGATTAGAGGGATTCCTTGGGTAATTTCTGGGAGTCAGAAGTGAAGTGGGGCTATCCTATTTTTATTGCTAGAGCAATAAATATTATTGTGGATAATATTTGGTTATAAGGAGGATTGGTTGTTCATTGTTGAGTTAGTAGATTGTTAAGGAAAATGGATACTAATAGAAGTATTGATGCTGTTGCTTGAATTAAAAATATTTGGTGGATGCCTCTGTGGGCGGGATTGGTACTTTTGGCAGGATTGGTACGATGGCTAGTATATTTAATTCTAGCCTATTCAGGCTAGGAATCAATGTGAGCTTAAAATTGTGATTACGGTCCCTGTTAGAATGGTGAGATAATAAGGTGGGCTAGAGGATTAATGTTAGCACGGGAAGGATTAAACCAACATTTTCGGGGTATGGGCCCGATAACTTATTTGTGACCTTACTGTTTAGGATATGGTGTAATCGGTAGCACGGAGAGTTTTGAGTTCTCGGGTGTAGGTTCAATTCCTGTAATCCTAGAAATAAGAGGGTTTGAACCTCTATAATTTACTCTATCAAAGTAACTCTTTTATCAGACATATTTCTTATGTTTGGGTGGGATGCCAGATGCTAGGATGGGTATTGACATATCATATACATAGTGCTAATGTAAGTGGTAAAAGTTTTCATAGTAAGAATATTAATTGATCGTAGCGGAAGCGAAGTGAAGCTGTTCGAATTCATAAAAATAAAGTGGTTAGTAGGAGGGTTTTGGCTATAAAATTAATAGTAAATGTTTCTGGTATAATTATATTGTAGGGTGTGGATGAGAAAATAGTGGTAGTTAGGGCATTTATTATAATAATATTTATATACTCTGCTATGAAGAATAAGGCGAATGAGCCTGCAGCGTATTCAATGTTGAAACCTGATACTAGTTCTGATTCGCCTTCAGCAATACGTAACA

>0bb30f4f-e52a-470c-add9-fe75f2920333

AGTATGCCTTCGTTTTCAGTTGTATATTGCTTAGAAAGAGCAGGAATCAGTGAATAAATCCCCCTATAATGGCAAATACTGCTCCTATTGATAACACATAGTGAAAGTGGGCTACTACATAGTATGTATCATGTAATACAATATCTAATGATGAGTTAGCTAACACAATTCCTGTTAGTCGCCACGGTAAAAGAAAAAATAAAATGCCCGGAGCTCATAATATTGCGGGGAGATCATTTGATATTACCGCCAATTATAAATCAATAATATAATCAGCTAATGCTTTTACTCCAGTGGGAATACTTAATGATTATGGTAGCTGATGTAAAAAATATGCACGAGTATCTACATCTATTCCTACTGTGAATATGTGATAGGCTCATACAATAAAGCCTAGAAACCAATAGATATTATGGCTCATACCATGCCCATATAACCAAGGGTTCTTTTTTATTAGAATAATATGTTACAATGTGTGAAATTATTCCAAAAACCCGGTAAGATTAGAATATATACTTCAGGGTGACCGAAGAATCAAGATAAGTGTTGGTATAGGATAGGGTCGCCGCCACCAGCGGGGATCAAAGTAGTATTTAAATTACGGTCAGTTAATAGTATGGTAATCCCTGCAGCTAGAACTGGAAGAAAGTAAAAGAAGGACTGCAGTAATTAGGACGGATCACACAAACAACGGAGTTTGATATTGAGTCATGGCTGGCTGGTTTCATATTTACAATTGTTGTAATAAAGTTAATAGCTCCAAGAATGAAGAAATGCCTGCTAAATGTAGTGAAAAAATGGTTAAATCTACAGAGGCTCCTGGGTAGGATAAATTGCCTGCTGGGGTGGGTAGACTGTCCAACCAGTGCCGGCACCGGCTTCTAAAGTTGATGATGCAAGCAGTAGGAGTAGGGAGATTGGTAGAAGTCAGAAGCTTATGTTATTTATTCCGAAATGCTATATCGGGAGCGCCAATTATTAAAGGTAAGTCAGTTCCCAAAACCCCCAATCATAATTGGTATTACTATGAAAAAATTATGATGAATGCGTGGACCGGTAATAACATTGTATACATGGTCGTCTTCTATTAGGCTTCGGGTTGTCCTAGTTCTGTTCGAATTAGGAGGCTTGGGAGCTGTACCTACTGCCCCCTTGTACCAAATAATAAATATAATGTCCCAATGTCTTTGTGATTAGTTGAAAATAGTCAGCGGTTTATGAACATAGGTAGGGGTAAAATGGCTGAGCAAGCATTAGACTGTAAATCTAAAGACAGAGGTAGACCCTCTTTTACCAGCCCTGAGTGAACTATCACATTGAATTGCAAATTCAAAGAAGCAGCTTCAATTCTGCAGCAATAA

>6ed0dd3f-6312-42b3-89e6-b6149e66e7d8

TTGTACTTCGTTCAGTTACGTATTGCTATATACTTCAAGAATTGACCGAAGAATCAGAATAAGTATTGGTATAGGATAGGGTCGCCGCCACCAGCGGGATCAAAGAAAGTAGTATTTAAATTACGGTCAGTTAATAGTATGGTAATCCCTGCAGCTAGAACTGGAAGAGAAAGTAAAAGAAGGACTGCAGTAATTAGGACGGATCATACAAACAACTTGAGTTTGATATTGAGTCATGGCTGGCGGTTTCATATTTACAATTGTTGTAATAAAGTTAATGGCTTCAAGAATAGAAGAAATGCCTGCTAAATGTAGTGAAAAAATGGTTAAATCTACAGAGGCTCCTAGGTGGGATAAATTGCCTGCTAGGGGTGGGTAGACTGTCCAACCAGTGCCGGCACCGGCTTCTGAAGTTGATGCAAGCGGTAGGAGTAGGGAGGGTGGTAGAAGTCAGAAGCTTATGTTATTTATTCGAGGAAATGCTATATCCAGGGCACCAATTATTAAAGGAATAAGTCAGTTCCCAAAACCCCCAATCATAATTGGTATTACTATGAAAAAAGATGTGATGAATGCGTGGGCGGTGACAATAACATTGTATACATGGTCGTCTTCTATTAGGCTTCCGGGTTGTCCTAGTTCTGTTCGAATTAGGAGGCTTGAGGCTGTACACACTACTTTACGCCCAGCGTACGCCAAATAATAAATATAATGTCCCAATGTCTTTGTGATTAGTTGAAAATAGTCAGCGGTTTATGAACATAGGTAGGGGTAAAATGGCTGAGCAAGCATTAGACTGTAAATCTAAAGACAGAGGAGTGAACCCTCTTTTTACCAGCCCTGAGGTGAACTATCACATTGAATTGCAAATTCAAAGAAGCAG

>2cd30ede-8a10-4897-bb9d-269f5e44c11d

GATGACCAAGCTGATTCCAGTTACGTATTGCTGGCTTCGAAGCCGGAAATGGTGGCTAGATGTGATGATATAATTGTTGGCGAAAGTAACAGGTGGTGAGGAAAGTGGTTCCGATAATTACGTGAAAGGCCATGAAAGCCTGTAGCCATAAAAAATGTGGATCCATATACTCCGTCGGAGATAGTAAATGGGGCTTCAGAATATTCTGATAATTGTAGGCAGGTGAAGTAGATCCCTAATATAATGAGTTGAAGGTAGTAGCAGGTTGATTCTTTTCAGTTGGCTTCTAGGCTGTGATGTGCTCATGTAATTGCTAACTCGTGCTAGTAATCACAGTTGTATTTAAGAAAGGGACTTCTATTGAGTTGAGAGATAATGCCTGTAGGTGGTCATAGTCCCCCTGTTTGTGGAATTGGGAGCTAGACTAGAATGATAAAATGCTCAGAAGAAACCTGCAAAGAAGAAAATTTCAGAAATAATAAATAGAGAGATTATTCCGTATCGTAGGCCTTTTTGGACAGGTGCAGTATGAGTGGCCTTGATATGTACTTTCTCCGTACTACATCACGTCATCATTGAAATATTGTTATAGAACTAGCTAGTAGACCTGCGGTGAAAGCAGTGTGTATTAAAAGTGGAATCATATAACTAAGCCGGATGTGAGAAGAAAAGCTGATAATGCTCCTGTTAGTAGTCCAAGGGCTTGAGTTGACTATGTGATAAGCATGGGTTTGGTGAGTCATTATGAGTTATCATGTAAGTACAGGCTTACTAAGAGGGTAAATACATGAGCTTGAATTAAGGCTACACCCAACTCTAGGGTAATTAATGGAAAGCAATAATAATGATGATGATTGTGGAGGTGAGAGGTAAATTGATAGAAGAGTTAATGTTGTATCCCAAGTAAATGCATTAATAGGTGACCTGCTGTAATATTGGCTGTTAATCGTACGGCTAAGGCTACAGGTTGGAATGAATAGACTAATTGTTTCAATAATAATTAATATAGGGATTAGTGGAATTGGTGTTCCTTGGTAAAAATGAGCGAGGATGATTTTGTTTTAAATCGGAGGCCTATTAGTACAGTTGCTCATGGGAATAGCTATGCCTAGATTTATTGACAGTTGGGTGTTGGTGTAAATGCATATGGTGTAAGTCAAGAGATGTTATTTAGGGCAATAAAGTGTAGGGCTAGGAGTATAAGGGATCAGGTTCGCCCTTTGATGGTGTGGGTTAATATTATTTGTTTAAGTGTTAGTTCGAATTAGCCATTGTTGAATTGAAGAGTCGGTTGTTGAATTAAGTTTTTTGGAGGATAAAATTAGTAATGTAGTGGGGAGTGCAATAATTAAAAATGCTAAAAATTTATCTAGGTATTGTTGGAATATTAGATGAGGCAAATAGATTTTGGTTCATTTTAGTTCTCAAGTTGTTTTATGTTTTTGTGTTTCTACTAATTTTGGTAGTGGGTAATAATGGAAAGTAAAGTTCAGTATTTTCAATTGTATAATATAAAAATAAGGTAACAATTATAGATATAATTACTATTGGTCACGGTGAAATATTTGGTTAAGGCATTCACTATAGAAGTTTGTTCTCTCAATCTTTAACTTAAAAGGTTAATGCTAAGTTAAAGCTTTACAGTGATACAATGTCTCAAGTATGAAGCTCATACTTCGAAATCTTGGAAATAAATGAATTCTGGAGCAATAGGTATAAAGCTGTGATTGGACCCGCAAATTTCCGAGCATTGTCCATAAAATAGGCCTGGTCGTATAGAGGCTAGTATGGCTTGGTTTAAACGTCCAGGAATTCATCTGTTTTTACGCCTAGTGATGGTGACTCATAGTGTAAGACTCTTGTGATGTGGAGTCAATAACCACGAATATCCGCTTCTATAGGTAAGTTGTTCGGTTATCAACTTCGAAAGTCGAAATTCCCTGACTCAAGGAAATATGTTGGCATAATGTAGAGTCAAGTCTAAGTCTTCATAGTCGGAATATTCATAGGTTCAGTATCATTGGTGACCAATTGCTTTAAGGGTTAAATAAGGTTTATTAAATTCGTCTGTCATATATAGAATACGCAGTGATGGGAGGGCAATTGTAATTAGAATCAATTGCAGGAGAATAGTTCAGATTATTTCGATCTCTTGAGCATTTATGGTGCTAGTATGAGTGAGTTTTGTGGTAAATGTAAAGGAAATAATATGTAAGACTAAGGAACTAATCAGGAAAATAATTATGAGTGCATGGTCGTGGAAAGCAATAAGTTCTTCTATGATAGGTGATGTGGCATTTGTAAACCTGGACATTGTTGCTATTAAGATATATAGATATTTAGTCTATAATTTAACTTTGACAAAGTTATGTAATTATTTTACTAATATCTTATTGAAAAAGTCATAGGGTATATGGGATTGGCAGAAACCAATTTTTTTTTTTTTAGGTTCAAATCCTTCCTTTTTCGTCTAGGATTTAACGTAAGCCTCTTCGAATGTGGTAAGGAGGAGGGCAGCCATTATAATCATTCTAGATTGGTGGATACGTTGCTCAATGGCTAAGACTTTTCGTTTTGAAAGCTTCTCAAATTATAAAAACCATCAGGACTACTGCTGTAAGTGAAATAAATGAACCGATAAGATGAGACGATATTTCATGTAGTATATGCGTCTGGATAGTCTGAGTATCGTCGAGGTATTCCGGATAAGCCCGAGAAGTGTTGTAGGAAGAAAGTTAAATTTACGCCGACAAATATAATGGTGAAGTGGATTTTGGCATATGTTTGGTCAAGTATGCCTGAAAAGAGCGGGAATCAGTGAATAAATCCCCTATAATGGCAAATACTGCTCCTATTGATAACACATAGTGAAAGTGGGCTACTACATAGTATGTATCATGTAATACAATATCTAATGATGAGTTAGCTAACACAATTCTGTTAGTCCACCCACGGTAAAAGAAAAATAAAGCCCAGGGCTCATAATATTGCAGGAGATCATTTGATATTACCGCCGTGCAGTGGCTAATCGGCTAAATACTTTTACTCCAGTGGGAATAGCAATGATTATGGTAGCTGATGTAAAATATGCACGAGTATCTACATCTATTCCTATGTGAATATGTGATGGGCTCATACAACAAGCCTAGGAAACCAATAGATATTATGACTCATACCATGCCCATATAACCAAAGGGTTCTTTTTTATTAGAATAATATGTTACAATGTGTGTAAAATTATTCCAAAACCCGGTAGATTAGAATATACTTCAGATAGGCCGAAGAATCAAGAATAAGTGTTGGCATAGGATAGGGTCACCGCCACCAGCAGGATCAAAGAAAGTAGTATTTAAATTACGGTCCAGTTAATAGTATGGTAATCCCTGCAGCTAGAACTGGAAGAGAAAGTAAAAGAAGGACTGCAGTAATTAGGACGGATCACACAAACAACGAGTTTGATATTGAGTCATGGCTGGCGGTTTCATATTTACAATTGTTGTAATAAAGTTAATGGCTCAGAATAGAAGAAATGCCTGCTAAATGTAGTGAAAAAATGGTTAAATCTACGAGGCTCCACCAGGTGGGATAAATTGCCTGCTAGGGGTGGGTGAACTGTCCAACCAGTGCCGGCACCGGCTTCTAAAGTTGATGATGCAAGCAGTAGGAGTAGGGAGGGTGGTAGAAGTCAGAAACATATTTATTTATTCGAGGAAATGCTATATCGGGAGCGCCAATTATTAAAGAATAAGTCAGTTCCCAAAACTGATATGATTGATATTACTATGAAAAAATTATGATGAATGCGTGGGCGGTGACAATAACATTGTATACATGGTCGTCTTCTATTAAGGCTTCCGGGATTGTCCTAGTTCTGTTCGAATTAGGAGGCTTAGGGCTGTGCACCTACTGCCCCCGCCCATGCGCCAAATAATAAATATAATGTCCCAATGTCTTTGTGATTAGTTGAAAATAGTCAGCGGTTTATATGAACATAGGTAGGGGTAAAATGGCTGAGCAAGCATTAGACTGTGTAAATCTAAAGACAGAGGAGTGAACCCTCTTTTTTACCAGCCCTGGGTGAACTATCACATTGAATTGCAAATTCAAAGAAGCAGCTTCAATTCTGCCGGGGCTTCTCCCGCCTTTTTTCCTAACGGCGGGAAGTAGATTGAAGCCAGTTGATTAGGTTATTTAGCTATTAGCTGTTTTTGTAGGTTAAAAATCCCATCAATCTAGGAAGAGGCTTAGCTTAATTAAAGTAATTGATTTGCGTTCAGTTGATGCAGAATAAAAATTTTTGCAGTCCTTATTTGACATTGCAGAAATTAAGTAAATTTGCTTACTAAGGGCTTTGAAGGCTCTTGGTCTTATTAACCTAAATTTCTAAGTTATAAGTATTAGTGGAGTTAGGGGGGTAGGAGACAGGTAGAACACTACAAGTGGGGATAGAAACGGTATTGGCTTTATATAAGTTTAGTTGCCAGTTGATTTTGTGTTATTTGATGATGAAACTTGTTTTGTATTATGTTGGGAATATTGTCATTGAGATATAATATATTAAACGTATATAAAAATATAAATTTATTAATGTTAGTAAAGCTATTATAAGGGGAATAATTAAGTTATCATTTTTAATAAGTTCTTGTATAATAGCTCATTTGGAGAGAAAAACCTGTTAGTGAGGTAGACTCTAGGGATATTATTATTAGTGGAATAATGGGTATTGTTCATGCTAGTTTGTTTCAGGTATGTGATAGGGATAGGGTTGTTACATTTGAAGGTTAAATAAAAGATTATTAATGTAGAGATTGTTGGGAAAATATAAATAGTTAAATTAATATAGTAATATTAGGGTCGTAATATATCTGCTATTATTCATCCTATGTGGTAATTGAAGAATAGGCTAGAGGATTTTGCAGGTTGTGTTTGGTTGAGTCCTCCTCAGCTGCCAAATTATAATTGATAGAACTGAGATTGTTAGAATCAAGTTTAGGTTTGTTGACGGAAAAATTTGGAGGAGAATTGAGTATTAGAACAAGTTTTTGTCACGTGAGAATAATTATAGCTGGGATTGATTCCTTGGGTAATTTCTGGGAGTCAGAAGTGAAGTGGGGCTATCCCTATTTTTATTGCTAGAGCAATAAATATTATTGTGGATAATATTTGGTTATAAGGAGGATTGATTGTTCATTGTTGAGTTAGTAGATTGTTAAGGAAAATGGATACTAATAGAAGTATTGATGCTGTTGCTTGAATTAAAAAATATTTGGTGGATGCCTCTGTGGGCAAGGGATTGGTACTTTTGGCAAGGATTGGTACGATGGCTAGTATATTTAATTCTAAGCCTATTCGAGCTAGGAATCAATGTGAGCTTAAAATTGTGATTACGGTCCCTGTTAGAATGGTGAAGGAGATAATAAGGTGGGCTAGAGGATTAATGTTAGCACGGGAAGGATGGCTTAACATTTTCGGGGTATGGGCCCGATAGCTTGTTACAGCTGACCTTACTGTTTGGGATATGGTGTAATCGGTAGCACGAGAGGATTTTGAGTTCTCAGGTGTAGAGTTCAAAATTTCTACCGTCTAGAAATAAGAAGGTTTGAACCTCTATAATTTACTCTATCAAGTAACTCTTTTATCAGACATATTTCTTATGTTTAAGGGTGGGATGCCAGATGCTAGGATGGGTATTGAAGCATATCATATGTGTGGTGCTAATGTAAGTGGTAAAGTTTTTTCATAGTAAGAATATTAATTGATCATGGCAGAGCGGGGTAGAGCTGTTCGAATTCATGTAAAAATAAAGTGGTTAGTAGGAGGGTTTGGCTATAAAATTAATGGTAAGTGTTTCTGGTATAATTATGTTGTAGGGTGTGGATAAGAAAATAGTGGTAGTTAGGGCATTTATTATAATAATATTTATATACTCTGCTATGAAAAAATAAGGCGAATGAGCCTGCAGCGTATTCAATGTTGAAACCTGATACTGATTCTGATTCGCCTTCTGTCAGGTCGAATGGAGCTCGATTGGTTCTGCTAATGTGGAAATAAATGTATTATGGCTAGGGCCATGATGGAAGTAGAAGTCAGTAGTGTTCTTGGTGGTAATAATAAGCGATTGTAGATTGAATGAGCCACGCTTATTAGTAGTGTTGATAAAGTAAGATAATGTAAGGGTGACCTCATATGAGATATGTTTGGGCTACAGCTCGTAGTGCGCCAATTAGTCGCATTGGTTTGAGTTGGATGCTCAGCCAGATCATAAAATTGAGTAAACAGCTAGGCTTGATATTTGCGAGGATAAATAGGAAGCCAAGATTAGAAGTTGATAAGAGGATATGGTATGGGAGTGGCGTTCATAGTAGAAGAGCAATGGAAAAGAGCTGGGGTGAGGCAGTTAAATATAAAGTTATGGTGGATGTAGTGAGTAATAAGGGTTCTTTTGTGAAGAGTTTTATGGCGTCATGATGGTTGAAAGTATTCAGCCAGGAGCCTATATTATTAGGAGCATACAGGTGTATATAACTGGGATTTTCGTTCTGTGAGTGTTAAAGGCTATGGCAATTAGACATTAGGACTAGTATTAGTAGTTAATTATATACATGTTGTTAAGAAGGAATTGAACCTCTGATTATAAAGTTTAAGTTTATGCAATTACCGGGCTCTACTTACCTTAACGAGCCCTGTTCTTGGGCAAGATTATAATATGTGAGTTGAATGGAGTCATCTGATTGTTGGGGGCGCTTTATGAAGTGGGCCCCATTTCTCTTGTCCTTTCGTACTGGGGAGAAATATTTAAATAGATAAAAACCGACCTGGATGCTCCAGATACAGACTCAGATCACGTAAGACTTTAATCGTTAACAAACGAACCCTTAATAGCTTCTGCGTGGGATAATAATGATCCAACATCGAGGTCGTAAACCCTATTATCGATATGGACTCTAAATAGGATTGCGCTGTTATCCCTAGGGTAACTTGGTCCGTTGATCAAATTATTATTGGGTCAATAGGTGCTTGCTTACTTGAACTGGTAAGGTCTTGGTATATGTTTTTCGGAGGTTCTATTTTACTCCGAGGTCGCCCCAACCGAAATTTATAATGCACATTGACGGTAAATTAATGCCTGTTGGTTTTTTACAAATCGTTTGTGACATATCATTAAATTAAAGCTCCACAGGTCTTCTCGTCTTATTTATGTATATCCACCTCTTCACAGATAGGTCAATTTCACTGATTAAAGAAAGTAGAGACAGTTAAACCCTCGTGTGGCCATTCATACAAGTCCTATTTAGAGAACAAATGATTATGCTACCTTTGCACGGTCAGAATGCCGGCCGTTGAACATATGTCACTG

>ece43544-0744-41f0-8da5-48f7e630f052

AAGTGTACTTCGTTCAGTTGCGTATTGCTAATATATACTTCAGGGTGACCGAAGAATCGAATAGAATGTTGGTATAGGATAAGGTCACGCCGCCGCAGCAGGATCAAAGAAAGTAGTATTTAAATTACGGTCAGTTAATGAGTATGGTAATCCCTGCAGCTAGAACTGGAAGAAAGTAAAAGAAGGACTGCAGTGGTAGGACGGATCACACAAACAACGGAGTTTGATATTGAGTCATGGCTGGCGGTTTCATATTTACAATTGTTGTAATAAAGTTAATAGCTCAAGAATAGAAGAAATGCCTGCTAAATGTGATGAAAAAATGGTTAAATCTACAGAGGCTCCTGGGTGGGATAAATTGCCTGCTAGGGGTGGGTAGACTGTCCAACCAGTGCCGGCACCGGCTTCTAAAGTTGATGATGCAAGCAGTAGGAGTAGGGAGGGTGGTAGAAGTCAAGCTTATGTTATTTTATTCAATGCTATATCGGGAGCGCCAATTATTAAAGGGAATAAGTCAGTTCCCAAAACCCCAATCATAATTGGTATTACTATGAAAAAAATTATGATGAATGCGTAGGCGGTGACAATAACATTGTATACATGGTCGTCTTCTATTAGGCTTCCGGGTTGTCCCTAGTTCTGTTCGAATTAGGAGGCTTAGGGCTGTACCTACTGCCCCCGCCCATGCGCCAAAATAATAAATATAATGTCCAATGTCTTTGTGATTAGTTGAAAATAGTCAGCGGTTTATGAACATAGGTAGGGTAAAATGGCTGAGCAAGCATTAGACTGTAAATCTAAAGACAGAGGTGAACCCTCTTTTTACCAACCTGAGGTGGTGCTATCACATTGAATTGCAAATTCAAAGAAGCAGCTTCAATTCTGCCGAGGCTTCTACTCCGCCTTTTTTTTTTCCCCTAACGGCGGGAGAAGTAGATTAAGCCAGTTGATTAGGTTATTTAGCTGTTAACTAAATTTTTGTGGTTAAATCCCATCAATCTAGGGAAGGGCTTAGCTTAATTAAAGTAATTGATTTCGTTCAGTTGATGCAGAATAAAGTTTGCAGTCCTTATTTGTTGTGCAGAAATTAAGTAAAATTTGCTAGCAATACGTAACT

>3634f245-07d2-4c6d-8d41-cb8ccbe2349c

GATAGCTGCTTTGAATTCCAGTTACGTATTACTAATAATTAGGGTGGGATTAGGGTAGTTTCAAATAGAATATAAAATAAATTAGTTCGGTAGCTGAAAAAGTTATAATAGTGAAATTTGTAATAGAATTAATATTGAGATATAGTTTTTTCGTGGATGAAGTTATTATATAAATGCTGTTGTGTTGCTAGAATTATTATTGGCGAAGTCAGGCTGTTAATATAAGAAGTGGTGATGTTGGTGAATCTGAGGAAAAAATCCATTTGACCAAGTTGCATGAGTTATTTAGTGTAGATAATATGGGTGCTTATATACTGATTAATGTTACAGATTATTATATTAGGTCATAATATATAATTTTTTTGAAAATCATATGGTGAGCATCTTATGATGATTGGAAAAATAAATTTTTAACAGTGTTGAACGGTTGGTTTGTACATAATCTAGGCCATATAGGTTGGAGACTAAAATTAACAAGGCTGAGCCTACGCTTGTGCGCGCTTAAATACTAGGAGAATGAGGGTATCATATGCATTATTATGGAAGTGCATATTGAAAGTTGTGAGTGTAATTATGATATAATAATGATAATATTATACCTTCAAACATAGCAGTGATGATATTAGGTGAGATCGATAAATTAATAACCCTATTAATGATATAAGAAATATGCTAGTATTACATTAATATAGATAAAAGGCATATTTGTAATCTGTGGAAAGCTTTCCTGTAATCTAATAAGTCAAATCATTTGTTTAATTAAACTATATACCAATTCAACCCAATCTAATCCTTTTTGGGACCTCTGTCAGCTAACCTAAAGCCAGAATAATAAATGGAGGTAAAAATTATATTTATTGTTAGAGTTAAATTATTTGTTTGGGTTGCCCATGGTAGGGGTAGAAGTAGGAGCAATTTCCAGATCAAATAGGGAGAAATGCTTGATGGCGATTAGGAAAAATTTTGGAGAATGGTAAGTGGGCGGAGGTTGTAGGATCAAATCCACCTCAATAAGGAGTTGTGCTTTTCTGCTCTAAATATTTAATTGTGGAAGTCAAAATGTAATTGTGATAGATAAGGCCAATGTGTAGTCATGCGAGGGCTAAATTAATTATAATACTCTCTCTCGAATCATTCGAGGCCATTGATTGGAAGTCAATAATACTTTTTATACTAAAGAGTAAAACCTCATCAATAGATGGAAATATAGAGGAAAAGTCGCTACATCTACGAAATGTCAATATCATGCAGCGGCTTCGAAGCCGAAATGGTGGCTAGATGTGAAAAGTGATATAATTGTTGGCGAAAGTAACGAATTGGTGAGGAAAGTGGTTCCATTAAGTACGTGGAAAACCATATGAAAGCCTGTAGCCATAAAAAATGTTGAATCATGTCTCCGTCGGAAGCGTAGTAGGGCTTCAGAATATTCTGATAATTGTAGGCAGGTAGTAGATCCCTAATATAATGGTTGGAGTAGTGCTTAGGTTGATTCTTTTCGGTTAGCTTCTATGAGGCTGTGATGTGCTCATGTAATTGTAACTCCTGATGCTAGTAGTACGGTTGTATTTAAGAGAGGACTTCTATTGATTTGAGGAAGTAATGCCTGTAGGTGGTCATAGTCCCCCTGTTTGTAGGTTGGGAACTGAACTAGAATGATAAAATGCTCAGAAGAAACCTGCAAAAGAAGAAAATTTCTGAAATAATAATAGAATTAATCCATCGTAGGCGGCCTTTTTGGACAGGTGCAGTATGGTGGCCTTGATATGTACTTTCTCGTACTACATCACGTCATCATTGAAATATTGTTATAGAACTAGCTAGTAGACCTGCGAACGAAAGTAATTATTGGTAAAAGTGGAATCATATGTGACCGGATGTAGAAAAGACTGATAATGCTCCTGTTGGTGGTCAAAGACTTGATTAAATATGTGATAAGCATGGGTTTAGGTGAGTCATTATGAATTATCATGTAAGTACAGGCTTACTAAGAGGTAAATATGAAGCTTGAATTAAAATGCCACCCAACTCACGTTAATTAATAAAATAATAATAATGATAGTGATTGTGGAAGTGGAGAGGTAAATTGATAGAAGAGTTAATGTTGTATCCCCAAGTAAATGCATTAATAGGTGACCTGCTGTAATATTGGCTGTTAATCGTACGGCTAAGGCTACAGGTTGAATGAATAGACTAATTGTTTCACAATAATAATTAATATAGGATTAATTAAATTGGTGTTCCTTGTGGTAAAAAATGAGCGAGGATGATTTTGTTGTTTTAAATCGGAGGCCTATTAGTACAGTTGCTGCTCATGAAACAAGCTATGCTGAATTTATTGACAGTTGATTGGTTGGTGTAAATGTATGGTGTAAGTCCGAGAATGTTATTTAGGGCAATATTAAAAGAAATTAGGGCTAGAATTACTAAGGGATCGAGTTCGCCCTTTAGTGGTTAATGAGTTAATATTATTTGTTTAAGTGTTGATTGAATTAGCCATTGTTGAATTAGGAAGAAGAGTCGGTTGTTGAATAAGTTTTTGGAGGATAAAATTAATGTAGTGGGGAGTGCAATAATTAAAAATACTAAAGGGTACTCCTAGTATTGTTGGAATATTGAATGAGGCAAATAGATTTTGGTTCATTTTTAGTTCTCAAGTTGTTTTGTATGTTTTTGTGTTTCTATAATTTGGTAAGTGGGTAATAATGGAAAGTAAAGTTCAGTATTTTCAATTGTATAATATAAAATAAGGTAACAATTATAGATATAATTACTAATATTGGTCACGGTGAAATATTTGGTTGAGGCATTCACTATAGAGAAGTTTTATTCTCTCAATCTTTAACTTAAAGTTAATGCTAAGTTAGCTTTACAGTGATACAATATATAAGTATGAAGCTCATACTTCGAAATCTTGGAAATAAATGAATTCTAGAACAATAGGTATAAAGCTGGTTGGACCCACCAAATTTCCGAGCATTGTCCATAAATAGGCCTGGTCGTATAGAGGCTAGTATGGCTTGGTTTAAACGTCCAGGAATTGCATCTGTTTTTACGCTAGTGATGGTACGGCTCATGAGTGTAAGACGTCTTGTGATGAGATTAATATGCGAATATCCGCTTCTATAGGTAAAGTTGTTCGGTTATCAACTTCGAAGTCGAAATTCCTAGCTCCAAGGAAATATGTTGGCATAATGCAAAGTCAAATACTAAGTCTTCATAGTCGGAATATTCATAGGTTCAGTATCATTGGTGACCAATTGCTTTAAAGGGTTAAATAAGGTTTGTCAAATTACGTCTGTCATATATAGAATACGCAGTGATGGGAGGCAATTGTAATTAGAATCGGTGCAGGGAGAATAGTTCAGATTATTTCGATCTCTTGGTATTTTTATGGTGCTAGTGAGTGAGTTTTGTGGTAGTCAAAATAATATATAGGGCTAGGGAACTAATCAGGAAAATAATTATGAGTGCATGGTCGTGGAAAGCAATAAGTTCTTCTATGATCATTAGGTGATGTGGCATTTTGTAAACCTAGTTGAGCTGGTGTTGCTATTAAGATATAGATATTTAGTCCTGTAATTGCTTTGACAAAGTTATGTAAATTATTTTACTAATATCTTATTGAAAGAATCACTAGGTATATGGATTTGCTTGAAACCAATTTTGGGGGTTCAAATCCTTCCTTTTCATCTGATTTAACGTAAGTTGCCTCTTCGAATGTGTGGTAGGAGGAGGGCAGCCATATAATCATTCTAGATTGGTGGATAGTTGCTCAATGGCTAAGACTTTTCGTTTTGAAGAGAAAGCTTCTCAAATTATAAAAACCATCAGGACTACTGCTGTAAGTGAAATAAATGAACCGATAGATGAGACGATATTTCATGTAGTATATGCGTCTGGATAGTCTGAGTGTCGTCGAGAATATTCCGGATAAGCTTCAAGGAAGTGTTGTGCTGGGAAGAAAGTTAAATTTACGCCGACAAATATAATGGTGATGGATTTTGGCATATGTTTGGTCAAGTATAGCCTGAAAAGAGCGGGAATCAGTGAATAAATCCCCCTATATTGGCAAATACTGCTCCTATTGATAACATAGTGAAAGTGGGCTGCTACATAGTATGTATCATGTAATACAATATCTAATGATGAGTTAGCTAACACAGTTCTTTATTAGTCCACCACGGTAAAAGAAAAATAAAGCCCAGGGCTCGCCATATTGCGGAGATCATTTGATATTACCGCCGTGCAGTGTGGCTAATCAGCTAAATACTTTTACTCAGTGGGAATAGCAATGATTATGGTACTTCAAATGTAAAATATGCACGAGAGTATCTACATCTATTCCTACTGTGAATATGTGTTAGGCTCATACAATAAAGCCTAGGAAACCAGCCAGATATTATGGCTCATACCATGCCCTTATAACAAAGGGGTTCTTTTGATAGAATAATATGTTACAATGTGTGAGTATTCCAAAACCCGGTAGATTAGAATATATACTTCAGGGTGACCGAAGAATCAGAATAAGTATTGGTATAGGATAGGGTCGCCGCCACAGCAGGATCAAGAAAGTAGTATTTGGTACGGTCAGTTAATAGTATGGTAATCCCTGCAGCTAGAACTGGAAGAAAGTAAAAGAAGGACTGCAGTAATTAGGACGGATCACACAAACAACGGAGTTTGATATTTAGTCATTAGCTGGCGGTTTCATATTTACAAGTGTTATGTCAGGGTCTAATAGCTGGAATGAAGAAGGCTGCTAAATGTAGTGAAAAAATGGTTAAATCTACAGAGGCTGGGTGGGATAAATTGCCTGCTAGGGGGGTGGGTAGACTGTCCAGCCAGTGCCGGCACCGGCTTCTAAAGTTGATGATGCAAAAAGCAGTAGGAGTGGGAGGGTGGTAAGTCGACTTATGTTATTTATTCGAGAAATGCCCACTATCGGGAGCGCCAATTATTAAAGGAATAAGTCAGTTCCCAAAACCCCCAATCATAATTGGTATTACTATGAAAAAATTACCTTGATGAATGTAAGGCAGTGACAATAACATTGTATACATGGTCGTCTTCTATTAGGCTTCGGGTTGTCCTAGTTCTGTTCGAATTAGGAGGCTTGGGGCTGTACTATCTGCCCCGCCCATGCGCCAAATAATAAATATAATGTCCCAATGTCTTTGTGATTAGTTGAAAATAGTCAGCGGTTTATGAACATAGGTAGGGTAAAATGGCTGAGCAAGAAGCATTAGACTGTAAATCTAAGAGACCAGAGAGTGAACCCTCTTTTACCAGCCCTGAGGTGAACTATCACATTGGTGCTAAATTGAAAAGCCCCTTCAATTCTGCCGGGGCTTCTCCCACCTTTTTCCCTAACGGCGGGAGAAGTAGATTGAAGCCAGTTGATTGGTTATTTAGCTGTTAACTAAATTTTTAATGATTCAAATCCCATCAATCTAGAAAGACAGCTTAATTAAAGTAATTGATTTGCGTTCAGTTGATCTAGAAATAAAGTTTTGATCCTTATTTGTTGTGCAGAATTAAATAAAATTTGCTTACTAAGGGCTTTGAAGAGCTCTTGGTCTTATTAACCTAAATTTCTAAGTTATAAGTATTAGTGGAGTTAGGGGTAGGAGACAGGTAAAGACACTACAAGTGGGGATAGAAACGGTATTGGCTTTATATAATTTAGTTATGATTGATTTTTGTGTTATTTGATGTTAGGAATATTGTCATTGAGATATAATATATTAAACGTATATAAAAATATAAATTTGTATTAATGTTAGTAAACTATTATAAAGGGGAATAATTAAGTTATCATTTTTAATAGTTCTAACGTATAATAGCTCATTTGGGGAAAAACCTGTTAGTGGGGGTAGACCTCTGGGATATTATTATTAGTGGAATAATGGGTATTGTTCATGCCCGGACCCGGAATGTATGCATGATGAGATGAAGGTTGTTACATTTGAGGTTAAATAAAAGATTATTAATGTAAAGATTGTTAGGAAAATATAAATAATTAAAGTTAATATAGTAATATTAGGGTCGTAATATAATACTATTATTCATCCTATGTGAGTAATTGAAGAATGAGCTAGGATTTTGCGAGTTGTGTTTGGTTGAGTCCTCCTCAGCTGCCAATTATAATTGATAGAACTGATTGTTAGAATCAAGTTTAGGTTTGTTGACGGAAAAATTTGGAGGAGAATTGATATGGGGCGAGTTTTTGTCACGTGAGAATAATTATAGCTGGGATTAGAGGTTCCTTGGGTAATTTCTGGGAGTCAAGTGAAGTGGGGCTATCCCTATTTTTATTGCTAGAGCAATAAATGGTATTGTGGATAATGTTTGGTTATAGGAGGATTGATTGTTCATTGGAGTTGATAGATTGTTAAGGAGAAATGGATACTAATGAAGTATTGATGCTGTTGCTTGAATTAAAAAATATTTGGTGGATGCCTCTGTGGAGGCGAGTTGGTATGCTTTTGGCAAGGATTGGTACGATGGCTAGTATATTTAATTCTAAGCCTATTCGATAGGAATCAATGTGAGCTTAAAATTGTGATTACGGTCAGCAATACGTAA

>70b990a1-74fd-4400-892c-cab63b965b8f

GTGTATTACTTCCATTCAATTCACATATTGCTATAAAGTTAATAGCTCCAGAATAAGAAAGAAATGCCTGCTAAATGTAAGTGAAAAAAATGGTTAATCTGCAGAGGCTCTAAGGTGGGATAAATTGCCTGCTAGGGGTGGGTAGACTGTCCAGCAGTGCCGGCACCGGCTTCTAAGTTGATGATGCCAGCAGTAGGAGTAGGGAGGGTGGTAGAGAATGAAGCATATTGTTTATTCGAGGAAATGCTATATCGGGGCGCCAATTGTAAAGGAGCCGAGTCAGTTCCCAACCCAATCATAATTGGTATTACTATCGAAAAAATTGTGATGAATGAAGTATGACGAGGTGATAATAACATTGTATACATGGTCGTCTTCTATTAGGCTTCCGGGTTGTCCTAGATATGTTCGTTAAGAGGCTTGGGGCTGCCTACCACTTTACTTGTATCAAATAATAAATACAATATTCCCCCAATCTTGTGATTGGTTGAAAATAGTCAGCGGTTTATGAACATAGGTAGGGGTAAAATGGCTAAAACAACATTGAGCTGTACAATCTAAAGACAGAGGAGTGAACCCTCTTTTACCAGCCCTGAGGTGAACTATCATTTGAATTGCAAATTCAAAGAAGCAGCTCGAGATCTGGGCTTCCTCCCTTTGCTTTTTTCCCTAACGTAGGGAGAATGGCTTAATACGTAGTAA

>ddf868dd-ab55-4940-ad88-5da425d3ba9e

GTATACTTCGTTCAGTTGCATCTTTATGATGGATAAATTGCCTGCTAGGGGTGGGTAGACTGTCCAGCCAGTGCCGGCACCGGCTTCTAAAGTTGATGGTACAAGCAGTAAAGGTAAGGAGGGTGGTAAAGTCAGAAGCATGTTATTTATCAGAAATGCTATATCAGAGGCGCCAATTATTATTAAAGAATAAATTAGTTCAAAACCTAAATCATAATTGGTATTATGAAAAATTATGATGAATGCTGGGCGGTGACAATAACATTGTATACATGATCGTCTTCTATTGGGCTTCAGGTTGTCCTAAGCGTTCTGTTCAAAGATTAAGGAGAGCAGATATACTTCCCTTAGCGCCAAATAATAAATATAGTAGCCCAATCTTGTGATTGGTTAGAAAATAGTCAGCGGGTTTATGAACATAAGTGGGAATGCAAAATGAGCAAGCATTAGACTGTAAATCTAAAGACAGAAGTGAACCCCTCTTTTTACCAACCCTAGAGGTGAACTATCACGTTAGTTGCAAATTCAAAGAAGCAGCTTCAATTCTGCCGGAAGCACTCCCGCCTTTTTTCGGCGGGAGAAGTAGGGTCAAGCCAGATTGATTAAGGTTATTAGCAATACGTAA

>811331a7-200b-46ae-9afc-51eccee0a941

ATATTGCGCTTCGTTCAGTTACGTATTGCTATTAGACCTGCAGTGAGAAGCAGTGTGTGGTAAAGTGGAATCATATAACTAGGCCGGATGTGAGAAGAAAAGCTGATAATGCTCCTGTTAGTGATCCAAGGAGCTTGGGTTGAGCTATGTGATAAGCATGGGTTTGGTGAGTCATTATGAATTATCATGTAAGTACAGGCTTACTAAGAGGGTAAATACATAGGCTTGAATTAAAAGGCTACACCCAACTCTAGGGTAATTAATAAAATAATAATGATAGTGATTGTGGAAGTGGAGAGGTAAATTGATAGAAGGATTAATGTTGTATCCCCAAGTAAATGCATTAATAGGTGACCTGCTGTAATATTGGCTGTTAATCGTACGGCTAAGGCTACAGGTTGAATGAATGAACTAATTGTTTCAATAATAATTAATATAGGGATTAGTGGATTGGGTATTCCTTTGTGGTAAAAAATGAGCGAGTTAGTTTTGTTTTAAATCCAGGCCTATTAGTACAGTTGCTGCTCATAGGGAATAGCTATGCCTAGATTTATTGACAGTTGGGTGGTTGATGTAGAAATGCATATGGTGTAAGTCCGAGAATGTTATTTAGAGACAATAAAAATTAGGGCTAGGAGTATAAGGGATCAGGTTCGCCCTTTAGTGGTGTGGGTTAATATTATTTGTTTAAGTGTTAGTTGAATTAGCCATTGTTGAATTGAAGAGAGTCGGTTGTTGAATAAGTTTTTGGAGGATAAAATTAATGTAGTGGGGGAGTGCAATAATTAAAAATACTAAGGGTACTCCTAGTATTGTTGGAATTGAATGAAGGAGCAAATAGATTTTGGTTTTCATTTTAGTTCTCAAGTTGTTTTATGTTTTTGTGTTTCTACTAATTTTGGTAGTGGTGTAATAATGGAAAGTAAAGTTCAGTATTTTCAATTGTATAATATAAAATAAAAGGTAACAATTATAGATATAATTACTATTGGTCACGGTGAAAGAAATATTTAGTTGGTATTCACTATAGAGTTTGTTCTCTCAATCTTTAACTTAAAGGTTAATGCTAAGTTAGCTTTACAGTGATACAATATATAAGTATGACTCATACTTCGAAATCTTGGAAATAAATGAATTCTAGAACAATGAGTATAAAGCTTGTGATTGGACCCGCAAATTTCCGAGCATTGTCCATAAAATATAGGCCTGTCGTATAGAGGCTAGTATGGCTTGGTTTAAACGTCCAGGAATTGCATCTGTTTTTACGCCTAGTGATGGTACGGCTCATGAGTGTAAGACGTCTTGTGATGAGATTAATATGCGAATATCGCTTCTATATAGGAATGAAAAGTTGTTCGGTTATCAACTTCGAGGAGTCCGAAATTCCCTGGCTCAAGGAAATATGTGGCATAATGTAAGAGTCAAATACTAAGTCTTCATAGTCGGAATATTCATAGGTTCAGTATCATTGACAATTGCTTTAAGGGTTAAATAAGGTTTATTAAATTCGTCTGTCATATAGAATACGCAGTGATGGGAGGGCAATTGTAATTAGAATCAGTGCAGGGAGAATAGTTCAGATAATTTTCGATCTCTTGAGCATTTATGGTGCTAGTATGAGTGAGTTTTGTGGTAAGTATAAGGAAATAATATATAAGACTAAGGAACTAATCAGAGAGGAAAATAATTATGAGTGCATGGTCGTGGAAAGCAATAAGTTCTTCTATGATAGGTGATGTGGCATTTGTAAACCTAGTTGAGCTGGTGTTGCTAAAGTAAGATATATAGATATTTAGTCTATAATTTAACTTTGACAAAGTTATGTAATTATTTTACTAATATCTTGTGAAAAAGTCATAGGGTATATGGGATTGGCTTGAAACCAATTTTTGGGGTTCAAATCCTTCCTTTTCGTCTAGGATTTAACGTAAGTTGCCTCTTCGAATGTGTGGTAGGAGGAGGGCAGCCGTATAATCATTCTAGATTGGTGGATAGTTGCTCAATGGCTAAGACTTTTCGTTTTGAAGAAGAAAGCTTCTCAAATTATAAAAACCATCAGGACTACTGCTGTAAGTGAAATAAAATGAACCGATAGATGAGACGATATTTCATGTAGTATATGCGTCTGGATAGTCTGAGTATCGTCGAGGTATTCCGGATAAGCCAAAGGAGATGTTGTGGGAAGAAAGTTAAATTTACGCCGACAAATATAGTGAAGTGGATTTTGGCATATGTTTGGTCAAGAGTATAGCCTGAAAAGAGCGGGAATCAGTGAATAAATCCCCCTATAATGGCAAATACTGCTCCTATTGATAACACATAGTGAAAGTGGGCTACTTTATAGTATGTATCATGTAATACAATATCTGATAATGATGGAGTTAGCTAACACAATTCCTGTTAGTCCACCCACATTAAAAGAAAATAAAGCCCAGGGCTCATAATATTGCGGGAGATCATTTGATATTACCGCCGTGCAGTGTGGCTAATCAGCTAAATACTTTTACTCAGTGGGAATAGCAATGATTATGGTAGCTGATGTAAATAATACTGCGAAGTATCTACATCTATTCCTACTGTGTGAATATGTGATGGAAAAATACATACAATAAAGCCTAGGAAACCAATAGATATTATGGCTCATACCATACCCATATAACCAAAGAGGTTTCTTTTATTGGGAATAATGTTACAATGTGTGAAGTATTCCAAAGCCCAGGCAAGATAGAATATATGGTGACCGAATCAGAATAAGTGTTGGTATAGGATAGGGTCGCCGCCACCAGCAGGATCAAAGAAAGTAGTATTTAAATTACGGTCAGTTAATAGTATGGTAATCCTGCAGCTAGAACTGGAAGAGAAAGTGAAGGACTGCAGTAATTAGGACGGATCACACAAACAACAGGTTTGATGATATTGAGTCATGGCTGGCGGTTTCATATTTACAATTGTTGTAAAGTTAATAGCTCCGAATAAAATACTGCTAAATGTGGTGAAAAATGGTTAAAATCTACAGAATGCACTGGGTGGGATAAAATTGCCTGCAGGGGTGGGTAGACTGTCCAGCAGTGCCGGCACCGGCTTCTAAAGTTGATGATGCAAGCAGTAGGGTAGGGAGGGTGGTAGAAGTCAAACATGTTATTTGTGAGGAAATGCTATATCAGGGCATAATTATTAAAGGAATAAGTCAGTTCCAAAACCCCAATCTCGTGGTATTACTATGAAAAAATTATGATGAATCGTGGGCGGTGACAATAACATTGTATACATGAGTCGTCTTCACAATGGGCTTCAGGTTGACCTAGTTCTGTTCGAATTAGGAGGCTTAGGGCTGTACCTACTGCCCCGCCCATGCGCCAAATAATAAATATAATGTCCCAATGTCTTTGTGATTAGTTGAAAATAGTCGAAGGTTTATGAACATAGGTAGGGGTAAATGGCTGAGCAAGCATTAGACATAAATCTAAAGACAGAGGAGTGAACCCTCTTTTACCAGCCCTGAGGTGAATATCATTGAATTGCAAAATTCAAAGCAGCTTCAATTCTGCCGGGGCTTCTCCCGCCTTTTTTCCCCTAGCGGCCGGAGAAGTAGGACAACCAGTTGATTTAGGTTATTTAGCTGTTAACTAAATTTTTGTGGGTTAAAATCCCCATCAATCTAGGAAGGCTTAGCTTAATTAAAGTAATTGATTTGCGTTCAGTTGATGCAGAATAAAGTTTTGCAGTCCTTATTTGTTGTGCAGAGCAATACGTAACTA

>5366b981-f091-41a4-83af-ba766bc692bc

CGGTATTACTTCGTTCAGTTACGTATTGCTGCAGTAATTAGGACGGATCACACAAACAACGGAGTTTGATATTGAGTCATGGCTGGCGGTTTCATATTTACAATTGTTGTAATAAAGTTAATAGCTCCAAGAATAGAAGAAATGCCTGCTAAATGTAGTGAAAAAATGGTTAAATCTGAAACAACCATAGGTGGGATAAATTGCCTGCTAGAGATTGGGTAGACTGTCCAACCAGTGCCGGCACCGGCTTCTAAAGTTGATGATGCAAGCAGTAGGAGTAGGAATTGGTAGAAGTCAAGCTTATGTTATTTATTCGAGGAAATGCTATATCGAGGCGCCAATTAATAAAGGAATAAGTCAGTTCCCAAAACCCCCAATCATAATTGGTATTACTATGAAAAAATTATGATGAATGCGTGGGCAGTGACAATAACATTGTATACATGGTCGTCTTCTATTAGGCTTCCGGAGTTGTCCTGGTTCTGTTCGAATTAGGAAGCTTGGGAGCTGTACCTACTGCCCCCACTTATGCATAAATAATAAATATAATGTCCCAATGTCTTTGTGATTAGTTGAAAATAGTCAGCGGTTTATGAACATAGGTAGGAGTAAAATGGCTGAGCAAGCATTAGACTGTAAATCTAAAGACAGAGTGAACCCTCTTTTTACCAGCCCTGAGGTGAACTATCACATTAGTGCAAATTCAAAGAAGGCAGCTTCAATTTGCCAGGGCTTCTCCTTTCCCTAACGGCGGGAGAAGTAGATTGAAGCCAGTTGATTAGGTTATTTAGCTGTTAAGCTAAATTTTTGTGAGTTAAATCCCATCAATCTAGGAAGGAGCTTAGCAATTAAAGTAATTGATTTGCGTTCAGTTGATGCAGAATAAGTTTTGCGGTCCTTATTTGTTGTGCAGAAATTAAGTAAAATTTACTTACTAAGGGCTTTGAAGCTCTTGGTCTTATTAACCTAAATTTCTAGGGTATAAGTAAGCAATATACGTAACTA

>189daf54-d7f2-4633-8a37-55df8d6c5e2b

AACTTCGTTCCAAGTTACGTATTGCTTTTACAATTGTTGTAATAAAGTTAATAGCTCCAAAGAATAGAAGAAATGCCTGCTAAATGCTTAATTGAAAAAATGGTTAAATCTACAGAGGAAGCTCCTGAATTGGGATAAATTGCCTGCTAGGGGTGGGTAGTTAGCCAACCAGTGCCGTATCAACTAAAGTTGATGATGCAAGCAGTAGGAGTAGGGAGGGTGGTAGAAAGTCAGAAGCTTATGTTATTTATTCGAGGAAATGCTATATCGGGAGCGCCAATTATTAAAAGGAATAAGTCAGTTCCCAAAACCCCAATCATAATTGGTATTACTATGAAAAAAATTATGATGAATGCGTGGGCGGTGACAATAACATTGTATACATGGTCGTCTTCTATTAGGCTTCCGGGTTGTCCTAGTTCTGTTCGAATTAGGAAGCAGGGTATACCTGCCCCCCGCCCATGCCTCAAATAATAAATATAATGTCCCAATGTCTTTGTGATTAGTTGAAAATAGTCAGCGGTTTATGAACATAGGTAGGGGTAAAATGGCTGAGCAAGCATTAGACTGTAAATCTAAAGACAGAGGAGTGAACCCTCTTTTACCCAGCCTGAGGTGAACTATCACATTGAATTACCAAATTCAAGAGCAATACGTAACC

>ecd1e93d-61dd-49a5-9b23-b09e0d25a33e

ATGTACTTCGTTCAGTTACGTATTGCTACAGGTGGTGAGAAAGTGGTTCCGATAATTACGTAGAAGGCCATGAAAGCCTGTAGCCATAAAAAATGGATCATATACTCGTCAGAGATGCGTAAATGGGGGAACTAGGAATATTCTGATAATTGTAGGCAGGTGAAGTAGATCCTAATACCAATAGTTAGGAGTAGTGCTTGGGTTGATTCTTTTCGGTTAGCTTCTATGAGGCTGTGATGTGCTCATGTAATTGTAACTCCTGATGCTAGTAGTACGGTTGTATTTAAGAGAGGGACTTCTATTGGGTTGAGGAAGTAATGCCTGTAGGTGGTCATAGTCCCCCTGTTTTGTGGAGTTGGGGCTGGACTAGAATGATAAAATGCTCAGAAGAAACCTGCAAAGAAGAAAATTTCTGAAATGACAAATAGAAATTATTCCGTATCGTAGGCCTTTTTGGACAGGTGCAGTGGTGAGCCCTTGATATGTACTTTCTCGTACTACATCACGTCATCATTGAAATATTGTTATAGAACTAGCTAGTAGACCTGCAGTGAGAAGCAGTGTGTGGTAAAGTGGAATCATATAACTGAACCACGGATGTGAGGAAAAAGCTGATGGTGCTCCTGTTAGTGGTCAAGGGCTTAGGTTGACTATGTGATAAGCATGGGGTTTGGTGAGTCATTATGAATTATCGTAAGTACAGGCTTACTAAGAGGGTAAATACATAGGCTTGAATTAAGAGGCTACACCCAACTCTAGGGTAATTAATAAAATAATAATAATGATAGTGATTGTGGAAGTGGAGAGGTAAATTGATAGAAGAGTTAATGTTGTATCCCAAGGTAAATGCATTAATAGGTGACCTGCTGTAATATTGGCTGTTAATCGTACGGCTAAGGCTACAGGTTGAATGAATAGACTAATTGTTTCAATAATAATTAATATAGGGATTCGTGGAATTGGTGTTCCTTGTGGTAAAAAATGAGCGAGGGATGATTTTTGTTTTAAATCGGAGGCCTATTAGTACAGTTGCTGTAGCAGAAGGAGCAGCTATGCCTAAGTTTATTGACAGTTGGGTGGTTGGTGTAAATGCATATGGTGTAAGTCCGAGAATGTTATTTAGGGCAATAAAAGAAATTAGGGCTAGGAGTATAAAGGGATCAGGTTCGCCCTTTAGTGGTGTGGGTTAATATTATTTGTTTAAGTGTTAGTTGAAGACTGTATTGAATTGAAAGTGATCAGTTTGATGGAATAAGTTTTAGAGGATAATTTAATGTAGTGGGGAGTGCAATAATTAAAAATACTAAGGGTACTCTAGTATTGTTGGAATATTGAATGAGGCAAATAGATTTTAGTTCATTTTTAGTTCTCAAGTTGTTTTATGTTTTTGTGTTTCTACTAATTTTGGTAGTGGGTAATAATATGGAAAGTAAAGTTCAGTATTTTCAATTGTATAATATAAAATAATTGCAACAATTATAGATATAATTACTATTGGTCACGGTGAAATATTTAGTTGAGGCATTCACTATAGAGAGTTTGTTCTCTCAATCTTTAACTTAAAAGGTTAATGCTAAGTTAGCTTTACAGTGATACAATATATAAGTATGAAGCTCATATACCGAAATCTTGGAAATAAATGAATTCTAGAACAATAGGTATAAAGCTGTGATTGGACCACAAATTTCCGAGCATTGTCCATAAAATAGGCCTGGTCGTATAGAGGCTAGTATGGCTTGGTTTAAGCCTACGTCCAGGAGTACATCTGTTTTTACGCCTAGTGATGAAATTTTTGGCTCATGAGTGTAGACGTCTTGTGATGAGATTAATATGCGAATATCCGCTTCTATAGGTAAAGTTGTTCGGTTATCAACTTCGAGAGTCGAAATTCCCACTGGCTCAAGGAAATATGTTGGCATAATGTAAGAGTCAAATACTAAGTCTTCATAGTCGGAATATTCATAGGTTCAGTATCATTGGTGACCAATTGCTTTAGGGTTAAAATAAGGTTTATTAAATTCGTCTGTCATATATAGAATACGCAGTGATGGGAGGGCAATTGTAATTAGAATCAGTGCAGGGAGAATAGTTCAGATTATTTCGATCTCTTGAGCATTTATGGTGCTAGTATGAGTGAGTTTTTGTGGTAAGTATAAGGGAAATAATATATAAGACTAAGGAACTAATCAGGAAAATAATTATGAGCGAGTGCATGGTCGTGGAAAGCAATAAGTTCTTCTTAATGATGGGTGATGTGACATTTGTAAACCTAGTTGAGCTGGTGTTGCTATTAAGATATATAGATATTTAGTCTATAATTTAACTTTGACAAAGTTATGTAATTATTTTACTAATATCTTTATTGAAAAAGTCATAGGTATATGGGATTATTGAAACCAATTTTGGGGGTTCAATCCTTCCTTTTTCGTCTAGGATTTAACGTAAGTTGCCTCTTCGAATGTGTGGTAAGAGGAGGGCGGCAAGCTATAATCATTCTAGATTGGTGAGATAGTTGCTCAATGGCTAAGACTTTTCGTTTTTGAAGAAAGCTTCTCAAATTATAAAAACCATCAGGACTACTGCTGTAAGTGAAATAAATGAACCGATAGATGAGACGATATTTCATGTAGTATATGCGTCTGGATAGTCTGAGTATCGTCAATTATTCCGGATAAGCCAAGGAAGTGTTGTGGGAAAGTTAAATTTACGCCGACAAATATAATGGTGAAGTGGATTTTGGCATATGTTTGGTCAAAGTATAGCCACAAGGTGGCGGGAATCAGTGAATAAATCCCCTATAATGGCAAATACTGCTCCTATTGATAACACATAGTGAAAGTGGGCTACTACATGGTAACCATGTCATGTAATACAATATCTAATGATGAGTTAGCTAACACAATTCCTGTTAGTCCACCCACGGTAAAAGAAAAATAAAGCCCAGGGCTCATAATATTGCGGGGAGATCATTTGATATTACCGCCATTAGGTATTAATACTAATCAGCTAAATACTTTTACTTCCCGGTACGGGAATAGCAATGATTATGGTAGCTGATGCAAAATATGCGAGTATCTACATCTATTCCTACTGTGAATATGCTGGATAAGGCTCATTACAATAAAGCCTAGGAAACCAATAGATGTTATGGCTCCTTCATGCCCATATAACCAAAGGGTTCTTTTTTATTAGAATGATGTATTTACAATGTAGAAATTATTCCAAAACCGGTAAGATTAGAATATATACTTCAGGGTGACCGAAGAATCAGAATAAGTGTTGGTATAGGATAGGGTCGCCGCCAACCGGATCCGAAAGTAGTATTTAAATTACGGTCAGTTAATAGTATGGTAATCCCTGCAGCTAGAACCTTGAAGAGAAAGTAAAAGAAGGATTACGGTAATTAGGACGGATCCCACAAACAACGGAGTTTGATATTGAGTCATGGCTGGCAGTTTCATATTTACAATTGTTGTAATAAAGTTAATAGCTCTCCAAAGGCTAAGACAAGAATGCCTGCTAAATGTAGTGAAAAAATGGTTAAATCTACAGAGGCTCCTGGGTGGGATAAATTGCCTGCTAGGGGGTGGGTAGACTGTCCAACCAGTGCGGCACCGGCTTCCTAAAGTTGATGATGCAAGCAGTAGGAGTAGGGAGGGTGGTAGAAGTCGAAGCTTATGTTATTTATTCGAGGAAATGCTATATCAGGAGCGCCAATTATTAAAGGAATAAGTCAGTTCCCAAAACCCCCAATCATAATTGACATACTATGAAAAAAATTATGATGAATGCGTGGGCGAGCTGACAATAACATTGTCCCTTGGTCGTCTTCTATTAGGCTTCCGGGTTGTCCTAGTTCTGTTCGAATTAGGAGGCTTAGGGCTGTACCTACTGCTTGCCCCCGCCCTGCGCCAAATAATAGATATAATGTCCCAATGTCTTTGTGATTAGTTGAAAATAGTCAGCGGTTTATGAACATAGGTAGGGGTAAAATGGCTGAGCAAGCATTAGACTGTAAATCTAGAACAGAGGAGTGAACCCTCTTTTTACCAGCCCTGGAGGGTGAATATCACATTGAATTGCAAAATTCAAAGAAGCAGCTTCAATTCTGCCAGGGCTTCTCCCGCCTTTTTCCCTAACGGCGGGAGAAGTAGATTGAAAGCCAGTTGATTAGGTTATTTAGCTGTTAACTAAATTTTTGTGGGTTAAAATCCCATCAATCTGATGGGAAACAGCTTAATTAAAGTAATTGATTTGCGTTCAGTTGATGCAGAATAAGTTTTTGCAGTCCTTATTTGTTGTGCAGAAATTAGTAAAATTTACTTACTAAGGCTTTGGACTCTTAGTCTTATTAACCTAAATTTCTAAGTTATAAGTATTAGTGGAGTTAGGGGTAGGAGACAGGTAGAACACTACAAGTGGGGATAGAAACGGTATTGGCTTTATATAATTTAGTTGCCAGTTGATTTTTGTGTTATTTGATGTTGGGAATATTGTCATTGAGATATAATATATTAAACGTATATAAAAATATAATTTATTAATGTTATTAAAGCTATTATAAGGGAATAATTAAGTTATCATTTTTAATAAGTTCTTGTATAATAGCTCATTTGGGGAAAAAACATATTGAGTGGGGAGTGGGAGCCTCCTAGGGATATTATTATTAGTGGAATAATGAATTATTGTTCATGCTGGTTGTTTCAGGTATGTGATAGGGATAGGGTTGTTACATTTGAGGATTAAATAAAAGATTATTAATGTAGAGATTGTTAGGAAAATATAAATAATTAAAATTAATATAGTAATATTAGGGTCGTAATATAATACTGCTATTATTCATCCTATGTGAAGTAATTGAAGAATAGGCTAGGATTTGCGGAGTTGTGTTTGGTTGAGTCCTCCTCAGCTGCCAATTATATTGATGGGACGAGATTGTTAGAATCAAGTTTAGGTTTGTTGACAGGAAAAATTTGGAGGAGAATTGATATTGGGCAGTTTTGTCACGTGAGAATAATTATAGCTGGGATTAGAGGGATTCCTTGGGTAATTTCTGGGAGTCAGAAGTGAAGTGGGGCTATCTATTTTTATTGCTAGAGCAATAGATGTTATTGTGGATAATATTTGGTTATAAGGAGGATTGATTGTTCATTGTTGAGTTGGTAGGTTATTAGGGAAAATGGATACTAATGAAAAGTGATGCTGTTGCTTGAATTAAAAAATATTTGGTGGATGCCTCTGTGGGCGGGATTGGTACTTTTGGCAAGTTGGTACAGTGGCTAGTATATTTAATTCTAAGCCTATTCAGGCTAGGAATCAATGTGAGCTTAAAATTGTGATTACGGTCCCTGTTAGAATGGTGAAGGAGATAATAAGGTGGGCTAGAGAGTTAATGTTAGCATGGGAAGGATTAAACCAACATTTTCTAAGGTATGGGCCCAAGATGGCTTATTAGCTGACCTTACTGTTTAGGATATGGTGTAATCAGTGGCACGGAGTTTTTGAGAGTTCTCAGGTGTAGGATTCAATTCCTGTAATCCTAGAAATAAGGGTTTGAACCTCTATAATTTACTCTATCAAAGTAACTCTTTTATCAGACATATTTCTTATGTTTGGGGTGGGATGCCAGATGCTAGGATGGGTATTGAAACATATCATATACATAGTGCTAATGTAAGTGGTAAAAAGTTTTTTCATAGTAAGAATATTAATTGATCGTAGCGGAAGCGGGGTAGGCTGTTCGAATTCATAAAAATGAGTGGTTAGTAGGAGGGTTTGGCTATAAAATTAATAGTAAATGTTTCTGGTATAATTATATTGTAGGGTGTGGATAGAAAATAGTGGTAGTTAGGGCATTTATTATAATAATATTTATATACTCTGCTATGAAGAATAAGGCGAATGAGCCTGCAGCGTATTCAATGTTGAAACCTGATACTAGTTCTGATTCGCCTTCTGTCAGGTCGAATGGAGCTCGGTGGATTTCTGCTAATGTGGAAATAAATCATATTGTAGCTAGGGGCATGACGATAGAAGTCAGTAGTGTTCTTGAGTGGTAATAAGCGATTGTAAATTGAATGAGCCGCTTATTAGTAGTGTTGATAGTAAGATAATGGCAAGGGTGACCTCATATGAGATTGTTTGGGCTACAGCTCGTGGTGCGCCAATTAGTGCGTAGTTTGAGTTGGATGCTCAGCCAGATCATAAAATTGAGTAAACAGCTAGGCTTGATGTTGCGAGGATAAATAGGAGACCAAGATTGAAGTTGATAAGAGGATATGGTATGGGGAGTGGCGTTCACTGGTAGAGCAATGGAAAAGGCAGGAGTGAGGGGCAGTTAAATATAAAGTTATGGTGGATGTGGTAGGTAATAAGGGTTCTTTTCAGCGAAGAGTTTTATGGCGTCAGCGATTGGTTGAAGTATTCCGTAGGGCCTACAATATTGGGAGCCTTTTCGGAGTGTATATAGCCAAGTTTTCGTTCTGTGAGTGTTAGAAAGGCTATGGCAATTAGGGCAGGTAGGACTAGTATTAGTAAATTAATTATATACATGTTGTTAAGAAGAGGAATTGAACCTCTGATTATAAAGTTTTAAGTTTTATGCAATTACCGGGCTCTGCCACCTTAACGAGCCTGTTCTTGGGCAAGACCCATATATGTGAGTTGAGATGGAGGTCATCTGATTGTTGAGGCCTTTATGAAGTGGGCCCCATTTCTCTTGTCCTTTCGTACTGGGAGAAATATTTAAATAGATAGAAACCGACCTGCTTGCTCCGGTCTGAACTCAGATCACGTAAGACTTTAATCGTTGAACAAACGAACCCTTAATAGCTTCTGCACCATTAGGATGTCTTGATCCAACATCGAGGTCGTAAACCCTATTATCGATATGGACTCTAAAATAGGATTGCGCTGTTATCCCTAGGGTAACTTGGTCCGTTGATCAAATTATTGTGGGTCAATAGGTGCTGCTTACTTAGACTGGTAAGGTCTTGGTATATGTTTTTCGGAGGTTCTATTTTACTCCGAGGTCGCTAAACCGAAATTTATAATACATTGACGGTAAATTAATGCCTGTTGGTTTTTACAAATCTGAGTTTCGTATCATTAAATTAAAGCTCCACGAGGGTCTTCTCGTCTTATTTATGTATATCCGCCTCTTCACGGATGGTCAATTTCACTGATTAAAAGTAAGAGACAGTTAAACCCTCGTGTGGCCATTCATACAAGTCCCTATTTAGAGAACAAGTGATTATGCTACCTTTGCACGGTCAGGGTACCGCGGCCGTTGAACATATGTCACTGGGCAGGCAGTGCCTCCTAATACTAGAAATGCTGAAGGTGATGTTTTTGGTAAACAGGCGAGTTTAGAGCTTGCCAGTTCTTTACTTTTTAATCTTTCCTTAGTGCATGCCTGTGTTGATTTAACAGTTTAGTTGAAATTGATATTTTAATTTGTAGTTTATGATTATTAGGCTGTTAACTAACAGTAGTTGTTTCGGTCTAGTAAGCAGCAAGGAAGTAGTTTCATGTTACTTATACTAACATTATTGCTTCTATTAGATAATAGATTAATCCAATTTGTTTCAGAA

>516c8f55-347e-4934-b436-0e7bec17aa48

AGTAATACTTCGTTCAATTTTATGTATGATAGGCTCATACAATAAAGCCTAGGAAACCAATAGATGTTATGGCTCATAAATATGCCCTCTTAAAGGGTTCTTTTTATTAGAATAATATGTTACAATGTGTAGAGTATTCCAAAACCCGGTAAGATTAGAATATATACTTCAGGTGACCGAAGAATCAGAATAAGTGTTGGTATAGGATAGGGTCGCCGCCACCAGCAGGATCTAAGAAAGTAGTATTTAAATTACGGTCAGTTAATAGTATAGTAATCCCTGCAGCTAGAACTGGAGAGAAAGTAAAAGAAGGACTGCAGTAATTAGGACGGATCACTACAAACACTTGAGTTTGATATTAGTCATGGCTGGCGGTTTCATATTTGATGTTGTAATAAAGTTAATAGCTCCAAGAATAGAAGAAATGCCTGCTAAATGTAGTGAAAAAATGGTTAAATCTACAGAGGCTCCTGGGTGGGATAAATTGCCTGCTAGAGGGGTGGGTGGAGCTGTCCCAGCCAGTGCCGGCACCGGCTAAAGTTGATGATGCAAGCAGTAGGAGTAGGGAGGGTGGTAGAAGTCAGAAGCTTATGTTATTTATTCGAGAAATGCGTATCCGGGAGCGCCAATTATTAGGAATAAGTCCAGTTCCCAAAACCCCAATCATAATTGGTATTACTATGAAAAAAAATTATGATGAATGCGTGGAGGTGAAACGACAATAACATTGTATACATGGTCGTCTTCTATTAGGCTTCCGGGTTGTCCTAGTTTGCTGTTCGAATTAGGCTTAGAGGGCTGTACCTACTGCCCCGCCCATGCGCCAAATAATAAATATAATGTCCCGTGTCTTTGTGATTAGTTGAAAATAGTCAGCGGTTTATGAACATAGGTAGAGCAATACGTAATGGCA

>d613bb10-1c7e-4732-9e53-715bcde4099d

AGTGTACTTCGTTCAGTTACGTATTGCTGTAAGTGAAATAAATGAACCGATAGATGAGACGATATTTCATGTAGTATATGCGTCTGGATAGTCTGAGTATCGTCGAGGTATTCCGGATAAGCCAAGGAAGTGTTGTGGGAAGAAAGTTAGATTTACGCCGACAAATATAATGGTGAAGTGGATTTTGGCATATGTTTGGTCAAGAGTATAGCCTGAAAAGAGCGAGGAATCAGTGAATAAATCCCCCTATAATGGCAAATACTGCTCCTATTGATAACACATAGTGAAGTGGGCTACTACATAGTATGTATCATGTAATACAATATCTAATGATGAGTTAGCTAACACAATTCCTGTTAGTCCACCCACGGTAAAGAAAAAATAAAGCCCAGGCTCATAATATTGCAGGAGATCATTTGATATTACCGCCGTGCAGTGTGGCTAATCAGCTAAATACTTTTACTCCAGTGGGAATAGCAATGATTATGGTAGCTGATGTAAAATATGCACAGTATCTACATCTATTCCTACTGTAGATATGTGATGGGCTCATACAATAAAGCCTAGAAACCAATAGATAATATGGCTCATACCATGCCCATATAACCAAAGGGTTCTTTTTATTAGAATAATATGTTACAATGTGAAATTATTCCAAAACCCGGTAAGATTAGAATATATACTTCAGGGTGACCGAAGAAATCAAGAATAAGTGTTGGTATAGGATAGGGTCGCCGCCACCAGCAAGGATCAAGAAAGTAGTATTTAAATTACGGTCAGTTAATAGTATAGTTAATCCTGCAGCTAGAACTGGAAGAGAAAGTAAGAAGGACTGCAGTAGGACGGATCACACAAACAACGGAGTTTGATATTGAGTCATGGCTGGCAGTTTCATATTTCACAATTGTTGTAATAAAGTTAATAGCTCCAAAGAATAGAAGAAATGCCTGCTAAATGTAGTGAAAAAATGGTTAAATCTACAGAGGCTCCTGGGTGGGATAAAGTGCCTGCTAGGGGTGGGTAGACTGTCCAACCAGTGCCGGCACCGGCTTCTAAAGTTGATGATGCAAGCAGTAGGAGTAGGGGAGGGTGGTAGAAGTCGAAGCTTATGTTATTTATTCGAAATGCTATATCGGGAGCGCCAATTATTAAAGGAATAAGTCAGTTCCCAAAACCCCAATCATAGTGGTATTACTATGAAAAATTATGATGAATGCGTGGGCGGTGACAATAACATTGTATACATGGTCATTCTATTAGGCTTCCGGGTTGTCCTAGTTCTGTTCGAATTAGGAGGCTTAGGGCTGTACCTACTGCCCCCGCCCATGCGCCAAATAATAAATATAATGTCCCAATGTCTTTGTGATTAGTTGAAAATAGTCGATGGTTTATGAACATAGGTAGGGGTAAAATGGCTGAGCAAGCATTAGACTGTAAATCTAAAGACAGAGGTGAACCCTCTTTTTACCAGCCCTGGGGGTGAACTATCACGTATGAATTGCAAATTCAAAGAAGCAGCCTATTCTCTGCCAGGGCTTCTCCGCCTTTTTTTCCCTAACGGCGGGAGAAGTAGATTGAAACCAGTTGATTAGGTTATTTAGCTGTTAACTAAATTTTTGTGGGTTAAATCCCATCAATCTAGGAAGGGCTTAGCTTAATTAAAGTAATTGATTTGCGTTCAGTTGATGCAGAATAAGTTTTGCAGTCATATTTGTTGTGCAGAAATTAAGTAAAATTTACTTACTAAGGGCTTTGAAGGCTCTTGGTCTTATTAACCTAAATTTCTAAGTTATAAGTATTAGTGGAGAGCAATACGTAACTA

>cc447376-2940-4159-8b3b-277292315b4e

AGTGTGCTTCGTTCGTTACGTATTGCTCAGGCTTACTAGAGGTAAATACATAGGCTTGAATTAAAGGCTACCAACTCTAGGGTAATTAATAAAATAATAATAATGATAGTGATTGTGGAAGTGGAGAGGTAAATTGATGAAGAAGGTTAATATGCTTGTATCCCCAAGTAAATGCATTAATGACCTGCTGTAATATTGGCTGTTAATCGTACGGCTAAGAATACAGGTTGAATGAATAAGCTAATTGTTTCAATAATAATTAATATAGGGATTAGTGGAATTGGTGTTCCTTGTGGTAAAAAATGAGCGAGGGGATGATTTTGTTTAAATCAGAGGCCTATTAGTACAGTTGCTGCTCATAGAGGAATAGCTATGCCTAGATTTATTAACGAGATTGAGTGGTTGGTGTAAATGCATATGGTGTAAGTCCGAGAATGTTATTTAGGGCAATAAAAGAAATTAGGGCTAGGAGTATAAGGATCAGGTTCGCCCTTTAGTGGTGTGGGTTAATATTATTTGTTTAAGTGTTAGTTAGATAGCCATTGTTGAATTAGAAGAGAGTCGGTTGTTGAATAAGTTTTTGGAGGATAAAATTAATGTAGTGGGGAGTGCAATAATTAAAAATACTAGGGTACTCCTAGTATTGTTGGAATATTGAATAGAACGATAGTTTTGGTTCATTTAGTTCTCAAGTTGTTTTATGTTTTTGTGTTTCTACTAATTTTTGGTAGTGGGTAATAATGGAAAGTAAAGTTCAAGTATTTTCAATTGTATAATATAAAATAAGGTAACAATTATAGATATAATTATAAACGGTCACGGTGAAATATTTAGTTGAGGCATTCACTATAGAGAGTTTGTTCTCTCAATCTTTAGCAAAAGGTTAATGCTAAGTTAGCTTTACAGTGATACAATATATAATGCCGACTCATACTTGAAATCTTGAAATAAATGAATTCAGAACAATAGGTATAAAGCTGTGATTGGACCCGCAAATTTCAAAGGCATTGTCCATAAAATAGGCCTGGTCGTATAGAGGCTAGTATGGCTTGGTTTAAACGTCCAGGAATTGCATCTGTTACACGCCTAGTGATGGTACGGCTCATAGGTGTAAGACGTCTTGTGATGAGATTAATATGCGAATATCCGCTTCTATAGGTAAAGTTGTTCGGTTATCAACTTCGAGAGTCGAGAATTCCCTGGCTCAAGGAAACTGTTGGCATAATGTAGAGTCAAATACTAAGTCTTCATAGTCGGAATATTCATAGGTTCAGTATCATTGGTGACCAATTGCTTTGAGTTAAATAAGGTTTATTAAATTCGTCTGTCATATATAGAATACGCAGTGATGGGAGGGCAAATTGTAATTAGAATCGGTGCAGGAGAATAGTTCGAATTATTTTGATCTCTTGGTATTTTTATGATTTTTAATGTGAGTGAGTTTGTGAAGTAGTATAAGGAAATAATATATAAGACTAGAACTAATCTGAAGGAAAATAATTATGAGTGCATGGTCGTGGAAAGCAATAAGTTCTTCTATGATGAGTGATGTGGCATTTTGTAGCTAGTTGAGCTGGTATTTTGCTATTAAGATATATAGATATTTAGTCTATAATTTAACTTTGACAAAGTTATGTAAATTATTTTACTAATATCTTATTGAAAAATCATGAGTATATAGGATTGGCGACCAATTTTTGGGGGTTCAAATCCTTCCTTTTTCGTCTGGTTTAACGTAAGTTGCCTCTTCGAATGTATCGAAGTAAGGAGGAGGGCGGCGGCATAATCATTCTAGATTGGTGGATAGTTGCTCAATGGCCAGACTTTTCATTTTGAAGAAGCTTTACTCAAATTATAAAAACCATCAGGACTACTAACTTGGCGAGTGTGAAATAAATGAACCGATGGATGAGACGATATTTCATATAGTATATGCGTCTGGATAGTCTGAGTATCGTCGAGGTATTCCGGATAAGCCAAGGAAGTGTTGTGGGAAGAAGATTAGATTTACGCCGACAAATATAATGGTAGTTGGATTTTGGCATATGTTTGGTCAAGTATGGCCTGAAAAGAGCGGGAATCAGTGAATAAATCCCCCTATAATGGCAAATACTGCTCCTATTGATAACACATAGTGAAAGTGGGCTACTACATAGTATGTATCATGTAATACAATATCTAATGATGATTAGCTAACACAATTCCTGTTGAATCACCCACGGTAAAAAGAAAAGCCAAGCAGGGCTCATAATATTGCGGGAGATCATTTGATATTACCGCCGTGCAGGTGGCATGGCTAATCAGCTAAATACTTTTACTCAGTGGGAATAGCAATGATTATGGTAGCTGGTGTAAAATATGCAGGTATCTACATCTATTCCTACTGTGAATATGTGATGGGCTCATACAATAAAGCCTAGGAAACCAATAGATATTATGGCTCATACCATGCCCATATAACCAAAGGGTTCTTTTTTAATAGAATAATATGTTACAATGTGGTGAAATTATTCCAAAAACCCGGTAAGATTAGAATATATACTTCAGGGTGACCGAAATCAGAATAAGTGTTGGTATAGGATAGGGTCGCCGCCACAGCGGGATCAAAGTAGTATTTAAATTGCGATTAAGTTAATAGTATGGTAATCCCTGCAGCTAGAACTGGAGAGAAAGTAAAAGAAGGACTGCAGTAATTAGGACGGATCACTGAAACAGCGGAGTTTGATGTTGAGTCATGGCTGGCGGTTTCCTTATTTACAATTGTTGTAATAAAGTTAATAGCTCCAAGAATAGAAGAAATGCCTGTAAATGTAGTGAAAAATGGTTAAATCTACAGGGGCTCTGGGTGGGATAAATTGCTAGGGGTGGGTAAACTGTCCAACCAGTGCCGGCACCGGCTTCTAAAGTTGATGATGCAAGCAGTAGGAGTAGGGAGGGTGGTAGAAGTCAGAAGCTTATGTTATTTTGTTAAATGCTATATCAGGAGCGCCAATTATTAAGGAATAAGTCAGTTCCAAACCCCCAATCATAATTGGTATTACTATGAAAAAAATTATGATGAATGCGTGGGCGGTGACAATAACATTGTATACATGTTAAATCTTCTATTAGGCTTCCGGAGTTGTCCTAGTTCTGTTCGAATTAGGAGGCTTAGGGCTGTACCTATTTACCCTTTTTACTCCAAATAATAAATATAATGTCCCAATGTCTTTGTGATTAGTTGAAAAATAGTCACGGCAGTTTATGAACATAGGTAGGGAATTAAAATGGCGACAAGCATTAGACTGTAAATCTAAAGACAGAGGAGTAGGCCTCTTTTTACCAGCTGAGGCGAACTATCACATTGAATTGCAAATTCAAAGAAGCAGCTTCAATTCTGCCGGGGCTTCTCCCACTTTTCCCCTAACGGCAGGAGAAGTAGATTAGCTCAGTTGATTAGGTTATTTAGCTGTTAACTAAATTTTTGTAGGTTAAAATCCCATCAATCTAGGAAGGGCTTAGCTTAATTAAAGTAATTGATTTGCGTTCAGTTGATGCAGAATAGGTTTTGCAGTCCTTATTTGTTATTGCAGAAATTAAGTAAAATTTACTAAAAGGGCTTTGAAGGCTCTTGGTCTTATTAACCTAAATTTAGTTATAGTATTAGTGGAGTTAGGGGTAGGAGACAGGTGAAGACACTACAAGTGGGATAGAAACGGTATTGGCTTTATATAATTTGGTTACAGTTGATTTTGTGTTATTTGATGTTGGTATTGTCATTGAGATATATTGTATGTTAAACGTATATAAAAATATAAATTTATTAATGTTAGTAAAGCTATTATAGAGATAATTAAGTTATCATTTTTAATAAGTTCTTGTATAATAGCTCATTTGGGGAAAAACCTGTTAGTGGGGGTAGACCTCTAGGGATATTATTAGTGGAATAATGGGTATTGTTCATGCTAGTTGTTTCAAAATTATGTGATAAGGATAGGGTTATTCATTTGAGGTTAAAGCCAAGAATTTATTAATGTAGAGATTATTGGAAAATATAAATAATTAAAGTTAATATAGTAATATTAGGGTCGTAATATAATACTGCTATTATTCATCCTATGTGAGTAATTGAATAAGCTAAGTTTTCGGAGTGTGTTTGGATTGAGTCCTCCTCAGCTGCCAATTATAATTGATAGAACTGAGATTGTTAGAATCAAGTTTAGGTTTGATGACAGAAAAATTTGGAGGAGAATTGATATTGGGGCGAGTTTTTTGTCGTGAGAATAATTGCAGCTGGGATAGAGGGATTCCTTGGGTAATTTCTAAGTCAGAAGTGAAGTGGGCTATCCTATTTTTATTGCTAGAGCAATAAATATTATTGTGGATGTGTGTTGGTTGTCGGGAGGATTGATTGTTCATTGTTGAGTTAGTAGATTGTTAAGAAATGGATACTAATAGAGAAGTATTGATGCTGTTGCTTAGTTAAAAAATATTTGGTGGATACCTCTGTGGGGTAGGGATTGATACTTTTAGCAGAGTTGGTACGATGCTTCTAGTATGGCCCAATTCTAAGCCTATTAAACAGGAATCAATGTGAGCTTAAAATTGTGATTACGGTCCCTGTTAGAATGGTGAAGGAGATAATAAAAGAATTGGCTAGAGGATTAATGTTAGCACGAGAAGGATTAAACCAACATTTTCGAGCAATACGTAACTT

>ae3ded2c-6d18-451c-8f61-9d913fbc42b1

GTATTGCTTCGTTCAGTTACGTATTACTGGTTAGAATTATTTGTTGGGTTGCCCATGGTAAAGGGGTAGAAGTAGGGCAATTTCCAGATCAAATAGGAGAAATGTGATGGCGATTGGAAAAATTTTATGGAGAATGGTAAGTGGGCGGAGGTTGTAGGATCAAATCCGCACTCGTAAGGGTTGACCTTTTCCTATTATAAATATTTAATTGTGGAAGTCAAAATGTAATTGTGATAAGAAGTAGGTAAATCAATGTTAGTCATTAGGTTTCAAATTAAATTTATAATACTCTCGAATCATTCGAGGCCCGTTGATTGGAAGTCAATAATACTTTTTATACTAAGAAGTGAGGAGACCTCATCCAAGTAGATGGAAATATAGAGGGAAAAGTCATACTACATCTACGAAATGTCAATATCATCTGCAGCGGCTGAAGCTGAAATGGTGGCTAGATGTGAAGTGATATAATTGTTGGCGAAAGTAACAGGTGGTGAGGAAAATGGTTCGATAATTACGTGAAGGCCATGAAAGCTGTAGCCATAAAAAATGTGAATCCATATGCTCAAATCCCAGGAGAGATAGTAAATGGAGCTTCAGAATATTCCTGATAATTGTAGGCAGGTGAAGTAGATCCTAATATAATGAGTTAGGAGTAGTGGCAAGGTTGATTCACTGGATTTAGCTTCTATGAAGCTGTGATGTACTCATGTGATTATAACTCCTGATACTAGTAGTACGGTTGTATTTAAGAGAGGACTTCTATTGGGTTGGGGAGTAATGCCTGTGGTGGTCATAGTCCCCCTGTTTGTGGAAGTTGGGGCTAGACTAGAATGATAAAATGCTCAGAAGAAACCTGCAAGAGAATTTCTGAAATAATAAATAAATTATTCCGTATCAATAAATATTTTGGACAGGTGCGAGTATGGTGAAAACGGTATGGCACTTTCTCGTACTACATCACATCATCATTGATATTGTTATAGAACTAGCCGTCCGACCTGCAGTGAGAAGCAGTGTACAATGGTAAAGAGTGGAATCATGTAACTGCAGGCCGGATGTGAAGAAAGCTGATAGTATATATTTGGTGGTCAAAGACTTGGGTTGACTATGTGATAAGCATGGGTTTGGTGGTCGTGTGGATACATCATGTAAAGTGAAGCACTAGAGGTAAATGCTGCAGAAGCCCGGAATTAAGGCTACAACTAGAATTGATCAAGCAAAATATTAATAATGATAGTGAGTTGTGGAGGTGGAGGTAAATTGATGAAGAGTTAATGTTGTATCCCCAAGTAAATGCATTATTAATTGACCTGCTGTAATAATGGCTGTTAATCATGCAGCTAGGCTAGGTTGGTTGAATAGACTAATTTAACATGTCATTAATATGGGATTAGTGGAATTGGTGTTCCTTGTGGTAAAAAATAGGCGAGGGATGGAATTTGTTTAAATCAGAGCTATTAGTACAGTTGCTGCTCATAGAATAGCTATGCGCTAGATTTATTGACAGTTGGGTGTGGTTGGTGTAAATACATATGGTGTATAAGTCGAGAATGTTGTTTGAACAATAAAAGAAATTAGGGCTAGGAGTATAAGGGATCAGGTTCGCCCTTTAGTGGTGTGGGTTAATATTATTTGTTTAAGTGTTAGTTGAGGTGTGGTATTGTTGAATTGAAGGAAGTCGGTTGTTGAATAAGTTTTTGGAGGATAAAATTAATGTAGTGGGGGGTAATAATTAAAAATACTAAAAGTGTACTCCTAGTATTATTTGGAATATTGAATGAGGCAAATAGATTTTGGTTCATTTTAGTTCTCTAAGTTGTTTATGTTTTTGTGTTTCTACTAACATTTTGGTAGTGGGTAATAATGGAAAGTAAAGTTCAGTATTTTCAATTGTATGCATGTAAAATAAGGTAACAATTATAGATATAATTACTATTGGTCACGGTGAAATATTTAGTTGAGGCATTACTTCTAGAGGTTTGTTCTCTCAATCCTTTAACTTTCGAGGTTAATGCTAAGTTAGCTTTACAGTGATACAATATATAAGTATGACTCATACTTAGAAATCTTGAAATAAATGAATTCTAGAACAATAGGTATAAAGCTGTGATTGGACCCGCAAATTTCATGACATTGTCATAAAATAGACATAGTCGTATAGAGCTAGTATCAGCTTGGTTTTCAAACGTTAGAGTGCATCTAATACGCCTAGTGATGGTACGGCTCATGAGTATGCAAGCCATCTTCAGCTGATGAGATTAATATGCGAATATCCGCTTCTGTAAAATTAAAGTTGTTCGGTTATCAACTGCGAGGAGTCGAAATTCCCTGGCTCAAGGAAATATGTTGGCATAATGTAAAGGTCGATATAAATCTTCCCGCAGTCGGAATATTCTTCAGTTCGTATCATTGGTGGTAATTGCTTTAAAGGTTAAACAAGGTTTATTAAATTCGTCTGTCATGTATATAGAATACGCAGTGATGGGAGGGCAATTGTAATTAGAATCAATTTTAGGGGAGAATAGTTCAGATTATTTCGATCTCTTGGCGAGCATTTATGGTGCTAGTATGAGTGAGTTTTGTGGTAAGTATAGAAATAGTAGCAATATAAAGATAAGGAATACTAATCAGGAAAATAATTATGAGTGCATGGTCGTGGAAAGCAATAAGTTCTTCTATGATAGGTGATGTGGCATTTTGTAAACCTGATTGTGGTGTTATTAAGATATATAGATATTTAGTCTATAATTTAACTTTGACAAAGTTATGCTATTGATTTTACTAATATCTTATTATGAAAAGAATCATAGGGTATATGGGATTGTCTTGAAACCAATTTTGGGGTTCAATCTTCCTTCGTCTAGGATTTAACGTAAGTTGCCTCTTCGAATGTGTGGTAAGGAGGAGGGCAGCCGTATAATCATTCTAGATTGGTGGATAGTTGCTCAATGGCTAAGACTTTCGTTTTGAAGAAAGCCTCAAATTATAAAAACCATCAGGACTACTGCTGTAAGTGAAATAAATGAACCGATAGATGAGACGATATTTCATGTAGTATATGCGTCTGGATAGTCTTAATTATCGTCGAGGTATTCCGGATAAGCCAAGGAAGTGTTGTGGGAAGAAAGTTAAATTTACGCCGACAAATATAATGGTGAAGTGGATTTTGGCATATGTTTGGTCAAGAGTATAGCCTGAAAAGAGCGGGAATCAGTGAATAAATCCCCCTATAATGGCAAATACTGCTCCTATTGATAACTTATAGTGAAAGTGGGCTACTACATAGTATGTATCATGTAATACAGTATCTAATGATGAGTTAGCTAACACAATTCCTGTTGATCCACCCACGGTAAAAGAAAAATAAAGCCCAGGGCTCATAATATTGCGGAGATCATTTGATATTACCGCCCAATTGCAGTGTGGCTAATCAGCTAAATACTTTTACTCCAGTGGGAATAGCAATGATTATGGTAGCTGATGTAAAATATGCACGAGTATCTACATCTATTCCTATAACCAGAATATGTGATGGGCTCATACAATAAAGCCTAGGAAACCAATAGATATTATGGCTCATACCATGCCCATATAACTTAAAGGGTTCTTTTTATTAGAATAATATGTTACAATGTGTGAAATTATTCCAAAACCCGGTAAGATTAGAATATATACTTCAGGGTGACCGAAGAATCAGAATAAGTGTTGGTATAGGATAGGGTCGCCGCCACCAGCAGGATCAAAAGTAGTATTTAAATTACGGTCAGTTAATAGTATGGTAATCCCTGCAGCTAGAACTGGAAGAAGTAAAGAAGGACTGCAGTAATTAGGACGGATCACACAAACAGCAGGTTTGATATTGAGTCATGGCTGGCGGTTTCATATTTACAATTGTTGTAATAAAGTTAATAGCTCCAAGAATAGAAGAAATGCCTGCTAAATGTAGTGAAAAAATGGTTAAATCTACAGAGGCTCCTGGGTGGGATAAATTGCCTGCTAGGGGTGGGTAGACTGTCCAACCAGTGCCGGCACCGGCCTAAAGTTGATGATGCAAGCAGTAGGAGTAGGGAGGGTGGTAGAAGTCAGAAGCTTATGTTATTTATTCGAGGAAATGCTATATCGGAGCGCCAAATTATTAAAGGAATAAGTCAGTTCCCAAAACCCCCAATCATAATTGGTATTACTATGAAAATTATGATGAATGCGTGGGCGGTGACAATAACATTGTACATGGTCGTCTTCTATTAGGCTTCCGGGATTTGTCCTAGTTCTGTTCGAATTAGGAGGCTTGGGGCTGTACCTACTGCCCCCCGCCCATGCGCCAAATAATAAATATAATGTCCCAATGTCTTTGATTAGTTGAAAATAGTCAGCAGTTTATGAACATAGGTAGGGGTAAAATGGCTGAGCAAGCATTAGACTGTAGATCTAAAGACAGAGGAGTGAACCCTCTTTTTACCAGCCTGAGGTGAACTATCACATTGGTGCAAATTCAAGAAGCGACTCTTAATTCTGCCGGGGCTTCTCCCGCCTTTTTTCCCTAACGGCGGGAGAAGTAGATTAGCGCCAGTTGATTAGGTTATTTAGCTGTTAACTAAATTTTTGTGGTTAAAATCCCATCAATCAGGAAGGGCTTAGCTTAATTAAAAGTAATTGATTTGCGTTCAGTTGATGCAGAATAAAGTTTTGCAGTCCTTATTTGGTGTGCAGAAATTAAGTAAAATTTACTATAAGGGCTTTGAAGGCTCTTGTTAAGCATTAACCTAAATTTCTAAGTTATAAGTATTTGGTGAGTTAGGGGTAGGAGACAGGTAGAAGACACTACAAGTGGGGATAGAAACAATTATTGGCTTTATATAATTTGATTGCCAGTTGATTTTTGTGTTATTTGATGTTGGGAATATTGTCATTGAGATATAATATATTAAACGTATATAAAATATAGGCATTAATGTTAGTAAAGCTATTATAAGAATAATTAAGTTATCTATTTTTAATAAGTTAGCAATACGTAACTT

>b30aa04a-3a1a-464c-ab45-17dfe4d13bcd

TTGTACTTCGTTCAGTTACATTATTGCTATAACTAAAAAATCAACTGGCAACTAAATTATATAAAGCCAATACCGTTTCTATCCCACTTGTAGTGTCTTCTACCTGTCTCCTACCTAACTCACTAATACTTATAACTTAGAAGTAGAGTTAATAAGACCAAGAGCCTTCAAAGCCCTTAGTAAGTAAATTTTACTTAATTTCTGCACAACAAATAAGGTTTGCAAAGACTTTGTTCTGCATCAACTGAACATAAATCAGTACTTTAATTAGCTAAGCCTTCTAGATTGATGGGATTTTAACCACAAAATTTGGTTAACAGCTAAATAACCTAATCCAACTGGCTTCAATCTACTTCTCCGCCGTTAGGAAAAAGCGAGAGAAGCTTGTAAATTGAAGCTGCTTCTTTGTTGCAATTCAATGTGATAGATTCACCTCAGGGCTGGTAAAGAAGGGTTCCACTCCTCTGTCTTTAGATTTACAGTCACAATGCTTGTCCGTATTTCACCACTGTGTTCTTAAACCATGACTATTTTCAACTAATCACAAAGACATTGGGACATTATATTTATTATTTGGCGCATGGGCCAGGGCGATAGTACAGCCCTAAGCTTCCACAATTCGAACAGAACTAGGACAACCCGAGAAGCCTAATAGGGTGACCATGTATATACAATATTGTCACCGCCCACACCATTCATCTTAATTTTTTTCATAAAATATACCAGTATGATTGGGAGGTTTCGGGAACTGACTTATTCCTTTAATAATTGGCGTCCCCGATATAGCATTTCTCGAATAAATAACATAAGCTTCTGACTTCTACCACCCTCTGCTCTACTGGCATCATCAACTTTTAGAAGCCGGTGCCGGCACTAGTTGGACAGTCTACCCACCTAGCAGGCAATTTATCCCACCAGGAGCACTCTGTAGATTTAACCATTTTTTCACTACATTTGTAATTTCTTCTATTCTTGGGCTAACTTTATTACAACAATTGTAAATATGAAACCGTAGCCACGGCTCAATATCAAGCTCCATTGTTTGTGTGATCCGTCTAATTACTGCAGTCCTTCTTTTACTTTCCTTAATACGTAAC

>39021012-0a62-4a26-8721-08f761007168

GATGTACTTCGTTCAGTTACGTATTGCTTGTTCATAAACCGCTGACTATTTTCAACTAATCACAAAGACATTGGGACATTATATTTATTATTTGGCGCATGGGCGGGGGCAATGGTACGGCTGAAAACCTCCTAATTCGAACAGAACTAGGACAACCCGGAAGCCTAATAGAAGACGACCATGTATACAATGTTATTGTCACCGCCCACGCATTCATCATAATTTTTTTTCATAGTAATACCAATTATGATTGGGGGTTTTGGAACTGACTTATTCCTTTAATAATTAGCGCTCGATATAGCATTTCCTCGAATAAATAACATAAGCTTCTGACTTCTACCACCCTCCTACTCCTACTGCTTGCATCATCAACTTTAGAAGCCGGTGCCGGCACTGGTTGGACAGTCTACCCACTAGCAGGCAATTTATCACCCAGGAGCCTCTGAATTTAACCATTTTTTCACTACATTTAGCAGGCATTTCTTCTATTCTTGGAGCTATTAACTTTATTACAACAATTGTAAATATGAAACCGCCAGCCATGACTCAATATCAAGCTCCGTTGTTTGAGCGTACGTAACCA

>a689c656-0b97-4154-91b7-727e07a999cb

AGTATGCTTCCGTTCAGTTACGTATTGCTGGATCAAAAGTGTTTAAATTACGGTCAGTTAATAGTATGAAATCCCTGCAGCTAGAACTGGAAGAAAGTAAAGAGGACTGCAGTAATTAGGACGGATCACACAAACAACGGGTTGATATTGAGTCATGGCTGGCGGTTTCCATATTTACAATTGTTGTAATAAAGTTAATAGCTCCAGAATAGAAGAAATGCCTGCTAAATGTAGTGAAATGGTTAAATCTACAGAGGCTCCTGGGTGGATGGTACACCTTTGGGGTGGGTAGAACTGTCAACCAGTGCCGGCACCGGCTTCTAAAGTTGATGATGCAAGCAGTAGGAGTAAGGGGAGGGTGGTGAAAAGTCAGAAAGCTATGTTGACTATTCAGGAAATGCTATATCAGGAGGCGCCAAGTTATTAAAGGAATAAGTCAGTTCCCAAAACCCCAATCATAATTGGTATTACTATGAAAAAATTACACTGATGAATGCGTGGGCAGTGACAATAACATTGTATACATGGTCGTCTTCTATTAGGCTTCCGGGTTGTCCTAGTTCTGTTCGAATTAGGAGGCTTAGGAGCTGTACCACTGCCCCGCCCATACGCCAAATAATAAATATAATGTCCAAATGTCTTTGTGATTAGTTGAAAATAGTCGGTTTATGAACAGGTAGGGGTAAAATGGCTGAGCAAGCATTAGACTGTAAATCTAAAGACAGAGGTGAACCTCTTTTTACCAGCCCTGAGGTGAAGGTATCACATTGAATTGCAAATTCAAAAGTAGCTTCAATTCTGCAGGGCTTCTCCCGCCTTTTTTTTTCCTAACGGCGGAGAAACAGGTGGAAACCAGTTGATTAGGTTATTTAGCTGTTAACTAAATTTTTGTAGGTTAAAATCCCATCAGTCTGGAAGGAGCTTAGCTTGATAAGTAATTGATTTGCGTTCAGTTGATGCAAAATAAGTTTGCAGTCCTTATTTGTTGTGCATCAACTGAACGCAAATCATTTACTTTAGTGTGGCTTTTTTCTTTAGTTGATGAGGTTACAACCCACAAAAACGGCAGCTAAATAACAATCAACTGGCTCCGTCACCGTTAGGAAAAAAAAGGCAGGGCGGCAAAATTGAAGCCGCTGATTTACTATAGTTCACCTCGGGAACTGGTAACAAAAAGAGGGTTCTCCTCTGATGGCTGCAGGTTACGGTCAGCTATTTGCATCCTATGTTCATAAACCGCATTGTCACAAAGACATTGGAACGTTATAGTATTTGAAAAAAAAAGTGGCAAGTAACGCAACCAAGCTGTAGAGACGACCATGTATACAGTATTTATTGTCACCGCCTCTACGCATTCATCTGAATTTTTTTAAAATGTAATAATACCAATTATGAATTGGGAGTTTGGTGGCGCATTATAGGTGTAGCATATTAAACTTCGACTTCACCCCTCCTACTCCTATAGCACATCATCAACTTTAGAAGCCGGTGCAACTTCGGTTGGACAGTCTACCCACTAGCGAGCCAATTTATCCCGCAGGAGCCTCTGTAGATTACAACAAATTTTTCACTACATTTAGCAGGCATTCTATTCTTGGAGCTATTAACTTTATTACAACAATTAAATGAAACCAAAAATACATGTCTCCGTTATTTGTCCGTCAGTATAGTCTCTTTTATATATTTGATTCCCTACAACTTTAAAGTTTACCTTTATTAACGATATCAATTAAATACTCTTTCAGTGTGCTTACGTTCCAGTTACGTATTGCTCTCACCCACTGATGCTCGGCGTGGATGCGCCTCCCCGTTACCAGCAGGGCCAGAGCGACGGGTAAGATACCTATCCAGCTTAGCCATAAAGGTTGCTTCGGGGCGCAGGGCTAAACACCGGGGCGGCCTTTACTGCAGGCGAGCTGCAGCGGAGAGCAGAACGAGCAGCGTTATGTAAGGGTAAGGATGTCGGGTAAGATGTCGGACATGACGACGAAAGCCGTCAGTGCCGCCGAGGCCGTTGCCGGAGTGTCGGACGCACCCGGCACTGCATAGCGCTAAACCAAGCAACAACCCCACGCCACCTATGCCGTATCTGAGACAGAGGCGAGTAGTTCGTTGAAAACGTAAGGGACGTTGTTCGCCAGCATCGTGGCCGGATGGTTCGGGTGCTGGAAAATGTTAGCCGAGCGGGCGCGCAGTCGCGGAGGATCCCGTCGCGTCCGGCCCGTAAGCGGTGCCTCGCGCTGATGCAGTCGGCCGTGACACGGTAAATGAGCAACCTGCCGTCCAGCTGAGGCGGACGCATGCGATAGGGTGCCGCCGTCAGTGCGAGGAGAATGGTCAGACATGCCAGGCCCGCCAGCAACCTGTGCATCGGTCTTAGGCGGTGTAAGGATCCTTGTCCGTTGTAGCAGCAACCAACCCGTTGCAACCAGTTGCGACTATCCAGAGCCGTGCGGGAGAACGTCAAAGCCTCAGGCAGCCATGACGAGCAGTGTGAGTGCTGCATAGATGATGACGTAAGGATGTTATGGCATTTTGCCCGTACCTGACTCACTTTGCAGAAAAGTAATTTGTCCAGGACTGAGGCAGCAATATGACA

>38220d46-a64b-4541-8403-8b7ba1c4624e

TTTTACTTCGTTCAGTTACATTATTACTGTTATTTATTCGAGAAATGCTATATCAGGGCGCCAATTATTAAGATAAGTCAGTTCCCAAAACCCCAATCATAATTAAGTATTACTATGAAAAAAATTATGATGAATGCGTGAAACGTGTAACTAATAACATTATATACTAGTGCAAATCACCTAATGGGCTTCGGGTTGTCCTAGTTCGCATTTCAATTAGGAGGCTTGAGGCTGTACCTACTGCCCCCGCCCATACGCCAAATAATAAATATAATGTCCCAATGTCTTTGTGATTAGTTGAAAATAGTCAGCGGTTTATGAACAGCAATACGTAACTT

>07eab511-7811-4557-b048-d37d7dd6cc2b

AATACTTCGTTCGGTGCAATATTGTAGTTAGCTAACACAATTCCTGCGAATCCACCCACGGTAAAGAAAATAAAGCCCAGGGCTCATAATATTGGGAGATCATTTGATATTACCACGTGCAGTAATGGCTAATCAGCTAAATACTTTACTCCAGTGGGAATAGCAATGATTATGGTAGCTGATGTAAAATATGCAGTATCTACATCTGTTTCTACCTTGAAAGGCAGCGGTGGGCTCATACAATAAAGCCACAGAAACCAATAGATATTATAGCTCATACCATACCCACATAACGGGTTCTTTTTTATTAGAATAATATTTACATGTGTGAAATTGTTCCAAAACTTGATGAATTAGAATATACTTCAGGGTGACCGAAGAATCAGAATAGAGTGTTGGTATAGGATAGGGTCGCCTACCAGCAGGATCAAAGTAGTATTTAAATTACGGTCAGTTAATAGTATGGTAATCCCTGCAGCTAGAACTGGAAGAAAATGCGAGGACTGCAGTAATTAGGACGGATCACAAACAACGGAGTTTGATGTTGAGTCATGGCTGGCAGTTTCATATTTACAATTGTTGTAATAAGATTAATAGCTCCAAGAATAGAGAGAAATGCCTGCTAAATGTGGTAAGAAAAATGGTTAAATCTACAGAGGCTCCTGGGTGGGACAAATTGCCTGCTAGGGGTGGGTAGACTATCCAGCCAGTGCCGGCTTTCCTTTACAAAGTTGATGATGCAAGCAGTAGGAGTAGGGAGGGTGGTGGGATTAAAACATGTTATTTATTCGAAATACTATGTCAGGAGCGCCAATTATTAAAGGGGAATAAGTCGGTTCCCAAAACCCCAATCATAAGTGGTATTACTATGAAAAAATTATGATGAATGCGTGGGCAGTGACAATAACATTGTATACATGGTCGTCTTCTATTAGGCTTCCGGAGTTCTAGTTCTGTTCCGAATTAGGAGGGCTTAGGAGCTGCCTGCACGCCCATGCGCCAAATAATAAATATAATGTCCCAATGTCTTTGTGATTAGTTGAAAATAGTCAGCGGTTTATGAACGGTAGGGTAAAATGACACGACAAGCATTAGACTGTAAATCTGAGACGAGAGGGTAGGCAATACGTAAC

>8d15c22f-0f8a-4a33-b82b-4fce7d826719

TTGTACTTCCGTTCAGTTACGTATTGCTATAATGGCAAATACTGCTCCTATTGATAACATAGTGAAAGTAGACTACTACATAGTATGTATCATGTAATACAATATCTAATGATGGGTTAGCCTTACAATTCATTAGTCCACCCACGGTAAAAAGAAAAATAAAGCCAGGGCTCATAATATTGCGGGAGATCATTTGATATTACCGCCGTGCGAGTGTGGCTAATCAGCTAAATACTTTTACTCGGTGAATAGCGGTATAGTAGCTGATGTAAAATATGCGAGTATCTACATCTATTCCTACTGTGAATATGTGATGGGCTCATACAATAAAGCCTAGGAAACCAATAGATATTATGGCTCATACCATGCCCATATAACCAAGGGTTCTTTTTTATTAGAATAATATGTTACAATGTGTGAAATTATTCCAAAACCCGGTAAGATTAAAATATATGCTGGGGTGACCGAAGATCAGAATAAGTGTTGGTATAGGATAGGGTCGCCGCCACCAGCAGGATCAAAGAAAGTAGTATTTAAATTACGGTCAGTTAATAGTATGGTAATCCTGCAGCTAGAACTGAAGAGAAATAAAAGAAGGACTGCAGTAATTAGGACGGATCCACAAACAACGGAGTTTGATATTGAGTCATGGCTGGCGGTTTCATATTTACAATTGTTGTAATAAAGTTAATGACTCCAAGAATAGAAGAAATGCCTGCTAAATGTAGTGAAAAAATGGTTAAATCTACAGAGGCTCCTGGGTGGGATAAATTGCCTGCTAGGGGTGGGTGAACTGTCCATGGTGCCGGCACCGGCTTCTAAAGTTGATGATGCAAACAGTAGGAGTAGGGAGGGTGGTAGAAGTCAGAAGCTTATGTTATTTATTCGAGGAAATGTACATATCGGGAGCGCCAATTATTAAGGAATAAAGTCGGTTCCCAAAACTTCCCCAATCATAATTGGTATTACTATGAAAAAAATTATGATGAATGCGTGGGCGGTGACAATAACATTGTATACATGGTCGTCTCTATTAGGCTTCCGGGTTGTCCTAGTTCTGTTCGAATTAGGAGGCTTAGGGCTACCTGCCCCGCCCATGCGCCAAATGCTTAAATATAATGTCCCAATGTCTTTGTGATTAATTGAAAATAGTCAGCGGTTTATGAACGGAAGTGAGGGTAAAATGGCAGCAATACGTAACTTCT

>c734e203-6be3-4b97-a479-d4fbfebd7f54

AGTACTTGATTCAGTTGCAATGTTAGCAATTTTACAGCAAATAAGGACTACCAAAACACATTCTGCATCAAAAGCTGAACGCAAATCAATTACTTTAAATTAACTAAACCCTTCTAGATTGATGGGATTTTAACCACAAAAAATTTAGATTGCTTCAGCTAAATAACCTAATCAACTAAGCTTCAAATCTGCTTTTACTCGCCGTTAGGAAAAAAAGGCAGGAAGAAGCCCCAGCAAGGTAAACTGCTTCTTTCGAATTTGCAATTCAATGTGATAGTTCACCTCAGGGCTGGTAAAAAGAGGGTTCACTCCTCTGTCTTTAGATTTAAGTCTAATGCTTGCTCAGCCATTTTACCTACTATAATTTCATAAACCATGACTATTTTCAACTAATCACAAAGACATTGGGACATTATATTTACATTATTTGGCGCTGGGCGGGGCAATGGAGTACACAGCCTAAGCCTCCTAATTCGAACAGAACTGGACAACCCGGAAACCTAATAAAGACGACCATGTATACAATGTTATTGTCACCGCCCACGCATTCATCACCCATAATTTTTCATAAGTAATGCAATTATGATTGGGGGTTTTAGACGACTTATTCCTTTAATATTGGCGCTCCCGATATAGCATTTCCTCGAATAAATAACATAAGCTTCTAGCTTCACACCCTCCCTACTCCTACTGTACAATACATCAACTTTAGAAGCCGGTGCCACTAAAACAGTTGATCCTACCCACCTAGCAGGCAATTTATCCCACCCAGGAGCCTCTGTAGATTTAACCATTTTTTCACTAAATTTAACGGGCATTTTCTTCTATTCTTGGAGCTATTAACTTTATTACAACAATTGTAAATATCAAAACCGCCAACTGTGACTCAATATCAAACTCCGTTGTTTGTGTGATCCGTCCTAATTGCTGCAGTCCTTCACTTTTTACTTTCTCAGCAATACGTAACTTCT

>7ce7cbff-5500-41e6-94e7-25a6dfba8d6e

GATGTACTTCGTTCAGTTACGTATTGCTGCTCAAGGAAATATGTTGGCATAATGTAAGTCAAATACTAAGTCTTCATAGTCGGAATATTCATAGGTTCAACGTATCATTGGTGACCAATTGCTTTAAGGGTTAAATAAGGTTTATTAAATTCGTCTGTCATATATAGAATACGCAGTGATGGGAGGGCAATTTGTAATTAGAATCAGTGCAGGGAGAATAGTTCAGATTATTTCGATCTCTTGAGCATTTATGAATTGCTAGTATGAGTGAGTTTGTGGTAAGTAGCAGGGAAATAATATATAAGACTAGGAACTAATCAGGAAAATAATTATGAGTGCATGGTCGTGGAAAGCAATAAGTTCTTCTATGATTGATGTGGCATTTTGCGCGCCAACCTAGTTGAGCTGGTGTTGCTATTAGATATATAGATATTTAGTCTATATTTAACTTTGACAAAGTTATGTAATTATTTTACTATCTTATTAGAAAAGTCATGGAGTATATGGGATTGGCTTGAAACCAATTTTTGGGGTTCAAATCAACCTTTTTCGTCTAAGTTGTAACGTAAGTTGCCTCTTCGAATGTGGTAAGGAGGAGGGCAGCCGTGTAAATCATTCTAGATTGTTTGGATAGTTGCTCAATGGCTAAGACTTTTCGTTTTGAAGAGAAAGCTTCTCAAATTATAAAAACCATCAGGACTACACTGTAAGTGAAATGATGAACCGATAGATGAGACGAATTTCATGTAGTATATGCGTCTGGATAGTCTGAGTATCGTCGAGGTGATATTCCGGATAAGCAGAAGTGTTGTGGGAAGAAAGTTAAATTTTACGCCGACAAATATAATGGTGAAGTGGATTTTGGCATATGTTTGGTCAAAGTATATATACAAGCAGAATCAGTAGACAAATCCCCCTATATTAGCAAATACGCTCTATTGATAACACATAGTGAAAGTGGGCTACTACATAGTATGTATCATGTAATACAATATCTAATGATGAGTTAAGCTAACACAATTCCTGTTAGTCCACCCACGGTAAAAAGAAAAATAAAGCCCAGGGCTCATATTATTGCAGGAGATCATTTTGATATTACGCCGTGCAGTGTCGCTAATCAGCTAAATACTTTTACTCCAGTGGGAATAGCAATGATTGGTAGCTGATGTAAAAAACACACACGAGTATCTACATCTATTCACACTACTGTGAGATGTGATGAAGCAATGATAAAGCACGAAACCAATAGATGTATGGCTCATACCATGCCCATATAACCCAAGGGTTCTTTTTTATTAAGAGATAATATGGCTACAATATATGGCGGTATTCTAAAACCCGGTAAGATTAGAGTTATTATATGGGGTAGCCGAAGAATCAGAATAAGTGTTGGTATAGGATAGGGTCTGCCACCAGCGGATAAAGTAAGTGTTGATTTACGGTCAGTTAATGGTATGGTAATCCACAGCGCAAACTGGAAGAAAGTAAAGAAGGACTGCGGTAATTAGGACGGATCCCACAAACAACAGGGTTTGATATTGGTGTAGCTGGCGGTTTTCATATTACAATTGTTGTGTAAAGTTAATAGCTCCAAGAATAGAAAGTACCTGCTAAATGTATTGAAAAGTGGTTAAATCTACTGAAGCTCCTGGGTGGGGATAAATTACTGGTGGGTGAGCTGTCCAGCAGTGCCGGCACCGGCTTCTAAAGTTGATGATGCAAGCAGTAGGAGTAGGGAGGGTGGTAGAAGTCAGAAGCTTATGTTTATTTATTCGAGGAAATGCTATATCAGGGCACTTAATTATTAAAAGAATAAGTCGATTCCCGAAACTTCCAATCTATAATTTGGTATTACTATGAAAAAAATTATGATGAATGCGTGGGGCGGTGACAATAACATTGTATATGGTCTCGTCTTCTATTAGGCTTCCGGGTTGTCCTAGTCTGTTCGAAGATTAGGAGGCTTGGGGCTGTACCTACTGCCCCCGCCCATGCCAGCAATAAATATATTAATGTCATGATTAGTTGAAAATGATCGGTTTATGAATTTTAAGTGAAATTAAAAATGGCTGAACAAGCATTAGACTGTAAATCACAAGAACAGGATGAACCCTCTTGCAATATAGAGTGGACTATCACATTGAATTGCTGGAAATTCAAGACGCTTCAATTCTGCCGGGGCTTCTCCCGCCTTTTTTTTTCCTAACGGCAAGGGAGGAAGTAGATTAAAGCAGTTGATTAAGAGTTATTTAACATTATTAATTTTTGTAGAGTTTTAAATCCATCAATATCTAGGAAGGGCTTAGCTTTAATTAGTAATTTGATTTTGCGTTCAGTTGATACTGCAGAATAAAGTTTTTGCAGTCTTATTTGTTATGCAGAAATTACAAAATTTGCATAAGGGCTTTAGAAGGCTCTTGGTCTTATTAACCTAAATTTCTAAGTTATGAAATATTAGTGGAGTTAGGGTAGGAGACAGGTAGAACGCTTTTCAAAGTGTAGGGATCAAAGCAGTATGGCTTTATATAATTTAGTTGCCAGTTGATTTTATGTTATTTGATGGTGGGAATATTGTCATTGGGATATATAATATAATAAGCAATATAAAATATATAAATTTATTAATGTTAGTAAAGCTATTATAAGGGGAATAATTAAGTTATCATTTTTAATAAGTTCTTGTATAATAAGCATTTAGGGGAAAAACCTGTTAGTGGTGGGCCTCTAGGGATATTATTGACTAGTGGAATAATGGGTATTGTTCATGCTAGTTTGTTTTCAGGTATGTTATGATAGGGATAGGGTTAATGTTAGGGGTTAAATAAAAGATTATTAATGTGTAGAAGTTGTTGGGAAAATATGAAATAATTAAAGTTAATATAGTAATATTAGGGTCGTAATAATACTGCTTTATTATTCATCTATAGCGAATGTAATTGGGGCAGAGTAGGTTTGCGGGTATGTTTTGGTTGAGTCCTCACTACATACCAGTTATGGTGATAGAACTATGATTGTTAGAATCAAGTTTAGGTTTGTTGACGGAAAAATTTGGAGGAGAATTGATATTAGGGCTTATTTTTGTCACGTGAGGCAATTATAGCTAATTAGAGTTCGCATGGGTAATTTCACGGTCAAGTAGTGGGGCTATCTATTTTGTATTATGGACGTAAATATTATTATGGATAATGTTTGGTTATAAAGGAGGATTGATTGTTCATTGTTGATTAGTGAATTATTAAGGAAAATGGATACTATTAGAAGTATTGATGCTGTTGCTTGAATTAAAAAATATTTGGTGGATGCCTCTGTGGGTGGGATTGGTACTTTTGTGAAGTTGGTACGATGGCTAGTATATTTAATTCTAAATATTCAGATGGGAGTCCAATGTGACTTAAAATTATGATTGATCCCTGTTAGAATGGTGAGAGGAGATAATAAGAAGGGTGGGTAGAGGATTAATGTTAGCACGGGAAGATTCGACCAACGCATTTTCGAAATTGTGGGCCCGATGGCTTATTAGCTAGCCCTTACTGTTTATGAGATGTGTTGTAATCGGTAGCACGGAAGTTTTGAGTTCTCGGGTGTAGGTTCAATTCCTGTAATCACAAAATAAGGGTTTTGCGTTCTCTATAATTTACTCTATCAAAGTAACTCTTTTTTTTATCAGGAACATATTTCTTATGTTTGGGTGGGATGTAGATACTAGGATGGTGGCCCAAACATATCATATACACTTGTTGTAATGTAGAGTGGTAAAGGTTTTCATAGTAGCAATACGTAA

>a2194b39-fd96-43a0-988d-b66001c6721d

AATGTACTTCGTTCAGTTACGTATTGCTGGTAATCCCTGCGGCTAGAACTGGAAAAGAGAAACAAAAGAAGGACTGCGATAATTAGGACGGATCACACAAACAACGGAGTTGTTGAGTCATGGCTGGCGGTTTCATATTTACAATTATTTGTAATAAAGTTAATAGCTCAAGAATAGAAGAAATGCCTGCTAAATGTAGTGAAAAAATGGTTAAATCTACAGAGGCTCCTGGGTGGGACAATTGCCTGCTAGGGGTGGGTAGACTGTCCAACCAGTGCCGGCACCGGCTTCTAAAGTTGATGATGCAAGCAGTAGGAGTAGGGAGGGTGGTAGAAGTCAGAAGCTTATGTTATTTATTCGAGGAAATGCTATATCGGGGGCGCCAATTATTATTAAAGGGAATAAGTCAGTTCCAAAACCCCAATCATAGTGGTATTACTATGAAAAAATTATGATGAATGCGTGGGCAGTGACAATAACATTGTATACATGGTCGTCTTCTATTAGGCTCCGGGTTGTCCTAGTTCTGTTCGAATTAGGAGGCTTGGGGCTGTACCTACTGCCCCGCCCATGCGCCAAATAATAAATATAATGTCCCAATGTCTTTGTGATTAGTTGAAAATAGTCAGCGGTTTATGAAAAAGACATAGGTAGGGTAAAATGGCTGAGCAAACATTAGACTGTAAATCTATAAAGACAGAGTGAACCCTCTTTTACCAGCCCTGAGGTGAACGCTATCACATTGAATTGCAAATTCAAAGAAAGCAGCTTCAATTCTGCCGGGGCTTCTCCCGCCTTTTTTCCTAACGGCGGGAGAAGTAGATTCGAAAGCCAGTTGATTAGGTTATTTAATATTAACTAAATTTTTGTGGGTTAAATCCCATCAATCTAGGAAGAGACTTAAGCAATTAAAGTAATTGATTTGCGTTCAGTTTGATGCAGAATAAAGTTTTGCAGTCCTTATTTGTTGTGCAGAAATTAAGTAAAATTTACTTACTAAGGGCTTTGAAGGCTCTTGGTCTTATTAACCTAAATTTCTAAGTTATAAGTATTATTGGTGGAGTTAGGAGTAGGAGACAGGTAGAAAGCAATACGTAACTA

>5f19582a-5961-47f5-a216-724dd6df17e5

AATGTGTAACCCCGTTCCATTTACGTATTGCTCATAAACCGCTGACTATTTTCAACTAATCACAAAGACATTGGGACATTATATTTGTTATTTGGCGCATGGGCAGGGCGGTAAATTGCAGCCTAAGCCTCTAAGTTCGAACAGAACTAGGACAACCCGGAAGCCTAATAGAAGACGTCCATGTATACAATGTTATTGTCACCGCCCGCATTCATCCTTAATTTTTTTTCATAGTAATACTAATTATGATTGGGGGTTTGAAACAAGCATTCGCTTTAATAATTAGCACTCCCGATATGGCATTTCCTCGAATGATAACGCAAGCTTCCTGACTTCTTACCTCTACTCCTACTGCTTGCATCATCAACTTTGAAATAGTGCAGCACTGGTTGGACAGTCTACCCACCCTAGCAGGCAATTTATCCCACCCCGGGAGCCTCTATGGATTTAGCCATTTTTTCACTACATTTTAGCAGGCATTTTCTTCTATTCTTGGGGCTATTAACTTTATTACAACAATTGTAAATGTAAAACCGCCAACCGGCGACTCAATATCAAACTCCGTTGTTTGTGTGATCCAATCCTAATTACTGCAGTCCTTCTTTTACTTTCTCTTCCAGTTCTAGCCACGGGGATTACCATACTATTAACTGACCGTAATTTAAATACTACTCTTTGATCCTGCTGGTGGCGGCGACCTAGCAATGCGTAA

>6d274715-e1fc-4902-9fea-971ca8326e73

GTTGTATTACTTCGTTCAGTTACGTATTGCTCATAAACCGCTGACTATTTTCAACTAATCACAAAGACATTGGGACATTATATTTATTATTTGGCGCATGGGCGGGGGCAGTAGGTACAGCCCTAAGCCTCCTAATTCGAACAGAACTAGGACAACCCGGAAGCCTAATAGAGAATGACCATGTATACAATGTTATTGTCACCGCCCGCATTCATCATAATTTTTCATAGTAATACCAATTATGATTGGGGGTTTAGGAACTGACTTATTCCTTTAATAATTGGCGCTCCCGATATAGCATTTCCTCGAATAAATAACATAAGCTTCTGACTTCTACCACCCTCCCTACTCCTACTGCTTGCATCATCAACTTTAGAAGCGGGGTACAGCACTGGTTGGACAATCTACCCACCCTAGCAGGCAATTTATCCCACACCCAGAGGCCTCTGTAGATTTAACGGTACTGCTACATTTAGCAGGCATTTCTTCTATTCTTGGAGCTATTAACTTTATTACAACAATTGTAAATATGAAACCGCCAGCCATGACTCAATATCAAGCCATTATTTGTGTGATCCGTCTCATTACTGCAGTCCTTCTTTACTTTCTCTTCCAGTTCTAGCTGCAGGGGATTACCATGCTATTAACTGACCGTAATTTAAATACTACTTTCTTTGATCCTGCTGGTGGCAGCGACCCTATCTATACCAACACTTATTCTGATTCTTCGGTCACCCTGAAGTATATATTCTAATCTTACCGGGTTTTGGAATAATTTCACACGACATAACATATTATTCTAATAAAAAGAACCCTTTGGTTATGGGCATGGTATGAGCCATAATATCTATTGGTTTCCTAGGCTTTATTGTATGAGCCCATCACATATTCACAGTAGGAATAGATGTAGATACTCGTGCATATTTTACATCAGCTACCATAATCATTGCTGTTCCCACTGGAGTAAAAGTATTTAGCTGATTAGCCACACTGCACGGCGGTAATATCAAATGATCTCCCGCAATATTATGAGCCCTGGGCTTTATTTTTCTTTTTACCGTGGGTGGACTAACAGGAATTGTGTTAGCTAACTCATCATTAGATATTGTATTACATGATACATACTATGTAGTAGCCCACTTTCACTATGTGTTATCAATGGTGTGTTGCCATTATAGGGGGATTTATTCACACAGTTCCCCGCTCTTTTCAGGCTATACTCTTGACCAAACATATGCCAAAATCCACTTCTCATTATATTTGTCGGCGTAAATTTAACTTTCTTCCCTAACACTTCCTTGGCTTATCCGGAATACCTCGACGATACTCAGACTATCCAGACGCATCTCTTCTGTAAATATCGTCTCATCTATCAGTTCATTTATTTCACTTACAGCAGTAGTCCTGATGGTTTATAATTTGAAGCTTTCTCTCGCCCCCGAAACGAAAGAGTCTTAGCCATTGAGCAACTATCCACCAATCTAGAATGATTATACGGCTGCCCTCCTCCTTACCACATTCAGAAGGCAACTTACGTTAAATCCTAGACGAAAAAGGAAGGATTTGAACCCCAAAATTGGTTTCAAGCCAATCCCATATACCCTATGACTTTTTCAATAAGATATTAGTAAAATAATTACATAACTTTGTCAAAGTTAAATTATAGACTAAATATCTATATATCTTAATAGCAACACCAGCTCAACTAGGTTTACAAAATGCCACATCACCTATCATAGAAGAACTTATTGCTTTCCACGACCATGCACTCATAATTATTTTCCTGATTAGTTCCTTAGTCTTATATATTATTTCCCTTATACTTACCACAAAACTCACTCATACTAGCACCATAAATGCTCAAGAGATCGAAATAATCTGAACTATTCG

>2286a8b1-b338-4506-a9d0-5c655539d590

AGTATTACTTCGTTCAGTTATGTATTGCTAAATCAATTACTTTAATTAAGCTAAGCCCTTCCTAGATTGATGGGTTTTAACCCACAAAATTTAGTTAACAGCCAAATAACCTAATCAACTGGCTTCAATCTACTTCTCCCGCCGTTAGGGAAAAAAGGCGGGAGAAGCCCCGGCAGAATTGAAGCTGCTTCTTTAATTTGCAATTCAATGTGATAGTTCACCTCAGGGCTGGTAAAAGAGGGTTCACTCCTCTGTCTTTAGATTTACAGTCTAATGCTTGCTCAGCCATTTACCCCTACCTATCTCATAAACCGCTGACTATTTTCAACTAATCACAAAGACATTGGGACATTATATTTATTATTTGGCGCATGGGCGGGGGCAGTAGGTACAGCCCTAAGCCTCCTAATTCGAACAGAACTAGGACAACCGGAAGCCTAATAGAAGACGACCATGTATACAATGTTATTGTCACCGCCCACGCATTCATCATAATTTTTTTCATAGTAATACCAATTATGATTGGGGTTTTGGGAACTGACTTATTCCTTTAATAATTGGCGCTCCCGATATAGCATTTCCTCGAATAAATAAGCAATACGTAACCAA

>c6a36746-5635-4c7b-a956-403ef9f15c25

ATACTTCGTTCAGTTGCGTATTGCTAATTTTGGGGTTCAAATCCTTCCTTTTCATCTTAGGTTAACGTAAGTTACTCTTCGAATGTGTGGTAAGAAGGAGGAGGGCGGCCGTATAATCATTCTAAGAATTGGTGGATAGTTGCTCCATTATTAAGACTTTTCGTTTTGAAGAAAGCTATAAATTATAAAAACCATCAGGACTACTGCTGTAAGTGAAATAAATAGAACCGATAGATGAGACAATCTTCATGTAGTATATGCGTCTGGATAGTCTGAAGTATCCATCGAGGTATTCCGGATAACCAAGGAAGTGTTGTGGGAAAAAGATTAAGTTACGCCGACAAATATATTAGAACATTGGATTTTTGGCATATGTTTGGTCAAGAGTATAGCCTGAAAAGGCAAGAATCGAGTGAAATGAATAATCCCTATAATGGCAAATACTGCTCCTATTGATAACACATAGTGAAAGTGGAGCTACTACATAGTATGTATCATGTAATACAATATCTAATGATGAGTTAGCTAACACAATTCCTGTTAGTCCACCCACGGTAAAGAAAAATAAAGCCCAGGAAGCCATAATATTGCAGGAGATCATTTGATATTACCGCCGTGCAGTGTGGCTAATCAGCTAAATACTTTTACTCAGTGGGAATAGCAATGATTATGGTAGCTGATGTAAAATATGCACAGTATCTACATCTATTCCTACTGTGAATATGTGATGAGCTCATATCCAAAGCCTAGGAAACCAATAGATATTATGGCTCACCATGCCCTTATAACCAAGGGTTCTTTTATTAAAGAATAATATGTTACAATGTGTGGTATTCAAAACCCGGTAAGATTAGAATATATACTTCAGGGTGACGAAGAATCAGAATAGAAGTGTTGGTATAGGATAGGGTCGCCGCCACCAGCAGGATCAAGAGAAAGTAATGACTAAATTACGGTCAGTTAATAGTATGGTAATCCTTGCAGCTAGAACTGGAAGAGAAAGTAAAGAAGGACTGCAGTAATTGGACGGATCACACAAACAACTGAGTTTGATATTGAGTCATGGCTGGCGGTTTCATATTTACAATTGTTGTAATAAAGTTAATAGCTCAAGAATGAAGAAATGCTAAATGTAGTGAAAAAATGGTTAAATCTACAGAGGCTCCTGGGTGGGATAAATTTACCTGCTAGGGGTGGGTAGACTGTCCAACCGAATGCGGCACCGGCTAAAGTTGATGATGCAAGCAGTAGGGAGTAGGGAGGGTGATGAAGTCAGAAGCTTATGTTATTCAAATGCTATATCAGGAGCGCCAATTATTAAAGGAATAAGTCAGTTCCCAGAAAACCCCAATCCTTAATTGGTATTACTATGAAAAAATTATGATGAATGCGTGGGCGGTGACAATAACGGTATACATGGTCGTCTTCTATTAGGCGGGTTGTCCTAGTTCTGTTCGAATTAGGAGGCTTAGGCTGTACCTACTGCCCCCGCCCGTACGCCAAATAATAATATGTCCCAATATTTGTGATTAGTTGAAAATAGTCGGTTTATGAGTACATAGGCCAAGGAGTAAAATGGCTGAGCAAGCATTAGACTGTAAATCTAAAGACAGAGGGTGAACCCTCTTTTATACAGCCCTGAGGTGAACTATCACATTGAATTGCAAATTCAAGCAGCTTCAATTCTCCGGGGCTTCTCCCGCCTTTTTCCCTAACGGCGGAGAAGTAGATTGAAGCCAGTTGATTAGGTTATTTAGCTGTTAACTAAATTTTTGTGGGTTAAAATCCCATCAATCTAGGAAGACTTAACCAATTAAAGTAATTGATTTGCGTTCAGTTGATGCAGAATAAAGTTTTGCAGTCCTTATTTGTTGTGCAGAAATTAAGTAAAATTTACTTACTAAGGGCTTTGAAAGGAAGGCTCTTGGTCTTATTAACCTAAATTTCTAAGTTATAAGTATTAGTGGAGTTAGGGGTAGGAGACAGGTAGAAGACACTACAAAGTGGGGATAGAAGCATTATTGGCTTTATATAATTTAGTTGCCAGTTGATTTTTTGTGTTATTTGATGTTGGGAATATTGTCATTGAGATATAATATATTAAACGTATATAAAAATATAAATTTATTAATGTTAGTAAAGCTATTATAAGGGGAATAATTAAGTTATCATTTTTATCTTAATGTCTGTAA

>143a1a64-0c50-49d4-9bb6-eb3c8aaa3fc8

GATATACTTCGGTCAGTTACGTATTACACTTTAGAACTGGAAGAAAGTAAAAGAGGACTGCAGTAATTAGGACGGATCACACAAACAGCGGAGTTTGATATTGAGTCATGGCTGGCGGTTTCATATTTACAATTGTTGTAATGAAGTTAATAGCTCCAAGAATAGAAGAAATGCCTGCTAAATGTAGTGAAAAAATGGTTAAATCTACAGAGGCTCCTGGGTGGGATAAATTGCCTGTAGGGGTGGGTAGACTGTCAACCAGTGCCGGCACCGGCTTCTAAAGTTGATGATGCAAGCGGTAGGAGTGGGGAGGGTGGTAGAAGTCAAGCTTATGTTATTTATTCGAGAAATAACATATCGGGAGCGCCAATTATTAAAGGAATAAGTCAGTTCCCAAAAACCCCAATCATAATTGGTATTACTATGAAAAAATTATGATGAATGCGTGAGCAGTGACAATAACAATATGTATACATGGTCGTCTTCTATTAGGCTTCCGGGTTGTCTAGTTCTGTTCAATTAGGAGGCTTAGGGCTGTACCTACTGCCCCCGCCCATGCGCCAAATAATAAATATAATGTCCCAATGTCTTTGTGATTAGTTGAAAATAGTCGGTTTATGTAGACATAGTGAGGAGTAAAATGCTTCGAGCAAGCATAGCAATACATGCA

>c2011926-9640-494b-8c3f-819ba9808fb0

AAATCTTCGTTCCGTTACGTATTGCTTTTAGATTTACAGTCTAATGCTTGCTCAGCCATTTTACCCTACCTATCATTATAAACCGCTAGCTATTTTCAACTAATCACAAAGACATTGGGACATTATATTTATTATTTGGCGCATGGGCGAGGGCAGTAATTACCAGCCTAAGCCTCTAATTCGAACAGAACTGGGACGGCGGAAGCTATTAGAGAATGACCATGTATACAATGTTATTGTCACATACGCATTCACTCTTAATTTTTCCTTGTGTACCAATTATGATTGGGGGTTTGGGAACTGACTTATTCCTTCAATAATTGGCGCTCCCGATATAGCATTTCCTCGAATAAATAACATAAACTTTGACTTCACCATACCACCCTCCCTACCTCCTACGCTTGCATCATCAACTTTAGAAGCCGGTGCCGGCACTGGTTGAGACAGTCCCTGCCATACCCACCCCTAGCAGGCAATTTATCCCGCAGGAGCCTCTGTAGATTTAGCCATTTTTTCACTACATTTGAAAGAACATTATATTCTTGGAGCTATTAACTTTCATGCAACAATTGTAAATATGAAACCGCCAATATGACTCAATAATCAAACTCCGTTGTTTGTGATCCCGTCCTAAGTACTGCAGTCCTTCTTTTACTTTCTCTTCCAGTTCTAGCTGCAGGGATTACCATAATAACTGACCGTAATTTAAATACTACTTTCTTTGATGCTGGTGGCGACTATCCTATACCAACACTTATTCTGATTCTTCGGTCACCCTGGAAAGTATATATTCTAATCTACCGGGTTTGGAATAATTTCACACATTGTAACATATTATTCTAATAAAAAAGAACCCTTTTAGTTTATATGGGCATGGTATGAGCCATAATATCTATTGGTTTCCTAGGCTTTATTATGAGCCCATCACATATTCACAGTAGGAATAGATGTAGATACTCGTGCATATTTTACATCAGCTACCATAATCATTGCTATTCCCATGAATAAAGTATTTAGCTGATTAGCCACTGCACGGCGGTAATATCAAATGATCTCCCGCAATAATACAGACCTGAGGCTTTAAGCAATACGTAACTTG

>44c0f3d2-4f71-4e43-9f5d-f66d76130b38

GATGTACTTCGTTCCGTATTGCTAATTGCCTGCTAGGGGTGGGTAGACTGTCCAGCCAGTGCGGCACCGGCTTCTAAAGGAAGTTGATGCAAGCAGTAGGAGTAGGAGGGTGGTAGAAGTCAGAAGCTTATGTTATTTATTCGAGGAAATGCTATATCGGGAGCGCCAATTATTAAAGGAATAAGTCAGTTCCCAAAACCCCCAATTGGTATTACTATGAAAAAAATTATGATGAATGCGTAGACAGTGACAATAACATTGTATACATGGTCGTCTTCATATTAGGCTTCGGGTTTGTCCTAGTTCTGTTCGAATTAGGAGGCTTAGGGCTGTCTACTGCCCCACTTCATGCGCCAAATAATAAATATAATGTCCCAATGTCTTTGTGATTAGTTGAAAATAGTCAGCGGTTTATAGACTTGCCATTTAGGGGTAAAATGGCTAGGCAAGCGTGGACTGTAAATCTAAAGACAGAGGAATTGAACCCTCTTTTTACCAGCCTGAGGTGAACTATCACGTG

>f3aa59fb-066d-4866-836a-1ae566f11013

GACATGCTTCCGTTCAGTTGCGTATTACTGCTGGCGGTTTCATATTTACAATTGTTGTAATAAAGTTAATAGCTCAAGAATGAAAGAAATGCCTGCTAAATGTGGTGAAAAAATGGTTAAATCTACAGAGGCTCCTGGAGTGGGATAAATTGCCTGCTAGGGGTGGGTAGACTGTCCAGCCAGTGCCGGCTTCCGACTAAAGTTGATGATGCAAGCAGTAGGAGTAGGGAGGGTGGTAGAAAGTCAGAAAGCTTATGTTATTTATTCGAAATGCATATCAGGAGCGCAATTATTAAGGAATAAGTCGGATTTAAAACCCCAATCATAATTGGTATTACTATGAAAAAAGTATGATGAATGCATTAGGCAGTGACAATAACATTAATATACTATGGTCGTCTTCTATTGAGCTTCCGGGTTGTCTGGTCTGTTCAATTAGGAGGCTTAGGGCTGGCACCCTGCTACCCCCGCCCATGCATGAAATAATAAATATAATGTCCCAATGTCTTTGTGATTGATTTGAAAATAGTCAGCGAGTTTATAAAGACACATAGGTAAGTAAAATGGCTGAGCAAGCATTAAGGCTGTAAATCTAAAACGAAAGTGAACCCTCTTTTTACCAGCCTGAGGTGAACTGCTCCGCATTGAATTGCAAATTCAAAGAAGCAGCTTCAATTAACCAGGGCTTCTCCGCCTTTTTTCAACGAGCAATACGTAG

>eb5a9f88-bfa6-4e2e-aa16-1ac0d0a613f8

GATGTACTTCGTTCAGTTACGTATTGCTCCGCAAATTTCGAAACGTGTCCATAAAATAGGCCTGGTCGTATAGAGGCTAGTATGGCTTGGTTTAAGCGTCCAGGAATTGCATCTGTTTTACGCCTAGTGATGGTACGGCTCATGAGTGTAAGACGTCTTGTGATGAGATTAATATGCGAATATCCGCTTCCATATAGGTAAAAAAATTGTTCGGTTATCAACTTCGAGAGTCGAAATTCCCCTGGCTCAAGGAAATATGTTGGCATAATGTAGAGTCAAATACTAAGTCTTCATAGTCGGAATATTCATAGGTTCAGTATCATTGGTGACCAATTGCTTTAAGGGTTAAATAAGGTTTATTAAATTCGTCTGTCATATATAGAATACGCAGTGATGGGAGGGCTTATTGTAATTAGAATCAGTGCAGGGAGGAATAGTTCGAATTATTTCAAGTCTCTTGAGCATTTATGGTGCTAGTATGAGTGAGTTTTTAATGGTAAGTATAAGGAAATAATATATAAGACTAAGGAACTAATCAGGAAAATAAGTATGAGTGCATGGTCGTGGAAAGCAATAAGTTCTTCATGATAGGTGATGTGGCATTTTGTAAACCTAGTTGAGCTGGTGTTGCTATTAAGATATATAGATATTTAGTCTATAATTTAACTTTGACAAAGTTATGTAATTATTTTACTAATATCTTATTGAAAAAGTCATAGGGTATGGGATTGGCTTGAAACCAATTTTTGGGGTTCAAATCCTTCCTTTTTCGTCTAGGATTTAACGTAAGTTGCCTCTTCGAATGTGGTAAGGAGGAGGGCAGCCGTATAATCATTCTAGATTGGTGGATAGTTGCTCAATGTAGGCTAAGACTTTTCGTTTTGAAGAGAAAAGCTTCTCAAATTATAAAAACCATCAGGACTACTGCTGTAAGTGAAATAAATGAACCGATGATGAGACGATATTTCATGTAGTATATGCGTCTGGATAGTCTGAGTATCGTCGAGGTATTCCGGATAAGCCAAGGAAGTGTTGTGGGAAGAAAGTTAAATTTTGCGACAAATATAATGGTGAAGTGGATTTTGGCATATGTTTGGTCAAGAGTATAGCCTGAAAAGAGCGGGAATCAGTGAATAAATCCCCCTATAATGGCAAATACTGCTCCTATTGATAACACATAGTGAAAGTGGGCTACTACATAGTATGTATCATGTAATACAATATCTGATGAGTTAGCTAACACAATTCCTGTTAGTCCACCCACGGTAAAAAGAAAAATAAAGCCCAGGGCTCATAATATTGCAGGAGATCATTTGATATTACCGCCGTGCAGTGTGGCTAATCAGCTAAATACTTTTACTCCAGTGGGAATAGCAATGATTATGGTAGCTGATGTAAAATATGCACGAGTATCCACATCTATTCCTACTGTGAATATGTGATGGGCTCATACAATAAAGCCTAGGAAACCAATAGATATTATGGCTCATACCATGCCCATATATTAACCAAAGGGGTTCTTTTATTAGAATAATGTTTACAATGTGTGAAATTATTCCAAAACCCGGTAAGATTAGAATATATACTTCAGGGTGACCGAAGAATCAGAATAAGTGTTGGTATAGGATAGGTCGCCGCCACCAGCAGGATCAAAAGAAAGTAGTAATTAAAGTGCGGTCCGGTTGTGAGTATGGTAATCCTGCAGCTAGAACTGGAAGAAAGTAAAAGAAGGACTGCAGTAATTAGGACGGATCACACAAGTAACGGAGTTTGATACGAGTCATGGCTGGCGGTTTCATATTTACAATTGTTGTAATAAAGTTAATAGCTCCAGAGAATAGAAGAAATGCCTGCTAAATGTATTTGAAAAAAATGGTTAAATCTACAGAGGCTCCTGGGTGGGATAAATTGCCTGCTAGGGTGGGTAGACTGTCCAACCAGTGCCGACACCGGCTTCTAAAGTTGATGATGCAAGCAGTAGGGTGAGGAATTGGTAGAAGTCGAGAAGCTTTATGTTATTCAGGAAATGCTATATCGGGAGCGCCAATTATTAAAGGAATAAGTCAGTTCCCAAAACCCCCAATCATAATTGGTATTACTGAAAAAAGAATTATGATGAATGCGTGAGCAGTGACAATAACATTGTATACATGGTCGTCTTCTATTAGGCTTCCGGGTTGTCCTAGTTCTGTTCGAATTAGGAGGCTTAGGGCTGTACCTACTGCCCCCGCCCATGCAACCAAATAATAAATATAATGTCCCAATGTCTTTGTGATTAGTTGAAAATGGTCAGCGGTTTATAGACATAGGTAGGGGTAAAATGGCTGAGCAAGCATTAGACTGTAAATCTAAAGACAGAGGAGTGAACCCTCTTTTACCAGCCCTGAGGTGAACTATCACATTGAATTACAAATTCAAAGAAGCAGCTTCAATTCTGCCGGGGCTTCTCCCGCCTTTTTTCCTAACATCGGGAGAGTAGTTAAAGCAGTTGATTAGGTTATTTAGCTGTTAACTAAATTTTTGTGGGTTAAATCCCATCAATCTAGGAAGGGCTTAGCTTAATTAAAGTAATTGATTTGCGTTCAGTTGATGCAGAATAAAGTTTTGCAGTCCTTATTTGTTGTGCAGAAATTAAGTAAATTTACTTACTAAGGGCTTTGAAGGCTCTTGGTCTTATTAACCTAAATTTCTAAGTTATAAGTATTAGTGGAGTTAGGGTAGCAATACGTAG

>ac6638c4-defd-4dc8-bb34-21cb2419fe18

GATATTACTTCGTTCAGTTTACGTATTACTATAGATATTTAGTCTATAATTTAACTTTGACAAAGTTGTGTAATTATTTACTAGTATCGCAATGAAAAAGTCTTAGGGTATATGGGATTATTTGAAACAAGTTTTGAGTTAAAGTCTACCTTTTTCGATACAGGATTTAACGTAAGTTGCCTCTTCAAATAGCTGGTAAGGAGGAGGTGTATTATAATCATTCTAGATTGGTGGATAGTTGCTCAATGGCCGGAGCATGGTTTTGAGGCTTTTCTCAATTATAAAACCATCAGAACTCATATATGCGAGTAAAATAAATGAACCGATAGATGAGACGATGTTCCATGTGGTATGTAGATGGATGGTCGGTGATAGTCTAGAATTATTCCGGATAAACCAAGGAAGTGTTGTGGGAAGAAAGTTGAGTTTGCGCCGACAAATATAATGGTGAAGTGGATTTTAGCAGCATATGTTTGATTGGTATGGCCTCGAAGGCGAAGAATCGAAAGTTGAATCGTCCTATAATGGCAAATACTGCTCCTATTGATAACACATTGGTGGAGGTGAACTTTTTACGTAAGTATGTATCATGTAATACAATATCTATTGATGAGTTAGCTAATACAATTCCTGTTAGTCACTGCAATAAAAGAAAAATAAAGCCAAGGACTCATGAAAATATTACAGAGTCATTTGATATTACCGCCTAGGTGCAGTCTAATGGCTAATCAACTAAATACTTTTACTCCCGGTAAATATATCAGTCTCATACGATGTAAAATACTTTTTAGTATCATCATTCTCATACTGTTGAACTTTAATAAAAAAACCATGATGGATGTACAATAAAAGCCTAGGGAAACCAATGAATGTTATAACCATCTCGCCATATATTAGAGGTTCTTTTTTATTAGAATAATATGTTACAATGTGTGAAATTATTCCAAAACCAGTAAGATTAGAATATATGCTATAAGGTGGACCGAGAATCAGAATAAGTGTTGGTACGCAAGTTGGTCAGCGCTCCTAGCAGAGGTCAAAAAGAAAGTAGTACGTCGGTCAGTTAATAGTATGGTAATCCTGCAGCTAGAACTGGAAGAGAAAGTAAAAGAAGGACTGCAGTAGTCAGACAATGGATCCACACAAACAGCGGGTTTATTATTGGAGTCATGGCTGGCGGTTTCGCATTTTTGCAGTTGTTATGCTCAAAGTTAATAGCTCAGAATGAAGAAATACTGCTAAATGTAGTGGCAATGGTTAAATCTGCAGAGGGCTCGCAGGTGGGATAAATTTGCCTGCTAGGGTGGGTAGACTGTCCAACCGGTGCCAGCACCGGCTTCTAAAGTTGATGATGCAAGCAGTAGGGTAGGGAGGGTGGTAGAAGTCAGAAGCTTATGTTGTTTGTTCGGGGAAATGCTATCAGGGCGCCAATTTATTGGGAATAAGTCAGTTCCCAAAGCTAATCATAATTGTTATTTACTATGAAAAGAAAATTATGATGAATGCGTGGGCGATACAATAACATTGTATACATGGTCGTCTTCTGTTAGGCTTCCGGGTTGTCCGGTCAATTTCGAATTAGGGAATAGGGTGTACCTACTGCCCCGCCCATGCATAAATAATAAATGTATGTCAATATTGTTTTGTGATTAATTGAAAAATAGTCTGTAGTTTATAGACATGGGTAGGGAGTAAGTATTTGAGCAAGCGTTAGACTAATATCTAAAGACAGAGAGTGAACCTCTTTTTTTACAACCCACAAGGTGAACTATCCATTGGATTGCAAATTCAAAAAAACAACTTTCAATTCTGCCGGGACTGCTCCACCTTTTTTCTAACGTAGGTAGATTGAGCCAATTGATTAGGTTATTTAGCTGTTAACTAAATTTTTGTAGGTTGAAGTCTATCTGTCTAGGAAGGGCTTGGCAATTAATAGTGGTTACATTAATTGTGCTGTACGTAACTTTA

>563c680a-2c94-4395-840e-d981eb1c2a7f

ACTTCGTTCAGTTACGTATTACTTAAACCATGGCTATTTTCAACTAATCACAAAGACATTGGGACATTATATTTATTATTTGGCGCATGGGCGGGGGCAGTAGGTACAGCCCTAAGCCTCCTAATTCGAACAGAACTAGGACAACCCGGAAGCCTAATAGAAGACGACCATGTATACAATGTTATTGTCACCGCCCACGCATTCATCATAATTTTTTCATAGTAATACCAATTATGATTAGGGGTTTTAACGGGAACTGACTTATTCCTTTAATAATTGGCGCTCCCGATATAGCATTTCCTCGAATAAATAACATAAGCTTCTGACTTCTACCACCCTCCCTACTCTACTGCCCACATCATCAACTTTAAAGCCGGTGCCGGCACTGGTTGGACAGTCTACCCACCCCTAGCAGGCAATTTATCCCACCCAGGAGCCTCTGTAGATTTAACCATTTTTTCACTACATTTA

>789d834e-8a6f-4739-832c-07bf90aee640

TTGTACTTTCGTTCAGTTACGTATACTAACAGCTAAATAACCTAATCAACTGGCTTCAATCTACTTCTCCCGCCGTTAGGGAAAAAAAGGCGGGAGAAGCCCCCGTTTAAATTGAAGCTGCTTCTTTCATTACAATTCAATGTGATAGTTCACCTCAGGGCTGGTAAAAGAGGTTCACTCCTCTGTCTTTAGATTTACAGTCTAATGCTTGCTCAGCCATTTACCCCTACCTATGTTAAATAAAACCGCTGACTATTTTCAACTAATCACAAAGACATTGGGACATTATATTTATTATTTGGCGCATGGGCGGGGGCGGTAGGTACAGCCTAAGCCTCCTAATTCGAACAGAACTAGGACAACCCGGAAGCCTAATAGAAGACGACCAGCATACAATGTTATTGTCACGCCCACGCATTCATCATAATTTTTTTCATAGTAATACCAATTATGATTGGGGGTTTGGGAACTGACTTATTCCTTTAATAATTAGCGCTCCCGATATAGCATTTCCTCGAATAAATAACTTAAGCTTCTGACTTCTACCACCCTCCCTACTCCTACTGCTTGCATCATCAACTTTAGAAGCCGGTGCCGGCACTGGTTGGACAGTCTACCACCCCTAGCAGGCAATTTATCACCAGGAGCCTCTGTAGATTTAACCATTTTTTCACTACTTAATACGTAA

>6a192476-b8fe-4627-ad2f-60f42241c78c

TTGTACTTCGTTCAGTTACATTGCTCAACTGAACACAAATCGAATACTTTAATTAAGCTAAGCCCTTCTAGATTGATGGGATTTTAACCCACAAAAAATTTAGTTAACAGCTAAATAACCTAATCAACTGGCTTCCAATCTACTTCTCCCGCCGTTAGGGAAAAAAGGCGGGAGAAGCCCCGGCAAACTGTAACGAATTTGCAATTCAATGTGATAGTTCACCTCAGGGCTGGTAAGAGGGTTCACTCCTCTGTCTTTAAGAATTTACAGTCTAATGCTTGCTCCAGCCATTTACACCCCCTATTCCATTGCCAAACCGCTGACTATTTTCAACTAATCACGGGCATTGGAACCATTATATTTATTATTTGGCGCATGGGCAGGGCGTGGGAGTACAGCCTAAGCCTCCTAATTCGAACAGAACTGAATCAGCCGGAAGCCTAATAGAAAGCGACCATGTATACAATGTTATTGTCACCGCCCACGCATTCATCATAATTTTTTTCATAGTAATACCAATTATGATTGGGGGTTTAAGTGACTTATTCCTTTAATAGTCGCTCCCGATATAGCATTTCCTCGAATAAATAACATAAGCTTCTGACTTCTACCACCCTCCCTACTCCTACTGCTTGCATCGCATCAACTTTAGAAAGCCGGTGCGGCACTGGTTGGACAATCTACCCACCTAGCAGGCAATTTATCCCACCAGGAGCCTCTGTAGATTTAACCATTTTCACTACATTTAGCAGGCATTTCTTCTATTCTTGGAGCTAGTAACTTTATTACAACAATTGTAAATATGAAACCGCCAGCCATGACTCAATGTATGCAAACTCCGTTGTTTGTGTGATCCGTCCTAATTACTGCAGTCCTTCTTTTACTTTCTCTTCCAGTTCTAGCTGCAGGGATTACCATACTATTAACTGACCGTAATTTAAATACTACTTTCTTTGATCCTGCTGGTGGCGGCGACCTATCCTATACCAACACTTATTCTGATTCTTCGGTCACCCTGAAGTATATATTCAATCTTACCGGGTTTTGGAATAATTTCACACATTGTAACATATTATTCTAATAAAGAACCCTTTGGTTATATGGGCATGGTATGAGCCATAATATCTATTGGTTTCTAGGCTTTATTGTATGAGCCATCACATATTCACAGTAGGAATAGATGTATGTGCATATTTTACATCAGCTACCATAAATGTTGCTATTCCACTGGGTAAAAGTATTTAGCTGATTAGCCGCACTGCACGGCAGTAATATCAAATGATCTCCCGCAATATTATGAGCCCTGGGCTTTATTTTTCTTTTTACCGTGGGTGGACTAACAGAATTGTGTTAGCTACTTTCATCATTAGATATTGTATTACATGATACATATGTAGTAGCCCACTTTCACTATGTGTTATCAATAGGAGCAGTATTTGCCATTATAGGGGTTTTATTCACTGATTCCCGCTCTTTTCAGGCTATACTTGACCAAACATATGCCAAAATCCACTTCACCATTATATTTGTCGGCGTAAATTTAACTTTTTCTTCCCACAACACTTCCTTGGCTTTATCGGAATACCTCGACGATACTCAGACTATCAGACATATACTACATGAAATATCGTCTCATCTATCAGTTCATTTATTTCACTTACAACCAGTAGTCCTGATGGTTTTTATAATTTGAGAAGCTTTCTCTTCAAAACGAAAAGTCTTAGCCATTGAGCAGCTATCCACCAATCTAGAATGATTATACGGCTGCCCTCCTCTTACCACACATTCGAAGGCAACACGTTAAATCCTAGACGAAAAAGGAAATGATTTGAACCCCAAGAAATTGGTTTCAAGCCAATCCCATATACCCTATGACTTTTTCAATAAGATATTAGTAAAATAATTGCATAACTTTGTCAAAGTTAGGTGTGTATTACGTAAT

>1c689e13-f14c-4b4b-aee2-6c3ab652f541

TGCTTCGTTCAGTTACGTATTGCTATACCTGAAACAAACTAGCATGAACAATACCCATTATTCCACTAATAATAATATCCCTAGGAGGTCTACCCCCACTAACAGGTTTTTCCCCCAAATGAGCTATTATACAAGAACTTATTAAAAATGATAACTTAATTATTCCCCTTATAATAGCTTTACTAACATTAATAAATTTATATTTTTATATACGTTTAATATATTATATCTCAATGACAATATTCCCAAACATCAAATAACACAAAATCAACTGGCAACTAAATTATATAAAGCCAATACCGTTTCACTCCCACTTGTAGTGTCTACCTGTCTCCCTACCCTAACTCCACTAATACTTATAACTTAGAAATTGGTTAATAAGACCAAGAGCCTTCAAAGCCCTTAGTAAGTAAATTTTTTATAATTTTCTGCACAACAAATAAGGACTGCAAAACTTTATTCTGCATCAACTGAACGCAAATCAATTTAATTAAGCTAAGCCCTTCTAGATTGATGGGATTTTTAACCCACAAAAATTTAGTTAACAGCTAAATAACCTAATCCTTCGGCTTCAATCTACTTCTCCGCCGTTAGGGAAAAAAGGCGGAGAAGCCCCACAACAGGTAAAGCAACTTCTTCAATTTGCAATTCAATGTGATAGTTCACCTCAGGGCTACAGTAAGAGAAGGGTTCACTCCTCTGTCTTTAGATTTACAGTCTAATGCTTGCTCAACCATTTTACCCCTACTTCTGTTACTTAAAACCGCTGACTATTTTCAACTAATCACAAGAACATTACGGGACATTATATTTATTTATTATTTGGCGCATGGGCGGGGGCAGTAAGAGTATCCTAAGCCTCTAATTCGAACAGAACTAGGACAACCCGGAAGCCTAATAGAAGACATATGTATACAATGTTATTGTCACCGCCCACGCATTCATCATAATTTTTCATAGTAATACCAATTATGATTAGGTTTGAACTTGACTTATTCCTTTAAATAATTGGCGCTCCCGATATAGCATTTCCTCGAATAAATAACATAGCTTCTGACTTCTACCACCCTCCTACTCCTACTGCTTGCATCATCAACTTTAGAAGCCGGTGCCGGCACTGGGTTGGACAGTCTACCCACTAACCCAGGCAATTTATCCCACCCAGAGGCCTCTGTAGATTTAACCATTTTCCCCTACATTTAGCAGGCATTTCTTCTATTCTTGGAGCTATTAACTTTATTACAACAATTGTAAATATGAAACCGCCAGCCATGACTCAATATCAAACTCCGTTGTTTGTGTGATCCGTCTAATTACTGCAGTCCTTCTTTTACTTTCTCTTCCAGTTCTAGCTGCAGGGATTACCATACTATTAACTGACCGTAATTTAAATACTACTTTCTTTGATCCTGCTGGTGGCGGCGACCCTATCCTATACCAACACTTATTCTGATTCTTCGGTCACCCTGAAGTATATATTCTATCTTACCGGGTTTGGAATAATTTCACACATTGTAACATATTATTCTAATAAAAAGAACCCTTTGGTTATATGGGCATGGTATGAGCCATAATATCTATTGGTTTCCTAGGCTTTATTGTATGAGCCCATCACATATTCACAGTAGGAATAGATGTAGATACTCGTGCATATTTTACATCAGCTACCATAATCATTGCTATTCCCACTGGAGTAAAAGTATTTAGCTGATAGCAATACGTAG

>66dd06bf-047c-4b10-8851-0568f5b836ae

GATATTACTTCGTTCAGTTACGTGACCCAAATTGCCTGCTAGGGTGGGTAGACTGTCCAGCAGTGCCGGCACCGGCCTAAAGTTGATGATGCAAGCAGTAGGGTAGAGGAGAATTGGTAGAAGTCAGAAGCTTATGTTATTTATTCGAAATGCTATATCAGGAGCAGCAATTATTAAAGGAATAAGTCAGTTCCCAAAACCCCAATCATAATTGGTATTACTATGAAAAAAATTATGATGTGAATGCGTGGGCGATAACATTGTATGGTCGTCTTCTAACATAAGGCTTCCAGGTTTGTCCCTAGTTCCTGTTCGAATTAGGGAAGCAGGAGCTGTACCTGCCCCACCAATGCGCCAATAATAAATATAATGTCCCAATGTCTTTGTGATTAGTTGAAAATAGTCAGCGGTTTGCCAGACATAGGTAGGGTAAAATGGCTAGCGAGCAAGCATTAAGACGTAAATCTAAAGACGGAAAA

>09c72d68-24a1-4ec7-894f-de1f8dd939ba

AGTATACTTCCGTTCAGTTTACATGTTGCTCTTCAAATACTTTTATCCAATTAGAATAGCAATGATTATGGTGGCTGATGTAAAATATGCAGTATCTACATCTATTCCTATATTGAATATGTGATGGGCTCATACAATAAAGCCTAGGAAACCAATAGATATTATGGCTCATACCATGCCCATATAACCAAGGGTTCTTTTTTATTAAGAACTAATATGTTACAATGTGTGAAATTATTCCAACCCACGGTAAAGGATGAATATATACTTCAGGTGACGAAAGAATCAGAATAAGTGTTGGTATGAATGGGTCGCCGCCACCAACGCAGGATCAAAAGAAGGCGTATTTAAATTACCCGGTCAGTTAATATTGTGATAATCCCTGCAGCTAGAACTGGAAAGAAAATAAAAGAAGGACACAAAATGGAAGTAGGACGGATCACACAAACAACGGGTTGATATTAGTCATGGCTGGCGGTTTTCATATTTACAATTGTTGTAATAAAGTTAATAGCTCAAGAATTAAGAAAATGCCTGCTAAATGTAGGCGAAAAAAATGGTTCGTCTGCAGAGGCTCTGGGTGGGATAAGAATTGCCTGCCCAGGGGTGGGTAGACTGTCCAACCAGTGCCGGCACCGGCTTCTAAAAGTTGATGATGCAAGCAGTAGGAGTAGGGAGGGTGGTAGAAGTCAGAAAGCTTATATTGTTATTCAGTATATATCAGAGCGCAATTATTAAAGGAATAGAATCCAGTTCAAAACCCCCAATCATAATTGGTATTACTATGAAAAAATTATGATGAATGCGTGGGCGGTGACAATAACATTGTATACATGGTCGTCTTCTATTGGGCTTCCCGGGTTGTCCTAGTTCTGTTCGAATTAGGAGGCTTAAGGCTGTACCTACTGCCCCGCCCATGCGCAAATAATAAATATAATGTCCCAATGTCTTTGTGATTAGTTGAAAATAGTCAGCGGTTTGCCGAACATGGTAGGGAGTAAAATGGCTGAATATGACATTAGACTGTAAATCTAAAGACGAGTGAACCTCTTGCAGCCTAGGGTGAACTATCACATTGAATTGCAAATTCAAAGAAACGAAGCTACTGGTGCCGAGGCTTCGCTCCCGCCTTTTTTTTTAACGGCGGGAGAAGTAGGTGGAAACCAGTTGGTCAGTTATTGCTTCATTAGCGCTAAATTTTTGTGGGTTAAAATCCCATCCGTCTAGGAAGGGCTTAGCTTAATTAAAGTAATTGATTTCCCGATTCAGTTGATGCAGAATAAAGATTTTTGAGTCATATTGTTGTTGTGCAGAAATTAAGTAAAATTTGCCACTAAAGGCTTTGAAGGCTCTTGGTCTTATTAAACACAAATTTCTAAGTTATAAGTATTAAGTGGAGTTAAGAGCAATACGTAACTTCTTA

>31423757-58fe-4ed1-9dbd-f2e8b4e6d57c

GATATACTTCGTTCAAGTTACATCAGTACTATAACCGCTGACTATTTACAACTAATCACAAAGACATTAGGACATTATATTTATTATTTGGCGTATAAAGGCGAGGCAATGCAGTACAGCCTAAACCTCCTAATTCGAACAGAACTAAGACAGCCGACCTAATAGAAGACGACCATGTATACAATGTTATTTGTACCTTGCATTCATCATAATTTTTTTCCTTAATAATACCAATTATGATTAGGTTCGGGAACGACTTATTCCTTCAATAATTGGCTCTCCCGATATAGCATTTCTCAGGCCAAGCCAACCTTAGCTTCTGACTTCTACCACCCTCCCTACTCCTACTGCATCATCAACTAGAAGCCGAATTGCCGGCACTGGTTGGACAGTCTACCTAACGAGCAGTTTATCCCACCAGAACCTCTATTAGATTTATGTTTTCCTGCAATTTGGCAGAGCATTTCTTTAATGTTGGAGCTATTAACTTTATTACAACAATTGTAAGAAATGCCGAAACGCCAGCCATGACTCAATATCGCTCCGTTGTTTGTGTGATCATCCTAATGCAGTCCTTCTTTTACTTTCTCTTCCCGGTTCTGGCTGCAGGGATTGCCATACTGTTAACTGACCGTAAATTCAAACCTTTCTTTCTTTGATCTGCTGGTGTGACATTACTATCTATGCAACAATACGTAACTT

>e6c5d7b3-5627-47aa-ba4b-ae721fca5f40

AGTGTACTTCGTTCAGTTACGTATTGCTTGAGACGATATTTCATGTAGTATATGCGTCTGGATAGTCTGAGTATCGTCGAGGTATTCCGGATAAGCCAAGGAAGTGTTGTGGAAGAAAGTTAAATTTACGCCGACAAATATAATGGTGAAGTGGATTTTGGCATATGTTTGGTCAAAGAGTATAGCCTGAAAAGAGCGGGAATCAGTGAATAAATCCCCTATAATGGCAAATACTGCTCTATTGATAACACATAGTGAAAGTGGGCTACTACATAGTATGTATCATGTAATACAATATCTAATGATGAGTTAGCCCTAACACAATTCTGTTAGTCCACCCACGGTAAAAGAAAAATAAAACCCAGGGCTCATAATATTGCGGGAGATCATTTGATATTACCGCCGTGCAGTATGGCTAATCAGCTAAATACTTTTACTCCAGTGGGAATAGCAATGATTATGGTAGCTGATGTAAAATATGCAGTATCTACATCTATTCCTACTGTGAATATGTGATGGGCTCATACAATAAAGCCTGGAAACCAATAGATATTATGGCTCATACCATGCCCATATAACCAAAGGGGTTCTTTTTTATTAGAATAATATGTTACAATGTGTGAAATTATTCCGAAACCCGGTAAGATTAGAATATATACTTCAGGGTGACCGAAGAATCAGAATAAGTGTTGGTATAGGATAGGGTCACGCGCCACCAGCAGGATCAAAGAAAGTAGTATTTAAATTACGGTCAGTTAATAGTATGGTAATCCCTGCAGCTAGAACTGGAAGAGAAAGTAAAAGAAGGACTGCAGTAATTAGGACGGATCACACAAACAACGAGGTTTGATGTTAGTCATGGCTGGCGGTTTCATATTTACAATTGTTGTAATAAAGTTAATAGCTCCAAGAATAGAAGAAATGCCTGCTAAATGTGATGTTGAAAAAATGGTTAAATCTACAGAGGCTCCTGGGTGGGATAAATTGCCTGCTAGGGTGGGTAGACTGTCCAACCAGTGCCGGCACCGGCTTCTAAAGTTGATGATGCAAGCAGTAGGAGTGGGGAGGGTGGTAGAGTGAAAGCTTATGTTATTTATTCGAGGAAATGCACTATATCAGGAGCGCCAATTATTAAAGAGACAAGTCAGTTCCCAAAACCCCCAATCATAATTGGTATTTTATGAAAAAAATTATGATGAATGCGTGGAGCAGTGACAATAACATTCATACATGGTCGTCTTCTGTTAGGCTTCCGGGTTGTCCTAGTTCTGTTCGAATTAGGAGCTTAGAGGCTGTACCTACTGCCCCCGCCCATGCGCCCAAATAATAAATATAATGTCCCAATGTCTTTGTGATTAGTTGAAAATAGTCGGCGGTTGCATAGACATAGGTGAGGTAAAATGGCTGAGCAGCATTAGACTGTAAATCTAAAGACAGAGGAGTGAACCCTCTTTTTACCAGCCCTGAGGTGTTGAACTATCACATTGAATTGCAAATTCAAAGAGCAGCTTCAATTCTGCCGGGGCTTCTCCGCCTTTTTCCTAACGGCGGGAGAAGTAGATTGAAAGCGATTGATTAGGTTATTTAGCTGTTAACTAAATTTTTGTGGGTTAAATCCCATCAATCTAGGAAGGGCTTAGCTTAATTAAAGTAATTGATTTGCATTCAGTTGATGCAGAATAAAGGATTTTGCAGTCCTTATTTGTTGTGCAGAAATTAAGTAAAAAAATTTACTTACTAAGGGCTTTGAAGAGCTCTTGGTCTTATTAACCTAAATTTCTAAGTTATAAGTATTAGTGGAGTTAGGGGTAGAGACAGGTAGTACTACAAGTGGGGATAGAAACAGTATTAGCTTTATATAATTTAGTTGCCAGTTGATTTTTGTATTTATTTGATGTTGGGAATATTGTCATTGAGATATAATATATTAAACGTATATAAAAATATAAATAGCAATATTGTAACCCA

>392a0802-8d00-4b89-b241-5c07d282e065

AGTACTTCGTTCAGTTATCTTATTGCTTTAATAAGACCAAGAGCCTTCAAAGCCCTTATTAAGTAAATTTTACTTAATTTCTGCACAACAAATAAGGACTGCAAAACTTTATTCTGCATCAACTGAACGCAAATCAATTACTTTAATTAAGCTAAGCCCTTCCTAGATTGATGGGATTTTAACCCACACAAAAATTTAGTTAACAGCTAAATAACCTAATCAACTGGCTTCAATCTACTTCTCCACGCCGTTAGGGAAAAAGGCGGGAGAAGCTGACAATTGAGTTAGCTGGTGCAATTCAATGTGATAGTTCTTACTCAGGCTGGTAAAAAGAGGGTTCACTCTCTGTGCTTTAGATTTACAGTCTAATGCTTGCTCAGCCATTTTACCCTACCTATGTTCACCGCCATGACTATTTTCAACTAATCACAAAAACATTGGGACATTATATTTATTATTTGGCGCATGGGCGAAGGGCAGTAGGTACAGCCCTAAGCCTCCTAATTCAGACAGAACTAACGGCGGAAGCCTAATAAAAGACGACCATGTATACAATGTTATTGTCACCGCCCGCATTCATCATAATTTTTTCATAGTAATACCAATTATGATTGGGGGTTTAAGGAACTGACTTATTCCTTTAATAAGCTGGCGCTCCCGATATAGCATTTCCTCGAATAAATAACATAAGCTTCTGACTTCTACCACCCTCCTACTCCTACTGCTTGCATCATCAACTTTAGAAGCCGGTGCCAGCACTGGTTGGACAGTCTACCCACCCCTAGCAGGCAATTTATCACAGGAGCCTCTGTAGATTTAACCATTTTTCACTACATTTAGCAGGCATTTCTTCTATTCTTGGAGCTATTAACTTTATTACAACAATTGTAAATATGAAACCGCCAGCCATGACTCAATATCAAACTCCGTTGTTTGTGTGATCCGTCCTAATTACTGCAGTCCTTCTTTTTACTTTCTCTTCCAGTTCTAGCTGCAGGGATTACCATACTATTAACTGACCGTAATTTTAAATACTACTTTCTTTGATCCTGCTGGTGGCGGCGACCCTATCCTATACCAACACTTATTCTGATTCTTCAGTCACCCTATAGTATATGTTCTAATCTTTCAGGTTTTGGAATAATTTCACATTGTAACATATTATTCTAATAAAAAAGAACCCTTTGGTTATATGGGCATGGTATGAGCCATAATATCTATTGGTTTCCTAGGCTTTGTTGTATGAGCCCATCATATTCACAGTAGGAATAATGTAGATACTCGTGCATATTTTACATCAGCTACCATAATCATTGCTATTCCCACTGGGTAAAAATGTTAGCTGATTAGCCACACTGCACGGCAGTAATATCAAATGATCTCCCGCAATATTATGAGCCCTGGGCTTTGTTTTCTTTTACCGTGGGTGGACTAGCCGGGAATTGTGTTGGCTAACTCATCATTAGATATTGTATTACATGATACATACTATGTAGTAGCCCACTGTCACTATGTGTTATCAATAGAATGTATTTGCCATTATAGGGGATTTATTCACTGATTCCCGCTCTTTTCAGGCTATACTCTTGACCAAACATATGCCAAAATCACTTCACCATTATATTTGTCGGCGTAAATTTAACTTTCTTCCTGCTTACTTCTTAGCTTATCCGGAATACCTCGACGATACTCAGACTATCCAGACGCATATACTACATGAAATATCGTCTCATCTATCAGTTCATTATTTCCGCCTTACAGCAGTAGTCCTGATGGTTTTATAATTTGAAGGCTTTCTCTTCAAAACGAAAAGTCTTAGCCATTGAGCAACTATCCACCAATCTAGAATGATTGTCGGCTGCCCTCCTCCTTACCACACATTCGAAGAGGCAACTTACGTTAAATCCTAGACGAAAAAGGAGATGATTTGAACCCCCAAAAATTGGTTCAAGCCAATCCATATACCCTATGACTTTTCAATAAAGATATTATTAAAATAATTACATAACTTTGTCAAAGTTAAGAATTATAATTCGTATCTATATATCTTAATAACAACTTTCAGCTCAACTAGGTTTACAAAATGCCACATCACCTATCGTAAGAGAACTTATTGCTTTCTCCGACCATGCACTCATAATTATTTTCCTAATTCTAGTTCCTTAGTCTTATATATTATTTCCCTTATACTTACCATAAAACTCACTCATACTAGCACCATAAATGCTCAAGAGATCGAAATAATCTGAACTATTCTCCCTGCACTGATTCTAATTACAACAATTGCCACTCCATCACTGCGTATTCTATATATGACAGACGAATTTAATAAACCTTATTTAACCCTTAAAGCAATTGGTCACCAATGAGAAATACTGAACCTATGAATATTCCGACTATGAAGACTTAGTATTTGACTCTTACATTATGCCAACACTTGCTTCCAGCAGGCAGGGGAATTTCCTGACTCCTCGAAGTTGATAGCAATACGTAAC

>bacbe249-f72e-4856-9ab2-b942587509e4

ATGTACTTCGTTCAGTTACGTATTGCTTTTTACTTAATTTCTGCACGCTAAATAAAGGATACAAAACTTTGTTCTGCATCAACTGAACGCAAATCGAATACTTGAACAGCTGACCCTTCCTAGATTGATGGGATTTGTACCCTAAAGAAAATTTAGTTAACAGCTAAATAACCTAATCCAATGGCCAATCTACCTTCCCCGCCGTTGCGGAAAAAGGCGGGCTGACGGGAGTGAAGCTGTAGCCTTTGAATTTGCAATTCAATGTTGATATTCACCTCAGGGCTGGTAAAAAAGAGTTCCTCCTCGTAATATTTAGATTACGATCAATGCTTGCTCCAGCCATTTGCCTACTGATGCCGACCGCCGCTGGCTATTTTTCAACTAATCACAAGACATTGGGACATTATATTTATTATTTGGCACATGACGAGAGCAGTAAGTACAGCCACAATATCTAATTCGAACAGAACTAGGACAACCCGGAAGCCTAATGAGGCGACCGTAACCTTCCGGTATTATTGTCGCTACGCCACGCATTCATCATGATTTTTTCATGGTAATACCAATTGTGATTGGGGTTTTAAGGAACTGACTTATTCCTTTAATAATTAGCGCTCCCGATATAGCATTTCCTCCGTTGACAACACCAAGCTTCACGGCTTCTGCCTCCTATACCATGGCA

>006270e6-eb1a-4110-899e-86cecae9839b

TTGTACTTCGTTCAGTTACGTATTGCTCATGGTCGTCTTCTATTAGCTTCCAGGTTGTCCTAGTTCTGTTCGAAGATTAGGAGGCTTGGGGCTGTACCTACTGCCCCCGCCCATGCGCCAAATAATAAATATAATGTCCCAATGTCTTTGTGATTAGTTGAAAATAGTCGGCGCCCAGTTTGTATGGACATAGGTAGGGGTAAAATGGCTGAGCAAGCATTAGACTGTAAATCTAAAGGCAGAGGAGTGAACCCTCTTTTTACCAGCCCTGGTGAACTATCACATTGAATTGCAAATTT

>c16b0410-7e26-4ff2-8435-6cfe460b84e8

GGTAGCCGCTTCGTTCAGTTACGTACTGCTGACTATTTTCAACTAATCACAAAGACATTGGGACATTATATTTATTATTTGGCGCATGGGCGGGGCAGTAGGTACAGCCCTAAGCCTCCTAATTCGAACGAACTAGGACAACCCGGAAGCCTAATAGAAGACGACCATGTATGTAATGTTATTGTCACCGCCCACGCATTCATCATAATTTTTTTTCATAGTAATACCAATTATGATTGGGGGTTTGAGACGACTTATTCCTTTAATAATTGGCGCTCCCGATATAGCATTTCCTCGAATAAATAACATAATTTCCTGACTTCTACCACCCTCCTAGCAATACGTAACT

>5f979278-6595-47ca-9903-3fea98e2bc2b

ACGTACTTCGTTCAGTTACGTATTGCTAAAATGTAGCTTAAAATAAACGTCAACTTGCAGAAGATGTCATAACAAGTGATCCATTTTGGCTAATCCTAGCCCAACTCTCCATTAAATATATTGTGCATTTATAATTAAAATCATTTACCTATAGTAAGAAGTATAGGCGATAGAAATTACATACTAGGCGCAATAGATATAGTACGTGAGGAAAGACATTACCTAATAAGCATATAAAAGCGAGGCAGTCATACCTTCTGCATAATGAATTAACTAGAAGTAGTTTTATAAAACTTCAACAAAGTACCCCGAAACCAAGCAGACTGCTGCAAGGACAGCTATAGAAGCACACCGTCTATGTGGCAAAATAATTTGGGAAGATCATGGGTAGTGGCGACAAACCTAACGAGCTTGGTGATAGCTGGTTATCAAGACAGAATCTTAGTTCGACTTTCCGAATTTATCCCCAGAATTATTAAATCCTCATGTAAATTTAACTGTTAGTCTAAGAGGACAGCTCTTTGAGCCTAGAGAAAAAACCTTTAATAAAGAGTAAGTAATATAAATCCCATAGTTGTGCAAAAGCCATCAATTAAGAAAGCGTTCAAAACAATACTATCATATATTCAATTCTACCAAATTTTATCGAACTCACAAAACAAAATTGGATTAATCTATTATCTAATAGAAGCAATAATGTTAGTATAAATGTGAATCTATTACTCCCGCATAAGCTTATTTCAGACCAGAAACAACTACTGTTAGTTAACAGCCTATGTCGCAAACTACAAATTAAAATATCAGTCAATAAGCTGTTATTGCTTCCCAGGCGGCACTAAGGAAAGATTAAAAAAGTAAAGGAGCTCGGCTTAGCTCCACTAGCTTTACGCCTGTTTTTACCAAAAACATCACACTCTAGCATTTCTAAATATTAGAGGCACTGCCTGCCCAGTGACATATGTTCAACGGCCGCGGTACCCTGACCGTGCAAAGTGCATAATCACTTGTTCTCTAAATAGGGACTTGTATGAATAGCCACACGAGGGTTTCAACTGTCACTTAATCAGTGAAATTGACCTATCCGGCGAAGGCGGATATACATAAATAAGACGAGAAGACCACGGGCTTTAATTTAATGATACAAACTAGATTTGTAAAAACCAACAGGCATTAATTTACCGTCAATGTATTATAAATTTCAGTTGGGGCGACTACTCAGGTAAAATAAGAGCCTCGAAAAACATATACAGGAACTGCTTCAGTCTAAGTAAGCAAACATCACCACAACCGTTCAATAATAATTGATGTGGATTGTCTACCTATAGGGATAACGAGCATAATCTAACTTTGAGATGATCCATATCGATAATAGGGTTTACAGCGACCTCGATGTTGGATCAAGACATCCTAATGGTGCAAGCTATTAAGGTTCGTTTGTTCAACCGTTGAAGTCTTACGTGATCTGGGTTCAGACCGGAGCAATCCGGAGTCGGTTTCTATCTGTTAAATATTCCCGCCGATGCCACGAAAGGACAAATGGGGCCCTTCATAAGAAGCGCCCTCAACAATCAGATGACCTCCATAAACTCACGCATATTATAATCACGCCAGAACAGGGCTCGTTAAGGTGGCCAGAGCCGGTAGTGCGCAAACTTTAAAACTTTGTAATCAGGTTCAATTCACTCTTCTTAACAACATGTATATAATTAATTTTATAATCTAGTCCTGCCTAATTGCCTGCTTCTTTCACAGCACTCACAGAACGAAAAATCCTGGAAGTATAGCACAATTCCGAAAAGGCCCACATGTTGTAGGCCCGGGTACTTTGAAAGCCAATCGCTGACGCCATAAAACTCTTCACAAAGAACCCTTGTTACCACCACATCCACCATAACTTTATATTTAACTGCCCCACCCTAGCTCTTTCGTGCTCTTCTACTGTGAGCGCCCTCCCCATACATGCATCATATCTTCAATCTTGGTCTCCTATTTATCCTCGCAACATCAAGGCTAACTGTTTGCTCAATTTTATCGATCTGGCTGAGCATCCAACAAACTACGCACTAATTGGCGCACTACGAAATATGCTTAAACAATCTCAGCAGCAGAGTCCCCTTGCCATTATCCACTATCAACACTACTAATAAGCGGCTCTCATTCGATACCACAATCGCTTATTACACTCGAAACTTCCTGACTTCTGCAGCTGCGTGGCCTAGCCATAATATGATTTATTTCCACATGAGAAACTAATCGAGCTCCATTCGACCACGACAGAGGCGGTCAGGGCAGTATCGGGTTTCAACATTGAATACGCGCATGAGCTCATTTAGCATATTCTTCATAGCAGGTATGTAAATATTATTATAATAAATGCCCTAACTACCACTATTTTTTCTTATCCACACCCTACAATATAATTATACCAGAAACATTTACTATTAATTTTATAATAGCTTCCTCACTACTAACTTAACTGTTTATGAATTCGAACAGCCTACCCCCCGCTTCCGCTACGATCAATTAATATTCTTGCGAAAAACTTACCACCACTTACGTGCAGCACTTGTATATGATATGTTCGTACCATCTAGCATCTGGCATCCACTTTAAACATAAGAAATATGCACGATAAAAAGAGATTACTTTGATAGAGTAAATTATAGAGGTTCAAACCCTCTTATTTCTGGATTACAGGAATTGAACCTACTGAGAACTCAAAACTCTCCGTGCTACCGATTACACCATATCCTAAACGATAAGGTCAGCTAAAATAAGCCTATCATTTACCACCCCGAAAATGTTGGTTTAATCAATACAGTGTAGCATTAATCCTCTAGCCACCTTATTCATACCATTCTAACAGGGATAATCACAATTTTAAGCTCACATTGGTTCCTGTATTACCTGAATGAGCAAGAATTAAATATACTAATATCGATGGCAATCCTTGCCAAAAGTATATTCCCCGCTCCACAGAGAGCATCCACTGATATTTTTAATTCCAAACAGCAACATCATACTTCTATTCAATGTCCATTTCCTTAACAATCTACCAATAACAATGTTAATCAATCCTCCTTGTAGCAAATATTATCTAAATAATATTTATTGCTCTGTAATAAAAAGCCGGACCAACCACTTTATGACTCCCACAGAAATTACCCAAAGGAATACTCTAATCCCAGCTATAATTATTCTCCTGCGTGACAAAAACTCGCCCCAATATCAATTCTCCTCCAAATTTTTCCGTCAACAAACCTAAGCAGTTCTAACAATCTCAGTTCTATCAATTATAATTTGGCGGCTGAGGAGGACTCAACCAAACACAACTCCGCAAAATCCTAGCCCCATATTCTTCAATTACTCATAGAATGAATAATAGCAGTAGTATATTCGACCTAATATTTACTATATTAACTGTAATTATTTATATTTTTTCTTTCAATCTCTACATTAATAATCTTTTATTTAACCTCAAATGTAACAACCCTATCCCTATCACATACCTGAAACAAACTAGCATGAACGTACATTATTCCACTAATAATAATATCCCTAGGAGAGTCTACCCCACTAACAGGTTTTCCCCCAAATGAGCTATTATACAAGAACTTATTAAAAATGATAACTTAATTATTCCCCTTACTAATGGCTTTACTAACATTAATAAATTTATATTTTTATATACGTTTAATATATTATATCTCAATGACAATATTCCCAACATCAAATAACACAAAAATCAACTGGCAACTAAATTATATAAAGCTAATGCCGTTTCTATCTTACTTGCTTAGTGTCTTCTACCTGTCTCACCTAACTCCATAATACTTACTTGAAATTTAGGTTAATAAGACCAAGAGCCTTCAAAGCCCATGATAAGTAAATTTTGCTTAATTTCTGCACAACAAATAAGGACTGCAGGCTTTATTCTGCATCAACTGAACAAATCAATTACTTTAATTAAGCTAAGCCCTTCCCGATTGATGGGATTTTAACCCACAAAAGTTTGGTTAACAGCTAAATAACCTAATCAACTGGCTTCAATCTACTTCTCCCGCCGTTAGGGGGAAAAAGGCAGAGCCCGGGTGAAGCTGCTTCTTTGAATTTGCAATTCAATGTGATAGTTCACCTCAGGGCTCTGGTAAAAAGAGGGTTCACTCCTCTGCAATGTTTGGAATTACAGTCTAATGCTTGCTCAGCCATTTTACTACCATGTTTAAGCGCATGACTATTTTCAACTAATAATCACAAAGACGGGACATGTTTATTATTTGGCGCGTGGAGGCGGGGGCGGTAGAGTACAGCCTAAGCCTCTAATTCGAACAGAACTAGGATATGTTGAAGCCTAATAGAATGACCATATGCAATGTTGGTGCTTCTCGCCGCATTCATCATTAATTTTTCATAGTAATTATGATTGGGGGTTTTGGGAACTGACTTATTCCTTTAATAATTGGCGCTCGATATAGCATTTCCTCGAATAAATAACATGACTACAACTACCACCCTCCCTACTCCTACTGCTTGCATCATCAACTTTAGAAGCCGGTGCCGGCACTGGTTGGAAGTCTACCCACCCCTAGCAGGCAATTTATCCCACCAGGAGCCTCTGTGAGTGTAACCATTTTTTCACTACATTTAGCAGGCATTTCTTCTGATAAGAGGCTATTAACTGTACAACAATTAAATATGAAACCGCCAACTTCATGACTCAATATCAAACTCCGTTGTTTGTGTGATCCGTCTAATTACTGCAGTCCTTCTTTTACTTTCTCTTCGGTTTAGCTGCAGGGATTACCGACACTGTGCTGACCGTAATTTAAATACTACTTTCTTTGATCCTGCTGGTGGCGGCGACCCTATCCTATACCAACACTTATTCTGATTCTTCAGTCACCTAGTATATATTCTAATCTTACCGGGTTTTGGAATAATTTCACACATTGTAACATATTATTCTAATAAAAAGAACCCTTTGGTTATGGGCATGGTATGAGCCATAATATCTATTAGTTTCCTATTTGTGTATGAGCCATCATATTCACATTAGGAATAGATGTGAAGTACTCGTGCATATTTTACATCAGCTACCATAATCATTGCTATTCCCACTGGAAGTAAAGAAATATTTAGCTGATTAGCCACACTGCACGGCGGTAATATCAAATGATCTCCCGCAATATTATGAGCCCTGGGCTTTATTTTTCTTTTTACCGTGGGTGGACTAACAGAATTGTGTTAGCTAACTCATCATTAGATGTTGTATTACGATACATATATGTAGTAGCCCACTTTCACTATGTGTGTTATCAATAAAGACAGTATTTTGCCATTATACCATTTATTCACTGATTCCCGCTCTTTTCAGGCTATACTCTTGACCAAACATATGCAAAATCCACTTCACCATTATATTTGTCGGCGTAAATTTAACTTTCTTCCCCAACACTTCCTTGGCTTATCCAGGAATACCTCGACAGCGTCTCAGACTATCAGACGCATATACTACATGGAAATATCGTCTCATCTATCAGTTCATTTATTTCACTTACAGCAGTAGTCCTGATGGTTTTTATAATTTGAGGAAACTTTCTCTTCAAAACGAAAAGTCTTAGCCATTGAGCAACTATCCACCAATCTAGAATGATTATACGGCTGCCCTCCTCCTTACCACACACATTCGAAGAGGCAACTTGCGTTAAATCCTAGACGAAAAAGGAAGGATTTGAACCCCCAAAAATTAGTTTCAAGCCAATCCCTTATACCCTGGCTTTTTCAATAAGATATTAGTAAAATAATTTATACTTTGTCAAAGTTAAATTATAGACTAAATATCTATATATCTTAATAGCAACACCAGCTCAACTAAAGTTTACAAAATGCCACATCACCCTATCATAGAAGAACTTATTGCTTTCCACGACCATGCACTCATAATTATTTTCCTGATTAGTTCCTTAGTCTTATATATTTCCTTATACTTACCACAAAACTCACTCATACTAGCACCATAAATGCTCAAGAGATCGAAATAATCTGAACTATTCTCCCTGCACGCTGATTCTAATTACAATTGCCCTCCCATCACTGCGTATTCTATATATGACAGACGAATTTAATAAACCTTATTTAACCCTTAAAGCAATTGGTCACCAATGATGCTGAACCTATGAATATTCCGACTATGAAGACTTAGTATTTGACTCTTACATTATACCAACATATTTCACTTGAGCCAGGGGAATTTCGACTCCTCGAAGTTGATAACCGAACAACACCGCCTATGAAGCGGATATTCGCATATTAATCTCATCACAAGACGTCTTACACTCCTTGAGCCGTACCATCACTGAGCGTAAAAACAGATGCAATTCCTGGACGTTTAAACCAAGCCATACTAGCCTCTATACGACCAGGCCTATTTTATGGACAATGCTCGGAAATTTGCGGGTCAATCACAGCTTTATACCTATTGTTCTAGAATTCATTTATTTCCAAGATTTCGAGAAGTATGAGCTTCATACTTATATATTGTATCGCTGTAAAGCTAACTTTAGCATTAACCTTTTAAGTTAAAGATTGAGAGAACAAACTCTCTATGGTAGATACCTCAACTAAATATTTCACCGTGACCAATAGTAATTATATCTATAATTATTACCTTATTTTATATTATACAATTGAAAATACTGAACTTTACTTTCCATTATTACCCACTACCAAAATTAGTAGAAACACAAAAACATAAAACAACTTGAGAACTAAAATGAACAAAATCTGTTGCCTCATTCAATATTCCAACAATACTAGGAGTACCCTTATTTTTAATTATGCACTCACCCATTAATTTTATCCTCAAAAAACTTATTAACAACCGACTCTTCAATTCAACAATGGCTAATTCAACTAACACTTAAACAAATAATATTAACCCACCACTAAAGGGCGAACCTGATCCCTTGCTACTCCTAGCTAAAATTTCTTTTATTGCCTAATAACATTCTCGGACTTACCATATGCATTTACACCAACCACCCAACTATAATAAATCTAGGCATAGCTATTCCTCTATGAGCAGCAACTATTACTAATAGGCCTCCCGATTTAAAACAAAATCATCCCTCGCTCATTTTACCACAAAAGAAACACCAATTCCACTAATCCTATATTAATTATTAGCTGCAAACAATTAGTCTATTCATTCAACCTGTAGCCTTAGCCGTACGATTAACAGCCAACATGCAGCAGATTATACCTATTGATCATTTGCCGGGGATACAACATTAACTCTTCTATCAATTACCTCTCATATACAATCACTATCATTATTATTATTTTATTAATTACCCTAGAGTTGGGTATGTAGCCTTAATTCAAACTATGTATTTACCCTCTTAGTAAGCCTGTACTTACATGATAATTCATAATGACTCACCAAACCCATGTATCACATAGTCAACCCAAGCCCTTGACCACTAACAGGAGCATTGTCAGCTTTTCTTCTCACATCCGGCCTAGTTATATGATTCCACTTTACCACACTGCTTCTCACTGCAGGTCTACTAAGCGGATTCTATCACTATTATTTCAATGATGACGTGATGTAGTACGAGAAAGTACATATCAAGGCCACCATACCTGTCAAAAAGGCCTACGATACGGAATAATTCTGTTATTATTTCAGAGATTTTCTTCTTTTGAGTTTCTTCTGGTATTTTATCATTCTAGTCTAGCCCCAACTCCACAAACAGAGACTATGACCACCTACGAGCATTACTCCCTCAACCAATAGAAGTCCCTCTCCAAATACAACCGTACTACTAGCATCAGGAGTTACAATTACATGAGCACATCACAGCCTCATAGAAGCTAACCAAAAAAGAAAGAATCCAACCCAAGCACTCTTTCTAACCATTATATTAGGGATCTGCATACCTGCCTACAATTATCGAATATTCTGAAGCCCCATTTACTATCTCCGACGGAGTATGGATCCCACATTTTTTATGGCTACAGGCTTTCGCCTTCACGTAAATTATCGGAACCACTTTCCTCACCACCTGTTACTTTCGCCAACAATTATATCTTTAGCATCCCAACCACCATTTTGGCGAAGCCTGCATGATATTGACATTTCACGTAGATGTAGTATATTTTCCTCTATATTTCCATCTATTGGTGAGCTCTACTCTCTTAGTATAAAGTATTATTGACTTCCAATCAACTTGAGCCTCGAATGATTCGAGAAAGTATTATAAATTTAATTTTAGCTAATGACTAACATTACTTTGGCCTTACTTCTTATCACAATTACATTTTGACTTCCACAATTAAATATTTATACAGAAGAAAGCACAACCCTTGCGAGTGCGGATTTGATCCTACAACCTCCGCCCGCTTGCATTCTCCGCCAAAATTTTTCCTAATCGCCATCACATTTCTCCTGTTGATCTGAAATTGCCCTATTCTCCCCATCAGGCAACCCAAACAAATAATTTAACTCTAACAATAAATATAATTTTTACCCTACTTATTATTGGCTTTAGGGTTAGCCTACGAGTGGTCCCAAAAGGATTAGATTGGGCTGAATTGGTATATGATTTAATTAAAACAAATGATTTCGACTCATTAGATTATGAAAGCTCATGTTGCAAATATGCCTTTTATCTATATTAATGTAATACTAGCATATTTTCGCGTATCATTATTAGGGTTATTAATTTATCGAATGTCACATATCTCACATCACTGCTATGTTTGGAAGGCATAATATTATCAATTATTTATCATAATTGCCACTCACAACTTTCAATATGCACTTCATATTAATGTATATGATACCCCTCATTCTCTGGTATTTGCCTTTAGCAATACAGTAAGCCTGTATGATGGTGATTATAACCAACCTATATGGCCTAGATTATGTACAAACCTAAACATACAAATGTTAAAATTTATTTTTCCAGCCATCATAATACTTACCACTGACTTTCAAAAAAATTATAATATGAATCAACACAATAATCTGTAGTCTACTAATCAGTATATACGCCCTCATATTACTCCACAAACAACTCATACAACCTATCACTGATTTTCCTCCAGATTCACTAACATCACCACTTCTTATATTAACAGCCCGACTTCTGCCGCTAATAATTCTGCTTAACTTCAACAGCATTTATATAATAACCCCACCCCACGAAAAAAACTATATATCTCTAATATTAATTCTATTATAAATTATTATAACTTTCAGCTACCGAACTAATTTTATTTTATATTCTATTTGAAACTACCTAATCCCACTAATTATTATTACCCGTTGGGGTATCAACCAAACATATATGCGGTTCATATTTTCTATTCTACACTAGCAGGATCTTACCTCTATTAATTACCTCACTATCTAAATACTTTGGGATCCCTAAGCATACTTACAATTATTAATTCCTTCAAATACTTGCCATGAACCAATAGTATTATATGATTGGGATGTATAATAGCTTTTATAGTCAAAATACCCCCATATGGGTTACACCTATGACTCCCAAAAGCTCACGTAGAAACCCCCATCGCTGGCTCAATAGTACCGCAGCAATCTTACTAAAACTAGGAGGGTATGGTATAGTGCGAAGGTACTCCTATCCTCAATCCACTAACAGAAAAAATAAGCTACACTTTTAGATCATTCTATCCTATGAGGGATAGCGTACAGCAAGCTCATCTGCGCGACAAACCGACCTAAAATCACTTATCGCTTACTCCTCCGTCAGCCACATAGCACTTGTTATTTTAGCTATTATAATTCAAACCCCATGAAGCCTTACCGAGGAGAGATTGTAATAATACTAATAATCTCCATGGACTTACCTCATCCCTGCTATTTTGTTTAGCAAATACCTAACTACGAACGAATTCACAGCCAGCGAACTATAAGCTTTAGACCTTCCAAACATTATTTTCCCCTTCTAGCACTTTGATGACTCCTAGCTAACCTAGCAAATTTGACTCTCCCAACTATTAATCTAATAGGTGAACTATTAACAATCTTAGCTTCTTTCTCTTGATCTAATTTTACCATCATATTTGCAGGTTTCAACATGTGATGCAGCCCTCTACTCACTTCACATATTTACCTCAACACAACGAGGCCATTAACGTACAGCACTAACAGCATCAAACCCCTCTTTACACGAGAAAATGTACTAATAGCTAATACATTTAGCAATACCCATCCTACTATTAACTACAAACCCCAAGATAATTATAGGTTTGACGCCCTGTAGTTATAGTTTAATAAAAACATTAGATTGAATTTGTATTAAACCCATAACTTCTTAACTACCGAAAGTATGCAAGAACTGCTAATTCATGCTACCAGGCCTAACAACTTGGCTTCCTCAACTTTTAAAGGATAGTAGTTATCCATTGGTCTGGGTCTTCAAAAATATTGGTGCAACTCCAGAATAAAAGTAAAAATATACTCCTCAATAATTATATTCACTGTTATTCCACTACTAGTACAATCTTAATAACTTCTATAGTTAATTTACACAAAAGCCTCTATATCCATACTTATTACGTAAAACTAGCTATTATTTGTACCCACTCACTGCCAGCATCCTATGTATAGCAATATATATACATCTTTACAGGCCAGAATTGATGATTTCAAACTGACACTGAACTATTCAAACTATCGATTATCACTCAGCTTTAAAATAGATTTTTCTCCACAATATTTGCCCCCGTAGCACTCTTTGTCACCTGATCAATTGTGAATTTCTCAACATGGTATATAAGCTCAGACCCAAACATTAACCAATTCATAAATATCTCATTTTCTTAATTACAATATTAATTTCTAATTACTGCCGACAATCTATTTCAACTTTTTATCCGGATGGGAGGGGATAGGTATTATATCATTTCTATTAATTAGCTGATGGTACGGACGAACAGATGCTAACTTGCGGCAGCTTTGAAATAATTTTATATAACCGGATCGGAGATATCGGTTTTATTTTAGCAATAACTTGTGGTTCTTTATATCATAACTCATGAGACTTTCAACAAATATTTATACTAGATTATACTTCCTAACTCTTTTCCCTAACAGCTTACTTTTAGCAGCAACAGGAAAATCTGCTCAATTTGGTCTACATCAATAAATTACCACTCCCGCTATAGAAGGGCCCACCCCCAGTGTCAGCACTACTGCACACTCGCACAATAGTTTGCAGGAATCTTTTAATTATCCGTTTTTATCCTTTTAATAGAAATAATCGATTTATGCAAACAATAGCGCTATCGCGGCGCAATCACACTATTTTGTAATTTGTGCCCCTAACACAAAATAAACTTAAAAAATTTAGCCTTTTTCTACCTCAAGCCAACTAGGCGCGCAATAATGACAATTGGTATTAACCAACCACACCTAGCCTTCCTCTCATATCTGCACTCACGCCTTCTTCAAAGCCATATTATTTCGTAATACGTAACT

>13c75f7b-2753-4bb3-a3fa-684f9dbc4faf

ATACTTCGTTCAGTTACATGTGCTCCCGCTTCACTTCTGACTCCCGAAATTACCCAAGGAATCCCTCTAATCCGCTATAATTATTCTCACGTGACAAAAACTCGCCCCAATATCAATTCTCCTCCAATTTTCCGTCAACAAACCTAAACTTGATTCTAACAATCTCAGTTCTATCAATTATAATTGGCAGCTGAGGAGGACTCAACCAAACACAACTCCCTCAAAATCCTAGCCTATTCTTCAATTACTCGCATAGGATGAATAATAGCAGTATTATATTACGACCCTAATATTACTATATTGTCTTTAATTATTTATATTTTCCTAACAATCTCTACATTAATAATCTTTTGTTTAACCTCAAATGTAACAACCCTATCCCTATCCCTTTGAAACATATTACTTGAATTAAATACCCATTATTCCACTAATAATAATATCCCTAGAGGAGTCTACCCCCACTAACAGGTTTTTTCCCCCAAATGAGCTATTATACAAGAACTTATTAAAAATGATAACTTAATTATTCCTTATAATAGCACCTTTAACATTAATAAATTTATATTATATGCGTTTAATATATTATATCTCAATGACAATATTCCAACATCAAATAACACAAAAATCAACGGCAACTAAAATTCATATAAAGCCATCACCGTTTCTATCCCCACTTGTAGTGTCTTCACTATAATCTCCTACCTAACTCACTAATACCATAACTTGAGAAATTTAGGTTAATAAGACCAAGAGCCTTCAAAGCCTTAGTAAGTAAATTTTTACTTAATTCTGCACAACAAATAAGGACTACCAAAACTTTATTCTGCATCAACTGAACATAGTCGTTACTTTGGTAAGCTAAGCCCTTCCCCTAGATTGATGGGATTTTTAACCCACAAAAATTTAGTTAACAGCTAAATAACCTAATCAACTTCTTCAATCTACTTCTCCCGCCGTTAGGGGGAAAAAAAGGCGGGAGAAGCCCCCGGCAGAATTGAAGCTGCTTCTTTTGAATTTGCAATTCAATGTGATGGTTCACCTCAGGGCTGGGTAAAAGAGGTTCGCTCTCTGTCTTTAGATTTACAGTCTAATGCTTGCTCAGCCATTTTTACCCTACCTATGTTCTGAAGCCATGACTATTTTCAACTAATCACAAAGACATTGGGACATTATATTGTTTATTATTTGGCCATAGGCAGGGAGCAGTAAATTTTGGCCCACAGCCTCTAATTCGAACAGAACTAGGACAACCGGAAACTAATAGAGAAGACGACCATGTATACAATGTTATTGTCACCTTTTTTGCATTCATCATAATTTTTTTCATAGTAATACCAATTATGATTGGGGGTTTTAAGGAACTGACTTATTCCTTTAATAATTGGCGCTCCCGATATAGCATTTCCTCGAATAAATAACATAAATATGACTGCCACCTCACATACTACTGCTTGCATCATCAACTTCAGAAGCGATTGTAAACACTGGTTGGACAATTACCACCTAGCGACAATTTATCCCGCAGGAGCCTCTGTAGATTTAACCATTTTTCACTACATTTAGCAGGCATTTCTTCTATTCTTGGAGCTATTAACTTTATTACAACAATTATGAATGTTGAAACCGCCAACCATGACTCAATATCAAACTCCGTTGTTTGTGTGATCCGTCCCTAATTACTGCAGTCCTTCTTTTACTTTCTCTTCCAGTTCTGCTGCAGGGATTACCATACTATTAACTGACCGTAATTTAAATACTACTTTCTTTGATCCTGCTGGTGGCGGCGACCCTATCCTATACCGACCACTTATTCTGATTCTTCGGTCGCCCTGGTATATATTCTAATCTTACCGGGTTTTGGAATAATTTCACACATTGTAACATATTGTTGTAAAAAGAACTAACTTTGGTTATATGGGCATGGTATGGCCATAATGTCTATTGGTTTCTGGCTTTGTGTATGAGCCATCACGCATATTCACAGTAGGAATAGATGTAGATACTCGTGCATATTTTACATCAGCTACCATGATCATTTGCTATTCCCACTGGAGTAAAAGTATTTAGCTGATTAGCCACACTGCACGGCGGTAATATCCAAGTGATCTCCCGCAATATTATGAGCCCTGGGCTTTATTTGCACCTTTTTACCGTGGGTGGACTAACAGGTGTGTTAGCTAACTCATCGTACATAGTATTGTATTACATGATACATACTATGTAGTAGCCCACTTTCACTATGTGTTATCAATAGGAGCAGTATTTGCCATTATAGAGTTTATTCACTGATTCCGCTCTTTTCAGGCTATACTCTTGACCAAACATATGCCAAAATCCACTTTCACCATTATATTTGTCGGCGTAAATTTAACTTTCTTCCCACAACACTTCCTTGGCTTATCCGGAATACCTCGACGATACTCAGACTATCCAGACGCATATACCATGAAATATCGTCTCATCTATCGGTTCATTTATTTCGCAGCAGTAGTCCTGATGGTTTATAATTTGAGAAGCTTTCTCTTCAAAACGAAAAAGTCTTAGCCATTGAGCAACTATCCACCAATCTAGAATGATTATACGGCTGCCCTCCTCCTTACCACACATTCGAAGAGGCAACTTACGTTAAATCCTAGACGAAAAAGGATTTGAACCCCCAAAAATTGGTTTCAAGCCAATCCCATATACCCTATGACTTTTTCAATAAGATATTAATGGAAAGCAATTACATAACTTTGTCAAAGTTAAATTATAGACTAAATATCTATATATCTTAATAGCAACACCAGCTCAACTAGGTTTACAAAATGCCACATCACCTCATAGAAGAACTTATTGCTTTCCACGACCATGCACTCATAATTATTTTCCTGATTAGCAATACGTAAC

>a1fc2a12-71e1-4487-b469-93f40840c8ea

AGTATGCTTCGTTCCGGTTGCGTGGCCCCATGGTAGAAGTCAGAAGCTTATATTATTTATTCAGGAAATGCTATGTCGGAGCACCAATTGTATGCAAGGAATAAGTCAGTTCCCGAAACCCCAATCGCAATTGGTATTACTATGAAAAAGGTATGAAGTGAATGCGTGGGCAGTGACAATAACATTGTATACATGGTCGTCTTCTATAATATTGAAACTTCCGGGGACGCCTAGTTCTGTTTGATTAGGGGCTTGGGGCTGTACCTACTGCCCCGCCCATACGCCAAATAATAAATATAATATCCCAATGTCTTTGTGATTAGTTGAAAATAGTCAA

>b6b9d666-3aa0-4d83-9640-de88b50eed0f

GTGCAACCGTTCAGTTACGTATTGCTTGATGCAAAGCAACGACTATTTTCAACTAATCACAAAGACATTGGGTTTAAAATTTATATTTTTAATATTGCATGGGCGGGGGCAGTGGAGCACAAGCCTCAAGCGCTCTAGTCAAACAGAAGACTCGAGGACAACCCCTGAGCCTAATGAACTTGACCATGTATACAATGTTATTGTCACCACACGCATTCATCATATAATTTTTTCATAGTAATACCAATTATGATTGGGGTTTCTAGACTGAGCTTATTCCTTTAATAATTGACGCTTTCCAATCTGCTTGTTTCCTCGAAATAATAACATAAACTTCTGACTTTACCACCCTCCTACTCTACTGCTTGCATCATCAACATCCAGTGCCGGCACCTGGTTGAATGATCTACCCACCCTGCGGAGAGCAATTTATCACTTTAAGACAACTCTGTGATTTAACCATTTTTTCACTACATTTAGCAGGCATTTCTTCTATTCTTGGAGCTGTGCTTTATTACAACAGAAATTGTAATGAAACCGCCATGCAGGCTCAATATCCAACTCCGTTGTTTGTGTGATCCGTCAATGACTGCAGTCCTTCTTTTACTTTCTCTTCCAGTTCTAGCTGCAGGGATTACCATACTAATTAACTGACCGTAATTTAAATACTACTTTCTTTGATCCCTGCACAATTGGCGGCGACCTATCCTATACCAACACTTATTCTGATTCGCTGAGTCACTACAAGTATATATTCTAATCTTACGGAGTTTTGGAATAATTTCACACATTGTAACATATTATTCTAATAAAAAAATTACTTGGTTATATGGGCATGGTATGAACCATAATATCTGGTTTCTAAACTGTTGTGTGAGCCCATCACATATTCACAGTAGAATGAATGTGATCACTCGATACATGTTTACATCAGCTACCATAATCGTGCTATTCCCACTGGAGTAAGTATTTAGCTGATTAGCCACTGCACGGCGGTAATATCAAATGATCTCCCTGTCTTAGCGGGCCCTGGGCTTTATTTTCTTTTTACCATTAGGTGGACTAACGGGAATTGTGTTAACTAACTCATCATTAGATATTACATATTACATGATTACATACTATGTAGTAGCCCACTTTCACTATCATGTTATCAATAGGAAATGATATTGCCGATAACAATACGTAA

>5b62d994-9d0c-48c0-8c03-fcc13b8b6c5e

ATTCTTCACGTTCAGTTACGTATTGCTGCTATTTATTTTCAACTAATCACAAAGACATTGGGACATTATATTTATTATTTGGCCATGGGCGGGGGCAGTAGAGTACAGCCTAAGCCTCCTAATTCGAACAGAACTAGGACAACCCGGAAGCCCTAATAGAAGACGACCATGTATACAATGTTATTGTCACCGCCCACGCATTCATCATAATTTTTTCATAGTAATACCAATTATGATTGGGGGGTTCGAACAATATTCCTTTAATAATTGGCGCTCCCGATATAGCATTTCCTCGAATAAATAACATAAGCTTCTGACTTCTACCACCCTCCCTACTCTACGCTTGCATCATCAAC

>6e5f19c9-97ef-48e2-8a8d-b28f9ec24e71

GATATACTTACGTTCAATTTACGTATTACTATGTTCTAAACTTCATATTCTTCAACTAATCACAAAGACATTGGGACATTATATTTATTATTTGGCGCATGGGCGGGGGCAGTAGGTACAGCCTAAGCCTCCTAATTCGAGGAGCAGAACTGGGACAACTATTAATAGAAGACGACCATGTATACAATGTTATTGTCACCGCCCACGCATTCATCATAATTTTTTTCACTTGAAAGTACAATTATGATTGGGGTTTCAGAACCTGACTTATTCCTTTAATAATTGGCGCTCCCGATATAAAGCATTTCCTCGAATAAATAACATAAGCTTCTGACTTCCACCCTCCCTACTCCTACTGCTTGCATCATCAACTTTGGACGGTGCCAGCACTGGTTGAACTTGATCTACCACCCCCAGGCAATTTGTCACCACAGGAGCCTCTGTAGATTTAACCATTTTTCACTACATTTAGCAGGCATTTCCTATTCTTGGAGCTATTAACTGTTAACAATTGTAAATATGAAACCGCCAACCATGACTCAATATCAACTCCGTTGTTTGTGATCCGTCCTAATTACTGCAGTCCTTCTTTACTTTCTCTTCCAGTTCTAGCTGCAGGATTACCATACTATTAACTGACCGTAATTTAAATACTACTTTCTTTGATCCTGCTGGTGGCGGCGACCTATCCTATGCCAGCACTTATTCTGATTCTTCGATGCCTGAAGTATGTATTCTAATCTTTCCACGGGTTTGGAGAATAATTCTTATTGTAATATATTATTCTCAATTCGAAAGAACCTTTTGAAGACTATCTGGTATGGTGCCTAGAAAGCCATAATATCTGTGTTTCTAGGCACACATTAATATACGGGCCATCTATTTTATTTACTTATTCACAGCCGAATAGATGTAGATGCTCGTGCATATTTTACATCAGCTACCATAATCGTTTAACTGTTCCACTGGAGTAAAAGTGTTTGGCTGATTGGCCACTGCACGCGGTAATATCAAATGATCTCCCGCAATATTATGAGCCCTGAGCTTTATTTTCTTTTTTTACCGTGGGTGGACTAACGGGAATTGTGTTAGCTAACTCATCATTAGATATTAACTGACCACTTCGTTACATACTATATGGTAACCACTACTATATTTATCAATAGAAGACACAGTATTTGCCATTATAGAGGTTATTCACTGATTCCCGCTCTTTTCAGGCTATACTCTTGACCAACATATGCCAAAATCCATACACCATTATATTTGTCGGCGTAAATTCGCTTTCTTCCACAACACTTCTGGCTTATCCGAATACCTCGGCGATACTCAGACTGTCCAGACATATACTACATGAAATATCGTCATCTATCGGTTCATTTATTTCACTTACAGCAGTAATAGTCCTGATGGTTTTTATAATTTGAGAGCTTTCTCTTCAAAACAGAAAAGTCTTAGCAGAAACGAGCAACTATCCACCAATCTAGAATGATTAGCAATGCGTAATAATACTA

>bb186964-7fb6-48fb-afde-168e38336b2f

TTGTACTTCGTTCAGTTAATATTCTTTGCCAATAGAATAATAAACCAAGGGCAGAAGAAATGCTAAATGTAGTGAAAAAATGGACAAATCTACAAGGCTCCTGGGTGGGATAATTTGCACCATGGGGTGGGTGAGCTGTCAGCAGTGCAGCACAAGCTACCAAAGTTGATGATGCAAGCAGTAGGAGTAGGGAGGGTGGTGAAGTCAGAAGCTTATGTTATTTATTCGAGGAAATGCTATGTGCTCAATTATTATTAAGAGAACAGTCAGTTCCAAAACCCCAATCATAATTGAAGTATTACTATGAAAAAAATTATGATGAATGCGTGGGCAGTGACAATAACATTGTATACATAGTCGTCTTCTATTAGGCTTCCGGGTTGTCGGTTCTGTTCGAATTAGGGAGGCTTAGGGCTGTACTGCCCCCGCCCATGCCGCCAAATAATAAATATAATGTCCCAATGTCTTTGTGATTAGTTAG

>5fb670c6-f143-4e66-911d-3467515e38b9

ATTGTACTTCGTTCAGTTACGTATTGCTGGGTGAACTGTCCAACCAGTGCCGGCACCGGCTTCTAAAGTTGATGATGCAAGCAGTAGGAGTAGGGAGGGTGGTAGAAGTCAAGAAACATGTTATTTATTCGAGGAAATGCTATATCGGGAGCGCCAATTATTAAGGAATAAGTCAGTTCCCAAAACCCCCAATCATAATTGGTATTACTATGAAAAAATTATGATGAATGCGTGGGCAGTGACAATAACATTGTATACATGGTCGTCTTCTATTAGGCTTCCGGGTTGTCTAGTTCTGTTCGAATTGGAAGCAGAAGGCTAACCTACTGCCCCGCCCATGCACGCCAAATAATAAATACTTAATGTCCCAATGTCTTTGTGATTAGTTTGAA

>6f164922-0383-40cb-825f-c9b1b280f6b3

GGTATGCTTCGTTCAGTTACGTATTACTTGCAGGGAGAATAGTTCGAATTATTTGATCTCAGGCATTTTATGGTGCTAGTATGAGTGAGTTTTGTGGTAAGTATAAAGGGAAATATTATATAAGACTGAGGAACTAATCAGGAAAATAATTATGAGTGCATGGTCGTGGAAAGCAATAAGTTCTTCTATGATAGGTGATGTGGCATTTTGTAAACCCAGTTGAGCTGGTGTTGCTATTAAGATATATAGATATTTGGTCTATAATTTAACTTTGACAAAGTTATGTAATTATTTACTAATATCTTATTGAAAAAAGTCATAGGGTATATGGGATTACGAGCTGGAAACCAATTTTTGGGGGTTCAAATCCTTCCTTTTCGTCTAGATTTGTGTAAGTTGCCTCTTCGAATGTGTGGTAAGGAGGAGGCAGCCGTATAATCATTCTAGATTGGTGGATAGTTGCTCAATGGCTAAGACTTTTCGTTTTTGAAGAAAGCTTCTCAAATTATAAAAATTCATCAGGACTACTGCTGTAGTGAAATAAATGAACCGATAGATGAGACGATATTTCATGTAGTATATGCGTCTGGATAGTCTGAGTATCGTCGAGGTATTCCGGATAAACGAGAGTATTTGTGGAAAAGAAAAGTTAAATTTACGCCATTAAATATAATGGTGAAGTGGATTTTGTATGTTTGGTCTGGTAGCTACAAGCGACAGGAATCAGTGAATAAATCCCCTATAATGGCGGTACTTTGCTCTATTTGATAACACACATAGTGAAAGTGGGCTACTACAAGTATGTATCATGTAATACAATATCTAATGATAGGTTAGCGCACAGTTTCCTGTTAGTCCACCCACGGTAAAGAAAAATAAAGCCAGAATATAATATTGCGGGAGATCATTTTGATGTTACCGCCGTGCAGTGTGGCTAATCAGCTAAATACTTTTACTCCAGTGGGAATAGCAATGATTATGGTAGCTGATGTAAAATATGCACAAGTACATCTACATCTATTCCTACGTGAATATGTGATGGGCTCTACAATAAAGCCTAGGAAACCAATAGGTATTGTGAAACCATACCATGCCCATATAAACCAAGGGTTCTTTTTATTAAAATAATATGTTACAATGTGTGAAATTATTCCAAAACCGGTGATTAGAATAGCACTTCAGGGTGACCGAAGAATCAGAATAAGTGTTGGTATAGGATGGGGTCGCCGCCACCAGCAGGATCAAAGAAAGTAGTATTTAAATTACGGTCCAGTTAATAAGCGTGTTAATCCCTGCAGCTAGAACTGGAAGAAAGTAAGAAGGACTGCAGTAATTAGGACGGATCACACAAACAACGGAGTTTGATATTGAGTCATGGCTGGCGGTTTCATATTTACAATTGTTGTAATAAAGTTAATAGCTCAAGAATAGAAGAAATGCCTGCTAAATGTGGTGAAAAAATGGTTAAATCTACAGAGGCTCCTGGGTGGGATAAATTGCCTGCTAGGGGGTGGGTAGACTGTCCAACCAGTGCCGGCACCGGCTTCTAAAGTTGATGATGCAAGCAGTAGGGTAGGGAGGGTGGTAGAAGTCAGAAGCTTATGTTGTTATTTATTCGAGGAAATGCTATATCCGGGAGCGCCAATTATTAAAGGAATAGTCAGTTCCCAAAACCCCAATCATAATTGGTATTACTATGAAAAATTATGATGAATGCGTGGGCAGTGACAATAACATTGTATACATGGTCGTCTTTATTAGGCTTCCGGGTTGTCCTAGTTCTGTTCGAATTAGGAGGCTTAGGGCTGTACCTACTGCTACTTCCATGCGCCAAATAATAAATATAATGTCCCAATGTCTTTGTGATTAGTTAGCAATACGTAACT

>e1bc8010-67d0-4d26-ac01-426fdc2b98ed

TTGTACTTCGTTCAGTTGTGTGTAATCACAAAGAGAACATTGGGACATTATATTTATTATTTGGCGCATGGGCGGGGGCGGTAAATTACAGCCCTAAGCCTCCTAATTCGAACAGAACTAGGACAACCTGACCCTAATAAGATGACCATGTATACAATATTGTTGTCACCGCCCACGCATTTCATCATAATTTTTCATAGTAATACCAATTATGATTGGGGTTTTCGAACGACTTATTCCTTTAATAGTGGCGCTCCCGTTAGCATTTCCTCGAATAAATAACATAAGCTTCTGACTTCTACCACCCTCCCTACTCCTACTGCTTGCATCATCAACTTTAAGAAGCAGTGCCGGCGCTATGCGTTGGACAGTCTACCCCACCCCAGCGAGCAATTTATCCCGCAGGAGCCTCTGTAGATTTAACCATTTTTTCACCCCATTTTGTAGGCATTTCTTCTATTCTTGAACTATTACGCTTTTATTACAACAATTGTAAATATGAAACCGCCAATATGACTCAATATCAAACTCCGTTGTTTGTGTGATCCGTCCTAATTACTGCGATCACTTCTTTTTCTCTTCCAGTTCTAGCTGCAGGGATTACCATACTATTAACTGACCGTAATTTGACTACTTTCTT

>fdd43c08-3539-49c9-b47f-05932ec260ee

GTGTACTTCGTTCAGTTACGTATTGCTTGCTAAATGCTTAAGTGAAAAAAATGGTTAAATCTACAAGGCTCCTGGAGTGAGATAAATTGCCTGCTAGGGGTAAGGTAGACTGTCCAACCAGTGCCGGCACCGGCTTTACAAAGTTGATGATGCAAGCAGTAGGAGTAGGGAGGGTGGTAGAAGTCAGAAGCTTATTATTTATTCGAGGAAATGCTATATCAGAACAATTATTAAAGGAATAAGTCAGTTCCGGAAACCCCCAATCATAATAATTGGTATTACTATGAAAAAAATTATGATGAATGCGTGGGCGGTGACAATTAACATTGTATACATGGTCGTCTTCTATTAGGCTTCCGGGTTGTCTAGTTCTGTTCGAATTAGGAGGCTTAGGCTACCTACTGCGCCCTTGCACCAAATAATAAATATAATGTCCCAATGTCTTTGTGATT

>45c69cd2-aef8-4847-9938-99212f0ab7bd

AAACACTTCGTTCAGTTACGTATTGCTCTTTTACTCAATTGGGAATAGCAATGATTATGGTAGCTGATGTAAAATATGCACGAGTATCTACATCTATTCCTACTGTGAATATGTGATGGGCTCATACAATAAAGCCTAGGAAACCAATAGATATTATGACACCTTACCATGCCCATATAACCAAAAGGGTTCTTTTTTTATTAGAATAATATGTTACAATGTGTGAAATTATTCCAAAACCCGGTAAGATTAGAATATATACTTCAGGGTGACCGAAGAATCCAGAATAAGTGTTGGTATAGGATGAGGTCGCCGCCACCAGCAGGATCAAAGAAAGTAGTATTTAAATTACGGTCGGTTAATAGTATGGTAATCCCTGCAGCTAGAACTGGAAGAGAAAGTAAAAGAAGGACTGCAGTAATTAGGACGGATCACACAAACAACGGGTTTGTTCTATTGAGTCATGGCTGGCGGTTTCATATTTACAATTGTTGTAATAAAGTTAATAGCTCCAAGAATAGAAGAAATGCCTGCTAAATGTAGTGAAAAAATGGTTAAATCTACAGAGGCTCCTGGGTGGGATAAATTGCCTGCTAGGGGTGGTAGACTGTCCAACCAGTGCCGGCACCGGCTTCTAAAGTTGATGATGCAAGCAGTAGGAGTAGGGAGGGTGGTAGAAGTCAGAAGCTTATGTTATTTATTCAAATGCTATATCGGGAGCGCCAATTATTAAAGGAATAAGTCAGTTCCCAAAACCCCAATCATAATTGGTATTACTATGAAAAAAATTATGATGAATGCGTGGGCGGTGACAATAACATTGTATACATGGTCGTCTTCTATTAGGCTTCCGGGTTGTCCTAGTTCTGTTCGAATTAGGAAGCTTGGGCATACTACTGCCCCCGCCATGCGCCAAATAATAAATATAATGTCCCAATGACCAATACAGCGTCTAACTATTTATCAATGAGTTAGACATACCAGTATTGTCCTCTAATATGATACCTAAGTGTAAATCACCGTTAGAACGTAACTGCTGATTTTCAGCAGTTAGCATCAACATTTTTCAAAAAAACATGTTATTAATTGGTATTGCAGAGCTGCCAAATTAGGCAGCCTTGACATTCACCTTTTTGTTCGAATTATAATTACATGGGATTAGGGACATGAAGTCGCACTCCCCCTCCATCAGTCGGGTGAGTATATCTTCTATATAATCGCCAAAATTAATCTCATTCAACTTGCAGCTCTCAAAAAAGTGAGAACATAAAGCTGTTTTCCACCTAAATGGCTACCGATCACTTTTTCGAAATATTCACTTGTTTCCATTATCAAACTGTTTTGATTACGGATGCAAAGGTAATATATTATATAGGTATATGCATACGGGCTATTTAAAGGAGATTCACGAGATGTTGAAAATAAGGAATGATTGGATCTAACATTTACTTTTGTATAAAGCTAAGGCAAAGTGCCACTTTTCCTACATCACTGCAAGTTTTAGCTGTAAGACGATTAGCTTCTTTCCTTGATGTACCCAACCGGAGATAACTTGTTCATATCACCTTCAGGAGAATTCGCAAACTTTGACTTGCTATTCCAAAGCACTTGTGATAGTAACGAAGCACATTAGGATAAACATAATCAAGTTCATCGCCAAATTGTTTTCACAAGCTACAAGTTCTTTCCGCAGCACTTTTTAAACTTCAAACCACTTCCACGGACATGGGTCATTTCGCCCAATGCCCTTATAGGGATTAACTACAGGCTCCTGTACGCCATTCACCAAAGCGGTCTAACTCCTTAGGACCCAGTTGTCCTAATAAAATCCTTGTGCGTTAAATGCCGAAACAGGAATCTTATCTTCAGATATTTTTCACCTGAATTATTTGGATTAGTCCATGCTTTGATATGTACTTCCTTGAGGTTTAGAGCAGAAAAAATTGGCAAAATCTCTGTAAATAGAAATCCTTGGCATTAATCTCACCCGTAATCTTCAGATTCTTAATCTTGGTATAGTCCTTATTAGCCGCAGTAATGGCATTCTTCAAACCTCCAGGAGTAGAGTTGATGACAATATAAGCAATACGTAACTA

>250caaae-541d-4b59-b384-e0db25ded1e1

ATGCTTCGTTCAATTACATGTACTATTGAGACATTATATTTATTATTTGGCGCATGGGCGAGGCAGTAGGTACAGCCCTAAGCCTCTAATTCGAACAGAACTAGAGACAACCCGGAAGCCCTAATAGAAGACGACCATGTATACAATATTATTGTCACCGCCCACGCATTCATCATAATTTTTTTCATAGTAATACCAGCGGTATGATTGGGGGGTTTTGAACTGACTTATTCCTTTAATAATTGGCGCTCCCGATATAGCATTTTCCTCGAATAAATAACATAAGCTTCTGACTTCACCTCCCTGCTCCTGCTGGCATCATCATTTAAAGCCGGTGCCGGCACTGGTT

>657fc487-66f9-470c-9523-9f54eafac290

AGTATGCCCCGATTTCAGTTGTACATGTTACTAAGAATAGAAGAAGTAAATGCTTGATGATGAAAAAAATGGTTAAATCTACAGAAAACTCCTGGGTGGGATAAATTGCCTGCTAGGGGTGGGTAGACTGTCCAACCAGTGCCGGCACCGGCTACAAAGTTGATGATGCAAGCAGTAGGAGTGGGAAGGGTGGTAGAAGTCAGAAGCATATTATTTTATTCGAGGAAATGCTATATCACGGAGCCGCCAATTATTAAAGGAATAAGTCAGTTCCCAAAACCCCAATCATAATTGGTATTACTATGAAAAAAAATTATGATGAATGCGTGGGCAGTGACAATAACATTGTATACATGGTCGTCTTCTATTAGGCTTCCGGGTTTGTCCTGGTTCTGTTCGAATTGGGAGGCTTGGGGGCTGTGCCTACTGCCCCCGCCCATGCTTAAATAATAAATATAATGTCCCAATAATACTGTGATTAGTAGCAATACATG

>780f8235-1799-4ea9-b0bc-13bad1a9242a

GATGCAGCATTCAGTTACGTATTGCTGGACATTATATTTATTATTTGGCGCATGGGCGGGGGGCAGTAGGTACAGCCCTAAGCCTCCTAATTGAACAGAACTGAGACAGCCCGGAAACTAATGAGAGGCGGCCATGTATACAATGTTATTGTCGCCACGCATTCATCATAATTTTCATATTGTACCAATTATGATTGCGGGGTTTTCGAACGACTTATTCCTTTAATAATTGGCGCTCCCGATATAGCATTTCCTCGAATAAATAACAGCTTCTGACTTCTACCACCCTCCCTACTCCTGCTGCTTGCATCATCAACTTTAGAAGCCGGTGCCGGCACTGGTTGGACAGTCTACCCACCCCTAGCAGGCAATTTATCACCCAGGGCCTCTG

>3ca0833c-f67c-4186-aa8d-9f0c8b129755

ATTGTACTTCGTTCAGTTACGTATTGCATATTTATTATTTGGCACTGGGCGGGGGCAGTAGGTACAGCCCTAAGCCTCTGAACAGAACTAGGACGAGCGGAAGCTAATAGAAGACGACCATGTATACAATGTTATTGTCACCGCCCACGCATTCATCATAATTTTTTTCATAGTAATACCAATTATGATTGGGGGTTTTGGGAACTGACTTATTCCTTTAATAATTGGCGCTCCCGATATAGCATTTCCTCGAATAAATAACATAACAAGCTTCTGACTTCACCACTCCCTACTCCTACTGCTTGCATCATCAACTTTAGAAGCCGGTGCCGGCACTGGTTGGACAGTCTACCCACCCCTAGCAGGCAATTTATCCCACCCACAGGAGCCTCTGTGGATTTAACCATTTTT

>ca9fddd7-c0de-4fd3-8cc0-b5ecbbda03cb

ATTGTACTTCGTTCAGTTACGTATTGCTATTTATTATTTGGCGCATGAGGCGGGGGCAGTGAGTACAGCCCTAAGCCTCTAGTCCCGAACAGAACTGGGACAACCCGAGAGCCTAATAGAAGACGACCATGTATACAATGTTATCACCCGCCCACGCATTCATCATAATTTTTCATAGTAATACCAATTATGATTTGGGGTTTTAAGAACTGACTTATTCCTTTAATAATTGGCGCTCCCGATATAGCATTTCCTCAAAATAAATAACATAAGCTTCTGACTTCTACCACCCTCCTACTCCTACTGCTTGCATCATCAACTTTAGAAGCCGGTGCCGGCACTGGTTAGACAGTCTACCCACCCCTAGCAGGCAATTTATCCCACCCAGGAGCCTCTGTAGATTTAACCATTTTTCACTACATTTAGCAGGCATTTCTTCCTTCTTGGAGCTATTAACTTTATTACAACAATTGTAAATATGAAACCGCCAGCCATGACTCAATATCAAACTCCGTTGTTTGTGTGATCCGTCCTAATTACTGCAGTCCTTCTTTTTTACTTTCTCTTCCAGTTCTAGCTGCAGGGATTACCATTGCAATAACTGACCGTAATTTAAATACTACTTTCTTTGATCCTGCTGGTGGCGGCGACCCTATCCTATACCAACACTTATTCTGATTCTTCGGTCACCCTGAAGTATATATTCTAATCTTACCGGGTTTGGAATAATTTCACACATTGTAACATATTATTCTAATAAAAAAGAACCCTTTGGTTATATGGGCATGGTATGAGCCATAACCATCTATTGGTTTCCTAGGCTTTATTGTATGAGCCCATCACATATTCACAGTAGGAATAGATGTAGATACTCGTGCATATTTTACATCAGCTACCATAATCATTGCTATTCCCACTGGAGTAAAAGTATTTAGCTGATTAGCCACACTGCACGGCGGTAATATCAAATGATCTCCCGCAATATTATGAGCCCTGGGCTTTATTTTTCTTTTTACCGTGGGTGGACTAACAGGTGTGTTAGCTAACTCATCATTAGATATTGTATTACATGATACATACCTATGTAGTAGCAGCAATACGTAACTTAA

>88dc3e51-164f-4c35-86dc-7fa55b1cb1ae

AGTATACTTCGTTCGGTTACGTATTATGTGGGATAAATTGCACTGAGGTGGGTAGACTGTCCAACCAGTGCCGGCACCGGCTTCTAAAGTTGATGATGCAAGCAGTAGGAGTAGGGTGGTAGAAGTCAGAAGCTTATGTTATTATTTATTCAGTGCTATATCAGGAGCGCCAATTATTAAAAGAATAAGTCAGTTCCCAAAACCCCCAATCATAATTAGTATTACTATGAAAAAATTATGATGAATGCGTGGGCGGTGACAATAACATTGTATACATGGCGATACGTCTTCTATTAGGCTTCGGGTTGTCTAGTTCTGTTCGAATTAGGAGGCAGGGCTGTACCTACTGCCCCCGCCCATGCGCCAAATAATAAATG

>60cb2d8a-1be9-43ac-935b-7f8dabe803fd

TTGTACTTCGTTCTAGTTACGTATTGCTCAGAGGCTCCTGGGTGGATAAATTTACTGCTAGGGGTGGGTAGTGTCAACCAGTGCCGGCACCGGCTTCTAAAGTTGATGATGCAGCAGTAGGAGTAGGGAGGGTGGTAGAAGTCAGAAGCATATTTATTTATTCAGGAAATGCTATATCGGGAGCGCCAATTGTCAAAGGTGGAGTCGGTTCCCAAAACCCCAATCATAATTGGTATTACTATGAGTACCAGTAGACCGTGGGCAGTGACAATAACATTGTATACATGGTCGTCTTCTATTGACTTCAGGTTGTCCTGATTCTGTTCGAATTAGGAGGCTTGGGAGCTGTACCTACTGCCCCCGCCCATGCGCCAAATAA

>0601da0d-fd7f-443c-8b79-dcb8d6ce6778

AGTACTTCGTTCAGTTACGTATTGCTGCATGGGCGGGACAATGCGTTACAGCCTAAACCTCTAATTCGAACAGAACTAAGGACAACCCGGAAGCCTAATAGAAGACGACCATGTATACAATGTTATTGTCTGCCCACGCATTCATCATAATTTTTCATAGTAATACCAATTATGATTAGGGGTTTTAAGGAACGAGCTTATTCCTTTAATAATTGGCGCTCCCGATATGGCATTCCTCCAATAAATAACATAAGCTTCATTGACTTCACCACCCTCCCTACTCCTACTGCTTGCATCATCAACTTTAGAAGCCGGGTGCCGGCACTGGTTGGACAGTCTACCCACCCCCTAACGAGCCAATTTGCTCCACCCAGGAGCCTCTGTAGATTTAACCATTTTTTCACTACATTTAGCAGGCATTTCTTCTATTCTTGGAGCTATTAACTTTATTACAACAATTGTAAATATGGAAACCGCCAGCCA

>1184f301-9055-44a2-8911-7b86eeae0db1

ATGTACTTCGTTCAATTGCATGTTTCTGTAAAACGTGGAAGTAGGGAGGGTGGTGATAAGAAACTTATATTATTTATTCGAGAAATGCTATATCGGGGAGCGCCAATTATTAAAGGAATAAGTCAGTTCCCAAAACCCCCAATCATAATTGGTATTACTATGAAAGAAATTATGATGAATGCGTGGGCGGTGACAATAACATTGTATACGGTCGTCTTCTATTAGGCTTCCGGGTTGTCCTAGTTCTACTGATACAATTAGGAAGCGGGCTGTACCTACTGCCCCCGCCCATGCATAAGCC

>b70ef145-bd75-4027-ae01-225cb1f535fb

ATTGTACTTCGTTCAGTTACGTATTGCTGGCGGGGCGGTAATTACAGCCCTAAGCCTCCTAATTCGAACAGAACTAGGACAACTTGAGCTTAATAGAAGACGATGTGTATACAATGTTATTGTCTGCCCACGCATTCATCATAATTTTTCATAGTAATACCAATTATGATTGGGGGGTTTTGGGAACTGACTTATTCCTTTAATAATTGGCGCTCCCGATATAGCATTTCCTCGAATAAATAACATAAGCTTCTGACTTCGCCACCCTCCCTACTCCCATAGCCATCATCAACTTTAGAAGCGGTGCCGGCACTGGTTTGGACAGTCCCACCCCTGAGGCAATTTATCCCACCCAGGAGCCTCTGTAGATTTAACCATTTTTTCACTACATTTAGCAGGCATTTCTTCTATTCTTGGAGCTATTAACTTTATTACAACAATTGTAAATATGAAACCGCCAGCCATGACTCAATATCAAACTCCGTTGTTTGT

>c3ff2f68-8919-4bc6-b71f-b97f43f588b4

GTATACTTCGTTCAATTACATGTATTGCTCCTGTATACATAAAAATGTGGATCCATATACTCAAATGGAAGTATATTAAATGGGAGCTTCAAGAATATTCCTGATAATGTTAGGCAGAGTGAAGTGATCCCTAATATAATGGTTAGGAGTAGTGCTTGGTTGATTCTTTTCAAGTTAGCTTCTATGAGGCTGTGATGTGCTCGTATAATTGTAACTCTGATGCTAGTAGTGCGGAATTTGTATTCAGAGAGGACTTCTATTGGGTTGAGGGGGAGTAATGCCTGTAGGTGGTCATAGTCCCCCTGTTTGTGGAAGTTAGAGGCTGTAGACTAGAATGATAAAATGCTCAGAAGAAACCTGCAAAGAAAATTTCTGAAATAATATTAGGGTATTCGTATCGTAGGCCTTTTTGGACAGGTGCAGTATGGTGGCCTTGATATGTACTTTCGTACTATATCCACGTCATCATTGAAGCGTCTACATTTATAGAACTAGCTAGTAGACCTGCTACGAGTGAGAAGCAGTGTGTGGTAAAAGTGAATCGTATAACTAGGCGGATGTGAGAAGAAAAGCTGATAATGCTCCTGTTAGTGGTCCAAAGACGGGTTGACATGTGATAAGCATGGGTTTGGTGAGTCATTATGAATTATCATGTAAAGTACAGGCTTACTAAGAGGGTAAATACATAGGCTTGAATTAAGGCTACACAACTCTAGGGTAATTAATGAAATAAATAATGATGAGTGATTGTGGAAGTGGAGAGGTAGAACAATGAAGAGTTAATGTTGTATCCCCAAGTAAAAATGCATTAATAGGTGACCTGCTGTAATATTGGCTGTTAATCGTACGGCTAAGGCTACAGGTTGAATGAATAGACTAATTGTTTCAATAATAATTAATATAGGGATTAGTGGAATTGGTGTTCCTTGTGGTAAAAAATAGGCGAGGATGATTTTGTTTTAAATCGGAGCCCTATTAGTACAGTTGCTGCTCATAGAGGAATAGCTATGCCTAGATTTATTGACAGTTGGGTGGTTGGTGTAAATGCATATGGTGTAAGTCGAGAATGTTATTTAGGGCAATAAAGAAATTAGGGCTAGGAGTATAGGGATCAGGTTCGCCTTTAGTGGTGTGGGTTAATATTATTTGTTTAAGTGTTAGTTGAATTAGCCATTGTTGAATTGAAGAGAGTCGGTTGTTGAATAAGTTTTAGAGATAAAATTAATGTAGTGGGGGTGCGTAATTAAAAATACTAAGGTACTCTAGTATTGTTGGAATATTGAATAGAGCAAATAGATTTTAGTTCATTTAGTTCTCAAGTTGTTTATGTTTTTGTGTTTCTACTAATTTTGGTGGTGGGTAATAATGGAAAGTAAAGTTCGGTATTTTCCAATTGTATAATATAAAATAAGGTAATTATGAAATGTAATTACTATTGAGTCACGGTGAAGTATTTGGTTGGGACGTTTCACTATAGAGTTTGTTCTCTCAATCTTTAACTTAAAAGGTTAATGCTAAGTTAGCTTTACAGTGATACAATATATAAGTATGAAGCTCATGCGCTTGAAATCTTGAAATAAATGAATTCTAGAACAATAGGTATAAAGCTGTGATTGGACCCGCAAATTTCCGGGCATTGTCCATGAAATAGGCCTGGTCGTATAGAGGCTAGTATGGCTTGGTTTAAACGTCAGGTGCATCTGTTTTGCCTAGTAAGTACGGCTCGCGAGTTAATAAAGACACGTCTTGTGATGAGATTAATATGCGAATATCCGCTTCTATAGTGAGATTGTTTCAGGTTATCAACTTCGAGGAGTCAATTCCCTGGCTCAAGGAAATATGTTGGCATAATGTCAGAGTCAAATACTAAGTCTTCATAGTCGGAATATTCATGGGTTCAGTATCATTGGTGACCAATTATTTAAGGGTTAAATAAAGGTTTATTAAATTCGTCATCATATATAGAATACGCAGTGATGGGAGGGCGGTGTAATTAGAATCAGTGCAGAATAGTTCAGATTATTTCAGTCTGCCGAGCATTTATGGTGCTAGTGTAATTGAGTTTTGTGGCTTAGTATAAGGAAATAATATATAAGATTAAGGAACTAATCAGGAAAATAATTAGTGCATGAATATTGAAAGCAATAAGTTCTTCTATGATAGGTGATGTGGCATTTTGTAAACCTAGTTGAGCTGGTGTTGCTATAAGATATATAGATATTTAGTCTATAATTTAGCTTTTTGACAAAGTTATCATTATTTTACTAATATCTTATTGAAAAAGTCATAGGGTATATGGGATTGGCTTGAAACCAATTTTTGGGGGTTCAAATCCTTCCTTTTTCGTCTAGGATTTAACGTAAGTTGCCTCTTCGAATGTGTGGTAAGGAGGGCAGCCGTATAATCATTCTAGATTGGTGGATAGTTGCTCAATGGCTAAGACTTTTCGTTTTGAAAGCTTCTCAAATTATAAAAACCATCAGGACTACTGCTGTAAGTGAAATAAATGAACCGATAGATGAGACGATATTTCATGTAGTATATGCGTCTGGATAGTCTGAAATTATCGTCGAGGTATTCCGGATAAGCAGGAAGTGTTGTGGGAAGAAAGTTAAGAATTTACTTAAACAAATATAATGGTGAAGTGGATTTTGGCATATGTTTGGTCAAGAGTATGTACAAGAAAAGAGCGGGAATCAGTGAATCAATCCCCCTATAATGGCAAATACTGCTCTATTGATAACACATAGTGGTGGGCTACTACATAGTATGTATCATGTGGCACAATATAATGATGAGTTAGCTAACACAATTCCTGTTAGTCACCCACGGTAAAAGAAAAATAAAGCCCAGGGCTCATAATATTGCAGAGATCATTTTGATATTATGGTAGGTGTGACTAATCAGTAATCTTTTACTCAGTGGGAATAACAAGCTGATTATGGTGACTGATGTAAATATACACGAAATGTCTGCATCTATTCCTACTGTGAATATGTGATGGGCCTGTACAAATAAAGCCTGGGAAACCAATAGATGTATAACGATACATACCATTGCCCATATAACCAAGGTTCTTTTTTATTAGATAATAATATTTACAAATGCGTGAAATTATTCCAAAACCAGTAAGATTAGAATATATACTTCAGGGTGACCAGAAGAATCAGAATAAGTGTTGGTATAGGATAGGGTCGCCGCCACCAGCAGGATCAAGAAAGTAGTATTTAAATTACGGTCAGTTAATAGTATGGTAATCCCTGCAGCTAGAACTGGAAGAGAAAGTAAAGAAGGACTGCAGTAATTAGGACGGATCACACAAACAACGGAGTTTGATATTGAGTCATGGCTGGCGGTTTCATATTTACAATTGTTGTAATAAAGTTAATAGCTCCAAGAATAGAAGAAATGCCTGCTAAATGTAGTGAAAAAATGGTTAAATCTACAGAGGCTCCTGGGTGGGATAAATTGCCTGCTAGGGGTGGGTAGACTGTCCAGTGCCGGCACCGGCTTCTAAAGTTGATGATGCAAGCAGTAGGAGTAGGGAGGGTAGTGAGAAGTCAGAAGCTTATGTTATTTATTCGAGGAAATGCTATATCGGGAGCGCCAATTATTAAAGGAATAAGTCAGTTCCCAAAACCCCCAATCATAATTGGTATTACTATGAAAAAAATTATGATGAATGCGTGGGCGGTGACAATAACATTGTATACATGGTCGTCTTCTATTAGGCTTCAGGTTGTACTCGTTCTGTTCGAATTAGGAAGCAGCGGGCTGCTTTACTGCCCCAACCAGCTTTATG

>98e24e5c-7489-4eb1-b5f9-6028b8e5329d

TTATACTTCGTTCAGTTACGTATTGCTAGTAGGTACAGCCCTAAGCCTCCTAATTCGAACAGAACTAGGACAACCCGGAAGCCTAATAGAAGACGACCATGTATGCAATGTTATTGTCACCGCCCACGCATTCATCTAATTTTTTTTTCATAGTAATACCAATTATGATTGGGGTTTACAGGAACTGACTTATTCCTTTAATAATTGGCGCTCCCGATATAGCATTTCCTCGAATAAATAACATAAGCTTCTGACTTCTACCACCCTCCCTACTCCTACTGCTTGCATCATCAACTTTAGAAGCCGGTGCCGGCACTGGTTGGACAGTCTACCCCACCCCTAGCGGGCAATTTATCCCCACCCAGGAGCCTCTGTAGGTTAACCATTTTTTCA

>2bf695c7-9bed-4ec8-bcf4-402d2b735839

GATGTACTTCGTTCAGTTACGTATTGCTGATCAAAAGAAAATAGTATTTAAATTACGGTCAGTTAATAGTATGGTAATCCCTGCAGCTAGAACTGGAAGAGAAAGTAAAAGAAGGACGGCTGCAGTAATTGGGACGGATCACACAAACAACGGAGTTTGATATTGAGTCATGGCTGGCGGTTTCATATTTACAATTGTTGTAATAAAGTTAGTAGCTCCAAGAATGAGAAGAAATGCCTGCTAAATGTAGTGAAAAAATGGTTAAATCTACAGAGGCTCCTGGGTGGGATAAATTGCCTGCTAGAGGTGGGTAGACTGTCAACCAATTGCCGGCACCGGCTTCTAAAGTTGATGATGCAAGCAGTAGGAGTAGGAGGGTGGTAGAAGTCAGAAGCTTATGTTATTTATTCGAGGAAATGCTATATCGGGAGCGCCAATTATTAAAGGAATAGTCAGTTCCCAAAACCCCCAATCATAATTGGTATTACTATGAAAAAAATTATGATGAATGCGTGGGCAGTGACAATAACATTGTATACATGGTCGTCTTCTATTAGGCTTCCGGGTTGTCCTAGTTCTGTTTCGAATTAGGAAGCTTAGGGCTGTACCTACAGCAATACGTAACTTAA

>425ee820-dd9a-408c-a835-1daa0ee76814

ATTGTACTTCGTTCAGTTACGTATTGCTGTGTGGGAATAGCAATGATTATAGTAGCTGATGTAAAATATGCACAGTATCTACATCTATTCCTACTCGAATATGTGATGGAGCTCATACAATAAAGCCTAGGAAACCAATAGATATTATGGCTCATACCATGCCCTTCAACCAAGGGTTCTTTTTTTATTAGAATAATATGTTACAATGTGTGAAATTATTCAAAACCAGTAGATTAGAATATACTTCAGGGTGACCGAAGAATCAGAATAAGTGTTGGTATAGGATAGGGTCGCCGCCACCAGCAGGATCAAAAGAAAGTAGTATTTAAATTACGGTCAGTTAATAGTATAGTAATCCCTGCAGCTAGAACTGGAAGGAAAGTAAAAGAAGGACTGCAGTAATTAGGACCGGATCACACAAACAACGGAGTTTGATATTGAGTCATGGCTGGCGGTTTCATATTTACAATTGTTGTAATGAAGTTAATAGCTCAAGAATAGAAGAAATGCCTGCTAAATGTAGTGAAAAATGGTTAAATCTACAGAGGCTCCTGGGTGGGACAAATTGCCTGCTAGGGGTGGGTAGACTGTCCAACCAGTGCCGGCACCGGCTAAAGTTGATGATGCAAGCAGTAGGAGTAGGGGTGGTAGAAGTCAGAAGCTTTACCTTATTTATTCGAGGAAATGCTATATCGGGAGCGCGGTATTAAAGGAATAAGTCAGTTCCCAAAACCCTAATCCTGAGTGGTATTACTATGAAAAAAATTATGATGAATGCGTGGGTGTGACAATAACATTGTATACATGGTCGTCTTCTATTAGGCTTCCGGGTTGTCTAGTTCTGTTCCGAATTAGGAGGCTTGGGGCTGTACCT

>2b2f4580-1259-484b-a154-e0e19a6db469

TTGTACTTCAGTTACGTATTGCTGTACAGCCTAAGCTATAATTCGAACAGAACTAGGACAACCCGGAGCTAATAAGAAGACGACCATGTATACAATGTTATTTGTCACCGCCCACGCATTCATGCTAATTTTTTTCATAGTAATACCAATTATGATTGGGGGTTTCTTTGGGAACTGACTTATTCATGCAGCAATTGGCGCTCCCGATACTCAGCATTCCTCGAATAAATACCATAGCTTCTGACTTCTACCGCCCTCCCTACTCCTACTGCCCATCACATCAACTTTAAACCGGTGCCGGCACTTTTCGGTTGGACAGTCTACCACACCTAACTTGGGCAATTTA

>fc7de2a8-6d58-4ec1-985e-2ce764eac13d

ATTATTACTTCGTTCAGTTACGTATTGCTGTACAGCCCTGACTCCCTAATTCGAACAGAACTAGGACAACCCGGAAGCCTAATAGAGAAGACGACCATGTATACAATGTTATTGTCACCGCCCACGCATTCATCATAATTTTTTCATAGTAATACCAATTATGATTAGGGGTTTTAGGGAACTGACTTATTCCTTTAATAATTGGCGCTCCCGATATAGCATTTCCTCGAATAAATAACATAAACTGACTACCACCCTCCCTACTCCTACTGCTTGCATCATCGCTTTAAGAAGCCGGTGCCGGCACTGGGTTGGACAGTCTACCCACCCCTAGCAGGCAATTTATCTCCCACCCAGGAGCCTCTGTAGATTTAACCATTTTCACTACATTTAGCAGACATTTCTTCTATTCTTGGAGCTATTAACTTTATTACAACAATTGTAAATATGAAACCGCCAGCCATGACTCAATATCAAACTCCGTTGTTTGTGTGATCCGTCCTAATTACCGCAGTCCTTCTTTTACTTTCTCTTCCAGTTCTAGCTGCAGGGATTACCATACTATTAACTGACCGTAATTTAAATACTACTTTCTTTGATCCTGCTGGTGTGGCGATTACTATCCTATACCAACACTTATTCTGATTCTTCGGTCACCCTGAAGTATATATTCTAATCTTACCGGGTTTGGAATAATTTCACACATTGTAACATATTATTCTAATAAAAAAGAACCCTTTGGTTCATGTGCGGTGCATATTTTTGGGCCATAATATCTATTGTTCTAGACTCTTTTATTATATGAATGCCTCACATATTCGATAGGAATAGATGTAGATACTCGTGCATATTTTACATCAGCTACCATAATCATTGCTATTCCCACTGGAGTAAAAGTATTTAGCTGATTAACCACACTGCACGGCGGTAATATCAAATGATCTCCCGCAATATTATGAGCCCTGGGCTTTTTATTTTCTTTTTACCGTGGGTGGACTAACAGGAATTGTGTTAGCTAACTCATCATTAGATATTGTATTACATGATACATACTATGTAGTAGCCCACTTTCACTATGTGTTATCAATAGGAGCAGTATTTTGCCATTATAGGGGGGATTTATTCACTGATTCCCGCTCTTTTCAGGCTATACTCTTGACCAAACATATGCCCGAAATCCACTTCACCATTATATTTGTCGGCGTAAATTTAACTTTCTTCCCACAACACTTCCTTGGCTTATCCGGAATACCTCGACGATACTCAGACTATCCAGACGCATATACTACATGAAATATCGTCTCATCTATCAGTTCATTTATTTCACTTACAGCAGTAGTCTGATGGTTTTTATAATTTGAGAAGCTTTCTCTTCAAAACGAAAAGTCTTAGCCATTGAGCAACTATCCACCAATCAGCAATACGTAGCA

>9c69b9b4-f520-4a15-88c9-ee31b1209c65

TTGTGCAGCTGGTTCGGTTACGTATTGCTACAGCCCTAAGCCTCTAATTCGAACAGAACTAGGACAACCCGGAAGCCTAATAGAAGACGACCATGTATTTAATGTTATTGTCACCGCTTTTTGCCTTCATCATAATTTTTCATAGTAATACTGGAAGAATTCATAGTTAAGGTTTAAGGAGCGACTTATTCCTTTAATAATTGGCGCTCCCGATATAGCATTTCCTCGAATAAATAACATAAGCTTCTGACTACACCGCCCTCCCTATCCTCCCTACTGCTTGCATCCTCAACTTTAGAAGCCAAGAATTGCTGGCACTGGGTTGGACAGTCTACCCACCTAGCAGGCAATTTATCACAGGAGCCTCTGTCAGGCCCCAACCATTTTCACTACATTTAGCAGGCATTTCTTCTATTCTTGGAGCTATTAACTTTATTACAACAATTGTAAATATGAAACCGCCAGCCATGACTCAATATCAAACTCCGTTGTTTGTGTGATCCGTCCTAATTACTGCAGTCCTTCTTTTACTTTCTCTTCCAGTTCTAGCTGCAGGGATTACCATACTATTAACTGACCGTAATTTAAATACTACTTTCTTTGATCCTGCTGGTGGCGGCGACCCCCCTCCTATACCAACACTTATTCCTGATTCTTCCGGTCACCCTGAAGTATATATTCTAATCTTACTGGGTTTTGGAATAATTTCACACATTTGTAATATATTATTCTAATAAAGACCTGGTTATATGGGCATGGTGAGCCATAATATCTATTGGTTTCCTGAGCTTTATTTATGAGCCCATCCCACATATTCACAGTAGGAATAGATGTAGATACTCGTGCATATTTTACATCAGCAATACGTAACTA

>b612615a-ca2d-48dd-a451-b61456349f1f

TTGTACTTCGTTCAGTTACGTATTGCTCAGCCCTAAGCCTCCACAATTCGAACAGAACTAGGACAACCCGGAAGCCTAATAGAAGACGACCATGCTAATGTTATTGTCACCGCCCACGCATTCATCATAATTTTTTTCATAGTAATACCAATTATGATTGGGGTTTTGGGAACTGACTTATTCCTTTAATAATTGGCGCTCCCGATATAGCATTTCCTCGAATAAATAACATAAGCTTCTGACTTCTACCCTCCCTACTCTACTGCTTGCATATCAGCTTTAGAAGCCGGTGCCAACTGGTTGAACAGTCTACCCACCCCTAACCCAGGCAATTTATCCCCGCAGACCTCTGTAGATTTAACCATTTTTCACTACATTTAGCAGGCATTTCTTCTATTCTTGGAGCTATTAACTTTATTACAACAATTGTAAATATGAAACCGCCAGCCATGACTCAATATCAAACTCCGTTGTTTGTGTGATCCGTCCTAATTACTGCAGTCCTTCTTTTACTTTCTCTTCAGTTCTAGCTGCAGGTTACCATACTATTAACTGACAAGTAATTTAAATACTACTTTCTTTGATCCTGCTGGTGGCGGCGACCCTATCCTATACCAACACTTATTCGATTCTTCGGTCACCCTGAAGTATATATTCTAATCTTACCGAGGTTTTGGAATAATTTCACACATTGTAACGATATTATTCTAATAAAGAATAACGGTTATATGGACGCAGTATGAGCCATAATATCTATTGGTTTCTAGGCTTTATTGTATGAGCCCATCATATTCTTGGTAGGAATAGATGTAGATACTCGTGCATATTTTACATCAGCTACCATAATCATTACTATTCCCACTGGAGTAAAAGTATTTAGCTGATTAGCCACACTGCGGCGGTAATATCAAGTGATCTCCCGCAATGTTATGAGCCCTGGGCTTTATTTTCTTTTTACCGTGGGTGGACTAACAGGAATTGTGTTAGCTAACTCATCATTAGATATTGTATTACACATGATACATACTATGTAGTAGCCCACTTTCACTATGTGTTATCAATAGGAACAGTATTTGCCATTATAGGGGGGATTTATTCACTGATTCCCGCTCTTTCCAGGCTATACTCTTGACCAAACATATGCCAAAATCCACTTCACCATTATATTTGTCGGCGTAAATTTAACTTTCTTTCCCCCACAACACTTCAAGCAATACGTAG

>db0a9a44-8eb2-4f2e-8002-bc069e42f654

AGTAGCCTTTAGTTCAGTTACGTATTGCTGCCCTAAGCCTCCTAATTCGAACAGAACTAGGACAACCGGAGCCTAATGAAGACGACCATGTATACAATATTATTGTCACCGCCCACGCATTCATCATAATTTTTTTCATAGTAATACCAATTATGATTAGGGGTTTTACAGAACTGACTTATTCCTTTAATAATTGGCGCTCCCGATATAGCATTTCCTCGAATAAATAACATAAACTTCTGACTTCTACCACCCTCCCTACTCTACTGCTTGCATCATCAACTTTAAAAGCCAGTGCCGGCACTGGTTGGACAGTCTACCCACCCCTAGCAGGCAATTTATCCACCCAGGATACTCTGTAGATTTAACCATTTTTTCACTACATTGCTTGAGCGTTTCTTCTATTCTTGGAGCTATTAACTTTATTACAACAATTGTAAATATGAAACCGCCAGCCATGACTCAATATCAACTCAAGTTGTTTGTGTGATCCGTCCTAATTACTGCAGTCCTTCTTTTACTTTCTCTTCCAGTTCTAGCAGGGATTACCATACTATTAACTGACCGTAATTTAAATACCTTCACTTTCTTTGATCCTGCTGGTGGCGGCGACCCTATCCTATACCAACACTTATTCTGATTCTTCGGTCACCTGAGTATATATTCATCTTACCGGGTTTTGAATAATTTCACACACATTGTAACATATTATTCTAATAGACCACTGGTTATATGGGCATGGTATAGAGCCATAATATCTATTGGTTTCCTAGGCTTTATTGTATGAGCCCCATCACATATTCACAGTAGAATAGATGTAGATACTCGTGCATATTTACATCAGCTACCATAATCCCATTGCTATTCCCACTGGAGTAAAAAGTATTTAGCTGATTAGCCACGCTTGCACGGCGGTAATATCAAATGATCTCCCTGCAATATTATGAGCCCTAGGCTTTATTTTCTTTTTACCGTGGGTGGACTAACAGGAATTGTGTTAGCTAACTCATCATTAGATGTTAGTATTACCATGATACGCTACTATGTAGTAGCCCACTTTCACTATGTGTTATCAATAGATGCAGTATTTGCCATTATGGGGATTTATTCACTGATTCCCGCTCTTTTCAGGCTATACTCTTGACCAAACATATGCCAAAATCACTTCACCATTATATTTGTCGGCGTAAAATTGCTTTCTTCCCACAACACTTCCTTGGCTTATCCGGAATACCTCGACGATGCACTCAGACTATCCAGACGCATATACTACATGAAATATCGTCTCATCTATCAGTTCATTTATTTCACTTACAGCAGTAGTCCTGATGGTTTTTATAATTTAGAAAGCTTTCTCTTCAAAACGAAAAGTCTTAGCCGTGAGCAGCTATCCACCAATCTAGAATGATTATACGGCTGCCCTCCTCCTTACCACACATTCGAAGAGGCAACTTGCGTTAAATCTAGACGAAAAAGGAAAGTTTTGAACCCCAAAAAATTAGTTTCAAACCCAATCCCATATACCCTATGACTTTCAATAAGATATTAGTAAAATGAATTACATAACTTTTTGATAGTTAAATTATAGACTAAATATCTATATATATCTTAATAACACCAGCTCAACTAGGTTTACAAAATGCCACATCACCTATCATGAAGACATTGCTTTTCACGACCATGCACTCATAATTATTTTTGATTAGTTTCATAGTCTTATATATTATTTCCTTATACTTGAAACTCACTCATACTAGCACCATAAATGCTCAAGGAATCAAGGAAATAATCTGAACTATTCTCCTACTGATTCTAGTACAATTGCCTCTACATCTGCGTATTCTATATATGACGAACTAATTTAACCGACATATTACCCTTAAACAATTGGTCACCAATGATACTGAACCTATGAATATTCCGACTATGAAGACTTAGTATTTGACTCTTACATTATGCCAACATATTTCCTTGAGCCAGAATTTCAGGCTTCCTCAGAAGTTGATAACCGAACAACTTTACCTATAAAGCGGATATTCGCATATTAATCTCATCACAAGACGTCTCTTACACTCATGAGCAGTACCATCACTAGGCGTAAAAACAGATATGAAATTCCTGGACGTTTAAACCAAACCTTACTAGCCTCCATACGACCAGGCCTATTTTATGGACAATGCTCCGGAAATTTGCGGGTCCAATCACAGCTTACCTATTGTTCTAGAATTCATTTATTTCCAAGATTTCCCGGTAATGAGCTTCATGCCCTTATATTGTATCACTGTAAGGCTAACTTAGCCAACCTTTTAAGTTAAAGATTGAGAGAACAAACTCTCTATAGTGAATGCCTCAACTAAATATTTCACCGTGATAATAGTAGTATATCTATAATTATTTACTGTGATATATTATACAATTGAAAATACTGAACTTTACTTTCCATTATTACCCACTACCAAAATTAGTAGAAACACAAAAACATAAAACAACTTGAACTAAATGAACCAAATCTATTTGCCTCAATCAATATTCCAACAATACTAGAATTACCCTTAGTATTTTTAATTATTACTCCCACTACATTAATTTTATCCTCAAAAACATTCTTTCAACAACCGACTCTCTTCAATTCGCTGTGGCTAATTCAGCTAACACTTAAACAAATAATATTAACCCACACACTAAAGGAGCGAACCTGATCCCATATACTCTAGCCTAATTTCTTTATTGCCTAAATAACATTCTCGGACTTACACCATATGCATTTACACCAACCACTACAACTGATGTCAATAAATCTAGGCATAGCTATTCCTCTATGAGCAGCAGCTGTACTAATAGGCCTCCGATTTAAAACAAAATCATCCCTCGCTCATTTTTTACCACAAGGAACACCAATTCCACTAATCCCTATATTAATTATTATTGAAACAATTAGTCTATTCATTCAACCTGTAGCCTTAACCGTACGATTAACAGCCAATATTGCAGCAGGTCACCTATTATGCATTTACTTGGGGATACAACATTAACTCTTCTATCAATTTACCTCACTTCCCAATCACTATCATTATTATTATTTTATTAATTACCCTAGAGTTGGGTGTAAGCCTTAATTCAAGCTATGTATTTACTCTCTTAGTAAGCCTGTACTTACATGATAATTCATAATGACTCACCAAACCCATGCTTATCACATAGTCAACCCAAGCCCTTGACCACTAACAGGAGCATTATCAGCTTTCTTCTCACATCCGTAGCCCTAGTTATGATTCCACTTTTACCACACACTGCTTCTCACTGGAGTCTACTAGCTAGTTCTATAACAATATTTCAATGATGACATTGATGTAGTACAGTACATATCAAGGCCACCATACCTGTCCAAAAAGGCCTACGATACGGAATAATTCTATTTATTATTTCAGAAATTTTCTTCTTTGCAGGTTTCTTCTGAGCATTTTATCATTTACAGTTCCAGCCCCAACTCACAAACAGGGGGACTATGACCACCTACAGGCATTACTCCCCTCAAGCCCAATAAGAAGTCCCTCTCTTAAATACAACCGTACTACTAGCATCCAGGAGTTACAATTACATGAGCACATCACAGCCTCATAGAAGCTAACCGAAAAGAATCAACCCAAGCACTACTCCTAACCATTATATTGAGATCTACTTCACCTGCCTACAATTATCAGAATATTCTGAAGCCCATTTATCTCCGACGGAGTATATGAATCCACATTTTTGTAATTTCACAGGCTTTCATGGCCTTCGTAGTATCAGGACCACTTTCCTCTACCTGTTACTTTCGCCAACAATTATATCACTTCACATCTAGCCACCATTTCCGGCTTCAAACCTTCATGATATTGACATTTCGTAGATGTGAGTATGTTTCCTCTATATTTCCATCTATTGATGAGGCTCTTACTCTTAGTATAAAAGTATTATTGACTTCAATCAACGAGGCCTCGAATGATTCGAAGAGTATTATAGAATTTTAATTTTTAGCCCTATTGACTAACATTACTTTGGCCTTACTTCTTATCACAATTACATTTTGACTTCCACAATTAAATATTCCTGCAGAAAAGCACAACCCTTACGAGTGCGGATTTGATCCTACAACCTCGCACACTTACCATTCTCAAATTTTCCTAATCGCCATCACATTTCTCTATTTGATCTGGAAATTGCCCTATACCTGTATTATTAAATAAATAATTTAACTCTAACAATAAATATAATTTTACCCTACTTATTATTCTGGCTTTAGGGTTATACGAAAGTGGTCCAAAGTTAGATTGGGCTGAATTGGTATATAGTTTAATTAAAACCAATGATTTCGACTCATTAGATTATGAAAGCTCATATTTGCAAATATGCCTTTATCTATATTAATCATAATACTAACATTTCATCATTATTAGGGTTATTAAATTTATCGATCTCACCTAATATCATCACTGCTATGTTTGGAAGGCATAATATTATCATTATTTATCATAATTACACTCACAACTTTCAATATGCACTTCATATTAATGTATATGATACCCCTCATTCTCAAATTACGCGCCGCATGCGAAGCTGCAGTAGGCCTAGCCTTGTTAATTTTAGTCTCCAACCTATATATGGCCTAGTTATGTATAAAACCTAAGCTTACTCCAATGTTAAAATTTTTTCAACCATCATAATACTTCCCACCATATGAAGCAAAAAATTATATAATATGAATCAACACAATAATCTGTAGTCTACTAATCAGTATATACGCCCTCATATTATCCCTTACCAAACAACTCATGCGACCTAATATCTTTGATTTTTCACTCAGATTCACTAACATCACCACTTCTTATATTAATATTACGACTGCCTTACTAATAATTTAGCAACACAACAGCATTTATATATCGCCCACCCACGAAAAAAACTATATATCTCAATATTAATTCTATTACAATTTACTTATTATACTTTTTCCTTCGAACTAATTTTATTTTATATTCTATTTGAAACTACCTAATCCCCACCCTAATTATTATTACCCGTTGAGGGTATCAACCAGAACGCCTTAATGCTGGTTCATATTTCTATTCTACACACTAGGATCCTTACCTCTATTAGTACCCTCCTATACTATCTAAATACTTTACAGGATCCTAAGCATACCATAATTATTAATTCTAGCAAAATACTTATTCATGAACCAATAGTATTGGGATGTATATTGCTTTTATAGTCAAATACCCCTATATGGGTTACCTATGACTCCAAAAGCTCACGTAGAAGCCCCCATCGCTGGCTCAATAGTACTTGCAGCAATCTTACTAAAACTAGGGGTATGGTATAGTGCAATTACTCTATCCTCAATCCACTAATAGAAAAATAAGCTACCCTTTTATCATTCTATCCCTATGAGGATAGTGATAATATGCTCCATCTGCTACGACAAGCCGACCTAAAATCACTTATCGCTTACTCCTCAAATCTACATAGCACTTATTTTAGCTATTATAATTCAAGCCCCATGAAGCCTTACCATATTACAATAATACTAATAATCTCCCATGGACTTACCTCATCCTGCTATTTTGTTTAGCAAATACTAACTACGAACGAATTCACAGCCGAACTATAATGTTACCTGAGAAAGCCTTCAAACATTATTTCCCCTTCACAACTTTTTGGTATTTCCCCAGCTAACCTAGCAAATTTAGCTCTCCCCCAACTAATCGGCTCTAATAGGTGAACAACTATTAACAATCTGCTTCTTTCTCTTGATCTAATTTTACCATCATATTTACGGAACAGCATGTTAATTACAGCCCTCTACTCACTTCACACATATTTACCTCAACACAACGGGGGCCATTAACGTACAGCTTTAGCAGCATCAAGCCCCTCTTTACACGAGAAAATGTACTAATAATAATACATTTAATACCCATCTACTATTAACTACAAAACCCCAAGATAATTATAGATTTGACGCCTAGTTATGGTTAATAAAAACGATAGATTGTGGGCCTAATGATAAGCCCCGCTTTACTTGCTACCGGTATGCAAAGACGCTACAATTAA

>d4f39517-39f0-42d8-9f31-85705358187a

CCTCATGTACTTCGTTCAGTTACGTATTGCTAAGCCTCCTAATTCGAACAGAACTAGGACAGCGACCTAATAGAAGACGACCATGTATACAATGTTATTGTCACCGCCCACGCATTTCATAATTTTCTTAGTAATACCAATTATGATTGGGGGTTTTGGGAACTTGACTTATTCCTTTAATAATTGGCGCTCCCGATATAGCATTTCCTCGAATAAATAACATAAACTTCTGACTTCTACCACCCTCCCCACTACTCCTACTGCTTGCATCATCAACTTTAGAAGCCGGTGCCGGCACTGGTTGGACAGTCTACCCACCCTAGCGAACCGTTATCCCCACCCAGGAGCCTCTGTAGATTTAACCATTTTTCACGTTTAGCGGGCATTTCTTCTATTCTTGGGTATTAACTTTATTACAACAATTCGCCAAATATGAAACCGCCAGCCACTGACAATGTCGACTCCGTTGTTTGTGTGATCCGTCTAATTACTGCAGTCCTTT

>57f6c795-a5c1-4ad1-a532-78fcb7d163ac

CCTTACTTGTTCAGTTACGTATTCTCTAAGCCTCCTAATTCGAACAGAACTAGGACAACCCGAGCCTAATGAAGACGACCATGTATACAATGTTAATGCGCTACGCGTTAATCTGCTGTTTTTTTTTCATAGTAATACCAATTATGATTGGGGTTTAAGGAACTGACTTATTCCTTTAATAATTGGCCTCTCCCGATATAGCATTTCCTCGAATAAATAACATAAGCTTCTGACTTCTACCACCCTCCCTACTCCTACCTTGCATCATCAACTGAGAAGCCGGTGCCGGCACTGGTTGGATGATCCTACCCACTAGCAGGCAATTTATCCACCAGGAGCCTCTGTAGATTTAACCATTTTTTCACTACATTAGCAGGCATTTCTTCTATTCTTGGAGCTATTAACTTTATTACAACAATTGTAAATATGAAACCGCCAGCCATGACTCAATATCAAACTCCGTTGGCAATACGTAAC

>f2224368-16c5-4c81-bf51-7ba6ea15f288

TTGTACTTCGCGTTCAGTTGCATGTGCTGTTATTTATTCGAGAAATGCTATATCCCGGAGCGCCAATTATTTAAAAAAAGGAATAAGTCAGTTCCCAAAACCCTATAATTATTACTATGAAAAAAATTATGATGAATGCGTGGGCGGTGACAATAACATTGTATACATGGTCGTCTTCTATTAGGCTTCCGAGTTCCTAGTTCTGTTCGAATTAGGAGGCTTGGCAATACGTAA

>95830065-c26f-4544-8827-09fd5f3e69e6

AGTAATAACTACGTTCAGATTTTATTGCTTCCTAATTCGAACAGAACTAGGACAACGGAAGCCTAATAGAAGACGACCATGTATACAATGTTATTGTCACCGCCCACGCATTCATCATAATTTTTTCATAGTAATACCAATTATGATTGGGGGTTTTAAGAACTGACTTATTCCTTTAATAATTAGTGATATAGCATTTCCTCGAATAAATAACATAAGCTTCTGACTTCTACCACCCTCCCTACTCCTACTGCTTGCATCATCAACTTTAGAAGCGGTGCCGGCACTGGTTGGACAGTCTACCCACCCCTAGCAGGCAATTTATCCCACCAGGGCCTCTGTAGATTTAACCATTTTTTCACTACATTGTAGGCATTTCTTCTATTCTTGGAGCTATTAGCTTTATTGCAATA

>670e4330-ff63-4e44-aaec-ab9acf4a31c8

TTGTACTTCGTTCTTGGTGCAATGACTGCTAATTCGAACAGAACTAGGACAGCCCGGAAGCCTAATAGGGCGACCATAATATGCAATGTTATTGTCACCGCCCACGCATTCATCATAATTTTTTTTCTGTGTGTATAATTATGATTGGGGGTTTTGGGAACGACTTATTCCTTTAATAATTGGCGCTCCCGATATAGCATTTCCTCGAATAAATAACATAAGCTTCTGACTTCACCACCCTCTACTCTACCAGCATCATCAACTTTAGAAGCCGGTGCCGGCACTGGTTGGACAGTCTACCCACCCCTATTGGGCAATTTATCCCACCCAGGAGCCTCTACTAATTTAACCATTTTCACTACATTTGTAGGCATTTTCTTCTATTCTTGGAGCTATTAACTTTATTACAACAATTGTAA

>beddd69c-7d72-4102-8d1c-19af7191cce9

GATGTACTTCGTTCAGTTACGTATTGCTATGCCTAGGTTATTGACAGTTGAATTGGTTGGTGTAAATGCATATGGTGTAAGTCCGAGAATGTTATTTGAGGCCAATAAAGAAATTAGGGCTAGGAGTATAAGGATCGGATTCGCCCTTTAAGTGGTGTGGGTTAATATTATTTGTTTAAGTGTTAGTTGAATTAGCCATTGTTGAATTGAAGAGAGTCGGTTGTTGAATAAGTTTTGGAGGATAAAATTAATGTAGTGAAGTGCAATAATTAAAAATACTAAAAATTTACTCTAGTATTGTTGGAATATTGAATAGAGGCAAATAGATTTTAGTTTCATTTTAGTTCTCAAGTTGTTTTATGTTTTTGTGTTTCTACTAATTTTGGTAGTGGGTAATAATGGAAAGTAAAGTTCAGTATTTTCAATTGTATAATATAAAATAAAGGTAACAATTATAGATATAATTACTATTGGTCACGGTGAAATATTTAGTTGAGGCATTCACTATAGAGAGTTTGTTCTCTCAATCTTTAACTTAAAAGGTTAATGCTAAGTTAGCTTTACGGTGATACAATATATAAGTATGAAGCTCATACTTCGAAATCTTAAAATAAATGATTCTAGAACAATAGGTATAAAGCTGTGATTGGACCCGCAAATTTCCGAGCATTGTCCATAAAATAGGCCTGGTCGTATAGAAGCTAGTATGGCTTGGTTTAAACGTCCAGGAATTGCATCTGTTTTTACGCCTAGTGATGGTACGGCTCATGAGTGTAAGACGTCTTGTGATGAGATTAATATGCGAATATCCGCTTCTATAGGTAAAGTTGTTCGGTTATCAACTTCGAGGAGTCGAAATTCCCCTGGCTCAAGGAAATGTTGGCATAATGTAGAGTCAAATACTAAGTCTTCATAGTCGGAATATTCATAGGTTCAGTATCATTGGTGACCAATTGCTTTAGGGTTAAATAAGGTTTATTAAATTCGTCTGTCATATATAGAATACGCAGTGATGGGAGGGCAATTGTATTAGAATCAGTGCAGGGAGAATGGTTCAGATTATTTCGATCTCTTGAGCATTTATGGTGCTAGTATGAGTGAGTTTTGTGGTAAGTATAAGGAAATAATATAAGACTAAGGAACTAATCAGGAAAATAATTATGAGTGCATGGTCGTGGAAAGCAATAAGTTCTTCTATGATAGGTGATGTGGCATTTTGTAAACCTAGTTGAGCTGGTGTTGCTATTAAGATATATAGATATTTAGTCTATAATTTAACTTTGACAAAGTTGTATGTAATTATTTTACTAATATCTTATTGAAAGAATCATAGGGTATATGGGATTGGCTTGAAACCAATTTTGGGGTTCAAATCCTTCCTTTTTCGTCTAGGATTTAACGTAAGTTGCCTCTTCGAATGTGTGGTAAGGAGGAGGGCAGCCGTATAATCATTCTAGATTGGTGGATAGTTGCTCAATGGCTAAGACTTTTCGTTTTGAAGAGAAAGCTTCTCAAATTATAAAAACCATCAGGACTACTGCTGTAAATTAAAATGAACCGATTAGATGAGACGATATTTCATGTGGTATATGCGTCTGGATAGTCTGAGTATCGTCGAGGTATTCGAATAAGCAAGGAAGTGTTGTGGGAAGAAAGTTAGAAGTTACGCCGACAAATATAATGGTGAAGTGGATTTTGGCATATGTTTGGTCAAAGGTATGGCCTGAAAAGAGCGGGAATCAGTGAATAAATCCCCCTATAATGGCAAATACTGCTCCTATTGATAACACATAGTGAAAGTGGGCTACTACATAGTATGTATCATGTAATACAATATCTAATGATGAGTTAGCTAACACAATTCCTGTTAGTCCACCCACGGTAAAAGAAAAATAAAGCCCAGGGCTCATAATATTGCGGGAGATCATTTGATATTACCGCCGTGCGGTGTGGCTAATCAGCTAAATACTTTTACTCCAGTGGGAATGGCAATGATTATGGTAGCTTTGATGTAAAATATGCACAGTATCTACATCTATTCCTACTGTGAATATGTGATGGGCTCATACAATAAAGCCTAGGAAACCAATAGATATTATGGCTCATACCATGCCCATATAACCAAGGAGTTCTTTTTATTAGAATAATATGTTACAATGTGTGGTATTCCAAAACCCAGTAAGATGAGAATATATACTTCAGGGTGACCGAAGAATCAGAATAAGTGTTGGTATAGGATAGGGTCGCCGCCACCAGCAGGATCAAAAGTAGTATTTAAATTACGGTCAGTTAATAGTATGGTAATCCCTGCAGCTAGAACTGGAAGAGAAAGTAAGAAGAGCTGCAGTAATTAGGACGGATCACACAAACAACGGAGTTTGATGTTGAGTCATGGCTGGCGGTTTCATATTTACAATTGTTGTAATAAAGTTAATAGCTCCAGAATAGAAGAAATGCCTGCTAATGTAGTGAAAAAATGGTTAAATCTACAGAGGCTCCTGGGTGGGATAAATTGCCTGCTAGGGGTGGGTAGACTGTCCAACCGGTGCGGCACCGGCTTCTAAAGTTGATGATGCAAGCAGTAGGAGTAGGGAGGGTGGTAGAAGTCAAACATGTTATTTATTCGAGGAAATGCTATATCGGGAGCGCCAATTATTAAAGGAATAAGTCAGTTCCCAAAACCCCCAATCATAATTGGTATTACTATGAAAAAAATTATGATGAATGCGTGGGCGGTGACAATAACATTGTATACATGGTCGTCTTCTATTAGGCTTCCGGAGTTGTCCTAGTTCTGTTCGAATTAGAA

>8d48935b-6c11-4e8b-976b-ee13e1a93475

TGTACTTCGTTCAGTTACACATTGCTGGTAGAAGTCAAGAAGCATGTTATTTATTCGAGGGAAATGCTATATCGGGAGCGCCAATTATTAAAGATGAAATAGAGATCAGTTCCCAAAACCCCCAATCATAATTGGTATTACTATGAAAAAAATTATGATGAATCATTGGGCGGTGACAATAACATTGTATACATGGTCGTCTTCTATTAGGCCGGGTTGTCCTAGTTCTGTTCGAATTAGAACAGGACTGCTGCCCCGCCCGTGGTGACTTGTTAGTGTCATTCCCGGGCAAGCGGTGACAACCTTGTGTGTCAAATCCGCGATTCCTTGCTGGAACTCAACGGCGTTTGGTGCCATCGGAAAGGTGAATCAACGCGATGTTGTGGACTTCTGAAGGAGTCGTTCGCTTGCAATATTTCCTTGCACGTTTCAAAGCTGCAATGGCTTTTTCATCGTTCTTGCCCGCAACTTTGCCGGAATAGCCCGGCTTCCACGTTGTACATCAAGATGATAGCCTTTGCATTCAATAAGAATGTTGTTCAAAACCATCGAACGTGTATTGCCGAGATAATATGTCGGTTGCGAATAACACCGTGCAGCAATACGTAATT

>47f1a1d0-f920-4829-baf5-f429f0dd51b5

TTGATGTACTTCGGTTCGTTGTATGCTATTCGAGACAGACTGGACAACCCGGAAGCCCTAATAGAAGACGACCATGTATCTAATGTTATTGTCACCGCCCGCATTCATCATGTTTTTTTCATAGTAATACCAATTATGATTGGGGGTTTGGGAACTGACTTATTCCTTTAATAATTGGCGCTCCCGATATAGCATTCCTCGAATAAATAACAACTGTTTCTGCCTCCTCCTACATGCACATCGTCAACTTTAGAAGCCGGTACAGCACTGGTTGGACAAGTCTCCCACCCCTAGCAGGCAATTTGTCGCAGGAGCCTCTGTAGATTTAACCATTTTTCACTACGATGACAGCAGGCATTTCTTCTATTCTTGGAGCTATTAACTTTGTCACGCGCAATTGTAAATATGAAACCGCCAGCCATGACTCAATGTCAACTCCCGTTGTTTGTGTGATCCGTCCTAATTACTGCAGTCCTTCTTTATATCTTCAGTTCTAGCTGCAGGGATTACCATACTGATAACTGACCGTAATTTAAATACTTCCTTTCTTTGATCCTGCTGGTGTGACCTATCCTATACCAACACTTATTCTGATTCTTCGGTCACCTAGTATATATTCTAATCTTACCGGGTTTTGGAATCATTTCACACATTGTAACATATTATTCTATTAAAAAGAACCCTTTGGTTATATGGGCATGGTATGAGCCATAATATCTATTGGTTTCTAGGCTTTATTGTATGAACTTATCACATATTCACAGTAGGAATAAGATGAAAAATACTCGTGCATATTTTACATCAGCTACTATAGTATTTACTATTATAGATACAAAAGTATTTAGCTGATTAGCCATATACAATGATATGCATCAAATGATCTCCACCAATATTACAGGCCCCTGAAGCACATTTTTATACTTTACCAGTGAGGTGGATTCTGGAATTGTGTTAGCTAACTCATCATTAGATATTGTATTACATGATACATACTATATGGTAACCCACTTTCACTATGTGTTATCTTGAACCAGTATTTTGCCCATTATAGAGTTTATTCACTGATTTAACTCTTTTAGACCTATACTCTAATGAAATAGCTGCCCAAAATCCGCTACCATTATCTTTATCGACGTAATTCTTTCTTCCCACAACACTTCCTTGGCTTATCCGGAATACCTCGACGATACTCAGACCTCGCAGACGCATATACTACATGAAATATCGTCTCATCTATCAGTTCATTTCCCAA

>b12a5ff7-33aa-44d4-8ece-797d2026725f

AAACGTACTTCGTTCAGTTACGTAATGCTAGGACAACCCGGAAGCCTAATAGAAGACGACCATGTATACAATGTTATTGTCACCGCCCACGCATTCATCATAATTTTTTTCACTTAGTAATACCAATTATGATTGGGGGTTTTGGGAACTGACTTATTCCTTTAATAATTGGCGCTCCCGATATAGCATTTCCTCGAATAAATAACATAGCTTCTGACTTCCTCCACCCTCCCTACTCCTACTGCTTGCATCATCAACTTTAGAAGCCGGTGCCGGCACTGGTTGGACAGTCTACCCCACCCCGCGGGCAGTTATCCCACCCAGGAGCCTCTGTGGGTTAACCACATTTTTTCACTGGCAGCAGGCATTTCTTTATTCTTGGAGCTATTAACTTTATTACAACAATTATA

>077a160c-b04f-49a3-be6a-ef50de7674ac

TTTTCGCTTCGTTCAGTTACGTATTGCTGTTAGTCACCCACGGTAAAAGAAAAATAAAGCCCAGGGCTCGCAATATTGCGGGAATCATTTGATATTACCGCCGTGCAGTCAATATTTAATCAGCTAAATACTTTTTACTCCAGTGGGAATAGCAATGATTATGGTAGCTGATGTAAAATATGCACGATGTCTACATCTATTCTACTGTGAATATGTGATGGGCTCATACAATAAAGCCTAAGGAAACCAATGAATATTATGGCTCTTTATTGCCCTTATAACCAAAGTTCTTTTTTATTAGAATAATATGTTACAATGTGTGAAATTATTCAAAACATTAAGATTAGAATATATGCATCAGGTGACCGAAGAATCAAGAATAAGTGTTGGTATAGGATAGGGTCGCCGCCACCAGCGGGATCAAAGAAGTAGTATTTAAATTACGGTCAGTTAATAGTATGGTAATCCCTGCAGCTAGAACTGGAAGAGAAAGTAAAAGAAGGACTATGATAATTAGGACGGATCACACAAACAACGGAGTTTGATATTGAGTCATGGCTGGCGGTTTCATATTTACAATTGTTGTAATAAAGTTAATAGCTCCAGAATAGAAGAAATGCCTGCTAAATGTGGTGAAAAAATGGTTAAATCTACAGAGGCTCCTGGGTGGGATGAATTGCACTAGGGGTGGGTAGACTGTCCAGCCAGTGCCGGCACCGGCTTCTAAAGTTGATGCAAGCAGTAGGAGTAGGGAGGGTGGTAGAAGTCAGAAGCTTATGTTATTTATTCGGAAATGCTATATCGGGAGCGCCAATTATTAAAGGAATAAGTCAGTTCCAAAACCCCAATCATAATTGGTATTACTATGAAAAAAATTATGATGAATGCGTGGGCGGTGACAATAACATTGTATACATGGTCGTCTTCTATTAGGCTTCAGGTTTGTCCTATCAATACGTAACTTCT

>f0a3bd57-80cf-4eea-9f5e-4f69d0356cb6

TTGTACTTCGTTCAGTTACGTATTGCTGACAACCCGGAAGCCTAATAGAAAGACGACCATGTACAATGTTATTGTCACCGCCCACGCATTCATCATAATTTTTTCATAGTAATACCAATTATGATTGGGGGTTTTAGAGACCCAATGACTTATTCCTTTAATAATTGGCGCTCCCGATATAGCATTTCCTCGAATAAATAACCTTAAGCTTCTGACTTCTACCACCCTCCACTACTCCTACTGCTTGCATCATCAACTTTAGAAGCCGGTACCGGCACTGGTTGGACGATCTACCCACCCCTAGCAGGCAATTTATCCCACCCAGGAGCCTCTGTGGATTGCCATTTTCTACATTTAGCAGGCATTTCTTCTAA

>f503e3f5-88b7-4d40-a682-0cdca68232f1

TTGTACTTCGTTCAGTTACATGCTATATCTGTAAGGGTTCTTTTTTATTAGAATAATATGTTACAATGTGTGAAATTATTCCAAAACCCGGTAAAGATTAGAATATATACTTCAGGGTGACCGAAGAATCAGAATAAGTGTTGGTATAGGATGAGGGTCCCAGCCGCCACCGGTGGGATCAAAGAAAGTAGTATTTAAATTACGGTCAGTTAATAGTATGGTAATCCCTGCAGCTAGAACTGGAAGAAAGTAAAAGAAAGGACTGCAGTAATTAGGACGGATCACACAAACAACGGAGTTTGATATTAGTCATGGCTGGCGGTTTCGCATATTTACAATTGTTGTAATAAAGTTAATAGCTCCAAGAATAGAAGAAATGCCTGCTAAATGTAGTGAAAAAATGGTTAAATCTACAGAGGCTCCTGGGTGGGATAAATTGCCTGCTAGGGGTGGGTAGACTGTCCAACCAGTGCCGGCACCAACCTAAAGTTGATGATGCAAGCAGTAGGAGTAGGGAGGGTGGTAGAAGTCAGAAGCTTATGTTATTTATTCGAAATGCTATATCGGGACGCCAATTATTAAGGAATAAGTCAGTTCCCCAAAACCCCCAATCATAATTGGTATTACTATGAAAAAAATTATGATGATCATTGGGCAGTGACAATAACATTGTATACATGGTCGTCTTCTATTAGGCTTCCGGGTTGTCTAAGCAATACGTAGCTTG

>22f9d724-09ac-4b9f-a3c2-75a1bfccafca

ATTGTACTTCGTTCGTTACCGTATTGCTAAATGTGAAGTGAAAAATGGTTTAAATCTACAGAAGCTCCTGGGTGGGATAAATTGCCTGCTAGGGGTGGGTAGACTGTCCAACCAGTGCCGGCACCATCTAAGTTGATGGTAGCAGTAGGAGTAGGGAGGGTGGTAGAAGTCAGAAGCTTATGTTGTTGTTAGAAATGCTATATCGGGAGCGCCAATTATTAAAGGGAATAAGTCAGTTCCCAAAACCCCCAATCATAATTGGTATTACTATGAAAAAATTATGATGAATGCGTGGGCGGTGACAATAACATTGTATACATGGTCGTCTTCTATTAGGCTTCCGA

>037e1a2c-7daa-4091-a682-c1b532e9ebfc

TGTTATTACTTCGTTCAGTTACATTATTGCTAATAGATATTATGGCTCATACCATGCCCATATAACAAAAGGGATCTTTTTATTAGAATAATATGTTACAATGTGTGAAATTATTCCAAAACCCGGTAAGATTAGAATATATACTTCAGGGTGACCGAAGAATCAGAATAGATGTTGGTATAGGATAGGGTCGCCGCCACCAGCAGGATCAAGAAAGTATTATTTAAATTACGGTCAGTTAATAGTATGGTAATCCCTGCAGCTAGAACTGGAAGAAAGTAAAAGAAGGACTGCAGTAATTAGGACGGATCACACAAACAACGGAGTTTGATATTGAGTCATGGCTGGCGGTTTCATATTTACAATTGTTGTAATAAAGTTAATAGCTCCAGAATAGAAGAAATGCCTGCTAAATGTAGTGAAAAAATGGTTAAATCTACAGAGGCTCTGGGTGGGATAAATTGCCTGCTAGGGTGGGTAGACTGTCCAACCAGTGCCGGCACCGGCTTCTAAAGTTGATGATGCAAGCAGTAGGAGTAGGGAGGGTGGTAGAAGTCAGAAGCTTATGTTATTTATTCGAGGAAATGCTATATCGGGAGCGCCAATTATTAAAGGAATAAGTCGGTTCCCAAAACCCCCAATCATAATTGGTATTACTATGAAAAAAATTATGATGAATGCGTGGGCAGTGACAATAACATTGTATACATGGTCGTCTTCTATTAGGCTT

>348ef245-d7f5-4ccd-af78-a5cb69e79f29

AGTATGCTTCGTTCAGTTACGTATTGCTGTAGTGAAAAATGGTTAAATCTACGAAGGCTCCTGGTGGGATAAATTGCCTGCTAGGGGTGGGTAGACTGTCCAACCAGTGCCGGCACCGGCTACTAAAGTTGATGATGCAAGCAGTAGGAGTAGGGAGGGTGGTAGAAATTAGAAGCTTATGTTATTTATTCGAGGAAATGCTATATCAGGAGCGCCAATTATTAAAGGAATAAGTCAGCTACAAACCCCAATCATAATTGGTATTACTATGAAAAATTATGATGAATGCGTGGGCGGTGACAATAACATTGTATACATGGTCTCGTCTTCTATTAGCAATACGTAATAACA

>b866e4fe-e630-4837-addc-4ec4ba9cf0dd

AGTATCCTTCGTTCAGTTACGTATTGCTATAGTTGCTCAATGGCTTTAAGACTTTTCGTTTTGAAGAAAGCTTCTCAAATTATAAAAACCATCCAACTGCTGCTGTAAGTGAAATAAATGAACCGATAGATGAGACGATATTTCATGTAGTATATGCGTCTGGATAGTCTGAGGTATCGTCGGGATATTTCCGGATAAGCCAAGGAGGTGTTGTGGGAAGAAAGTTAAACGCCGACAAATATAATGGTGAAGTGGATTTTGGCATATGTTTGGTCAAAGAGTATAGCCTGAAAAAGAGCGAAATCAGTGAATAAATCCCTATAATGGCAAATACTGCTCCTATTGATAACACATAGTGAAAGTGGGCTACTACATAGTATGTATCATGTAATACAATATCTAATGATGAGTTAGCTAACACAATTCCTGTTAGTCCACCCACGGTAAAAGAAAAATAAAACCCAGAGCTCATAATATTGCGGGGAGATCATTTGATATTACCGCCGTGCAGTGTAATCAGCTAAATACTTTTACTCAGTGGGAATAGCAATGATTATGGTAGCTGATGTAAAATATGCACGAGTATCTACATCTATTCCTACTGTGAATATGTGATGGGCTCATACAATAAAGCCTAGAAACCAATAGATATTATGGCTCATACCATGCCCATATAACCAAAGGTTCTTTTTTTATTAGAATAATATGTTACAATGTGTGAAATTATTCCAAAACCCGGTAAGATTAGAATATATACTTCAGAATTTGACCGAAGAATCAGAATAAGTGTTGGTATAGGATAAGGTCCTTGCCACCAGCAGGATCAGAGAAGTAGTATTTAAATTACGGTCAGTTAATAGTATGGTAATCCCTGCAGCTAGAACTGGAGAGAAAGTAAAAGAAGGACTGCAGTAATTAGGACGGATCACACAAACAACGGAGTTTGATATTGAGTCATGGCTGGCGGTTTCATATTTACAATTGTTGTAGCAAAGTTAATAGCTCCAGAATAGAAGAAATGCCTGCTAAATGTAGTGAAAAAATGGTTAAATCTACAGAGGCTCTAGAACAGGATAAATTGCCTGCTAGGGGTGGGTAGACTGTCCAGCCAGTGCCGGCACCGGCTTCTAAAGTTGATGATGCAAGCAGTAGGAGTAGGGAGGGTGGTAGAAGTCAGAAGCTTATGTTATTCGAGAATGCTATATCGGGAGCGCCAATTATTAAAGGAATAAGTCAGTTCCCAAAACCCCCAATCATAATTGGTATTACTATGAAAAAAATTATGATGAATGCGTGGGCGGTGACAATAACATTGTATACATGGTCGTCTTCTATTAGCAATACGTAACTTCG

>76c765aa-c232-4bcf-8511-91fafdebfff5

GTTGTACTTCGTTCAGTTACGTATTGTAATAGAAGACGACCATGTATACAATGTTATTGTCACCGCCCACGCATTCATCATAATTTTTTTCATAGTAATACCAATTATGATTGGGGTTTTGGGAACTGACTTATTCCTTTAATAATTGGCGCTCCCGATATAGCATTTCCTCGAATAAATAACATAAGCTTCTGACTTCTACCACCCTCTCTCCTACTGCTTGCATCATCAACTTTAGAAGCCGGTGCGGCACTGGTTGGACAGTCACCCACCTAACCAGGCAATTTATCACCAGGAGCCTCTGTAGATTTAACCATTTTTTCACTACATTTAGCAGGCATTTCTTCTATTCTTGAGACTATTAACTTTATTACAACAATTGTAAATATGAAACCGCCAGCCATGACTCAATATCAAACTCCGTTGTTTGTGTGATCCGTCTAATTACACCGCAGTCCTTCTTTTACTTTCGCTTCCAGTTCTAA

>865d2855-5f8d-4a09-b02e-238e3d1b914b

GATAATACTTCGTTCAGTTACGTATTGCTAGAAGAAATGCCTGCTAAAATGTAGTGAAAAATGGTTAAATCTACAGAAAGGCTCTAGATAAGGATAAATTGCCTGCTAGGGTGGGTAGACTGTCAACCAGTGCCGGCACCGACTTCTAAAGTTGATGATGCAAGCAGTAGGAGTAGGAGGTGGTAGAAGTCAGAAGCATGTTTATTCGAGGAAATGCTATATCGGGAGCGCCAATTATTAAAGGAATAAGTCAGTTCCCAAAACCCCAATCATAATTGGGTATTACTATGAAAAAGGTATGATGAATGCGTGAGCGGTGACAATAACATTGTATACATGGTCATCTTCTATTGGAAG

>7fe117f6-8a74-471b-8ffe-d1d2e9afbbec

ATTACTTCGTTCAGTTACGTATTGTAGAAGACGACCATGTATACAATGTTATTGTCACCGCCCACGCGATTCATCGCAATTTTTTCATAGTAATACCAATTATGATTGGGGTTTTTGAACTGACTTATTCCTTTAACTAATTGGCGCTCCCGATATAGCATTTCCTCGAATAAATAACATAGCTTCTGACTTCTACCACCCTCCCTACTCCTACTGCTTGCATCATCAACTTTAGAAGCCGGTGCCACTGGTTGGACAGTCTACCCACCCCTAGCAGACAGTATCCCCACCAGGAGCCTCTGTAGATTTAACCATTTTTTTCTTTGTTTGTGAGCATTTTCTTCTATTCTTGAGCTATTAACTTTATTTAACAATTGTAAATATGAAACCGCCATGTGACTCAATATCAAACTCCGTTGTTTGTGTGATCCGTCTAATTACTGCAGTCTTCCTTCTTTACTTTCTCTTTGATTTT

>b95de8a4-9d61-413a-bc94-871ee7eb5ecd

ATTGTACTTCGTTCAGTTACGTATTAATGGTTAAATCTACAGAGGCTCCTAGATTGGGATAAATTGCTAGGGGTGGGTAGACTGTCCAACCAGTGCCGGCACCGGCTTCTAAAGTTGATGATGCAAGCAGTAGGAGTAGGGGTGAGTAGAAGTCAGAAGCTTATGTTATTTATTGAGGAAATGCTATATCGGGAGCGCCAATTATTAAAAAGGAATAAGTCAGTTCCCAAAACCCCCAATCATAATTGGTATTACTATGAAAAAATTATGATGAATGCGTGAAGCGAGTGACAATTAACATTGTATACATGGTCGTCTTCTAATGGAAG

>499c5a7c-257c-48db-b4a1-43994580fa2a

GATTGTACTTCGTTCAGTTACGTATTGCTATGTATACAATGTTGTCACCGCCCACGCATTCATCTTAATTTTTTTTCATAGTAATACCAATTATGATTGGGGGTTTTGGGAACTGACTTATTCCTTTGCGTAAATTGGCGCTCCCGATATAGCATTTCCTCGAATAAATAACATAAGCTTCTGACTTCTGCCACCTCCCCTACTCCTACTGCTTGCATCATCAGCACAGAAGCCGGTGCCGGCGCTGGTTGGACAGTCCTACCCACCCTAGCAGGCAATTTATCACCAGGGCCTCTGTAGATTTAACCATTTTTTCACTACATTTAGCAGGCATTTTCTTCTATTCTTGGAGCTATTAACTGTGCAACAATTGTAATGGTTAATAGCTCAAATAGAAGAATGCCTGTAAATATGAACGTGGTTAAATTACGGAGACTCATAGGTAGATATAAATTGCCTGCTGAGTGGGTGAGGCTGTCCATTGGGAACAACATAAAATACCATCTAAAATTGATGACAAGCGGTAGAAATTTATGTGTTTATTCGATGAAATTATATCGGGAGTGTGAGTGATAAGTCCTACAAACCCAATTGATGTTGCTATGAAAAATTATGATGAATGCGTGTTTTTAATGGCGGTAACATGCATGGCAATACGTAAAG

>fffd3b52-232f-43bf-989f-b7dd08de5f7c

AGTAATACGCTACGTTCGTTACGTATTGCTATATTTACAATTGTTGTAATAAAGTTAATAGCTCAAGAATAGAAGAAATGCTGCCACATAAATGTAATTGAAAAAATGGTTAAATCTACAGAGGCTCCTGGGTGGGTTAAATTGCCTGCTAGGGGTGGGTAGACTGTCCAACCAGTGCCGGCACCGGCTCTAAAGTTGATGATGCAAGCAGTAGGAGTAGGGAGAGATTGGTAGAGAAATTAAGAGCTTATGTTATTTATTCGAGGAAATGCTATATCAGGAGCGCCAATTATTAAAGAATAAGTCGGTTCCCAAAACCCCCAATCATAATTGGTATTACTATGAAAAAAATTATGATGAATGCGTGGGCAGTGACAATAACATTGTATACATA

>feb807f9-bb4b-469b-b76e-8dc021961c2b

AGTGTACTTCGTTCAATTTACGTATTTTATATAGTAATTGTAGTAACTGATGTATAAATGTGCGTATCTATCTATTCCTGCAACTGAATATGTAATTGGGCTCATACAATAAAGCCTAGGAAACCAATAGATATTATGGCTCATACCATGCCCATATAACCAAAGGGTTCTTTTTATTAGAATAATATGTTACAATGTGAAATTAAAACCCGGTAAGATTAGAATATCTTCCGGGGTGACCGAAGAATCAGAATAAGTGTTGGTATAGATAAGGTCGCCCATACAACGGATCAAAGAAATGTGTTAAATTACACGGATCCCAGTTAATAGTATGGTAATCCCTCTGGCTGAACGGAAGAGAAATGCAAAAGAAAGGACTGCAGTAATTAGGACGGATCCACACAAACAACAAGTTTGATATTGAGTCATGGCTGGCGGTTTCATATTTGGTGTTGTAATAAAGTTAATAGCTCAAGAATAGAAAATGCCTGCTAAATCGTAGTGAAAAAATGGTTAAATCTACGAAGGCTCCTGGGTGCGATAAATTGCCTGCTAGGGGTGGGTAGACTGTCCAACTGGTGCTTGGCACCGGCTATAAAGTTGATGATGCAAGCAGTAGGAGTAGGGAGGGTGAAGGAAGTCAGAAGCTTATGTTATTTATTCGAGGAAATGCTATATGGGAAGCGCCAATTATTTAAAAGAGAATAAGTGGTTAAAACCTGAAATCTTAATTATATTACTATGAAAAAATTATATGATGAATGCGTGAGCAGTAAACAACGCAATTGTATACATTGATTGATGATATTAACAGCCGCGACGAAGAAAGAAAGCG

>da229c55-3a06-4cd6-b7e4-4fd6d942ff7b

AGTATACTTCAATTTCAGTTACGTAATGTATAAAATATGCGAGTATCTACATCTATTCCTATAACAGAATATGTGATGAAGCTCATACAATAAAGCCTAGGAAACCAATAGATATTATGCGGCTCATACATGCCATATAACCGAGGTTCTTTTTATTAGAATAATATGTTACAATATGTGAAATTATTAAAAAACCCGGTAAGATTAGAATATAACCGCTTCAGGGTGACCGAAGAATCAGAATAAGTGTTGGTATAGGATAGGGTCGCCGCCACCAGCAGGATCGAAAGTAGTATTTAAATTACGGTCAGTTAATAGTATGGTAATCCCTGCAGCTAGAACTGGAAGAAAGTAAAGAAAGGACTGCAGTAATTAGGACGGATCACCAAACAACGAGTTGATAAAGATATGGCTGGCGGTTTTCATATTTAATTGTTGTAATAAGGTCGTAGCTCAAGAATAGAAAGAAATGCCTGCTAAATGTAGTAGAAAAAATGGTTAAATCTGGAGGCTCCTGGGTGGGATAAATTGCCTGCTAGGGGTGGGTAGACTGTCCAACCAGTGCCGGCACCGGCTAAAGTTGATGATGCAAGCAGTAGGAAGTAGGGAGGGTGTTAGAAGTCAGAAGCTTATGTTATTTATTCGAGGAAATGCTATATCAGGAGCGCCAATTATTAAAGGGAATAAGTCAGTTCCCAAACCCCAATCTGAAATTGGTATTACTATGAAAAAAATTATGATGAATGGCGTAGGCAGTGACAATAACATTGTGTACGGAAACGTACGTAGCACA

>f99486e4-dae4-4b3a-9c17-2ca3e3d663d4

AATTGTACTTCGTTCAGTTACGTATTGCTATACAATGTTATTGTCACCGCCCACGCATTCATCATAATTTTTTTTTCATAGTAATACCAATTATGATTGGGGGTTTTGGGAACTGACTTATTCCTTTAATAATTAGCGCTCCGATATAGCATTTCCTCGAATAAATAACATAAGCTTCTGACTTCACCACCCTCCCTACTCCTACTGCTTGCATCATCAACTTTAGAAGCCGGTGCCGGCACTGGTTGGACAGTCTACCCACCCCTAGCAGGCAATTTATCCCACCAGGAGCCTCTGTAGATTTAACCATTTTCACTACATTTAGCAGGCATTTCTTCTATTCTTGGAGCTATTAACTTTATTACAACAATTGTAAATATGAAACCGCCAGCCATGACTCAGCCGCAAACTCCGTTGTTTGTGTGATCCGTCCTAATTGCGCAGTCCTTCTTTTACTTTCTCTTCCAGTTCTAGCTGCAGGGATTACCATACTATTAACTGACAAAATTTAAATACTACTTTTCTGTTTGATCCTGCTGGTGGCGGCGGCCCTATCTATACAACACTTATTCACAGTTATGGTCACCTGAGAGTACCATATTCTAATCTGCCAGGTTTGGAATAATTTCACACATTGTAACATATTATTCTAATAAAGAACCCTTTGGTTATATGGGCATGGTATGAGCCATAATATCTATTGGTTTCTAGGCTTTTGTGTAGCAGGCCCTCACATATTCACAGTAGGAATAGATGTAGATACTCGTGCATATTTTACATCAGCTTCTGTAATCATTGCTATTCCCACTGGAGTAAAAGTATTTAGCTGATTAGCCACACTGCACGGCGGTAATATCAAATGATCTCCCGCAATATTGTGAGCCTAGACTGTTTTCTTTTTACCGTGGGTGGACTAACAGGAATTGTGTTAGCTAACTCATCATTAGATATTGTATTACATGATACATACTATGTAGTAGCCACTTTCACTATGTGTTATCAATAGGAGCAGTATTTGCCATTAGAAGTTGTTCACTGATTCCCGCTCTTTTCAGGCTATACTCTTGACCAAACATATGCCAAAATCACTTCAGCAATACGTAAC

>8c612090-42f7-410e-b6bc-443714baf07d

AAATGTACTTCGTTCAGTTACATTATTGCTAATTGCCGGCACCGGCTTCTAAAAGTTGATGATGCAAACGGTAGGAGTAGGGAGGGTGGTAGAAGTCGGGCTTATATTATTATTCGAGAAGCTATATCGAGGGCGCCAATTATTAAAGGGAATAAGTCAGTCCCCAAAACCCCCAATCATAATTGGTATTACTATGAAAAAAATTATGATGAATGCGCGTAAGGCGGTGACAATAACATTG

>e36adedf-44e2-4e34-a11f-fa3e53f214e9

GTTGTACTTCGTTCAGTTACGTATTGCTGTTATTGTCACCGCCCACGCATTCATCATAATTTTTTCATAGTAATACCAATTATGATTGGGGGTTTTGAATTTGACTTATTCCTTTAATATTGGCGCTCCCGATATAGCATTTCCTCGAATAAATAACATAAGCTTCTGACTTCTACCACCCTCCCTACTCCTACTGCTTGCATCATCATCAACTTTAGAAGCCGGTGCCGGCACTGGTTGGACAGTCTACCCACCCCTAGCAGGCAATTTATCACAGAATCTGTAGATTTAACCATTTTTTTCACTACATTTTTCAGGCATTTCTTCTATTCTTGAGGCTATTAACTTTATTACAACAATTGTAAATATGGAAACCGCCAGCCATGACTCAATATCAAACTCCGTTGCAGCTTGTGATCCGTCCTAGTACTGCAGTCCTTCTTTTACTTTCTCTTCCAGTTCTAGCTGCGGGGATTACACCATACTGATAACTGACCGTAATTTAAATACTACTTTCTTTGATCCTGCTGGTAACGGCGACCTATCCTATACCAACACTTATTCTGATTCTTCGGTCACCTGAAGTATATATTCTAATCTTACCGGGTTTTGGAATAATTTCACACATTGTAACATATTATTCTAATAAAAAAGAACCCTTTGTGGTTATATGGGCATGGTATGAGCCATAATATCTGTTATTGGTTTCTAGGCTTTATTAGGCAATGCGTAACA

>b6e59923-10a4-4c8c-af3f-4cbdf17c54ce

TTGTATGATTTCAGTTACGTATTGCTTATTGTCACCGCCCACGCATTCATCGCGAGCAATTTTTTCATAGTAATACCAATTATGATTGGGGGTTTTCAGGAACATTCCTTTAATAATTGGCGCTCCCGATAGCATTTCCTCGAATAAATAACGTTAAGCTTCTGACTTCTACCACCCTCCTACTCCTACTGCTTGCATCATCATCAACTTTAGAAGCCGGTGCCGTACTGGTTGGACAGTCTACCCACCCCTAGCAGGCAATTTATCCCCAGCTCTGTAGATTTAACCATTTTTTTCACTACAGGCATTTCTTCTATTCTTGGAGCTATTAGCAGCATCGTAACTT

>b9de6024-2bce-4a17-9d50-39dba3881dd0

ACTTCGTTCAGTTACGTATTGCTATTGTCACCGCCCACGCATTCATCATAATTTTTTCTTAGTAATACAGTATGATTGGGGTTTCAGGAACTGACTTATTCCTTTAATAATTGGCAGCTCTGATCCTGGCATTTCCCTCGAATAAATAACATAAGCTTCTGACTTCACCGCCCTCCTACTCCTATGCATCTGACAAGCCGGTGCCTTTTTGGTTGGACAGTCTACCCACCCTAGCAGGCAATTTATCCCACCCACAAGGGCCTCTGTAGCAATACGTAACA

>3c7ab4f6-1e20-42d4-9dbd-31e63fac59cc

TTGTACTTCGTTCGATTGCGTATTGCTACCGCCACGCATTCATCATAATTTTTTCATAGTAATACCAATTATGATTGAGGGGTTTGAAGACGACATTCCTTTAATGAGTGGCGCTCCCGATATAGCATTTCCTCGAATATTCTGACTTCTACCCTCTACTCTATTTGCTTGCATCATCAACTTTAAAACGCCTTACCTTGGTTTAGTTCTACCCACCCCTAGCAGGCAATTTTATCCACCCAGGGAGCCTCTGTATGGGTCAACCATTTTTTTCACTACACAGGCATTTCTTCTATTCTTGGAGCTATTAACTTTATTACAATTGTAATGTATGACCATGACTCAATATCGAAA

>dd0beda5-b482-40fd-bd87-0c158fb9a385

TTGTACTTCGTTCCGTTTACGTATTGCTCCGCCCGCATTCATCATAATTTTTCATAGTAATACCGGTATGATTGGGGGTTTGGGAACTGACTTATTCCTTTAATAATTGGCGCTCCCGATATAGCATTTCCTCGAATAAATAACATAAGCTTCTGACTTCTACCACCCTCCCTACTCCTCACCTTGTCATCAACTTTAGAAGCCGGTGCCGGCACTGGTTGGACCGATGCCACCACTAACTTGAGCAATTTATCCCACCAGGAGCCTCTGTAGATTAACCATTTTTTTACTACATTTAGCAGGCATTTCTTCTATTCTTGGAGCTATTAACTTTATTGCAACAATTGCATAAATATGAAACCGCCAGCCATGACTCAATATCAA

>7ac1f36a-7a6d-4c1a-bdad-f5c1f6a37406

ATTGTACTTCGTTCAGTTACGTACTGTCACCGCCCACGCATTCATCATAATTTTTTTCATAGTAATACCAATTATGATTGGGGGTTTTTGGGAACTGACTTATTCCTTTAATAATGGCTCCCGATATAGCATTTCCTCGAATAAATAACATAAGCTTCTGACTTCTACCACCTCCCCTACTCCTACTGCTTGCATCATCAACTTTAGAAGCCGGTGCCGGCACTGGTTGGACAATCTACCCACCCCTAGCAGGCAATTATCCCACAGGAGCCTCTGTAGATTTAACCATTTTTTCACTACATTTAGCAGGCATTTCTTCTATTCTTGGAGCTATTAACTTTGTTACAACAATTGTAAATATGAAACCGCCAGCCATGACTCAATATCAAACTCCGTTGTTTGTGTGATCCGTCTAATTACTGCAGTCTGTCTTTTACTTTCTCTTCAGTTCTAGCTGCAGGGATTACCATACTATTAACTGACCGTAATTTAAATACTTTCTTTGATCCTGCTGGTGTGGCGACCTATCCTATACCAACACTTATTCTGATTCTTCGGTCACCCTGAAGTATATATTCTAATCTTACCGGGTTTTGGAATAATTTCACACATTGTAACATATTATTCCTAATAAAAAAGAACCCTTTGGTTATATGGGCATAGTATGAGCCATAATATCTGTGGTTTCCTAGGCTTTATTGTATAGGCCTCACATATTCACAGTAGGAATAGATGTAGATACTCGATGCATATTTTGTAGCTACCATAATCATTGCTATTCCCACTGGGTAAAAAGTATTTGTAGCTGATTAGCCACACTGCACGGCGGTAATATCAAACGGTCTCCCGCAATATTATGAGCCCTGGGCTTTATTTTCTTTTTTACCATGGGTGGACTATAAATTGTGTTAGCTAACTCATCATTAGATATTGTATTACATGATACATATTACTATGTAGTAGCCCACTTTCACTATGTGTTATCAATAGGAGCAGTATTTGCCATTATAGGGGATTTATTCACTGATTCCCGCTCTTTTCAGGCTATACTCTGTAATACGTAACT

>2cd033d4-975f-464f-8431-6c182226fdef

AGTGTTGTACTTCGTTCAGTTACGTACTACCGCCCACGCATTCAAATAACAATTTTTTCATAGTAAGCAATACCAATTATGATTGAGGGGTTTCTAAGGAACATTTACATTTCTTTAATAATTGGCGCTCCCGATATAGCATTTCCTCGAATAAATAACATAAGCTTCATGACTTCTACCACCCTCCCTACTCCTACCTTGCTTGCATCATCAACTTTAGAAGCCGGTGCCGGCACTGGTTGGACAATCTACCCACTAGCAGGCAATTTGTACCACCCAGGAGCCTCTGTAGATTTAACCATTTTTTCACTACATTTATGAGCATTTCTTCTATTCTTGGAGCTATTAACTTTATTACAACAATTGTAAATATGAAACCGCCAACCATGACTCAATATCAAACTCCGTTGTTTGTGTGATCCGTCCTAATTACTGCAGTCCTTGCTTTTACTTTCTCTTCCAGTTCTAGCTGCAAGGGATTACCATACTATTAACTGACCGTAATTTAAATACTGCTTTCTTTGATCCTGCTGGTGGCGGCGACCCTATCTATACCAACACTTATTCTGATTCTTCGGTCACCCTGAAGTATATATTCTAATCTTACCGGTTTTGGAATAATTTCACACATTGCCCAACATATTATTCTAATAAAAGACCCTTTGGTTATATGGGCATGGTATGAGCCATAATGTCTATTGGTTTCTAGGCTTTATTGTATGGGCAAATACATATTCACAGTAGAATAGATGTAGATACTCGTGCATATTTTACATCATGCCATAATCATTGCTATTCACTGGAGTAAAAGTATTTAGCTGATTAGCCACACTGCGGCAGTAATATCAAATGATCTCCCGCAATATTATGAGCCCTGGGCTTTATTTTCTTTTACCGTGGGTGGACTAACTTAAGGAATTGTGTTAGCTAACTCATCATTGAATATTGTATTACATGATACATACTATGTAGTAGCCCACTTTCACTATGTGTTATCAATAGGAGCAGTATTTGCCATTATAGAGTTTATTCACTGATTCCCGCTCTTTTCAGGCTATACTCTTGACCAAACATATGCAAATCACTTCACCATTATATTTGTCGGCGTAAATTTAACTTTCTTCCCCAACACTTCCTTGGCTTATCCGGAATACCTCGACGATACTCAGACTATCCAGACGCATATACTACATGAAATATCGTCTCATCTGTCGGTTCATTTATTTCACTTACAACAGTAGTCCTGATGGTTTTTATAATTTGACTTTCTCTTCAAAACGAAAAGTCTTAGCCATTGAGCAACTATCCATAATCTAGAATGATTATACGGCTGCCCTCCTCAGCAATACGTAA

>ee6178d2-f3e7-4519-b221-e874368bd4fc

GATGTCTTCGTTCAGTTACGTATTGCTGCTACTACATAGTATGTATCATGTAATACAATATCTAATGATGAGTTAGCTAACACAATTCCTGTTAGTCCACCCACGGTAAAGAAAAATAAAGCCAGGGCTCATAATATTGCAGAGATCATTTGATATTACCGCCGCCGTGCAATTAATGGCTAATCAGCTAAATACTTTTACTCCAGTGGGAATAGCAATGATTATGGTAGCTGATGTAAAATATGCACGAGTATCTACATCTATTCCCTACTGTGAATATGTGATGGGCTCATACAATAAAGCCTAGGAAACCAATAGATATTATGGCTCTTCACCATGCCATATAACCAAAGGGTTCTTTTTTATTAGAATAATATGTTACAATGTGTAAATTATTCCAAAACCCGGTAGATTAGAATATATACTTCAGGGTGACCGAAGAATCAGAATAAGTGTTGGTATAGGATATAGGGTCGCCGCCAGCAGGATCAAGAAAATTAGTATTTAAATTACGGTCGGATTAATAGTATGGTAATCCCTGCAGCTAGAACTGGAAGAAAGTAAAAGAAGGACTGCAGTAATTAGGACGGATCACACAAACAACGGAGTTTGATGTGAGTCATATTTGTGATTTCATATTTACAATTGTTGTAATAAAGTTAATAGCTCCAGAATAGGAAAATGCCTGCTAAAATGTAGTGAAAAAATGGTTAAATCTACAGAGGCTCCTGGGTAGAATAAATTGCCTGCTAGGGGTAGACTGTCCAACCGAATTGCCGGCACCGGCTTCTAAAGTTGATGATGCAAGCAGTAGGAGTAGGGAGGAATTGGTAGAAGTCAGAAGCTATGTTATTTATTCGAGGAAATGCTATATCGGGAGCGCCAATTATTAAAGGAATAAGTCAGTTCCCAAAACCCCCAATCATAATTGGTATTACTATGAAAAAAATTATGATGAATGCGTGGGAGCAATACGTAGCTT

>ebbc18ec-29cf-4a8e-987f-9b2f1510a32d

TTGTGCTTCGTTCAATTACCGTATTGCTCGCATTCATCATAATTTTTTTCATAGTAATACCAATTATGATTGGGGGTTTAGAGACGACTTATTCCTTTAATAATTGGCGCTCCCGATGTAACGTTTCCTCGAATAAATAACATAAGCTTCCTGACTTCTACCACCCTCCCTACTCCTACTGCTTGCATCATCAACTTTAGAAGCCGGTGCCGGCACTGGTTGGACAGTCTACCCACTAGCAGGCAATTTATCACCAAGGCCTCTGTAGATTTAACCATTTTTTCACTACATTTTAGCAGGCATTTCTTCTATTCTTGGAGCTATTAACTGTGCAACAA

>5ba24dc7-2c55-43f5-98a0-fa1afa0375a6

TTGTACTTCGTTCAGTTACATGCTGCATTCATCATATTTTCTTAGTAACTAGTGATTAGGGGTTTAGGAACATTTTATTCCTTTAATAATTGGCCTTCCGGTATAGCATTTCCTCGAATAATAACATAGCCACGGCTTCCACCACCCTCCCTACTCCTACTATTTATCATCAACTGACCGGTGCCAGCACTGGTTTGGACAGTCATACTTTATGAACAATTTATCACAGGAGCCTCTGTAGTTAGCATTTTTTATACATTTGCTTAGGCATTTCTTCTATTCTTGGAGCTATTAACTATTACAACAGTACCTTTAAATATGAGCATAATACTTGTACCAATATCACTTCCGTTGTTTGTGTGATCCGTCCTAATTACTGCAGTCCTTCTTTTACTTTCTCTTCCAGTTCGCTGCAGGGATTACCATACTAATGCTTGGCGTAATTTACTACTTTCTTTGATCCACCTGCTGGTGGCGGCGACCCTATCTACGCCATAACCTTGCACGTACGTTATTTAGTGCCTCTGAAGTATATATTCTAATCTTACCGGGTTGAATAATTTCACACATTGTAACATATTCTAATAAAAAGTAGTTAATATGGGCATGGTATGAGCCATATTATCTATTGGTTTCTAGGCTTTATTGTATGAGCCCATCATATTCACAGTAGGAATAGATGTGAATACTCGTGCATATTTTATCAACTACTATCATCATTATATTCCCACTGGAGTAAAAGTATTTAGCTAGCCACACTGCACGGCGGTAATATCAAATGATCTCCGCAATATTGTGAACACAGGCTTTATTTACCGTGGGTGGAATTGTGTTAGCTAACTCATCATTAGATATTGAGCAATACGTAA

>8885f46f-9f1a-49f4-af9e-9bb82d4078ea

AATGTACTTCGTTCAATGTTACGTATTGCTCTGGCAGTTTCATATTTACAATTGTTGTAATAAAGTTAATAGCGCTCCAAGAATAGAAAAATGCCTGCTAAATGTAGTGAAAAAATGGTTAAATCTGCAAGAGGCTCCTGGGTGGGATAAATTTGCTGCTAGGGGTGGGTAGACTGTCCAACCAGTGCCGGCACCGGCTTCTAAAGTTGATGATGCAAGCAGTAGGAGTAGGGAGGGTGGTAGAAGTCAAGCTTATGTTATTTATTCGAGAAATGCTATATCAGGGGAGCGCGCCAATTATTAAAGGAATAAAGTCAGTTCCCAAAACCCCCAATCATAATTGGTATTACTATGAAAAAAATTATGATGAATG

>f83a6b43-d13b-4a4c-a6ad-3c5148cbf5f6

ATTGTACTTCGTTCCAGTTACGTATTGCTAAAAATGGTTAAATCTACAGAGGCTCCTGGAGTGGGATAAATTGCCTGCTAGGAGTGGGTAGACTGTCCAACCAGTGCCGGCACCAAAGCTAAAAGTTGATGATGCAAGCAGTAGGAGTAGGGAGGGTGGTAGAAGTCAAGCTTATGTTATTTATTCGAGGAAATGCTATATCAGGAGCGCCAATTATTGAGAATAGTCCAGTTCCCAAAACCCCCGTCATAATTGGTATTACTATGAAAAAATTATGACTTCGAGAATTTCTCGTTCCGTGCCAACGTCATTGCCTACCTCAAGGCATGCGTGCTCTATGTTGCCAACAGCTACAGATGGGAGCCGGAGATGGATGACTTCATCCGCTGGAGCGAGCGTTACGACCTCTACTGCAAGATG

>57d0a176-716c-424d-b493-be4fc9b9569a

ATTGTACTTCGTTCAATTACATGTACGTATTGAGTCATAGCTGCTTGGTTTCATAGCTACAATTGTTGTAATAAGTTAATAGCTTTCAAGAATGAAGAAATGCCTGTAAATGTAGTGAAAAAAATGGTTAAATCTGAGGCTCCTGGGTGGGATGAATTGCCTGCTAGGGGTGGGTAGACTGTCAACCAGTGCCGGCACCGGCGCTTCTAAAGTTGATGATGCAAGCATTATAGGAGTAGGAATTGGTAGAAGTCAGAAGCTTATGTTGTTTATTCGAGGGAATCTTTATATCGGGAGCGCCAATTATTAAAAGGAATAAGTCAGTTCCCAAACCCCAATCATGAAGTGGTATTACTATGAA

>960800e7-3be5-4bb3-a9b5-0907275a8e34

TTATACTTCGTTCAGTTACGTATTGCTAGTAATACCAATTATGATTAGGGGGTTTGGGAACTGACTTATTCCTTTAATAATTGGCGCTCCCGATATAGCATTTCCTCGAATAAATAACATAAGCTTCTGACCTACCACCTCCATCTCCTACTGCTTGCATCATCAACTTTAAGCCGGTGCCGGCACTGGTTGAGACAGTCTACCCACCCCTGAGCAATTTATCCCACCCAGGAGCCTCTGTAGATTTAACCATTTTCACTACATTTAGCAGGCATTTTCTTCTATTCTTGGGCTATTCTTTATTACAACAATTGTAAAATATAGAAACCGCCAGCCATGACTCAATATCAAGCTAAATTTGTTTGTGCTGATCCGTCCTATTACTGCAGTCCTTCTTTTACTTTCTCTTCCAGTTCTAGCAGGGATTACCAACCACTATTAACCATGATATAATTTAAATACTACTTTCTTTGAGTCTGCTGGTGGCGGCGACCCTATCCTA

>ead2d1c2-6853-44e6-9cd9-b6c143fdfed2

ATTGTACTTCGTTCAGTTACGTATTGCTAATACCAATTATGATTGGGGGTTTGGGAACTTGACTTATTCCTTTAATAATTGGCGCTCCCGATATAGCATTTCCTCGGAATAAATAACATAAGCTTCTGACTTCTACCACCCTCCCTACTCCTACTGCTTGCATCATCAACTTTAGAAGCCGGTGCCGGCGCTGGTTGGACAGTCTACCCACCCCTAGCGAGCAATTTATCCCACCCAGGAGCCTCCTGTGAGTTAACCATTTTTTCACTACCATTTAGCAGGCATTTCTTCTATTCTTGGAGCTATTAACTTTATTACAACAATTGTAAATATGAAACCGCCAGCCATGACTCAATATCAAACTCCGTTGTTTGTGTGATCCGTCCTAATTACTGCAGTCCTTCTTTACTTTCT

>7a76579c-a55c-4d0e-b80f-cd7cd24199cf

AATGTACTTCGTTCAGTTACGTATTGCTATTACATTAATATAGATAAAAGGCATATTTGGTAAATATGAGCTTTCATAGCTCTAATGAGTCAAGAAATCATTTGTTTTAATTAAACTATATACCAATTCAGCCCAATCTAATCCTTTTTGGGACCACTCGTAGGCTAACCCTAAAGCCAGAATAATAAGTAGGGTAAAAATTATATTTATTGTTAGAGTTAAATTATTTGTTTGGGTTGCCCATGGTAGGGGTAGAAGTAGGGCAATTTTCAAGATCAAATGGGAGAAATGTGATGGCGATTAGGAAAAATTTTATGGAGAATGGTAAGTGGGCGGAGGTTGTAGGATCAAATCCGCACTCGTAAGGGTTAGCGCTTTTCTGTATATAATATTTAATTGTGGAAGTCAAAATGTAATTGTGATAAGAAGTAAAAGGCCAAAGTAATGTTAGTCATTAGGGCTAAAATTAAATTTATAATACTCTCTCTCGAATCATTCGAGGCCCGTTGATTGGAAGTCAATAATACTTTTTATACTAAGAGAGTAAGAGCCTCATCAATAGATGGAAATATAGAGGAAAAGTCATACTACATCTACGAAATGTCAGCTATCGTAGCGGCTTCGAAGCCGAAATGGTGGCTATGATGTGAAGTGATATAATTGTTGGCGAAAGTAACAGGTGGTGAGGAAAGTGGTTCCGATAATTACGTGAAGGCCATGAGAAGCCTGTAGCCATAAAAAATGTGGATCCATATACTCCGTCGGAGATAGTAAATGGGGCTTCAGAATATTCTGATAATTGTAGGCGAATTGAAGTAGATCCCTAATATAATGGTTAGGAGTAGTGCTTGGGTTGATTCTTTTCGGTTAGCTTCTATGAGGCTGTGATGTGCTCATGTAATTGCTTAACTCTGATGCTAGTAGTGCGGTTGTATTTAAGAGAGGGACTTCTATTGGGTTGGGGAGGTAATGCCTGTAGGTGGTCATAGTCCCCTGTTTGTGGGATTGGGGCTAGACTAGAATGATAAAATGCTCAGAAGAAACCTGCAAGAAGAAAATTTCTGAAATAATAAATAGAATTATTCCGTATCGTAGGCCTTTTTGGACAGGTGCAGTATGGTGGCCTTGATATGTACTTTCTCGTACTACATCGTCATCATTGAAATATTGTTATAGAACTAGCTAGTAGACCTGCAGTGAAGCGGTGTGGTAAAAGTGGAATCATATAACTAGGCCAGATGAGAAGAAAATAATAAATACCTGTTTAGTGGTCAAGGCCATGTTGACCATATGATGGGCATAGTTTTGAGCGAGTCAAATTTATGAATTATCATGTAAGTACAGGCTTACTAAGAGGGTAAATACATAGGCTTGAATTAAGGCCACACCCAACTCTAGGGTAATTAATAAAATAATAATAATGATAGTGATTGTGGAAGTGGAGAGGTAGAATTGATAGAAGAGTTAATGTTGTATCCCCAAGTAAATGCATTAATAGGTGACCTGCTGTAATATTGGCTGTTAATCGTACGGCTAAGGCTACAGGTTGAATGAATAGACTAATTGTTTCAATAATAATTAATATAGGGATTAGTGGAATTGGTGTTCCTTGTGGTAAAAAATGAGCGAGGGATGATTTTGTTTTAAATCGGAGGCCTATTAGTACAGTTGCTGCTCATAGAGGAATAGCTATGCCTAGATTTATTGACAGTTGGGTGGTTGGTGTAAATGCATATGGTGTAAGTCCGAGAATGTTATTTAGGGCAATAAAAGAAATTAGGGCTAGAATTATAAGGGATCAGGTTCGCCCTTTAATTGGTGTGGGTTAATTATTATTTGTTTAAGTGTTAGTTGAATTAGCCATTGTTGAATTGAAGAGAGTCGGTTGTTGAATAAGTTTTTGGAGGATAAAATTAATGTAGTGGGGAGTGCAATAATTAAAAATACTAAGGGTACTCCTAGTATTGTTGGAATATTGAATGAGGCAAATAGATTTTGGTTCATTTTAGTTCTCAAGTTGTTTTATGTTTTTGTGTTTCTACTAATTTTGGTAGTGGGTAATAATGGAAAGTAAAGTTCAGTATTTTCAATTGTATAATATAAAATAAGGTAACAATTATAGATATAATTACTATTGGTCACGGTGAAATATTTAGTTGAGGCATTCACTATAGAGAGTTCTCTCAATCTTTAACAAAAAGGTTAATGCTAAGTTAGCTTTACGATACAATATATAAGTATGAAGCTCTTACTTCGAAATCTTGGAAATAAATGAATTCTAGAACAATAGGTATAAAGCTGTGATTGGACCCGCAAATTTCGAGCATTGTCCATAAAATAGGCCCTGGTCGTATAGAGGCTAGTATGGCTTGAGTTTAAACGTCAGGAATTGCATCTGTTTTTACGCCTAGTGATGGTACGGCTCATGAGTGTAAGACGTCTTGTGATGAGATTAATATGCGAATATCCGCTTCTATAGGTAAAGTTGTTCGGTTATCAACTTCCGAGGTCGAAATTCCCTGGCTCAAGGAAATATGTTGGCATAATGTAAGAGTCAAATACTAAGTCTTCATAGTCGGAATATTCATAGGTTCAGTATCATTGGTGACCAATTGCTTTAAGGGTTAAATAAGGTTTATTAAATTCGTCTGTCATATATAGAATACGCAGTGATGGGAGGGCAATTGTAATTAGAATCAGTGCAGGGGAGAATAGTTCAGATTATTCGATCTCTTGAGCATTTATGGTGCTAGTATGAGTGAGTTTTGTGGTAAGTAAAGGGAAATAATATATAAGACTAAGGAACCCAATCAGGAAAATAATTATGAGTGCATGGTCGTGGAAAGCAATAAGTTCTTCTATGATAGGTGATGTGGCATTTTGTAAACCTAGTTGAGCTGGTGTTGCTATTAAGATATATAGATATTTAGTCTATAATTTAACTTTGACAAAGTTATGTAATTATTTTACTAATATCTTATTGAAAAAAGTCATAGGGTATATGGGATTGGCTTGGAAACCAATTTTGGGGGTTCAAATCCTTCCTTTTTCGTCTAGGATTTAACGTAAGTTGCCTCTTCGAATGTGTGGTAAGGAGGAGGGCAGCCGTATAATCATTCTAGATTGGTGGATAGTTGCTCAATGGCTAAGACTTTTCGTTTTGAAGAGAAAGCTTCTCAAATTATAAAAACCATCAGGACTACTGCTGTAAGTGAAATAAATGAACCGATAGATGAGACGGTATTTCATGTAGTATATGCGTCTGGATAGTCTGGTATCGTCGAGGTATTCCGGATAAGCCAAGGAAGTGTTGTGGGAAGAAAGTTAAATTTACGCCGACAAATATAATGGTGAAGTGAAAGTTTTGGCATATGTTTGGTCAAGAGTATAGCCTGAAAAGAGCGAATCAGTGAATAAATCCCCCTATAATGGCAAATGCTGCTCCTATTGATAACACATAGTGAAAGTGGGCTACTACATAGTATGTATCATGTAATACAATATCTAATGATGAGTTAGCTAACACAATTCCTGTTAGTCCACCCACGGTAAAAAGAAAAAATAAACCCAGGGCTCATAATATTGCAGGAGATCATTTGATATTACCGCCGTGCAGTGTGGCTAATCAGCTAAATACTTTTACTCCAGTGGGAATAGCAGATTATGGTAGCTGATGTAAAATATGCACGAGTATCTACATCTATTCCTACTGTGAATATGTGATGGGCTCATACAATAAAGCCTAGAAAACCAATAAGATATTATGGCTCATACCATGCCCATATAACCAAAGGGTTCTTTTATTAGAAATAATATGTTACAATGTGTGAAATTATTCCAAAACCCGATAGAATATATACTTCAGGGTGACCGAAGAATCAAGTAAAGTGTTGGTATAGGATAGGGTCGCCGCCACCAGCGAGGATCAAAGAAAGTAGTATTTAAAATTACGGTCAGTTAATAGTATGGTAATCCCTGCAGCTAGAACTGGAAGAGAAAGTAAAGAAGGACTGCAGTAATTAGGACGGATCACACAAACAACGGAGTTTGATAAAACGAGTCATGGCTGGCGGTTTCATATTTGAATTGTTGTAATAAAGTTAATGACTCCAGAATGAAGAAATACCTGCTAAATGTAGTGAAAAAATGGTTAAATCTACAGAGGCTCCTGGGTGGGATAAATTGCCTGCTAGGGGTGGGTAGACTGTCCAACCGGTGCCGGCACCGGCTTCTAAAGTTGATGATGCAAGCAGTAGGGAGTAGGGAGGGTGGTAGAAGTCAGAAGCTTATGTTTTATTTATTCGAGGAAATGCTATATCGGGAGCGCCAATTATTAAAGGAATAAGTCAGTTCCCAAAACCCCAATCATAATTGGTATTG

>c4026a41-9497-4ad4-81fd-cfc9e26d05cb

TTGTACTTCGTTTCAGTTACGTATTGCTATACCAATTATGATTGGGGTTTTGGGAACTGACTTATTCCTTTAATAATTAGCGCTCCCGATATAGCATTTCCTCGAATAAATAACATAAGCTTCTGACTTCTACCACCCTCCCTACTCCTACTGCTTGCATCATCAACTGTGACCGGTGCCGGCACTGATTTGGACAGTCTGCAACCTTCTAGCAGGCAATTTATCCCACCCAGGAGCCTCTGTAGATTTAACCGTTTTTCACTACATTTTAGCAGGCATTTCTTCTATTCTTGGAGCTATTAACTTTATTACAACAATTGTAAATATGAAACCGCCAGCCATGACTCAATATCAAACTCCGTTGTTTGTGTGATCTAATCCTAATTACTGCAGTCCTTCTTACTTTCTCTTCCAGTTCTAGCTGCAGGGATTACCATACTATTAACTGACCGTAATTTAAATACTACTTTCTTTGATCCTGCTGGTGGCGGCAGGCTATCTATACCAACACTTGTTACCAGTTCTTCGGTCACCTGAAGTATATATTCTAATCTTACCGGGTTTGGAATAATTTCACACATTGCTGTATATTATTCTAATAAAAAAGAACCCTTTGGTTATGGGCATGGTATGAGCCATAATATCTGTGGTTTCCTAGGCTTTATTGTATGAGCCCATCACGCATATTCACAGTAGGAATAGATGTAGATACTCGTGCATATTTTACATCAGCTACCATAATCATTGCTATTCCCATAGATAAAAGTATTTAATGATTAGCCACACTGCACGGCGGTAATGTCAGATGATCTCCCGCAATATTATGAGCCCTGGGCTTTATTTTTCTTTTTACCGTGGGTGGACTAACAGGAATTGTGTTAGCTAACTCATCATTAGATATTGTATTACATGATACATACTATGTAGTAGCCTTTCCTGCTGTGTTATCAATAGGAGCAGTGTGCCATTATAGGGGGATTATTCACTGATTCCCGCTCTTTTCAGGCTATACTCTTGACCAAACATGTGCCAAAATCCACTTCACCATTATATTTGTCGGCGTAAATTTAACTTTCTTCCCACAACGCTTGCTTCCTTGGCTTATCCGGAATACCTCGACGATACTCAGACTATCCAGACGCATATACTACATGAAATATCGTCATCTATCAGTTCATTTATTTCACCCGGTAGTCACAAGTGGTTTTAGCAATACGTAACT

>a5ae9eb9-a89f-462f-9fbc-fbe4084e94bc

TGTGCTCACTTCGTTCAGTTACGTATTGCAGAATAGAAGAAATGCCTGCTAAATGTAGTGAAAAAATGGTTAAATCTACAGGCTTCCTGGGTGGGATAAAATTGCCTGCTAGGGGGTGGGTAGACCTGTCAACCAGTGCCGGCACCGGCTACCAAGTTGATGATGCAAGCGATAAAATGAGGAAGGTAGTGAAGTCAGAAGCTTATGTTATTTATTCGAGGAAATGCTATATCAGGAGCGCCAATTATTAAAGGAATAAGTCGGATTCCCAAAACCCCAATCATAATTGGAGCAATACGTAACTA

>a5ca124a-0536-4f80-aebb-89f3f856e27a

TTGTACTTCGTTCAGTTACGTGCTCAATTATGATTGGGGGTTTGGGAACTGACTTATTCCTTCCAACAATTGGCGCTCCCGATATAGCATTTCCTCGAATAAATAACATAAGCTTCTGACTTCTACCACCCTCCCTACTCCTACACTTGCATCATCAACTTTAGAAGTGGCGGTGCCGGCACTGGTTGAAAAGGATCTACCCACCCTAGCAGGCAATTTATCCCACCCAGGAGCCTCTGTAGATTTAACCATTTTTTCACTACATTTAGCAGGCATTTCTTCTATTCTTGGAGCTATTAACTTTATTACAACAATTGTAAATATAGAGCCGCCAGCCATGACTCAATATCAAACTCCGTTGTTTGTGTGATCCGTCTAATTACTGCAGTCCTTCTTTTACTTTCTCTTCCAGTTCTAGCTGCAGGGATTACCATACTATTAACTGACCGTAATTTAAATACTACTTTCTTTGATCCTGCTGGTGGCGGCGACCCTATCCTATACCAACACTTATTCTGATTCTTCAGTCACACAAGAGTATATATTCTAATCTTACCGGGTTTTGGAATAATTTCACACATTGAGCAATACGTAACTA

>4c053366-4c29-4173-a402-edfdea28c3a0

GATGTGCTTCGTTCAGTTACTATTGCTGCTAGAACTGGAAGAGAAAGTAAAGAAGGACTGCAGTAATTAGGACGGATCATTTGGCTCGCGGAGTTTGATATTGAGTCATGGCTGGCGGTTTCATATTTACAATTGTTGTAATAAGATTAATAGCTCAAGAATAGAAGAAATGCCTGCTAAATGTAGTGAAAAAATGGTTAAATCTACAGAGGCTCCTGAGTGGGATAAATTGCCTGCTAGGGGTGGGTAGACTGTCAACCAGTGCCGGCACCGGCTTCTAAAGTTGATGATGCAAGCAGTAGGAGTAGGGAGGGGTGGTAAGTCAGAAGCTTATGTTATTTATTCGAGGAAATGCTATATCAGGAGCGCCAATTATTAAAGGGAGTCAGTCAGTTCCCAAAACCCCAATCATAATTGAGTA

>c99ada11-82f8-4b61-8bbb-ec570ff1c4dc

AAATGTACTGCTTCGTTCAGTTACGTATTGCTGATTAGAGGTTTTGGGAACTGACTACTTATTCCTTTAATAATTGGCGCTCCCGATATAGCATTTCCTCGAATAAATAGCATAAGCTTCTGACTTCTGCCGCCTCTACTCCCTGCACCAGCATCATCAACTTTAGAAGCCGGTGCCGGCACTGGTTGGACGATCTACCCACCCTGGCAGGCAATTTATCACAGGAGCCTCTGTAGATTTAACATTTTTCCACTACATTTCGCTTGAGCATTTCTTCTATTCTTGGAGCTATGCTTTATTACAACAATTGTAAATATGAAACCGCCAGCCATGACTCAATATCAAACTCCGTTGTTTGTGTTAGTCTAGTACCACCCGGTCAACTTCTTTTACTTTCTCTTCCAGTTCTAGCTGCAGGGATTACCAA

>2600eadd-1422-4c41-af41-63c453486e8c

AACTTCGTTCAGTTACGTATTGCTGTTTTGGGAACTGACTTATTCCTTTAATGATGGCGCTCCCGATAGCATTTCCTCGAATAAATAACATAAGCTTCTGACTTCTACCACCCTCCTACTCCTACTTTGCATCATCAACTTTAGAAGCCGGTGCCGGCACTGGTTGGACAGTCTACCCACCCCTAGCAGGCAATTTATCCCACCCAGGAGCCTCTGTAGATTTAACCATTTTTTCACCTACATTTAGCAGGCATTTCTTCTATTCTTGGAGCTATTAACTTTATTACAACAATTGTAAATATGAAACCGCCAGCCATGACTCAATATCAAACTCCGTTGTTTGTGTGATCCGTCCCCTAATTACTGCAGTCCTTCTTTTACTTTCTCTTCCAGTTCTAGCTGCAGGGATTACCATACTATTAACTGACCGTAATTTTAAATACTACTTTCTTTGATCATGCTGGTGGCAGCGACCATATCACTATGCCAACACTTGTTACGTTCTTCGGTCACCCTGAAGTATATATTCTAATCTTACCGGGTTTTGGAATAATTTCACACATTTTAACATATTATTCTAATAAAAAAAAATTCCTTGGTTATATGGGCATGGTATGAGCCATAATATCTATTGGTTTCTAGGCTTTATTGTATGAGCCCATCACATATTCACAGTAGGAATAGATGTAGATACTCGTGCATATTTTACATCAGCTACCATAATCATTTGCTATTCCCACTGGAGTAAAAGTATTTAGCTGATTAGCCACACTGCCTGGCAGTAATATCAAATGATCTCCCGCAATATTATGAGCCCTGGAGCTTTATTTTCTTTTACCGTGGGTGGACTAACAGGAATTGTGTTAGCTAACTCATCATTAGATATTGTATTACATGACCATACTATGTAGTAGCCCACTTTCACTATGTGTTATCAATAGAGACGTATTTGCCATTATAGGGATTTATTCACTGATTCCACTCTTTTCGAATATACTCTTGACAGGCATATTGGCGAAAGTCACTTCACCATTATATTTGTCGGCGTAAATTTAACTTTCTTCCCACAACACTTCCTTGGCTTATCCGGAATACCTCGACGATACTCAGACTATCCAGACGCATATACTACATGAAATATCGTCTCATCATATCGGTTCATTTATTTCACTTACAGCAGTAGTCCTGATGGTTTTTATAATTTGAGAAGCTTTTTCTCTTCAAAACGAAAAGTCTTAGCCATTGAGCAACTATCCACCAATCTAGAATGATTATACGGCTGCCCTCCTCCTTACCAATGCCAACATCAATGAGGCATATTACTTCAACGGTCACTGGGAGACCTTCGGAGGTTTGAACTGGCAGGCAGCAAAGCGTCTGGCTCTCGGCTGCACCGTAGTCAACTTCCTCAACCAGACTGGTGCCAAGGGTAGTATCGCAGGTGCCGAGCTGATTACCAAGGATGAGGCCAAAGGAATCAAGAACCAGATAATGACGGGTAGTTATCTCCGTCCATTCACGGTAGAATTCACCGCTTCGCTGAAGTTCTAATTAGTTCAAACACATAATAAGATAAGGCAGTACTATATAATAATGTATAGTGCTGCCGCTTTTTTTTCTTGTTTTTGGTTTGGAAATGATTGGAAAAATATGGCTTTTGGTT

>011738ab-2e5e-4a0d-9ba7-48172e47a842

GAGCATACTTCGTTCAGTTACGTATTGCTGATGTAAAAAACAACGATGCTCGCTATTCCTACTGTAGACCATGTGATGGGCTCTTCTACAATAAAGCCTAAGGAAACAAGCCGTATTATAGCGCATATACATGCCCATACCAACAGGGTTCTTTTTATGCGAATAATATGTTACAATGTGTGAAATTATTCCGAAACCCGGTAAGATTAGAATATATACTTCAAGGTGACCGAAGAATCGAATAAGTGTTGGTATGAGATAGGGTCGCCACTTCAGCAGGATCAAAAGTAGTATTTAAATTACGGTCCAGTTAATGATGTAAACGTCCTATAGCTAGAACTGGAAGAGAAAGTAAAAGGGACACGTGGTGGGACGGATCACGCAAACAACGGAGTTTGATATTGAGTCATGGCTGGCGGTTTCATATTTTACAATTGTTGTAATAAGTTAATACATCAAGAATAGGAAGAAATGCCTGTAAATGTAGTGAAAAAATGGTTAAATCTTTCTGCAGAGCTCTGGGTGGGATAAATTGCCTGCTAGGGGTGGGTAGACTGTCCAACCAGTGCCGGCACCGGCTACAAAGTTGATGATGCAAGCAGTAGAGTAGGGAGGGTGGTAGAGATGAAGCATGTTATTTATTCAGAGAAATGCTATATCAGGAGCGCCAATTATTAAAGGAATAGTCAGTTCCAAAACAGCAATACGTA

>00c1be4d-f5ab-4929-a2b9-dde0103e01cf

AGTGTACTTCGTTCAGTTACGTATTGCTCTGACTTATTCCTTTAATAATTGGCGCTCCCGATATAGCATTTCACTCGAATAAATAACATAAGCTTCTGACTTCTACCACCCTCCCTACTCCTACTGCTTGCATCATCGCTTTGTAGAAGCCGGTGCCGGCACTGGTTGGACAGTCTACCCTCCTAGCAGGCAATTTATCCCACCCAGAACCTCTGTAGATTTAACCATTTTTTCACTACATTTAGCAGGCATTTCTTCTATTCTTGGAGCTATTAACTTTATTACAACAATTGTAAATATGAAACCGCCAGCCATGACTCAATATCAAGAAATCCGTTGTTTGTGTGATCCGTCCTAATTACTGCAGTCC

>f6b72196-982b-4a1e-b92f-c6851a590777

AATGTACTTCGTTCAGTTACGTATTGCTTAATCCCTGCAGCTAGAACTGGAAGAAGAAAGTAAAAGAAGGACTGCAGTAATTAGGACGGATCACACAAACAGCGGAGTTTGATATTGAGTCATGGCTGGCGGTTTCATATTTACAATTGTTGTAATAAAGTTAATAGCTCAAGAATGAAGAAATGCCTGCTAAATCATAGGCAATGGTTAAATCTACAGAAGGCTTCCTGGGTGGGATAAATTGCCTGCTACAGGGTGGGTGAATATTGTCCAGCCAGTGCCGGCACCGGCCCTAAAGTTGATGATGCAAGCAGTAGGAGTAGGGGGAGGGTGGTAGAAGTCAGAAGCATATTTATTTATTCGAGGAAATGCTATATCGGGAGCGCCAATTATTAAAAATTAAGTCCA

>fb3e1596-4115-44ce-ac74-beaa30d9780b

ATTGTCGCCTTCGTTCAGTTACGTATTGCTCTTATTCCTTTAATAATTGGCGCTCCCGATATAGCATTTCCTCGAATAAATAACATAAGCTTCTGACTTCTACCACCCTCCTACTCCACCTGCTTGCATCATCAACTTTAAAGCCGGTGCCAGCACTGGTTGGACAGTCTACCCACCTAGCAGGTATCCCACCCAGGAGCCTCTGTGGGGTTGCCATTTTTTTTCACTACATTTTAGCAGGCATTGCTTCTATTCTTGGAGCTATTAACTTTATTACAACAATTGTAAATATGAAAGAGCCGCCAGCCATGACTCAATATCAAACTCCGTTGTTTGTG

>59e28ce6-8adf-486b-95af-e4236df1f822

TTGTACTTCGTTCAGTTTACATCATTGCGCCATTCCTTTAATAATTGGCACTCCCGATATAGCATTTCCTCGAATAAATAACATAAGCTTCTGACTTCTACCACCTCCTACTCCTACTGCTTGCATCATCAACTTTAGACGGTGCCGGCACTGGTTGGACAAGTCTACCCACCCCTAGCAGGCAGGTTATCCCACCCAGGGCCTCTGTAGATTTAACCATTTTTTCACTACATTTAGCAGGCATTTCTTCTATTCTTGGAGCTATTAACTTTATTACAGCAATTGTAAATAATAAACCGCCAGCCATGACTCAATATCAAACTCCGTTGTTTTGTGTGATCCGTCCTAATTACC

>b32535ca-61be-44a1-bd30-1e2061e55ff7

TTGTACTTCGTTCAGTTACGTATTGCTGTAGCTGATGCCAAATAGCCACGAGTATCTACATCTATTCCTACTGTGAATATGTGATGGGCTCATACAATAAAGCCTAGGAAACCAATAGATATTATGGCTCATACCATGCCCATATAACCAAAGGGTTCTTTTTTATTAGAATAATATGTTACAATGTGTGAAATTATTCCAAAACCCGGTAAGATTAGAATATATACTTCAGGGTGACCGAAAGATCAGAATAAGTGTTGGTATAGGATAGGGTCGCCGCCACCAGCAGGATCAAAAGAAAGTAGTATTTAAATTACGGTCAGTTAATGGTATGGTAATCCCTGCAGCTAGAACTGGAGAGAAAGTAAAAGAAGGACTGCAGTAATTGGGACGGATCACACAAACAGCGGGTTTGATATTGAGTCATGGCTGGCGGTTTCATATTTACAATTGTTGTAATAAAGTTAATGGCTCAAGAATAGAAGAAATGCCTGCTAAATGTAGTGAAAAATGGTTAAATCTACAGAGGCTCCTGGGTGGGATAAATTGCCTGCTAGGGGTGGGTAGACTGTCCAACCAGTGCCGGCACCGGCTTCTAAAGTTGATGATGCAAGCAGTAAGGAGTAGGGAGGGTGGTAGAAGTCAGAAGCTTATGTTATTTATTCGAAGAAATGCTATATCGGGAGCGCCAATTATTAAAGGAATGA

>d1a58e76-2180-44b8-93cf-62cfa2d4bc81

ATTGTACTTCGTTCAATTACGTATTGCTTATGTGGTAATCCCTGCAGCTAGAACTTGGAAGAGAAAGTAAAAGAAGGACTGCAGTAATTAGGACGGATCACACAAACAACGGAGTTTGATATTGAGTCATGGCTGGCGGTTTCACTATTTACAATTGTTGTATAAAGTTAATAGCTCCAAGAATAGAAGAAATGCCTGCTAAATGTAGTGAAAAAATGGTTAAATCTACAAGGCTCTGGGTGATAAATTGCCTGCTAGGGGTGGGTAGACTGTCCAGCCAGTGCCGGCACCGGCTTCTAAAGTTGATGATGCAAGCAGTAGGAGTAGGGAGGGTGGTAGAGAGTCAGAAGCTTATGTTATTTATTCGAGGAAATGCTATATCGGGGCGCCAGTG

>bc5e2882-5330-420d-819f-4b020adb0d97

GGTATGCTTCGTTCGGTTACGTGTGCTTTTGTAGTCAGCGCTCGATACAGCATTTCCTCGAATAAATAATATAAGCTTCTGACTTCTACCACCTCCTACTCCTACTGCTTGCATCATCAACTTTAGAAGCCGGTGCCGGCACTGGTTGGACAGTCTCCACCCCTACAGCAGGCAATTTATCCCACCCGGAGCCTCTGTAGATTTGTATTTTTTCACTACTTTAGCAGGCATTTCTTCTATTCTTGGAGCTGGCATTACAGCAATTGTAAACAAACCGCCAGCCATACGACTCAATATCAAACTCCGTTGTTTGTGATCCGTCCCTAGTACTGCAGTCCTTCTTTTACTTTCTCTTCCAGTTCTAGCTGCAGGGATTACCATACTATTAACTGACCGTAATTCCAGCCATACTTTGATCCTGCTGGTGGCGGCGACTATCCTATACCAACACTTATTCTGATTCTTCGTGCA

>6ed43b27-56c6-4b29-b576-7a8632eb06c3

AGTGTACTTCGTTCCAGTTACGTATTGCTCCCGATGTAGCATTCCTCGAATAAATAACATAAGCTTCGACTTCTACCACCCTCCCTCTCCTACTATATGCATCATCAACTTTAGAAACCGGTGCCAAACATGGTTGAACCAGTCTACCCACCCCTAGCGGGCAATTTATCCCGCAAGGCCTCTGTAGGTTATACATTTTTTCACTACATTTAGCAAGCATTTCTTCTATTCTTTGGAGCCTAACTATTGTAATTGTAAATATGAAACCGCCAGCCATGACTCAATATCCAAACTCCAGGTGTTTGTGTGATCCATCCTAATTACTGCAGTCCTTCTTTACTTTCTCTTCCAGTTCTATAACTACAGGTGCCATACTATTAACTGACCTATTAATTTAAATACTATATAACTTTGATCCTGCTGGTGGCGGCGACCCTATCCTATACCAACACTTATTCGGATTCTTCGGTCACCCTGAAGTATATATTCTAATCTTACTATAGGTTTGGAATAATTTCACACATTGTAACATATTATTCTAATAAAAAAAGACCCTTTGGTTATGGGCATGGTATGAGCCATAATATCTATTGGTTTCCTAGAGCTTTGTGTATGAGCCCATCACATATTCACAGTAGGAATAGATGCTGAAATACTCGATGCATATTTTACATCAGCTACCATAATCATTGCTATTCCCACTGGAGTAAGTATTCTTCGATTAGCCACACTGCACGGCAGTAATATCAAATGATCTCCACAATATTATGAGCCCTGGGCTTTATTTTCTTTTACCGTGGGTAGCAACCACACAACA

>75761bb7-155c-448f-99a9-7a79a7a4a7fb

GATACATGCCGTTCAGTTACGTATTGCTAATATTGCGGGAATCATTTGATATTACCGCCGTGCAGTATTCAGCTAATCAATAAATACTTTTACTCCAGTGGGAATAGCAATGATTATGGTAGCTGATGTAAAATATGCACAGTATCTACATCTATTCCTACTGTGAATATGTGATGGGCTCATACAAATAAAGCCCTAGAAACCAATAGATATTATGGCTCATACCATGCCCATATAACCAAAGGGTTCTTTTTTTATTAGAATAATATGTTACAATGTGTGAAATTATTCCAAAACCCGGTAAGGTTAGAATATATACTTCAGGGTGACCGAAGAATCAGAATAAGTCTTGGTATAGAGGATAGGGTCGCCGCCACCAGCAAGGATCAAAGTAGTATTTAAATTACGGTCAGTTAATAGTATGGTAATCCCTGCAGCTAGAACTGGAAGAGAAAGTAAAGAAGGACTGCAGTAATTAGGACGGATCACACAAACAACGGAGTTTGATGTTGAGTCATGGCTGGCGGTTTCATATTTACAATTGTTGTAATGAGTTAATAGCTCCAAGAATAGAAGAAATGCCTGCTAAATGTAGTCGAAAAAATGGTTAAATCACAGAGGCTCCTGGGTGGGATAAATTGCCTGCTAGGGGTGGGTAGACTGTCCAGCCAGTGCCGGCACCGGCTTCTAAAGTTGATGATGCAAGCATTGGGAGTAGGGAGGAGTGGTAGAAGTCGAAATATGTTGTTATTCGAGGAAATGCTATATCAATACGTGAAAAAGCA

>7814f84b-63d8-42e9-adae-449ca94c6407

TTGTACTTCGTTCAGTTACGTATTGCTATATAGCATTTCCTCGAATAAATAACATAAGCTTCTGACTTCTACCACCTCCCTACTCCTACTGCTGCATCATCAACTTTAGAAGCCGGTGCCGGCACTGGTTGGACAGTCTACCCACCTTGGGCAATTTATCCCACCCAGGAGCCTCTGTGAATTTAACCATTTTTTCACTACATTTAGCAGGCATTTCTTCTATTCTTGGAGCTATTAACTTTATTACAACAATTGTAAATATGAAACCGCCAGCCAAAAGAGCTCAATATCAAACTCCGTTGTTTGTGTGATCCGTCCTAGTACTACCCAGTCCCTTCTTTTACTTTCTCTTCCAGTTCTAGCTGCAGGGATTACCATACTGTTGTGACCGCTAATTTAGAAATACTA

>a8fe4925-9695-4178-af24-e0036a7322b1
[truncated: 4,994,118 more chars]
